# Proteomic and phosphoproteomic profiling of shammah induced signaling in oral keratinocytes

**DOI:** 10.1038/s41598-021-88345-x

**Published:** 2021-04-30

**Authors:** Shankargouda Patil, Mohd Younis Bhat, Jayshree Advani, Sonali V. Mohan, Niraj Babu, Keshava K. Datta, Tejaswini Subbannayya, Pavithra Rajagopalan, Firdous A. Bhat, Nezar Al-hebshi, David Sidransky, Harsha Gowda, Aditi Chatterjee

**Affiliations:** 1 Department of Maxillofacial Surgery and Diagnostic Sciences, Division of Oral Pathology, College of Dentistry, Jazan University, Jazan, Saudi Arabia; 2 Institute of Bioinformatics, International Technology Park, Bangalore, India; 3 School of Biotechnology, Amrita Vishwa Vidyapeetham, Kollam, India; 4 Manipal Academy of Higher Education, Manipal, India; 5 Department of Oral Health Sciences, Maurice H. Kornberg School of Dentistry, Temple University, Philadelphia, USA; 6 Department of Otolaryngology-Head and Neck Surgery, Johns Hopkins University School of Medicine, Baltimore, MD USA

**Keywords:** Proteomics, Cancer, Oral cancer

## Abstract

Shammah is a smokeless tobacco product often mixed with lime, ash, black pepper and flavorings. Exposure to shammah has been linked with dental diseases and oral squamous cell carcinoma. There is limited literature on the prevalence of shammah and its role in pathobiology of oral cancer. In this study, we developed a cellular model to understand the effect of chronic shammah exposure on oral keratinocytes. Chronic exposure to shammah resulted in increased proliferation and invasiveness of non-transformed oral keratinocytes. Quantitative proteomics of shammah treated cells compared to untreated cells led to quantification of 4712 proteins of which 402 were found to be significantly altered. In addition, phosphoproteomics analysis of shammah treated cells compared to untreated revealed hyperphosphorylation of 36 proteins and hypophosphorylation of 83 proteins (twofold, p-value ≤ 0.05). Bioinformatics analysis of significantly altered proteins showed enrichment of proteins involved in extracellular matrix interactions, necroptosis and peroxisome mediated fatty acid oxidation. Kinase-Substrate Enrichment Analysis showed significant increase in activity of kinases such as ROCK1, RAF1, PRKCE and HIPK2 in shammah treated cells. These results provide better understanding of how shammah transforms non-neoplastic cells and warrants additional studies that may assist in improved early diagnosis and treatment of shammah induced oral cancer.

## Introduction

Oral cancer is a subgroup of head and neck cancers and most common type of cancer worldwide with a dismal 5-year survival rate of less than 50%^1, 2^. Cancers of the lip and oral cavity are ranked at the 17th spot based on incidence worldwide^3^. The major risk factors associated with oral squamous cell carcinoma (OSCC) are tobacco use, alcohol consumption and human papilloma virus (HPV) infection^4^. The consumption of tobacco in both smoke or smokeless form makes an individual susceptible to oral cancer^5^. There are more than twenty forms of orally consumed or nasally inhaled smokeless tobacco (ST) reported in Sweden, USA and in Indian subcontinent^6, 7^. These include tobacco chewing, snuff dipping, betel quid chewing and shammah amongst others. Shammah is a smokeless or unburned form of tobacco which consists of powdered leaves, carbonate of lime among other substances and is commonly chewed in Yemen and south of Saudi Arabia (KSA). Alkaline burn is often observed at the site of shammah placement (buccal cavity or under the cheek or lip) in oral cavity leading to oral mucosal change which resembles leukoplakia^8^. A recent study on 346 males from Yemen reported that shammah consumers are at 13 times higher risk of developing leukoplakia-like lesions compared to non-users^9^. Another study with 210 participants from Jazan KSA revealed that shammah users have 33 times higher chance of developing oral cancer compared to non-users^10^. Similarly, a case–control study from Yemen demonstrated shammah to be the major risk factor of OSCC^11^. Increased usage of shammah in Jazan Province has been linked to higher occurrence of oral squamous cell carcinoma^12^.

In vitro genotoxicity test have shown the presence of direct-acting mutagen in chloroform extract of shammah^13^. Whole exome sequencing analysis of 15 archival OSCC samples with history of shammah exposure reported mutation in genes previously associated with OSCC along with novel mutational driver events including CSMD3, TRPM2 and NOTCH3. Copy number alteration (CNAs) analysis of these samples revealed amplification of genes categorized as oncogenic such as MDM2 and BAG1 and deletion of potential tumor suppressor gene SMARCC1^14^.

Various in-vitro cell-based studies have reported effects of acute exposure of different types of smokeless tobacco extract (STE)^15, 16^. Altered expression of key cell regulatory proteins such as cyclin D1, pRb and O6-methyl guanine-DNA methyl transferase (MGMT) has been observed in oral epithelial cells in response to smokeless tobacco ‘Khaini’^17^. Increased production of reactive oxygen species (ROS) and consequent cellular damage has been reported in oral epidermal carcinoma cells in response to acute exposure to STE^16^. Effect of shammah has been demonstrated in liver cells HepG2 reporting its ability to induce apoptosis and cell cycle arrest at G2 to M phase^18^. However, there is lack of literature about the effect of shammah on oral cells which is the direct site of exposure.

In this study, we generated an in vitro cellular model using normal, non-transformed oral keratinocytes (OKF6/TERT1)^19^, which were chronically treated with shammah for a period of 6 months. Upon establishment of the cellular model, we employed tandem mass tag (TMT) based quantitative proteomic and TiO_2_-based phosphoenrichment approach to study global proteomic and phosphoproteomic alterations in OKF6/TERT1 cells upon chronic exposure to shammah.

## Results

### Chronic shammah treatment induces phenotypic alterations in oral keratinocytes

**Figure 1:**
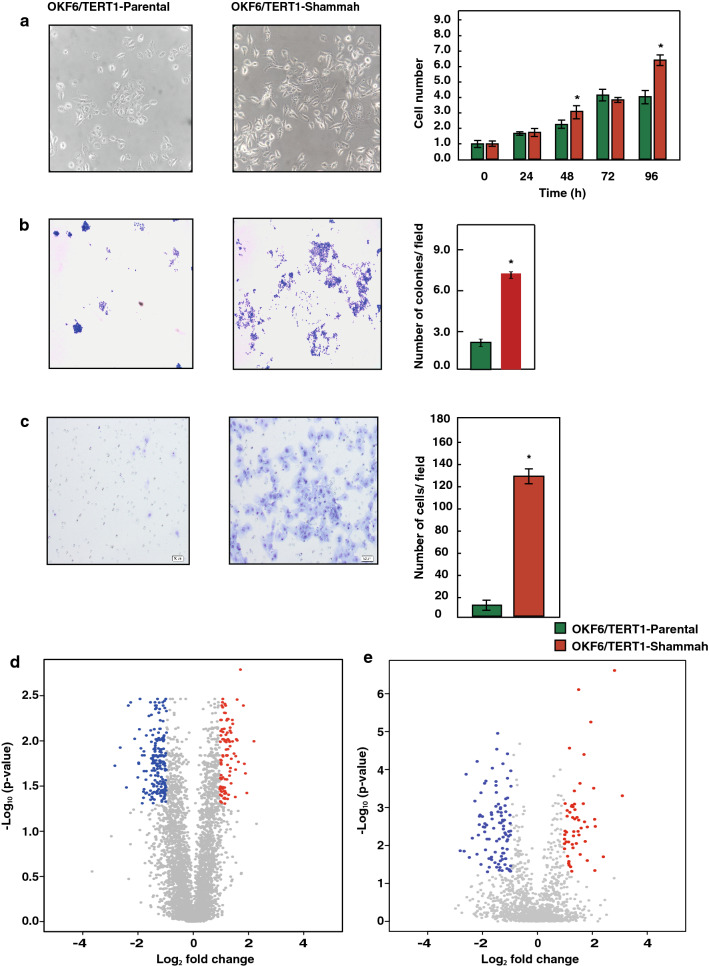
Chronic shammah treatment induces phenotypic alterations in oral keratinocytes. OKF6/TERT1-Parental and OKF6/TERT1-Shammah cells were assessed for (**a**) cellular proliferation (**b**) colony formation and (**c**) invasive capability with corresponding bar graphs shown in the right panel. Volcano plot representation of OKF6/TERT1 cells treated with shammah extract (**d**) total proteomics (**e**) phosphoproteomics. Significantly (p ≤ 0.05) overexpressed and downregulated proteins are depicted in red and blue, respectively.

Non-neoplastic oral keratinocytes, OKF6/TERT1, were treated at varying concentrations of shammah extract ranging from 0 to 5% to determine the optimum concentration for chronic treatment (data not shown). The highest concentration with which the cells could be treated chronically was 0.1%. Cells treated at higher concentrations of shammah (> 0.1%) underwent apoptosis/necrosis within days of treatment (data not shown). Six months of chronic exposure to shammah resulted in cellular morphological changes in OKF6/TERT1-Shammah as compared to OKF6/TERT1-Parental cells. A significant increase in the proliferative ability of the cells was observed after chronic treatment with shammah (Fig. 1a). We further observed significant increase in colony formation capability and the invasive ability of OKF6/TERT1-Shammah cells in comparison to OKF6/TERT1-Parental cells (Fig. 1b,c). These results indicated that shammah exposure induces growth signals that trigger cellular transformation in OKF6/TERT1 cells.

### Chronic treatment with shammah alters the cellular proteome and phosphoproteome

We employed TMT and TiO_2_ based proteomic and phosphoproteomic approach to identify proteomic and phosphoproteomic alterations in oral keratinocytes in response to shammah exposure (Supplementary Fig. S1A). Total proteome analysis led to the quantification of 4712 proteins, amongst which 129 proteins were overexpressed (≥ twofold, p-value ≤ 0.05) and 273 proteins were downregulated (≤ twofold, p-value ≤ 0.05) (Fig. 1d). TiO_2_ based phosphoenrichment of peptides led to quantification of 1555 phosphopeptides that contained phosphorylation sites mapping to 836 proteins. We identified 2767 unique phosphosite modifications comprising 2275 serine, 440 threonine and 52 tyrosine sites (Supplementary Fig. S1B). Of the 836 proteins which were identified, 36 proteins were hyperphosphorylated (≥ twofold, p-value ≤ 0.05) while 83 proteins were hypophosphorylated (≤ twofold, p-value ≤ 0.05) (Fig. 1e). The complete list of identified proteins and phosphopeptides is provided in Supplementary Table S1 and Supplementary Table S2, respectively.

### Chronic treatment with shammah induces signaling associated with extracellular matrix and peroxisomal biogenesis

**Figure 2:**
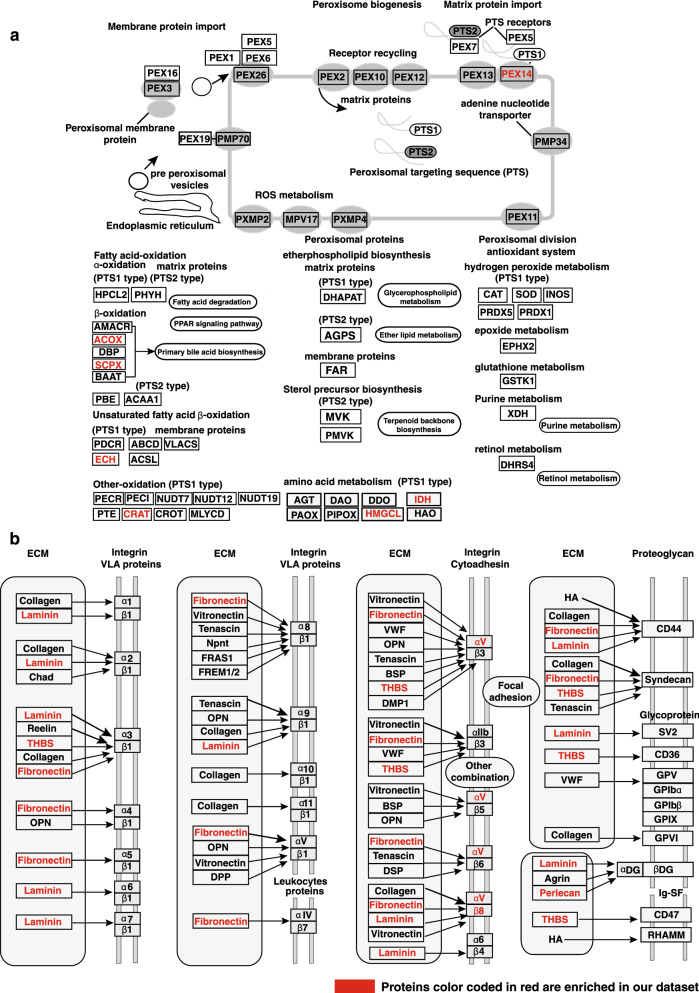
Chronic treatment with shammah induces signaling associated with extracellular matrix and peroxisomal biogenesis. Kyoto Encyclopedia of Genes and Genomes (KEGG)^80^ pathway analysis of overexpressed and/or hyperphosphorylated proteins (**a**) overexpressed and/or hyperphosphorylated proteins showed enrichment of peroxisome mediated long chain fatty acid oxidation and (**b**) enrichment of proteins in extracellular matrix organization (ECM). Proteins color coded in red are enriched in our dataset.

Bioinformatics analysis of overexpressed and/or hyperphosphorylated proteins revealed enrichment of proteins involved in extracellular matrix (ECM) interactions and peroxisome mediated fatty acid oxidation (Fig. 2a,b and Supplementary Fig. S2A). ECM is a dynamic non-cellular component of tissue organisation comprised of proteoglycans, collagen, glycoproteins, laminins and fibronectin, which provides structural and biochemical support to surrounding cellular architecture. Higher expression of ECM components such as expression of fibronectin (FN1) (3.60-fold; p-value ≤ 0.05), thrombospondin 1 (THBS1) (3.26-fold; p-value ≤ 0.05) and heparan sulfate proteoglycan 2 (HSPG2) (1.95-fold; p-value ≤ 0.05) were identified in OKF6/TERT1-Shammah cells compared to OKF6/TERT1-Parental cells. Moreover, laminins which form an integral part of ECM were also found to be significantly elevated in shammah treated cells. Laminin subunit alpha-3 (LAMA3) (2.52-fold; p-value ≤ 0.05), laminin subunit beta-3 (LAMB3) (2.69-fold; p-value ≤ 0.05) and laminin subunit gamma-2 (LAMC2) (3.51-fold; p-value ≤ 0.05) were seen significantly overexpressed in OKF6/TERT1-Shammah cells. Integrins are transmembrane receptor proteins responsible for attachment of cells to ECM and transmitting growth signals from ECM to cells for cellular proliferation and differentiation. Abundance levels of integrin proteins ITGB8 (2.17-fold; p-value ≤ 0.05) and ITGAV (1.97-fold; p-value ≤ 0.05) were found significantly high in OKF6/TERT1-Shammah cells.

Peroxisome are highly dynamic unit membrane cell organelle which performs numerous functions notably β-oxidation of long chain fatty acids, reduction of reactive oxygen species (ROS) and biosynthesis of plasmalogens (ether phospholipids). Our data shows an increased expression of proteins involved in peroxisome mediated catabolism of long chain fatty acids such as peroxisomal biogenesis factor 14 (PEX14) (2.2-fold; p-value ≤ 0.05), acyl-CoA oxidase 1 (ACOX1) (2.42-fold; p-value ≤ 0.05), sterol carrier protein 2 (SCP2) (2.59-fold; p-value ≤ 0.05), enoyl-CoA hydratase 1 (ECH1) (1.98-fold; p-value ≤ 0.05), carnitine O-acetyltransferase (CRAT) (2.02-fold; p-value ≤ 0.05), isocitrate dehydrogenase (IDH2) (1.96-fold; p-value ≤ 0.05), isocitrate dehydrogenase [NAD] subunit gamma (IDH3G) (2.00-fold; p-value ≤ 0.05) and hydroxymethylglutaryl-CoA lyas (HMGCL) (1.95-fold; p-value ≤ 0.05) in OKF6/TERT1-Shammah compared to OKF6/TERT1-Parental cells.

### Chronic shammah treatment alters necroptosis pathway

**Figure 3:**
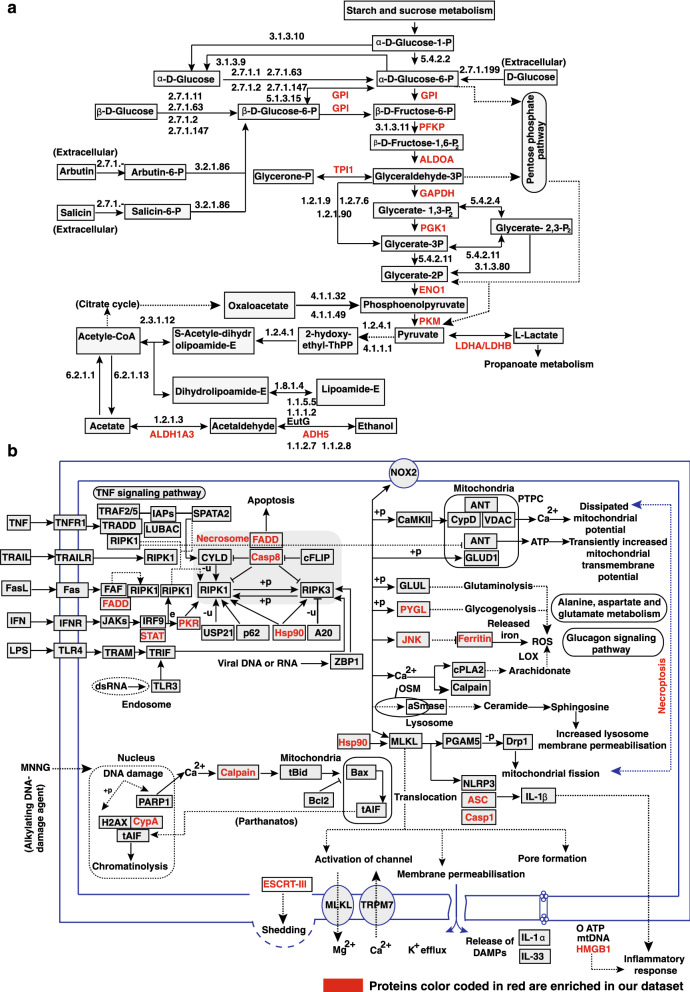
Chronic shammah treatment alters necroptosis pathway. Kyoto Encyclopedia of Genes and Genomes (KEGG)^80^ pathway analysis of downregulated and/or hypophosphorylated proteins (**a**) downregulated and/or hypophosphorylated proteins showed enrichment of metabolic pathway glycolysis and (**b**) necroptotic pathway. Proteins color coded in red are enriched in our dataset.

Downregulated and/or hypophosphorylated proteins showed enrichment of proteins mapped to metabolic pathway glycolysis/gluconeogenesis in addition to programmed form of necrosis called necroptosis (Fig. 3a,b and Supplementary Fig. S2B). Proteins/enzymes which drive the glycolytic pathway such as glucose-6-phosphate isomerase (GPI) (0.39-fold; p-value ≤ 0.05), phosphofructokinase (PFKP) (0.46--fold; p-value ≤ 0.05), aldolase A (ALDOA) (0.43-fold; p-value ≤ 0.05), glyceraldehyde-3-phosphate dehydrogenase (GAPDH) (0.44-fold; p-value ≤ 0.05), phosphoglycerate kinase 1 (PGK1) (0.51-fold; p-value ≤ 0.05), enolase 1 (ENO1) (0.38-fold; p-value ≤ 0.05) and pyruvate kinase (PKM) (0.25-fold; p-value ≤ 0.05) were observed to be significantly downregulated in OKF6/TERT1-Shammah cells. Necroptosis is a programmed form of necrotic cell death which mechanistically resembles apoptosis and morphologically has similarities with necrosis. Evidences have shown necroptosis has both tumor promoting and tumor suppressing effects in different cancer types. Decreased expression of various necrotic factors has been identified in breast cancer, colorectal cancer, gastric cancer and head and neck squamous cell carcinoma (HNSCC)^20–23^. We found reduced expression of several genes mapped to necroptic pathway such as Fas associated via death domain (FADD) (0.49-fold; p-value ≤ 0.05), peptidylprolyl isomerase A (PPIA) (0.43-fold; p-value ≤ 0.05), caspase 8 (CASP8) (0.48-fold; p-value ≤ 0.05), caspase 1 (CASP1) (0.37-fold; p-value ≤ 0.05), glycogen phosphorylase (PYGL) (0.39-fold; p-value ≤ 0.05), calpain 2 (CAPN2) (0.51-fold; p-value ≤ 0.05), PYD and CARD domain containing (PYCARD) (0.43-fold; p-value ≤ 0.05), eukaryotic translation initiation factor 2 alpha kinase 2 (EIF2AK2) (0.44-fold; p-value ≤ 0.05) and high mobility group box 1 (HMGB1) (0.41-fold; p-value ≤ 0.05) in OKF6/TERT1-Shammah cells.

### Alteration of protein kinase mediated signaling

**Figure 4:**
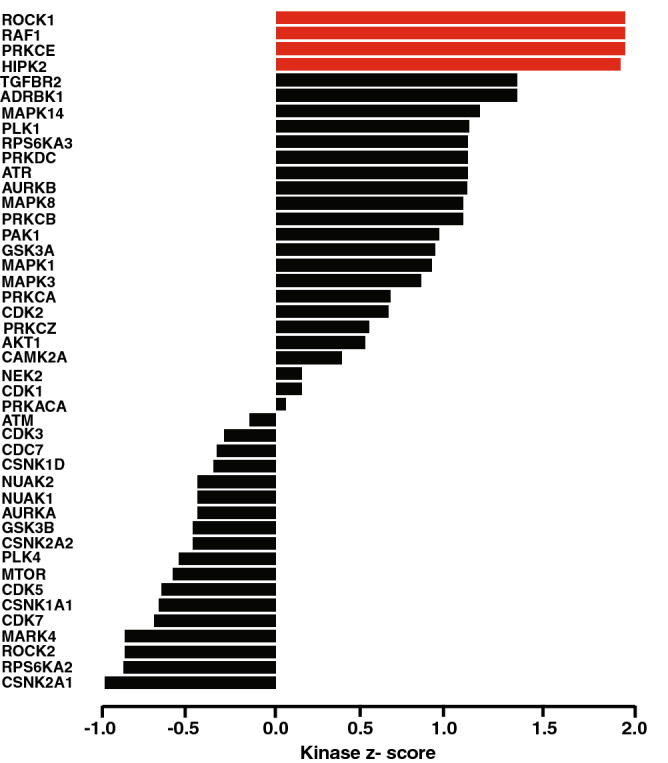
Alteration of protein kinase mediated signaling. Kinase–Substrate Enrichment Analysis (KSEA) tool scores each kinase based on the relative hyper or hypophosphorylation of its substrates. The positive or negative score implies increase or decrease in kinase’s overall activity as compared to the control. The kinases which are color coded in red are significantly activated in shammah treated oral keratinocytes.

Protein kinases play key regulatory role in signaling cascade which affect cell cycle regulation, growth and differentiation. Aberrant regulation of kinase activity leads to dysregulation of key cell signaling pathways which are responsible for multiple disease conditions including cancer. We used Kinase-Substrate Enrichment Analysis (KSEA) tool for the characterisation of kinase activity from the phosphoproteomics data set. Rho associated coiled-coil containing protein kinase 1 (ROCK1), Raf-1 proto-oncogene, serine/threonine kinase (RAF1), protein kinase C epsilon (PRKCE) and homeodomain interacting protein kinase 2 (HIPK2) were predicted to be significantly enriched (Fig. 4). ROCK1 (p = 0.017; z-score = 2.1), RAF1 (p = 0.017; z-score = 2.1) and PRKCE (p = 0.017; z-score = 2.1) were predicted to be activated and responsible for the phosphorylation of vimentin (VIM) at site S299. HIPK2 (p = 0.018; z-score = 2.07) was predicted to be activated upstream kinase of substrates HNRNPH1 (S104), H2AFX (S140), LMNA (S392), NCL (S563) and PKD2 (S812).

## Discussion

Epidemiological studies have shown direct association of shammah exposure with development of oral lesions such as leukoplakia, erythroplakia and OSCC^9, 24^. Genomic studies have identified driver mutations associated with oral cancer in shammah users. Though the first contact with shammah remains oral cavity, the precise molecular alterations in response to shammah exposure is lacking. In addition, the alterations induced by any tobacco product can be best appreciated by chronic exposure and not acute. We and others have shown the effect of chronic exposure to different forms of tobacco on multiple cell types using in vitro models^25–32^. In this study, we have developed an in vitro cellular model to study the effect of chronic shammah exposure on oral keratinocytes. Non-neoplastic oral keratinocytes were chronically treated with shammah for a period of 6 months followed by evaluation of proteomic and phosphoproteomic alterations in response to chronic exposure to shammah. Our data indicates phenotypic alterations associated with oncogenic transformation in normal oral keratinocytes chronically treated with shammah. Shammah treated cells showed a higher rate of proliferation, colony formation and invasive ability compared to the parental cells, which is one of the hallmarks of malignant transformation. Our data indicates significant dysregulation of proteins and phosphorylated proteins in cells chronically treated with shammah.

KEGG pathway analysis of upregulated and/or hyperphosphorylated proteins showed enrichment of proteins in ECM and peroxisome mediated fatty acid oxidation. Frequently disorganised and dysregulation of ECM proteins have been reported in multiple cancer types which stimulates tumor progression and metastasis. Tumor microenvironment plays a pivotal role in the formation and dissemination of cancer cells to distant locations. Alterations in cell adhesion and ECM disorganisation/remodelling are prerequisites for cancer invasion and metastasis. Overexpression of ECM factors have been associated with chemoresistance and is a marker for poor prognosis^33^. Thrombospondin 1 (THBS1) (3.26-fold; p-value ≤ 0.05) which was significantly overexpressed in OKF6/TERT1-Shammah cells has been documented to be elevated in OSCC specimens induced by TGFBI which further promotes tumor migration and invasion^34^. High expression levels of LAMC2 have been reported in various cancer types such as lung carcinoma, colorectal carcinoma, and pancreatic cancer^35–38^. Higher expression of LAMC2 was also shown to be associated with poor prognosis in ESCC patients^39^. Elevated levels of LAMC2 were observed in oral tongue squamous cell carcinoma and HNSCC tissues in comparison to normal tissues^40^. We observed an overexpression of LAMC2 (3.51-fold; p-value ≤ 0.05) in OKF6/TERT1-Shammah compared to OKF6/TERT1-Parental cells. Fibronectin (FN1) is an extracellular matrix glycoprotein which binds collagen, fibrin and integrin receptors and plays a major role in cell migration and adhesion. Higher expression of FN1 has been reported in various cancers including HNSCC, renal carcinoma and lung cancer^41–44^. Our study shows significant overexpression of FN1 (3.60-fold; p-value ≤ 0.05) in OKF6/TERT1-Shammah cells.

Peroxisomes play an essential role in diverse metabolic activities and have been reported to be deregulated in various disease conditions including cancer. This includes prostate, hepatocellular, colorectal, breast and bladder carcinoma^45–48^. Peroxisome mediated metabolism of ether phospholipids serves as an alternative source of energy to meet the energy requirements of highly proliferating cancer cells and has been well documented in multiple cancers^49^. Potential role of peroxisomes in tumorigenesis is supported by its crosstalk with mitochondria in beta-oxidation of fatty acids^50^. Peroxisome containing proteins/enzymes have either oncogenic or tumor suppressor effect on tumor growth which in turn depends on the tumor cell type and tumor microenvironment. Higher expression of PEX14 has been reported in III and IV grade glioma both at transcriptomic and proteomic levels in comparison to normal healthy brain specimens^51^. We observed increased expression of PEX14 (2.2-fold; p-value ≤ 0.05) in shammah treated oral keratinocytes. Higher expression of ACOX1 is reported in both human epidermal growth factor receptor 2 (HER2) and estrogen receptor (ER) positive breast cancers and has been associated with poor survival^52^. ACOX1 has been shown to promote tumorigenesis in hepatocellular carcinoma by the succinylation of ACOX1 with concomitant downregulation of sirtuin 5 (SIRT5)^53^. In concordance with these studies ACOX1, which is integral part of peroxisomal pathway, was found to be overexpressed in OKF6/TERT1-Shammah cells.

Necroptosis is initiated by tumor necrosis factor (TNF), TNF-related apoptosis-inducing ligand (TRAIL), Fas ligand (FasL) and many other agents which further requires activity of receptor-interacting serine/threonine-protein kinase 1 (RIPK1) and receptor-interacting serine/threonine-protein kinase 3 (RIPK3). Fas-associated protein with death domain (FADD) and caspase-8 proteins forms complex with RIPK1 and RIPK3 to mediate necroptosis. The role of necroptosis in cancer biology is quite complicated; expression of some of the key signaling proteins associated with necroptosis are reported to be downregulated in majority of cancers^54, 55^. Reduced expression of necroptotic factors favours cancer progression and metastasis. Promoter hypermethylation of FADD gene and its reduced expression has been reported in OSCC patients compared to healthy cases^56^. However, there are limited studies which describe the tumor promoting or oncogenic role of necroptotic pathway. A study by Li et al.; has shown necroptosis promotes tumor migration and invasion by releasing damage-associated molecular patterns (DAMPs) in head and neck squamous cell carcinoma^57^. We observed reduced expression of necroptotic proteins in shammah treated oral keratinocytes which could possibly be responsible for transformation of normal non-transformed oral cells.

Recent advances in cancer research have established the crucial role of kinases in modulating carcinogenesis/metastasis and are considered to be key therapeutic targets in multiple tumor malignancies^58^. KSEA based analysis of our data set revealed increased activity of four kinases ROCK1, RAF1, PRKCE and HIPK2. Vimentin is known to be phosphorylated by ROCK1, RAF1, and PRKCE at S299. ROCK1 is frequently upregulated in various malignancies including OSCC. A study by Jin et al.*;* has shown overexpression of ROCK1 in OSCC tumor tissues in comparison to healthy controls^59^. PRKCE is known to be implicated in EMT by which it mediates the motility of cells and tumor invasion. It also affects the ECM interactions by regulating the assembly of cytoskeletal elements within the cell. Nevertheless, PRKCE activation has a protective role in cardiac and brain ischemia while its aberrant activation induces cell proliferation, disruption of cell–cell contacts, tumor progression and metastasis. Higher expression of PRKCE is reported in multiple cancer types such as HNSCC, breast cancer and lung cancer^60^. RAF1 is a part of the signaling module RAS/RAF/ERK pathway and its dysregulation is associated with colorectal and thyroid carcinoma^61–63^. Moreover, invasive property of lung cancer cells A549 was significantly increased on RAF1 transfection^64^. HIPK2 regulates the expression of various genes by interacting with homeobox genes and is found to be significantly overexpressed in cervical and prostate carcinoma^65, 66^.

Interestingly, we observed many differentially phosphorylated proteins that were unaltered at the protein expression level in OKF6/TERT1-Shammah cells. Proteins such as myeloid leukemia factor 2 (MLF2), ladinin-1 (LAD1), apoptotic chromatin condensation inducer in the nucleus isoform 1 (ACIN1), keratin, type II cytoskeletal 8 isoform 1 (KRT8), RNA-binding protein 10 isoform X1 (RBM10), lamin-B2 (LMNB2), and collagen alpha-1(XVII) chain (COL17A1) amongst others were found to be significantly hyperphosphorylated (p-value ≤ 0.05) while they showed no change in protein expression in response to shammah exposure. Phosphorylation of MLF2 at serine 24 plays an essential role in the proliferation and oncogenicity in chronic myelogenous leukemia (CML)^67^. LAD1 is a downstream phosphoserine and phosphothreonine effector protein of EGFR/ERK signaling pathway and its phosphorylation has been seen to control cell proliferation and migration in untransformed mammary epithelial cells on stimulation with EGF^68^. ACIN1 is serine/arginine-rich domain (SR proteins) protein which aids in mRNA splicing and processing. It was shown to regulate the expression of Cyclin A1 via SRPK2 mediated phosphorylation in human leukemia cells and patients with myeloid hematological malignancies^69^. Phosphoproteomic study carried out by Ruan et al.*;* has shown KRT8 as downstream target of EGFR mediated signaling network in nasopharyngeal carcinoma^70^. Similarly, proteins heat shock protein HSP 90-beta isoform X1 (HSP90AB1), ubiquitin carboxyl-terminal hydrolase 24 isoform X1 (USP24), myosin-9 isoform X1 (MYH9), integrin beta-4 isoform X1 (ITGB4), serine/arginine-rich splicing factor 2 (SRSF2), high mobility group protein HMG-I/HMG-Y isoform a (HMGA1), cyclin-dependent kinase 2 isoform X1 (CDK2), proline-rich AKT1 substrate 1 isoform a (AKT1S1), and eukaryotic translation initiation factor 4B isoform 1 (EIF4B) were found to be significantly hypophosphorylated (p-value ≤ 0.05) although their protein expression remained unaltered in OKF6/TERT1-Shammah cells. Phosphorylation of HSP90AB1 at Ser^255^ was found to be associated with lung adenocarcinoma. Site specific inhibition of HSP90AB1 phosphorylation (Ser 255) leads to suppression of key signaling pathways involved in cancer progression and metastasis, including the MAPK/ERK cascade^71^. Protein tyrosine phosphatase 1B (PTP1B) positively regulates the EGFR expression by dephosphorylating MYH9 at Y1408 to promote cell migration and invasion in ESCC^72^.

Our study presents the first comprehensive analysis of proteome and phosphoproteome of oral keratinocytes exposed to shammah tobacco preparation. Our findings indicate the chronic exposure of oral keratinocytes to shammah results in the altered expression/phosphorylation of multiple proteins/kinases which play a critical role in ECM modulation, lipid metabolism, necroptosis and cancer signaling. These findings will serve as a rich resource to understand the molecular underpinnings of shammah induced OSCC. Our results are corroborating with previously published studies and we hypothesise that altered expression/phosphorylation of these proteins play an essential role in shammah induced transformation of oral keratinocytes leading to malignancy. However, given the tumor complexity and heterogeneity, these findings warrant further experimentation and clinical validation in larger cohorts of OSCC patients habitual of shammah smoking.

## Materials and methods

### Preparation of shammah extract

Shammah was procured from Jazan, Saudi Arabia. 50 g of shammah homogenized in 100 ml of 1× phosphate buffer saline (PBS). The mixture was stirred at 37 °C for 24 h, followed by centrifugation at 2000*g* for 15 min. The supernatant was collected, filtered using a Whatman filter paper, and sterilized using 0.22 µm filter. This was considered as 100% extract of shammah. The shammah extract was aliquoted and stored at 80 °C until further use.

### Treatment of OKF6/TERT1 cells with shammah

Human oral keratinocytes OKF6/TERT1 cells were a generous gift from Dr. James Rheinwald (Brigham and Women’s Hospital, Boston, MA). OKF6/TERT1 cells are normal non-transformed oral mucosal epithelial cells immortalized by hTERT^19^. OKF6/TERT1 were cultured and maintained in keratinocytes serum free medium (KSFM) supplemented with 1% penicillin/streptomycin, CaCl_2_ (0.4 mM), bovine pituitary extract (25 µg/ml) and epidermal growth factor (EGF) (0.2 ng/ml). Cells were grown at 37 °C in a humidified 5% CO_2_ incubator. OKF6/TERT1 cells were treated with 0.1% of shammah extract for a period of 6 months to study chronic shammah exposure. Untreated parental OKF6/TER1 cells which were not treated with shammah were maintained for the same duration. Henceforth, OKF6/TERT1 cells treated with shammah are referred as “OKF6/TERT1-Shammah” and parental control cells are referred as “OKF6/TERT1-Parental” cells.

### Cell proliferation assays

OKF6/TERT1-Parental and OKF6/TERT1-Shammah cells were seeded in 96-well plate at a density of 5 × 10^3^ cells/ well. Cellular proliferation was monitored for 4 days using MTT (3-(4,5-dimethylthiazol-2yl)-2,5-diphenyl tetrazolium bromide) assays as previously described^73^. Absorbance was measured at 570 nm and 650 nm. All experiments were carried out in triplicate and repeated thrice. Paired t-test was carried out to evaluate the difference between the control and treated groups. P-value < 0.05 was considered to be significant.

### Colony formation assays

Colony formation assays were carried out as described previously^25^. Briefly, OKF6/TERT1-Parental and OKF6/TERT1-Shammah cells were seeded in triplicate at a density of 3 × 10^3^/well in 6-well plates and cells with complete media. Cell colonies were allowed to grow for 10–14 days, before the colonies were fixed with methanol and stained with 4% methylene blue solution.

Colonies formed was counted for ten randomly selected viewing fields and representative images were photographed at 2.5× magnification. All experiments were performed in triplicate and repeated thrice.

### Cell invasion assays

Invasion assays were performed in a transwell system (BD Biosciences) with Matrigel-coated filters, and cellular invasion was evaluated after 48 h as described previously^25^. Invasiveness of the cells was assayed in the membrane invasion culture system using polyethylene terephthalate (PET) membrane (8-μm pore size) in the upper compartment of a transwell coated with Matrigel (BD BioCoat Matrigel Invasion Chamber; BD Biosciences). OKF6/TERT1-Parental and OKF6/TERT1-Shammah were seeded at a density of 2 × 10^4^ cells/well in serum free media in the upper compartment of the transwell. The lower compartment was filled with complete growth media and the plates were maintained at 37 °C for 48 h. At the end of the incubation time, the upper surface of the insert membrane was wiped with a cotton-tip applicator to remove non-migratory cells. Cells that have invaded the matrigel to the lower surface of the membrane were fixed and stained with 4% methylene blue (Sigma, St. Loius, MO) in 50% methanol. All experiments were carried out in triplicates and repeated thrice. The number of cells that invaded were counted for 10 randomly selected fields and imaged at 10× magnification. Paired t-test was carried out to evaluate the difference between the control and treated groups. P-value < 0.05 was considered to be significant.

### Protein extraction

OKF6/TERT1-Parental and OKF6/TERT1-Shammah were cultured and grown till 80% confluence. The cells were serum starved for 8 h in ice-cold 1X PBS, lysed in 2% of SDS buffer (2% SDS, 5 mM sodium fluoride, 1 mM β-glycerophosphate, 1 mM sodium orthovanadate in 50 mM Triethyl ammonium bicarbonate (TEABC) followed by sonication (Branson Sonifier, Danbury, CT) and centrifugation. The cell lysates were sonicated at 40% amplitude and centrifuged at 12,000 rpm for 10 min. Protein was precipitated overnight at − 80 °C using the ice cold acetone. The samples were centrifuged at 12,000 rpm for 15 min; the acetone was removed and air-dried. The pellet was then dissolved in 4 M urea. Protein estimation was carried out using bicinchoninic acid assays (BCA) method (Thermo Scientific, Bremen, Germany)^74^. Equal amounts of protein from each cell line were reduced using dithiothreitol (DTT) at 60 °C for 20 min and alkylated with iodoacetamide (IAA) for 10 min at room temperature. Proteins were then digested using Lysyl Endopeptidase, Mass Spectrometry Grade (Catalog#125-05061, Wako, Richmond, VA) at 1:100 enzyme to protein ratio for 4 h at 37 °C. After 4 h, the urea concentration was reduced from 4 to 2 M using 50 mM TEABC. The samples were then digested using TPCK-treated trypsin (Worthington, NJ) at a 1:20 enzyme to protein ratio for 16 h at 37 °C. The samples were cleaned using Sep-PAK Classic C_18_ columns (Catalog#WAT051910, Waters, Milford, MA).The samples were then completely dried and reconstituted in 50 mM TEABC buffer.

### TMT labeling and basic reversed-phase liquid chromatography (bRPLC)

After tryptic digestion of proteins, the peptides were labelled with tandem mass tag (TMT) reagents as described previously^25^. Briefly, peptide samples were dissolved in 50 mM TEABC (pH 8.0) and added to TMT reagents dissolved in anhydrous acetonitrile. Peptides from OKF6/TERT1-Parental and OKF6/TERT1-Shammah cells were labelled with 129 and 130 TMT tags, respectively. After 60 min incubation at room temperature, the reaction was quenched with 5% hydroxylamine. The labelled samples were pooled and subjected to fractionation. The 96 fractions obtained were concatenated into 6 fractions.

### Phosphopeptide enrichment using titanium dioxide

To carry out phosphoproteomic, phosphopeptide enrichment was carried out using TiO_2_-based enrichment method, as described previously^75^. Briefly, TiO_2_ beads were washed with 5% 2,5-dihydroxybenzoic acid (DHB) at room temperature for 2 h using a rotor. Peptide fractions were dissolved in 5% DHB containing TiO_2_ beads and incubated for 30 min while placed on the rotor. Following this, TiO_2_ beads were washed repeatedly and eluted with 2% ammonia. The enriched peptides were vacuum-dried and desalted using C_18_ Stage Tips. The enriched and desalted peptides were further taken for LC/MS/MS analysis.

### LC–MS/MS analysis

Enriched phosphopeptides and total proteome were analyzed in triplicate on Orbitrap Fusion Tribrid mass spectrometer (Thermo Scientific) that was interfaced with nanoACQUITY UPLC liquid chromatography system (Waters, Milford, MA). Peptides reconstituted in 0.1% formic acid were loaded onto a trap column (180 µm × 20 mm, Acquity UPLC M-Class V/M symmetry) at a flow rate of 5 µL/min. The peptides were then resolved on an analytical column (75 µm × 200 mm Acquity UPLC Peptide BEH C18 nano column) at a flow rate of 300 nl/min using 10–35% solvent B (0.1% formic acid in 95% acetonitrile) for 97 min and 35–70% solvent B for 97 to 99 min. The MS and MS/MS scans were acquired at a mass resolution of 60,000 and 50,000 at 200 m/z. Full MS scans were acquired in the m/z range of 350–1400. Precursor ions with single charge or unassigned charge were rejected. Dynamic exclusion of fragmented precursor ions was set to 60 s.

### Data analysis

Proteome Discoverer software suite (https://www.thermofisher.com/store/products/OPTON-30945#/OPTON-30945, version 2.2.0.388, Thermo Fisher Scientific) was used for MS/MS searches using SEQUEST (version 2.4.1) algorithms against NCBI RefSeq human protein database (version 89). The search parameters included trypsin as the protease with a maximum of 2 missed cleavages; oxidation of methionine was set as a dynamic modification, carbamidomethylation of cysteine and TMT modification at peptide N-terminus and lysine were set as static modifications. For phosphoproteomic data, phosphorylation of serine, threonine, and tyrosine were defined as additional dynamic modifications. Precursor ion mass tolerance and fragment ion mass tolerance were allowed with 20 ppm and 0.05 Da, respectively and all the PSMs were identified with 1% FDR^76^. A false discovery rate of 1% was applied to peptide and protein levels. Proteins identified by at least one unique peptide were taken for further analysis. The ratio was calculated as follows: 130 (OKF6/TERT1-Shammah)/129 (OKF6/TERT1-Parental).

### Bioinformatics analysis

Student's t-test of all quantified proteins and phosphopeptides in OKF6/TERT1-Parental and OKF6/TERT1-Shammah cells was calculated using Perseus proteomics software (Version 1.6.2.2). Pathway analysis of differentially expressed/or phosphorylated proteins were carried out using clusterProfiler package in R (version 4.0.0) with Kyoto Encyclopedia of Genes and Genomes (KEGG) database resource^77, 78^. To identify and visualise kinase level annotations from the phosphoproteomic dataset we have used Kinase–Substrate Enrichment Analysis (KSEA) App. KSEA tool scores each kinase based on the relative hyper or hypophosphorylation of its substrates, as identified from phosphosite-specific Kinase–Substrate (K–S) databases. The positive or negative score implies increase or decrease in kinase’s overall activity as compared to the control^79^.

## Supplementary Information

Supplementary Information 1.

Supplementary Information 2.
