# Supplementary material for: Proteomic and phosphoproteomic profiling of shammah induced signaling in oral keratinocytes
Source: Sci Rep. 2021 Apr 30;11:9397. doi: 10.1038/s41598-021-88345-x (PMC8087671; doi:10.1038/s41598-021-88345-x)
Supplement: Supplementary file 1 — Supplementary Information 1. [file 41598_2021_88345_MOESM1_ESM.docx]

**Supporting information**

**Supplementary Figure S1. (A)** Workflow for the TMT/TiO_2_-based quantitative proteomic and phosphoproteomic analysis of OKF6/TERT1 cells chronically treated with shammah extract **(B)** Pie chart representation of phosphosites identified in shammah treated oral keratinocytes.

**Supplementary Figure S2. (A)** Pathway analysis of upregulated and/or hyperphosphorylated proteins **(B)** Pathway analysis of downregulated and/or hypophosphorylated proteins

**Supplementary Table S1:** List of proteins identified and quantified in OKF6/TERT1-Shammah and OKF6/TERT1-Parental total proteome using TMT-based quantitative proteomic approach.

**Supplementary Table S2:** List of phosphopeptides identified and quantified in OKF6/TERT1-Shammah and OKF6/TERT1-Parental phosphoproteome using TMT/TiO_2_-based quantitative phosphoproteomic approach.
