# Supplementary material for: Proteomic and phosphoproteomic profiling of shammah induced signaling in oral keratinocytes
Source: Sci Rep. 2021 Apr 30;11:9397. doi: 10.1038/s41598-021-88345-x (PMC8087671; doi:10.1038/s41598-021-88345-x)
Supplement: Supplementary file 2 — Supplementary Information 2. [file 41598_2021_88345_MOESM2_ESM.pdf]

Proteomic and phosphoproteomic profiling of shammah-induced signaling in oral keratinocytes

Shankargouda Patil[1#], Mohd Younis Bhat[2,3#], Jayshree Advani[2¥], Sonali V Mohan[2¥], Niraj Babu[2,4] , Keshava K. Datta[2], Tejaswini Subbannayya[2], Pavithra Rajagopalan[2], Firdous A. Bhat[2,3], Nezar Al-hebshi[5] , David Sidransky[6], Harsha Gowda[2,4*], Aditi Chatterjee[2,4*]

[1]Department of Maxillofacial Surgery and Diagnostic Sciences, Division of Oral Pathology, College of Dentistry, Jazan University, Jazan, Saudi Arabia
[2]Institute of Bioinformatics, International Technology Park, Bangalore, India
[3]School of Biotechnology, Amrita Vishwa Vidyapeetham, Kollam, India
[4]Manipal Academy of Higher Education, Manipal, India
[5]Department of Oral Health Sciences, Maurice H. Kornberg School of Dentistry, Temple University, Philadelphia, USA
[6]Department of Otolaryngology-Head and Neck Surgery, Johns Hopkins University School of Medicine, Baltimore, MD, USA

[#] These authors contributed equally to the manuscript.

[¥] These authors contributed equally to the manuscript.

***Corresponding Authors:**
Aditi Chatterjee
Institute of Bioinformatics
International Technology Park
Bangalore 560066, India
aditi@ibioinformatics.org

And

Harsha Gowda
Institute of Bioinformatics
International Technology Park
Bangalore 560066, India
harsha@ibioinformatics.org

Supplementary Figure S1

a

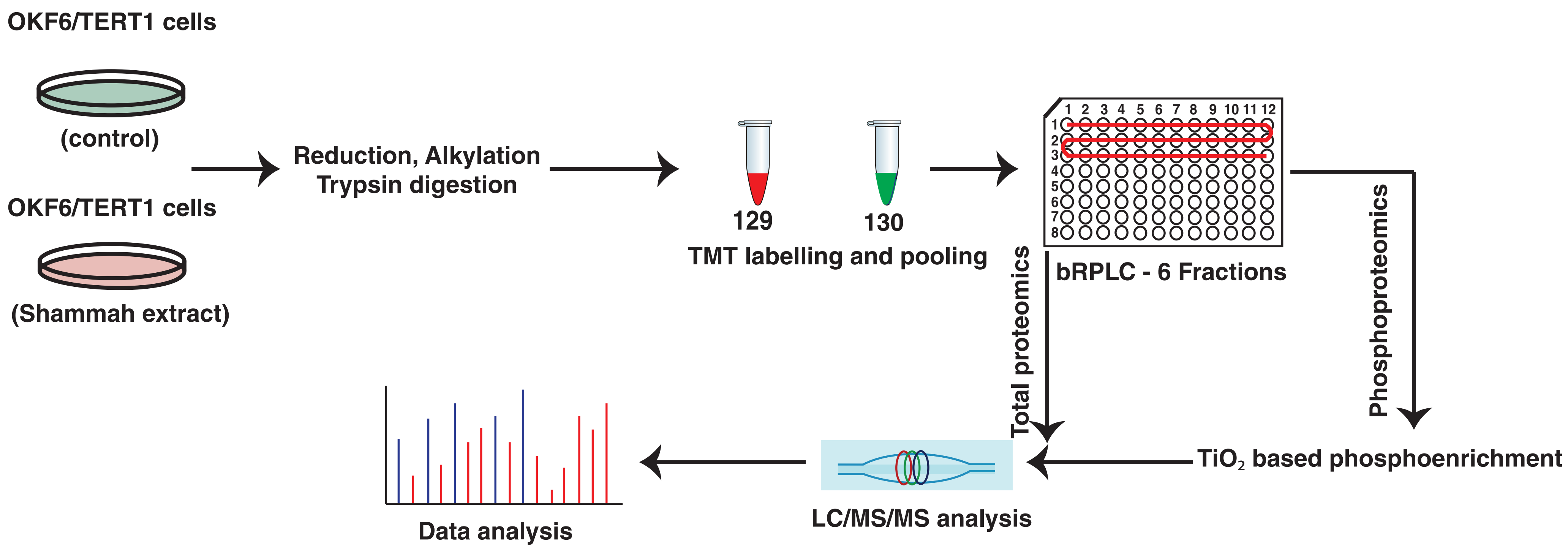

b

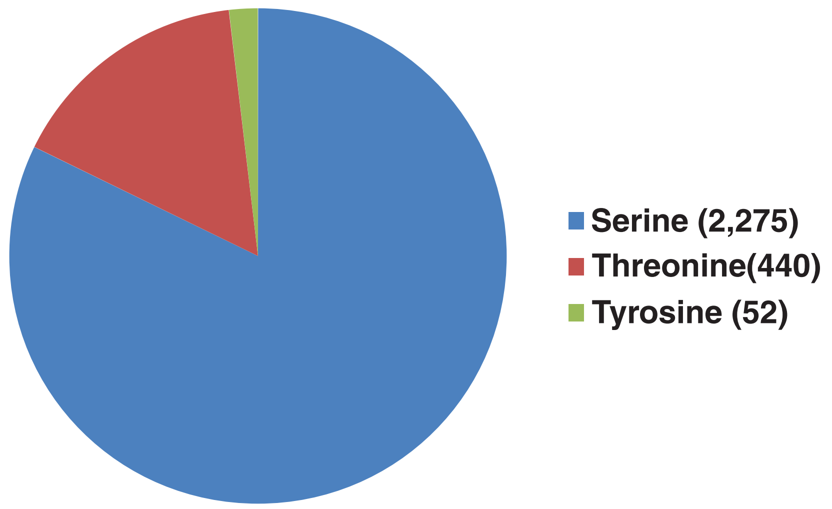

Supplementary Figure S2

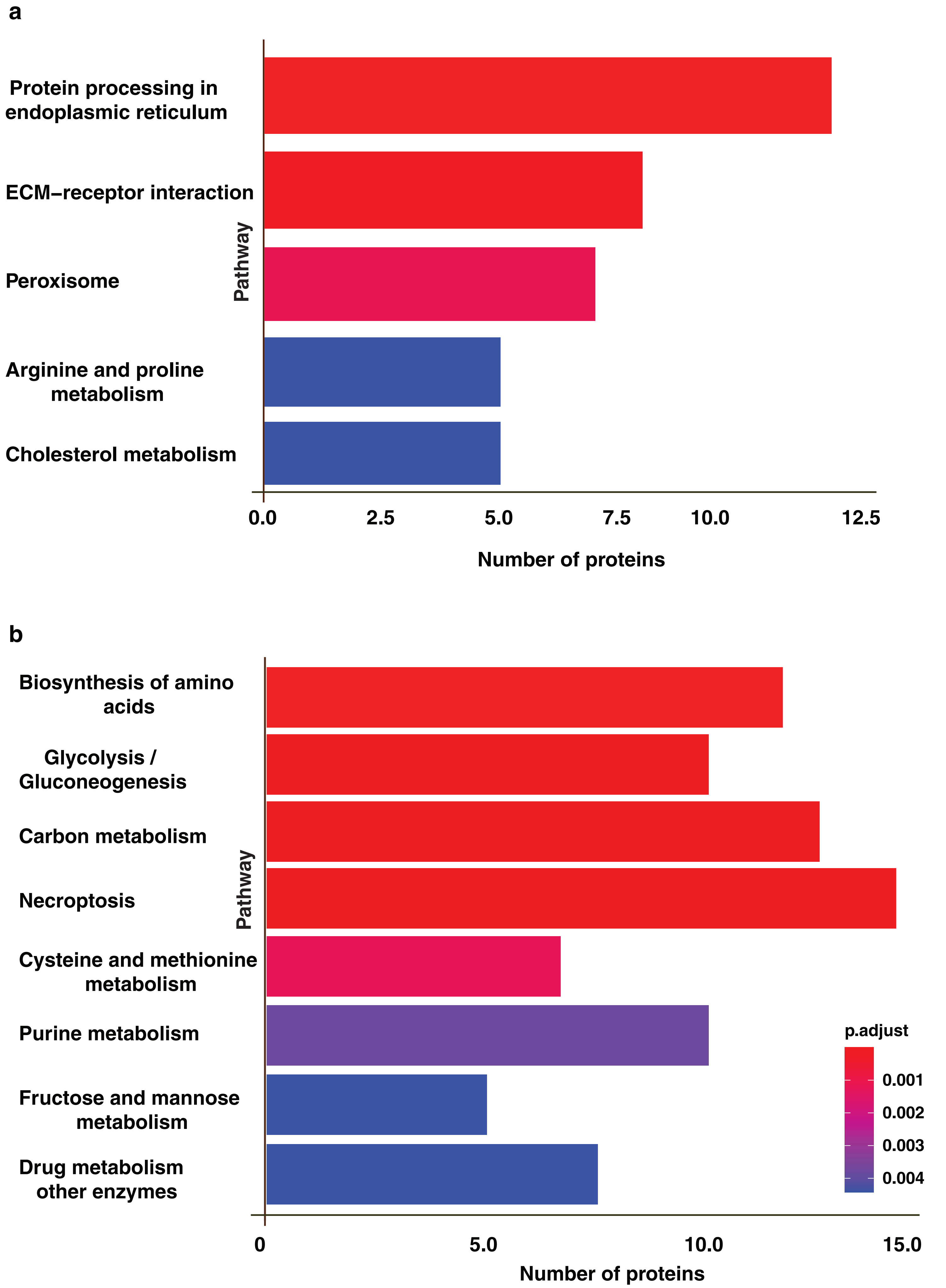

Patil et al,2020. Proteomic and phosphoproteomic profiling of shammah-induced signaling in oral keratinocytes

Supplementary Table S1: List of proteins identified and quantified in OKF6/TERT1-Shammah and OKF6/TERT1-Parental total proteome using TMT-based quantitative proteomic approach

Accession	Protein Description	Gene Symbol	Gene ID	Fold Change	Coverage [%]	# Peptides	# PSMs	# Unique Peptides	# AAs	MW [kDa]	p-value
XP_005251037.1	plectin isoform X5	PLEC	5339	1.241	58	264	1454	2	4552	516.6	0.776947352
XP_005251033.1	plectin isoform X3	PLEC	5339	0.654	56	264	1448	1	4689	532.1	0.459644787
XP_011515432.1	plectin isoform X2	PLEC	5339	0.718	58	261	1443	1	4552	516.4	0.035006968
NP_001333374.1	neuroblast differentiation-associated protein AHNAK isoform 1	AHNAK	79026	1.212	71	257	1134	256	5890	628.7	0.000714303
XP_016884292.1	myosin-9 isoform X1	MYH9	4627	0.555	60	139	1076	120	1960	226.4	3.26E-05
NP_001448.2	filamin-B isoform 2	FLNB	2317	0.974	64	130	810	122	2602	278	0.218270484
NP_001104026.1	filamin-A isoform 2	FLNA	2316	0.828	52	105	701	97	2647	280.6	4.27E-06
NP_006280.3	talin-1	TLN1	7094	0.675	50	93	484	93	2541	269.6	0.000306609
NP_000218.3	laminin subunit alpha-3 isoform 2 precursor	LAMA3	3909	2.524	53	79	736	79	1724	190.3	0.000101966
NP_001367.2	cytoplasmic dynein 1 heavy chain 1	DYNC1H1	1778	0.619	33	125	423	125	4646	532.1	1.54E-05
NP_004095.4	fatty acid synthase	FASN	2194	0.502	46	86	533	85	2511	273.3	2.14E-06
XP_005257069.1	clathrin heavy chain 1 isoform X1	CLTC	1213	0.804	51	67	372	67	1682	192.3	0.01678764
XP_006717309.1	spectrin alpha chain, non-erythrocytic 1 isoform X2	SPTAN1	6709	1.242	42	87	293	87	2505	288.4	0.001874093
XP_006720633.1	pyruvate kinase PKM isoform X1	PKM	5315	0.441	61	37	583	4	605	65.8	0.000373163
XP_006717563.1	vimentin isoform X1	VIM	7431	1.547	89	55	920	47	466	53.6	0.000186983
XP_005273181.1	laminin subunit beta-3 isoform X1	LAMB3	3914	2.693	55	49	542	49	1172	129.5	8.51E-05
NP_008835.5	DNA-dependent protein kinase catalytic subunit isoform 1	PRKDC	5591	1.436	25	101	333	101	4128	468.8	0.001107841
NP_005545.1	keratin, type II cytoskeletal 6C	KRT6A	3853	1.275	68	57	840	6	564	60	0.052717729
NP_001193725.1	pyruvate kinase PKM isoform c	PKM	5315	0.259	56	35	510	2	605	65.9	1.51E-05
NP_005546.2	keratin, type II cytoskeletal 6B	KRT6B	3854	1.898	67	54	777	5	564	60	0.001859798
NP_733821.1	lamin isoform A	LMNA	4000	2.256	65	53	459	51	664	74.1	2.95E-05
NP_004915.2	alpha-actinin-4 isoform 1	ACTN4	81	0.949	68	58	417	39	911	104.8	0.176668941
NP_003290.1	endoplasmin precursor	HSP90B1	7184	2.003	62	55	560	53	803	92.4	0.000392761
NP_002147.2	60 kDa heat shock protein, mitochondrial	HSPD1	3329	1.829	72	42	479	42	573	61	0.000111785
NP_001093.1	alpha-actinin-1 isoform b	ACTN1	87	0.824	65	55	421	36	892	103	0.002506357
NP_005338.1	endoplasmic reticulum chaperone BiP precursor	HSPA5	3309	1.855	61	48	612	45	654	72.3	1.78E-05
NP_001186883.1	actin, cytoplasmic 2	ACTG1	71	0.952	63	25	1505	9	375	41.8	0.109018254
NP_001017963.2	heat shock protein HSP 90-alpha isoform 1	HSP90AA1	3320	0.513	48	51	409	32	854	98.1	0.000844344
NP_002017.1	fibronectin isoform 3 preproprotein	FN1	2335	3.604	36	57	309	57	2355	259.1	2.04E-05
NP_003861.1	ras GTPase-activating-like protein IQGAP1	IQGAP1	8826	0.787	49	66	255	66	1657	189.1	0.009091348
XP_024302189.1	heat shock protein HSP 90-beta isoform X1	HSP90AB1	3326	0.533	54	46	410	26	724	83.2	0.000135935
NP_006079.1	tubulin beta-4B chain	TUBB4B	10383	0.451	71	26	436	3	445	49.8	0.003613154
NP_001952.1	elongation factor 2	EEF2	1938	0.421	59	43	370	43	858	95.3	4.46E-05
NP_821133.1	tubulin beta chain isoform b	TUBB	203068	0.35	71	26	445	5	444	49.6	0.00023078
NP_005553.2	laminin subunit gamma-2 isoform a precursor	LAMC2	3918	3.517	41	47	442	47	1193	130.9	3.05E-05
NP_001036041.1	ribosome-binding protein 1	RRBP1	6238	2.272	58	52	230	51	977	108.6	0.000107914
NP_009057.1	transitional endoplasmic reticulum ATPase isoform 1	VCP	7415	0.775	58	42	336	41	806	89.3	0.001051863
NP_004406.2	desmoplakin isoform I	DSP	1832	0.961	31	72	217	72	2871	331.6	0.351084033
NP_001677.2	ATP synthase subunit beta, mitochondrial precursor	ATP5F1B	506	1.484	65	24	372	24	529	56.5	0.000314628
XP_011541100.1	heat shock cognate 71 kDa protein isoform X1	HSPA8	3312	0.702	63	39	378	24	646	70.9	0.001270753
NP_000415.2	keratin, type II cytoskeletal 5	KRT5	3852	0.569	57	47	600	29	590	62.3	0.001306815
NP_000909.2	protein disulfide-isomerase precursor	P4HB	5034	2.388	74	42	345	42	508	57.1	0.00016803
XP_005264574.1	spectrin beta chain, non-erythrocytic 1 isoform X1	SPTBN1	6711	1.273	36	72	201	65	2364	274.4	0.049165344
NP_002037.2	glyceraldehyde-3-phosphate dehydrogenase isoform 1	GAPDH	2597	0.441	68	26	607	26	335	36	2.47E-05
NP_001310912.1	catenin alpha-1 isoform 1	CTNNA1	1495	1.445	53	38	172	31	906	100	0.004285984
NP_002267.2	keratin, type I cytoskeletal 19	KRT19	3880	1.611	83	43	589	28	400	44.1	1.01E-05
NP_000173.2	trifunctional enzyme subunit alpha, mitochondrial precursor	HADHA	3030	1.843	58	37	251	37	763	82.9	7.91E-05
NP_001419.1	alpha-enolase isoform 1	ENO1	2023	0.386	78	33	698	30	434	47.1	1.99E-05
NP_000517.2	keratin, type I cytoskeletal 14	KRT14	3861	0.426	72	42	523	16	472	51.6	1.41E-05
NP_001393.1	elongation factor 1-alpha 1	EEF1A1	1915	0.423	70	27	345	17	462	50.1	2.04E-05
NP_005304.3	protein disulfide-isomerase A3 precursor	PDIA3	2923	1.766	61	37	299	37	505	56.7	0.000572192
XP_016885266.1	ubiquitin-like modifier-activating enzyme 1 isoform X1	UBA1	7317	0.786	39	32	212	32	1109	123.1	0.028659453
NP_004902.1	protein disulfide-isomerase A4 precursor	PDIA4	9601	2.283	55	43	301	43	645	72.9	4.32E-06
NP_001060.1	tubulin beta-2A chain isoform 1	TUBB2A	7280	0.518	56	22	334	2	445	49.9	1.50E-05
NP_005337.2	heat shock 70 kDa protein 1B	HSPA1B	3304	0.885	60	36	219	25	641	70	0.000161517
NP_001002858.1	annexin A2 isoform 1	ANXA2	302	0.705	79	37	640	37	357	40.4	0.000729494
NP_573566.2	leucine-rich PPR motif-containing protein, mitochondrial precursor	LRPPRC	10128	1.658	43	54	209	54	1394	157.8	0.000723468
NP_000413.1	keratin, type I cytoskeletal 17	KRT17	3872	1.693	80	43	418	22	432	48.1	8.05E-05
XP_011522177.1	myosin-10 isoform X7	MYH10	4628	0.571	28	52	211	32	2037	235.7	0.00320755
XP_005269750.1	myoferlin isoform X1	MYOF	26509	1.332	36	61	194	61	2080	236.5	0.001665896
NP_001243211.1	keratin, type II cytoskeletal 8 isoform 1	KRT8	3856	1.765	61	36	290	26	511	56.6	0.000166549
XP_005246629.1	unconventional myosin-Ib isoform X1	MYO1B	4430	1.554	42	44	228	44	1136	131.9	1.46E-05
XP_011529261.1	moesin isoform X1	MSN	4478	0.562	61	47	289	34	610	71.3	7.28E-07
NP_733765.1	sarcoplasmic/endoplasmic reticulum calcium ATPase 2 isoform b	ATP2A2	488	1.703	35	34	191	34	1042	114.7	0.000155596
XP_011532356.1	transketolase isoform X1	TKT	7086	0.741	52	30	214	30	631	68.8	0.003459082
XP_016872584.1	hypoxia up-regulated protein 1 isoform X1	HYOU1	10525	1.989	38	36	208	36	1000	111.3	1.66E-05
NP_000684.2	aldehyde dehydrogenase family 1 member A3 isoform 1	ALDH1A3	220	0.387	70	32	283	28	512	56.1	3.49E-05
NP_006000.2	tubulin alpha-1A chain isoform 1	TUBA1A	7846	0.442	65	24	433	2	451	50.1	0.464763068
NP_001001937.1	ATP synthase subunit alpha, mitochondrial isoform a precursor	ATP5F1A	498	1.641	59	32	355	32	553	59.7	1.06E-05
XP_005261035.1	interferon-induced GTP-binding protein Mx1 isoform X1	MX1	4599	0.196	57	32	207	29	662	75.5	1.74E-05
NP_001275905.1	X-ray repair cross-complementing protein 6 isoform 1	XRCC6	2547	1.006	55	31	210	31	609	69.8	0.730028633
NP_001348.2	ATP-dependent RNA helicase A	DHX9	1660	1.579	38	36	161	36	1270	140.9	2.50E-05
NP_001146.2	annexin A6 isoform 1	ANXA6	309	0.383	61	38	196	2	673	75.8	0.000789561
XP_024308600.1	vigilin isoform X1	HDLBP	3069	1.285	43	44	170	44	1268	141.4	0.012304508
NP_006827.1	eIF-2-alpha kinase activator GCN1	GCN1	10985	0.636	21	42	149	42	2671	292.5	0.014263294
NP_005991.1	tubulin alpha-4A chain isoform 1	TUBA4A	7277	0.417	60	22	346	5	448	49.9	0.000219547
XP_024308018.1	aconitate hydratase, mitochondrial isoform X1	ACO2	50	1.504	50	35	159	35	780	85.4	0.001762398
NP_066964.1	X-ray repair cross-complementing protein 5	XRCC5	7520	1.115	45	30	189	30	732	82.7	0.333015226
NP_001290043.1	tubulin alpha-1C chain isoform a	TUBA1C	84790	0.816	52	23	403	2	519	57.7	0.138255361
NP_005989.3	T-complex protein 1 subunit gamma isoform a	CCT3	7203	0.758	54	29	158	29	545	60.5	0.116574461
NP_004125.3	stress-70 protein, mitochondrial precursor	HSPA9	3313	1.277	55	33	203	33	679	73.6	0.017892447
NP_110379.2	T-complex protein 1 subunit alpha isoform a	TCP1	6950	0.856	61	26	141	26	556	60.3	0.142173998
NP_006422.1	T-complex protein 1 subunit beta isoform 1	CCT2	10576	0.77	68	32	207	32	535	57.5	0.001741427
NP_001290203.1	ATP-citrate synthase isoform 3	ACLY	47	0.487	36	36	169	36	1155	126.1	7.75E-06
NP_004334.1	calreticulin precursor	CALR	811	1.765	63	24	233	24	417	48.1	0.000404376
NP_001230106.1	fructose-bisphosphate aldolase A isoform 2	ALDOA	226	0.437	69	28	284	23	418	45.2	0.000648526
NP_000692.2	sodium/potassium-transporting ATPase subunit alpha-1 isoform a	ATP1A1	476	1.526	31	33	167	33	1023	112.8	0.02078266
NP_006436.3	pre-mRNA-processing-splicing factor 8	PRPF8	10594	1.305	26	52	141	52	2335	273.4	0.017014835
NP_002145.3	heat shock 70 kDa protein 4	HSPA4	3308	0.437	49	34	133	32	840	94.3	2.92E-05
NP_114032.2	heterogeneous nuclear ribonucleoprotein U isoform a	HNRNPU	3192	1.619	34	30	191	30	825	90.5	4.60E-05
NP_002941.1	dolichyl-diphosphooligosaccharide--protein glycosyltransferase subunit 1 precursor	RPN1	6184	2.095	58	33	187	33	607	68.5	2.94E-05
NP_001350043.1	annexin A6 isoform 3	ANXA6	309	0.684	61	37	189	1	667	75.2	0.495306743
NP_001072989.1	kinectin isoform a	KTN1	3895	1.92	38	48	143	48	1357	156.2	0.000522035
NP_000282.1	phosphoglycerate kinase 1	PGK1	5230	0.511	74	27	188	27	417	44.6	7.37E-05
XP_016858683.1	MICOS complex subunit MIC60 isoform X1	IMMT	10989	1.555	47	36	141	36	758	83.6	0.004698137
NP_006077.2	tubulin beta-3 chain isoform 1	TUBB3	10381	0.589	41	18	286	2	450	50.4	0.211922613
XP_024304524.1	serpin H1 isoform X1	SERPINH1	871	1.678	57	20	185	20	418	46.4	0.000801045
NP_006816.2	cytoskeleton-associated protein 4	CKAP4	10970	2.063	54	30	196	30	602	66	4.85E-05
NP_938148.1	neutral alpha-glucosidase AB isoform 2 precursor	GANAB	23193	1.815	48	38	194	38	944	106.8	6.20E-05
NP_001152759.1	triosephosphate isomerase isoform 2	TPI1	7167	0.49	74	20	199	20	286	30.8	1.77E-05
NP_059447.2	major vault protein isoform 1	MVP	9961	0.63	48	31	141	31	893	99.3	1.04E-05
NP_054733.2	U5 small nuclear ribonucleoprotein 200 kDa helicase	SNRNP200	23020	1.39	24	44	134	44	2136	244.4	0.000386408
NP_001290453.1	tubulin beta-6 chain isoform 1	TUBB6	84617	0.503	63	23	233	13	446	49.8	0.003298214
NP_060918.2	cullin-associated NEDD8-dissociated protein 1 isoform 1	CAND1	55832	0.659	41	40	135	40	1230	136.3	0.00147818
XP_006721929.1	integrin beta-4 isoform X1	ITGB4	3691	1.723	23	35	149	35	1910	211.6	0.002893959
NP_005909.2	malate dehydrogenase, mitochondrial isoform 1 precursor	MDH2	4191	1.922	66	19	220	19	338	35.5	0.000388018
NP_001309294.1	hexokinase-1 isoform a	HK1	3098	1.591	37	34	156	32	952	106.2	0.00011564
XP_005252017.1	heterogeneous nuclear ribonucleoprotein K isoform X1	HNRNPK	3190	0.845	53	22	205	2	464	51	0.548430849
NP_005150.1	actin, alpha cardiac muscle 1 precursor	ACTC1	70	1.86	49	22	575	2	377	42	0.002960693
NP_005372.2	nucleolin	NCL	4691	1.179	41	36	205	36	710	76.6	0.056276486
NP_000405.1	peroxisomal multifunctional enzyme type 2 isoform 2	HSD17B4	3295	1.599	46	26	117	26	736	79.6	0.000402808
NP_001091.1	actin, alpha skeletal muscle	ACTA1	58	1.17	49	21	572	1	377	42	0.936974783
XP_016870146.1	annexin A1 isoform X1	ANXA1	301	0.362	62	22	219	22	357	40.1	0.000129759
XP_011536757.1	LIM domain and actin-binding protein 1 isoform X1	LIMA1	51474	1.203	45	27	136	27	760	85.2	0.010258587
NP_003370.2	ezrin	EZR	7430	0.492	46	32	177	19	586	69.4	0.001069119
NP_001609.2	poly	PARP1	142	1.119	38	34	117	34	1014	113	0.003031903
NP_003312.3	elongation factor Tu, mitochondrial isoform 1 precursor	TUFM	7284	1.581	61	22	121	22	455	49.8	0.00108802
NP_004437.2	bifunctional glutamate/proline--tRNA ligase	EPRS	2058	0.682	33	41	131	41	1512	170.5	0.044305034
NP_057212.1	coatomer subunit gamma-1	COPG1	22820	0.627	42	25	117	23	874	97.7	0.001964422
NP_054706.1	vinculin isoform meta-VCL	VCL	7414	0.641	43	37	150	37	1134	123.7	0.000365979
NP_006421.2	T-complex protein 1 subunit delta isoform a	CCT4	10575	0.806	55	26	125	25	539	57.9	0.07152285
NP_002854.3	glycogen phosphorylase, liver form isoform 1	PYGL	5836	0.397	49	33	133	28	847	97.1	9.64E-06
NP_006420.1	T-complex protein 1 subunit eta isoform a	CCT7	10574	0.764	55	26	142	26	543	59.3	0.000815537
NP_001407.1	eukaryotic initiation factor 4A-I isoform 1	EIF4A1	1973	0.432	52	21	154	12	406	46.1	0.000300336
NP_002799.3	26S proteasome non-ATPase regulatory subunit 2 isoform 1	PSMD2	5708	0.688	39	31	119	31	908	100.1	0.013055703
NP_060090.2	interleukin enhancer-binding factor 3 isoform d	ILF3	3609	1.528	28	23	135	23	898	95.7	0.009598982
NP_055205.2	staphylococcal nuclease domain-containing protein 1	SND1	27044	1.195	44	33	119	33	910	101.9	0.011026237
NP_005959.2	heterogeneous nuclear ribonucleoprotein M isoform a	HNRNPM	4670	1.52	42	31	148	31	730	77.5	0.000546315
NP_056991.2	cytosol aminopeptidase	LAP3	51056	0.495	56	22	92	22	519	56.1	0.025483172
XP_005260998.1	trifunctional purine biosynthetic protein adenosine-3 isoform X1	GART	2618	0.471	38	28	123	28	1010	107.7	0.002272816
NP_003283.2	nucleoprotein TPR	TPR	7175	1.564	24	51	128	51	2363	267.1	0.029465066
NP_001739.2	calpain-2 catalytic subunit isoform 1	CAPN2	824	0.51	50	28	114	28	700	80	3.46E-06
NP_002194.2	integrin alpha-2 precursor	ITGA2	3673	1.685	34	29	124	29	1181	129.2	0.003539304
XP_011531639.1	collagen alpha-1(VII) chain isoform X1	COL7A1	1294	2.223	20	34	106	34	2944	295	0.000805089
NP_003237.2	thrombospondin-1 precursor	THBS1	7057	3.268	28	32	140	30	1170	129.3	2.58E-07
XP_005268883.1	heterogeneous nuclear ribonucleoprotein A1 isoform X1	HNRNPA1	3178	1.141	45	20	165	15	372	38.7	0.314867967
NP_002620.1	phosphoglycerate mutase 1 isoform 1	PGAM1	5223	0.54	72	19	136	19	254	28.8	0.000279713
XP_016865667.1	heterogeneous nuclear ribonucleoprotein Q isoform X4	SYNCRIP	10492	0.923	47	27	136	22	562	62.6	0.615219508
NP_001258142.1	sulfide:quinone oxidoreductase, mitochondrial	SQOR	58472	0.871	55	24	171	24	450	49.9	0.025139993
XP_005252522.1	ATP-dependent 6-phosphofructokinase, platelet type isoform X1	PFKP	5214	0.462	39	29	136	26	835	90.8	0.012104938
NP_001311233.1	dolichyl-diphosphooligosaccharide--protein glycosyltransferase subunit 2 isoform 7 precursor	RPN2	6185	1.546	45	18	90	18	647	71.1	0.010612362
NP_001091868.1	coatomer subunit alpha isoform 1	COPA	1314	0.657	37	35	136	35	1233	139.2	0.000112639
NP_000134.2	fumarate hydratase, mitochondrial	FH	2271	1.252	50	19	113	19	510	54.6	0.037091945
NP_006576.2	T-complex protein 1 subunit theta isoform 1	CCT8	10694	0.859	47	25	133	25	548	59.6	0.006348256
NP_001012680.1	4F2 cell-surface antigen heavy chain isoform b	SLC3A2	6520	0.838	43	24	148	23	631	68.1	0.110690126
NP_001337409.1	adenylyl cyclase-associated protein 1 isoform a	CAP1	10487	0.37	55	23	162	1	475	51.6	0.000855612
XP_005252020.1	heterogeneous nuclear ribonucleoprotein K isoform X3	HNRNPK	3190	1.11	50	21	191	1	440	48.5	0.144348305
NP_006112.3	keratin, type II cytoskeletal 1	KRT1	3848	1.574	38	32	128	25	644	66	0.060193961
NP_004719.2	nucleolar RNA helicase 2 isoform 1	DDX21	9188	1.553	40	26	110	24	783	87.3	0.011921241
NP_001269633.1	protein disulfide-isomerase A6 isoform a	PDIA6	10130	1.68	41	18	158	18	492	53.9	0.000153203
NP_036205.1	T-complex protein 1 subunit epsilon isoform a	CCT5	22948	0.79	58	27	136	26	541	59.6	0.00106875
NP_003533.1	histone H4	HIST1H4C	8364	1.879	55	12	340	12	103	11.4	2.42E-06
NP_001302466.1	L-lactate dehydrogenase B chain isoform LDHBx	LDHB	3945	0.35	54	18	154	17	341	37.4	4.69E-05
NP_001337411.1	adenylyl cyclase-associated protein 1 isoform b	CAP1	10487	0.434	55	23	141	1	474	51.6	0.033514357
NP_055040.2	serine/threonine-protein phosphatase 2A 65 kDa regulatory subunit A alpha isoform isoform 1	PPP2R1A	5518	0.538	44	23	123	18	589	65.3	0.005985087
NP_001158886.1	L-lactate dehydrogenase A chain isoform 3	LDHA	3939	0.454	56	21	151	20	361	39.8	7.22E-05
NP_001341935.1	nucleophosmin isoform 1	NPM1	4869	0.974	49	17	179	17	294	32.6	0.438442432
NP_705935.1	tropomyosin alpha-3 chain isoform Tpm3.1cy	TPM3	7170	1.075	66	27	135	15	248	29	0.475290873
NP_005210.3	protein diaphanous homolog 1 isoform 1	DIAPH1	1729	0.501	31	32	103	32	1272	141.3	0.006856044
XP_005262891.1	protocadherin Fat 1 isoform X1	FAT1	2195	1.639	11	39	102	39	4600	507.2	0.002371243
NP_001309898.1	ornithine aminotransferase, mitochondrial isoform 1 precursor	OAT	4942	2.048	63	20	104	20	439	48.5	2.11E-05
NP_001091974.1	probable ATP-dependent RNA helicase DDX17 isoform 3	DDX17	10521	1.426	40	26	120	19	731	80.4	0.002112064
NP_006133.1	14-3-3 protein sigma	SFN	2810	0.392	63	17	250	13	248	27.8	9.34E-05
NP_002630.2	serpin B5	SERPINB5	5268	0.602	57	20	102	20	375	42.1	0.003952286
NP_001247422.1	radixin isoform 1	RDX	5962	0.656	40	27	142	12	604	71	0.007683443
NP_612429.2	protein AHNAK2 isoform 1	AHNAK2	113146	1.449	25	46	107	45	5795	616.2	0.02863402
NP_001188306.1	alpha-aminoadipic semialdehyde dehydrogenase isoform 2	ALDH7A1	501	1.1	46	18	108	18	511	55.3	0.089372935
NP_004448.2	long-chain-fatty-acid--CoA ligase 3	ACSL3	2181	1.415	37	24	107	21	720	80.4	0.029977659
XP_005272132.1	voltage-dependent anion-selective channel protein 1 isoform X1	VDAC1	7416	1.494	82	18	261	17	283	30.8	0.078990574
NP_001351766.1	C-1-tetrahydrofolate synthase, cytoplasmic isoform 2	MTHFD1	4522	0.47	32	28	103	28	964	104.6	0.000249977
XP_005249786.1	heterogeneous nuclear ribonucleoproteins A2/B1 isoform X1	HNRNPA2B1	3181	1.251	58	23	179	18	353	37.4	0.022766368
NP_056107.1	extended synaptotagmin-1 isoform 2	ESYT1	23344	1.406	33	27	105	27	1104	122.8	0.046992955
NP_001485.2	rab GDP dissociation inhibitor beta isoform 1	GDI2	2665	0.376	56	23	119	15	445	50.6	1.35E-05
NP_000959.2	60S ribosomal protein L4	RPL4	6124	0.798	40	22	121	22	427	47.7	0.08525542
NP_954659.1	matrin-3 isoform a	MATR3	9782	1.429	33	21	126	21	847	94.6	0.00204215
NP_001264153.1	transgelin-2 isoform a	TAGLN2	8407	0.408	65	15	152	14	220	24.4	5.61E-06
NP_002256.2	importin subunit beta-1 isoform 1	KPNB1	3837	0.688	38	25	121	25	876	97.1	0.064464877
NP_002878.2	arginine--tRNA ligase, cytoplasmic	RARS	5917	0.561	47	28	103	28	660	75.3	0.000429362
NP_002071.2	aspartate aminotransferase, mitochondrial isoform 1 precursor	GOT2	2806	1.464	53	21	131	21	430	47.5	0.011103204
NP_064505.1	UDP-glucose:glycoprotein glucosyltransferase 1 precursor	UGGT1	56886	1.586	28	33	100	32	1555	177.1	0.000236922
NP_002517.1	5'-nucleotidase isoform 1 preproprotein	NT5E	4907	1.159	45	22	94	21	574	63.3	0.023491174
NP_005023.2	plastin-3 isoform 1	PLS3	5358	0.411	43	23	117	17	630	70.8	0.000168071
XP_006718627.1	nuclear mitotic apparatus protein 1 isoform X3	NUMA1	4926	1.301	20	30	84	30	2115	238.1	0.000652891
XP_024309291.1	microtubule-associated protein 4 isoform X1	MAP4	4134	0.573	17	33	101	33	2326	248.2	0.003273761
XP_005245768.1	heterogeneous nuclear ribonucleoprotein R isoform X2	HNRNPR	10236	1.172	36	20	105	2	633	70.9	0.729616793
NP_004506.2	interleukin enhancer-binding factor 2 isoform 1	ILF2	3608	1.59	52	15	108	15	390	43	0.000132465
NP_055018.2	AP-2 complex subunit alpha-1 isoform 1	AP2A1	160	1.091	35	27	105	21	977	107.5	0.03189908
NP_002121.4	hydroxymethylglutaryl-CoA synthase, cytoplasmic isoform 1	HMGCS1	3157	0.478	43	19	89	19	520	57.3	0.000700271
NP_002100.2	histidine--tRNA ligase, cytoplasmic isoform 1	HARS	3035	0.37	57	24	97	19	509	57.4	0.001291451
NP_596867.1	integrin beta-1 isoform 1A precursor	ITGB1	3688	1.572	37	21	121	21	798	88.4	0.000455237
XP_016869299.1	14-3-3 protein zeta/delta isoform X1	YWHAZ	7534	0.481	60	17	211	12	245	27.7	4.47E-05
NP_038470.1	stomatin-like protein 2, mitochondrial isoform a	STOML2	30968	1.664	52	16	88	16	356	38.5	0.009808017
NP_001914.3	DNA damage-binding protein 1	DDB1	1642	1.176	34	35	111	35	1140	126.9	0.06552482
NP_954657.1	keratin, type I cytoskeletal 18	KRT18	3875	1.261	61	27	157	25	430	48	0.102984882
NP_005403.2	serine hydroxymethyltransferase, mitochondrial isoform 1 precursor	SHMT2	6472	1.216	51	21	111	21	504	56	0.074779498
XP_005249419.1	valine--tRNA ligase isoform X1	VARS	7407	0.725	28	22	96	22	1265	140.5	0.016428718
NP_001303249.1	procollagen-lysine,2-oxoglutarate 5-dioxygenase 1 isoform 1 precursor	PLOD1	5351	2.519	33	23	128	22	774	88.2	0.000296145
NP_002853.2	glycogen phosphorylase, brain form	PYGB	5834	0.592	35	26	83	21	843	96.6	0.105733185
NP_004491.2	heterogeneous nuclear ribonucleoproteins C1/C2 isoform b	HNRNPC	3183	1.459	44	18	136	18	293	32.3	1.40E-05
NP_001136067.1	prolyl 4-hydroxylase subunit alpha-1 isoform 2 precursor	P4HA1	5033	2.318	49	23	93	23	534	61	0.000318932
NP_004238.3	116 kDa U5 small nuclear ribonucleoprotein component isoform a	EFTUD2	9343	1.208	34	24	101	24	972	109.4	0.063074599
NP_116126.3	lamin-B2	LMNB2	84823	1.862	43	32	128	26	620	69.9	9.22E-07
NP_005564.1	lamin-B1 isoform 1	LMNB1	4001	1.616	50	28	113	22	586	66.4	0.02545046
NP_005498.1	cofilin-1	CFL1	1072	0.429	70	14	130	11	166	18.5	0.001196373
NP_006635.2	heat shock protein 105 kDa isoform 1	HSPH1	10808	0.563	34	25	81	22	858	96.8	0.025146265
NP_003741.1	eukaryotic translation initiation factor 3 subunit A	EIF3A	8661	0.603	25	30	100	30	1382	166.5	0.000787327
NP_001753.1	T-complex protein 1 subunit zeta isoform a	CCT6A	908	0.845	48	22	127	22	531	58	0.000494977
NP_006817.1	14-3-3 protein theta	YWHAQ	10971	0.549	56	16	183	10	245	27.7	0.016921576
NP_004035.2	bifunctional purine biosynthesis protein PURH	ATIC	471	0.524	50	21	94	21	592	64.6	6.96E-05
NP_005013.1	profilin-1	PFN1	5216	0.269	74	12	212	12	140	15	0.000365664
NP_061816.1	tubulin alpha-8 chain isoform 1	TUBA8	51807	0.433	37	13	246	1	449	50.1	0.01382244
NP_004484.1	3-hydroxyacyl-CoA dehydrogenase type-2 isoform 1	HSD17B10	3028	1.222	84	15	89	15	261	26.9	0.014851604
NP_002566.1	plasminogen activator inhibitor 2	SERPINB2	5055	0.308	56	17	91	17	415	46.6	6.82E-05
NP_006752.1	14-3-3 protein epsilon	YWHAE	7531	0.411	60	17	167	14	255	29.2	0.00170516
XP_016873357.1	pyruvate carboxylase, mitochondrial isoform X1	PC	5091	1.455	30	27	78	27	1178	129.6	0.000209435
NP_000962.2	60S ribosomal protein L7 isoform 1	RPL7	6129	0.866	53	18	122	18	248	29.2	0.041631195
NP_001274522.1	chloride intracellular channel protein 1	CLIC1	1192	0.395	78	16	118	15	241	26.9	0.000463516
XP_011538773.1	heterogeneous nuclear ribonucleoprotein R isoform X1	HNRNPR	10236	0.746	36	19	104	1	636	71.2	0.84901864
NP_003281.1	tropomyosin alpha-4 chain isoform Tpm4.2cy	TPM4	7171	0.958	63	26	112	5	248	28.5	0.862677227
NP_000349.1	transforming growth factor-beta-induced protein ig-h3 precursor	TGFBI	7045	1.856	39	22	89	22	683	74.6	0.000598928
NP_001276718.1	glucose-6-phosphate isomerase isoform 3	GPI	2821	0.396	41	21	111	21	597	67.2	2.87E-05
NP_001054.1	serotransferrin isoform 1 precursor	TF	7018	1.653	42	29	87	29	698	77	0.051706211
NP_000201.2	integrin alpha-6 isoform b preproprotein	ITGA6	3655	1.711	29	25	84	25	1073	119.4	0.00139835
NP_001015001.1	creatine kinase U-type, mitochondrial isoform 1 precursor	CKMT1A	548596	1.358	47	15	105	15	417	47	0.007557726
NP_001145.1	annexin A5	ANXA5	308	0.329	63	22	138	21	320	35.9	4.05E-05
NP_006308.3	brain acid soluble protein 1	BASP1	10409	2.367	92	16	67	16	227	22.7	0.004308457
XP_016867690.1	laminin subunit beta-1 isoform X1	LAMB1	3912	1.623	17	25	80	25	1810	200.3	0.004704917
NP_001349.2	pre-mRNA-splicing factor ATP-dependent RNA helicase DHX15	DHX15	1665	1.025	35	25	90	25	795	90.9	0.296660854
XP_011542031.1	core histone macro-H2A.1 isoform X1	H2AFY	9555	1.594	43	13	108	11	372	39.6	0.003115124
NP_004784.2	lon protease homolog, mitochondrial isoform 1 precursor	LONP1	9361	1.771	35	25	90	25	959	106.4	0.003772002
NP_644671.1	signal transducer and activator of transcription 1-alpha/beta isoform beta	STAT1	6772	0.692	38	23	97	1	712	83	0.055533943
NP_005262.1	glutamate dehydrogenase 1, mitochondrial isoform a precursor	GLUD1	2746	1.769	42	21	89	21	558	61.4	0.000842237
NP_036226.1	peroxiredoxin-5, mitochondrial isoform L precursor	PRDX5	25824	0.913	57	12	112	12	214	22	0.141116924
NP_000117.1	electron transfer flavoprotein subunit alpha, mitochondrial isoform a	ETFA	2108	1.997	60	13	97	13	333	35.1	0.000123903
XP_016860272.1	signal transducer and activator of transcription 1-alpha/beta isoform X1	STAT1	6772	0.846	37	23	97	1	752	87.6	0.801652051
NP_940978.2	agrin isoform 2 precursor	AGRN	375790	1.422	19	26	75	26	2045	214.7	0.223920569
NP_001025177.1	AP-2 complex subunit beta isoform a	AP2B1	163	0.958	36	26	92	15	951	105.6	0.477507573
XP_024307077.1	transcription intermediary factor 1-beta isoform X1	TRIM28	10155	1.087	32	23	108	23	872	92.4	0.40013124
NP_001181876.1	eukaryotic translation initiation factor 4 gamma 1 isoform 6	EIF4G1	1981	0.578	20	27	84	27	1606	176.1	0.011118387
XP_016880077.1	junction plakoglobin isoform X1	JUP	3728	1.338	43	23	90	18	762	83.6	0.070853737
NP_003079.1	fascin	FSCN1	6624	0.501	54	20	98	20	493	54.5	0.00019905
NP_891988.1	procollagen-lysine,2-oxoglutarate 5-dioxygenase 2 isoform 1 precursor	PLOD2	5352	1.389	36	24	97	24	758	87	0.001529499
NP_005882.2	acetyl-CoA acetyltransferase, cytosolic isoform 1	ACAT2	39	0.568	56	14	65	14	397	41.3	0.002333378
XP_024303862.1	delta-1-pyrroline-5-carboxylate synthase isoform X1	ALDH18A1	5832	2.404	37	25	96	25	829	91.1	4.02E-05
NP_001184222.1	dihydropyrimidinase-related protein 2 isoform 1	DPYSL2	1808	0.818	43	18	78	15	677	73.5	0.09968501
NP_004512.1	kinesin-1 heavy chain	KIF5B	3799	0.728	30	22	61	22	963	109.6	0.034481236
XP_005261040.1	interferon-induced GTP-binding protein Mx2 isoform X1	MX2	4600	0.555	34	22	95	19	715	82	0.021777946
NP_001307526.1	probable ATP-dependent RNA helicase DDX5 isoform b	DDX5	1655	1.114	40	26	97	19	614	69	0.00060419
NP_003698.1	ruvB-like 1 isoform 1	RUVBL1	8607	0.726	44	14	83	14	456	50.2	3.47E-06
XP_005259654.1	polypyrimidine tract-binding protein 1 isoform X1	PTBP1	5725	0.961	35	12	91	9	559	59.8	0.750048622
NP_001092315.2	annexin A8-like protein 1 isoform 1	ANXA8L1	728113	0.23	62	20	99	3	327	36.9	0.000212128
NP_064502.9	leucine--tRNA ligase, cytoplasmic isoform 1	LARS	51520	0.628	28	28	95	28	1176	134.4	8.00E-06
NP_444505.1	60S acidic ribosomal protein P0	RPLP0	6175	0.723	48	11	134	11	317	34.3	0.013141322
XP_011538398.1	annexin A8 isoform X1	ANXA8	653145	0.305	46	20	106	3	448	49.6	0.000195562
NP_001268644.1	prohibitin isoform 1	PHB	5245	1.781	71	15	146	15	272	29.8	0.006487832
NP_000958.1	60S ribosomal protein L3 isoform a	RPL3	6122	0.81	38	19	98	19	403	46.1	0.001154724
XP_011531105.1	trifunctional enzyme subunit beta, mitochondrial isoform X1	HADHB	3032	1.891	49	21	96	21	474	51.3	0.001886318
NP_002215.2	inositol 1,4,5-trisphosphate receptor type 3	ITPR3	3710	1.626	12	26	81	22	2671	303.9	0.037558229
NP_000996.2	40S ribosomal protein S3 isoform 1	RPS3	6188	0.638	68	17	116	17	243	26.7	0.009534992
XP_016864369.1	WD repeat-containing protein 1 isoform X1	WDR1	9948	0.607	44	22	90	22	659	72	0.00092572
NP_036450.1	mitochondrial proton/calcium exchanger protein precursor	LETM1	3954	1.566	31	18	63	18	739	83.3	0.004420518
NP_056474.2	ribosomal L1 domain-containing protein 1	RSL1D1	26156	1.654	42	23	85	23	490	54.9	0.000401128
NP_006089.1	receptor of activated protein C kinase 1	RACK1	10399	0.696	77	18	95	18	317	35.1	0.002719631
NP_001078927.1	catenin delta-1 isoform 1ABC	CTNND1	1500	1.571	29	24	87	24	968	108.1	0.00250601
NP_001137533.1	coatomer subunit beta	COPB1	1315	0.559	24	17	77	17	953	107.1	0.000915196
NP_037506.2	programmed cell death 6-interacting protein isoform 1	PDCD6IP	10015	0.695	44	30	85	30	868	96	0.002824655
XP_016873595.1	ribonuclease inhibitor isoform X1	RNH1	6050	0.571	59	18	71	18	461	49.9	0.000121455
NP_000998.1	40S ribosomal protein S4, X isoform	RPS4X	6191	0.656	55	17	125	17	263	29.6	0.000606878
NP_663782.2	AP-1 complex subunit beta-1 isoform b	AP1B1	162	0.767	33	24	76	13	939	103.5	0.090742836
NP_005548.2	keratin, type I cytoskeletal 16	KRT16	3868	0.976	37	20	231	5	473	51.2	0.862824843
XP_005249816.1	2-oxoglutarate dehydrogenase, mitochondrial isoform X1	OGDH	4967	1.274	28	22	79	18	1038	117.6	0.117814731
NP_001395.1	elongation factor 1-gamma	EEF1G	1937	0.629	47	19	99	19	437	50.1	0.005504717
NP_001087240.1	thioredoxin reductase 1, cytoplasmic isoform 5	TXNRD1	7296	0.474	41	18	70	18	649	70.9	0.015864264
NP_002038.2	glycine--tRNA ligase isoform 1 precursor	GARS	2617	0.437	35	18	72	18	739	83.1	0.000198002
XP_005250918.1	polyadenylate-binding protein 1 isoform X1	PABPC1	26986	0.681	36	21	97	16	636	70.6	0.000813963
XP_024303291.1	gelsolin isoform X5	GSN	2934	1.223	31	21	92	1	731	80.6	0.883249652
NP_001012.1	40S ribosomal protein S17	RPS17	6218	0.398	62	8	73	8	135	15.5	0.000820245
NP_000963.1	60S ribosomal protein L7a	RPL7A	6130	0.821	45	17	97	17	266	30	0.000980338
NP_006383.2	nucleolar protein 56	NOP56	10528	1.177	43	20	85	20	594	66	0.032017288
NP_068814.2	heat shock-related 70 kDa protein 2	HSPA2	3306	1.011	22	16	117	2	639	70	0.645865591
XP_024307247.1	heterogeneous nuclear ribonucleoprotein L isoform X1	HNRNPL	3191	1.75	32	14	101	13	626	68	4.29E-05
NP_001171712.1	voltage-dependent anion-selective channel protein 2 isoform 1	VDAC2	7417	2.091	62	15	147	15	309	33.4	0.000235102
NP_001531.1	heat shock protein beta-1	HSPB1	3315	0.452	80	13	92	13	205	22.8	0.000607145
NP_002798.2	26S proteasome non-ATPase regulatory subunit 1 isoform 1	PSMD1	5707	0.884	31	20	71	20	953	105.8	0.174836049
XP_016870137.1	gelsolin isoform X4	GSN	2934	0.782	31	21	92	1	742	81.9	0.503918436
XP_005259352.1	calpain small subunit 1 isoform X1	CAPNS1	826	0.565	64	12	103	12	322	33.8	0.001793423
XP_016883198.1	adenosylhomocysteinase isoform X2	AHCY	191	0.598	42	20	101	18	432	47.7	0.000699033
NP_036565.2	splicing factor 3B subunit 1 isoform 1	SF3B1	23451	1.198	27	25	54	25	1304	145.7	0.420046175
NP_005712.1	actin-related protein 3 isoform 1	ACTR3	10096	0.824	59	17	81	17	418	47.3	0.000412721
NP_000414.2	keratin, type II cytoskeletal 2 epidermal	KRT2	3849	2.197	35	22	257	12	639	65.4	0.001644115
NP_000843.1	glutathione S-transferase P	GSTP1	2950	0.344	70	10	103	10	210	23.3	0.000137574
XP_005257400.1	keratin, type I cytoskeletal 10 isoform X1	KRT10	3858	2.002	30	20	104	16	624	63.3	0.001421621
NP_001427.2	rRNA 2'-O-methyltransferase fibrillarin	FBL	2091	1.798	51	15	69	15	321	33.8	0.000269642
NP_055102.3	nodal modulator 1 precursor	NOMO1	23420	1.926	23	21	58	21	1222	134.2	0.00055509
NP_060979.2	leucine-rich repeat-containing protein 59	LRRC59	55379	1.974	48	12	84	12	307	34.9	0.000128916
NP_001805.3	dipeptidyl peptidase 1 isoform a preproprotein	CTSC	1075	1.279	18	11	85	11	463	51.8	0.031771349
XP_006710589.1	microtubule-actin cross-linking factor 1 isoform X14	MACF1	23499	0.913	6	31	65	28	7595	860.9	0.484257432
NP_001269374.1	EH domain-containing protein 1 isoform 2	EHD1	10938	0.565	42	19	71	13	548	61.9	0.006666358
NP_005310.1	histone H1.2	HIST1H1C	3006	2.26	39	15	115	3	213	21.4	0.02565586
NP_005312.1	histone H1.4	HIST1H1E	3008	2.256	42	16	116	4	219	21.9	0.00485882
NP_004981.2	methionine--tRNA ligase, cytoplasmic	MARS	4141	0.645	30	19	64	19	900	101.1	0.000197678
NP_057226.1	very-long-chain 3-oxoacyl-CoA reductase	HSD17B12	51144	2.04	43	11	65	11	312	34.3	0.052421166
NP_000393.4	glucose-6-phosphate 1-dehydrogenase isoform a	G6PD	2539	0.585	45	22	97	22	545	62.4	0.003401057
NP_722550.1	reticulon-4 isoform B	RTN4	57142	1.052	41	10	110	10	373	40.3	0.140930987
NP_002152.2	isoleucine--tRNA ligase, cytoplasmic	IARS	3376	0.657	29	27	80	27	1262	144.4	0.028091612
NP_001347.3	ATP-dependent RNA helicase DDX3X isoform 1	DDX3X	1654	0.947	32	18	67	18	662	73.2	0.195717266

Patil et al,2020. Proteomic and phosphoproteomic profiling of shammah-induced signaling in oral keratinocytes

Supplementary Table S1: List of proteins identified and quantified in OKF6/TERT1-Shammah and OKF6/TERT1-Parental total proteome using TMT-based quantitative proteomic approach

Accession	Protein Description	Gene Symbol	Gene ID	Fold Change	Coverage [%]	# Peptides	# PSMs	# Unique Peptides	# AAs	MW [kDa]	p-value
XP_006712157.1	exportin-1 isoform X1	XPO1	7514	0.631	23	19	72	19	1071	123.3	0.017930048
NP_001141.2	aminopeptidase N precursor	ANPEP	290	0.896	24	20	67	20	967	109.5	0.054193022
NP_001138632.1	tropomyosin alpha-4 chain isoform Tpm4.1cy	TPM4	7171	0.948	54	23	95	1	284	32.7	0.821650973
NP_005808.3	perilipin-3 isoform 1	PLIN3	10226	0.659	44	11	74	11	434	47	0.05628748
NP_004930.1	ATP-dependent RNA helicase DDX1	DDX1	1653	0.931	35	19	55	19	740	82.4	0.798253782
NP_001245366.1	threonine--tRNA ligase, cytoplasmic isoform 1	TARS	6897	0.383	41	25	82	25	723	83.4	0.002344317
NP_001307.2	exportin-2 isoform 1	CSE1L	1434	0.558	25	21	70	21	971	110.3	0.000634823
NP_060530.3	isoleucine--tRNA ligase, mitochondrial precursor	IARS2	55699	1.524	29	24	67	24	1012	113.7	0.031405922
NP_002296.1	galectin-1	LGALS1	3956	1.715	81	10	348	10	135	14.7	4.65E-06
NP_001138303.1	prohibitin-2 isoform 1	PHB2	11331	1.715	63	17	110	17	299	33.3	0.00322352
NP_001244.1	cell division cycle 5-like protein	CDC5L	988	1.295	28	19	69	19	802	92.2	0.054908069
NP_004159.2	succinate dehydrogenase	SDHA	6389	1.043	38	18	65	18	664	72.6	0.609545776
NP_001165131.1	NADH-cytochrome b5 reductase 3 isoform 3	CYB5R3	1727	1.59	49	12	80	12	334	38.2	0.060754642
NP_001527.3	protein arginine N-methyltransferase 1 isoform 1	PRMT1	3276	0.729	45	17	68	17	371	42.4	0.021459396
NP_006254.1	proteasome activator complex subunit 1 isoform 1	PSME1	5720	0.515	55	15	61	15	249	28.7	7.65E-05
NP_036558.3	splicing factor 3B subunit 3	SF3B3	23450	1.271	19	20	82	20	1217	135.5	0.012012997
XP_024309124.1	catenin beta-1 isoform X1	CTNNB1	1499	1.32	29	20	75	15	781	85.4	0.047006727
NP_001229236.1	presequence protease, mitochondrial isoform 1 precursor	PITRM1	10531	1.338	28	21	59	21	1038	117.4	0.117852048
NP_001107228.1	thymidine phosphorylase isoform 1 precursor	TYMP	1890	0.401	43	15	86	15	482	49.9	0.007062044
NP_003356.2	cytochrome b-c1 complex subunit 1, mitochondrial precursor	UQCRC1	7384	1.515	34	12	64	12	480	52.6	0.00026543
XP_011527330.1	protein-glutamine gamma-glutamyltransferase 2 isoform X1	TGM2	7052	0.598	35	19	84	19	687	77.3	0.000681305
XP_011537840.1	protein RRP5 homolog isoform X1	PDCD11	22984	1.425	16	23	59	23	1872	208.7	0.005222197
NP_001257411.1	26S proteasome non-ATPase regulatory subunit 11	PSMD11	5717	0.793	45	19	65	19	422	47.4	0.018926084
NP_001269468.1	guanine nucleotide-binding protein G(I)/G(S)/G(T) subunit beta-1 isoform 1	GNB1	2782	1.452	51	11	71	6	340	37.4	0.037793741
NP_036611.2	14-3-3 protein gamma	YWHAG	7532	0.67	47	14	166	8	247	28.3	0.0193384
XP_006713191.1	inosine-5'-monophosphate dehydrogenase 2 isoform X1	IMPDH2	3615	0.459	24	12	46	11	608	66.1	0.004675974
NP_001900.1	cathepsin D preproprotein	CTSD	1509	1.962	33	10	90	10	412	44.5	0.026712172
NP_002201.1	integrin alpha-V isoform 1 preproprotein	ITGAV	3685	1.978	24	20	67	20	1048	116	0.002238085
NP_006494.1	26S proteasome regulatory subunit 6B isoform 1	PSMC4	5704	0.881	51	17	85	17	418	47.3	0.238295472
NP_000261.2	purine nucleoside phosphorylase	PNP	4860	0.469	52	11	65	11	289	32.1	0.000934642
XP_011511565.1	GMP synthase	GMPS	8833	0.642	38	18	54	18	699	77.4	0.000122238
NP_002159.2	isocitrate dehydrogenase	IDH2	3418	1.966	45	18	62	17	452	50.9	0.003843325
NP_005549.2	ladinin-1	LAD1	3898	1.485	42	20	71	20	517	57.1	0.000493025
XP_016857542.1	splicing factor, proline- and glutamine-rich isoform X1	SFPQ	6421	1.504	28	18	81	17	707	76.1	2.24E-05
XP_016868908.1	aspartyl/asparaginyl beta-hydroxylase isoform X1	ASPH	444	1.641	26	18	66	18	800	90.4	0.000541752
NP_001074248.1	unconventional myosin-Ic isoform a	MYO1C	4641	0.9	24	21	68	21	1063	121.6	0.493311776
NP_002794.1	26S proteasome regulatory subunit 7 isoform 1	PSMC2	5701	0.863	44	21	83	21	433	48.6	0.236161839
NP_001934.2	desmoglein-2 preproprotein	DSG2	1829	1.297	27	19	53	19	1118	122.2	0.056115998
NP_001018146.1	NME1-NME2 protein	NME1-NME2	654364	0.408	72	14	108	6	267	30.1	0.002318677
NP_000933.1	peptidyl-prolyl cis-trans isomerase B precursor	PPIB	5479	1.588	58	16	131	16	216	23.7	0.004934819
NP_002262.3	importin-5	IPO5	3843	0.548	22	18	67	15	1115	125.5	0.000489599
NP_001300870.1	transforming protein RhoA isoform 1 precursor	RHOA	387	0.965	60	10	82	2	193	21.8	0.906116441
NP_006301.3	puromycin-sensitive aminopeptidase isoform 1	NPEPPS	9520	0.696	24	19	65	19	919	103.2	0.004725023
XP_011539620.1	basement membrane-specific heparan sulfate proteoglycan core protein isoform X1	HSPG2	3339	1.952	8	24	57	24	4574	488.2	0.00246391
NP_006293.2	mannosyl-oligosaccharide glucosidase isoform 1	MOGS	7841	1.817	27	18	78	18	837	91.9	0.000998159
XP_024309513.1	ras-related protein Rab-7a isoform X1	RAB7A	7879	1.3	72	15	85	15	207	23.5	5.00E-05
NP_001265393.1	dynamin-1-like protein isoform 5	DNM1L	10059	0.584	43	21	74	21	749	83.3	0.001381641
NP_001317178.1	heterogeneous nuclear ribonucleoprotein A3 isoform a	HNRNPA3	220988	1.319	39	19	88	17	378	39.6	0.162672054
NP_570850.2	dynamin-like 120 kDa protein, mitochondrial isoform 8	OPA1	4976	1.621	23	18	61	18	1015	117.7	0.050734036
NP_003839.2	succinate--CoA ligase	SUCLG2	8801	1.42	39	16	60	16	432	46.5	0.005180834
XP_005257602.2	E3 ubiquitin-protein ligase RNF213 isoform X1	RNF213	57674	0.575	6	24	62	24	5256	596.1	0.01181944
XP_011531289.1	interferon-induced, double-stranded RNA-activated protein kinase isoform X1	EIF2AK2	5610	0.442	32	15	71	15	551	62.1	0.004340049
NP_006746.1	transaldolase	TALDO1	6888	0.661	45	18	81	18	337	37.5	0.010707007
NP_031389.3	non-POU domain-containing octamer-binding protein isoform 1	NONO	4841	1.458	42	18	81	16	471	54.2	0.125221808
XP_005264059.1	E3 SUMO-protein ligase RanBP2 isoform X1	RANBP2	5903	1.383	8	24	67	24	3258	361.8	0.0169955
NP_001958.2	eukaryotic initiation factor 4A-II	EIF4A2	1974	1.318	54	19	100	10	407	46.4	0.323644505
NP_078938.2	RNA cytidine acetyltransferase isoform a	NAT10	55226	1.388	27	22	66	22	1025	115.6	0.002027238
NP_066272.1	PDZ and LIM domain protein 1	PDLIM1	9124	0.696	67	17	56	17	329	36	0.057960506
XP_005259725.1	far upstream element-binding protein 2 isoform X1	KHSRP	8570	1.504	30	18	58	16	747	76.6	0.027441388
XP_011532966.1	calnexin isoform X3	CANX	821	1.42	35	20	110	20	646	73.4	0.012148348
NP_001291217.1	40S ribosomal protein SA isoform 2	RPSA	3921	0.56	50	10	73	10	300	33.3	0.001069894
NP_005073.2	E3 ubiquitin/ISG15 ligase TRIM25	TRIM25	7706	0.439	33	17	60	17	630	70.9	0.000669659
NP_036364.2	caveolae-associated protein 1	CAVIN1	284119	1.237	30	14	90	14	390	43.5	0.245077849
NP_006818.3	transmembrane emp24 domain-containing protein 10 precursor	TMED10	10972	1.813	44	11	67	11	219	25	0.003360267
NP_009035.3	60S ribosomal protein L10a	RPL10A	4736	0.662	44	11	73	11	217	24.8	0.00011131
NP_002793.2	26S proteasome regulatory subunit 4 isoform a	PSMC1	5700	0.894	53	21	68	20	440	49.2	0.080278545
NP_055555.1	eukaryotic initiation factor 4A-III	EIF4A3	9775	1.333	42	13	71	11	411	46.8	0.032133947
NP_004896.1	peroxiredoxin-6	PRDX6	9588	0.349	58	16	107	16	224	25	0.000764651
XP_011523086.1	keratin, type I cytoskeletal 15 isoform X1	KRT15	3866	2.143	34	18	205	9	463	50	0.000213781
XP_011528188.2	catechol O-methyltransferase isoform X1	COMT	1312	1.233	30	10	57	10	408	45.1	0.110322778
NP_112737.1	heterogeneous nuclear ribonucleoprotein D0 isoform b	HNRNPD	3184	1.841	38	13	84	1	336	36.2	0.539554859
NP_005868.1	splicing factor 3A subunit 1	SF3A1	10291	1.509	30	19	55	19	793	88.8	0.086534791
NP_112738.1	heterogeneous nuclear ribonucleoprotein D0 isoform a	HNRNPD	3184	1.548	36	13	82	1	355	38.4	0.729120301
NP_002795.2	26S proteasome regulatory subunit 6A	PSMC3	5702	0.905	40	13	67	13	439	49.2	0.350832005
XP_005263481.1	protein/nucleic acid deglycase DJ-1 isoform X1	PARK7	11315	0.472	63	12	69	12	189	19.9	0.000260706
NP_001121620.1	transferrin receptor protein 1 isoform 1	TFRC	7037	1.764	29	21	61	21	760	84.8	0.00239635
NP_003396.1	14-3-3 protein eta	YWHAH	7533	0.564	46	15	136	11	246	28.2	0.023835421
XP_006715894.1	calcium-binding mitochondrial carrier protein Aralar2 isoform X1	SLC25A13	10165	1.489	32	14	57	11	686	75.4	0.097796342
NP_001017992.1	beta-actin-like protein 2	ACTBL2	345651	1.079	34	11	200	4	376	42	0.056846726
NP_060705.2	cytosolic non-specific dipeptidase isoform 1	CNDP2	55748	0.363	44	14	51	14	475	52.8	0.006283774
NP_002699.1	serine/threonine-protein phosphatase PP1-alpha catalytic subunit isoform 1	PPP1CA	5499	1.018	63	15	83	5	330	37.5	0.973101677
NP_004472.1	polypeptide N-acetylgalactosaminyltransferase 2 precursor	GALNT2	2590	1.492	37	20	59	20	571	64.7	0.125309258
NP_006657.1	ruvB-like 2 isoform 1	RUVBL2	10856	0.73	34	17	70	17	463	51.1	0.222513277
NP_066953.1	peptidyl-prolyl cis-trans isomerase A isoform 1	PPIA	5478	0.431	72	12	148	12	165	18	4.81E-05
NP_002061.1	guanine nucleotide-binding protein G(i) subunit alpha-2 isoform 1	GNAI2	2771	1.377	57	14	71	9	355	40.4	0.000124527
NP_001976.1	electron transfer flavoprotein subunit beta isoform 1	ETFB	2109	1.804	62	17	76	17	255	27.8	0.005113415
NP_001646.2	coatomer subunit delta isoform 1	ARCN1	372	0.654	32	16	63	16	511	57.2	0.025810783
NP_001092006.1	ubiquitin carboxyl-terminal hydrolase 5 isoform 1	USP5	8078	0.629	29	17	56	17	858	95.7	0.006959888
NP_037518.3	calcium-binding mitochondrial carrier protein SCaMC-1 isoform 1	SLC25A24	29957	1.708	49	22	61	22	477	53.3	0.000625388
NP_000662.3	alcohol dehydrogenase class-3	ADH5	128	0.439	37	15	60	15	374	39.7	0.019142364
XP_005251533.1	cytoplasmic aconitate hydratase isoform X1	ACO1	48	0.57	28	19	72	19	889	98.3	0.010736322
NP_004631.1	spliceosome RNA helicase DDX39B	DDX39B	7919	1.323	39	15	82	5	428	49	0.106335858
NP_005156.1	fructose-bisphosphate aldolase C	ALDOC	230	0.746	38	13	78	8	364	39.4	0.162563191
NP_005900.2	microtubule-associated protein 1B isoform 1	MAP1B	4131	0.651	11	19	47	18	2468	270.5	0.220913655
NP_863654.1	cytosolic acyl coenzyme A thioester hydrolase isoform hBACHb	ACOT7	11332	1.417	42	13	56	13	380	41.8	0.0271413
XP_011527866.1	lanosterol synthase isoform X1	LSS	4047	1.472	25	16	65	16	742	84.3	0.010263461
NP_000217.2	keratin, type I cytoskeletal 9	KRT9	3857	1.083	28	13	67	12	623	62	0.660590196
NP_001036143.1	rho-related GTP-binding protein RhoC precursor	RHOC	389	0.736	60	10	73	2	193	22	0.00347619
NP_001668.1	sodium/potassium-transporting ATPase subunit beta-1	ATP1B1	481	1.661	48	16	56	16	303	35	0.010818843
NP_001278798.1	insulin-like growth factor 2 mRNA-binding protein 2 isoform c	IGF2BP2	10644	1.102	30	14	76	12	605	66.7	0.56564156
XP_016883528.1	14-3-3 protein beta/alpha isoform X1	YWHAB	7529	0.611	52	13	150	5	246	28.1	0.031005898
NP_056289.2	deoxynucleoside triphosphate triphosphohydrolase SAMHD1 isoform 1	SAMHD1	25939	0.492	35	19	52	19	626	72.2	0.005099535
NP_001922.2	dihydrolipoyllysine-residue acetyltransferase component of pyruvate dehydrogenase complex, mitochondrial precursor	DLAT	1737	1.73	31	15	75	15	647	69	0.000806331
XP_005256612.1	protein flightless-1 homolog isoform X1	FLII	2314	0.581	22	22	54	22	1296	147.5	0.198132949
NP_002800.2	26S proteasome non-ATPase regulatory subunit 3	PSMD3	5709	0.984	34	16	70	16	534	60.9	0.684964278
NP_001128512.1	dynactin subunit 1 isoform 3	DCTN1	1639	0.581	23	22	51	21	1253	138.7	0.024931944
NP_000995.1	60S acidic ribosomal protein P2	RPLP2	6181	1.035	81	8	76	8	115	11.7	0.900915796
NP_002565.1	peroxiredoxin-1	PRDX1	5052	0.407	61	13	93	11	199	22.1	0.000972942
NP_001186913.1	NADH-ubiquinone oxidoreductase 75 kDa subunit, mitochondrial isoform 5	NDUFS1	4719	1.333	32	14	62	14	741	80.9	0.059314157
XP_005253946.1	2'-5'-oligoadenylate synthase 3 isoform X1	OAS3	4940	0.365	19	20	62	20	1089	121.4	0.000598702
NP_003671.1	tyrosine--tRNA ligase, cytoplasmic	YARS	8565	0.406	40	23	59	23	528	59.1	0.000728574
NP_001032248.1	acyl-coenzyme A thioesterase 9, mitochondrial isoform a precursor	ACOT9	23597	1.542	38	18	62	18	448	50.8	0.018439687
NP_004757.1	coatomer subunit beta'	COPB2	9276	0.627	28	19	60	19	906	102.4	5.14E-05
NP_001317629.1	DNA topoisomerase 2-beta isoform 1	TOP2B	7155	1.209	15	22	62	14	1626	183.2	0.725095884
XP_005258516.1	heterogeneous nuclear ribonucleoprotein U-like protein 1 isoform X1	HNRNPUL1	11100	1.265	25	17	52	17	866	96.8	0.148232297
NP_001484.1	rab GDP dissociation inhibitor alpha	GDI1	2664	0.538	44	15	58	7	447	50.6	0.000764499
NP_001307070.1	60S ribosomal protein L6 isoform 1	RPL6	6128	0.795	43	16	72	16	288	32.7	0.08524185
NP_001129125.1	polyadenylate-binding protein 4 isoform 1	PABPC4	8761	0.781	25	14	62	9	660	72.3	0.019448908
NP_001001.2	40S ribosomal protein S6	RPS6	6194	0.7	39	12	67	12	249	28.7	0.082027107
NP_006297.2	structural maintenance of chromosomes protein 1A isoform 1	SMC1A	8243	1.454	18	21	60	21	1233	143.1	0.010384349
XP_016870579.1	tropomyosin beta chain isoform X2	TPM2	7169	0.881	33	16	72	2	284	33	0.93066343
NP_848927.2	protein LYRIC isoform 1	MTDH	92140	1.438	36	16	50	16	582	63.8	0.086879198
NP_001539.3	interferon-induced protein with tetratricopeptide repeats 1 isoform 1	IFIT1	3434	0.263	37	14	53	13	478	55.3	4.05E-06
XP_011537116.1	early endosome antigen 1 isoform X1	EEA1	8411	1.603	19	24	46	24	1453	167.2	0.220669579
NP_036333.2	Golgi apparatus protein 1 isoform 1 precursor	GLG1	2734	1.398	18	20	56	20	1203	137.1	0.218592919
NP_001276033.1	glucosidase 2 subunit beta isoform 3 precursor	PRKCSH	5589	2.27	34	18	75	18	535	60.2	0.000202936
NP_001072992.1	multifunctional protein ADE2 isoform 2	PAICS	10606	0.568	40	18	65	18	425	47	0.018074289
XP_024305442.1	double-stranded RNA-specific adenosine deaminase isoform X1	ADAR	103	1.385	19	20	66	20	1269	140.7	2.38E-05
XP_011516104.1	aldehyde dehydrogenase X, mitochondrial isoform X1	ALDH1B1	219	1.495	36	18	72	14	517	57.2	0.001330117
NP_000370.2	xanthine dehydrogenase/oxidase	XDH	7498	0.488	16	16	46	15	1333	146.3	0.000208759
NP_002622.2	6-phosphogluconate dehydrogenase, decarboxylating isoform 1	PGD	5226	0.507	37	16	76	15	483	53.1	0.000728774
NP_005678.3	phenylalanine--tRNA ligase beta subunit	FARSB	10056	0.632	38	19	69	19	589	66.1	0.003125522
NP_005264.2	guanine nucleotide-binding protein G(I)/G(S)/G(T) subunit beta-2	GNB2	2783	1.408	41	10	59	5	340	37.3	0.016983894
NP_000975.2	60S ribosomal protein L23a	RPL23A	6147	0.779	46	13	70	13	156	17.7	0.031804782
NP_006182.2	proliferation-associated protein 2G4	PA2G4	5036	0.532	35	12	57	12	394	43.8	2.67E-05
NP_005313.1	histone H1.5	HIST1H1B	3009	2.518	36	14	72	10	226	22.6	3.86E-05
NP_004332.2	CAD protein isoform 1	CAD	790	0.626	9	17	49	16	2225	242.8	0.160625385
NP_001035809.1	ATP-binding cassette sub-family E member 1	ABCE1	6059	0.609	35	16	61	16	599	67.3	0.004665923
XP_011513391.1	insulin-like growth factor 2 mRNA-binding protein 3 isoform X1	IGF2BP3	10643	0.924	29	14	80	12	584	64.2	0.58424191
NP_002717.3	prolyl endopeptidase	PREP	5550	0.449	32	17	56	17	710	80.6	0.000341596
NP_058197.2	2'-5'-oligoadenylate synthase 2 isoform 1	OAS2	4939	0.368	26	17	57	17	719	82.4	0.000655836
NP_001129166.1	voltage-dependent anion-selective channel protein 3 isoform 2	VDAC3	7419	1.901	36	11	121	10	284	30.8	0.000780503
NP_000099.2	dihydrolipoyl dehydrogenase, mitochondrial isoform 1 precursor	DLD	1738	0.884	39	12	46	12	509	54.1	0.049576899
NP_057018.1	nucleolar protein 58	NOP58	51602	1.339	36	13	41	13	529	59.5	0.043736062
NP_001273196.1	heme oxygenase 2 isoform a	HMOX2	3163	1.182	43	11	46	11	370	41.6	0.191564393
NP_003650.1	alkyldihydroxyacetonephosphate synthase, peroxisomal precursor	AGPS	8540	0.887	32	14	44	14	658	72.9	0.408524372
XP_005260391.1	RNA-binding protein Raly isoform X1	RALY	22913	2.056	48	12	42	12	306	32.4	0.007881396
XP_005268061.1	inverted formin-2 isoform X1	INF2	64423	1.538	19	16	54	16	1281	139	8.81E-05
NP_002783.1	proteasome subunit alpha type-7	PSMA7	5688	0.732	48	11	55	11	248	27.9	0.006055396
NP_001351860.1	proteasome adapter and scaffold protein ECM29 isoform 4	ECPAS	23392	0.585	13	18	62	18	1845	204.2	2.91E-05
NP_078932.2	procollagen galactosyltransferase 1 precursor	COLGALT1	79709	1.847	28	17	78	17	622	71.6	0.003743003
NP_001675.3	plasma membrane calcium-transporting ATPase 4 isoform 4b	ATP2B4	493	1.777	19	16	53	12	1205	133.8	0.002402059
XP_024302561.1	serrate RNA effector molecule homolog isoform X1	SRRT	51593	1.51	21	16	55	16	906	104	0.005167043
NP_000997.1	40S ribosomal protein S3a isoform 1	RPS3A	6189	0.707	64	19	124	19	264	29.9	0.000289225
NP_001008938.1	cytoskeleton-associated protein 5 isoform a	CKAP5	9793	0.799	14	20	46	20	2032	225.4	0.03841478
NP_002943.2	40S ribosomal protein S2	RPS2	6187	0.642	45	12	94	12	293	31.3	0.000270357
NP_005130.1	annexin A3	ANXA3	306	0.41	45	16	66	16	323	36.4	0.000131889
NP_003357.2	cytochrome b-c1 complex subunit 2, mitochondrial precursor	UQCRC2	7385	1.58	31	11	56	11	453	48.4	0.003052423
NP_064601.3	aminopeptidase B isoform a	RNPEP	6051	0.492	35	17	59	17	650	72.5	9.40E-05
NP_710154.1	inhibitor of nuclear factor kappa-B kinase-interacting protein isoform 1	IKBIP	121457	1.974	45	19	65	15	377	43.1	0.042024661
NP_077310.1	transmembrane protein 43	TMEM43	79188	1.326	42	14	52	14	400	44.8	0.033825332
NP_150644.1	60S ribosomal protein L8	RPL8	6132	0.523	35	10	60	10	257	28	0.061384113
NP_001186040.1	malate dehydrogenase, cytoplasmic isoform 2	MDH1	4190	0.523	42	13	53	13	352	38.6	0.004495124
NP_004554.3	phosphoenolpyruvate carboxykinase	PCK2	5106	1.426	29	15	63	15	640	70.7	0.030740009
NP_001317273.1	tropomyosin alpha-1 chain isoform Tpm1.9cy	TPM1	7168	1.442	37	14	65	4	248	28.7	0.386062861
XP_011540693.1	far upstream element-binding protein 1 isoform X1	FUBP1	8880	1.182	30	18	50	15	688	72.1	0.349149319
NP_066997.3	cell cycle and apoptosis regulator protein 2 isoform 1	CCAR2	57805	1.603	23	14	57	14	923	102.8	0.009092094
NP_001616.1	adenylate kinase 2, mitochondrial isoform a	AK2	204	1.251	49	9	69	9	239	26.5	0.17138797
NP_005994.2	cytochrome b-c1 complex subunit Rieske, mitochondrial	UQCRFS1	7386	1.734	49	12	53	12	274	29.6	0.005576396
NP_000192.2	intercellular adhesion molecule 1 precursor	ICAM1	3383	1.881	28	13	59	13	532	57.8	0.002165098
NP_001340.2	aspartate--tRNA ligase, cytoplasmic isoform 1	DARS	1615	0.626	37	16	60	16	501	57.1	0.008850786
NP_006319.1	RNA-binding protein 14 isoform 1	RBM14	10432	1.482	31	16	53	16	669	69.4	0.104571703
NP_001070675.1	protein transport protein Sec31A isoform 1	SEC31A	22872	0.55	19	19	59	19	1220	132.9	0.004184843
NP_001309906.1	sideroflexin-1 isoform 1	SFXN1	94081	1.297	48	11	54	9	322	35.6	0.329588024
NP_001317558.1	calpastatin isoform s	CAST	831	0.373	33	14	34	1	763	82.2	0.641443066
NP_005737.1	nicotinamide phosphoribosyltransferase precursor	NAMPT	10135	0.516	35	12	51	12	491	55.5	0.003483062
NP_000010.1	acetyl-CoA acetyltransferase, mitochondrial precursor	ACAT1	38	1.729	37	13	53	13	427	45.2	0.008321633
NP_006347.1	ATP synthase subunit d, mitochondrial isoform a	ATP5PD	10476	2.008	86	12	77	12	161	18.5	0.004193047
NP_002780.1	proteasome subunit alpha type-4 isoform 1	PSMA4	5685	0.748	66	13	63	13	261	29.5	0.033851955
XP_011531424.1	macrophage-capping protein isoform X1	CAPG	822	0.586	45	11	40	11	348	38.5	0.001385383
NP_001748.1	carbonyl reductase	CBR1	873	0.603	63	14	56	11	277	30.4	0.01870633
NP_004152.1	ras-related protein Rab-1A isoform 1	RAB1A	5861	1.618	54	10	107	4	205	22.7	0.025628422
NP_001002.1	40S ribosomal protein S7	RPS7	6201	0.643	61	12	67	12	194	22.1	0.000470742
NP_005219.2	epidermal growth factor receptor isoform a precursor	EGFR	1956	1.166	18	16	38	16	1210	134.2	0.23154998
NP_005222.2	src substrate cortactin isoform a	CTTN	2017	0.9	30	15	52	15	550	61.5	0.635584598
NP_001317559.1	calpastatin isoform t	CAST	831	0.438	39	14	34	1	653	70.5	0.737142901
NP_002778.1	proteasome subunit alpha type-2	PSMA2	5683	0.899	46	7	57	7	234	25.9	0.618426689
XP_011539410.2	E3 ubiquitin-protein ligase UBR4 isoform X1	UBR4	23352	0.597	5	19	44	19	5272	582.9	0.121326618
XP_011512635.1	leukocyte elastase inhibitor isoform X1	SERPINB1	1992	0.492	40	13	49	12	379	42.7	0.001359129
NP_001410.2	ELAV-like protein 1	ELAVL1	1994	1.769	38	11	50	11	326	36.1	0.003164503
NP_001949.1	elongation factor 1-alpha 2	EEF1A2	1917	1.188	38	13	136	3	463	50.4	0.15197215
NP_001257376.1	very long-chain specific acyl-CoA dehydrogenase, mitochondrial isoform 3	ACADVL	37	0.604	33	18	51	18	678	72.9	0.012104464
NP_057123.1	RNA transcription, translation and transport factor protein	RTRAF	51637	0.827	56	12	50	12	244	28.1	0.086319062
NP_001688.1	ATP synthase subunit O, mitochondrial precursor	ATP5PO	539	1.716	57	10	66	10	213	23.3	0.002939943
NP_004550.2	nuclease-sensitive element-binding protein 1	YBX1	4904	0.598	51	10	47	8	324	35.9	0.022102679
NP_003133.1	lupus La protein	SSB	6741	1.011	38	17	82	17	408	46.8	0.942457148
NP_009210.1	splicing factor U2AF 65 kDa subunit isoform a	U2AF2	11338	1.128	28	7	48	7	475	53.5	0.273673052
NP_112243.1	ras-related protein Rab-1B	RAB1B	81876	1	66	10	107	4	201	22.2	0.801957724
NP_004199.1	apoptosis-inducing factor 1, mitochondrial isoform AIF precursor	AIFM1	9131	1.096	33	14	50	14	613	66.9	0.450595153
XP_016873669.1	FACT complex subunit SSRP1 isoform X2	SSRP1	6749	0.874	23	15	60	15	843	94.5	0.2286255
NP_002700.1	serine/threonine-protein phosphatase PP1-beta catalytic subunit isoform 1	PPP1CB	5500	0.73	46	12	75	3	327	37.2	0.087632174
NP_002818.1	tyrosine-protein phosphatase non-receptor type 1 isoform 1	PTPN1	5770	1.208	39	14	52	14	435	49.9	0.150379406
NP_002148.1	10 kDa heat shock protein, mitochondrial	HSPE1	3336	1.872	85	12	123	12	102	10.9	5.56E-05
NP_598000.2	CD109 antigen isoform 1 preproprotein	CD109	135228	1.689	15	16	66	16	1445	161.6	0.045737896
NP_004083.3	enoyl-CoA hydratase, mitochondrial	ECHS1	1892	1.567	41	10	53	10	290	31.4	0.000509851
XP_011517227.1	niban-like protein 1 isoform X2	FAM129B	64855	0.754	27	16	55	16	776	87.5	0.07828124
NP_001334786.1	erlin-1 isoform a	ERLIN1	10613	1.559	39	13	54	10	348	39.1	0.01394663
NP_001406.1	eukaryotic translation initiation factor 2 subunit 3	EIF2S3	1968	0.725	36	12	46	12	472	51.1	0.16219197
NP_149130.1	caldesmon isoform 4	CALD1	800	0.968	35	18	42	18	558	64.2	0.912004497
NP_055399.1	ERO1-like protein alpha precursor	ERO1A	30001	0.859	33	12	56	12	468	54.4	0.01285125
NP_683877.1	proteasome subunit alpha type-1 isoform 1	PSMA1	5682	0.736	54	14	63	14	269	30.2	0.002849215
NP_938405.1	ras GTPase-activating protein-binding protein 1	G3BP1	10146	0.748	29	12	52	12	466	52.1	0.021524835
NP_005042.1	glutamine--tRNA ligase isoform a	QARS	5859	0.802	29	19	50	19	775	87.7	0.341888393
XP_011511172.1	serine/threonine-protein kinase PAK 2 isoform X1	PAK2	5062	0.681	31	13	47	11	524	58	0.000636036
XP_011536806.1	serine/threonine-protein phosphatase PP1-gamma catalytic subunit isoform X1	PPP1CC	5501	1.321	38	11	71	1	362	41.4	0.394573298
XP_005247428.1	golgin subfamily B member 1 isoform X1	GOLGB1	2804	1.529	7	16	41	16	3269	376.9	0.19737408
NP_000968.2	60S ribosomal protein L13 isoform 1	RPL13	6137	0.795	54	14	67	14	211	24.2	0.383030006
NP_001123561.1	lysine--tRNA ligase isoform 1	KARS	3735	0.696	26	18	57	18	625	71.5	0.00064601
NP_056175.3	exosome RNA helicase MTR4	MTREX	23517	0.934	26	20	47	20	1042	117.7	0.681229003
NP_001596.2	alanine--tRNA ligase, cytoplasmic	AARS	16	0.447	21	16	53	16	968	106.7	0.003181132
NP_003655.3	AP-3 complex subunit beta-1 isoform 1	AP3B1	8546	0.629	22	20	65	20	1094	121.2	0.005334994
XP_024308688.1	septin-2 isoform X1	SEPT2	4735	0.744	35	12	49	12	428	49.4	0.230084492
NP_001075.1	procollagen-lysine,2-oxoglutarate 5-dioxygenase 3 precursor	PLOD3	8985	1.792	23	15	70	15	738	84.7	0.000389822
NP_001143.2	ADP/ATP translocase 2	SLC25A5	292	1.744	35	12	104	4	298	32.8	0.006434344
NP_006126.1	F-actin-capping protein subunit alpha-1	CAPZA1	829	0.929	51	10	50	7	286	32.9	0.67816439
NP_000512.1	beta-hexosaminidase subunit beta isoform 1 preproprotein	HEXB	3074	0.772	34	15	51	14	556	63.1	0.046498504
NP_005007.2	poly(rC)-binding protein 2 isoform a	PCBP2	5094	0.67	50	11	69	7	366	38.6	1.78E-05
NP_004068.2	citrate synthase, mitochondrial precursor	CS	1431	1.764	32	13	75	13	466	51.7	0.002373641
XP_006724323.1	eukaryotic translation initiation factor 3 subunit L isoform X1	EIF3L	51386	0.659	33	16	49	16	580	67.5	0.056382491
NP_001008.1	40S ribosomal protein S13	RPS13	6207	0.661	64	15	91	15	151	17.2	0.003204918
XP_024305108.1	high mobility group protein B1 isoform X1	HMGB1	3146	0.419	43	11	59	9	215	24.9	0.002670587
NP_001073027.1	heterogeneous nuclear ribonucleoprotein U-like protein 2	HNRNPUL2	221092	1.738	30	19	59	19	747	85.1	0.004912618
NP_115679.2	hydroxysteroid dehydrogenase-like protein 2 isoform 1	HSDL2	84263	1.728	38	14	56	14	418	45.4	0.021935554
NP_003339.1	ubiquitin-conjugating enzyme E2 N	UBE2N	7334	0.569	80	11	60	11	152	17.1	0.030513048
NP_001099008.1	myb-binding protein 1A isoform 1	MYBBP1A	10514	0.645	16	15	48	15	1332	149.3	0.156884852
NP_060225.4	tRNA (cytosine(34)-C(5))-methyltransferase isoform 1	NSUN2	54888	1.348	24	15	48	15	767	86.4	0.091559249
XP_024308602.1	annexin A4 isoform X1	ANXA4	307	0.488	55	17	66	16	321	36.1	0.002874135
NP_000687.3	4-trimethylaminobutyraldehyde dehydrogenase	ALDH9A1	223	0.801	29	13	52	13	518	56.3	0.219790725
XP_016882594.1	regulator of nonsense transcripts 1 isoform X1	UPF1	5976	0.747	19	15	47	15	1137	125.1	0.073386152
XP_016874610.1	coronin-1C isoform X2	CORO1C	23603	1.263	32	14	52	14	527	58.9	0.467447991
NP_001289958.1	plakophilin-3 isoform PKP3b	PKP3	11187	1.108	24	17	52	17	812	88.6	0.051466806
NP_000652.2	60S ribosomal protein L9	RPL9	6133	0.687	51	9	53	9	192	21.9	0.010837251
NP_001106963.1	septin-9 isoform a	SEPT9	10801	0.914	30	14	69	14	586	65.4	0.694616049
XP_005266896.1	beta/gamma crystallin domain-containing protein 1 isoform X1	CRYBG1	202	0.862	10	16	46	16	2131	231.6	0.411197174
NP_001229766.1	AP-2 complex subunit alpha-2 isoform 1	AP2A2	161	0.987	20	15	65	9	940	104	0.844444805
NP_079472.1	grpE protein homolog 1, mitochondrial precursor	GRPEL1	80273	2.185	48	11	50	11	217	24.3	4.08E-05
NP_055129.2	probable ATP-dependent RNA helicase DDX58	DDX58	23586	0.357	23	16	45	16	925	106.5	0.000806134
NP_002782.1	proteasome subunit alpha type-6 isoform a	PSMA6	5687	0.712	46	11	54	11	246	27.4	0.003782189
NP_001139748.1	neutral cholesterol ester hydrolase 1 isoform a	NCEH1	57552	1.828	32	10	44	10	448	49.9	0.008556784
XP_011532844.1	heterogeneous nuclear ribonucleoprotein H isoform X1	HNRNPH1	3187	1.157	36	11	77	6	472	51.2	0.271607291
NP_002146.2	heat shock 70 kDa protein 6	HSPA6	3310	0.84	15	11	66	1	643	71	0.84068574
NP_001303969.1	beta-galactosidase isoform d precursor	GLB1	2720	1.446	14	8	51	8	725	81.7	0.006978417
XP_024304570.1	chromodomain-helicase-DNA-binding protein 4 isoform X1	CHD4	1108	1.323	12	18	48	15	1941	220.8	0.345964595
NP_009123.1	FACT complex subunit SPT16	SUPT16H	11198	1.144	24	19	54	19	1047	119.8	0.546970754
NP_000681.2	aldehyde dehydrogenase, mitochondrial isoform 1 precursor	ALDH2	217	1.566	34	14	49	11	517	56.3	0.000367854
NP_001184223.1	dihydropyrimidinase-related protein 3 isoform 1	DPYSL3	1809	0.823	26	13	45	9	684	73.9	0.095570695
NP_003510.1	histone H2B type 1-L	HIST1H2BL	8340	2.044	62	9	171	3	126	13.9	0.004498024
XP_016868723.1	poly(U)-binding-splicing factor PUF60 isoform X5	PUF60	22827	1.313	23	11	50	11	596	64	0.072774384
XP_024306608.1	26S proteasome regulatory subunit 8 isoform X1	PSMC5	5705	0.896	34	12	59	11	433	48.3	0.354519804
XP_005267319.1	protein transport protein Sec23A isoform X1	SEC23A	10484	0.967	21	12	48	9	789	88.9	0.820839225
NP_005796.1	26S proteasome non-ATPase regulatory subunit 14	PSMD14	10213	0.935	43	10	63	10	310	34.6	0.816629143
NP_006833.2	splicing factor 3B subunit 2	SF3B2	10992	1.635	26	20	56	20	895	100.2	0.009790436
XP_016875261.1	GTP-binding nuclear protein Ran isoform X1	RAN	5901	0.591	42	11	57	11	222	25.3	0.0007611
NP_066952.1	inorganic pyrophosphatase	PPA1	5464	0.384	50	13	59	12	289	32.6	0.000325705
NP_066406.1	histone H2B type 1-B	HIST1H2BB	3018	1.479	62	9	162	3	126	13.9	0.005974239
XP_005269031.1	ATP-dependent 6-phosphofructokinase, muscle type isoform X1	PFKM	5213	0.585	17	12	47	11	883	96.8	0.010855899
NP_002005.1	peptidyl-prolyl cis-trans isomerase FKBP4	FKBP4	2288	0.552	39	14	49	14	459	51.8	0.014601863
XP_006714792.1	prolyl 4-hydroxylase subunit alpha-2 isoform X2	P4HA2	8974	1.477	33	14	48	14	533	60.6	0.001047256
XP_024307703.1	fermitin family homolog 1 isoform X1	FERMT1	55612	0.792	29	16	50	14	677	77.4	0.051499703
NP_001632.2	DNA-(apurinic or apyrimidinic site) lyase	APEX1	328	0.659	46	10	28	10	318	35.5	0.046898834
NP_690601.1	actin-related protein 2/3 complex subunit 2	ARPC2	10109	0.684	46	13	44	13	300	34.3	0.000547878
XP_024308119.1	long-chain-fatty-acid--CoA ligase 4 isoform X2	ACSL4	2182	1.126	22	13	43	10	717	79.8	0.65998883
XP_006711560.1	60 kDa SS-A/Ro ribonucleoprotein isoform X2	TROVE2	6738	0.822	26	13	51	13	538	60.6	0.161190849
NP_001001973.1	ATP synthase subunit gamma, mitochondrial isoform L (liver) precursor	ATP5F1C	509	0.845	32	11	67	1	298	33	0.678570713
XP_005268559.1	treacle protein isoform X1	TCOF1	6949	1.105	12	14	43	14	1526	156.1	0.216015472
NP_002387.1	NAD-dependent malic enzyme, mitochondrial isoform 1 precursor	ME2	4200	1.297	27	10	40	10	584	65.4	0.027538899
NP_004453.3	squalene synthase isoform 1	FDFT1	2222	1.457	34	12	74	12	417	48.1	0.051280678
NP_006342.2	mitochondrial import inner membrane translocase subunit TIM44	TIMM44	10469	1.463	32	14	40	14	452	51.3	0.005214091
XP_024309900.1	probable ATP-dependent RNA helicase DDX60 isoform X1	DDX60	55601	0.599	15	17	47	17	1712	197.7	0.099010419
NP_001035930.1	prosaposin isoform b preproprotein	PSAP	5660	2.32	20	11	68	11	527	58.4	6.66E-05
NP_000960.2	60S ribosomal protein L5	RPL5	6125	0.832	43	13	56	13	297	34.3	0.259350739
NP_060024.2	poly	PARP14	54625	0.534	13	17	44	17	1801	202.7	0.12260845
NP_005165.1	ATP synthase subunit gamma, mitochondrial isoform H (heart) precursor	ATP5F1C	509	1.571	32	11	58	1	297	32.9	0.110692428
XP_011518397.1	rho GTPase-activating protein 1 isoform X2	ARHGAP1	392	0.718	31	9	38	9	439	50.4	0.038395595
NP_004993.1	NADH dehydrogenase	NDUFA9	4704	1.21	41	12	46	12	377	42.5	0.111378044
XP_024307972.1	SUN domain-containing protein 2 isoform X4	SUN2	25777	1.049	23	11	35	11	752	83.9	0.937131414
NP_995314.1	nck-associated protein 1 isoform 2	NCKAP1	10787	0.822	17	14	35	14	1134	129.4	0.643382348
NP_002284.3	laminin subunit gamma-1 precursor	LAMC1	3915	1.159	12	14	41	14	1609	177.5	0.304022294
NP_000886.1	leukotriene A-4 hydrolase isoform 1	LTA4H	4048	0.557	29	16	48	16	611	69.2	0.014033993
NP_005038.1	26S proteasome non-ATPase regulatory subunit 5 isoform 1	PSMD5	5711	0.644	32	13	49	13	504	56.2	0.022964847

Patil et al,2020. Proteomic and phosphoproteomic profiling of shammah-induced signaling in oral keratinocytes

Supplementary Table S1: List of proteins identified and quantified in OKF6/TERT1-Shammah and OKF6/TERT1-Parental total proteome using TMT-based quantitative proteomic approach

Accession	Protein Description	Gene Symbol	Gene ID	Fold Change	Coverage [%]	# Peptides	# PSMs	# Unique Peptides	# AAs	MW [kDa]	p-value
NP_006187.2	poly(rC)-binding protein 1	PCBP1	5093	0.797	58	12	57	8	356	37.5	0.251700441
NP_002261.3	transportin-1 isoform 1	TNPO1	3842	0.538	21	15	51	11	898	102.3	0.002636462
NP_001350.1	2,4-dienoyl-CoA reductase, mitochondrial isoform 1 precursor	DECR1	1666	1.363	38	9	39	9	335	36	0.132534776
NP_001019636.1	superoxide dismutase	SOD2	6648	1.028	43	11	57	11	222	24.7	0.976672656
NP_000916.2	pyruvate dehydrogenase E1 component subunit beta, mitochondrial isoform 1 precursor	PDHB	5162	1.62	42	12	59	12	359	39.2	0.000185804
NP_006001.4	mesencephalic astrocyte-derived neurotrophic factor precursor	MANF	7873	1.98	46	10	50	10	185	21.1	0.022655408
NP_110437.2	thioredoxin domain-containing protein 5 isoform 1 precursor	TXNDC5	81567	1.768	32	13	47	13	432	47.6	0.00012967
NP_057190.2	sec1 family domain-containing protein 1 isoform a	SCFD1	23256	1.124	26	12	42	12	642	72.3	0.585899781
NP_004085.1	eukaryotic translation initiation factor 2 subunit 1	EIF2S1	1965	0.701	52	15	52	15	315	36.1	0.002661498
NP_001269581.1	stress-induced-phosphoprotein 1 isoform a	STIP1	10963	0.578	27	16	49	16	590	68	0.000588751
NP_056274.3	atlastin-3 isoform 1	ATL3	25923	1.589	32	13	40	13	541	60.5	0.081786053
NP_005436.1	structural maintenance of chromosomes protein 3	SMC3	9126	1.391	21	21	51	21	1217	141.5	0.015404507
NP_055975.1	mitochondrial-processing peptidase subunit alpha isoform 1 precursor	PMPCA	23203	1.484	29	12	46	12	525	58.2	0.004354189
NP_001627.2	ADP/ATP translocase 3	SLC25A6	293	1.935	44	12	87	1	298	32.8	0.008374719
NP_001137232.1	eukaryotic translation initiation factor 5A-1 isoform A	EIF5A	1984	0.254	59	11	76	11	184	20.2	0.000154168
NP_002856.1	ras-related protein Rab-2A isoform a	RAB2A	5862	1.417	46	9	47	9	212	23.5	0.0072299
NP_963906.1	inhibitor of nuclear factor kappa-B kinase-interacting protein isoform 2	IKBIP	121457	1.783	37	15	58	11	350	39.3	0.00263255
NP_066565.1	glycylpeptide N-tetradecanoyltransferase 1	NMT1	4836	0.571	30	10	32	10	496	56.8	0.00078455
NP_874365.3	protein scribble homolog isoform a	SCRIB	23513	1.579	9	10	33	9	1655	177.6	0.02104586
NP_001276978.1	general vesicular transport factor p115 isoform 1	USO1	8615	0.869	18	14	42	14	973	109.1	0.480868315
XP_005252029.1	tenascin isoform X5	TNC	3371	1.762	10	14	37	14	2110	230.7	0.00070367
NP_065392.1	adipocyte plasma membrane-associated protein	APMAP	57136	2.449	37	12	55	12	416	46.5	5.42E-05
NP_057406.2	ras-related protein Rab-14	RAB14	51552	1.243	65	13	64	12	215	23.9	0.154376339
XP_024305465.1	28S ribosomal protein S29, mitochondrial isoform X1	DAP3	7818	0.988	29	9	39	9	398	45.5	0.874044363
XP_005261752.1	ran GTPase-activating protein 1 isoform X1	RANGAP1	5905	1.002	29	16	45	16	642	69.5	0.825298761
NP_002583.1	proliferating cell nuclear antigen	PCNA	5111	0.45	41	9	44	9	261	28.8	0.002154359
XP_005273449.1	erlin-2 isoform X1	ERLIN2	11160	2.019	38	13	50	10	339	37.8	0.000847064
NP_001354.1	H/ACA ribonucleoprotein complex subunit DKC1 isoform 1	DKC1	1736	1.631	21	11	38	11	514	57.6	0.009704743
NP_002797.3	26S proteasome regulatory subunit 10B	PSMC6	5706	0.912	31	11	45	11	403	45.8	0.687838643
XP_011536695.1	nucleosome assembly protein 1-like 1 isoform X1	NAP1L1	4673	0.409	26	10	46	8	391	45.3	0.00117194
NP_055866.1	endoplasmic reticulum resident protein 44 precursor	ERP44	23071	1.579	31	12	52	11	406	46.9	0.03021151
NP_002809.2	proteasome activator complex subunit 2	PSME2	5721	0.434	46	7	49	7	239	27.4	0.000784602
NP_112233.2	sideroflexin-3	SFXN3	81855	1.431	39	10	44	8	325	36	0.129598968
XP_011516714.1	constitutive coactivator of PPAR-gamma-like protein 1 isoform X1	FAM120A	23196	0.974	17	14	38	14	1146	125.2	0.48765398
NP_006175.2	nucleobindin-1 precursor	NUCB1	4924	1.651	39	14	48	13	461	53.8	0.001456045
NP_001005360.1	dynamin-2 isoform 1	DNM2	1785	0.812	22	15	46	12	870	98	0.108436148
NP_150241.2	protein PML isoform 1	PML	5371	1.069	21	15	49	15	882	97.5	0.218734958
NP_061119.1	core histone macro-H2A.2	H2AFY2	55506	1.71	29	9	45	7	372	40	0.051881893
XP_005254127.1	tripeptidyl-peptidase 2 isoform X1	TPP2	7174	1.026	17	18	40	18	1304	144.4	0.944116042
NP_005795.2	ATP-dependent RNA helicase DDX39A	DDX39A	10212	0.738	31	12	60	2	427	49.1	0.341852157
NP_001290553.1	60S ribosomal protein L10 isoform a	RPL10	6134	0.526	39	8	61	8	214	24.6	0.00077386
NP_055227.1	calcyclin-binding protein isoform 1	CACYBP	27101	0.433	60	14	42	14	228	26.2	1.06E-05
NP_001193997.1	basic leucine zipper and W2 domain-containing protein 1 isoform 2	BZW1	9689	0.573	35	14	44	13	451	51.2	0.002814826
NP_001142.2	ADP/ATP translocase 1	SLC25A4	291	1.316	42	11	69	2	298	33	0.32813793
NP_037473.3	obg-like ATPase 1 isoform 1	OLA1	29789	0.519	43	13	56	13	396	44.7	0.001816959
NP_006550.1	KH domain-containing, RNA-binding, signal transduction-associated protein 1 isoform 1	KHDRBS1	10657	1.621	26	9	44	9	443	48.2	0.057650469
NP_001010942.1	ras-related protein Rap-1b isoform 1 precursor	RAP1B	5908	1.463	42	10	48	3	184	20.8	0.246089356
XP_006718761.1	calpain-1 catalytic subunit isoform X1	CAPN1	823	0.575	28	17	46	17	714	81.8	0.000577498
NP_004270.2	mitochondrial-processing peptidase subunit beta precursor	PMPCB	9512	1.587	27	12	41	12	489	54.3	4.82E-06
NP_001350617.1	NPC intracellular cholesterol transporter 2 isoform 1 precursor	NPC2	10577	1.463	45	7	50	7	174	19.2	0.065750826
NP_006136.1	dnaJ homolog subfamily B member 1 isoform 1	DNAJB1	3337	0.569	40	13	49	12	340	38	0.0006906
NP_001238978.1	protein-L-isoaspartate(D-aspartate) O-methyltransferase isoform 2	PCMT1	5110	0.752	47	9	43	9	286	30.3	0.067807637
NP_055792.1	apoptotic chromatin condensation inducer in the nucleus isoform 1	ACIN1	22985	1.485	14	15	44	15	1341	151.8	0.063464981
NP_055950.1	nuclear pore complex protein Nup205 isoform 1	NUP205	23165	1.073	11	17	48	17	2012	227.8	0.258657495
XP_016877512.1	cytoplasmic FMR1-interacting protein 1 isoform X1	CYFIP1	23191	0.691	11	15	44	15	1420	163.1	0.009465885
NP_001269091.1	F-actin-capping protein subunit beta isoform 4	CAPZB	832	0.914	44	11	48	11	301	33.8	0.283767477
XP_005261192.1	ATP-dependent 6-phosphofructokinase, liver type isoform X3	PFKL	5211	0.645	18	12	42	9	803	87.6	0.139003262
XP_016870502.1	protein SET isoform X1	SET	6418	0.704	43	10	43	10	309	35.8	0.060417373
XP_011509279.1	glycerol-3-phosphate dehydrogenase, mitochondrial isoform X1	GPD2	2820	1.019	29	16	44	16	727	80.8	0.526809585
NP_001004.2	40S ribosomal protein S9 isoform a	RPS9	6203	0.742	55	17	84	17	194	22.6	0.024193623
NP_006138.1	interferon regulatory factor 6 isoform 1	IRF6	3664	0.616	33	10	48	10	467	53.1	0.156490572
NP_072045.1	40S ribosomal protein S18	RPS18	6222	0.641	49	12	64	12	152	17.7	0.002690081
NP_002898.2	ATP-dependent DNA helicase Q1	RECQL	5965	1.006	21	14	41	14	649	73.4	0.947309134
XP_006718788.1	182 kDa tankyrase-1-binding protein isoform X1	TNKS1BP1	85456	0.746	12	13	35	13	1729	181.7	0.109079495
NP_476516.1	N-alpha-acetyltransferase 15, NatA auxiliary subunit	NAA15	80155	0.547	23	19	47	16	866	101.2	0.039855367
NP_001924.2	dihydrolipoyllysine-residue succinyltransferase component of 2-oxoglutarate dehydrogenase complex, mitochondrial isoform 1 precursor	DLST	1743	1.948	21	8	47	8	453	48.7	0.002382091
NP_003130.2	signal recognition particle receptor subunit alpha isoform 1	SRPRA	6734	1.603	26	14	44	14	638	69.8	0.035279102
NP_001598.1	3-ketoacyl-CoA thiolase, peroxisomal isoform a	ACAA1	30	1.388	37	10	39	10	424	44.3	0.007069182
XP_005266431.1	plastin-2 isoform X1	LCP1	3936	0.689	28	13	46	8	627	70.2	0.116937503
XP_024306610.1	26S proteasome non-ATPase regulatory subunit 12 isoform X1	PSMD12	5718	0.653	35	15	49	15	485	56	0.068041134
NP_001093896.1	rap1 GTPase-GDP dissociation stimulator 1 isoform 1	RAP1GDS1	5910	0.429	26	12	48	12	608	66.3	0.003632234
NP_002070.1	aspartate aminotransferase, cytoplasmic	GOT1	2805	0.471	37	11	37	11	413	46.2	0.000109801
NP_001287789.1	probable ATP-dependent RNA helicase DDX46 isoform 1	DDX46	9879	1.265	20	20	44	20	1032	117.4	0.009782553
XP_011513901.1	eukaryotic translation initiation factor 3 subunit B isoform X1	EIF3B	8662	0.672	23	15	39	15	814	92.4	0.114895608
NP_006835.2	acetolactate synthase-like protein	ILVBL	10994	1.183	28	9	33	9	632	67.8	0.074502874
NP_006397.1	peroxiredoxin-4 precursor	PRDX4	10549	1.473	40	8	47	7	271	30.5	0.049872262
NP_001186071.1	eukaryotic translation initiation factor 3 subunit C isoform a	EIF3C	8663	0.808	22	16	45	16	913	105.3	0.1032098
NP_078856.4	elongation factor-like GTPase 1 isoform 1	EFL1	79631	0.623	21	14	40	14	1120	125.4	0.334906746
NP_001351796.1	gamma-interferon-inducible protein 16 isoform 3	IFI16	3428	0.956	21	15	50	15	785	88.2	0.355557473
XP_024302613.1	extended synaptotagmin-2 isoform X1	ESYT2	57488	1.562	16	12	40	12	914	101.2	0.06956321
NP_077305.2	EF-hand domain-containing protein D2	EFHD2	79180	0.868	43	13	44	13	240	26.7	0.037275106
NP_001530.1	dnaJ homolog subfamily A member 1 isoform 1	DNAJA1	3301	0.718	36	10	34	9	397	44.8	0.152619786
NP_006699.2	lactoylglutathione lyase	GLO1	2739	0.399	43	10	49	10	184	20.8	0.002358981
NP_004823.1	glutathione S-transferase omega-1 isoform 1	GSTO1	9446	0.293	54	11	48	11	241	27.5	0.00025094
XP_005245932.1	heterochromatin protein 1-binding protein 3 isoform X1	HP1BP3	50809	1.739	24	16	55	16	553	61.2	0.000587025
NP_001171634.2	hydroxyacyl-coenzyme A dehydrogenase, mitochondrial isoform 1 precursor	HADH	3033	1.822	36	13	61	13	331	36	0.00876239
NP_060697.4	ubiquitin-like modifier-activating enzyme 6	UBA6	55236	0.608	17	14	39	14	1052	117.9	0.00012576
NP_056417.2	torsin-1A-interacting protein 1 isoform 2	TOR1AIP1	26092	1.626	21	11	43	10	583	66.2	0.012890187
NP_001243441.1	single-stranded DNA-binding protein, mitochondrial precursor	SSBP1	6742	1.667	59	9	40	9	148	17.2	0.013276216
NP_001180236.1	sterile alpha motif domain-containing protein 9	SAMD9	54809	0.372	11	12	39	11	1589	184.2	0.000722601
NP_002130.2	RNA-binding motif protein, X chromosome isoform 1	RBMX	27316	1.563	25	9	60	6	391	42.3	0.036145125
NP_057376.2	heat shock protein 75 kDa, mitochondrial isoform 1 precursor	TRAP1	10131	1.659	29	17	70	16	704	80.1	0.049171209
NP_006750.3	UTP--glucose-1-phosphate uridylyltransferase isoform a	UGP2	7360	0.63	35	15	51	15	508	56.9	0.01019142
NP_055428.1	FAS-associated factor 2	FAF2	23197	1.16	41	12	37	12	445	52.6	0.172849341
NP_001136402.1	apoptosis inhibitor 5 isoform a	API5	8539	0.928	27	13	42	13	524	59	0.768663561
NP_940890.3	protein FAM83H	FAM83H	286077	1.141	17	13	35	13	1179	127.1	0.536840326
NP_004883.3	vesicle-trafficking protein SEC22b precursor	SEC22B	9554	1.484	44	7	41	7	215	24.7	0.001501419
XP_006715869.1	septin-7 isoform X2	SEPT7	989	0.854	29	11	46	10	448	52	0.42411679
NP_001966.1	gamma-enolase	ENO2	2026	1.164	32	9	223	6	434	47.2	0.487699188
NP_005558.1	galectin-3-binding protein precursor	LGALS3BP	3959	1.142	22	10	44	10	585	65.3	0.091166646
NP_005887.2	isocitrate dehydrogenase	IDH1	3417	0.394	41	15	55	14	414	46.6	0.000848092
NP_055635.3	mitochondrial import receptor subunit TOM70	TOMM70	9868	1.812	24	13	47	13	608	67.4	0.002457326
NP_001018077.1	plasminogen activator inhibitor 1 RNA-binding protein isoform 1	SERBP1	26135	0.421	32	12	64	12	408	44.9	0.001028634
NP_055317.1	pre-mRNA-processing factor 19	PRPF19	27339	1.243	31	10	51	10	504	55.1	0.226526705
NP_001559.1	eukaryotic translation initiation factor 3 subunit E	EIF3E	3646	0.609	37	14	44	14	445	52.2	0.016985955
NP_005975.1	tricarboxylate transport protein, mitochondrial isoform a precursor	SLC25A1	6576	1.841	34	9	43	9	311	34	0.000114707
NP_001289978.1	myosin regulatory light chain 12A isoform 2	MYL12A	10627	0.45	55	8	45	8	177	20.4	0.004230059
NP_002970.2	non-specific lipid-transfer protein isoform 1 precursor	SCP2	6342	2.593	19	13	51	13	547	59	0.009271019
XP_016875215.1	paxillin isoform X1	PXN	5829	0.732	13	8	29	8	1135	121.8	0.14205097
NP_036387.2	5'-3' exoribonuclease 2 isoform 2	XRN2	22803	1.188	15	13	39	13	950	108.5	0.06421053
NP_478059.1	phosphoserine aminotransferase isoform 1	PSAT1	29968	0.387	40	12	41	12	370	40.4	0.000403008
NP_001290430.1	calponin-2 isoform d	CNN2	1265	0.547	32	9	38	8	330	35.9	0.001048866
NP_001308413.1	40S ribosomal protein S19 isoform 1	RPS19	6223	0.741	50	12	56	12	145	16.1	0.007053552
NP_001189342.1	alcohol dehydrogenase	AKR1A1	10327	0.6	38	11	42	10	325	36.6	0.000569974
NP_001166925.1	pyruvate dehydrogenase E1 component subunit alpha, somatic form, mitochondrial isoform 2 precursor	PDHA1	5160	1.676	42	14	42	14	428	47.5	0.002675608
XP_016866714.1	dystonin isoform X33	DST	667	0.955	8	17	30	10	3195	370	0.86089115
XP_011512318.2	ankycorbin isoform X1	RAI14	26064	1.07	17	14	32	14	986	110.4	0.767089513
NP_001003.1	40S ribosomal protein S8	RPS8	6202	0.719	52	10	64	10	208	24.2	0.102548802
NP_006391.1	dynactin subunit 2 isoform 1	DCTN2	10540	0.689	37	11	42	11	406	44.8	0.012000277
NP_055121.1	tRNA-splicing ligase RtcB homolog	RTCB	51493	0.976	36	12	41	12	505	55.2	0.784518497
NP_004530.1	asparagine--tRNA ligase, cytoplasmic	NARS	4677	0.579	33	14	37	14	548	62.9	0.007381538
NP_112740.1	heterogeneous nuclear ribonucleoprotein D-like isoform a	HNRNPDL	9987	1.433	26	10	54	8	420	46.4	0.004263976
NP_937818.1	nucleoside diphosphate kinase A isoform a	NME1	4830	0.879	50	9	63	1	177	19.6	0.990096042
NP_003925.1	far upstream element-binding protein 3	FUBP3	8939	1.282	36	15	42	13	572	61.6	2.95E-05
XP_024303470.1	histidine triad nucleotide-binding protein 2, mitochondrial isoform X1	HINT2	84681	1.581	41	4	22	4	165	17.8	0.008609312
NP_000894.1	NAD(P)H dehydrogenase	NQO1	1728	0.255	39	11	44	11	274	30.8	0.000669162
XP_005266964.1	monofunctional C1-tetrahydrofolate synthase, mitochondrial isoform X1	MTHFD1L	25902	1.386	17	14	41	14	986	106.6	0.102208743
NP_009034.2	NADH dehydrogenase	NDUFV1	4723	1.358	36	11	36	11	464	50.8	0.240004564
NP_006808.1	endoplasmic reticulum resident protein 29 isoform 1 precursor	ERP29	10961	1.854	50	9	46	9	261	29	5.17E-05
NP_112556.2	heterogeneous nuclear ribonucleoprotein A/B isoform a	HNRNPAB	3182	0.829	27	8	51	7	332	35.9	0.14177788
NP_149100.2	polyribonucleotide nucleotidyltransferase 1, mitochondrial precursor	PNPT1	87178	1.307	23	16	51	16	783	85.9	0.124683551
NP_009005.1	coronin-1A	CORO1A	11151	0.723	27	10	38	10	461	51	0.244997503
NP_002786.2	proteasome subunit beta type-3	PSMB3	5691	0.645	41	7	40	7	205	22.9	0.060121725
NP_003899.2	eukaryotic translation initiation factor 2 subunit 2 isoform 1	EIF2S2	8894	0.796	47	14	34	14	333	38.4	0.307309329
XP_024304757.1	plasma membrane calcium-transporting ATPase 1 isoform X1	ATP2B1	490	1.786	10	10	31	6	1249	137.7	0.044584606
NP_001351228.1	succinyl-CoA:3-ketoacid coenzyme A transferase 1, mitochondrial isoform 1 precursor	OXCT1	5019	1.579	29	12	43	12	527	57.1	0.026030423
NP_057001.1	glutathione S-transferase kappa 1 isoform a	GSTK1	373156	1.251	52	9	41	9	226	25.5	0.075594957
NP_001950.1	elongation factor 1-beta	EEF1B2	1933	0.767	52	11	51	9	225	24.7	0.248813101
NP_005207.2	dolichyl-diphosphooligosaccharide--protein glycosyltransferase 48 kDa subunit precursor	DDOST	1650	1.551	28	11	61	11	456	50.7	0.027742657
NP_000572.2	glutathione peroxidase 1 isoform 1	GPX1	2876	1.207	77	11	50	11	203	22.1	0.003823104
NP_001619.1	aldose reductase isoform 1	AKR1B1	231	0.584	48	11	33	10	316	35.8	0.055126188
NP_003565.4	vesicle-associated membrane protein-associated protein A isoform 1	VAPA	9218	1.839	26	7	53	6	294	32.6	0.00173635
NP_005871.1	dnaJ homolog subfamily A member 2	DNAJA2	10294	0.846	30	9	28	9	412	45.7	0.620259928
NP_001317598.1	serine--tRNA ligase, cytoplasmic isoform b	SARS	6301	0.358	28	11	29	11	536	61.3	0.000430398
XP_024303687.1	RRP12-like protein isoform X1	RRP12	23223	1.024	11	10	31	10	1357	149.5	0.990182981
NP_001073272.1	lysosomal alpha-glucosidase preproprotein	GAA	2548	2.22	11	8	28	8	952	105.3	0.00454504
NP_001305051.1	adenylate kinase isoenzyme 1 isoform 2	AK1	203	0.605	46	10	48	10	210	23.4	0.141651708
NP_000365.3	uroporphyrinogen decarboxylase	UROD	7389	0.525	32	7	26	7	367	40.8	0.002447985
XP_005273057.1	3'(2'),5'-bisphosphate nucleotidase 1 isoform X2	BPNT1	10380	0.89	39	10	27	10	308	33.4	0.57116063
XP_016876283.1	serine/threonine-protein kinase 24 isoform X1	STK24	8428	0.598	29	14	45	6	517	56.9	0.107934885
NP_620305.2	protein SON isoform F	SON	6651	1.358	8	12	30	12	2426	263.7	0.093221203
NP_006261.1	ras-related protein R-Ras	RRAS	6237	1.301	35	7	41	5	218	23.5	0.023247939
NP_001540.2	interferon-induced protein with tetratricopeptide repeats 3 isoform a	IFIT3	3437	0.404	28	9	31	9	490	56	0.002732888
NP_004791.1	transmembrane 9 superfamily member 2 precursor	TM9SF2	9375	1.192	20	10	29	10	663	75.7	0.040629011
NP_060542.4	HEAT repeat-containing protein 1	HEATR1	55127	1.223	8	14	40	14	2144	242.2	0.196235673
NP_644670.1	EH domain-containing protein 4	EHD4	30844	0.618	33	14	40	9	541	61.1	0.022009739
NP_005727.1	alpha-centractin	ACTR1A	10121	0.587	41	10	39	9	376	42.6	0.002926613
XP_024308303.1	LOW QUALITY PROTEIN: HLA class I histocompatibility antigen, A-1 alpha chain isoform X1	HLA-A	3105	0.872	42	13	37	7	426	47.8	0.498494876
NP_001007.2	40S ribosomal protein S12	RPS12	6206	0.756	45	8	45	8	132	14.5	0.025277828
NP_006382.1	importin-7	IPO7	10527	0.581	15	11	37	11	1038	119.4	0.001407002
NP_004175.2	tryptophan--tRNA ligase, cytoplasmic isoform a	WARS	7453	0.383	22	9	38	9	471	53.1	0.001079685
NP_002784.1	proteasome subunit beta type-1	PSMB1	5689	0.881	44	8	35	8	241	26.5	0.453828178
NP_004090.4	erythrocyte band 7 integral membrane protein isoform a	STOM	2040	1.47	37	7	42	7	288	31.7	0.000356295
NP_001258963.1	syntaxin-binding protein 2 isoform c	STXBP2	6813	0.901	23	12	34	12	604	67.7	0.371538764
NP_002624.2	phosphoglucomutase-1 isoform 1	PGM1	5236	0.619	24	11	36	11	562	61.4	0.015321603
NP_001091675.1	heterogeneous nuclear ribonucleoprotein F	HNRNPF	3185	1.206	32	9	72	7	415	45.6	0.487029781
XP_016880042.1	acetyl-CoA carboxylase 1 isoform X1	ACACA	31	0.683	9	16	36	16	2391	270.5	0.063150502
NP_705618.1	nuclear pore complex protein Nup155 isoform 1	NUP155	9631	1.026	11	12	37	12	1391	155.1	0.658553071
NP_001258708.1	26S proteasome non-ATPase regulatory subunit 6 isoform 1	PSMD6	9861	0.805	36	14	49	14	442	51.9	0.019144849
NP_006787.2	AFG3-like protein 2	AFG3L2	10939	1.48	24	19	42	19	797	88.5	0.056755882
NP_003678.2	PDZ and LIM domain protein 4 isoform 1	PDLIM4	8572	0.891	49	10	32	10	330	35.4	0.350305066
NP_003081.2	U2 small nuclear ribonucleoprotein A'	SNRPA1	6627	1.155	44	10	39	10	255	28.4	0.000185527
NP_002781.2	proteasome subunit alpha type-5 isoform 1	PSMA5	5686	0.734	44	7	40	7	241	26.4	0.039541852
NP_005490.1	SUMO-activating enzyme subunit 2	UBA2	10054	0.923	24	12	37	12	640	71.2	0.547482263
NP_001288170.1	rho GDP-dissociation inhibitor 1 isoform c	ARHGDIA	396	0.512	46	9	45	9	235	25.8	0.01063394
NP_001132929.1	B-cell receptor-associated protein 31 isoform a	BCAP31	10134	1.501	35	15	56	15	313	34.7	0.031427385
NP_036339.1	heterogeneous nuclear ribonucleoprotein H3 isoform a	HNRNPH3	3189	1.649	34	8	36	7	346	36.9	0.000145927
XP_011543800.1	SH3 domain-containing kinase-binding protein 1 isoform X1	SH3KBP1	30011	0.527	16	10	32	10	709	77.9	0.000597247
XP_024304469.1	cysteine--tRNA ligase, cytoplasmic isoform X1	CARS	833	0.612	20	16	45	16	867	98.4	0.000392646
NP_006784.1	thioredoxin-dependent peroxide reductase, mitochondrial isoform a precursor	PRDX3	10935	2.048	34	6	44	6	256	27.7	0.008758069
NP_004541.1	NADH dehydrogenase	NDUFS2	4720	1.527	33	11	36	11	463	52.5	0.104095011
NP_981946.1	stathmin isoform a	STMN1	3925	0.502	56	10	42	10	149	17.3	0.019637085
NP_004951.1	RNA-binding protein FUS isoform 1	FUS	2521	1.406	14	9	54	7	526	53.4	0.01007712
XP_024304740.1	ADP-ribosylation factor 3 isoform X1	ARF3	377	0.646	44	6	76	3	181	20.6	0.139086813
NP_009109.3	serine-threonine kinase receptor-associated protein	STRAP	11171	0.566	30	8	24	8	350	38.4	0.016575729
NP_057134.1	39S ribosomal protein L11, mitochondrial isoform a	MRPL11	65003	1.755	55	10	30	10	192	20.7	0.004277229
NP_001058.2	DNA topoisomerase 2-alpha	TOP2A	7153	1.153	8	14	42	6	1531	174.3	0.27023522
NP_001186600.1	calumenin isoform c precursor	CALU	813	2.512	39	12	41	12	323	38	0.000148839
NP_001035807.1	histone H2A type 2-A	HIST2H2AA4	723790	4.586	36	8	238	2	130	14.1	0.000280401
NP_057215.3	ras-related protein Rab-10	RAB10	10890	1.372	58	13	80	10	200	22.5	0.129837015
NP_955392.1	tumor protein D54 isoform a	TPD52L2	7165	0.971	44	11	35	11	229	24.8	0.814179555
NP_003818.2	alpha-soluble NSF attachment protein	NAPA	8775	0.877	43	11	34	11	295	33.2	0.442274521
NP_001186284.1	RPL17-C18orf32 protein isoform 1	RPL17-C18orf32	100526842	0.493	23	6	69	6	228	26.4	0.000951163
NP_005713.1	actin-related protein 2 isoform b	ACTR2	10097	0.707	28	9	52	8	394	44.7	0.051616638
NP_001308948.1	NADH dehydrogenase	NDUFA10	4705	1.803	26	10	36	10	375	42.8	0.038192067
NP_001273677.1	28S ribosomal protein S27, mitochondrial isoform 1	MRPS27	23107	1.278	28	9	32	9	428	49.1	0.080066701
NP_005110.2	thyroid hormone receptor-associated protein 3 isoform 1	THRAP3	9967	1.219	15	15	39	15	955	108.6	0.243271781
NP_001271317.1	nucleolar and coiled-body phosphoprotein 1 isoform 1	NOLC1	9221	0.61	19	12	39	1	709	74.7	0.007789055
NP_000969.1	60S ribosomal protein L23	RPL23	9349	0.663	46	6	50	6	140	14.9	0.002852486
NP_036220.1	6-phosphogluconolactonase	PGLS	25796	0.679	46	8	40	8	258	27.5	0.007165402
XP_016884680.1	E3 ubiquitin-protein ligase HUWE1 isoform X1	HUWE1	10075	0.841	5	12	34	12	4501	494	0.112926832
NP_036473.2	nucleolar GTP-binding protein 1	GTPBP4	23560	1.184	24	13	39	13	634	73.9	0.274857496
NP_004485.1	hepatoma-derived growth factor isoform a	HDGF	3068	1.041	55	12	44	10	240	26.8	0.776107414
NP_001744.2	caveolin-1 isoform alpha	CAV1	857	1.051	41	6	33	6	178	20.5	0.699514426
NP_036546.2	rab3 GTPase-activating protein non-catalytic subunit	RAB3GAP2	25782	0.655	13	17	41	16	1393	155.9	0.002872206
NP_066299.2	myosin light polypeptide 6 isoform 1	MYL6	4637	0.639	42	6	56	4	151	16.9	0.01418636
NP_000584.2	antigen peptide transporter 1 isoform 1	TAP1	6890	1.143	20	9	22	9	808	87.2	0.868902982
NP_001269282.1	integrin beta-6 isoform a precursor	ITGB6	3694	1.883	15	8	35	8	788	85.9	0.00356245
XP_005253288.1	CD44 antigen isoform X1	CD44	960	1.787	12	8	73	8	743	81.6	0.000254199
XP_005270945.1	nuclear autoantigenic sperm protein isoform X1	NASP	4678	1.14	21	14	42	14	790	86.2	0.371748832
NP_055045.2	signal recognition particle subunit SRP68 isoform 1	SRP68	6730	0.889	25	14	35	14	627	70.7	0.125326644
NP_127492.1	general transcription factor II-I isoform 1	GTF2I	2969	1.445	17	16	40	16	998	112.3	0.046949879
NP_000974.1	60S ribosomal protein L22	RPL22	6146	0.683	40	4	29	4	128	14.8	0.089414101
NP_060555.2	importin-9	IPO9	55705	0.454	16	11	41	11	1041	115.9	0.049251532
XP_005249668.1	chromobox protein homolog 3 isoform X1	CBX3	11335	1.19	53	7	39	6	183	20.8	0.607575335
NP_000182.2	hydroxymethylglutaryl-CoA lyase, mitochondrial isoform 1 precursor	HMGCL	3155	1.958	36	9	44	9	325	34.3	0.002609317
NP_001027454.1	thymopoietin isoform beta	TMPO	7112	1.433	30	11	32	6	454	50.6	0.17607875
NP_061185.1	protein RCC2	RCC2	55920	0.672	23	11	31	11	522	56	4.58E-05
NP_002096.1	histone H2AX	H2AFX	3014	1.247	38	7	92	1	143	15.1	0.092910991
NP_002808.3	26S proteasome non-ATPase regulatory subunit 13 isoform 1	PSMD13	5719	0.874	27	10	37	10	376	42.9	0.49564077
NP_001117.2	adenylosuccinate synthetase isozyme 2 isoform 1	ADSS	159	0.553	30	11	35	11	456	50.1	0.000779107
XP_016867006.1	A-kinase anchor protein 12 isoform X1	AKAP12	9590	1.475	13	14	31	14	1782	191.4	0.124261127
NP_002940.2	39S ribosomal protein L12, mitochondrial	MRPL12	6182	2.299	41	7	38	7	198	21.3	0.000516934
NP_001006666.1	ribosomal protein S6 kinase alpha-1 isoform b	RPS6KA1	6195	0.748	22	12	26	8	744	83.9	0.202194183
NP_000867.2	cation-independent mannose-6-phosphate receptor precursor	IGF2R	3482	1.11	8	14	32	14	2491	274.1	0.468819431
NP_001287847.1	high mobility group protein HMGI-C isoform c	HMGA2	8091	2.543	45	5	34	5	118	12.7	0.000716785
NP_061485.1	ras-related C3 botulinum toxin substrate 1 isoform Rac1b	RAC1	5879	1.089	36	9	46	5	211	23.5	0.540904149
NP_001020241.1	40S ribosomal protein S14	RPS14	6208	0.661	33	6	27	6	151	16.3	0.005668329
XP_005269798.1	annexin A11 isoform X1	ANXA11	311	0.705	19	11	34	11	605	65.6	0.034534706
NP_004588.1	small nuclear ribonucleoprotein Sm D2 isoform 1	SNRPD2	6633	1.272	62	9	38	9	118	13.5	0.005770997
XP_005254853.1	kinesin-like protein KIF23 isoform X1	KIF23	9493	1.551	14	10	29	10	974	111.6	0.118203009
NP_001653.1	ADP-ribosylation factor 5	ARF5	381	0.852	41	6	65	3	180	20.5	0.047761333
NP_064535.1	GTP-binding protein SAR1a	SAR1A	56681	0.85	34	6	50	3	198	22.4	0.010576507
NP_001684.2	V-type proton ATPase subunit B, brain isoform	ATP6V1B2	526	0.906	39	12	28	12	511	56.5	0.748390748
XP_011509125.1	rab3 GTPase-activating protein catalytic subunit isoform X1	RAB3GAP1	22930	1.022	16	11	26	11	1024	114.9	0.487876128
NP_005751.2	CCAAT/enhancer-binding protein zeta	CEBPZ	10153	1.214	15	12	35	12	1054	120.9	0.088609235
NP_001245238.1	probable 28S rRNA (cytosine(4447)-C(5))-methyltransferase isoform 3	NOP2	4839	1.235	16	11	33	11	845	92.8	0.619627723
XP_006719799.1	golgin subfamily A member 3 isoform X1	GOLGA3	2802	1.128	9	11	27	11	1498	167.3	0.475105856
NP_003277.1	DNA topoisomerase 1	TOP1	7150	1.838	17	12	39	12	765	90.7	0.037643048
XP_024303866.1	ATPase family AAA domain-containing protein 3A isoform X1	ATAD3A	55210	1.233	22	11	38	4	612	68.9	0.252244741
NP_003906.2	copine-1 isoform b	CPNE1	8904	0.826	21	9	36	9	542	59.7	0.004251942
NP_005076.3	nuclear pore complex protein Nup214 isoform 2	NUP214	8021	0.906	9	11	27	11	2090	213.5	0.716931015
NP_114414.2	eukaryotic translation initiation factor 2A isoform 1	EIF2A	83939	0.72	29	12	34	12	585	64.9	0.266879644
NP_056988.3	eukaryotic translation initiation factor 5B	EIF5B	9669	0.637	13	13	33	13	1220	138.7	0.016698441
NP_852133.1	eukaryotic translation initiation factor 6 isoform a	EIF6	3692	0.783	50	7	34	7	245	26.6	0.290455359
NP_009215.1	twinfilin-2	TWF2	11344	0.621	31	8	28	8	349	39.5	0.027384924
NP_002957.1	protein S100-A10	S100A10	6281	1.459	49	8	47	8	97	11.2	0.071164065
NP_002144.1	estradiol 17-beta-dehydrogenase 2	HSD17B2	3294	1.332	36	10	35	10	387	42.8	0.051873799
NP_001129064.1	ubiquitin-40S ribosomal protein S27a precursor	RPS27A	6233	0.961	47	7	82	7	156	18	0.663380776
NP_001016.1	40S ribosomal protein S23	RPS23	6228	0.764	35	6	34	6	143	15.8	0.147813314
NP_006351.2	eukaryotic translation initiation factor 3 subunit M isoform 1	EIF3M	10480	0.425	28	10	34	10	374	42.5	0.014282107
NP_036555.1	60S ribosomal protein L13a isoform 1	RPL13A	23521	0.927	35	11	59	11	203	23.6	0.116799781
NP_940999.1	protein transport protein Sec24C	SEC24C	9632	0.876	15	13	40	12	1094	118.2	0.646697785
NP_001298127.1	AP-2 complex subunit mu isoform c	AP2M1	1173	1.209	22	8	39	8	460	52.3	0.147727945
NP_000088.3	oxygen-dependent coproporphyrinogen-III oxidase, mitochondrial precursor	CPOX	1371	1.208	28	10	47	10	454	50.1	0.46788987
NP_001123612.1	endoplasmic reticulum aminopeptidase 2 isoform 1	ERAP2	64167	0.866	17	15	41	15	960	110.4	0.543315885
XP_006714129.1	PDZ and LIM domain protein 5 isoform X1	PDLIM5	10611	0.704	22	13	28	13	739	79	0.000641149
NP_001651.1	ADP-ribosylation factor 4	ARF4	378	0.671	45	7	65	5	180	20.5	0.004283918
NP_036335.1	glyoxylate reductase/hydroxypyruvate reductase	GRHPR	9380	0.707	37	7	30	7	328	35.6	0.026856164
XP_024306664.1	signal transducer and activator of transcription 3 isoform X5	STAT3	6774	0.93	19	11	31	11	802	91.4	0.811155932
NP_000970.1	60S ribosomal protein L18 isoform 1	RPL18	6141	0.894	34	6	57	6	188	21.6	0.16972477
NP_003696.2	calcium-binding mitochondrial carrier protein Aralar1	SLC25A12	8604	1.246	25	13	40	10	678	74.7	0.502290887
NP_057390.1	dnaJ homolog subfamily B member 11 precursor	DNAJB11	51726	1.715	27	8	24	8	358	40.5	0.084663366
XP_024308991.1	E3 ubiquitin-protein ligase TRIP12 isoform X1	TRIP12	9320	0.946	6	10	31	10	2092	231.5	0.156082412
NP_002195.1	integrin alpha-3 preproprotein	ITGA3	3675	1.324	10	9	27	9	1051	116.5	0.215324803
NP_005800.3	peroxiredoxin-2	PRDX2	7001	0.477	51	9	38	8	198	21.9	0.000220738
NP_001275891.1	cdc42-interacting protein 4 isoform 1	TRIP10	9322	0.884	27	11	34	11	601	68.3	0.625855305
NP_031401.1	TAR DNA-binding protein 43	TARDBP	23435	1.269	25	5	24	5	414	44.7	0.223328581
NP_054768.2	acyl-CoA dehydrogenase family member 9, mitochondrial	ACAD9	28976	0.945	20	10	31	10	621	68.7	0.636852373
NP_000476.1	adenine phosphoribosyltransferase isoform a	APRT	353	0.532	76	9	47	9	180	19.6	0.002316367
XP_016866762.1	protein DEK isoform X1	DEK	7913	1.067	29	13	36	13	380	43.2	0.685282331
NP_001229971.1	HLA class I histocompatibility antigen, Cw-1 alpha chain precursor	HLA-C	3107	0.691	33	8	27	2	366	40.7	0.067568938
NP_036557.1	ras suppressor protein 1 isoform 1	RSU1	6251	0.874	30	6	35	6	277	31.5	0.369067869
NP_057626.2	serine/threonine-protein kinase 26 isoform 1	STK26	51765	0.678	33	14	39	6	416	46.5	0.02654896
NP_001303936.1	protein S100-A16	S100A16	140576	0.83	46	5	29	5	103	11.8	0.477395386
NP_001005742.1	glucosylceramidase isoform 1 precursor	GBA	2629	1.877	23	10	36	10	536	59.7	0.004144653
NP_001137359.1	COP9 signalosome complex subunit 2 isoform 2	COPS2	9318	0.69	27	12	29	12	450	52.4	0.053325164
NP_001544.1	insulin-like growth factor-binding protein 7 isoform 1 precursor	IGFBP7	3490	2.104	31	7	30	7	282	29.1	0.00107764
NP_001352054.1	nuclear pore complex protein Nup98-Nup96 isoform 5	NUP98	4928	1.418	9	15	35	15	1831	198.9	0.004224238
XP_011528984.1	plexin-B2 isoform X1	PLXNB2	23654	1.179	9	11	26	11	1901	211.7	0.785995973
NP_003923.2	hsc70-interacting protein isoform 1	ST13	6767	0.517	22	7	35	7	369	41.3	0.000470168
NP_005969.1	protein S100-A2	S100A2	6273	0.291	35	7	69	7	97	11	0.001675833
NP_000981.1	60S ribosomal protein L27a	RPL27A	6157	0.625	38	7	34	7	148	16.6	0.000534814
XP_016879141.1	ubiquitin carboxyl-terminal hydrolase 7 isoform X1	USP7	7874	1.095	15	13	39	13	1113	129	0.467800221
NP_065796.1	alanine--tRNA ligase, mitochondrial	AARS2	57505	1.44	18	13	33	13	985	107.3	0.061376296
NP_112485.1	acylpyruvase FAHD1, mitochondrial isoform 2	FAHD1	81889	1.965	48	7	29	7	224	24.8	0.001815436
XP_006724878.1	host cell factor 1 isoform X1	HCFC1	3054	1.252	8	11	24	11	2080	213.3	0.316713191
NP_002939.2	60S ribosomal protein L15 isoform 1	RPL15	6138	0.789	43	9	38	9	204	24.1	0.444822504
XP_005262724.1	UDP-glucose 6-dehydrogenase isoform X1	UGDH	7358	0.808	31	11	25	11	507	56.4	0.467708404

Patil et al,2020. Proteomic and phosphoproteomic profiling of shammah-induced signaling in oral keratinocytes

Supplementary Table S1: List of proteins identified and quantified in OKF6/TERT1-Shammah and OKF6/TERT1-Parental total proteome using TMT-based quantitative proteomic approach

Accession	Protein Description	Gene Symbol	Gene ID	Fold Change	Coverage [%]	# Peptides	# PSMs	# Unique Peptides	# AAs	MW [kDa]	p-value
NP_001131026.1	malignant T-cell-amplified sequence 1 isoform 2	MCTS1	28985	0.586	47	5	18	5	182	20.5	0.355197958
NP_036601.2	pre-mRNA-processing factor 6	PRPF6	24148	1.257	15	14	37	14	941	106.9	0.298823023
NP_001229687.1	HLA class I histocompatibility antigen, A-1 alpha chain A*01:01:01:01 precursor	HLA-A	3105	1.437	41	10	35	5	365	40.8	0.033093639
XP_005250877.2	elongation factor 1-delta isoform X1	EEF1D	1936	0.689	13	8	40	6	647	71.4	0.001715469
NP_001124437.1	SUN domain-containing protein 1 isoform a	SUN1	23353	1.568	16	10	33	10	785	87.1	0.007316706
NP_078986.1	isochorismatase domain-containing protein 2 isoform 2	ISOC2	79763	1.199	42	5	25	5	221	24.1	0.221784588
NP_005561.1	protein ERGIC-53 precursor	LMAN1	3998	2.213	19	9	34	8	510	57.5	0.003557151
NP_008855.1	serine/arginine-rich splicing factor 1 isoform 1	SRSF1	6426	1.354	45	12	62	12	248	27.7	0.002450691
NP_060592.2	aspartate--tRNA ligase, mitochondrial isoform 1	DARS2	55157	1.426	22	11	38	11	645	73.5	0.016412999
NP_005442.2	PDZ and LIM domain protein 7 isoform 1	PDLIM7	9260	0.785	34	11	34	11	457	49.8	0.093016501
NP_001243284.1	ubiquitin-conjugating enzyme E2 L3 isoform 4	UBE2L3	7332	0.464	51	8	31	4	212	24	0.000702725
NP_057575.2	39S ribosomal protein L37, mitochondrial isoform 2	MRPL37	51253	1.446	35	11	45	11	423	48.1	0.037833792
NP_002892.1	reticulocalbin-1 precursor	RCN1	5954	1.803	42	11	34	11	331	38.9	0.037963065
NP_004308.2	ATPase ASNA1	ASNA1	439	0.738	32	8	27	8	348	38.8	0.204618224
NP_004542.1	NADH dehydrogenase	NDUFS3	4722	1.429	45	10	35	10	264	30.2	0.280069734
XP_005248776.1	unconventional myosin-VI isoform X1	MYO6	4646	0.771	10	10	27	10	1294	149.5	0.623248395
NP_000396.2	ganglioside GM2 activator isoform 1 precursor	GM2A	2760	1.678	51	8	58	8	193	20.8	0.068476572
NP_057164.3	glyoxalase domain-containing protein 4	GLOD4	51031	0.358	35	8	32	8	298	33.2	0.000846141
NP_064587.1	omega-amidase NIT2	NIT2	56954	0.583	45	11	36	11	276	30.6	0.035148244
NP_003744.1	eukaryotic translation initiation factor 3 subunit D	EIF3D	8664	0.758	26	10	30	10	548	63.9	0.534558556
NP_001034891.1	cell division control protein 42 homolog isoform 1 precursor	CDC42	998	0.909	37	6	50	6	191	21.2	0.620908988
XP_011513814.1	uridine phosphorylase 1 isoform X1	UPP1	7378	0.404	40	12	40	12	310	33.9	0.003282532
NP_002258.2	importin subunit alpha-4	KPNA3	3839	0.718	28	7	25	6	521	57.8	0.009560529
NP_002863.1	ras-related C3 botulinum toxin substrate 2	RAC2	5880	1.495	35	8	37	4	192	21.4	0.001679721
XP_011512974.1	serpin B6 isoform X1	SERPINB6	5269	0.615	24	8	29	7	454	51.2	0.001740342
NP_001011.1	40S ribosomal protein S16 isoform 1	RPS16	6217	0.742	49	8	68	8	146	16.4	0.180749515
XP_006711821.1	protein ELYS isoform X1	AHCTF1	25909	1.225	7	13	35	13	2301	255.8	0.404903435
NP_036265.3	coatomer subunit gamma-2 isoform 1	COPG2	26958	0.59	10	7	33	5	871	97.6	0.119945745
NP_006074.2	protein Red	IK	3550	1.335	22	10	24	10	557	65.6	0.331533908
NP_005907.3	DNA replication licensing factor MCM7 isoform 1	MCM7	4176	0.873	14	7	23	7	719	81.3	0.455027996
NP_005142.1	ubiquitin carboxyl-terminal hydrolase 14 isoform a	USP14	9097	0.683	26	9	38	9	494	56	0.023823998
NP_001139247.1	peptidyl-prolyl cis-trans isomerase FKBP5 isoform 1	FKBP5	2289	0.628	29	11	27	11	457	51.2	0.038523288
NP_005691.2	dipeptidyl peptidase 3 isoform 1	DPP3	10072	0.48	19	10	29	10	737	82.5	0.005388946
NP_001014446.1	OCIA domain-containing protein 2 isoform 1	OCIAD2	132299	0.934	43	6	38	6	154	16.9	0.689070521
NP_002515.1	GTPase NRas	NRAS	4893	1.316	52	7	28	2	189	21.2	0.772945135
NP_000977.1	60S ribosomal protein L24	RPL24	6152	0.685	21	4	38	4	157	17.8	3.17E-05
NP_005611.1	protein S100-A11	S100A11	6282	0.337	69	5	100	5	105	11.7	0.00382072
NP_055557.2	transmembrane 9 superfamily member 4 isoform 1 precursor	TM9SF4	9777	1.859	14	8	32	8	642	74.5	0.000593531
NP_002779.1	proteasome subunit alpha type-3 isoform 1	PSMA3	5684	0.68	31	8	26	8	255	28.4	0.159651819
NP_004209.2	ras-related protein Rab-11B	RAB11B	9230	1.379	39	9	40	8	218	24.5	0.009626049
NP_000971.1	60S ribosomal protein L18a	RPL18A	6142	0.675	41	9	37	9	176	20.7	0.005397893
NP_006640.2	U3 small nucleolar RNA-associated protein 14 homolog A isoform 1	UTP14A	10813	1.272	16	8	23	8	771	87.9	0.410529358
NP_002860.2	ras-related protein Rab-6A isoform a	RAB6A	5870	1.191	48	8	49	1	208	23.5	0.093800737
NP_778235.1	histone H2A type 2-B	HIST2H2AB	317772	1.971	35	5	79	1	130	14	0.610728919
NP_001719.2	basigin isoform 1 precursor	BSG	682	1.794	30	8	40	8	385	42.2	0.002260592
NP_001350464.1	probable histidine--tRNA ligase, mitochondrial isoform 4 precursor	HARS2	23438	1.045	23	11	35	6	512	57.5	0.954559835
NP_002259.1	importin subunit alpha-3	KPNA4	3840	0.694	26	7	16	6	521	57.9	0.122908049
XP_024308976.1	kynureninase isoform X1	KYNU	8942	0.41	24	8	27	8	465	52.3	0.017102435
NP_003267.1	thymopoietin isoform alpha	TMPO	7112	1.053	27	11	25	6	694	75.4	0.520960398
NP_003320.2	thioredoxin isoform 1	TXN	7295	0.403	43	7	64	7	105	11.7	0.000150366
NP_001243239.1	glutaminase kidney isoform, mitochondrial isoform 2	GLS	2744	1.519	20	9	28	9	598	65.4	0.01772048
NP_000932.3	NADPH--cytochrome P450 reductase	POR	5447	1	23	11	35	11	680	77	0.950221599
NP_004688.2	U4/U6 small nuclear ribonucleoprotein Prp4 isoform 1	PRPF4	9128	1.294	22	11	31	11	522	58.4	0.001339662
NP_005821.2	28S ribosomal protein S31, mitochondrial	MRPS31	10240	1.113	20	6	26	6	395	45.3	0.637545268
XP_016874389.1	diacylglycerol kinase alpha isoform X1	DGKA	1606	0.679	13	8	29	8	891	100.7	0.140255692
XP_024308482.1	catenin alpha-2 isoform X1	CTNNA2	1496	1.185	12	9	37	2	953	105.2	0.880650261
NP_001258665.1	coatomer subunit zeta-1 isoform 4	COPZ1	22818	0.695	26	5	19	5	185	21.1	0.075369081
NP_003841.1	succinate--CoA ligase	SUCLA2	8803	1.651	31	12	28	12	463	50.3	0.001040537
NP_000242.1	DNA mismatch repair protein Msh2 isoform 1	MSH2	4436	0.94	15	9	25	9	934	104.7	0.853216949
XP_024307207.1	peptidyl-prolyl cis-trans isomerase FKBP8 isoform X1	FKBP8	23770	1.475	28	8	30	8	442	47.2	0.055902097
NP_066949.2	3-mercaptopyruvate sulfurtransferase isoform 1	MPST	4357	1.333	31	7	23	7	317	35.2	0.110822668
NP_254280.1	histone H2A type 3	HIST3H2A	92815	2.366	36	7	230	1	130	14.1	0.000764768
NP_001338285.1	MMS19 nucleotide excision repair protein homolog isoform 6	MMS19	64210	0.738	12	9	20	9	1069	117.5	0.557230143
NP_057373.1	calcium-binding protein 39	CAB39	51719	0.311	37	14	41	14	341	39.8	0.001072241
NP_004729.1	vesicle-associated membrane protein-associated protein B/C isoform 1	VAPB	9217	2.211	28	8	39	7	243	27.2	0.000100275
NP_001041659.1	regulator of chromosome condensation isoform a	RCC1	1104	1.418	24	7	24	7	452	48.1	0.073453237
NP_078934.3	importin-4	IPO4	79711	0.531	9	6	26	6	1081	118.6	0.000118465
XP_024309834.1	farnesyl pyrophosphate synthase isoform X1	FDPS	2224	0.635	17	6	35	6	454	52.1	0.002066811
NP_079348.1	prostaglandin E synthase 2 isoform 1	PTGES2	80142	1.389	29	8	26	8	377	41.9	0.104633697
XP_016864628.1	drebrin isoform X1	DBN1	1627	1.152	21	8	24	8	697	76.4	0.61546673
NP_789845.1	inorganic pyrophosphatase 2, mitochondrial isoform 1 precursor	PPA2	27068	1.059	28	9	45	8	334	37.9	0.115536955
NP_001185648.1	histone-binding protein RBBP7 isoform 1	RBBP7	5931	1.329	22	9	31	4	469	52.3	0.020581363
XP_016885053.1	sterol-4-alpha-carboxylate 3-dehydrogenase, decarboxylating isoform X1	NSDHL	50814	1.581	27	9	28	9	389	43.8	0.080412254
NP_061820.1	cytochrome c	CYCS	54205	1.049	57	8	48	8	105	11.7	0.768490424
NP_003592.3	SWI/SNF-related matrix-associated actin-dependent regulator of chromatin subfamily A member 5	SMARCA5	8467	1.342	14	15	37	8	1052	121.8	0.453398769
NP_000180.2	hexokinase-2	HK2	3099	0.975	17	12	36	10	917	102.3	0.710154557
NP_001681.2	V-type proton ATPase catalytic subunit A	ATP6V1A	523	0.864	21	11	36	11	617	68.3	0.121930326
NP_006139.1	LIM and SH3 domain protein 1 isoform a	LASP1	3927	0.442	33	10	32	10	261	29.7	0.000222362
XP_005247749.1	guanine nucleotide-binding protein subunit beta-4 isoform X1	GNB4	59345	0.831	38	8	41	3	340	37.5	0.86055515
NP_060422.4	pentatricopeptide repeat domain-containing protein 3, mitochondrial precursor	PTCD3	55037	1.224	24	11	26	11	689	78.5	0.432281488
XP_005270330.1	nucleolar and coiled-body phosphoprotein 1 isoform X1	NOLC1	9221	0.738	19	12	34	1	710	74.8	0.597242306
NP_001026976.1	fatty aldehyde dehydrogenase isoform 1	ALDH3A2	224	1.023	13	6	27	6	508	57.6	0.961695457
NP_057366.2	GTP:AMP phosphotransferase AK3, mitochondrial isoform a	AK3	50808	2.599	55	11	37	11	227	25.6	0.000878093
NP_004351.1	cadherin-1 isoform 1 preproprotein	CDH1	999	1.606	19	10	34	9	882	97.4	0.012053688
NP_002094.2	glycogen	GYS1	2997	0.68	20	10	27	10	737	83.7	0.236029484
NP_004138.1	developmentally-regulated GTP-binding protein 1	DRG1	4733	0.762	28	8	28	8	367	40.5	0.07575895
NP_005601.1	histone-binding protein RBBP4 isoform a	RBBP4	5928	1.092	27	9	32	4	425	47.6	0.861195646
NP_000485.3	collagen alpha-1(XVII) chain	COL17A1	1308	1.688	5	9	28	9	1497	150.3	0.002186695
NP_001006.1	40S ribosomal protein S11	RPS11	6205	0.707	39	8	43	8	158	18.4	0.001129281
XP_005268645.1	cleavage and polyadenylation specificity factor subunit 6 isoform X1	CPSF6	11052	1.138	19	8	19	8	589	63.6	0.534636876
NP_001135888.1	aminoacyl tRNA synthase complex-interacting multifunctional protein 1 isoform b precursor	AIMP1	9255	0.851	38	9	29	9	336	37	0.001896991
NP_036478.2	nuclear pore glycoprotein p62	NUP62	23636	1.297	18	8	22	8	522	53.2	0.114396658
NP_612144.1	E3 ubiquitin-protein ligase DTX3L	DTX3L	151636	0.55	22	11	23	11	740	83.5	0.00639554
NP_060895.1	phosphatidylinositol 4-kinase type 2-alpha	PI4K2A	55361	1.004	29	10	31	10	479	54	0.999112567
NP_149124.3	2',3'-cyclic-nucleotide 3'-phosphodiesterase isoform 1	CNP	1267	1.286	19	6	20	6	421	47.5	0.304037412
NP_006406.1	serine palmitoyltransferase 1 isoform a	SPTLC1	10558	1.663	23	11	26	11	473	52.7	0.086080891
NP_001238968.1	ras-related protein Rab-5C isoform b	RAB5C	5878	0.916	41	7	36	4	249	27	0.662612228
NP_059122.2	nucleoporin p54 isoform 1	NUP54	53371	1.189	16	8	31	8	507	55.4	0.008184463
NP_001005353.1	adenylate kinase 4, mitochondrial isoform a	AK4	205	1.934	51	9	30	9	223	25.3	0.001969771
NP_067062.1	RNA-binding protein 25	RBM25	58517	1.394	13	10	35	10	843	100.1	0.043347889
NP_037460.2	pyrroline-5-carboxylate reductase 2 isoform 1	PYCR2	29920	2.387	28	8	32	7	320	33.6	6.84E-05
NP_003959.3	NEDD8-activating enzyme E1 catalytic subunit isoform 1	UBA3	9039	0.934	32	9	27	9	463	51.8	0.799286461
NP_942599.1	ras-related protein Rab-6A isoform b	RAB6A	5870	0.997	48	8	48	1	208	23.6	0.431346204
XP_016857451.1	ras-related protein Rap-1A isoform X2	RAP1A	5906	1.22	34	8	39	1	184	21	0.968620813
NP_006392.1	acidic leucine-rich nuclear phosphoprotein 32 family member B	ANP32B	10541	0.499	27	10	38	5	251	28.8	0.009944186
NP_055362.1	tropomodulin-3	TMOD3	29766	0.995	26	7	25	6	352	39.6	0.996018993
XP_011509985.1	POTE ankyrin domain family member J isoform X1	POTEJ	653781	0.84	6	6	246	2	1048	118.5	3.59E-05
NP_055416.2	EH domain-containing protein 2	EHD2	30846	0.98	28	12	34	10	543	61.1	0.950551093
NP_005619.1	neutral amino acid transporter B(0) isoform 1	SLC1A5	6510	1.048	16	6	28	6	541	56.6	0.521485767
NP_892116.2	eukaryotic translation initiation factor 5	EIF5	1983	0.79	20	9	36	8	431	49.2	0.079166156
NP_005723.2	DNA repair protein RAD50	RAD50	10111	1.067	11	13	35	13	1312	153.8	0.315486822
NP_002678.2	pinin	PNN	5411	1.855	17	10	26	10	717	81.6	0.084293305
NP_000967.1	60S ribosomal protein L12	RPL12	6136	0.783	50	7	43	7	165	17.8	0.164430096
NP_000994.1	60S acidic ribosomal protein P1 isoform 1	RPLP1	6176	0.822	57	3	36	3	114	11.5	0.019827538
XP_011526998.1	microtubule-associated protein RP/EB family member 1 isoform X1	MAPRE1	22919	0.62	52	10	36	10	268	30	0.007936264
NP_055454.1	tetratricopeptide repeat protein 37	TTC37	9652	0.594	10	12	20	12	1564	175.4	0.004845663
NP_789839.1	proteasome activator complex subunit 3 isoform 2	PSME3	10197	0.799	30	8	39	8	267	30.9	0.011375435
NP_004721.1	eukaryotic peptide chain release factor subunit 1 isoform 1	ETF1	2107	0.462	26	9	32	9	437	49	0.000199847
NP_001918.3	desmin	DES	1674	1.411	10	7	83	2	470	53.5	0.220169313
NP_000013.2	adenosine deaminase isoform 1	ADA	100	0.666	35	8	28	8	363	40.7	0.008942751
NP_000923.1	1-phosphatidylinositol 4,5-bisphosphate phosphodiesterase beta-3 isoform 1	PLCB3	5331	0.796	16	13	32	13	1234	138.7	0.603438176
NP_004214.1	ubiquitin/ISG15-conjugating enzyme E2 L6 isoform 1	UBE2L6	9246	0.52	49	6	17	6	153	17.8	0.031731201
NP_001295.2	carboxypeptidase D isoform 1 precursor	CPD	1362	1.38	9	10	28	10	1380	152.8	0.104694047
XP_005248837.1	flotillin-1 isoform X1	FLOT1	10211	1.166	24	8	26	8	427	47.3	0.010238056
NP_001909.3	lipoamide acyltransferase component of branched-chain alpha-keto acid dehydrogenase complex, mitochondrial precursor	DBT	1629	1.752	26	8	24	8	482	53.5	0.006978644
NP_001159583.1	neuropathy target esterase isoform a	PNPLA6	10908	1.102	11	9	27	9	1375	150.9	0.906481447
XP_011524077.1	serine/threonine-protein phosphatase 4 regulatory subunit 1 isoform X1	PPP4R1	9989	0.556	8	8	27	8	1002	112.2	0.039797142
NP_002787.2	proteasome subunit beta type-4	PSMB4	5692	0.704	42	7	34	7	264	29.2	0.112589021
NP_003625.2	protein NipSnap homolog 1 isoform 1	NIPSNAP1	8508	1.869	30	9	33	7	284	33.3	0.054664268
XP_005251987.1	golgin subfamily A member 2 isoform X1	GOLGA2	2801	1.251	11	9	27	9	1029	116	0.376655345
NP_006364.2	synaptic vesicle membrane protein VAT-1 homolog	VAT1	10493	0.892	30	9	35	9	393	41.9	0.462570994
NP_005505.2	major histocompatibility complex, class I, B precursor	HLA-B	3106	0.701	21	5	29	2	362	40.4	0.051737385
XP_016874792.1	prolow-density lipoprotein receptor-related protein 1 isoform X1	LRP1	4035	1.879	4	15	32	15	4561	506	0.002635573
NP_005361.2	ras-related protein Rab-8A	RAB8A	4218	1.485	36	8	61	3	207	23.7	0.439019901
NP_055181.3	guanine nucleotide-binding protein-like 3 isoform 1	GNL3	26354	1.193	16	9	29	9	549	62	0.065345637
NP_009134.1	PALM2-AKAP2 protein isoform 1	PALM2-AKAP2	445815	0.744	16	10	25	8	1103	122	0.420944578
NP_006764.3	ATP-dependent RNA helicase DDX18	DDX18	8886	1.573	21	12	32	12	670	75.4	0.005300169
XP_005249200.1	methylmalonyl-CoA mutase, mitochondrial isoform X1	MUT	4594	1.868	14	9	27	9	750	83.1	0.01534514
NP_006294.2	aminoacyl tRNA synthase complex-interacting multifunctional protein 2 isoform a	AIMP2	7965	0.683	42	8	30	8	320	35.3	0.006367721
NP_002097.1	histone H2A.Z	H2AFZ	3015	1.544	31	5	88	3	128	13.5	0.003645014
NP_060117.3	pre-rRNA processing protein FTSJ3	FTSJ3	117246	1.392	16	8	28	8	847	96.5	0.003893605
NP_006132.1	cytoplasmic dynein 1 light intermediate chain 2 isoform 1	DYNC1LI2	1783	0.583	33	9	20	9	492	54.1	0.033043374
NP_001975.1	S-formylglutathione hydrolase	ESD	2098	0.419	41	8	32	8	282	31.4	0.012209361
XP_016866367.1	afadin isoform X1	AFDN	4301	1.057	10	13	29	13	1832	207.6	0.846440876
NP_075066.1	39S ribosomal protein L44, mitochondrial	MRPL44	65080	1.535	23	6	25	6	332	37.5	0.003980027
NP_003120.2	squalene monooxygenase	SQLE	6713	1.192	22	9	22	9	574	63.9	0.391889204
XP_024309320.1	CTP synthase 1 isoform X1	CTPS1	1503	0.479	17	9	19	8	598	67.4	0.19532014
XP_016860322.1	POTE ankyrin domain family member F isoform X1	POTEF	728378	1.219	8	7	263	1	1085	122.5	0.449737805
XP_011511974.1	sister chromatid cohesion protein PDS5 homolog A isoform X1	PDS5A	23244	1.22	12	13	29	11	1337	150.7	0.39230386
NP_001007075.1	60S ribosomal protein L32	RPL32	6161	0.996	36	7	43	7	135	15.9	0.927304576
NP_036277.2	thymidylate kinase isoform 1	DTYMK	1841	0.921	43	10	24	10	212	23.8	0.189213984
NP_071415.1	methylcrotonoyl-CoA carboxylase beta chain, mitochondrial isoform 1 precursor	MCCC2	64087	1.204	28	11	24	11	563	61.3	0.597789979
NP_064576.1	28S ribosomal protein S22, mitochondrial isoform 1	MRPS22	56945	1.275	38	10	20	10	360	41.3	0.044719367
NP_001784.2	cadherin-3 isoform 1 preproprotein	CDH3	1001	2.091	15	8	27	7	829	91.4	0.003505389
XP_005268422.1	transcription elongation regulator 1 isoform X1	TCERG1	10915	1.268	10	10	33	10	1115	125.9	0.051777536
NP_001341838.1	paraspeckle component 1 isoform 1	PSPC1	55269	1.597	20	9	29	8	523	58.7	0.000202715
XP_016885242.1	spermine synthase isoform X1	SMS	6611	0.522	42	9	27	9	369	41.4	0.021536613
XP_016872115.1	ATP-dependent RNA helicase DDX50 isoform X1	DDX50	79009	1.283	13	9	31	7	737	82.5	0.424847752
NP_001139580.1	prostaglandin reductase 1 isoform 1	PTGR1	22949	0.581	20	7	27	7	329	35.8	0.011448306
NP_005330.1	ubiquitin-conjugating enzyme E2 K isoform 1	UBE2K	3093	0.594	54	7	24	7	200	22.4	0.004910116
NP_001248755.1	AP-3 complex subunit delta-1 isoform 3	AP3D1	8943	0.879	13	11	20	11	1215	136.6	0.246147952
NP_003745.1	eukaryotic translation initiation factor 3 subunit F	EIF3F	8665	0.731	26	7	31	7	357	37.5	0.184324332
NP_067026.3	signal recognition particle receptor subunit beta	SRPRB	58477	1.225	37	8	26	8	271	29.7	0.454974522
NP_001316055.1	dnaJ homolog subfamily C member 13 isoform 1	DNAJC13	23317	0.884	7	11	24	11	2248	254.9	0.54027888
NP_060934.1	CDGSH iron-sulfur domain-containing protein 1	CISD1	55847	1.912	44	5	29	5	108	12.2	0.002088542
NP_597728.1	phostensin	PPP1R18	170954	1.124	26	10	21	10	613	67.9	0.475547679
NP_056138.1	E3 UFM1-protein ligase 1	UFL1	23376	1.441	20	12	32	11	794	89.5	0.02422432
NP_004991.1	NADH dehydrogenase	NDUFA5	4698	1.156	60	5	21	5	116	13.5	0.275105827
NP_006444.2	transducin beta-like protein 3	TBL3	10607	1.169	14	7	28	7	808	89	0.149216889
NP_597709.2	ribonucleoprotein PTB-binding 1	RAVER1	125950	1.31	15	7	21	7	756	79.5	0.014111297
NP_998776.1	phosphate carrier protein, mitochondrial isoform b precursor	SLC25A3	5250	1.248	33	10	33	10	361	39.9	0.435718809
NP_001186797.1	glutaredoxin-3 isoform 1	GLRX3	10539	0.555	22	6	25	6	335	37.4	0.002806935
NP_002477.1	nuclear cap-binding protein subunit 1 isoform 1	NCBP1	4686	0.939	14	8	22	8	790	91.8	0.868645561
NP_031398.2	ATP-dependent RNA helicase DDX42	DDX42	11325	1.221	15	9	21	9	938	102.9	0.312202461
NP_006861.1	destrin isoform a	DSTN	11034	0.736	44	7	29	6	165	18.5	0.020788845
XP_011536691.1	nascent polypeptide-associated complex subunit alpha isoform X2	NACA	4666	0.327	3	5	27	5	2078	205.3	0.001067947
XP_011519406.1	dnaJ homolog subfamily C member 3 isoform X1	DNAJC3	5611	2.172	24	11	28	11	533	61	0.003183715
NP_005137.1	U4/U6.U5 tri-snRNP-associated protein 1	SART1	9092	1.5	15	9	29	9	800	90.2	0.01189276
XP_006714824.1	protocadherin Fat 2 isoform X1	FAT2	2196	0.735	4	11	21	11	4349	479	0.127457528
NP_859050.1	serine/threonine-protein phosphatase 2A 65 kDa regulatory subunit A beta isoform isoform b	PPP2R1B	5519	0.933	13	7	27	2	667	73.5	0.76225612
NP_073753.3	CUB domain-containing protein 1 isoform 1 precursor	CDCP1	64866	1.418	14	11	29	11	836	92.9	0.071597588
NP_002706.1	serine/threonine-protein phosphatase 2A catalytic subunit alpha isoform isoform 1	PPP2CA	5515	0.691	34	8	28	8	309	35.6	0.186031616
NP_057213.2	COP9 signalosome complex subunit 4 isoform 1	COPS4	51138	0.784	25	8	17	8	406	46.2	0.357882353
NP_002991.2	succinate dehydrogenase	SDHB	6390	1.49	36	10	33	10	280	31.6	0.013352007
NP_006321.1	acyl-protein thioesterase 1 isoform 1	LYPLA1	10434	0.929	41	6	26	6	230	24.7	0.73990648
NP_001304310.1	heat shock 70 kDa protein 4L isoform 2	HSPA4L	22824	0.645	14	9	23	5	870	97.6	0.535661397
NP_000984.1	60S ribosomal protein L31 isoform 1	RPL31	6160	0.7	49	8	59	8	125	14.5	0.048343342
XP_011539049.1	quinone oxidoreductase isoform X1	CRYZ	1429	1.119	26	7	23	7	329	35.2	0.596878234
XP_006715888.1	actin-related protein 2/3 complex subunit 1B isoform X1	ARPC1B	10095	0.749	26	8	20	8	372	40.9	0.123387456
NP_149975.1	GPI transamidase component PIG-S	PIGS	94005	1.529	20	7	27	7	555	61.6	0.000292097
XP_011514038.1	protein FAM3C isoform X1	FAM3C	10447	2.111	50	9	33	9	227	24.7	0.000340683
NP_009145.1	translocation protein SEC63 homolog	SEC63	11231	1.87	15	10	26	10	760	87.9	0.008572729
NP_002801.1	26S proteasome non-ATPase regulatory subunit 4 isoform 2	PSMD4	5710	0.976	27	7	23	7	377	40.7	0.732209184
NP_001135974.1	relA-associated inhibitor	PPP1R13L	10848	0.748	15	8	23	8	828	89	0.003962555
XP_024308591.1	mannose-1-phosphate guanyltransferase alpha isoform X1	GMPPA	29926	0.776	20	6	25	6	420	46.3	0.524823855
NP_009039.1	elongin-B isoform a	ELOB	6923	0.614	85	8	30	8	118	13.1	0.087449057
NP_036300.2	F-box only protein 2	FBXO2	26232	0.767	24	6	23	6	296	33.3	0.018835615
NP_061141.2	protein unc-45 homolog A isoform 2	UNC45A	55898	0.75	14	11	28	11	944	103	0.2098538
NP_004989.2	unconventional myosin-Ie	MYO1E	4643	0.564	10	10	34	10	1108	127	0.002537331
XP_016869434.1	copine-3 isoform X1	CPNE3	8895	0.764	22	9	25	8	537	60.1	0.339702358
NP_060676.2	vacuolar protein sorting-associated protein 35	VPS35	55737	0.517	19	12	30	12	796	91.6	0.049248203
NP_006798.1	chromobox protein homolog 1	CBX1	10951	1.911	46	6	35	5	185	21.4	0.001493006
NP_542984.2	39S ribosomal protein L39, mitochondrial isoform b	MRPL39	54148	1.557	19	7	24	7	353	40.4	0.009166982
NP_004246.2	cytochrome c oxidase subunit 5A, mitochondrial precursor	COX5A	9377	1.452	53	7	22	7	150	16.8	0.000783641
NP_001243339.1	ras-related protein Rab-18 isoform 2	RAB18	22931	1.271	43	8	34	8	235	26.4	0.291104192
NP_001304161.1	mitochondrial carrier homolog 2 isoform 2	MTCH2	23788	1.66	25	7	29	7	294	32.2	0.030311957
NP_001153408.1	probable rRNA-processing protein EBP2 isoform 1	EBNA1BP2	10969	1.565	24	8	25	8	361	40.7	0.001786234
XP_005245715.1	ubiquitin-associated protein 2-like isoform X1	UBAP2L	9898	0.654	11	8	21	8	1115	117.6	0.043565391
NP_001409.3	eukaryotic translation initiation factor 4 gamma 2 isoform 1	EIF4G2	1982	0.718	17	13	28	13	907	102.3	0.208050575
NP_001116105.1	carbamoyl-phosphate synthase	CPS1	1373	1.536	8	10	28	9	1506	165.5	0.005901668
NP_000777.1	lanosterol 14-alpha demethylase isoform 1 precursor	CYP51A1	1595	1.926	20	10	35	10	509	57.2	0.015324727
NP_001679.2	ATP synthase F(0) complex subunit B1, mitochondrial precursor	ATP5PB	515	1.477	30	9	36	9	256	28.9	0.025653605
NP_001190174.1	40S ribosomal protein S10	RPS10	6204	0.582	53	9	45	8	165	18.9	0.018891143
NP_055077.2	prolyl 3-hydroxylase 3 precursor	P3H3	10536	1.472	14	6	30	6	736	81.8	0.060106967
NP_001027565.1	heterogeneous nuclear ribonucleoprotein H2	HNRNPH2	3188	1.226	21	7	42	3	449	49.2	0.187832134
NP_055768.3	exosome complex exonuclease RRP44 isoform a	DIS3	22894	0.882	14	11	28	11	958	108.9	0.640446556
XP_016868262.1	tyrosine-protein kinase BAZ1B isoform X1	BAZ1B	9031	0.94	8	9	25	9	1483	170.8	0.075405068
NP_001032726.1	transcription factor BTF3 isoform A	BTF3	689	0.342	45	8	24	8	206	22.2	0.004040233
NP_001344.2	aldo-keto reductase family 1 member C1	AKR1C1	1645	0.504	32	8	38	4	323	36.8	0.009171916
XP_016864475.1	activated RNA polymerase II transcriptional coactivator p15 isoform X1	SUB1	10923	0.474	38	7	26	7	127	14.4	0.003473039
NP_001349700.1	casein kinase II subunit alpha isoform a	CSNK2A1	1457	0.983	30	8	17	7	391	45.1	0.878439595
NP_002547.1	oxysterol-binding protein 1	OSBP	5007	0.672	15	10	27	10	807	89.4	0.042111684
NP_003125.3	signal recognition particle 14 kDa protein isoform 1	SRP14	6727	1.143	52	6	23	6	136	14.6	0.5635064
NP_008878.3	signal recognition particle subunit SRP72 isoform 1	SRP72	6731	0.853	26	12	31	12	671	74.6	9.16E-05
NP_002803.2	26S proteasome non-ATPase regulatory subunit 8	PSMD8	5714	0.775	31	8	22	8	350	39.6	0.000548186
NP_006807.1	vesicular integral-membrane protein VIP36 precursor	LMAN2	10960	1.406	29	8	33	8	356	40.2	0.002946293
XP_006719132.1	GTPase KRas isoform X1	KRAS	3845	1.196	48	6	23	1	189	21.6	0.837280086
XP_016883082.1	protein transport protein Sec23B isoform X1	SEC23B	10483	0.68	13	7	28	4	767	86.4	0.056439209
NP_877423.1	DNA replication licensing factor MCM4	MCM4	4173	0.608	13	7	18	7	863	96.5	0.019612534
NP_001310991.1	transport and Golgi organization protein 1 homolog isoform 3 precursor	MIA3	375056	1.697	5	9	26	9	1931	216.1	0.010179485
XP_016874946.1	vacuolar protein sorting-associated protein 29 isoform X1	VPS29	51699	0.599	44	8	26	8	214	24	0.005769988
NP_001336387.1	small nuclear ribonucleoprotein-associated protein N	SNRPN	6638	1.122	23	7	34	7	240	24.6	0.711811599
NP_036347.1	protein O-GlcNAcase isoform a	OGA	10724	0.519	13	8	24	8	916	102.8	0.065267329
XP_016882419.1	microtubule-associated protein 1S isoform X1	MAP1S	55201	0.718	14	9	26	9	1068	113.1	0.300321408
NP_689419.2	sorting nexin-6 isoform b	SNX6	58533	0.653	22	9	23	9	418	47.8	0.12703847
NP_000680.2	retinal dehydrogenase 1	ALDH1A1	216	1.687	18	12	33	9	501	54.8	0.074467302
NP_057417.3	serine/arginine repetitive matrix protein 2	SRRM2	23524	1.401	6	9	23	9	2752	299.4	0.01100507
NP_006487.1	guanine nucleotide-binding protein G(k) subunit alpha	GNAI3	2773	1.146	34	9	43	4	354	40.5	0.082927382
NP_001000.2	40S ribosomal protein S5	RPS5	6193	0.767	28	5	47	5	204	22.9	0.16890284
NP_064555.2	nicalin isoform 1 precursor	NCLN	56926	1.45	18	9	23	9	563	62.9	0.006680002
NP_000972.1	60S ribosomal protein L19 isoform 1	RPL19	6143	0.63	22	6	38	6	196	23.5	0.000794218
NP_001350687.1	glutamine amidotransferase-like class 1 domain-containing protein 3B, mitochondrial isoform 1	GATD3B	102724023	2.039	28	6	23	6	268	28.1	0.005554041
NP_009167.1	calcineurin B homologous protein 1	CHP1	11261	1.656	52	9	29	9	195	22.4	0.005091946
NP_001265485.1	actin-related protein 2/3 complex subunit 3 isoform 1	ARPC3	10094	0.639	33	7	33	7	178	20.5	0.096063121
NP_065143.2	manganese-transporting ATPase 13A1	ATP13A1	57130	1.285	12	10	20	10	1204	132.9	0.196250529
NP_002802.2	26S proteasome non-ATPase regulatory subunit 7	PSMD7	5713	0.721	30	7	25	7	324	37	0.001420098
NP_057132.2	isochorismatase domain-containing protein 1	ISOC1	51015	0.648	30	6	22	6	298	32.2	0.114801801
NP_000091.1	cystatin-B	CSTB	1476	0.609	55	4	18	4	98	11.1	0.00205285
XP_016855505.1	serine/arginine repetitive matrix protein 1 isoform X7	SRRM1	10250	1.433	10	6	22	6	943	106	0.219051271
NP_001292553.1	calmodulin-2 isoform 1	CALM2	805	1.723	51	5	31	5	197	22.2	0.004434654
NP_008937.1	cleavage and polyadenylation specificity factor subunit 5	NUDT21	11051	1.003	47	9	31	9	227	26.2	0.992198333
NP_055554.1	bcl-2-associated transcription factor 1 isoform 1	BCLAF1	9774	0.993	15	11	26	11	920	106.1	0.890946568
NP_000089.1	carnitine O-palmitoyltransferase 2, mitochondrial isoform 1 precursor	CPT2	1376	1.697	18	8	23	8	658	73.7	0.015740952
NP_006491.2	cell surface glycoprotein MUC18 precursor	MCAM	4162	1.164	13	7	25	7	646	71.6	0.008175231
NP_056284.1	protein NipSnap homolog 3A isoform 1	NIPSNAP3A	25934	2.031	35	6	22	6	247	28.4	0.026527023
XP_024308739.1	dnaJ homolog subfamily C member 10 isoform X1	DNAJC10	54431	1.659	14	9	32	9	793	91	0.010945471
NP_005580.1	methylmalonate-semialdehyde dehydrogenase	ALDH6A1	4329	1.548	21	7	17	7	535	57.8	0.028384699
NP_001026859.1	phospholipase A-2-activating protein isoform 1	PLAA	9373	0.748	14	8	27	8	795	87.1	0.243641425
NP_002044.2	guanylate-binding protein 1	GBP1	2633	0.467	19	10	23	6	592	67.9	0.004347324
NP_002746.1	dual specificity mitogen-activated protein kinase kinase 1	MAP2K1	5604	0.409	29	10	25	6	393	43.4	0.000381612
NP_005906.2	DNA replication licensing factor MCM6	MCM6	4175	0.704	15	10	22	10	821	92.8	0.240354935
NP_001163887.1	tight junction protein ZO-2 isoform 3	TJP2	9414	0.674	11	9	22	9	1221	137.3	0.002040248
NP_057110.3	retinol dehydrogenase 11 isoform 1 precursor	RDH11	51109	1.818	26	8	32	8	318	35.4	0.019263466
NP_055955.1	tubulin--tyrosine ligase-like protein 12	TTLL12	23170	0.737	20	9	21	9	644	74.4	0.446506351
XP_005262729.1	protein NOXP20 isoform X1	FAM114A1	92689	0.727	12	5	15	5	563	60.7	0.059875836
XP_016859290.1	glutamine--fructose-6-phosphate aminotransferase	GFPT1	2673	0.456	16	10	26	10	724	81.5	0.00538559
NP_061903.2	ATP-dependent RNA helicase DHX29 isoform 1	DHX29	54505	0.754	8	8	18	8	1369	155.1	0.128775821
NP_001015509.1	peptidyl-tRNA hydrolase 2, mitochondrial isoform a	PTRH2	51651	1.802	43	5	15	5	180	19.3	0.119004749
NP_006581.2	U4/U6.U5 tri-snRNP-associated protein 2 isoform 1	USP39	10713	1.454	19	8	25	8	565	65.3	0.314316615
XP_011532244.1	cAMP-dependent protein kinase type II-alpha regulatory subunit isoform X1	PRKAR2A	5576	1.225	30	9	26	9	404	45.5	0.07424048
XP_005247494.1	importin subunit alpha-5 isoform X1	KPNA1	3836	0.878	24	9	20	5	538	60.2	0.419987258
NP_079172.2	endoplasmic reticulum metallopeptidase 1	ERMP1	79956	1.509	11	8	21	8	904	100.2	0.003225908
NP_659412.3	schlafen family member 5 isoform 1	SLFN5	162394	0.65	14	10	23	9	891	101	0.17076226
NP_055873.1	von Willebrand factor A domain-containing protein 8 isoform a precursor	VWA8	23078	1.216	6	10	20	10	1905	214.7	0.320375608
NP_001284529.1	hsp70-binding protein 1 isoform 1	HSPBP1	23640	0.509	28	7	21	7	405	44.4	0.007597476
NP_001139699.1	40S ribosomal protein S20 isoform 1	RPS20	6224	0.779	25	5	28	5	142	16	0.342317945
NP_006730.2	DNA replication licensing factor MCM5	MCM5	4174	0.653	17	9	27	9	734	82.2	0.299119112
NP_006467.4	ATP synthase subunit g, mitochondrial	ATP5MG	10632	1.789	53	6	32	6	103	11.4	0.053921844
NP_001073884.1	U2 snRNP-associated SURP motif-containing protein isoform 1	U2SURP	23350	0.944	11	9	22	9	1029	118.2	0.948006463
NP_001098016.1	ribonuclease P protein subunit p30 isoform a	RPP30	10556	1.205	37	8	20	8	322	35.5	0.007104333
NP_054861.2	ADP-sugar pyrophosphatase isoform 1	NUDT5	11164	0.377	22	6	19	6	219	24.3	0.001684631
NP_005521.1	isocitrate dehydrogenase	IDH3A	3419	1.811	34	10	29	10	366	39.6	0.010497398
XP_016868076.1	zyxin isoform X1	ZYX	7791	0.486	13	8	23	8	631	67.1	0.052326203
NP_057614.1	ras-related protein Rab-8B	RAB8B	51762	1.855	38	8	59	3	207	23.6	0.722121943
NP_000518.1	low-density lipoprotein receptor isoform 1 precursor	LDLR	3949	1.792	13	9	26	9	860	95.3	0.248877346
NP_004860.2	vacuolar protein sorting-associated protein 4B	VPS4B	9525	0.681	20	10	29	5	444	49.3	0.104400151
NP_001171485.1	propionyl-CoA carboxylase beta chain, mitochondrial isoform 2 precursor	PCCB	5096	1.222	20	8	18	8	559	60.5	0.410836941
NP_003361.1	vasodilator-stimulated phosphoprotein	VASP	7408	0.939	27	8	16	8	380	39.8	0.874058432
NP_110400.1	tubulin beta-1 chain	TUBB1	81027	0.698	12	7	68	1	451	50.3	8.77E-05
NP_001171062.1	serine/threonine-protein phosphatase 2A 55 kDa regulatory subunit B alpha isoform isoform 2	PPP2R2A	5520	0.523	25	9	29	9	457	53	0.012436243

Patil et al,2020. Proteomic and phosphoproteomic profiling of shammah-induced signaling in oral keratinocytes

Supplementary Table S1: List of proteins identified and quantified in OKF6/TERT1-Shammah and OKF6/TERT1-Parental total proteome using TMT-based quantitative proteomic approach

Accession	Protein Description	Gene Symbol	Gene ID	Fold Change	Coverage [%]	# Peptides	# PSMs	# Unique Peptides	# AAs	MW [kDa]	p-value
NP_001269210.1	pyrroline-5-carboxylate reductase 1, mitochondrial isoform 5	PYCR1	5831	3.837	18	5	23	4	346	36	0.004871699
NP_002085.2	eukaryotic peptide chain release factor GTP-binding subunit ERF3A isoform 1	GSPT1	2935	0.465	15	9	26	9	637	68.6	9.10E-06
NP_004563.2	plakophilin-2 isoform 2b	PKP2	5318	1.278	9	6	14	6	881	97.4	0.501645938
NP_008996.1	hsp90 co-chaperone Cdc37	CDC37	11140	0.58	23	7	30	7	378	44.4	0.085661632
NP_000364.1	uridine 5'-monophosphate synthase	UMPS	7372	0.82	19	7	25	7	480	52.2	0.036924972
NP_003896.1	NEDD8-activating enzyme E1 regulatory subunit isoform a	NAE1	8883	0.649	25	7	21	7	534	60.2	0.0172506
NP_006793.1	splicing factor 3A subunit 3 isoform 1	SF3A3	10946	1.36	20	7	25	7	501	58.8	0.040171913
NP_001229820.1	cold shock domain-containing protein E1 isoform 4	CSDE1	7812	0.581	14	13	39	13	844	93.7	0.008287258
NP_001254500.1	TBC1 domain family member 2A isoform 1	TBC1D2	55357	0.592	10	6	23	6	928	105.3	9.89E-05
NP_005919.2	lactadherin isoform a preproprotein	MFGE8	4240	1.578	27	8	20	8	387	43.1	0.175194685
NP_064621.3	39S ribosomal protein L1, mitochondrial precursor	MRPL1	65008	1.455	24	5	22	5	325	36.9	0.048406102
XP_011531579.1	ubiquitin-like modifier-activating enzyme ATG7 isoform X1	ATG7	10533	0.599	16	10	26	10	738	81.7	0.003521962
NP_060140.2	ubiquitin thioesterase OTUB1	OTUB1	55611	0.474	34	7	24	7	271	31.3	0.001044894
NP_002766.1	serine protease HTRA1 precursor	HTRA1	5654	0.542	20	8	24	8	480	51.3	0.00023286
NP_060220.3	all-trans-retinol 13,14-reductase precursor	RETSAT	54884	1.121	22	9	21	9	610	66.8	0.273720791
XP_006718732.1	spectrin beta chain, non-erythrocytic 2 isoform X1	SPTBN2	6712	1.098	5	10	28	3	2397	271.5	0.988431279
NP_058642.1	junctional adhesion molecule A isoform 1 precursor	F11R	50848	2.783	28	6	29	6	299	32.6	0.000151985
NP_005660.4	receptor expression-enhancing protein 5	REEP5	7905	1.264	21	8	30	8	189	21.5	0.143501853
XP_016868586.1	cathepsin B isoform X1	CTSB	1508	2.026	23	7	32	7	339	37.8	1.10E-05
NP_002877.2	ras-related protein Rap-2b	RAP2B	5912	1.647	42	6	21	6	183	20.5	0.085617792
NP_002063.2	guanine nucleotide-binding protein G(q) subunit alpha	GNAQ	2776	0.987	28	9	32	6	359	42.1	0.879449019
NP_057122.2	ribosome maturation protein SBDS	SBDS	51119	0.579	41	12	27	12	250	28.7	0.020520662
NP_065777.1	neurolysin, mitochondrial	NLN	57486	0.942	12	8	20	8	704	80.6	0.901315632
XP_024306434.1	flotillin-2 isoform X1	FLOT2	2319	1.703	18	7	20	7	461	50.7	0.116407314
NP_003080.2	U1 small nuclear ribonucleoprotein 70 kDa isoform 1	SNRNP70	6625	1.497	16	7	25	7	437	51.5	0.000103757
NP_001028675.1	GTP-binding protein SAR1b	SAR1B	51128	0.526	25	5	30	2	198	22.4	0.02116659
NP_006796.1	heterogeneous nuclear ribonucleoprotein A0	HNRNPA0	10949	1.216	29	8	54	6	305	30.8	0.193608501
NP_008860.4	helicase SKI2W	SKIV2L	6499	0.723	9	8	21	8	1246	137.7	0.09117545
NP_006100.2	protein arginine N-methyltransferase 5 isoform a	PRMT5	10419	0.631	23	11	30	11	637	72.6	0.033607154
NP_001435.1	fatty acid-binding protein 5	FABP5	2171	0.637	59	7	36	7	135	15.2	0.016084056
NP_056167.1	GDP-fucose protein O-fucosyltransferase 1 isoform 1 precursor	POFUT1	23509	1.042	20	5	14	5	388	43.9	0.860762769
XP_011518045.1	cullin-2 isoform X1	CUL2	8453	0.694	14	9	23	9	808	94.2	0.401780336
NP_006651.2	ATP-dependent Clp protease ATP-binding subunit clpX-like, mitochondrial precursor	CLPX	10845	1.204	19	10	29	10	633	69.2	0.482641843
XP_005263604.1	mitofusin-2 isoform X1	MFN2	9927	1.734	12	7	20	6	757	86.3	0.062533683
NP_036377.1	SNW domain-containing protein 1 isoform 2	SNW1	22938	1.535	24	9	26	9	536	61.5	0.230925938
NP_001180197.1	echinoderm microtubule-associated protein-like 2 isoform 1	EML2	24139	0.641	11	8	23	8	850	92	0.081409206
XP_005261252.2	pyridoxal kinase isoform X1	PDXK	8566	0.809	24	5	24	5	406	44.2	0.440212409
NP_001317398.1	coatomer subunit epsilon isoform d	COPE	11316	0.65	28	6	22	6	331	36.9	0.166246995
NP_001273201.1	translationally-controlled tumor protein isoform 1	TPT1	7178	0.56	35	5	36	5	197	22.6	0.004251224
XP_011528354.1	phosphatidylinositol transfer protein beta isoform isoform X1	PITPNB	23760	0.482	43	12	31	9	274	31.8	0.00756689
NP_006296.1	acidic leucine-rich nuclear phosphoprotein 32 family member A	ANP32A	8125	0.68	22	8	33	3	249	28.6	0.225761285
NP_003840.2	succinate--CoA ligase	SUCLG1	8802	1.843	24	6	33	6	346	36.2	0.006150661
NP_001310826.1	regulator of microtubule dynamics protein 3 isoform 3	RMDN3	55177	1.387	17	5	17	5	496	54.8	0.004919848
NP_060037.4	N-acetyl-D-glucosamine kinase isoform 1	NAGK	55577	0.385	22	6	23	6	344	37.4	0.000876737
NP_150093.1	transcriptional activator protein Pur-beta	PURB	5814	1.213	36	6	19	5	312	33.2	0.253619425
NP_001181.2	branched-chain-amino-acid aminotransferase, mitochondrial isoform a precursor	BCAT2	587	1.395	18	5	16	5	392	44.3	0.018490419
NP_039234.1	chloride intracellular channel protein 4	CLIC4	25932	0.554	28	5	18	4	253	28.8	0.01776825
NP_061156.1	N-acylneuraminate cytidylyltransferase	CMAS	55907	1.813	23	8	26	8	434	48.3	0.010320974
XP_011530070.1	palladin isoform X1	PALLD	23022	0.788	8	8	21	8	1468	159.6	0.10625344
NP_001035526.1	tyrosine--tRNA ligase, mitochondrial precursor	YARS2	51067	1.201	23	7	19	7	477	53.2	0.861854355
NP_003869.1	gamma-glutamyl hydrolase precursor	GGH	8836	1.297	21	6	22	6	318	35.9	0.359436048
NP_665906.1	high mobility group protein HMG-I/HMG-Y isoform a	HMGA1	3159	1	31	4	27	2	107	11.7	0.980002963
NP_055706.1	28 kDa heat- and acid-stable phosphoprotein	PDAP1	11333	0.577	30	7	19	7	181	20.6	0.017605152
NP_114108.1	28S ribosomal protein S5, mitochondrial isoform a	MRPS5	64969	1.168	26	10	31	10	430	48	0.410066486
NP_005198.1	crk-like protein	CRKL	1399	0.509	31	6	21	6	303	33.8	0.000790366
NP_079272.4	elongation factor G, mitochondrial isoform 2	GFM1	85476	1.552	18	9	24	9	751	83.4	0.01570575
NP_149073.1	SAP domain-containing ribonucleoprotein	SARNP	84324	1.228	34	7	25	7	210	23.7	0.277831254
NP_001308032.1	sodium bicarbonate cotransporter 3 isoform d	SLC4A7	9497	0.797	9	8	24	8	1259	140.7	0.550993681
NP_001122389.1	mitochondrial import receptor subunit TOM40 homolog	TOMM40	10452	1.143	21	7	27	7	361	37.9	0.217990097
NP_004166.1	small nuclear ribonucleoprotein Sm D3	SNRPD3	6634	1.318	32	3	50	3	126	13.9	0.000948548
NP_057055.2	28S ribosomal protein S7, mitochondrial	MRPS7	51081	1.281	33	7	19	7	242	28.1	0.022939282
NP_002078.1	granulins precursor	GRN	2896	1.67	17	6	15	6	593	63.5	0.001009299
NP_055112.2	persulfide dioxygenase ETHE1, mitochondrial isoform 1	ETHE1	23474	3.087	22	5	20	5	254	27.9	0.010336438
NP_001024.1	ribonucleoside-diphosphate reductase large subunit isoform 1	RRM1	6240	0.688	12	7	16	7	792	90	0.08084264
NP_061819.2	sialic acid synthase	NANS	54187	0.497	31	8	26	8	359	40.3	0.001537293
NP_060272.3	dynein assembly factor 5, axonemal	DNAAF5	54919	0.68	13	9	22	9	855	93.5	0.173694656
NP_057327.2	NADH-cytochrome b5 reductase 1	CYB5R1	51706	1.362	33	8	21	8	305	34.1	0.074449997
NP_001295155.1	40S ribosomal protein S15 isoform 1	RPS15	6209	0.538	53	5	22	5	152	17.7	0.114275213
NP_005552.3	lysosome-associated membrane glycoprotein 1 precursor	LAMP1	3916	1.153	10	4	25	4	417	44.9	0.248502987
NP_060668.2	dnaJ homolog subfamily C member 11	DNAJC11	55735	1.86	16	7	23	7	559	63.2	0.040138478
XP_005265126.1	golgin subfamily A member 4 isoform X1	GOLGA4	2803	1.289	5	12	20	11	2285	266.7	0.039320332
NP_001186408.1	CORO7-PAM16 protein	CORO7-PAM16	100529144	1.395	10	7	20	7	1048	114.1	0.388304059
XP_011532633.1	serine/threonine-protein kinase OSR1 isoform X1	OXSR1	9943	0.55	16	7	20	7	580	63.8	0.003967664
NP_001309722.1	insulin-degrading enzyme isoform 3	IDE	3416	0.597	11	8	20	8	1019	117.9	0.008137796
NP_003737.1	dynein light chain 1, cytoplasmic	DYNLL1	8655	1.207	57	8	25	5	89	10.4	0.817036513
NP_004517.2	DNA replication licensing factor MCM2	MCM2	4171	0.593	10	8	20	8	904	101.8	0.070006616
NP_057113.1	dehydrogenase/reductase SDR family member 7 isoform 1 precursor	DHRS7	51635	1.825	27	7	22	7	339	38.3	0.012342666
XP_016857364.1	protein S100-A14 isoform X1	S100A14	57402	1.582	76	6	38	6	104	11.7	0.157941039
NP_258412.1	inosine triphosphate pyrophosphatase isoform a	ITPA	3704	0.394	51	6	23	6	194	21.4	0.024499388
NP_006852.1	ras-related protein Rab-35 isoform 1	RAB35	11021	1.228	32	6	55	4	201	23	0.023116206
XP_006710735.1	endophilin-B1 isoform X1	SH3GLB1	51100	0.8	28	8	20	7	402	45.2	0.002704005
NP_003524.1	histone H3.1	HIST1H3I	8354	0.478	58	8	87	1	136	15.4	0.020201142
NP_005960.1	nucleosome assembly protein 1-like 4	NAP1L4	4676	0.524	18	6	26	4	375	42.8	8.81E-05
XP_024307176.1	delta(3,5)-Delta(2,4)-dienoyl-CoA isomerase, mitochondrial isoform X1	ECH1	1891	1.984	27	8	22	8	390	42.1	0.00264681
XP_005263784.1	ras-related protein Ral-B isoform X1	RALB	5899	1.6	27	6	22	3	206	23.4	0.084263004
XP_024304145.1	probable ATP-dependent RNA helicase DDX6 isoform X1	DDX6	1656	0.868	24	8	22	8	483	54.4	0.19826374
XP_005252973.1	rho-related GTP-binding protein RhoG isoform X1	RHOG	391	1.425	46	7	22	7	191	21.3	0.014059516
NP_002379.3	DNA replication licensing factor MCM3 isoform 1	MCM3	4172	0.736	11	7	25	7	853	95.8	0.158064952
NP_004274.1	ras-related protein Rab-3D	RAB3D	9545	1.781	38	7	32	4	219	24.3	0.001380541
NP_056195.3	sorting and assembly machinery component 50 homolog	SAMM50	25813	1.238	19	8	23	8	469	51.9	0.006870909
NP_658985.2	NAD(P)H-hydrate epimerase precursor	NAXE	128240	0.601	26	5	14	5	288	31.7	0.108247367
NP_000980.1	60S ribosomal protein L30	RPL30	6156	0.989	57	5	35	5	115	12.8	0.851290508
NP_004682.2	V-type proton ATPase subunit d 1	ATP6V0D1	9114	1.554	27	9	28	9	351	40.3	0.202427131
NP_055144.3	enhancer of mRNA-decapping protein 4	EDC4	23644	0.927	7	7	21	7	1401	151.6	0.847415818
NP_076424.1	DDRGK domain-containing protein 1	DDRGK1	65992	1.11	25	5	18	5	314	35.6	0.593014393
XP_011509591.1	fumarylacetoacetate hydrolase domain-containing protein 2A isoform X3	FAHD2A	51011	1.297	26	8	22	3	314	34.6	0.147267371
NP_004102.1	flap endonuclease 1	FEN1	2237	0.657	24	8	26	8	380	42.6	0.00133241
NP_000296.2	serum paraoxonase/arylesterase 2 isoform 1	PON2	5445	1.275	32	6	21	6	354	39.4	0.229522735
NP_001203.1	complement component 1 Q subcomponent-binding protein, mitochondrial precursor	C1QBP	708	0.693	28	6	23	6	282	31.3	0.019409664
NP_002558.1	phosphatidylethanolamine-binding protein 1	PEBP1	5037	0.593	64	7	28	7	187	21	0.004870377
NP_000966.2	60S ribosomal protein L11 isoform 1	RPL11	6135	0.94	31	5	42	5	178	20.2	0.441187177
NP_005792.1	eukaryotic translation initiation factor 1	EIF1	10209	0.65	42	6	17	6	113	12.7	0.294447517
NP_055464.1	scaffold attachment factor B2	SAFB2	9667	1.439	13	10	21	4	953	107.4	0.001257561
NP_004715.1	centromere/kinetochore protein zw10 homolog	ZW10	9183	0.753	13	6	19	6	779	88.8	0.088643034
NP_004499.2	isopentenyl-diphosphate Delta-isomerase 1 isoform a	IDI1	3422	0.628	20	6	23	6	284	32.5	0.085547168
NP_037520.1	prolactin regulatory element-binding protein isoform 1	PREB	10113	1.902	21	5	22	5	417	45.4	0.012894999
NP_060167.2	epithelial splicing regulatory protein 1 isoform 1	ESRP1	54845	0.947	19	6	19	5	681	75.5	0.879317823
NP_001034180.1	ribose-phosphate pyrophosphokinase 2 isoform 1	PRPS2	5634	0.511	32	8	29	3	321	35	0.022353567
NP_057368.3	CCR4-NOT transcription complex subunit 1 isoform a	CNOT1	23019	0.907	4	8	20	8	2376	266.8	0.427409634
XP_016870597.1	guanine nucleotide exchange factor VAV2 isoform X1	VAV2	7410	0.757	11	7	22	7	896	103	0.206061562
XP_024302228.1	phosphoacetylglucosamine mutase isoform X4	PGM3	5238	0.713	16	8	21	8	570	62.9	0.062886247
NP_631961.1	TATA-binding protein-associated factor 2N isoform 1	TAF15	8148	0.982	14	6	28	4	592	61.8	0.878695692
XP_011534404.1	utrophin isoform X1	UTRN	7402	1.18	4	8	16	8	3469	398	0.801777221
NP_006102.2	3-ketoacyl-CoA thiolase, mitochondrial	ACAA2	10449	2.244	23	6	26	6	397	41.9	3.53E-05
XP_006718110.1	Golgi-specific brefeldin A-resistance guanine nucleotide exchange factor 1 isoform X1	GBF1	8729	0.718	6	10	17	10	1893	210.2	0.180676695
NP_861448.2	transmembrane and TPR repeat-containing protein 3	TMTC3	160418	1.512	13	9	18	9	914	103.8	0.065098098
XP_024302438.1	gasdermin-E isoform X1	GSDME	1687	0.788	17	7	22	7	496	54.5	0.050181287
NP_057724.2	39S ribosomal protein S30, mitochondrial	MRPS30	10884	1.653	28	7	19	7	439	50.3	0.011885313
NP_008869.1	small nuclear ribonucleoprotein Sm D1 isoform 1	SNRPD1	6632	1.207	37	4	43	4	119	13.3	0.601867776
NP_071908.2	nucleoredoxin isoform 1	NXN	64359	0.529	23	6	18	6	435	48.4	0.003842189
NP_001304106.1	MICOS complex subunit MIC19 isoform 1	CHCHD3	54927	1.612	24	6	24	6	232	26.7	0.001402599
XP_024307774.1	helicase with zinc finger domain 2 isoform X1	HELZ2	85441	0.807	4	8	16	8	2677	297.3	0.765943005
NP_005984.3	tubulin-specific chaperone D	TBCD	6904	0.485	9	8	16	8	1192	132.5	0.429702248
NP_001191455.1	translocon-associated protein subunit delta isoform 1 precursor	SSR4	6748	1.587	23	3	19	3	184	20.2	0.215073468
NP_060197.4	transmembrane protein 214 isoform 1	TMEM214	54867	1.421	17	10	23	10	689	77.1	0.13029536
NP_733844.1	delta-1-pyrroline-5-carboxylate dehydrogenase, mitochondrial isoform a precursor	ALDH4A1	8659	1.449	18	8	18	8	563	61.7	0.101603636
NP_002790.1	proteasome subunit beta type-7 precursor	PSMB7	5695	0.744	23	7	32	7	277	29.9	0.126434543
XP_005260640.1	mRNA export factor isoform X3	RAE1	8480	1.35	24	8	18	8	406	45	0.305598579
XP_011517567.1	polypyrimidine tract-binding protein 3 isoform X1	PTBP3	9991	1.032	10	6	34	3	561	60.7	0.888248521
NP_036252.1	CD2-associated protein	CD2AP	23607	0.369	17	8	23	8	639	71.4	0.030264789
XP_005246733.3	pre-mRNA-processing factor 40 homolog A isoform X2	PRPF40A	55660	1.351	10	9	20	9	1036	116.8	0.719726372
NP_001935.2	desmoglein-3 preproprotein	DSG3	1830	1.454	10	7	26	7	999	107.5	0.241264273
XP_005270360.1	cyclin-dependent kinase 1 isoform X1	CDK1	983	0.524	33	8	22	6	297	34.1	0.015453962
NP_004153.2	ras-related protein Rab-5A isoform 1	RAB5A	5868	0.922	33	5	26	2	215	23.6	0.999525207
XP_011511210.1	plexin-A1 isoform X1	PLXNA1	5361	1.422	7	10	26	7	1914	212.9	0.366013408
NP_001335116.1	probable ATP-dependent RNA helicase DDX27 isoform 1	DDX27	55661	1.609	12	10	27	10	827	93.2	5.93E-05
NP_062556.2	RNA binding motif protein, X-linked-like-1	RBMXL1	494115	1.385	13	4	34	1	390	42.1	0.209462851
NP_065147.1	ATP-dependent RNA helicase DDX24	DDX24	57062	1.055	13	8	20	8	859	96.3	0.747360947
NP_114127.3	ATPase family AAA domain-containing protein 3B isoform AAA-TOB3l	ATAD3B	83858	1.383	15	8	24	1	648	72.5	0.070359963
XP_005253109.1	synembryn-A isoform X1	RIC8A	60626	0.787	18	8	21	8	535	60.1	0.13962299
NP_006712.2	adenosine kinase isoform b	ADK	132	0.448	31	10	26	10	362	40.5	0.01076526
XP_024302117.1	band 4.1-like protein 2 isoform X7	EPB41L2	2037	1.088	12	8	20	8	1079	120.3	0.404396424
NP_001025178.1	AP-1 complex subunit gamma-1 isoform a	AP1G1	164	0.862	8	6	19	6	825	91.7	0.367922756
NP_001238965.1	ras-related protein Rab-5B isoform 1	RAB5B	5869	0.902	30	5	26	2	215	23.7	0.733404515
XP_016859148.1	CLIP-associating protein 1 isoform X3	CLASP1	23332	0.697	6	7	17	1	1554	171.3	0.032072209
XP_005272732.1	probable ubiquitin carboxyl-terminal hydrolase FAF-X isoform X1	USP9X	8239	0.791	4	9	16	9	2575	292.7	0.303265304
NP_149078.1	alpha-1,3/1,6-mannosyltransferase ALG2	ALG2	85365	1.931	27	7	22	7	416	47.1	0.001816771
NP_909122.1	RNA-binding protein 39 isoform a	RBM39	9584	1.342	10	5	27	5	530	59.3	0.021191876
NP_005138.3	dnaJ homolog subfamily A member 3, mitochondrial isoform 1	DNAJA3	9093	1.315	18	8	19	8	480	52.5	0.445261715
XP_011540393.1	leucine zipper protein 1 isoform X1	LUZP1	7798	1.216	10	10	20	10	1076	120.2	0.130004563
XP_011511152.1	melanotransferrin isoform X1	MELTF	4241	2.579	18	9	23	9	765	83.1	0.011691295
NP_065134.1	nuclear pore complex protein Nup107 isoform 1	NUP107	57122	1.176	10	7	16	7	925	106.3	0.512456974
NP_000185.1	hypoxanthine-guanine phosphoribosyltransferase	HPRT1	3251	0.804	39	6	29	6	218	24.6	0.095065079
NP_004716.1	mitotic checkpoint protein BUB3 isoform a	BUB3	9184	1.128	22	7	21	7	328	37.1	0.189664677
NP_733779.1	S-phase kinase-associated protein 1 isoform b	SKP1	6500	0.804	44	7	18	7	163	18.6	0.238333688
NP_004809.2	probable ATP-dependent RNA helicase DDX23	DDX23	9416	1.458	13	9	23	9	820	95.5	0.255037057
NP_003104.2	nuclear autoantigen Sp-100 isoform 2	SP100	6672	0.812	7	7	33	6	879	100.4	0.268207071
NP_113608.1	39S ribosomal protein L9, mitochondrial isoform 1	MRPL9	65005	1.276	31	8	22	8	267	30.2	0.001102282
XP_016866694.1	dystonin isoform X1	DST	667	0.529	2	9	18	1	7827	888.9	0.001530214
XP_016873620.1	epidermal growth factor receptor kinase substrate 8-like protein 2 isoform X1	EPS8L2	64787	0.684	20	9	17	9	715	80.6	0.429096631
XP_005272072.1	endoplasmic reticulum aminopeptidase 1 isoform X1	ERAP1	51752	0.826	10	8	25	8	951	108.3	0.058713617
NP_001518.3	histone deacetylase 2	HDAC2	3066	1.2	26	9	24	5	488	55.3	0.057135483
NP_077274.3	lysophospholipid acyltransferase 7 isoform 1	MBOAT7	79143	1.822	15	6	18	6	472	52.7	0.00042278
NP_705831.1	conserved oligomeric Golgi complex subunit 7	COG7	91949	0.877	15	8	20	8	770	86.3	0.450203405
NP_036243.1	activator of 90 kDa heat shock protein ATPase homolog 1 isoform 1	AHSA1	10598	0.709	19	5	12	5	338	38.3	0.417249394
XP_005247514.1	muscleblind-like protein 1 isoform X1	MBNL1	4154	1.056	13	5	24	2	431	46.5	0.004334579
NP_659477.2	caveolae-associated protein 3	CAVIN3	112464	1.11	18	5	21	5	261	27.7	0.014834295
XP_005271136.1	receptor-type tyrosine-protein phosphatase F isoform X1	PTPRF	5792	1.394	6	8	17	8	1922	214.3	0.052431353
XP_016874003.1	barrier-to-autointegration factor isoform X1	BANF1	8815	1.177	48	3	19	3	89	10.1	0.081238666
NP_053733.2	RNA-binding protein EWS isoform 1	EWSR1	2130	1.122	7	4	22	4	661	68.9	0.536332703
NP_079426.2	threonine--tRNA ligase, mitochondrial isoform a	TARS2	80222	1.493	17	9	23	9	718	81	0.088396126
NP_066972.1	coactosin-like protein	COTL1	23406	0.356	65	10	30	10	142	15.9	0.000152237
XP_024304747.1	rho GDP-dissociation inhibitor 2 isoform X1	ARHGDIB	397	0.445	35	4	22	4	201	23	2.80E-06
NP_001853.2	cytochrome c oxidase subunit 5B, mitochondrial precursor	COX5B	1329	1.309	36	6	22	6	129	13.7	0.101075241
XP_011508215.1	UDP-N-acetylhexosamine pyrophosphorylase isoform X1	UAP1	6675	0.568	18	7	16	7	522	58.7	0.002186642
NP_000382.3	tripeptidyl-peptidase 1 preproprotein	TPP1	1200	1.833	12	5	22	5	563	61.2	0.00497018
NP_001311404.1	integrator complex subunit 3	INTS3	65123	1.256	9	5	9	5	1042	117.9	0.489852379
NP_001307577.1	splicing factor U2AF 35 kDa subunit-like protein isoform 2	U2AF1L5	102724594	1.267	31	6	25	6	240	27.9	0.289921737
NP_002788.1	proteasome subunit beta type-5 isoform 1	PSMB5	5693	0.984	32	7	17	7	263	28.5	0.773204474
XP_005266355.1	sister chromatid cohesion protein PDS5 homolog B isoform X1	PDS5B	23047	0.967	8	10	24	8	1448	164.7	0.877249659
XP_011531009.1	proteasome activator complex subunit 4 isoform X3	PSME4	23198	1.014	7	7	14	7	1847	211.6	0.783553585
XP_011543857.1	ribosomal protein S6 kinase alpha-3 isoform X1	RPS6KA3	6197	1.01	16	10	17	6	746	84.4	0.87113061
NP_003748.1	eukaryotic translation initiation factor 3 subunit I	EIF3I	8668	0.726	36	8	29	8	325	36.5	0.138551858
NP_003127.1	signal recognition particle 54 kDa protein isoform 1	SRP54	6729	0.985	23	8	21	8	504	55.7	0.817905944
NP_008941.3	probable ATP-dependent RNA helicase DDX52 isoform 1	DDX52	11056	1.165	16	6	14	6	599	67.4	0.519870331
XP_006712741.1	mitochondrial chaperone BCS1 isoform X1	BCS1L	617	1.408	21	6	18	6	419	47.5	0.004430448
NP_000549.1	hemoglobin subunit alpha	HBA1	3039	1.714	35	5	24	5	142	15.2	8.02E-06
NP_065174.1	coronin-1B	CORO1B	57175	0.85	22	10	22	10	489	54.2	0.736653504
XP_005252153.1	volume-regulated anion channel subunit LRRC8A isoform X1	LRRC8A	56262	1.035	16	10	19	9	810	94.1	0.993513126
NP_004955.2	histone deacetylase 1	HDAC1	3065	1.268	23	8	21	4	482	55.1	0.203218585
XP_011514672.1	RNA-binding protein 28 isoform X1	RBM28	55131	1.471	14	9	17	9	793	89.5	0.080175763
NP_004956.5	non-histone chromosomal protein HMG-14	HMGN1	3150	1.587	23	4	12	4	100	10.7	0.44239003
NP_001020.2	40S ribosomal protein S26	RPS26	6231	0.769	44	4	33	4	115	13	0.127366492
NP_001258714.1	cytoplasmic dynein 1 intermediate chain 2 isoform 1	DYNC1I2	1781	0.627	17	7	21	7	638	71.4	0.007820049
NP_001019.1	40S ribosomal protein S25	RPS25	6230	0.531	30	5	36	5	125	13.7	0.06619747
NP_005944.1	metallothionein-2	MT2A	4502	0.198	54	3	18	1	61	6	0.146025751
NP_003090.2	sorting nexin-1 isoform a	SNX1	6642	0.705	12	5	13	4	522	59	0.406735179
NP_060289.2	tRNA methyltransferase 10 homolog C precursor	TRMT10C	54931	1.105	31	10	27	10	403	47.3	0.553816471
XP_016859273.1	SPATS2-like protein isoform X3	SPATS2L	26010	0.618	15	7	15	7	519	57.6	0.290105827
NP_653171.1	jupiter microtubule associated homolog 2	JPT2	90861	0.69	66	9	20	9	190	20.1	0.021116339
NP_006801.1	protein disulfide-isomerase A5 precursor	PDIA5	10954	1.933	14	8	22	8	519	59.6	0.000195268
XP_024301813.1	leucyl-cystinyl aminopeptidase isoform X1	LNPEP	4012	1.43	8	7	24	7	1025	117.3	0.001019322
NP_003749.2	eukaryotic translation initiation factor 3 subunit J isoform 1	EIF3J	8669	0.676	24	7	21	7	258	29	0.024741518
NP_056444.3	U4/U6 small nuclear ribonucleoprotein Prp31	PRPF31	26121	1.304	16	6	18	6	499	55.4	0.2229118
NP_001307540.1	importin subunit alpha-1	KPNA2	3838	0.555	21	7	24	7	529	57.8	0.011230625
XP_005258334.1	NPC intracellular cholesterol transporter 1 isoform X1	NPC1	4864	1.164	7	8	26	8	1303	144.8	0.053082686
NP_945353.1	rho guanine nucleotide exchange factor 1 isoform 1	ARHGEF1	9138	0.713	12	9	19	9	927	104	0.153120281
XP_024306481.1	COP9 signalosome complex subunit 1 isoform X1	GPS1	2873	0.793	15	6	20	6	617	68.1	0.019762625
NP_001305754.1	beta-hexosaminidase subunit alpha isoform 1 precursor	HEXA	3073	1.173	14	7	25	6	540	62	0.418072109
NP_006545.1	metaxin-2 isoform 1	MTX2	10651	1.393	37	6	19	6	263	29.7	0.015328586
NP_002865.1	UV excision repair protein RAD23 homolog B isoform 1	RAD23B	5887	0.737	20	8	27	8	409	43.1	0.000526078
NP_001342176.1	ubiquitin-conjugating enzyme E2 L5	UBE2L5	171222	0.841	63	6	16	2	154	17.9	0.689991347
XP_024305003.1	liprin-beta-1 isoform X4	PPFIBP1	8496	0.932	14	10	19	10	1025	115.4	0.833789434
NP_001026854.1	serine/arginine-rich splicing factor 7 isoform 1	SRSF7	6432	1.502	27	7	29	6	238	27.4	0.018635105
NP_005782.1	U3 small nucleolar ribonucleoprotein protein MPP10	MPHOSPH10	10199	1.479	17	8	20	8	681	78.8	0.06076257
NP_061980.1	myeloid-derived growth factor precursor	MYDGF	56005	1.851	33	6	22	6	173	18.8	8.10E-05
NP_001308969.1	nuclear transport factor 2	NUTF2	10204	0.443	51	4	25	4	127	14.5	0.005274754
XP_005268405.1	glucosamine-6-phosphate isomerase 1 isoform X1	GNPDA1	10007	0.374	21	4	20	3	318	36.1	0.00036975
XP_011513768.1	ras-related protein Ral-A isoform X1	RALA	5898	1.767	25	5	17	2	206	23.6	0.393628293
NP_005491.1	SUMO-activating enzyme subunit 1 isoform a	SAE1	10055	0.81	32	8	26	8	346	38.4	0.05893105
XP_016857298.1	guanine nucleotide-binding protein G(I)/G(S)/G(O) subunit gamma-12 isoform X1	GNG12	55970	1.722	46	3	16	3	72	8	0.010558754
XP_011527602.1	NSFL1 cofactor p47 isoform X1	NSFL1C	55968	0.875	26	6	14	6	375	41.2	0.84433876
NP_003760.1	serine/arginine-rich splicing factor 9	SRSF9	8683	1.75	38	9	36	9	221	25.5	0.002896071
NP_001020276.1	casein kinase I isoform alpha isoform 1	CSNK1A1	1452	1.068	19	6	21	6	365	41.9	0.66435789
NP_001265568.1	ran-specific GTPase-activating protein isoform 1	RANBP1	5902	0.303	31	6	24	6	278	31.9	0.000592908
NP_067000.1	U6 snRNA-associated Sm-like protein LSm2	LSM2	57819	0.69	67	4	13	4	95	10.8	0.30119576
NP_006800.2	mitochondrial import receptor subunit TOM34	TOMM34	10953	0.623	18	4	15	4	309	34.5	0.00150905
NP_003065.3	SWI/SNF complex subunit SMARCC1	SMARCC1	6599	1.38	7	5	12	2	1105	122.8	0.442166988
NP_036357.1	cytosolic Fe-S cluster assembly factor NUBP2 isoform 1	NUBP2	10101	0.681	30	4	15	4	271	28.8	0.100538152
NP_001311062.1	xaa-Pro aminopeptidase 1 isoform 6	XPNPEP1	7511	0.593	13	7	20	7	695	77.5	0.128266221
NP_001336851.1	60S ribosomal protein L27	RPL27	6155	0.897	54	8	33	7	136	15.8	0.176599581
XP_011531792.1	putative ATP-dependent RNA helicase DHX30 isoform X1	DHX30	22907	1.039	10	11	19	11	1265	140.4	0.828896223
NP_006362.1	cartilage-associated protein precursor	CRTAP	10491	1.489	19	6	20	6	401	46.5	0.153275099
NP_000928.1	DNA-directed RNA polymerase II subunit RPB1	POLR2A	5430	1.228	6	9	18	9	1970	217.1	0.441864197
NP_001258522.1	protein phosphatase methylesterase 1 isoform b	PPME1	51400	0.656	17	6	24	6	400	43.8	0.284336253
XP_016863889.1	septin-11 isoform X1	SEPT11	55752	0.867	17	6	27	5	439	50.8	0.689622284
XP_005269082.1	peripherin isoform X1	PRPH	5630	0.207	9	5	43	1	471	53.7	0.007823019
XP_011537270.1	formin-like protein 3 isoform X1	FMNL3	91010	1.107	6	5	20	2	1047	119.6	0.871888579
NP_001185709.1	actin-related protein 2/3 complex subunit 4 isoform c	ARPC4	10093	0.687	47	7	24	7	187	21.6	0.003187382
NP_065741.3	ANKHD1-EIF4EBP3 protein	ANKHD1-EIF4EBP3	404734	0.7	4	7	12	7	2617	277	0.324874736
NP_113673.3	glutamate-rich WD repeat-containing protein 1	GRWD1	83743	0.746	15	5	14	5	446	49.4	0.403072284
XP_024306246.1	mitochondrial Rho GTPase 2 isoform X1	RHOT2	89941	1.249	11	6	17	5	790	86.2	0.119800922
NP_001020114.1	argininosuccinate lyase isoform 1	ASL	435	0.884	16	6	17	6	464	51.6	0.553131997
NP_001168.1	ADP-ribosylation factor-like protein 1 isoform 1	ARL1	400	0.768	35	4	18	4	181	20.4	0.000724388
XP_016873261.1	double-strand break repair protein MRE11 isoform X1	MRE11	4361	0.921	13	8	24	8	708	80.5	0.566610961
NP_004810.2	symplekin	SYMPK	8189	0.696	6	6	18	6	1274	141.1	0.397810671
NP_060216.2	testis-expressed protein 10 isoform 1	TEX10	54881	0.691	7	4	13	4	929	105.6	0.133064302
NP_001306045.1	aspartyl aminopeptidase isoform a	DNPEP	23549	0.698	25	7	18	7	493	54.5	0.062692106
NP_005309.1	histone H1.0	H1F0	3005	2.029	20	5	36	5	194	20.9	0.014740924
XP_005270768.1	importin subunit alpha-7 isoform X1	KPNA6	23633	0.357	16	6	18	2	566	63	0.004481913
NP_036561.1	SEC14-like protein 2 isoform 1	SEC14L2	23541	0.604	26	7	26	7	403	46.1	0.000241251
NP_001188267.1	scaffold attachment factor B1 isoform 1	SAFB	6294	1.9	12	10	20	4	917	102.8	0.032459729
XP_005245570.1	acidic leucine-rich nuclear phosphoprotein 32 family member E isoform X1	ANP32E	81611	0.543	17	4	17	4	286	32.9	0.372405919
NP_001008389.1	CDGSH iron-sulfur domain-containing protein 2	CISD2	493856	1.072	42	5	17	5	135	15.3	0.524512739
NP_683720.2	proteasome subunit beta type-8 isoform E2 precursor	PSMB8	5696	0.635	25	5	16	5	276	30.3	0.279948864
NP_001257327.1	inactive tyrosine-protein kinase 7 isoform e	PTK7	5754	1.247	7	6	17	6	1078	119.1	0.040212467
NP_000973.2	60S ribosomal protein L21	RPL21	6144	0.799	26	6	27	6	160	18.6	0.028621871
NP_006322.4	ribosomal RNA small subunit methyltransferase NEP1 isoform 1	EMG1	10436	1.012	32	6	14	6	244	26.7	0.830207311
NP_001122321.1	transcription activator BRG1 isoform A	SMARCA4	6597	1.336	6	9	21	9	1679	188	0.193316197
NP_001123996.1	AP-1 complex subunit mu-1 isoform 1	AP1M1	8907	0.69	22	7	14	7	435	49.8	0.352487158
NP_689953.1	3-hydroxyisobutyrate dehydrogenase, mitochondrial precursor	HIBADH	11112	1.24	24	5	12	5	336	35.3	0.844559466
NP_619579.1	cofilin-2 isoform 1	CFL2	1073	0.656	30	4	30	1	166	18.7	0.086889216
NP_002347.5	myristoylated alanine-rich C-kinase substrate	MARCKS	4082	1.243	32	6	20	6	332	31.5	0.207150709
NP_036585.1	transducin beta-like protein 2 isoform 1 precursor	TBL2	26608	1.402	15	6	17	6	447	49.8	0.158675354
NP_000535.3	antigen peptide transporter 2 isoform 1	TAP2	6891	1.208	14	7	17	7	703	77.6	0.519552688
NP_057439.2	probable ATP-dependent RNA helicase DDX47 isoform 1	DDX47	51202	1.43	17	6	20	6	455	50.6	0.331762015
NP_060654.1	ADP-ribosylation factor-like protein 8B	ARL8B	55207	1.252	37	6	19	6	186	21.5	0.003015816
XP_016858330.1	vesicle-associated membrane protein 3 isoform X1	VAMP3	9341	1.324	41	5	17	5	122	13.8	0.593019524
NP_004584.1	transformer-2 protein homolog beta isoform 1	TRA2B	6434	1.428	23	5	24	5	288	33.6	0.16453172
NP_000593.1	plasminogen activator inhibitor 1 precursor	SERPINE1	5054	1.727	15	5	20	5	402	45	0.09083499
NP_001350420.1	zinc finger CCCH-type antiviral protein 1 isoform 3	ZC3HAV1	56829	1.057	9	7	17	7	1024	114	0.704849872
NP_079100.2	zinc finger CCCH domain-containing protein 14 isoform 1	ZC3H14	79882	1.065	11	6	17	6	736	82.8	0.779576591
XP_024302603.1	acylglycerol kinase, mitochondrial isoform X1	AGK	55750	1.391	17	7	19	7	422	47.1	0.028531875
XP_005265738.1	G-rich sequence factor 1 isoform X1	GRSF1	2926	1.28	17	6	22	6	561	61.5	0.097757783
NP_002736.3	mitogen-activated protein kinase 1	MAPK1	5594	0.66	25	8	29	5	360	41.4	0.002118435
XP_005274380.1	phosphatidylinositol-binding clathrin assembly protein isoform X2	PICALM	8301	1.019	19	7	20	7	681	73.7	0.946179981
NP_116219.2	U3 small nucleolar RNA-associated protein 4 homolog isoform 1	UTP4	84916	1.383	8	5	19	5	686	76.8	0.279897263
NP_001760.1	CD9 antigen isoform 1	CD9	928	1.454	21	4	17	4	228	25.4	0.051691526
XP_011513028.1	leucine-rich repeat-containing protein 1 isoform X1	LRRC1	55227	1.148	19	6	19	5	524	59.2	0.344272558
NP_004420.1	ephrin-B1 precursor	EFNB1	1947	1.679	29	6	19	6	346	38	0.007817025
NP_006612.2	S-adenosylhomocysteine hydrolase-like protein 1 isoform a	AHCYL1	10768	1.327	15	9	29	7	530	58.9	0.223312744
NP_001306000.1	phosphatidylinositide phosphatase SAC1 isoform 1x	SACM1L	22908	1.494	14	8	23	8	609	69.4	0.00076339
NP_001743.1	catalase	CAT	847	1.851	20	10	22	10	527	59.7	0.203943028
NP_777549.1	NADH dehydrogenase	NDUFAF2	91942	1.458	41	6	24	6	169	19.8	0.075803965
NP_056046.1	nuclear pore complex protein Nup160 isoform 1	NUP160	23279	1.258	7	8	23	8	1436	162	0.099357323

Patil et al,2020. Proteomic and phosphoproteomic profiling of shammah-induced signaling in oral keratinocytes

Supplementary Table S1: List of proteins identified and quantified in OKF6/TERT1-Shammah and OKF6/TERT1-Parental total proteome using TMT-based quantitative proteomic approach

Accession	Protein Description	Gene Symbol	Gene ID	Fold Change	Coverage [%]	# Peptides	# PSMs	# Unique Peptides	# AAs	MW [kDa]	p-value
NP_115727.5	39S ribosomal protein L45, mitochondrial isoform 1	MRPL45	84311	1.386	34	7	19	7	306	35.3	0.538719158
NP_002703.1	protein phosphatase 1 regulatory subunit 7 isoform 1	PPP1R7	5510	0.689	22	8	31	8	360	41.5	0.263817912
XP_006724166.1	nuclear pore complex protein Nup50 isoform X1	NUP50	10762	1.016	20	6	16	6	468	50.1	0.900860777
NP_057154.2	28S ribosomal protein S23, mitochondrial	MRPS23	51649	1.213	26	6	22	6	190	21.8	0.031193768
XP_005273221.1	plexin-A2 isoform X1	PLXNA2	5362	0.979	7	9	20	6	1974	219.5	0.729001532
XP_005262538.1	cullin-4B isoform X1	CUL4B	8450	1.118	11	9	22	6	915	104.1	0.574383496
NP_001334633.1	THO complex subunit 6 homolog isoform 1	THOC6	79228	1.195	28	6	21	6	341	37.5	0.3369599
NP_004699.1	programmed cell death protein 5	PDCD5	9141	0.615	49	7	20	7	125	14.3	0.010090693
NP_057152.2	mitochondrial fission 1 protein	FIS1	51024	1.678	16	3	14	3	152	16.9	0.000674272
XP_006713723.1	plastin-1 isoform X1	PLS1	5357	0.73	13	6	24	3	629	70.2	0.233076726
NP_057040.2	39S ribosomal protein L4, mitochondrial isoform a	MRPL4	51073	1.228	27	6	19	6	311	34.9	0.004716706
NP_004986.1	matrix metalloproteinase-14 preproprotein	MMP14	4323	1.911	17	9	24	9	582	65.9	0.002540578
NP_858058.1	UDP-N-acetylglucosamine--peptide N-acetylglucosaminyltransferase 110 kDa subunit isoform 1	OGT	8473	0.792	10	6	11	6	1046	116.9	0.47745937
NP_055484.3	nuclear pore complex protein Nup93 isoform 1	NUP93	9688	0.946	12	7	20	7	819	93.4	0.760274522
NP_001317217.1	SWI/SNF complex subunit SMARCC2 isoform d	SMARCC2	6601	1.787	8	7	13	4	1245	136.1	0.255429279
NP_003115.1	sepiapterin reductase	SPR	6697	0.908	26	5	21	5	261	28	0.843245646
NP_001020419.1	deoxyuridine 5'-triphosphate nucleotidohydrolase, mitochondrial isoform 1 precursor	DUT	1854	0.553	20	4	16	4	252	26.5	0.005798999
NP_006353.2	nuclear RNA export factor 1 isoform 1	NXF1	10482	1.129	15	7	20	7	619	70.1	0.001425568
XP_011533736.1	collagen alpha-1(XII) chain isoform X1	COL12A1	1303	1.505	5	9	19	9	3118	339.4	0.625520214
NP_059980.2	transmembrane emp24 domain-containing protein 9 precursor	TMED9	54732	1.944	36	7	18	7	235	27.3	0.004409079
XP_011528773.1	MICAL-like protein 1 isoform X1	MICALL1	85377	0.842	12	7	16	7	929	100.3	0.40273771
XP_006715771.1	basic leucine zipper and W2 domain-containing protein 2 isoform X1	BZW2	28969	0.606	20	7	18	6	419	48.1	0.032784045
NP_068817.1	protein transport protein Sec24A isoform 1	SEC24A	10802	0.659	7	6	22	5	1093	119.7	0.061800579
XP_016874205.1	prostaglandin E synthase 3 isoform X1	PTGES3	10728	0.508	35	5	21	5	165	19.4	0.02098475
NP_003964.3	60S ribosomal protein L14	RPL14	9045	0.712	25	5	34	5	215	23.4	0.025057
NP_004422.2	ephrin type-A receptor 2 isoform 1 precursor	EPHA2	1969	1.066	8	8	23	8	976	108.2	0.688566585
NP_001116428.1	drebrin-like protein isoform c	DBNL	28988	0.711	14	5	19	5	439	49	0.362641153
NP_001129675.1	coiled-coil domain-containing protein 124	CCDC124	115098	1.046	19	4	15	4	223	25.8	0.582456422
XP_006711906.1	centrosomal protein of 170 kDa isoform X1	CEP170	9859	0.769	7	11	18	11	1656	183.2	0.40312332
NP_037379.1	serine protease HTRA2, mitochondrial isoform 1 preproprotein	HTRA2	27429	1.536	16	5	18	5	458	48.8	0.058715949
NP_002755.1	ribose-phosphate pyrophosphokinase 1 isoform 1	PRPS1	5631	0.641	33	7	23	2	318	34.8	0.567783003
XP_016859146.1	CLIP-associating protein 1 isoform X1	CLASP1	23332	0.936	6	7	15	1	1588	174.7	0.541223412
XP_016862117.1	phospholipid scramblase 1 isoform X1	PLSCR1	5359	0.721	21	5	13	5	319	35.1	0.422765931
XP_011523281.1	nuclear protein localization protein 4 homolog isoform X1	NPLOC4	55666	0.852	12	4	15	4	617	69.4	0.457862331
XP_005256556.1	developmentally-regulated GTP-binding protein 2 isoform X1	DRG2	1819	0.502	15	5	17	5	385	42.9	0.036303496
NP_056399.1	polymerase delta-interacting protein 2 isoform 1	POLDIP2	26073	1.182	22	5	17	5	368	42	0.210159513
NP_110382.3	thioredoxin-related transmembrane protein 1 precursor	TMX1	81542	1.317	20	6	17	6	280	31.8	0.147023007
NP_056229.2	60S ribosomal protein L36	RPL36	25873	0.669	30	4	24	4	105	12.2	0.059383851
XP_024309484.1	fibronectin type III domain-containing protein 3B isoform X1	FNDC3B	64778	1.436	7	5	14	5	1204	132.8	0.10686879
NP_005902.1	S-adenosylmethionine synthase isoform type 2	MAT2A	4144	0.485	19	8	25	8	395	43.6	0.02321038
NP_055814.1	ras-related protein Rab-21	RAB21	23011	0.908	28	6	19	6	225	24.3	0.752642167
NP_705841.2	lysophospholipid acyltransferase LPCAT4	LPCAT4	254531	1.12	13	5	23	5	524	57.2	0.349307227
NP_037377.1	vacuolar protein sorting-associated protein 4A	VPS4A	27183	0.699	17	9	26	4	437	48.9	0.021962733
NP_001248763.1	FAST kinase domain-containing protein 4 isoform 3	TBRG4	9238	1.658	17	7	23	7	642	71.8	0.382596497
XP_016882478.1	dual specificity mitogen-activated protein kinase kinase 2 isoform X1	MAP2K2	5605	0.485	17	7	21	3	512	56.3	0.091310265
NP_001654.1	ADP-ribosylation factor 6	ARF6	382	1.014	25	3	16	3	175	20.1	0.919438861
NP_002516.2	nardilysin isoform a	NRDC	4898	0.629	6	7	11	7	1219	139.3	0.042908014
NP_998785.1	V-type proton ATPase subunit H isoform 1	ATP6V1H	51606	0.797	19	7	15	7	483	55.8	0.758033333
NP_000301.1	palmitoyl-protein thioesterase 1 isoform 1 precursor	PPT1	5538	1.555	20	5	18	5	306	34.2	0.070052293
NP_002060.4	guanine nucleotide-binding protein G(i) subunit alpha-1 isoform 1	GNAI1	2770	1.542	21	7	34	2	354	40.3	0.943093991
NP_060695.2	WD40 repeat-containing protein SMU1	SMU1	55234	1.193	10	6	19	6	513	57.5	0.164484036
NP_002058.2	guanine nucleotide-binding protein subunit alpha-11	GNA11	2767	1.494	20	7	22	4	359	42.1	0.754382621
NP_001305771.1	protein canopy homolog 3 isoform 3 precursor	CNPY3	10695	1.723	23	7	20	7	311	34.1	0.021449128
NP_006507.2	solute carrier family 2, facilitated glucose transporter member 1	SLC2A1	6513	1.578	7	5	13	5	492	54	0.166100846
XP_005270320.1	sphingosine-1-phosphate lyase 1 isoform X1	SGPL1	8879	1.431	11	6	22	6	568	63.5	0.004123293
XP_016864062.1	toll-like receptor 2 isoform X1	TLR2	7097	1.326	12	8	22	8	784	89.8	0.151248417
NP_000929.1	DNA-directed RNA polymerase II subunit RPB2 isoform 1	POLR2B	5431	1.063	9	10	21	10	1174	133.8	0.977916376
NP_003960.1	NEDD8-conjugating enzyme Ubc12	UBE2M	9040	0.739	32	5	21	5	183	20.9	0.007164155
NP_003085.1	small nuclear ribonucleoprotein E isoform 1	SNRPE	6635	0.899	52	3	20	3	92	10.8	0.680293398
NP_006065.2	E3 ubiquitin-protein ligase TRIM22 isoform 1	TRIM22	10346	0.798	20	7	19	7	498	56.9	0.036803061
NP_005434.4	bifunctional 3'-phosphoadenosine 5'-phosphosulfate synthase 1	PAPSS1	9061	0.386	14	6	16	5	624	70.8	0.030101067
NP_037415.1	methionine adenosyltransferase 2 subunit beta isoform 1	MAT2B	27430	0.589	26	7	18	7	334	37.5	0.149106534
NP_055070.1	protein canopy homolog 2 isoform 1 precursor	CNPY2	10330	2.107	23	3	16	3	182	20.6	3.41E-05
NP_066954.2	serine/threonine-protein phosphatase 2A activator isoform b	PTPA	5524	0.602	20	5	18	5	323	36.8	0.015882875
NP_055969.1	LRP chaperone MESD precursor	MESD	23184	1.311	25	6	16	6	234	26.1	0.113681933
NP_004805.2	U5 small nuclear ribonucleoprotein 40 kDa protein	SNRNP40	9410	1.171	29	7	19	7	357	39.3	0.621791754
NP_115766.3	MKI67 FHA domain-interacting nucleolar phosphoprotein	NIFK	84365	1.039	19	5	14	5	293	34.2	0.995661141
NP_055415.1	EH domain-containing protein 3	EHD3	30845	0.766	18	8	14	1	535	60.8	0.014794323
XP_016884588.1	GTP-binding protein 1 isoform X1	GTPBP1	9567	0.695	9	5	16	5	734	79.6	0.684247086
NP_003156.1	syntaxin-binding protein 1 isoform a	STXBP1	6812	0.79	10	5	15	5	603	68.7	0.571741858
NP_001317.1	cleavage stimulation factor subunit 3 isoform 1	CSTF3	1479	0.789	14	7	18	7	717	82.9	0.45604572
NP_942130.1	serpin B8 isoform a	SERPINB8	5271	0.44	18	7	20	5	374	42.7	0.334634194
NP_001209.1	carbonic anhydrase 12 isoform 1 precursor	CA12	771	1.979	25	5	23	5	354	39.4	0.000583573
NP_037366.1	eukaryotic translation initiation factor 3 subunit K isoform 1	EIF3K	27335	0.843	20	4	16	4	218	25	0.378451627
NP_037468.1	protein transport protein Sec61 subunit alpha isoform 1	SEC61A1	29927	1.982	14	5	21	5	476	52.2	0.001741829
NP_060238.3	leucine-rich repeat-containing protein 40	LRRC40	55631	0.876	15	8	20	8	602	68.2	0.396874077
NP_001124384.1	protein SGT1 homolog isoform B	SUGT1	10910	0.729	27	6	17	6	365	41	0.074290052
NP_065761.1	leucine-rich repeat-containing protein 47	LRRC47	57470	0.646	12	5	14	5	583	63.4	0.100451169
NP_001171501.1	splicing factor 1 isoform 6	SF1	7536	1.327	14	6	20	6	673	71.7	0.006585277
NP_054745.1	m7GpppX diphosphatase isoform 2	DCPS	28960	1.055	27	6	14	6	337	38.6	0.912950205
NP_071381.1	xaa-Pro aminopeptidase 3 isoform 1	XPNPEP3	63929	1.21	12	4	20	3	507	57	0.284494151
NP_002826.3	tyrosine-protein phosphatase non-receptor type 12 isoform 1	PTPN12	5782	0.799	12	7	16	7	780	88.1	0.154708517
NP_003553.2	mitochondrial 2-oxoglutarate/malate carrier protein isoform 1	SLC25A11	8402	0.933	30	8	30	8	314	34	0.215652716
NP_000935.1	serine/threonine-protein phosphatase 2B catalytic subunit alpha isoform isoform 1	PPP3CA	5530	0.881	19	9	21	9	521	58.7	0.614052973
XP_024307187.1	phenylalanine--tRNA ligase alpha subunit isoform X1	FARSA	2193	0.616	12	6	18	6	569	64.2	0.215300297
NP_065108.1	fructose-2,6-bisphosphatase TIGAR	TIGAR	57103	0.419	33	6	15	6	270	30	0.009646831
XP_024305248.1	cysteine-rich protein 2 isoform X1	CRIP2	1397	0.748	29	5	15	5	280	30	0.431809201
NP_055958.2	methionine aminopeptidase 1	METAP1	23173	0.663	16	4	10	4	386	43.2	0.027120526
NP_001001520.1	hepatoma-derived growth factor-related protein 2 isoform 1	HDGFL2	84717	1.387	6	6	19	4	671	74.3	0.604247039
NP_002084.2	glycogen synthase kinase-3 beta isoform 1	GSK3B	2932	0.611	18	4	15	4	433	48	0.005278736
XP_011512612.2	mitogen-activated protein kinase 14 isoform X1	MAPK14	1432	0.548	17	5	15	4	401	45.4	0.179497441
XP_024308498.1	fumarylacetoacetate hydrolase domain-containing protein 2B isoform X4	FAHD2B	151313	0.841	25	6	19	1	314	34.6	0.488279866
XP_011522496.1	brain-specific angiogenesis inhibitor 1-associated protein 2 isoform X1	BAIAP2	10458	0.775	13	6	17	6	568	63	0.080476674
NP_057370.1	L-xylulose reductase isoform 1	DCXR	51181	1.709	27	5	22	5	244	25.9	0.045965429
NP_001138986.1	secernin-1 isoform b	SCRN1	9805	0.338	10	3	23	3	434	48.7	0.001299771
NP_006563.2	guanine nucleotide-binding protein subunit alpha-13 isoform 1	GNA13	10672	1.326	12	4	30	3	377	44	0.001856213
NP_001316166.1	serine/threonine-protein kinase Nek9 isoform 1	NEK9	91754	0.751	7	5	6	5	991	108.4	0.864935714
NP_778238.1	keratin, type II cytoskeletal 73	KRT73	319101	1.209	6	5	68	1	540	58.9	0.480137485
XP_011522203.1	platelet-activating factor acetylhydrolase IB subunit alpha isoform X1	PAFAH1B1	5048	0.545	21	8	25	7	428	48.7	0.01620404
XP_005249072.1	guanine nucleotide-binding protein-like 1 isoform X1	GNL1	2794	0.784	10	4	16	4	607	68.6	0.429694516
XP_005263532.2	exosome component 10 isoform X1	EXOSC10	5394	1.134	13	10	19	10	890	101.3	0.666338696
NP_001025180.1	40S ribosomal protein S15a	RPS15A	6210	0.667	31	4	23	4	130	14.8	0.002905585
NP_002757.2	phosphoribosyl pyrophosphate synthase-associated protein 1 isoform 1	PRPSAP1	5635	0.813	17	5	11	3	385	42.4	0.766653737
XP_005271395.1	serine/arginine-rich splicing factor 11 isoform X1	SRSF11	9295	1.337	8	3	15	3	491	54.3	0.004994064
NP_061924.1	39S ribosomal protein L50, mitochondrial	MRPL50	54534	1.164	48	5	14	5	158	18.3	0.102178215
NP_001309424.1	glutathione synthetase	GSS	2937	0.453	20	10	26	10	474	52.4	0.002375899
XP_005268461.1	la-related protein 1 isoform X1	LARP1	23367	0.625	8	9	22	9	1096	123.4	0.119656771
NP_056986.2	E3 ubiquitin-protein ligase UBR5 isoform 1	UBR5	51366	1.231	3	8	14	8	2799	309.2	0.757237233
XP_011517288.1	coronin-2A isoform X1	CORO2A	7464	1.527	16	7	21	7	525	59.7	0.010095259
NP_002408.3	proliferation marker protein Ki-67 isoform 1	MKI67	4288	0.865	3	6	17	6	3256	358.5	0.388872256
XP_016868591.1	cytochrome c1, heme protein, mitochondrial isoform X1	CYC1	1537	1.419	28	5	16	5	357	38	0.014206053
NP_055521.1	squamous cell carcinoma antigen recognized by T-cells 3	SART3	9733	1.258	13	10	18	10	963	109.9	0.707083669
NP_872578.1	28S ribosomal protein S9, mitochondrial	MRPS9	64965	1.254	15	7	23	7	396	45.8	0.175108749
NP_006824.2	COP9 signalosome complex subunit 6	COPS6	10980	0.717	28	7	18	7	327	36.1	0.021678867
XP_011508164.1	protein S100-A13 isoform X1	S100A13	6284	0.372	49	6	21	6	98	11.5	0.000423745
NP_003879.2	retinal dehydrogenase 2 isoform 1	ALDH1A2	8854	1.222	14	7	30	3	518	56.7	0.646890228
NP_001152862.1	guanylate kinase isoform a	GUK1	2987	1.07	41	6	14	6	218	23.8	0.518246839
NP_036548.1	ran-binding protein 6 isoform 1	RANBP6	26953	1.017	6	5	15	2	1105	124.6	0.989194036
NP_071451.2	interferon-induced helicase C domain-containing protein 1	IFIH1	64135	0.51	8	6	15	6	1025	116.6	0.092930645
NP_001618.2	CD166 antigen isoform 1 precursor	ALCAM	214	1.244	11	7	18	7	583	65.1	0.40466531
NP_056016.1	ribosome biogenesis protein BOP1	BOP1	23246	1.576	14	7	19	7	746	83.6	0.117075605
NP_001474.1	protein NipSnap homolog 2 isoform 1	NIPSNAP2	2631	1.878	18	6	19	4	286	33.7	0.000476257
NP_060700.2	nuclear pore complex protein Nup133	NUP133	55746	1.159	10	8	14	8	1156	128.9	0.848957424
XP_011539919.1	filamin-binding LIM protein 1 isoform X2	FBLIM1	54751	0.69	25	7	13	7	373	40.6	0.057115219
XP_016871767.1	nuclear factor NF-kappa-B p100 subunit isoform X1	NFKB2	4791	0.686	9	5	12	5	1081	115.8	0.06049761
NP_056146.1	nicastrin isoform 1 precursor	NCSTN	23385	0.819	6	3	12	3	709	78.4	0.040952064
NP_005683.2	ATP-binding cassette sub-family F member 2 isoform b	ABCF2	10061	0.745	10	5	14	5	634	72.4	0.352123038
NP_006378.3	calcium homeostasis endoplasmic reticulum protein	CHERP	10523	1.069	8	5	11	5	916	103.6	0.687250726
NP_002328.1	alpha-2-macroglobulin receptor-associated protein precursor	LRPAP1	4043	2.484	18	7	24	7	357	41.4	0.001942213
NP_001171488.1	acyl-CoA-binding protein isoform 4	DBI	1622	0.332	24	4	17	4	148	16.5	0.004904007
NP_001191813.1	ras-related protein Rab-43 isoform a	RAB43	339122	0.95	17	4	26	3	212	23.3	0.830843889
XP_011526206.1	DAZ-associated protein 1 isoform X1	DAZAP1	26528	1.065	15	6	14	6	550	59	0.770411943
XP_006720071.1	fermitin family homolog 2 isoform X1	FERMT2	10979	1.037	7	4	18	2	706	80.7	0.936717493
NP_001268231.1	ribosome-releasing factor 2, mitochondrial isoform 4	GFM2	84340	1.041	11	6	13	6	811	90.4	0.844294145
NP_001185808.1	ATP5J2-PTCD1 fusion protein	ATP5MF-PTCD1	100526740	0.902	5	3	16	3	749	84.1	0.464850101
XP_011518671.1	mitochondrial glutamate carrier 1 isoform X1	SLC25A22	79751	1.188	23	6	22	6	323	34.4	0.203086965
NP_003642.3	Y-box-binding protein 3 isoform a	YBX3	8531	1.058	20	5	18	3	372	40.1	0.947286835
NP_003240.1	thimet oligopeptidase	THOP1	7064	0.602	12	7	18	7	689	78.8	0.010956393
NP_001830.1	calponin-3 isoform 1	CNN3	1266	0.751	19	5	14	4	329	36.4	0.115769983
NP_068593.2	28S ribosomal protein S35, mitochondrial isoform 1 precursor	MRPS35	60488	1.53	27	6	21	6	323	36.8	0.015584848
NP_001131025.1	leucine-rich repeat flightless-interacting protein 1 isoform 5	LRRFIP1	9208	0.754	11	6	14	6	752	82.6	0.082321753
NP_001244127.1	cullin-3 isoform 3	CUL3	8452	1.16	10	7	16	7	774	89.7	0.443529478
NP_001026866.1	phospholipase D3	PLD3	23646	2.354	12	4	22	4	490	54.7	1.77E-05
NP_057479.2	very-long-chain (3R)-3-hydroxyacyl-CoA dehydratase 3	HACD3	51495	0.911	13	3	16	3	362	43.1	0.760609393
NP_001257817.1	mitochondrial dicarboxylate carrier isoform 1	SLC25A10	1468	1.17	22	4	12	4	296	32.1	0.491600594
NP_001164014.1	serine/threonine-protein phosphatase PGAM5, mitochondrial isoform 1	PGAM5	192111	1.86	25	7	21	7	289	32	0.007377902
NP_060791.3	ribosome biogenesis protein BRX1 homolog	BRIX1	55299	1.022	24	6	20	6	353	41.4	0.893657698
NP_004730.2	metastasis-associated protein MTA2 isoform 1	MTA2	9219	1.452	12	7	18	7	668	75	0.037964088
NP_004613.1	translin isoform 1	TSN	7247	0.689	30	5	15	5	228	26.2	0.009665087
XP_011526707.1	dipeptidyl peptidase 9 isoform X1	DPP9	91039	0.704	8	7	18	7	946	107.5	0.084168215
XP_024307075.1	SURP and G-patch domain-containing protein 2 isoform X1	SUGP2	10147	1.507	9	6	15	6	1106	122.6	0.329448261
NP_976074.1	CD59 glycoprotein preproprotein	CD59	966	3.005	26	4	24	4	128	14.2	3.33E-06
XP_011513776.1	biliverdin reductase A isoform X1	BLVRA	644	0.438	18	6	13	6	296	33.4	0.010855058
XP_011517485.1	ribosome-recycling factor, mitochondrial isoform X1	MRRF	92399	1.116	31	6	19	6	283	31.5	0.804509613
NP_000111.1	epoxide hydrolase 1	EPHX1	2052	1.222	14	5	14	5	455	52.9	0.458440678
XP_011515392.1	regulator of microtubule dynamics protein 1 isoform X1	RMDN1	51115	0.945	17	6	22	6	386	44	0.539032662
NP_001291278.1	cAMP-dependent protein kinase catalytic subunit alpha isoform 3	PRKACA	5566	0.616	14	5	23	1	427	49.1	0.015833738
NP_064696.1	microsomal glutathione S-transferase 1 isoform a	MGST1	4257	1.307	13	4	12	4	155	17.6	0.185399794
NP_060941.2	zinc finger CCCH domain-containing protein 15	ZC3H15	55854	0.728	14	5	16	5	426	48.6	0.005818611
XP_016872371.1	calcium uniporter protein, mitochondrial isoform X1	MCU	90550	1.664	16	5	13	5	358	41.5	0.001278522
XP_011536676.1	protein phosphatase 1 regulatory subunit 12A isoform X1	PPP1R12A	4659	0.76	10	8	12	8	1069	119.5	0.158321886
NP_001308286.1	nuclear receptor-binding protein isoform 1	NRBP1	29959	0.539	10	4	15	4	535	59.8	0.01886157
NP_076983.1	nucleolar complex protein 4 homolog	NOC4L	79050	0.932	15	5	17	5	516	58.4	0.756666054
NP_060308.1	H/ACA ribonucleoprotein complex subunit 2 isoform a	NHP2	55651	1.611	38	6	16	6	153	17.2	0.000652846
NP_005848.2	CAAX prenyl protease 1 homolog	ZMPSTE24	10269	1.258	16	6	17	6	475	54.8	0.036837266
XP_011509100.1	four and a half LIM domains protein 2 isoform X1	FHL2	2274	1.101	32	8	17	8	279	32.2	0.936269807
XP_011516801.1	GTPase-activating protein and VPS9 domain-containing protein 1 isoform X1	GAPVD1	26130	0.78	6	6	15	6	1487	166.1	0.002307054
NP_008986.2	DNA-directed RNA polymerase III subunit RPC1	POLR3A	11128	1.103	6	6	13	5	1390	155.5	0.584879659
NP_066403.2	histone H3.2	HIST2H3C	126961	0.508	58	8	73	1	136	15.4	0.268613771
NP_006127.1	F-actin-capping protein subunit alpha-2	CAPZA2	830	0.757	30	6	15	3	286	32.9	0.533773604
XP_011541005.1	pleckstrin homology-like domain family B member 1 isoform X1	PHLDB1	23187	0.954	5	6	15	6	1586	173.3	0.700068623
NP_005460.2	acyl-coenzyme A thioesterase 8	ACOT8	10005	1.749	21	4	16	4	319	35.9	0.138290457
NP_055422.1	UBX domain-containing protein 4	UBXN4	23190	1.667	11	4	17	4	508	56.7	0.011794034
NP_078805.3	parafibromin	CDC73	79577	1.143	15	7	26	7	531	60.5	0.395276673
NP_001231513.1	C7orf55-LUC7L2 protein	C7orf55-LUC7L2	100996928	0.838	11	5	21	5	458	54.2	0.492262369
NP_009200.2	syntaxin-binding protein 3	STXBP3	6814	1.733	13	7	20	7	592	67.7	0.047088024
XP_016855583.1	nuclear migration protein nudC isoform X1	NUDC	10726	0.414	15	7	24	7	392	45.2	0.000164959
NP_037390.2	apoptosis-associated speck-like protein containing a CARD isoform a	PYCARD	29108	0.435	26	5	14	5	195	21.6	0.004464869
NP_005508.1	non-histone chromosomal protein HMG-17	HMGN2	3151	2.66	46	3	12	3	90	9.4	0.158766362
NP_001124295.1	DNA (cytosine-5)-methyltransferase 1 isoform a	DNMT1	1786	0.917	5	6	15	6	1632	184.7	0.244387116
NP_060943.1	acyl-coenzyme A thioesterase 13 isoform 1	ACOT13	55856	2.1	29	3	23	3	140	15	2.78E-06
NP_001317366.1	tyrosine-protein phosphatase non-receptor type 11 isoform 3	PTPN11	5781	0.599	9	5	14	5	597	68.4	0.030864136
NP_954673.1	TMEM189-UBE2V1 fusion protein	TMEM189-UBE2V1	387522	0.567	19	8	23	4	370	42.2	0.011051493
XP_011536034.1	cyclin-dependent kinase 2 isoform X1	CDK2	1017	0.911	22	6	22	4	346	39.2	0.829924345
NP_071732.1	ras-related protein Rab-38	RAB38	23682	0.832	27	5	12	3	211	23.7	0.533620791
NP_112240.1	actin-related protein 2/3 complex subunit 5-like protein	ARPC5L	81873	0.733	58	5	13	5	153	16.9	0.210865207
NP_612453.1	BTB/POZ domain-containing protein KCTD12	KCTD12	115207	0.522	30	7	22	7	325	35.7	0.001591365
XP_011508832.1	formin-like protein 2 isoform X1	FMNL2	114793	0.979	4	4	18	1	1099	124.8	0.984839391
NP_003746.2	eukaryotic translation initiation factor 3 subunit G	EIF3G	8666	0.841	25	6	15	6	320	35.6	0.045815299
NP_002887.2	RNA-binding protein 4 isoform 1	RBM4	5936	1.255	17	7	15	7	364	40.3	0.238017981
XP_024306826.1	1-phosphatidylinositol 4,5-bisphosphate phosphodiesterase eta-2 isoform X1	PLCH2	9651	0.944	5	8	14	7	1755	189.2	0.926277587
XP_005260057.1	dedicator of cytokinesis protein 6 isoform X6	DOCK6	57572	0.862	5	6	16	6	2113	236.3	0.284210927
NP_085125.1	cytochrome P450 2S1 precursor	CYP2S1	29785	1.412	9	4	20	4	504	55.8	0.032625403
NP_055118.1	pescadillo homolog isoform 1	PES1	23481	1.151	11	6	14	6	588	68	0.807658818
NP_055946.1	WD repeat-containing protein 43	WDR43	23160	1.338	22	9	21	9	677	74.8	0.003166487
NP_002813.3	twinfilin-1 isoform 2	TWF1	5756	1.001	22	6	18	5	350	40.3	0.685768684
NP_060640.2	regulation of nuclear pre-mRNA domain-containing protein 1A isoform 1	RPRD1A	55197	0.93	19	6	14	4	312	35.7	0.80490957
NP_001350823.1	alpha-ketoglutarate-dependent dioxygenase FTO isoform 1	FTO	79068	0.703	12	5	20	5	526	60.7	0.553421282
NP_001910.2	enoyl-CoA delta isomerase 1, mitochondrial isoform 1 precursor	ECI1	1632	0.997	26	5	17	5	302	32.8	0.802692974
NP_996667.2	enoyl-CoA delta isomerase 2, mitochondrial isoform 2	ECI2	10455	1.27	14	5	13	5	394	43.6	0.16425053
XP_005247877.1	poly	PARP9	83666	0.485	11	8	24	8	854	96.3	0.032876388
NP_079422.1	N-alpha-acetyltransferase 50 isoform 1	NAA50	80218	0.648	40	6	13	6	169	19.4	0.029364339
NP_001278357.1	apoptosis regulator BAX isoform 1	BAX	581	0.755	35	6	21	6	221	24.3	0.404128282
NP_061895.3	protein disulfide-isomerase TMX3 isoform 1 precursor	TMX3	54495	1.676	11	5	20	5	454	51.8	0.009622823
NP_001350626.1	60S ribosomal protein L28 isoform 6	RPL28	6158	0.791	21	5	17	5	170	19.1	0.41082268
NP_003730.4	aldo-keto reductase family 1 member C3 isoform 1	AKR1C3	8644	0.533	23	6	23	2	323	36.8	0.009153238
NP_006419.2	39S ribosomal protein L28, mitochondrial	MRPL28	10573	1.646	27	5	19	5	256	30.1	0.252642694
NP_003121.1	sorcin isoform A	SRI	6717	0.736	32	5	17	5	198	21.7	0.164354523
XP_005272318.1	C-terminal-binding protein 1 isoform X1	CTBP1	1487	1.275	20	9	26	5	441	47.6	0.258511111
NP_001258766.1	reticulocalbin-2 isoform b precursor	RCN2	5955	2.583	19	6	14	6	335	39.1	0.000815411
NP_056147.2	nudC domain-containing protein 3	NUDCD3	23386	0.653	17	5	18	5	361	40.8	0.665735379
NP_056240.2	DNA-directed RNA polymerase I subunit RPA1	POLR1A	25885	1.156	8	8	19	7	1720	194.7	0.529749582
NP_060864.1	mycophenolic acid acyl-glucuronide esterase, mitochondrial isoform 1 precursor	ABHD10	55347	1.472	18	5	19	5	306	33.9	0.064654214
NP_057392.1	UMP-CMP kinase isoform a	CMPK1	51727	0.421	35	7	17	7	228	25.8	0.168697149
NP_079483.3	FAD synthase isoform 1	FLAD1	80308	1.079	20	6	15	6	587	65.2	0.627725845
NP_060192.1	tRNA (guanine(26)-N(2))-dimethyltransferase isoform 1	TRMT1	55621	0.801	12	5	10	5	659	72.2	0.147428881
XP_005250211.1	protein CASP isoform X6	CUX1	1523	0.692	13	6	15	2	767	86.4	0.027776128
XP_005247870.2	fragile X mental retardation syndrome-related protein 1 isoform X1	FXR1	8087	1.111	10	7	16	5	677	76.2	0.758487758
NP_852615.1	39S ribosomal protein L21, mitochondrial isoform d	MRPL21	219927	1.171	34	5	16	5	205	22.8	0.653606359
XP_005262745.1	protein transport protein Sec24B isoform X1	SEC24B	10427	0.803	5	6	19	5	1299	140.5	0.713764299
NP_003891.1	sequestosome-1 isoform 1	SQSTM1	8878	0.887	24	6	16	6	440	47.7	0.061672531
NP_001340027.1	phosphoribosyl pyrophosphate synthase-associated protein 2 isoform 6	PRPSAP2	5636	0.663	14	4	10	2	423	46.9	0.117668164
NP_009140.1	60S ribosomal protein L35	RPL35	11224	0.74	26	4	24	4	123	14.5	0.413446528
NP_003042.3	monocarboxylate transporter 1	SLC16A1	6566	2.794	16	6	13	6	500	53.9	0.020773144
NP_064543.3	exosome complex component RRP46	EXOSC5	56915	1.117	40	6	14	6	235	25.2	0.561497722
XP_016857363.1	prostaglandin F2 receptor negative regulator isoform X1	PTGFRN	5738	1.343	6	6	14	6	885	99.3	0.411179708
NP_851853.1	LEM domain-containing protein 2 isoform 1	LEMD2	221496	1.813	15	7	18	7	503	56.9	0.000768495
XP_006723859.1	laminin subunit alpha-5 isoform X1	LAMA5	3911	1.602	2	6	16	6	3700	399.9	0.04473288
NP_001001563.1	mitochondrial import inner membrane translocase subunit TIM50 isoform 1	TIMM50	92609	1.353	12	5	15	5	456	50.4	0.384618277
NP_886552.2	CCA tRNA nucleotidyltransferase 1, mitochondrial isoform 1	TRNT1	51095	0.932	15	5	10	5	434	50.1	0.899331404
NP_001336275.1	pyridoxal phosphate homeostasis protein isoform 1	PLPBP	11212	0.708	17	4	20	4	285	31.5	0.0170387
XP_016871749.1	ADP-ribosylation factor-like protein 3 isoform X1	ARL3	403	0.695	23	4	17	4	182	20.4	0.29361742
NP_066552.2	NADH dehydrogenase	NDUFV2	4729	1.562	14	4	18	4	249	27.4	0.021724413
NP_005040.2	periodic tryptophan protein 2 homolog	PWP2	5822	1.031	7	5	19	5	919	102.4	0.991448457
XP_011514713.1	trypsin-1 isoform X1	PRSS1	5644	0.911	4	2	83	2	472	51.3	0.60214346
NP_443077.1	cob(I)yrinic acid a,c-diamide adenosyltransferase, mitochondrial precursor	MMAB	326625	1.345	20	4	10	4	250	27.4	0.709813173
XP_006713535.1	protein TFG isoform X1	TFG	10342	0.56	15	4	14	4	400	43.4	0.001070154
NP_008974.5	KRR1 small subunit processome component homolog	KRR1	11103	1.601	22	7	13	7	381	43.6	0.002860373
XP_005266132.1	dipeptidyl peptidase 2 isoform X1	DPP7	29952	1.545	16	6	16	6	565	61.8	0.040654095
NP_002072.2	glypican-1 precursor	GPC1	2817	1.416	13	6	18	6	558	61.6	0.006934142
XP_011515882.1	elongin-C isoform X1	ELOC	6921	0.568	56	5	18	5	112	12.5	0.022621561
XP_024309267.1	acylamino-acid-releasing enzyme isoform X1	APEH	327	0.345	8	5	19	5	804	88.4	0.001989039
XP_011523944.1	structural maintenance of chromosomes flexible hinge domain-containing protein 1 isoform X1	SMCHD1	23347	1.155	6	9	16	9	2067	233.6	0.619464124
NP_008921.1	wiskott-Aldrich syndrome protein family member 2 isoform 1	WASF2	10163	0.864	17	6	14	6	498	54.3	0.786199855
NP_055095.2	dnaJ homolog subfamily C member 8	DNAJC8	22826	0.778	25	5	12	5	253	29.8	0.308129109
NP_891993.1	cAMP-dependent protein kinase catalytic subunit beta isoform 1	PRKACB	5567	0.842	15	5	18	1	398	46.2	0.801300242
NP_000566.3	interleukin-1 alpha precursor	IL1A	3552	0.686	17	3	15	3	271	30.6	0.041137439
NP_116120.1	thioredoxin domain-containing protein 17	TXNDC17	84817	0.391	54	5	24	5	123	13.9	0.028758555
NP_817092.1	protein phosphatase 1G	PPM1G	5496	0.913	16	6	19	6	546	59.2	0.586494564
NP_001316422.1	ER membrane protein complex subunit 2 isoform 1	EMC2	9694	1.173	20	5	12	5	306	36	0.519832288
NP_002523.2	nuclear pore complex protein Nup88 isoform 2	NUP88	4927	1.085	14	7	13	7	741	83.5	0.720819287
NP_001269803.1	probable global transcription activator SNF2L1 isoform d	SMARCA1	6594	1.383	7	8	20	1	1070	124.2	0.737496709
NP_001265126.1	CDK5 regulatory subunit-associated protein 3 isoform c	CDK5RAP3	80279	1.652	10	5	20	5	531	59.6	0.062184414
NP_001035997.2	bifunctional coenzyme A synthase isoform c	COASY	80347	0.824	11	4	17	4	593	65.3	0.594367381
NP_009190.2	vacuolar protein sorting-associated protein 45 isoform 1	VPS45	11311	0.933	9	4	11	4	570	65	0.724438095
NP_057381.3	prenylcysteine oxidase 1 precursor	PCYOX1	51449	0.883	18	6	14	6	505	56.6	0.566775322
NP_054894.1	39S ribosomal protein L15, mitochondrial	MRPL15	29088	1.672	35	8	18	8	296	33.4	0.002483955
XP_006711430.1	ubiquitin carboxyl-terminal hydrolase isozyme L5 isoform X1	UCHL5	51377	0.864	18	6	18	6	421	47.2	0.273939856
XP_016865309.1	ubiquitin-conjugating enzyme E2 D2 isoform X1	UBE2D2	7322	0.633	20	2	14	2	173	19.6	0.443769772
NP_071751.3	prolyl 3-hydroxylase 1 isoform 1 precursor	P3H1	64175	1.46	8	7	17	7	736	83.3	0.119091838
NP_002649.1	urokinase-type plasminogen activator isoform 1 preproprotein	PLAU	5328	1.591	13	5	12	5	431	48.5	0.216303282
NP_001341528.2	isovaleryl-CoA dehydrogenase, mitochondrial isoform 5	IVD	3712	1.588	14	5	13	5	452	49.3	0.016900398
NP_036382.2	ras-related protein R-Ras2 isoform a	RRAS2	22800	1.339	19	3	20	1	204	23.4	0.027906004
NP_067038.1	regulation of nuclear pre-mRNA domain-containing protein 1B	RPRD1B	58490	1.1	25	6	16	4	326	36.9	0.919806505
NP_079491.2	tRNA pseudouridine synthase A isoform 1	PUS1	80324	1.264	15	6	18	6	427	47.4	0.032455993
NP_003680.2	aflatoxin B1 aldehyde reductase member 2 isoform 1	AKR7A2	8574	0.797	9	2	13	2	359	39.6	0.197214924
XP_024304726.1	rac GTPase-activating protein 1 isoform X1	RACGAP1	29127	1.169	16	7	17	7	651	73.1	0.765257618
NP_004598.1	tubulin-specific chaperone A isoform 2	TBCA	6902	0.548	52	7	18	7	108	12.8	0.011658637
NP_001957.2	peroxisomal bifunctional enzyme isoform 1	EHHADH	1962	1.148	9	5	13	5	723	79.4	0.31118547
NP_783316.2	metallothionein-1E isoform 2	MT1E	4493	0.079	54	3	12	1	61	6	0.103972336
NP_006256.1	double-strand-break repair protein rad21 homolog	RAD21	5885	1.278	20	7	12	7	631	71.6	0.663628418
NP_006773.2	zinc finger protein-like 1	ZFPL1	7542	1.403	17	5	15	5	310	34.1	0.172984562
NP_003095.2	sorbitol dehydrogenase	SORD	6652	0.45	28	7	18	7	357	38.3	0.000316287
XP_011523401.1	peptidyl-prolyl cis-trans isomerase FKBP10 isoform X1	FKBP10	60681	1.788	11	6	20	6	601	66.3	0.000522246
XP_011541109.1	dolichyl-diphosphooligosaccharide--protein glycosyltransferase subunit STT3A isoform X1	STT3A	3703	2.007	8	6	29	6	705	80.5	0.015375179
NP_001344607.1	galectin-3 isoform 3	LGALS3	3958	1.062	27	7	21	7	264	27.8	0.596738288
NP_078930.3	polynucleotide 5'-hydroxyl-kinase NOL9	NOL9	79707	0.996	12	4	5	4	702	79.2	0.959711053
NP_001271270.1	peptidyl-prolyl cis-trans isomerase FKBP9 isoform 2 precursor	FKBP9	11328	1.112	9	5	18	5	623	69.1	0.458618549
NP_001337434.1	probable arginine--tRNA ligase, mitochondrial isoform 3 precursor	RARS2	57038	0.982	11	5	12	5	588	66.6	0.877035117
NP_060518.1	protein mago nashi homolog 2 isoform 1	MAGOHB	55110	0.877	47	6	15	6	148	17.3	0.832052486

Patil et al,2020. Proteomic and phosphoproteomic profiling of shammah-induced signaling in oral keratinocytes

Supplementary Table S1: List of proteins identified and quantified in OKF6/TERT1-Shammah and OKF6/TERT1-Parental total proteome using TMT-based quantitative proteomic approach

Accession	Protein Description	Gene Symbol	Gene ID	Fold Change	Coverage [%]	# Peptides	# PSMs	# Unique Peptides	# AAs	MW [kDa]	p-value
NP_003192.1	transcription factor A, mitochondrial isoform 1 precursor	TFAM	7019	2.173	28	7	22	7	246	29.1	0.080554476
NP_001138888.1	transmembrane protein 205	TMEM205	374882	1.547	16	2	11	2	189	21.2	0.301271216
NP_002363.2	alpha-mannosidase 2	MAN2A1	4124	1.015	6	7	16	7	1144	131.1	0.667849358
NP_079510.1	WD repeat-containing protein 61 isoform a	WDR61	80349	0.933	30	6	8	6	305	33.6	0.79134854
NP_060707.2	cell division cycle and apoptosis regulator protein 1 isoform a	CCAR1	55749	1.455	7	8	19	8	1150	132.7	0.136764594
XP_011507442.1	protein Niban isoform X1	FAM129A	116496	0.558	6	5	11	5	987	110.3	0.021766029
NP_067639.1	retinoid-inducible serine carboxypeptidase precursor	SCPEP1	59342	1.234	12	5	13	5	452	50.8	0.706824906
XP_005263439.1	ras GTPase-activating protein-binding protein 2 isoform X1	G3BP2	9908	0.893	12	4	10	4	482	54.1	0.323546132
NP_060754.2	guanylate-binding protein 3 isoform 1	GBP3	2635	0.612	12	5	10	1	595	68.1	0.57771489
XP_011532899.1	endoplasmic reticulum-Golgi intermediate compartment protein 1 isoform X1	ERGIC1	57222	1.066	18	4	8	4	314	35.1	0.976497365
NP_001670.1	sodium/potassium-transporting ATPase subunit beta-3	ATP1B3	483	1.857	44	8	23	8	279	31.5	0.017344513
NP_001309046.1	pyruvate dehydrogenase phosphatase regulatory subunit, mitochondrial isoform 1 precursor	PDPR	55066	1.695	7	5	15	5	879	99.3	0.00392334
XP_005257825.1	C-Jun-amino-terminal kinase-interacting protein 4 isoform X1	SPAG9	9043	0.644	7	7	13	7	1334	147.5	0.000610794
XP_024304262.1	integrin-linked protein kinase isoform X1	ILK	3611	0.817	13	5	15	5	483	54.6	0.574906752
NP_001274730.1	tubulin-specific chaperone E isoform b	TBCE	6905	0.496	8	5	17	5	578	64.8	0.0010541
NP_075068.2	nucleolar protein 6 isoform alpha	NOL6	65083	1.125	9	7	17	7	1146	127.5	0.563105469
NP_073605.4	RNA-binding protein 15 isoform 1	RBM15	64783	1.481	10	7	11	7	977	107.1	0.775074467
NP_002907.1	replication factor C subunit 4	RFC4	5984	0.996	34	8	16	8	363	39.7	0.958300513
NP_079106.3	lysophosphatidylcholine acyltransferase 1	LPCAT1	79888	1.274	9	4	11	4	534	59.1	0.00710409
NP_005092.1	ubiquitin-like protein ISG15	ISG15	9636	0.262	32	4	11	4	165	17.9	0.017335404
NP_001159864.1	trophoblast glycoprotein precursor	TPBG	7162	1.53	14	5	24	5	420	46	0.128557927
NP_115683.3	queuosine salvage protein isoform 1	C9orf64	84267	0.691	15	5	18	5	341	39	0.00530941
NP_003253.1	translocation protein SEC62	SEC62	7095	1.791	12	6	17	6	399	45.8	0.000423795
XP_011527207.1	acetyl-coenzyme A synthetase, cytoplasmic isoform X1	ACSS2	55902	0.873	13	8	17	8	737	82.7	0.716533142
XP_016869245.1	serine/threonine-protein kinase 3 isoform X1	STK3	6788	0.722	7	3	10	1	580	66.2	0.006168369
NP_036552.1	interferon-induced protein with tetratricopeptide repeats 5	IFIT5	24138	0.851	17	7	12	6	482	55.8	0.38208391
NP_001124313.1	RNA 3'-terminal phosphate cyclase isoform a	RTCA	8634	0.584	10	3	17	3	379	40.7	0.005070025
NP_001239007.1	ubiquitin carboxyl-terminal hydrolase 15 isoform 1	USP15	9958	0.633	6	5	17	5	981	112.3	0.00222866
XP_011518601.1	chitinase domain-containing protein 1 isoform X1	CHID1	66005	1.507	13	6	17	6	478	53.4	0.236914672
NP_000704.1	flavin reductase (NADPH)	BLVRB	645	0.535	43	5	8	5	206	22.1	0.160272701
NP_006215.1	phosphatidylinositol transfer protein alpha isoform	PITPNA	5306	0.521	25	6	20	3	270	31.8	0.640320762
NP_061830.3	SH3 domain-binding protein 1 isoform 1	SH3BP1	23616	0.833	10	6	12	6	701	75.7	0.470930333
NP_056377.1	UBX domain-containing protein 7	UBXN7	26043	0.834	13	4	13	4	489	54.8	0.188515732
NP_006030.2	C-type mannose receptor 2 precursor	MRC2	9902	1.744	5	6	19	6	1479	166.6	0.000411391
NP_071933.2	ubiquitin-like-conjugating enzyme ATG3 isoform 1	ATG3	64422	0.514	22	4	12	4	314	35.8	0.164755281
NP_057665.2	evolutionarily conserved signaling intermediate in Toll pathway, mitochondrial isoform 1 precursor	ECSIT	51295	1.053	15	5	15	5	431	49.1	0.888259806
NP_056292.1	paired amphipathic helix protein Sin3a	SIN3A	25942	0.867	5	5	13	5	1273	145.1	0.782566651
NP_001003796.1	NHP2-like protein 1	SNU13	4809	1.323	37	4	20	4	128	14.2	0.036083494
XP_024302967.1	focal adhesion kinase 1 isoform X1	PTK2	5747	0.92	10	8	16	7	1119	126.9	0.078510257
NP_006038.2	RNA-binding protein 12	RBM12	10137	0.914	8	8	23	8	932	97.3	0.129331739
NP_000108.1	emerin	EMD	2010	1.469	13	4	13	4	254	29	0.096178755
NP_004703.1	hepatocyte growth factor-regulated tyrosine kinase substrate	HGS	9146	0.84	7	6	15	6	777	86.1	0.678433941
NP_004689.1	U4/U6 small nuclear ribonucleoprotein Prp3 isoform 1	PRPF3	9129	1.59	10	5	13	5	683	77.5	0.016424446
NP_000225.1	DNA ligase 1 isoform 1	LIG1	3978	0.472	10	5	9	5	919	101.7	0.431681876
NP_563615.3	discoidin, CUB and LCCL domain-containing protein 2 precursor	DCBLD2	131566	1.039	10	3	9	3	775	85	0.899018491
XP_016874755.1	inositol 1,4,5-trisphosphate receptor type 2 isoform X1	ITPR2	3709	1.109	2	7	17	3	2721	310	0.934492143
NP_002018.1	protein farnesyltransferase/geranylgeranyltransferase type-1 subunit alpha	FNTA	2339	0.498	15	4	12	4	379	44.4	0.289686893
NP_001293135.1	cleavage stimulation factor subunit 2 isoform 1	CSTF2	1478	1.453	12	4	19	4	597	62.9	0.000257007
NP_065385.2	pre-B-cell leukemia transcription factor-interacting protein 1 isoform 1	PBXIP1	57326	1.379	10	6	14	6	731	80.6	0.23599309
XP_016876581.1	peptidyl-prolyl cis-trans isomerase FKBP3 isoform X1	FKBP3	2287	0.697	19	6	15	6	229	26	0.170267661
NP_644810.1	WD repeat-containing protein 36	WDR36	134430	1.201	8	6	16	6	951	105.3	0.687728066
NP_000128.1	fumarylacetoacetase	FAH	2184	0.633	15	5	15	5	419	46.3	0.028017303
NP_478126.1	exosome complex component MTR3	EXOSC6	118460	1.038	22	5	16	5	272	28.2	0.903441328
NP_001123543.1	epsin-1 isoform a	EPN1	29924	1.055	15	4	6	3	662	69	0.798562989
NP_001139443.1	retinol dehydrogenase 13 isoform 1 precursor	RDH13	112724	1.092	20	4	12	4	331	35.9	0.182813637
NP_056311.2	protein virilizer homolog isoform 1	VIRMA	25962	1.028	4	5	13	5	1812	201.9	0.925588684
NP_001159403.1	ribonucleoside-diphosphate reductase subunit M2 isoform 1	RRM2	6241	0.754	16	5	19	5	449	51.1	0.204557195
NP_004949.1	serine/threonine-protein kinase mTOR	MTOR	2475	0.587	4	8	13	8	2549	288.7	0.030487481
NP_004918.1	39S ribosomal protein L49, mitochondrial	MRPL49	740	1.785	35	5	11	5	166	19.2	0.235116766
NP_075067.2	vacuolar protein sorting-associated protein 33A isoform 1	VPS33A	65082	0.628	12	5	13	5	596	67.6	0.09421394
NP_542408.1	dynein light chain 2, cytoplasmic	DYNLL2	140735	1.092	33	5	15	2	89	10.3	0.94310083
NP_001308765.1	cleavage and polyadenylation specificity factor subunit 3 isoform d	CPSF3	51692	1.064	8	4	11	4	688	78	0.962658425
NP_001824.1	clathrin light chain A isoform a	CLTA	1211	1.503	32	8	20	7	218	23.6	0.064033774
XP_005264930.1	phosphatidate cytidylyltransferase, mitochondrial isoform X1	TAMM41	132001	1.673	14	4	10	4	452	51	0.092827959
XP_016880484.1	nucleolar transcription factor 1 isoform X1	UBTF	7343	1.338	6	4	9	4	781	91.2	0.527102126
NP_001135757.1	40S ribosomal protein S24 isoform d	RPS24	6229	0.774	12	3	21	3	289	32.4	0.071278235
NP_006398.1	PRA1 family protein 3	ARL6IP5	10550	1.876	20	3	14	3	188	21.6	0.014432304
NP_055829.2	RNA-binding protein 34 isoform 1	RBM34	23029	1.013	19	6	21	6	430	48.5	0.986165757
NP_006313.1	gamma-tubulin complex component 3 isoform 1	TUBGCP3	10426	0.732	8	5	14	5	907	103.5	0.500503027
NP_064574.1	transcription and mRNA export factor ENY2 isoform 1	ENY2	56943	1.406	26	3	12	3	101	11.5	0.017119789
NP_057501.2	glutaredoxin-related protein 5, mitochondrial precursor	GLRX5	51218	1.738	37	5	9	5	157	16.6	0.029416046
NP_001341917.1	tyrosine-protein kinase CSK	CSK	1445	0.755	18	7	20	7	450	50.7	0.111760494
XP_024302876.1	exportin-7 isoform X1	XPO7	23039	0.631	8	6	16	6	1099	125.2	0.12609598
NP_001227.1	carbonyl reductase	CBR3	874	0.54	20	4	15	1	277	30.8	0.901062231
NP_060598.3	pre-rRNA-processing protein TSR1 homolog	TSR1	55720	0.683	10	5	9	5	804	91.8	0.492319499
NP_001020262.1	ATP-binding cassette sub-family F member 1 isoform a	ABCF1	23	0.814	15	7	19	7	845	95.9	0.354757202
NP_055577.1	delta(24)-sterol reductase precursor	DHCR24	1718	0.739	14	8	23	8	516	60.1	0.12516714
NP_055851.1	endonuclease domain-containing 1 protein precursor	ENDOD1	23052	1.01	18	4	15	4	500	55	0.647902136
NP_055984.1	ribosome biogenesis regulatory protein homolog	RRS1	23212	1.89	22	6	8	6	365	41.2	0.079091521
NP_001278907.1	nucleolar protein 14 isoform 1	NOP14	8602	1.289	9	7	14	7	857	97.6	0.253507323
XP_024305924.1	cytochrome c oxidase subunit 4 isoform 1, mitochondrial isoform X1	COX4I1	1327	1.058	26	5	11	5	175	20.1	0.68757384
XP_016874257.1	oxysterol-binding protein-related protein 8 isoform X4	OSBPL8	114882	1.209	8	5	15	5	912	103.9	0.289609571
NP_115867.2	39S ribosomal protein L38, mitochondrial	MRPL38	64978	1.386	19	6	15	6	380	44.6	0.280503546
XP_005270309.1	ATPase family AAA domain-containing protein 1 isoform X1	ATAD1	84896	1.573	21	7	17	7	361	40.7	0.175533861
NP_054736.1	ragulator complex protein LAMTOR2 isoform 1	LAMTOR2	28956	0.863	32	4	21	4	125	13.5	0.393533416
XP_011537080.1	aladin isoform X1	AAAS	8086	1.179	11	5	10	5	560	60.9	0.31469778
NP_001265586.1	polymerase delta-interacting protein 3 isoform 3	POLDIP3	84271	0.955	16	4	13	4	438	48.1	0.449089167
NP_001887.1	casein kinase II subunit alpha'	CSNK2A2	1459	0.413	20	6	11	5	350	41.2	0.082386774
NP_001259004.1	ubiquitin carboxyl-terminal hydrolase 10 isoform 1	USP10	9100	0.819	11	7	16	7	802	87.5	0.811235317
XP_024303707.1	AP-3 complex subunit mu-1 isoform X1	AP3M1	26985	0.685	14	4	14	4	418	46.9	0.102204778
NP_061184.1	conserved oligomeric Golgi complex subunit 1	COG1	9382	0.803	9	5	8	5	980	108.9	0.709281217
NP_001015055.1	rhotekin isoform a	RTKN	6242	0.781	12	4	11	4	563	62.6	0.699194457
NP_006311.2	membrane-associated progesterone receptor component 2	PGRMC2	10424	1.845	26	4	13	4	247	26.2	0.054895294
NP_056338.2	oligoribonuclease, mitochondrial precursor	REXO2	25996	0.8	20	5	10	5	237	26.8	0.688967861
NP_056009.1	unconventional myosin-Id isoform 1	MYO1D	4642	1.045	8	7	14	7	1006	116.1	0.988518628
NP_001988.1	ubiquitin-like protein fubi and ribosomal protein S30 precursor	FAU	2197	0.891	9	4	26	4	133	14.4	0.342827961
XP_011515885.1	ubiquitin-conjugating enzyme E2 variant 2 isoform X1	UBE2V2	7336	0.559	32	6	16	3	173	19.5	0.299850947
NP_001015.1	40S ribosomal protein S21	RPS21	6227	0.839	53	4	15	4	83	9.1	0.300731904
NP_003123.2	spermidine synthase	SRM	6723	0.636	28	6	15	6	302	33.8	0.006411053
NP_057078.1	V-type proton ATPase subunit D	ATP6V1D	51382	0.914	25	5	10	5	247	28.2	0.860590345
XP_016874237.1	exportin-T isoform X1	XPOT	11260	0.499	9	5	12	5	962	109.9	0.012044066
NP_001269516.1	GDH/6PGL endoplasmic bifunctional protein isoform 1 precursor	H6PD	9563	1.406	9	6	15	6	802	90.1	0.089255002
NP_001139019.1	splicing factor 45	RBM17	84991	1.312	17	6	15	5	401	44.9	0.001044666
NP_055844.2	ribonucleases P/MRP protein subunit POP1	POP1	10940	0.918	6	4	11	4	1024	114.6	0.684848062
XP_011544158.2	acyl carrier protein, mitochondrial isoform X1	NDUFAB1	4706	2.026	18	3	23	3	187	20.8	0.00183485
XP_006714818.1	synaptopodin isoform X1	SYNPO	11346	1.046	5	4	11	4	1147	122.1	0.88503266
NP_001137467.1	leupaxin isoform 1	LPXN	9404	0.478	14	3	16	3	391	43.7	0.000222595
NP_057086.2	saccharopine dehydrogenase-like oxidoreductase	SCCPDH	51097	1.904	18	5	15	5	429	47.1	0.009374535
XP_005270419.1	peptidyl-prolyl cis-trans isomerase H isoform X1	PPIH	10465	0.779	27	5	16	5	177	19.2	0.262233137
NP_006285.1	cytochrome b-c1 complex subunit 7 isoform 1	UQCRB	7381	1.825	47	5	14	5	111	13.5	0.000676236
NP_067027.1	enolase-phosphatase E1 isoform 1	ENOPH1	58478	0.456	26	4	8	4	261	28.9	0.050521049
NP_001317692.1	isocitrate dehydrogenase	IDH3B	3420	0.908	14	5	16	5	387	42.4	0.995723674
NP_001280569.1	WASH complex subunit 4 isoform 1	WASHC4	23325	0.712	4	4	13	4	1174	136.4	0.049408199
XP_016866899.1	serine/threonine-protein kinase PRP4 homolog isoform X1	PRPF4B	8899	1.317	8	7	15	7	1007	116.9	0.074553446
NP_055545.1	malectin isoform1 precursor	MLEC	9761	1.942	24	6	18	6	292	32.2	0.000290121
XP_005247645.1	protein CDV3 homolog isoform X1	CDV3	55573	0.523	43	7	14	7	259	27.5	0.299989265
XP_016872590.1	CUGBP Elav-like family member 1 isoform X1	CELF1	10658	1.41	7	4	11	4	514	55.1	0.239694268
NP_060829.2	ufm1-specific protease 2	UFSP2	55325	1.078	16	6	14	6	469	53.2	0.765722003
NP_056330.3	keratin, type I cytoskeletal 23 isoform 1	KRT23	25984	2.137	18	6	18	6	422	48.1	0.009769943
XP_016878726.1	multidrug resistance-associated protein 1 isoform X1	ABCC1	4363	1.324	4	4	15	4	1549	173.8	0.240897211
NP_001017928.1	coiled-coil domain-containing protein 58 isoform 1	CCDC58	131076	1.419	32	5	16	5	144	16.6	0.010915036
XP_005257516.1	V-type proton ATPase 116 kDa subunit a isoform X1	ATP6V0A1	535	1.174	11	7	13	7	844	97.2	0.544930885
XP_024309466.1	60S ribosomal protein L29 isoform X1	RPL29	6159	0.989	14	2	17	2	168	18.6	0.969259779
NP_009031.1	ATP synthase subunit e, mitochondrial	ATP5ME	521	1.07	42	3	17	3	69	7.9	0.88234211
XP_011517854.2	sickle tail protein homolog isoform X1	KIAA1217	56243	1.304	4	5	8	5	2017	222	0.670668831
XP_005256131.1	TLD domain-containing protein 1 isoform X1	MEAK7	57707	0.653	17	4	11	4	494	54.8	0.020264718
NP_127460.1	H/ACA ribonucleoprotein complex subunit 1	GAR1	54433	1.283	19	4	15	4	217	22.3	0.378469158
XP_011539879.1	peroxisomal membrane protein PEX14 isoform X1	PEX14	5195	2.2	15	5	13	5	391	43	0.0057811
NP_001687.1	V-type proton ATPase subunit E 1 isoform a	ATP6V1E1	529	0.677	15	3	6	3	226	26.1	0.522222776
XP_005265063.1	probable leucine--tRNA ligase, mitochondrial isoform X1	LARS2	23395	1.3	10	6	20	6	903	101.9	0.080375801
NP_808881.3	2',5'-phosphodiesterase 12 isoform 1 precursor	PDE12	201626	0.905	13	5	13	5	609	67.3	0.172931865
XP_005267329.1	exocyst complex component 5 isoform X1	EXOC5	10640	0.887	9	6	13	6	746	85.4	0.756966095
NP_054884.1	NADH dehydrogenase	NDUFAF4	29078	1.363	29	4	14	4	175	20.3	0.230692757
XP_024307215.1	ferritin light chain isoform X1	FTL	2512	0.475	16	4	13	4	345	37.9	0.000771584
NP_001091990.1	oxidoreductase HTATIP2 isoform a precursor	HTATIP2	10553	1.824	20	5	13	5	276	30.1	1.66E-06
XP_011509820.1	prothymosin alpha isoform X1	PTMA	5757	0.544	9	3	18	3	263	27.9	0.007605741
NP_057225.2	cytoplasmic dynein 1 light intermediate chain 1 isoform 1	DYNC1LI1	51143	0.536	19	5	11	5	523	56.5	0.003883114
XP_011536999.1	signal transducer and activator of transcription 2 isoform X1	STAT2	6773	0.615	10	6	14	6	859	98.7	0.091962798
XP_011526303.1	long-chain fatty acid transport protein 1 isoform X1	SLC27A1	376497	1.829	11	5	13	5	658	72.4	0.13296092
NP_001135452.1	TP53-binding protein 1 isoform 1	TP53BP1	7158	1.137	7	8	16	8	1977	214	0.753558972
NP_005889.3	caprin-1 isoform 1	CAPRIN1	4076	0.742	10	6	17	6	709	78.3	0.406480861
NP_006627.2	bifunctional methylenetetrahydrofolate dehydrogenase/cyclohydrolase, mitochondrial precursor	MTHFD2	10797	0.904	18	4	9	4	350	37.9	0.876887453
NP_003631.2	elongator complex protein 1 isoform 1	ELP1	8518	0.549	8	8	14	8	1332	150.2	0.004438401
NP_003747.1	eukaryotic translation initiation factor 3 subunit H	EIF3H	8667	0.888	15	5	19	5	352	39.9	0.545162036
XP_005260587.1	serine/threonine-protein kinase 4 isoform X1	STK4	6789	0.82	11	4	8	2	503	57.1	0.814542328
NP_060760.2	phosphoglucomutase-2	PGM2	55276	0.703	14	6	13	6	612	68.2	0.381186923
NP_006766.1	protein quaking isoform 1	QKI	9444	1.077	18	5	15	5	341	37.6	0.696592531
NP_055535.2	STE20-like serine/threonine-protein kinase isoform 1	SLK	9748	0.662	5	5	12	5	1235	142.6	0.002911569
XP_011544421.1	bcl-2-like protein 13 isoform X1	BCL2L13	23786	1.169	16	5	11	5	509	54.4	0.661520606
NP_065724.1	ras-related protein Rab-22A	RAB22A	57403	1.547	26	4	12	2	194	21.8	0.019278269
XP_016883301.1	protein GNAS isoform X1	GNAS	2778	1.347	6	4	24	3	1038	111	0.46124586
XP_011509802.1	mitochondrial fission factor isoform X1	MFF	56947	1.184	29	4	12	4	342	38.4	0.65465179
XP_011517554.1	protein transport protein Sec16A isoform X5	SEC16A	9919	0.891	5	7	11	7	2357	251.7	0.827572496
NP_057529.1	pleckstrin-2	PLEK2	26499	0.67	13	3	10	3	353	39.9	0.242143993
NP_005850.1	transcriptional activator protein Pur-alpha	PURA	5813	0.841	17	5	12	4	322	34.9	0.650619346
NP_056005.1	dnaJ homolog subfamily C member 9	DNAJC9	23234	0.433	15	4	9	4	260	29.9	0.000150315
NP_005497.1	lysosome membrane protein 2 isoform 1 precursor	SCARB2	950	1.442	11	5	17	5	478	54.3	0.26477585
XP_016874803.1	mevalonate kinase isoform X3	MVK	4598	0.576	23	6	13	6	396	42.4	0.149193725
NP_001061.2	tubulin gamma-1 chain	TUBG1	7283	1.174	18	5	11	5	451	51.1	0.956645753
NP_078813.1	probable cysteine--tRNA ligase, mitochondrial isoform 1	CARS2	79587	1.626	11	6	15	6	564	62.2	0.024776294
XP_005253223.1	ADP-ribosylation factor GTPase-activating protein 2 isoform X1	ARFGAP2	84364	0.612	8	4	11	4	560	60.9	0.36728409
XP_005247421.1	Golgi integral membrane protein 4 isoform X1	GOLIM4	27333	1.018	6	4	13	4	697	81.9	0.936750215
XP_011533696.1	activating signal cointegrator 1 complex subunit 3 isoform X1	ASCC3	10973	0.824	4	7	19	7	2207	251.9	0.208457697
XP_011508283.1	39S ribosomal protein L24, mitochondrial isoform X1	MRPL24	79590	1.37	34	7	18	7	216	24.9	0.752147828
NP_115866.1	39S ribosomal protein L41, mitochondrial	MRPL41	64975	1.168	63	6	15	6	137	15.4	0.594391891
XP_011511125.1	lipoma-preferred partner isoform X1	LPP	4026	0.687	11	4	9	4	637	68.4	0.673243872
NP_057701.1	ubiquitin-fold modifier 1 isoform 1 precursor	UFM1	51569	0.538	59	3	17	3	85	9.1	0.035094188
NP_057085.2	U3 small nucleolar RNA-associated protein 18 homolog	UTP18	51096	1.514	12	4	10	4	556	62	0.536353302
NP_001108090.1	alpha-1,3-mannosyl-glycoprotein 2-beta-N-acetylglucosaminyltransferase	MGAT1	4245	1.219	15	6	15	6	445	50.8	0.230790675
NP_060537.3	MAP7 domain-containing protein 1 isoform 1	MAP7D1	55700	0.561	7	5	16	5	841	92.8	0.017443
NP_001077861.1	D-dopachrome decarboxylase	DDT	1652	0.376	35	4	20	4	118	12.7	0.033485977
NP_115729.1	vacuolar protein-sorting-associated protein 25	VPS25	84313	0.7	28	5	12	5	176	20.7	0.026819327
NP_001339425.1	asparagine synthetase	ASNS	440	0.308	13	6	15	6	561	64.3	2.30E-05
XP_005263436.1	protein transport protein Sec24D isoform X1	SEC24D	9871	0.373	6	6	15	5	1033	113	0.003049769
XP_024309756.1	protein furry homolog-like isoform X1	FRYL	285527	0.85	3	7	15	7	3120	351.4	0.095254244
XP_011533514.1	ubiquitin carboxyl-terminal hydrolase isozyme L3 isoform X1	UCHL3	7347	0.67	16	5	20	5	294	33.2	0.024687225
NP_490597.1	bromodomain-containing protein 4 isoform long	BRD4	23476	1.455	4	5	14	3	1362	152.1	0.002666466
NP_009049.2	triple functional domain protein	TRIO	7204	0.619	2	4	11	4	3097	346.7	0.010655941
XP_016866764.1	large proline-rich protein BAG6 isoform X1	BAG6	7917	0.686	7	6	10	6	1182	124.2	0.680241627
XP_005250207.1	protein CASP isoform X1	CUX1	1523	1.358	4	5	12	1	1604	174.5	0.874921386
NP_997636.1	cAMP-dependent protein kinase type I-alpha regulatory subunit isoform a	PRKAR1A	5573	0.837	14	5	18	5	381	43	0.28954895
XP_005255118.1	ataxin-2-like protein isoform X1	ATXN2L	11273	0.814	8	6	13	6	1104	116.3	0.587136658
NP_001287750.1	eukaryotic translation initiation factor 4B isoform 1	EIF4B	1975	0.513	11	5	16	5	616	69.7	0.043336556
XP_011524051.1	mRNA cap guanine-N7 methyltransferase isoform X1	RNMT	8731	1.252	10	4	10	4	512	58.5	0.391835051
NP_443082.2	growth arrest and DNA damage-inducible proteins-interacting protein 1	GADD45GIP1	90480	1.158	36	5	8	5	222	25.4	0.830296314
XP_016856465.1	dynamin-3 isoform X1	DNM3	26052	0.594	5	4	11	1	921	103.7	0.465112691
NP_003918.1	methyl-CpG-binding domain protein 2 isoform 1	MBD2	8932	1.375	16	5	10	5	411	43.2	0.127919434
XP_016882814.1	UBX domain-containing protein 6 isoform X1	UBXN6	80700	0.617	22	5	11	5	475	53.1	0.588065556
NP_003767.2	39S ribosomal protein L40, mitochondrial isoform 1	MRPL40	64976	1.703	13	2	18	2	206	24.5	0.002222857
XP_016869602.1	WASH complex subunit 5 isoform X1	WASHC5	9897	0.713	6	5	10	5	1159	134.2	0.397967408
NP_001303963.1	dolichol-phosphate mannosyltransferase subunit 1 isoform 1	DPM1	8813	1.561	19	6	18	6	295	33.3	0.200523804
NP_003238.2	thrombospondin-2 precursor	THBS2	7058	1.65	6	5	12	3	1172	129.9	0.352957919
NP_055726.3	AP2-associated protein kinase 1	AAK1	22848	0.775	8	6	13	6	961	103.8	0.687753439
NP_079110.1	mini-chromosome maintenance complex-binding protein isoform 1	MCMBP	79892	0.572	14	5	10	5	642	72.9	0.036086258
NP_443173.2	guanylate-binding protein 4	GBP4	115361	0.736	10	4	11	3	640	73.1	0.460727109
XP_011528149.1	protein kinase C and casein kinase substrate in neurons protein 2 isoform X1	PACSIN2	11252	0.654	10	6	13	6	488	55.9	0.151028685
NP_055100.2	exosome complex component RRP4 isoform 1	EXOSC2	23404	0.841	15	3	10	3	293	32.8	0.721851401
NP_001139266.1	anthrax toxin receptor 2 isoform 2 precursor	ANTXR2	118429	0.807	9	4	8	4	489	53.6	0.866287898
NP_000989.1	60S ribosomal protein L37a	RPL37A	6168	0.847	59	5	26	5	92	10.3	0.067705238
NP_002386.1	NADP-dependent malic enzyme	ME1	4199	0.426	16	5	9	5	572	64.1	0.07739181
XP_011510613.1	structural maintenance of chromosomes protein 4 isoform X1	SMC4	10051	0.653	7	8	14	8	1288	147.1	0.02862006
NP_067653.3	UPF0160 protein MYG1, mitochondrial precursor	C12orf10	60314	0.719	17	4	12	4	376	42.4	0.209320183
NP_004544.1	NADH dehydrogenase	NDUFS6	4726	1.864	23	2	8	2	124	13.7	0.327413142
NP_006829.1	methionine aminopeptidase 2 isoform 1	METAP2	10988	0.62	9	4	12	4	478	52.9	2.16E-05
NP_064581.2	pre-mRNA-splicing factor SYF1	XAB2	56949	1.194	6	4	9	4	855	99.9	0.687252169
XP_016884213.1	apolipoprotein L2 isoform X2	APOL2	23780	1.27	10	4	12	4	449	48.9	0.293617365
XP_024309835.1	nuclear factor NF-kappa-B p105 subunit isoform X1	NFKB1	4790	0.927	7	5	9	5	977	106.4	0.949958816
XP_011511807.1	cell growth-regulating nucleolar protein isoform X1	LYAR	55646	1.008	21	6	12	6	379	43.6	0.803396539
NP_060087.3	neurogenic locus notch homolog protein 1 preproprotein	NOTCH1	4851	1.038	3	5	14	5	2555	272.3	0.982436964
NP_002906.1	replication factor C subunit 3 isoform 1	RFC3	5983	0.853	24	7	14	7	356	40.5	0.432267405
NP_000445.1	superoxide dismutase	SOD1	6647	0.806	32	5	14	5	154	15.9	0.4903623
NP_115898.2	protein MAK16 homolog	MAK16	84549	1.149	15	4	11	4	300	35.3	0.789651096
NP_060531.2	pre-mRNA-splicing factor 38B isoform a	PRPF38B	55119	1.44	7	5	13	5	546	64.4	0.017038748
XP_024304295.1	nucleobindin-2 isoform X1	NUCB2	4925	1.812	13	5	16	4	425	50.8	0.119277267
NP_005827.1	2-iminobutanoate/2-iminopropanoate deaminase	RIDA	10247	1.477	53	5	13	5	137	14.5	0.361266415
NP_835361.1	adenosine 3'-phospho 5'-phosphosulfate transporter 1 isoform a precursor	SLC35B2	347734	1.598	12	5	11	5	432	47.5	0.041763528
NP_001308498.1	calcium/calmodulin-dependent protein kinase type II subunit delta isoform 9	CAMK2D	817	0.667	12	5	11	3	533	60	0.555838843
NP_036590.1	mitochondrial import inner membrane translocase subunit Tim13	TIMM13	26517	1.368	26	2	11	2	95	10.5	0.416542998
NP_003135.2	translocon-associated protein subunit alpha isoform 1 precursor	SSR1	6745	1.649	12	3	26	3	286	32.2	0.028658809
XP_006712541.1	arf-GAP domain and FG repeat-containing protein 1 isoform X1	AGFG1	3267	0.751	10	4	11	4	602	62.6	0.020303808
NP_006003.1	ATP-dependent Clp protease proteolytic subunit, mitochondrial precursor	CLPP	8192	1.268	28	4	12	4	277	30.2	0.235046318
NP_004441.1	enhancer of rudimentary homolog	ERH	2079	1.618	38	4	13	4	104	12.3	8.45E-06
XP_011512465.1	pachytene checkpoint protein 2 homolog isoform X1	TRIP13	9319	0.755	12	4	11	4	432	48.5	0.207983299
NP_003837.1	peroxisomal membrane protein 11B isoform 1	PEX11B	8799	1.462	15	4	9	4	259	28.4	0.090095388
NP_001304028.1	dnaJ homolog subfamily B member 4 isoform a	DNAJB4	11080	0.778	9	3	10	2	337	37.8	0.776911976
NP_996790.3	5'-AMP-activated protein kinase catalytic subunit alpha-1 isoform 2	PRKAA1	5562	0.786	13	5	10	5	574	65.5	0.079585974
NP_006224.1	DNA-directed RNA polymerase II subunit RPB9	POLR2I	5438	0.796	18	1	6	1	125	14.5	0.336056916
XP_011534876.1	nesprin-2 isoform X1	SYNE2	23224	1.103	1	6	16	6	6936	801.7	0.613418154
NP_036256.2	cysteine and histidine-rich domain-containing protein 1 isoform a	CHORDC1	26973	0.585	14	4	9	4	332	37.5	0.126746736
NP_001029097.1	nucleolysin TIAR isoform 2	TIAL1	7073	1.153	15	5	13	3	392	43.4	0.78038878
NP_066932.1	thymosin beta-4	TMSB4X	7114	0.44	45	4	10	4	44	5.1	0.069893625
XP_011514858.1	eukaryotic translation initiation factor 4H isoform X1	EIF4H	7458	0.446	30	4	20	4	250	27.6	0.09632595
XP_024307137.1	kinesin light chain 3 isoform X1	KLC3	147700	0.606	10	5	11	5	703	76.7	0.608236564
XP_011538278.1	transmembrane 9 superfamily member 3 isoform X1	TM9SF3	56889	1.786	7	4	17	4	607	70.2	0.068436606
NP_004292.1	actin-like protein 6A isoform 1	ACTL6A	86	1.064	12	5	11	4	429	47.4	0.739806221
NP_006690.1	mannosyl-oligosaccharide 1,2-alpha-mannosidase IB	MAN1A2	10905	1.08	7	4	9	4	641	73	0.8896579
NP_006442.2	polyadenylate-binding protein-interacting protein 1 isoform 1	PAIP1	10605	0.272	9	3	10	3	479	53.5	0.01135185
NP_001303260.1	ABHD14A-ACY1 readthrough (NMD candidate)	ABHD14A-ACY1	100526760	0.554	14	6	12	6	498	56	0.268256594
XP_024303161.1	WD repeat-containing protein 5 isoform X1	WDR5	11091	1.307	17	4	13	4	334	36.6	0.528557575
NP_001337049.1	semaphorin-3C isoform 1	SEMA3C	10512	2.546	7	6	17	6	769	87.2	0.022833158
NP_037481.1	neudesin precursor	NENF	29937	1.905	27	3	11	3	172	18.8	0.003955036
NP_001190991.1	COMMD3-BMI1 read-through protein	COMMD3-BMI1	100532731	0.609	6	2	9	2	469	53.3	0.002281252
NP_003560.2	syntaxin-7 isoform a	STX7	8417	1.699	23	5	15	5	261	29.8	0.002069357
NP_001230208.1	acyl-CoA synthetase family member 3, mitochondrial isoform 1 precursor	ACSF3	197322	0.83	8	3	11	3	576	64.1	0.921572309
NP_036218.1	general transcription factor 3C polypeptide 3 isoform 1	GTF3C3	9330	1.482	7	4	10	4	886	101.2	0.08526919
NP_057396.1	WW domain-binding protein 11	WBP11	51729	1.453	8	4	10	4	641	70	0.0044459
NP_110517.2	beta-catenin-like protein 1 isoform 1	CTNNBL1	56259	1.195	9	6	20	6	563	65.1	0.612152591
XP_024307564.1	very-long-chain enoyl-CoA reductase isoform X8	TECR	9524	1.444	17	5	20	5	338	39.2	0.227259949
NP_001092281.1	septin-8 isoform a	SEPT8	23176	0.736	10	5	26	4	483	55.7	0.238214309
XP_011538248.1	2-oxoglutarate dehydrogenase-like, mitochondrial isoform X1	OGDHL	55753	0.893	6	5	17	1	1010	114.4	0.653458492
XP_006719975.1	muscleblind-like protein 2 isoform X1	MBNL2	10150	1.179	9	4	11	1	391	42.4	0.747163321
NP_942568.2	TBC1 domain family member 9B isoform a	TBC1D9B	23061	0.738	4	4	10	4	1250	140.4	0.688045324
NP_065132.1	Golgi-associated PDZ and coiled-coil motif-containing protein isoform a	GOPC	57120	1.017	11	5	8	5	462	50.5	0.979007377
NP_001026997.1	bolA-like protein 2 isoform 1	BOLA2	552900	0.591	29	3	7	3	152	16.9	0.349592707
NP_005650.2	ubiquitin recognition factor in ER-associated degradation protein 1 isoform A	UFD1	7353	0.851	18	5	12	5	307	34.5	0.568046984
XP_016869043.1	protein FAM49B isoform X2	FAM49B	51571	0.782	17	4	11	4	324	36.8	0.575668941
NP_005754.2	alpha-aminoadipic semialdehyde synthase, mitochondrial	AASS	10157	1.123	9	5	9	5	926	102.1	0.778723392
XP_016882424.1	serine/threonine-protein phosphatase 5 isoform X1	PPP5C	5536	0.418	10	5	10	5	551	62.3	0.622574845
NP_009185.2	bifunctional polynucleotide phosphatase/kinase	PNKP	11284	0.833	13	4	10	4	521	57	0.86374022
XP_005250972.1	RNA-binding protein 12B isoform X1	RBM12B	389677	0.709	4	3	10	3	1001	118	0.029580687
NP_116254.4	tensin-4 precursor	TNS4	84951	0.702	10	7	15	7	715	76.7	0.470634182
NP_085131.1	integrator complex subunit 5	INTS5	80789	1.15	6	5	11	5	1019	107.9	0.233293965
NP_060377.1	ragulator complex protein LAMTOR1	LAMTOR1	55004	1.369	16	2	12	2	161	17.7	0.041764638
NP_002196.4	integrin alpha-5 precursor	ITGA5	3678	1.408	7	7	21	7	1049	114.5	0.001000088
NP_055412.2	deoxynucleotidyltransferase terminal-interacting protein 2	DNTTIP2	30836	1.206	10	5	8	5	756	84.4	0.064082696
NP_001229536.1	neural cell adhesion molecule 1 isoform 5 precursor	NCAM1	4684	1.217	6	3	8	3	884	97.3	0.892580785
XP_005248611.1	erbin isoform X1	ERBIN	55914	0.989	6	5	12	5	1460	163.3	0.9904745
NP_060802.1	ATP-dependent RNA helicase DDX19A isoform 1	DDX19A	55308	0.751	16	6	13	6	478	53.9	0.302633838
NP_000160.1	alpha-galactosidase A precursor	GLA	2717	1.151	8	3	9	3	429	48.7	0.686531367
NP_689942.2	5'-nucleotidase domain-containing protein 1	NT5DC1	221294	0.573	21	5	15	5	455	51.8	0.090321322
NP_001305131.1	FH1/FH2 domain-containing protein 1 isoform 1	FHOD1	29109	0.58	8	5	9	5	1190	129.2	0.011352865
NP_006806.1	transmembrane emp24 domain-containing protein 2 isoform 1 precursor	TMED2	10959	1.336	31	4	21	4	201	22.7	0.207796621
XP_011541872.1	nucleotide exchange factor SIL1 isoform X1	SIL1	64374	1.012	12	5	13	5	471	53.2	0.956021652
NP_055448.1	RNA polymerase-associated protein CTR9 homolog isoform 1	CTR9	9646	1.213	6	5	9	5	1173	133.4	0.49791788
NP_110438.1	28S ribosomal protein S26, mitochondrial	MRPS26	64949	1.509	23	5	14	5	205	24.2	0.005642437
NP_056997.1	thioredoxin domain-containing protein 12 precursor	TXNDC12	51060	1.73	30	5	19	5	172	19.2	0.00180444
NP_000058.1	carbonic anhydrase 2 isoform 1	CA2	760	0.549	17	4	14	4	260	29.2	0.100594963
NP_000377.1	bleomycin hydrolase	BLMH	642	0.501	15	5	12	5	455	52.5	0.044630231

Patil et al,2020. Proteomic and phosphoproteomic profiling of shammah-induced signaling in oral keratinocytes

Supplementary Table S1: List of proteins identified and quantified in OKF6/TERT1-Shammah and OKF6/TERT1-Parental total proteome using TMT-based quantitative proteomic approach

Accession	Protein Description	Gene Symbol	Gene ID	Fold Change	Coverage [%]	# Peptides	# PSMs	# Unique Peptides	# AAs	MW [kDa]	p-value
NP_775813.2	putative phospholipase B-like 2 isoform 1 precursor	PLBD2	196463	0.901	8	4	10	4	589	65.4	0.812315928
XP_016857447.1	ras-related protein Rab-3B isoform X1	RAB3B	5865	1.59	17	4	22	1	219	24.7	0.000208543
NP_001262.3	chromodomain-helicase-DNA-binding protein 2 isoform 1	CHD2	1106	1.413	4	7	15	7	1828	211.2	0.619940366
XP_024302997.1	syntenin-1 isoform X1	SDCBP	6386	0.742	24	3	8	3	319	34.9	0.607921109
NP_001244047.1	caspase-1 isoform alpha precursor	CASP1	834	0.375	14	4	10	4	404	45.1	0.023585153
XP_011511369.1	D-beta-hydroxybutyrate dehydrogenase, mitochondrial isoform X1	BDH1	622	1.242	13	3	13	3	347	38.8	0.219143131
XP_005269076.1	5'-AMP-activated protein kinase subunit gamma-1 isoform X1	PRKAG1	5571	0.918	15	5	10	5	368	41.4	0.504349686
NP_112487.1	SRA stem-loop-interacting RNA-binding protein, mitochondrial isoform 1 precursor	SLIRP	81892	1.482	33	4	13	4	109	12.3	0.286080554
XP_005254606.1	beta-2-microglobulin isoform X1	B2M	567	0.764	19	3	13	3	122	14	0.209127516
XP_011508213.1	sterol O-acyltransferase 1 isoform X1	SOAT1	6646	1.17	8	3	10	3	550	64.7	0.126903431
NP_071413.1	Golgi phosphoprotein 3	GOLPH3	64083	0.347	20	4	10	4	298	33.8	0.448155269
NP_060692.3	alpha-parvin	PARVA	55742	0.73	12	4	11	4	372	42.2	0.124305063
NP_000700.1	2-oxoisovalerate dehydrogenase subunit alpha, mitochondrial isoform 1 precursor	BCKDHA	593	1.751	9	2	5	2	445	50.4	0.359011308
NP_057131.1	splicing factor 3B subunit 6	SF3B6	51639	1.19	28	3	16	3	125	14.6	0.120260098
XP_011535262.1	actin-related protein 10 isoform X1	ACTR10	55860	0.582	15	4	14	4	424	47.1	0.125677028
NP_057021.2	GPI transamidase component PIG-T isoform 1 precursor	PIGT	51604	1.357	12	6	13	6	578	65.7	0.251972825
NP_006859.2	ras-related protein Rab-31	RAB31	11031	1.732	27	4	11	2	195	21.7	0.352506186
NP_001030335.1	60S ribosomal protein L38	RPL38	6169	0.287	36	3	12	3	70	8.2	0.006023006
XP_024307826.1	GDP-fucose protein O-fucosyltransferase 2 isoform X1	POFUT2	23275	1.273	12	5	11	5	510	58.9	0.216688465
XP_011542496.1	methionine synthase isoform X1	MTR	4548	0.556	5	5	10	5	1321	145.6	0.025824451
NP_878256.1	geranylgeranyl transferase type-2 subunit alpha	RABGGTA	5875	0.675	12	5	12	5	567	65	0.099504428
XP_011531983.1	inositol 1,4,5-trisphosphate receptor type 1 isoform X1	ITPR1	3708	0.997	2	5	15	1	2759	313.9	0.491284542
NP_009165.1	dynactin subunit 3 isoform 1	DCTN3	11258	0.743	23	5	12	5	186	21.1	0.194395072
XP_016885151.1	THO complex subunit 2 isoform X1	THOC2	57187	0.879	4	5	16	5	1697	194.3	0.325299702
NP_060726.3	ribosome biogenesis protein WDR12	WDR12	55759	1.222	11	3	14	3	423	47.7	0.210816881
NP_001295169.1	MICOS complex subunit MIC13 isoform 1	C19orf70	125988	1.146	42	3	17	3	140	15.4	0.625607973
XP_011540792.1	cyclin-dependent kinase 11B isoform X1	CDK11B	984	1.271	7	5	11	5	804	93.5	0.411662476
NP_001157940.1	TRAM adaptor with GOLD domain isoform 1 precursor	TMED7-TICAM2	100302736	1.333	9	3	9	3	404	46.1	0.615661922
NP_001008273.1	transgelin-3	TAGLN3	29114	0.659	22	3	9	2	199	22.5	0.016338443
NP_001352154.1	ADP-dependent glucokinase isoform 4 precursor	ADPGK	83440	1.686	12	4	11	4	497	54.1	0.002839561
XP_006711023.1	volume-regulated anion channel subunit LRRC8C isoform X1	LRRC8C	84230	1.231	7	4	15	3	808	93	0.105321376
XP_006711593.1	specifically androgen-regulated gene protein isoform X1	C1orf116	79098	0.534	11	4	11	4	601	63.9	0.026295127
XP_005259970.1	lysosomal alpha-mannosidase isoform X1	MAN2B1	4125	1.731	6	5	12	5	1012	113.8	0.060075475
XP_005245844.1	ER membrane protein complex subunit 1 isoform X1	EMC1	23065	1.169	6	4	11	4	996	112	0.472651518
NP_004306.3	acid ceramidase isoform b	ASAH1	427	1.678	10	4	15	4	411	46.5	0.013073567
NP_060945.2	transmembrane protein 165 precursor	TMEM165	55858	1.599	20	4	11	4	324	34.9	0.159524198
NP_056993.2	neuroblastoma-amplified sequence	NBAS	51594	1.213	3	5	11	5	2371	268.4	0.811218588
NP_057004.1	40S ribosomal protein S27-like	RPS27L	51065	1.103	39	4	19	2	84	9.5	0.990187869
XP_011536945.1	replication factor C subunit 5 isoform X1	RFC5	5985	1.073	17	4	8	4	340	38.5	0.802269191
NP_001309859.1	double-stranded RNA-binding protein Staufen homolog 1 isoform b	STAU1	6780	0.95	12	6	13	6	577	63.1	0.778445693
XP_011536637.1	keratin, type II cuticular Hb4 isoform X1	KRT84	3890	1.46	4	5	64	1	600	64.8	0.634907685
XP_005265826.1	pre-mRNA 3'-end-processing factor FIP1 isoform X1	FIP1L1	81608	0.917	11	4	11	4	603	67.4	0.589063213
NP_001093115.1	60S ribosomal protein L22-like 1 isoform 1	RPL22L1	200916	0.592	45	4	9	4	122	14.6	0.341739949
XP_005264696.2	heterogeneous nuclear ribonucleoprotein L-like isoform X1	HNRNPLL	92906	1.196	9	4	14	3	595	66.4	0.826159736
NP_001272972.1	medium-chain specific acyl-CoA dehydrogenase, mitochondrial isoform d precursor	ACADM	34	0.614	18	5	11	5	454	50.2	0.376914017
XP_011530170.1	arfaptin-1 isoform X1	ARFIP1	27236	0.877	13	4	13	4	379	42.6	0.380764611
XP_011515831.1	tyrosine-protein kinase Lyn isoform X1	LYN	4067	1.007	8	5	13	4	682	76.6	0.985696856
XP_005247411.1	calcium-transporting ATPase type 2C member 1 isoform X1	ATP2C1	27032	1.967	7	5	9	5	983	108.1	0.038163777
NP_055271.2	programmed cell death protein 4 isoform 1	PDCD4	27250	0.618	16	5	13	5	469	51.7	0.100596522
XP_011533481.1	RNA-binding protein 26 isoform X1	RBM26	64062	1.199	8	7	12	6	1025	115.4	0.626521423
NP_477352.3	phosphatidylinositol 4-kinase alpha isoform 1	PI4KA	5297	0.544	3	4	8	4	2102	236.7	0.275015504
NP_001291479.1	THUMP domain-containing protein 1	THUMPD1	55623	0.986	12	5	15	5	353	39.3	0.992154589
NP_001273974.1	endophilin-B2 isoform a	SH3GLB2	56904	0.724	18	6	14	5	404	44.7	0.259592472
NP_001139411.1	platelet-activating factor acetylhydrolase IB subunit gamma	PAFAH1B3	5050	0.806	22	5	18	5	231	25.7	0.237269738
NP_078988.3	engulfment and cell motility protein 3	ELMO3	79767	0.464	8	4	11	4	720	81.4	0.00217792
NP_006339.3	conserved oligomeric Golgi complex subunit 5 isoform 1	COG5	10466	0.695	7	3	9	3	860	94.8	0.270089716
NP_056357.1	regulator of nonsense transcripts 2	UPF2	26019	0.606	4	4	10	4	1272	147.7	0.025430365
NP_002861.1	ras-related protein Rab-13 isoform 1	RAB13	5872	1.536	16	4	39	1	203	22.8	0.782778805
XP_005265184.1	laminin subunit beta-2 isoform X1	LAMB2	3913	0.932	4	4	11	4	1798	195.9	0.773641692
NP_078862.4	oxysterol-binding protein-related protein 9 isoform c	OSBPL9	114883	0.964	11	5	12	5	736	83.1	0.831005728
NP_001003800.1	protein bicaudal D homolog 2 isoform 1	BICD2	23299	0.87	9	5	9	5	855	96.7	0.774197352
NP_005717.3	elongation factor Ts, mitochondrial isoform 2 precursor	TSFM	10102	1.235	23	5	11	5	325	35.4	0.16040448
NP_076968.2	spermatogenesis-associated protein 5-like protein 1 isoform 1	SPATA5L1	79029	0.707	10	5	11	5	753	80.7	0.330358158
XP_005264383.1	protein Mpv17 isoform X1	MPV17	4358	1.561	18	2	9	2	176	19.7	0.320837087
NP_005246.2	cyclin-G-associated kinase isoform 1	GAK	2580	0.85	8	6	15	6	1311	143.1	0.425138925
NP_055578.2	39S ribosomal protein L19, mitochondrial	MRPL19	9801	1.297	20	6	14	6	292	33.5	0.400831172
NP_659486.2	ubiquitin-like domain-containing CTD phosphatase 1	UBLCP1	134510	0.376	14	5	13	5	318	36.8	0.039106478
NP_001139373.1	serine--tRNA ligase, mitochondrial isoform a precursor	SARS2	54938	0.845	13	4	9	4	520	58	0.339763164
NP_004026.2	peroxisomal acyl-coenzyme A oxidase 1 isoform a	ACOX1	51	2.421	12	4	10	4	660	74.6	0.001486182
NP_004069.1	cysteine and glycine-rich protein 1 isoform 1	CSRP1	1465	0.635	25	3	12	3	193	20.6	0.200051689
NP_003326.2	ubiquitin-like modifier-activating enzyme 7	UBA7	7318	0.764	6	3	8	3	1012	111.6	0.039417566
XP_005248298.1	NAD kinase 2, mitochondrial isoform X1	NADK2	133686	1.491	9	4	15	4	464	51.9	0.088144149
NP_004857.4	secretory carrier-associated membrane protein 1 isoform 1	SCAMP1	9522	1.197	16	4	10	4	338	37.9	0.593244843
NP_003809.1	phosphatidate cytidylyltransferase 2	CDS2	8760	1.262	12	3	8	3	445	51.4	0.808764957
NP_001351645.1	myosin phosphatase Rho-interacting protein isoform 3	MPRIP	23164	1.24	1	2	9	2	2457	277.4	0.034819465
NP_006010.2	V-type proton ATPase 116 kDa subunit a isoform 3 isoform a	TCIRG1	10312	1.111	7	4	9	4	830	92.9	0.869514489
NP_054797.2	39S ribosomal protein L13, mitochondrial	MRPL13	28998	1.224	19	3	12	3	178	20.7	0.422514887
XP_005265042.1	raftlin isoform X2	RFTN1	23180	1.533	11	5	10	5	578	63.1	0.230374772
NP_005461.2	abl interactor 1 isoform a	ABI1	10006	0.737	9	3	8	2	508	55	0.005144297
NP_002785.1	proteasome subunit beta type-2 isoform 1	PSMB2	5690	0.643	10	2	20	2	201	22.8	0.145849126
XP_011521284.1	N-acetylgalactosamine-6-sulfatase isoform X1	GALNS	2588	1.006	10	5	11	5	581	64.9	0.999143583
NP_071942.1	28S ribosomal protein S25, mitochondrial	MRPS25	64432	0.814	27	4	9	4	173	20.1	0.678555818
NP_056280.2	gem-associated protein 5 isoform 1	GEMIN5	25929	0.641	4	6	15	6	1508	168.5	0.043608894
XP_011533354.1	general transcription factor IIF subunit 2 isoform X1	GTF2F2	2963	0.87	29	5	8	5	275	30.9	0.66027076
NP_037364.1	programmed cell death protein 6 isoform 1	PDCD6	10016	0.954	32	4	15	4	191	21.9	0.841503746
NP_000055.2	complement C3 preproprotein	C3	718	1.551	5	6	12	6	1663	187	0.007740751
NP_473357.1	serine/arginine-rich splicing factor 10 isoform 2	SRSF10	10772	1.668	15	3	12	3	262	31.3	0.036260984
NP_056473.2	nucleolar complex protein 2 homolog	NOC2L	26155	1.22	7	5	14	5	749	84.9	0.184485484
NP_057662.3	remodeling and spacing factor 1	RSF1	51773	1.299	5	5	11	5	1441	163.7	0.270121258
NP_060579.3	poly(A) RNA polymerase, mitochondrial precursor	MTPAP	55149	1.141	11	4	9	4	582	66.1	0.707104058
NP_001277120.1	myeloid-associated differentiation marker	MYADM	91663	0.942	10	2	9	2	322	35.3	0.777428009
NP_001272.2	tubulin-folding cofactor B isoform 1	TBCB	1155	0.55	25	5	16	5	244	27.3	0.001349675
NP_001302460.1	60S ribosomal protein L26	RPL26	6154	0.552	30	6	25	1	145	17.2	0.0001159
XP_024302900.1	putative helicase MOV-10 isoform X1	MOV10	4343	0.929	7	5	10	5	1003	113.6	0.728691273
NP_001342338.1	histone cluster 2 H3 pseudogene 2	HIST2H3PS2	440686	2.683	35	6	39	1	136	15.4	0.064385544
NP_001186902.1	RPL36A-HNRNPH2 protein isoform a	RPL36A-HNRNPH2	100529097	0.898	18	5	14	5	154	17.7	0.031669115
XP_024309639.1	major facilitator superfamily domain-containing protein 10 isoform X1	MFSD10	10227	2.193	5	3	15	3	491	51.9	0.004732283
NP_000934.1	peptidyl-prolyl cis-trans isomerase C precursor	PPIC	5480	0.696	15	3	19	3	212	22.7	0.139490343
NP_057023.2	tRNA (adenine(58)-N(1))-methyltransferase non-catalytic subunit TRM6 isoform 1	TRMT6	51605	0.835	9	4	8	4	497	55.8	0.96602501
XP_011531194.1	protein MEMO1 isoform X1	MEMO1	51072	0.404	13	2	9	2	297	33.7	0.017298588
NP_004044.3	bystin	BYSL	705	1.088	14	6	11	6	437	49.6	0.693470768
NP_001340975.1	rho guanine nucleotide exchange factor 7 isoform i	ARHGEF7	8874	0.776	7	5	10	5	862	97.1	0.66351183
NP_001021.1	40S ribosomal protein S27 isoform 1	RPS27	6232	1.118	39	4	22	2	84	9.5	0.804927983
XP_016868703.1	protein-tyrosine kinase 2-beta isoform X1	PTK2B	2185	0.766	8	6	13	5	1009	115.8	0.595069942
XP_005255285.3	calcium-regulated heat-stable protein 1 isoform X1	CARHSP1	23589	0.278	14	2	12	2	168	18.1	0.000270314
XP_011511846.1	actin filament-associated protein 1 isoform X1	AFAP1	60312	1.966	5	4	10	4	814	89.7	0.09354308
XP_011539528.1	D-3-phosphoglycerate dehydrogenase isoform X1	PHGDH	26227	0.654	13	6	12	6	607	64.7	0.52636989
XP_011528279.1	adenylosuccinate lyase isoform X1	ADSL	158	0.537	12	5	12	5	526	59.3	0.16314813
NP_079498.2	WD repeat-containing protein 82	WDR82	80335	0.77	13	3	10	3	313	35.1	0.727566611
NP_001310871.1	methyl-CpG-binding domain protein 1 isoform 14	MBD1	4152	1.029	6	2	5	2	680	75.1	0.953866156
XP_005273905.1	peptidyl-prolyl cis-trans isomerase FKBP2 isoform X1	FKBP2	2286	1.446	39	5	12	5	142	15.6	0.000103176
XP_006717116.1	osteoclast-stimulating factor 1 isoform X1	OSTF1	26578	0.46	20	3	11	3	217	24	0.004679167
NP_002067.1	N-acetylglucosamine-6-sulfatase precursor	GNS	2799	1.085	8	4	15	4	552	62	0.693068996
NP_006825.1	ras-related protein Rab-32	RAB32	10981	1.272	18	4	9	2	225	25	0.500808173
NP_001305428.1	39S ribosomal protein L48, mitochondrial isoform 2	MRPL48	51642	1.665	9	2	9	2	253	28.2	0.004884824
NP_001138749.1	adaptin ear-binding coat-associated protein 2 isoform 2	NECAP2	55707	0.662	22	3	9	3	273	29.4	0.013046129
NP_003786.1	sorting nexin-3 isoform a	SNX3	8724	0.854	43	5	11	4	162	18.8	0.524560574
XP_005256486.1	chromodomain-helicase-DNA-binding protein 3 isoform X7	CHD3	1107	0.743	3	4	12	1	2060	233	0.026545733
NP_004392.1	DNA fragmentation factor subunit alpha isoform 1	DFFA	1676	0.957	20	5	9	5	331	36.5	0.802926667
NP_003110.1	paraplegin isoform 1 precursor	SPG7	6687	1.505	9	4	8	4	795	88.2	0.602333986
XP_011521388.1	diphosphomevalonate decarboxylase isoform X1	MVD	4597	0.744	12	3	8	3	434	47	0.717922398
NP_001138350.1	inositol monophosphatase 1 isoform 2	IMPA1	3612	0.717	21	5	12	5	336	36.7	0.13959646
NP_542763.1	sulfiredoxin-1	SRXN1	140809	0.738	35	2	4	2	137	14.3	0.863154634
NP_001032242.1	forkhead box protein K1	FOXK1	221937	1.023	5	3	12	2	733	75.4	0.895607051
NP_001327.2	cathepsin Z preproprotein	CTSZ	1522	2.288	14	5	12	5	303	33.8	0.000273255
NP_001034554.1	serine/arginine-rich splicing factor 5	SRSF5	6430	1.062	16	4	14	3	272	31.2	0.128563576
NP_005863.1	pre-mRNA-splicing factor SPF27	BCAS2	10286	1.096	26	4	11	4	225	26.1	0.622586563
NP_001678.1	ATP synthase subunit delta, mitochondrial precursor	ATP5F1D	513	1.925	14	2	13	2	168	17.5	0.029198015
XP_011530505.1	alpha-synuclein isoform X1	SNCA	6622	0.848	27	4	9	4	170	18.4	0.321265033
NP_112576.1	SH3 domain-binding glutamic acid-rich-like protein 3	SH3BGRL3	83442	0.847	20	2	11	2	93	10.4	0.33828832
NP_003162.2	ATP-dependent RNA helicase SUPV3L1, mitochondrial isoform 1	SUPV3L1	6832	1.601	8	4	8	4	786	87.9	0.093716945
NP_056073.1	FK506-binding protein 15	FKBP15	23307	0.842	5	5	10	5	1219	133.5	0.765108442
NP_003713.3	tumor protein 63 isoform 1	TP63	8626	1.219	7	4	7	4	680	76.7	0.69457289
NP_005326.2	hematopoietic lineage cell-specific protein isoform 1	HCLS1	3059	0.515	15	6	13	6	486	54	0.020033654
XP_005248173.1	transmembrane protein 33 isoform X1	TMEM33	55161	0.848	19	5	14	5	247	28	0.526334579
NP_694992.2	leucine-rich repeat-containing protein 57	LRRC57	255252	0.982	15	3	14	3	239	26.7	0.915256354
NP_001121093.1	UDP-glucose 4-epimerase	GALE	2582	0.413	19	4	10	4	348	38.3	0.073078014
NP_005331.1	histidine triad nucleotide-binding protein 1	HINT1	3094	0.287	19	2	9	2	126	13.8	0.004382261
XP_006720771.1	integrator complex subunit 14 isoform X1	INTS14	81556	0.984	15	5	6	5	550	60.8	0.473094019
XP_005251191.1	brefeldin A-inhibited guanine nucleotide-exchange protein 1 isoform X1	ARFGEF1	10565	0.573	4	5	14	3	1849	208.6	0.6250483
NP_004887.2	vacuolar protein sorting-associated protein 26A isoform 1	VPS26A	9559	0.671	18	4	12	4	327	38.1	0.375885405
NP_036560.1	neuroplastin isoform b precursor	NPTN	27020	1.375	13	4	11	4	398	44.4	0.606711424
NP_000231.1	amine oxidase	MAOA	4128	0.739	12	4	8	4	527	59.6	0.275940601
XP_005268284.1	serine/threonine-protein kinase MRCK beta isoform X1	CDC42BPB	9578	1.042	5	6	10	6	1728	196	0.842856469
NP_003817.1	gamma-soluble NSF attachment protein	NAPG	8774	0.901	10	3	11	3	312	34.7	0.733214251
XP_011520324.1	SAFB-like transcription modulator isoform X1	SLTM	79811	1.319	5	5	9	5	1176	132.4	0.142219927
NP_149098.2	GTP-binding protein 10 isoform 2	GTPBP10	85865	0.838	11	5	14	5	387	42.9	0.741969424
NP_001299801.1	cytochrome c oxidase assembly protein COX20, mitochondrial isoform 2	COX20	116228	1.193	21	2	8	2	130	14.7	0.688247093
NP_001252.1	cyclin-dependent kinase 9	CDK9	1025	1.319	12	4	11	3	372	42.8	0.222505968
NP_150597.1	28S ribosomal protein S36, mitochondrial	MRPS36	92259	1.161	42	3	9	3	103	11.5	0.443755677
XP_006710537.1	lysine-specific histone demethylase 1A isoform X1	KDM1A	23028	0.912	5	4	9	4	878	95.3	0.725102219
NP_004538.2	NADH dehydrogenase	NDUFB4	4710	1.731	41	4	12	4	129	15.2	0.011190951
XP_005249549.1	mediator of DNA damage checkpoint protein 1 isoform X1	MDC1	9656	1.455	4	5	11	5	2110	229	0.241547558
XP_005262416.1	cohesin subunit SA-2 isoform X1	STAG2	10735	0.984	4	5	11	4	1268	145.7	0.780300829
NP_055204.3	proline-, glutamic acid- and leucine-rich protein 1 isoform 1	PELP1	27043	0.857	4	4	13	4	1180	124.9	0.720485241
NP_001538.4	interferon-induced protein with tetratricopeptide repeats 2	IFIT2	3433	0.446	12	5	15	4	472	54.6	0.061136049
XP_016873006.1	triokinase/FMN cyclase isoform X2	TKFC	26007	0.691	8	4	10	4	621	64.1	0.259714747
NP_997198.2	UMP-CMP kinase 2, mitochondrial isoform 1 precursor	CMPK2	129607	0.634	12	4	6	4	449	49.4	0.377233862
NP_002309.1	lysyl oxidase homolog 2 precursor	LOXL2	4017	1.631	7	4	6	4	774	86.7	0.543158436
NP_002615.2	prefoldin subunit 5 isoform alpha	PFDN5	5204	0.573	26	3	8	3	154	17.3	0.472801854
NP_001317140.1	mediator of RNA polymerase II transcription subunit 24 isoform 3	MED24	9862	0.954	5	4	7	4	1008	112.1	0.87637946
NP_006017.1	histone H1x	H1FX	8971	2.065	10	3	10	3	213	22.5	0.543257761
NP_004695.1	U3 small nucleolar RNA-interacting protein 2	RRP9	9136	1.233	19	7	14	7	475	51.8	0.173511027
NP_036270.1	protein AATF	AATF	26574	1.174	9	3	7	3	560	63.1	0.910578223
NP_001035887.1	monocarboxylate transporter 4	SLC16A3	9123	1.926	6	3	20	3	465	49.4	0.001944054
XP_005264900.1	oxysterol-binding protein-related protein 10 isoform X1	OSBPL10	114884	0.848	5	3	15	2	769	84.3	0.035437715
XP_005248331.1	NAD(P) transhydrogenase, mitochondrial isoform X1	NNT	23530	1.101	5	4	10	4	1086	113.8	0.625014406
NP_006298.1	sushi repeat-containing protein SRPX isoform 1 precursor	SRPX	8406	1.484	8	3	10	3	464	51.5	0.213045858
NP_001015049.1	BAG family molecular chaperone regulator 5 isoform a	BAG5	9529	0.725	9	4	11	4	488	56	0.200306402
XP_016869431.1	disintegrin and metalloproteinase domain-containing protein 9 isoform X1	ADAM9	8754	1.364	5	2	8	2	821	91	0.049743221
NP_036526.2	prefoldin subunit 2	PFDN2	5202	0.591	26	3	14	3	154	16.6	0.001638089
NP_002479.1	NADH dehydrogenase	NDUFA2	4695	1.504	42	3	8	3	99	10.9	0.253216611
NP_777567.1	serine/threonine-protein phosphatase 4 regulatory subunit 2 isoform 1	PPP4R2	151987	0.998	10	3	8	3	417	46.9	0.821252609
NP_001825.1	clathrin light chain B isoform a	CLTB	1212	0.729	20	5	14	5	211	23.2	0.005906039
NP_006603.2	kinesin-like protein KIF1C	KIF1C	10749	0.814	4	4	9	2	1103	122.9	0.938106367
NP_003814.1	tumor necrosis factor receptor superfamily member 6B precursor	TNFRSF6B	8771	1.258	10	2	8	2	300	32.7	0.496298159
NP_006247.1	serine/threonine-protein kinase N2 isoform 1	PKN2	5586	0.754	5	4	9	4	984	112	0.000286263
XP_016859298.1	zinc finger protein 638 isoform X1	ZNF638	27332	1.242	5	6	10	6	2084	231.4	0.729732562
XP_016879084.1	28S ribosomal protein S34, mitochondrial isoform X1	MRPS34	65993	0.665	15	4	12	4	248	28.7	0.170391923
NP_001120793.1	chromobox protein homolog 5	CBX5	23468	1.221	32	5	9	5	191	22.2	0.387295796
NP_004444.2	electron transfer flavoprotein-ubiquinone oxidoreductase, mitochondrial isoform 1 precursor	ETFDH	2110	1.125	9	3	9	3	617	68.5	0.708263719
NP_055567.2	signal peptidase complex subunit 2	SPCS2	9789	1.302	23	5	15	5	226	25	0.274874641
XP_016866269.1	60S ribosomal protein L7-like 1 isoform X1	RPL7L1	285855	0.885	13	3	11	3	255	29.7	0.813058611
NP_001275897.1	acyl-CoA synthetase family member 2, mitochondrial isoform 1	ACSF2	80221	1.454	7	3	6	3	640	70.6	0.216003742
NP_001281269.1	zinc finger CCCH domain-containing protein 18 isoform 1	ZC3H18	124245	1.494	4	3	7	3	977	108.9	0.127289162
XP_011522151.1	lethal(2) giant larvae protein homolog 1 isoform X1	LLGL1	3996	0.808	5	4	7	4	1105	119.8	0.879496132
NP_003132.2	E3 ubiquitin-protein ligase TRIM21	TRIM21	6737	0.473	13	4	8	4	475	54.1	0.112709582
NP_000550.2	hemoglobin subunit gamma-1	HBG1	3047	2.155	17	3	29	3	147	16.1	0.005343915
NP_060309.2	lysophosphatidylcholine acyltransferase 2	LPCAT2	54947	1.002	10	3	8	3	544	60.2	0.920736116
NP_001070667.1	splicing regulatory glutamine/lysine-rich protein 1 isoform a	SREK1	140890	1.3	8	3	11	3	624	71.6	0.513870178
NP_057678.1	peptidyl-prolyl cis-trans isomerase FKBP11 isoform 1 precursor	FKBP11	51303	1.477	15	3	10	3	201	22.2	0.067511434
XP_024305360.1	cTAGE family member 5 isoform X1	MIA2	4253	1.766	3	4	8	4	1426	161.3	0.225840669
NP_001028740.1	mitochondrial Rho GTPase 1 isoform 1	RHOT1	55288	1.481	6	4	10	3	691	79.5	0.092063393
NP_004569.2	ras-related protein Rab-4A isoform 1	RAB4A	5867	0.992	22	4	17	3	218	24.4	0.894186104
NP_852480.1	exosome complex component RRP43	EXOSC8	11340	1.073	27	5	11	5	276	30	0.82559696
NP_056169.1	nucleoporin NUP188 homolog	NUP188	23511	0.854	3	3	10	3	1749	195.9	0.981937423
NP_038466.2	ubiquilin-1 isoform 1	UBQLN1	29979	0.999	5	2	9	1	589	62.5	0.658280911
XP_005263429.1	lipopolysaccharide-responsive and beige-like anchor protein isoform X1	LRBA	987	0.76	1	4	11	4	2868	319.5	0.638206015
NP_006510.1	dynein light chain Tctex-type 1 isoform 1	DYNLT1	6993	0.597	30	2	8	2	113	12.4	0.022627686
NP_071383.1	28S ribosomal protein S14, mitochondrial	MRPS14	63931	1.857	17	2	8	2	128	15.1	0.619828553
NP_065916.2	ATP-dependent RNA helicase DHX36 isoform 1	DHX36	170506	0.456	5	4	7	4	1008	114.7	0.344390682
NP_001333694.1	unconventional myosin-XVIIIa isoform c	MYO18A	399687	0.884	3	5	9	5	2058	233.1	0.814359037
NP_057267.2	mRNA turnover protein 4 homolog	MRTO4	51154	0.951	35	5	14	5	239	27.5	0.876342045
XP_005269346.1	disks large homolog 1 isoform X1	DLG1	1739	1.39	7	5	11	5	926	103.3	0.030527305
NP_001008895.1	cullin-4A isoform 1	CUL4A	8451	1.281	9	6	11	3	759	87.6	0.875318996
NP_005056.3	protein sel-1 homolog 1 isoform 1 precursor	SEL1L	6400	1.195	7	3	6	3	794	88.7	0.862810054
NP_008824.2	eukaryotic translation initiation factor 2D isoform 1	EIF2D	1939	0.764	12	3	3	3	584	64.7	0.983870541
NP_057191.2	zinc finger RNA-binding protein	ZFR	51663	1.225	5	4	9	4	1074	116.9	0.900718869
XP_024309519.1	F-box-like/WD repeat-containing protein TBL1XR1 isoform X1	TBL1XR1	79718	1.093	10	5	11	5	514	55.6	0.690055465
NP_849193.1	dolichyl-diphosphooligosaccharide--protein glycosyltransferase subunit STT3B	STT3B	201595	1.579	8	7	17	7	826	93.6	0.195363056
NP_001317259.1	luc7-like protein 3 isoform 2	LUC7L3	51747	1.028	9	4	11	4	489	58.2	0.933883596
NP_071353.4	HEAT repeat-containing protein 6	HEATR6	63897	0.599	4	3	8	3	1181	128.7	0.091967008
NP_001306161.1	60S ribosomal protein L34	RPL34	6164	0.872	28	4	19	4	117	13.3	0.431822272
NP_054737.1	28S ribosomal protein S28, mitochondrial	MRPS28	28957	1.109	23	3	10	3	187	20.8	0.618782711
NP_055417.1	phosphoinositide 3-kinase regulatory subunit 4	PIK3R4	30849	0.775	3	3	10	3	1358	153	0.601910261
NP_056345.3	Golgi reassembly-stacking protein 2 isoform 1	GORASP2	26003	1.475	16	5	8	5	452	47.1	0.325516829
NP_659471.1	torsin-1A-interacting protein 2 isoform b	TOR1AIP2	163590	1.044	10	3	7	2	470	51.2	0.964540311
XP_005274491.1	N-acetylserotonin O-methyltransferase-like protein isoform X1	ASMTL	8623	0.854	6	3	8	3	621	68.8	0.676351562
NP_036425.1	peroxidasin homolog precursor	PXDN	7837	2.128	5	5	13	5	1479	165.2	0.002614099
NP_002846.3	nectin-1 isoform 1 precursor	NECTIN1	5818	1.66	9	3	8	3	517	57.1	0.002047732
NP_058431.2	adapter molecule crk isoform a	CRK	1398	0.486	11	2	9	2	304	33.8	0.001517863
NP_001305699.1	39S ribosomal protein L14, mitochondrial isoform c	MRPL14	64928	1.339	19	3	11	3	198	22.1	0.387770811
NP_078772.1	interferon regulatory factor 2-binding protein-like	IRF2BPL	64207	0.847	6	3	8	3	796	82.6	0.306795751
NP_001273345.1	small ubiquitin-related modifier 3 isoform 2	SUMO3	6612	0.916	18	2	10	2	141	15.8	0.799277792
NP_037368.1	ataxin-10 isoform 1	ATXN10	25814	0.549	10	5	14	5	475	53.5	0.258809053
NP_054899.2	39S ribosomal protein L22, mitochondrial isoform a	MRPL22	29093	1.442	20	4	8	4	206	23.6	0.801670497
XP_016883513.1	proto-oncogene tyrosine-protein kinase Src isoform X1	SRC	6714	1.654	11	5	11	2	542	60.6	0.913964692
NP_001746.1	core-binding factor subunit beta isoform 2	CBFB	865	0.686	20	3	7	3	182	21.5	0.60939356
NP_057149.1	28S ribosomal protein S16, mitochondrial	MRPS16	51021	1.061	53	5	9	5	137	15.3	0.649664379
NP_940916.2	NHL repeat-containing protein 2	NHLRC2	374354	0.66	10	4	13	4	726	79.4	0.778385064
XP_024309873.1	OCIA domain-containing protein 1 isoform X1	OCIAD1	54940	1.337	23	4	9	4	260	29.1	0.09730075
NP_006701.1	COP9 signalosome complex subunit 8 isoform 1	COPS8	10920	0.719	18	3	8	3	209	23.2	0.714661731
XP_011519908.2	unconventional myosin-Va isoform X1	MYO5A	4644	0.795	3	4	9	4	1904	221.2	0.608601189
NP_006283.1	tumor susceptibility gene 101 protein	TSG101	7251	0.836	13	4	12	4	390	43.9	0.399055286
NP_065142.2	39S ribosomal protein L47, mitochondrial isoform a	MRPL47	57129	1.591	17	4	11	4	250	29.4	0.007619573
XP_011524044.1	nucleoporin SEH1 isoform X1	SEH1L	81929	1.077	12	4	11	4	441	49	0.370534362
XP_005253433.1	cation-dependent mannose-6-phosphate receptor isoform X1	M6PR	4074	1.208	18	4	10	4	277	31	0.080130781
NP_003008.1	serine/arginine-rich splicing factor 3	SRSF3	6428	1.632	23	4	18	3	164	19.3	0.002900272
NP_071896.8	nucleolar complex protein 3 homolog	NOC3L	64318	1.105	5	4	9	4	800	92.5	0.701237326
NP_001254747.1	oligosaccharyltransferase complex subunit OSTC isoform 3	OSTC	58505	1.797	7	1	12	1	171	19.2	0.000115569
NP_001124151.1	eukaryotic translation initiation factor 4E isoform 2	EIF4E	1977	0.651	18	5	17	5	248	28.8	0.106751823
NP_060310.1	39S ribosomal protein L16, mitochondrial	MRPL16	54948	1.537	16	3	11	3	251	28.4	0.219845994
XP_011532519.1	leucine-rich repeat flightless-interacting protein 2 isoform X1	LRRFIP2	9209	0.73	6	3	9	3	721	82.1	0.008176244
XP_006710865.2	ATP-binding cassette sub-family D member 3 isoform X1	ABCD3	5825	1.815	8	4	11	4	683	78.4	0.008075815
NP_004291.1	low molecular weight phosphotyrosine protein phosphatase isoform c	ACP1	52	0.624	33	5	10	5	158	18	0.160751175
NP_001306889.1	ER degradation-enhancing alpha-mannosidase-like protein 3 isoform 1 precursor	EDEM3	80267	1.311	5	4	8	4	948	106.3	0.261067056
XP_016865008.1	60S ribosomal protein L26-like 1 isoform X1	RPL26L1	51121	0.816	30	6	22	1	145	17.2	0.838122163
NP_055693.4	pumilio homolog 3	PUM3	9933	1.02	8	4	10	4	648	73.5	0.989028731
XP_024308055.1	activating signal cointegrator 1 complex subunit 2 isoform X1	ASCC2	84164	0.728	8	4	7	4	816	92.7	0.478158375
NP_055680.3	condensin complex subunit 1	NCAPD2	9918	0.531	4	4	9	4	1401	157.1	0.020921492
NP_001244941.2	NADPH:adrenodoxin oxidoreductase, mitochondrial isoform 3 precursor	FDXR	2232	1.195	18	6	17	6	534	58.2	0.69709051
NP_060357.1	UPF0587 protein C1orf123 isoform 1	C1orf123	54987	0.633	35	3	7	3	160	18	0.491684654
NP_001337011.1	sterile alpha motif domain-containing protein 9-like	SAMD9L	219285	0.338	3	4	8	3	1584	184.4	0.130118372
NP_060082.2	zinc finger CCHC domain-containing protein 8 isoform 1	ZCCHC8	55596	1.077	8	3	6	3	707	78.5	0.823907598
NP_932332.1	glucosamine 6-phosphate N-acetyltransferase	GNPNAT1	64841	0.951	23	3	9	3	184	20.7	0.729573223
XP_024302098.1	cytochrome c oxidase subunit 7A2, mitochondrial isoform X1	COX7A2	1347	1.021	20	2	12	2	115	12.8	0.646586351
NP_110416.1	minor histocompatibility antigen H13 isoform 1	HM13	81502	2.04	11	4	19	4	377	41.5	0.003915508
NP_001303991.1	methylosome protein 50 isoform 1	WDR77	79084	0.971	10	4	11	4	354	38	0.976766911
XP_016878013.1	tight junction protein ZO-1 isoform X5	TJP1	7082	0.846	5	6	11	6	1798	201.8	0.035596079
NP_110440.1	caseinolytic peptidase B protein homolog isoform 1	CLPB	81570	1.163	9	4	5	4	707	78.7	0.918951984
XP_011532575.1	DDB1- and CUL4-associated factor 1 isoform X1	DCAF1	9730	1.267	4	4	8	4	1507	168.9	0.112220636
NP_001186558.1	myosin light chain 6B	MYL6B	140465	0.703	16	3	19	1	208	22.8	0.654665124
NP_001137407.1	peroxisomal carnitine O-octanoyltransferase isoform 1	CROT	54677	0.905	8	3	9	3	640	73.3	0.725284728
NP_073750.2	28S ribosomal protein S11, mitochondrial isoform a	MRPS11	64963	1.766	14	2	6	2	194	20.6	0.000166272
NP_976035.1	DNA-directed RNA polymerases I and III subunit RPAC1 isoform 1	POLR1C	9533	1.065	18	4	8	4	346	39.2	0.922836826
XP_016863193.1	alpha-adducin isoform X2	ADD1	118	0.967	6	3	7	3	768	84.3	0.971637679
NP_056281.1	tyrosine-protein phosphatase non-receptor type 23 isoform 1	PTPN23	25930	0.722	5	5	12	5	1636	178.9	0.329056955
NP_055744.2	FAST kinase domain-containing protein 2, mitochondrial	FASTKD2	22868	1.081	8	4	10	4	710	81.4	0.748932921
NP_001123579.1	kinesin light chain 1 isoform 3	KLC1	3831	0.703	10	6	12	4	618	69.7	0.446191829
XP_011539174.1	endothelin-converting enzyme 1 isoform X1	ECE1	1889	1	6	3	8	3	778	88.3	0.417679614
NP_055051.1	dihydroxyacetone phosphate acyltransferase isoform 1	GNPAT	8443	0.704	10	5	10	5	680	77.1	0.28095192
NP_001014433.1	protein CutA isoform 1	CUTA	51596	0.847	16	2	9	2	198	20.9	0.34138081
NP_001488.2	beta-1,4-galactosyltransferase 1	B4GALT1	2683	0.646	15	4	13	4	398	43.9	0.055065716
NP_004081.1	dual specificity protein phosphatase 3	DUSP3	1845	0.75	19	3	6	3	185	20.5	0.509893177
NP_001300950.1	unhealthy ribosome biogenesis protein 2 homolog	URB2	9816	1.059	4	4	10	4	1524	170.4	0.824814136
NP_056238.2	L-aminoadipate-semialdehyde dehydrogenase-phosphopantetheinyl transferase	AASDHPPT	60496	0.555	14	4	15	4	309	35.8	0.007221498
XP_024308587.1	DNA mismatch repair protein Msh6 isoform X1	MSH6	2956	0.851	4	4	10	4	1391	156.4	0.809097217
NP_036602.1	transportin-3 isoform 1	TNPO3	23534	0.43	6	3	10	3	923	104.1	0.357033263
NP_001035830.1	glycerol-3-phosphate phosphatase	PGP	283871	0.639	19	4	14	4	321	34	0.010310101
NP_004992.2	NADH dehydrogenase	NDUFA7	4701	1.722	38	4	6	4	113	12.5	0.29492582
NP_003303.2	thiosulfate sulfurtransferase	TST	7263	1.431	15	4	10	4	297	33.4	0.084221711
NP_055050.1	ubiquitin-like protein 4A	UBL4A	8266	0.723	31	4	10	4	157	17.8	0.068838708
XP_011543382.1	serine/threonine-protein kinase PAK 1 isoform X2	PAK1	5058	0.948	7	3	9	1	553	61.6	0.90369657
NP_004652.2	cell division cycle protein 23 homolog	CDC23	8697	0.873	9	5	10	5	597	68.8	0.253938579
NP_064583.2	coiled-coil domain-containing protein 47 precursor	CCDC47	57003	1.19	10	4	8	4	483	55.8	0.772723392
XP_024303171.1	endoplasmic reticulum mannosyl-oligosaccharide 1,2-alpha-mannosidase isoform X1	MAN1B1	11253	0.946	5	3	8	3	754	83.9	0.759453561

Patil et al,2020. Proteomic and phosphoproteomic profiling of shammah-induced signaling in oral keratinocytes											
Supplementary Table S1: List of proteins identified and quantified in OKF6/TERT1-Shammah and OKF6/TERT1-Parental total proteome using TMT-based quantitative proteomic approach											
Accession	Protein Description	Gene Symbol	Gene ID	Fold Change	Coverage [%]	# Peptides	# PSMs	# Unique Peptides	# AAs	MW [kDa]	p-value
XP_024302907.1	cleavage and polyadenylation specificity factor subunit 1 isoform X1	CPSF1	29894	1.214	5	5	9	5	1469	163.7	0.812625265
NP_001304653.1	nucleolar protein 7	NOL7	51406	1.437	12	3	5	3	257	29.4	0.9518499
NP_065118.2	RNA exonuclease 4 isoform 1	REXO4	57109	1.386	14	4	9	4	422	46.6	0.68965051
NP_005995.2	cytochrome b-c1 complex subunit 6, mitochondrial isoform 1 precursor	UQCRH	7388	2.334	29	2	6	2	91	10.7	0.013209689
XP_016867428.1	sulfatase-modifying factor 2 isoform X1	SUMF2	25870	1.093	7	2	7	2	371	41.8	0.801044905
NP_079087.3	cleavage and polyadenylation specificity factor subunit 7 isoform 1	CPSF7	79869	1.357	9	5	17	5	514	56.3	0.003930308
NP_054765.1	28S ribosomal protein S18b, mitochondrial	MRPS18B	28973	1.177	20	4	6	4	258	29.4	0.797844227
XP_024303906.1	supervillin isoform X1	SVIL	6840	1.099	3	4	7	4	2334	261.2	0.992135721
NP_057308.1	sorting nexin-9	SNX9	51429	0.599	11	3	6	3	595	66.6	0.448701243
NP_063937.2	glycogen synthase kinase-3 alpha	GSK3A	2931	0.971	10	3	7	3	483	50.9	0.778287378
NP_001027581.1	2'-5'-oligoadenylate synthase 1 isoform 3	OAS1	4938	0.768	10	3	5	3	414	47.4	0.484780076
NP_005435.1	CCR4-NOT transcription complex subunit 9 isoform 2	CNOT9	9125	0.269	12	3	5	3	299	33.6	0.368891099
NP_006149.2	neurofilament light polypeptide	NEFL	4747	1.058	6	3	22	2	543	61.5	0.722198587
NP_057584.2	protein PBDC1 isoform 1	PBDC1	51260	0.478	18	3	10	3	233	26	0.013252888
NP_001007562.1	immunity-related GTPase family Q protein	IRGQ	126298	0.647	8	3	8	3	623	62.7	0.009965264
NP_000604.1	hemopexin precursor	HPX	3263	1.395	10	3	7	3	462	51.6	0.301156542
XP_005246998.1	N-myc-interactor isoform X1	NMI	9111	0.33	16	4	9	4	307	35	0.026446339
NP_002619.1	profilin-2 isoform b	PFN2	5217	0.536	32	4	13	4	140	15.1	0.107813577
XP_005268417.1	protein FAM114A2 isoform X1	FAM114A2	10827	0.597	10	3	7	3	505	55.4	0.538979892
NP_001686.1	V-type proton ATPase subunit C 1	ATP6V1C1	528	0.749	13	5	8	5	382	43.9	0.615019045
XP_005267899.1	serine/threonine-protein phosphatase 4 regulatory subunit 3A isoform X1	PPP4R3A	55671	1.311	4	4	11	4	833	95.3	0.528362863
NP_001545.2	protein CYR61 precursor	CYR61	3491	1.438	10	4	7	4	381	42	0.309469203
NP_001317392.1	atlastin-2 isoform 8	ATL2	64225	1.29	9	4	10	3	584	66.7	0.858018146
NP_115649.1	transmembrane protein 126A isoform 1	TMEM126A	84233	0.663	10	1	6	1	195	21.5	0.000185175
NP_001070993.1	myotubularin-related protein 14 isoform 2	MTMR14	64419	0.59	8	2	8	2	650	72.2	0.069727701
NP_115545.3	acyl-CoA dehydrogenase family member 11	ACAD11	84129	1.456	9	5	13	5	780	87.2	0.008957616
NP_002737.2	mitogen-activated protein kinase 3 isoform 1	MAPK3	5595	0.922	12	5	16	2	379	43.1	0.07996183
NP_062826.2	N6-adenosine-methyltransferase catalytic subunit	METTL3	56339	0.906	7	3	8	3	580	64.4	0.697691678
XP_024306618.1	zinc phosphodiesterase ELAC protein 2 isoform X1	ELAC2	60528	0.902	9	5	8	5	879	97.7	0.526744877
NP_115544.1	WD repeat-containing protein 75 isoform 1	WDR75	84128	1.182	7	5	9	5	830	94.4	0.094151223
NP_078837.3	N-alpha-acetyltransferase 16, NatA auxiliary subunit isoform 1	NAA16	79612	0.354	5	4	9	1	864	101.4	0.042920333
NP_000840.2	glutathione S-transferase Mu 3	GSTM3	2947	0.529	22	4	14	3	225	26.5	0.038362807
NP_005644.2	alpha-globin transcription factor CP2 isoform 1	TFCP2	7024	0.949	10	3	9	3	502	57.2	0.484574162
NP_000299.2	lysosomal protective protein isoform a precursor	CTSA	5476	1.148	13	5	11	5	498	56.2	0.502840796
XP_005268224.1	AP-1 complex subunit gamma-like 2 isoform X1	AP1G2	8906	0.692	6	4	8	4	842	93.6	0.18670527
NP_149061.1	haloacid dehalogenase-like hydrolase domain-containing 5 isoform 2 precursor	HDHD5	27440	1.224	6	1	4	1	423	46.3	0.680201046
NP_001070883.1	cysteine-rich with EGF-like domain protein 1 isoform 2 precursor	CRELD1	78987	1.806	6	3	10	3	420	45.4	0.130903773
XP_011540102.1	type-1 angiotensin II receptor-associated protein isoform X1	AGTRAP	57085	1.24	13	1	6	1	171	18.7	0.030327436
NP_056415.1	monoacylglycerol lipase ABHD12 isoform b	ABHD12	26090	1.161	10	4	9	4	404	45.5	0.576145808
NP_079229.2	N-alpha-acetyltransferase 25, NatB auxiliary subunit	NAA25	80018	0.631	6	4	6	4	972	112.2	0.296430824
NP_055819.2	exosome complex component RRP42	EXOSC7	23016	1.17	9	2	6	2	291	31.8	0.377708562
XP_011531902.1	EGF domain-specific O-linked N-acetylglucosamine transferase isoform X1	EOGT	285203	1.209	7	4	10	4	545	63.8	0.564041735
XP_005271903.1	ribosome biogenesis protein BMS1 homolog isoform X1	BMS1	9790	1.651	4	4	9	4	1282	145.7	0.094401839
NP_064516.2	ubiquilin-4 isoform 1	UBQLN4	56893	0.793	5	2	8	1	601	63.8	0.063248728
NP_071505.2	nucleolysin TIA-1 isoform p40 isoform 2	TIA1	7072	0.487	10	3	10	1	386	42.9	0.031963926
XP_005251870.1	E3 ubiquitin-protein ligase TRIM32 isoform X1	TRIM32	22954	1.121	6	3	10	3	653	71.9	0.303697382
XP_005268638.1	protein OS-9 isoform X1	OS9	10956	1.06	6	3	8	3	668	75.6	0.559990412
XP_006723917.1	engulfment and cell motility protein 2 isoform X1	ELMO2	63916	0.794	3	1	5	1	720	82.6	0.583529528
XP_016864032.1	condensin complex subunit 3 isoform X1	NCAPG	64151	0.49	4	4	10	4	1017	114.5	0.266557341
NP_001291937.1	endoplasmic reticulum junction formation protein lunapark isoform 1 precursor	LNPK	80856	2.105	7	3	8	3	494	54.8	0.041810709
NP_057071.2	heme-binding protein 1	HEBP1	50865	0.864	28	4	8	4	189	21.1	0.982663462
NP_001003701.1	ATP synthase-coupling factor 6, mitochondrial isoform b precursor	ATP5PF	522	1.881	29	3	11	3	116	13.4	0.000995429
NP_076997.1	transmembrane protein 109 precursor	TMEM109	79073	1.704	9	2	10	2	243	26.2	0.001784648
NP_006745.1	synaptophysin-like protein 1 isoform a	SYPL1	6856	1.156	10	3	4	3	259	28.5	0.808334129
NP_001161419.1	mitochondrial import inner membrane translocase subunit Tim17-B isoform 1	TIMM17B	10245	1.544	20	2	5	2	222	23.8	0.60827238
NP_000311.2	dihydropteridine reductase isoform 1	QDPR	5860	0.978	17	3	8	3	244	25.8	0.882346538
XP_011517164.1	E3 ubiquitin-protein ligase BRE1A isoform X1	RNF20	56254	1.165	3	3	8	3	975	113.6	0.569925541
XP_024304387.1	N-terminal kinase-like protein isoform X1	SCYL1	57410	0.706	5	2	7	2	847	93.9	0.178811585
NP_008846.2	protein XRP2	RP2	6102	1.262	6	3	11	3	350	39.6	0.213042894
NP_543010.3	D-aminoacyl-tRNA deacylase 1 isoform 2	DTD1	92675	0.867	15	2	7	2	209	23.4	0.723424262
NP_001257368.1	actin-related protein 2/3 complex subunit 5 isoform 2	ARPC5	10092	0.52	25	3	9	3	154	16.6	0.000249809
NP_060427.2	epsin-3	EPN3	55040	1.111	6	3	8	1	632	68.2	0.980169328
XP_011515093.1	protein NDRG1 isoform X1	NDRG1	10397	0.842	12	3	7	3	411	44.7	0.568542611
NP_003783.1	endothelial differentiation-related factor 1 isoform alpha	EDF1	8721	0.609	28	5	16	5	148	16.4	0.116272151
NP_647473.1	ATP-dependent zinc metalloprotease YME1L1 isoform 1	YME1L1	10730	1.821	5	3	7	3	773	86.4	0.39976831
XP_024308564.1	acyl-protein thioesterase 2 isoform X1	LYPLA2	11313	0.789	18	3	12	3	287	30.5	0.749486907
NP_001022.1	40S ribosomal protein S28	RPS28	6234	0.827	30	2	14	2	69	7.8	0.483219891
NP_003469.2	cullin-5	CUL5	8065	0.741	5	3	7	3	780	90.9	0.079347765
NP_003016.1	endophilin-A2 isoform 1	SH3GL1	6455	0.455	12	4	11	4	368	41.5	0.030281595
NP_076962.2	nucleoporin Nup37	NUP37	79023	1.1	17	5	11	5	326	36.7	0.547619735
XP_011509255.1	3-hydroxyisobutyryl-CoA hydrolase, mitochondrial isoform X1	HIBCH	26275	1.558	11	4	17	4	386	43.5	0.112776542
XP_005245421.1	transcriptional repressor p66-beta isoform X1	GATAD2B	57459	1.368	7	3	6	3	593	65.2	0.79770906
NP_057588.1	39S ribosomal protein L27, mitochondrial	MRPL27	51264	1.252	26	3	8	3	148	16.1	0.550447935
XP_011539137.1	guanylate-binding protein 6 isoform X1	GBP6	163351	0.583	4	2	9	1	633	72.4	0.034846098
NP_001273737.1	protein PRRC1 isoform 1	PRRC1	133619	0.514	9	3	5	3	462	48.9	0.364453553
NP_001139730.1	phosphatidylinositol 5-phosphate 4-kinase type-2 gamma isoform a	PIP4K2C	79837	0.964	11	5	14	5	421	47.3	0.73679383
XP_016871651.1	annexin A7 isoform X1	ANXA7	310	0.839	10	4	15	4	488	52.7	0.631583897
NP_063940.1	diablo homolog, mitochondrial isoform 1 precursor	DIABLO	56616	1.864	22	3	7	3	239	27.1	0.140058789
NP_006741.1	beta-2-syntrophin	SNTB2	6645	1.392	8	5	9	5	540	57.9	0.746523054
NP_001265138.1	nuclear pore complex protein Nup153 isoform 1	NUP153	9972	1.399	4	4	5	4	1506	157.2	0.825949008
NP_001350808.1	TATA element modulatory factor isoform 2	TMF1	7110	1.236	6	5	5	5	1096	123.1	0.888798968
XP_011516742.1	rab GTPase-activating protein 1 isoform X1	RABGAP1	23637	0.402	5	4	8	3	1069	121.7	0.000158298
NP_060587.8	WD repeat-containing protein 11	WDR11	55717	0.934	4	4	9	4	1224	136.6	0.721572742
NP_073742.2	axin interactor, dorsalization-associated protein	AIDA	64853	0.975	18	5	8	5	306	35	0.857009025
NP_006747.1	transcription elongation factor A protein 1 isoform 1	TCEA1	6917	0.712	13	3	8	3	301	33.9	0.06672308
NP_068747.1	signal peptidase complex subunit 3	SPCS3	60559	1.327	12	2	8	2	180	20.3	0.168137612
NP_065797.2	mitochondrial antiviral-signaling protein isoform 1	MAVS	57506	1.086	9	3	8	3	540	56.5	0.679254151
NP_001658.2	ADP-ribosylation factor-like protein 2 isoform 1	ARL2	402	0.711	18	3	8	3	184	20.9	0.413036612
NP_005862.1	survival of motor neuron-related-splicing factor 30	SMNDC1	10285	1.117	13	2	9	2	238	26.7	0.601300244
NP_002791.1	proteasome subunit beta type-9 precursor	PSMB9	5698	0.555	19	3	9	3	219	23.3	0.029154231
NP_116258.2	nudC domain-containing protein 1 isoform 1	NUDCD1	84955	0.63	9	4	11	4	583	66.7	0.035137393
XP_006717229.1	nucleolar protein 8 isoform X1	NOL8	55035	1.56	5	4	8	4	1167	131.5	0.372963792
NP_006828.2	COP9 signalosome complex subunit 5	COPS5	10987	0.488	15	5	15	5	334	37.6	0.087523569
NP_068746.2	translation factor GUF1, mitochondrial isoform 1	GUF1	60558	0.866	6	2	6	2	669	74.3	0.042552816
NP_060561.3	elongator complex protein 3 isoform 1	ELP3	55140	0.52	4	2	8	2	547	62.2	0.025328067
XP_016859259.1	polypeptide N-acetylgalactosaminyltransferase 3 isoform X1	GALNT3	2591	0.961	10	4	9	4	633	72.6	0.997979971
NP_005981.3	serine/threonine-protein kinase 10	STK10	6793	0.598	5	4	15	4	968	112.1	0.002568681
NP_066957.3	39S ribosomal protein L23, mitochondrial	MRPL23	6150	1.21	35	4	12	4	153	17.8	0.001446661
NP_112486.1	queuine tRNA-ribosyltransferase catalytic subunit 1	QTRT1	81890	0.861	14	3	11	3	403	44	0.774231353
NP_835461.2	E3 ubiquitin-protein ligase ZNF598	ZNF598	90850	0.721	5	4	8	4	904	98.6	0.040838914
NP_004172.2	ubiquitin carboxyl-terminal hydrolase isozyme L1	UCHL1	7345	1.635	20	4	12	4	223	24.8	0.051154042
NP_001308658.1	exocyst complex component 6B isoform 1	EXOC6B	23233	0.685	5	3	6	3	815	94.6	0.379405999
NP_665912.1	high mobility group protein HMG-I/HMG-Y isoform b	HMGA1	3159	2.028	23	3	9	1	96	10.7	0.942540337
XP_005252123.1	methylglutaconyl-CoA hydratase, mitochondrial isoform X1	AUH	549	1.248	10	2	7	2	349	36.8	0.643963561
NP_000181.2	porphobilinogen deaminase isoform 1	HMBS	3145	0.544	16	3	9	3	361	39.3	0.000931179
NP_001313309.1	heme-binding protein 2 isoform 1	HEBP2	23593	0.606	14	3	9	3	216	24.2	0.001198885
NP_001005339.1	regulator of G-protein signaling 10 isoform a	RGS10	6001	0.657	16	2	7	2	181	21.2	0.210504565
NP_665807.1	myotrophin	MTPN	136319	0.745	17	2	5	2	118	12.9	0.745619097
XP_005246722.1	telomere-associated protein RIF1 isoform X1	RIF1	55183	1.236	1	3	6	3	2472	274.3	0.875828618
NP_001135858.1	pyruvate dehydrogenase kinase, isozyme 3 isoform 1 precursor	PDK3	5165	1.366	8	2	5	2	415	48	0.479738502
NP_037365.2	STE20/SPS1-related proline-alanine-rich protein kinase	STK39	27347	0.762	10	4	5	4	545	59.4	0.761416215
NP_942590.1	nucleoporin Nup43	NUP43	348995	1.065	14	4	9	4	380	42.1	0.943076639
XP_024302523.1	inosine-5'-monophosphate dehydrogenase 1 isoform X1	IMPDH1	3614	0.579	5	3	8	2	621	66.1	0.083070411
NP_062535.2	YLP motif-containing protein 1	YLPM1	56252	1.02	2	3	8	3	2146	241.5	0.986562687
NP_009139.1	39S ribosomal protein L3, mitochondrial	MRPL3	11222	1.417	9	3	7	3	348	38.6	0.553033062
NP_001776.1	cytidine deaminase	CDA	978	0.706	36	3	5	3	146	16.2	0.742676142
NP_002650.1	urokinase plasminogen activator surface receptor isoform 1 precursor	PLAUR	5329	1.076	13	2	6	2	335	37	0.413227479
NP_065090.1	PEST proteolytic signal-containing nuclear protein isoform a	PCNP	57092	0.587	17	2	6	2	178	18.9	0.338751108
NP_853553.1	ancient ubiquitous protein 1	AUP1	550	1.087	8	3	7	3	410	45.8	0.914540776
NP_006546.1	synaptobrevin homolog YKT6 isoform 1	YKT6	10652	0.843	14	4	11	4	198	22.4	0.481736658
NP_000839.1	glutathione S-transferase Mu 2 isoform 1	GSTM2	2946	1.554	12	2	9	1	218	25.7	0.029303668
XP_006720310.1	serine/threonine-protein kinase VRK1 isoform X2	VRK1	7443	1.048	11	5	6	5	420	48	0.991139167
NP_001257905.1	IST1 homolog isoform c	IST1	9798	0.877	10	4	8	4	379	41.5	0.689192915
NP_005861.2	histone deacetylase complex subunit SAP18	SAP18	10284	1.682	29	5	15	5	172	19.5	0.068294028
XP_024304778.1	serine/threonine-protein kinase TAO3 isoform X1	TAOK3	51347	0.755	6	5	14	5	907	106.3	0.08611538
XP_005246300.1	lanC-like protein 1 isoform X1	LANCL1	10314	0.726	11	3	6	3	411	46.5	0.495865067
NP_056456.1	testin isoform 1	TES	26136	0.875	12	3	8	3	421	48	0.576363682
NP_001020471.2	ras-related protein Rab-12	RAB12	201475	1.72	12	3	15	2	244	27.2	0.73936634
NP_002881.1	ras GTPase-activating protein 1 isoform 1	RASA1	5921	0.536	3	3	8	3	1047	116.3	0.369567551
NP_068815.2	optineurin	OPTN	10133	0.338	4	2	6	2	577	65.9	0.200237489
NP_061899.2	calcium load-activated calcium channel isoform a	TMCO1	54499	2.361	13	3	11	3	239	27.1	0.066015312
NP_003674.1	ribosomal RNA processing protein 1 homolog A	RRP1	8568	1.201	8	3	8	3	461	52.8	0.828667757
NP_057475.2	nucleolar protein 16 isoform 3	NOP16	51491	1.023	23	3	4	3	178	21.2	0.886116832
XP_006715809.1	anillin isoform X1	ANLN	54443	1.037	3	4	8	4	1142	126.4	0.916431287
NP_003084.1	U1 small nuclear ribonucleoprotein C	SNRPC	6631	1.503	13	3	10	3	159	17.4	0.02433832
NP_004326.1	bone marrow stromal antigen 2 precursor	BST2	684	1.83	14	2	6	2	180	19.8	0.16622317
NP_003083.1	U2 small nuclear ribonucleoprotein B''	SNRPB2	6629	1.722	18	5	14	3	225	25.5	0.338946484
NP_000453.2	ubiquitin-protein ligase E3A isoform 2	UBE3A	7337	0.693	5	3	8	3	875	100.6	0.061372023
NP_003468.2	pyruvate dehydrogenase protein X component, mitochondrial isoform 1 precursor	PDHX	8050	2.036	7	4	10	4	501	54.1	0.007239892
XP_016863314.1	spermatogenesis-associated protein 5 isoform X1	SPATA5	166378	0.6	5	3	20	2	917	100.4	0.7075041
XP_006714694.1	probable glutathione peroxidase 8 isoform X1	GPX8	493869	0.988	15	3	5	3	210	24	0.859117344
NP_001290386.1	TELO2-interacting protein 1 homolog	TTI1	9675	0.528	5	3	5	3	1089	122	0.475339659
NP_000468.1	serum albumin preproprotein	ALB	213	3.706	5	3	7	3	609	69.3	0.001837091
NP_004252.2	selenoprotein F isoform 1 precursor	SELENOF	9403	1.299	15	2	9	2	165	18.1	0.77615232
XP_016862508.1	protein SEC13 homolog isoform X1	SEC13	6396	0.795	9	2	4	2	368	40.7	0.805057057
XP_005248862.1	1-acyl-sn-glycerol-3-phosphate acyltransferase alpha isoform X1	AGPAT1	10554	1.096	18	3	8	3	287	32.2	0.952314141
NP_066362.2	interferon-induced transmembrane protein 3	IFITM3	10410	0.378	13	2	5	2	133	14.6	0.003159232
NP_079434.3	RUN and FYVE domain-containing protein 1 isoform a	RUFY1	80230	1.385	6	4	10	4	708	79.8	0.203766876
NP_060517.1	pre-mRNA-splicing factor RBM22	RBM22	55696	1.182	10	3	6	3	420	46.9	0.999789924
XP_011526806.1	death-inducer obliterator 1 isoform X1	DIDO1	11083	1.423	4	5	12	5	2276	247.6	0.03639675
NP_002792.1	proteasome subunit beta type-10 precursor	PSMB10	5699	0.278	14	3	8	3	273	28.9	0.003841926
NP_000628.2	glutathione reductase, mitochondrial isoform 1 precursor	GSR	2936	0.82	8	3	6	3	522	56.2	0.460886171
NP_001269588.1	ubiquitin carboxyl-terminal hydrolase 47 isoform a	USP47	55031	0.771	3	4	7	4	1355	154.6	0.711744355
NP_115990.3	methylmalonyl-CoA epimerase, mitochondrial precursor	MCEE	84693	2.289	15	2	4	2	176	18.7	0.054528017
NP_005424.1	tyrosine-protein kinase Yes	YES1	7525	1.175	7	4	14	1	543	60.8	0.153043427
NP_003091.2	sorting nexin-2 isoform 1	SNX2	6643	0.69	9	4	6	3	519	58.4	0.98202252
NP_001273542.1	[F-actin]-monooxygenase MICAL1 isoform 3	MICAL1	64780	0.938	6	3	13	3	1086	119.7	0.830076708
NP_054808.1	nucleoporin p58/p45 isoform a	NUP58	9818	1.238	8	3	9	3	599	60.9	0.006015858
XP_005250728.1	protein BUD31 homolog isoform X1	BUD31	8896	0.751	21	2	6	2	145	17.3	0.589003118
NP_000149.3	1,4-alpha-glucan-branching enzyme	GBE1	2632	0.476	6	3	12	3	702	80.4	0.029386679
NP_057569.2	vacuolar protein sorting-associated protein VTA1 homolog isoform a	VTA1	51534	0.67	9	2	5	2	307	33.9	0.628167203
NP_057283.1	U6 snRNA-associated Sm-like protein LSm7	LSM7	51690	1.037	40	3	9	3	103	11.6	0.844024181
NP_038461.2	transportin-2 isoform 2	TNPO2	30000	0.905	6	5	13	1	887	100.3	0.082430696
NP_115500.1	haloacid dehalogenase-like hydrolase domain-containing protein 2 isoform 1	HDHD2	84064	0.935	13	2	5	2	259	28.5	0.593458446
NP_073713.2	C-terminal-binding protein 2 isoform 2	CTBP2	1488	1.269	5	5	12	1	985	106.1	0.204849743
NP_003124.1	signal recognition particle 9 kDa protein isoform 2	SRP9	6726	1.13	22	2	12	2	86	10.1	0.247196838
NP_776155.1	FUN14 domain-containing protein 1	FUNDC1	139341	1.407	11	1	9	1	155	17.2	0.482498187
NP_077005.2	WD repeat-containing protein 18	WDR18	57418	1.074	6	3	9	3	432	47.4	0.535738624
NP_060294.1	E3 ubiquitin-protein ligase MARCH5	MARCH5	54708	0.906	18	3	6	3	278	31.2	0.906773549
XP_011535277.1	epimerase family protein SDR39U1 isoform X1	SDR39U1	56948	0.879	12	2	13	2	316	33.5	0.465280223
NP_001290472.1	prefoldin subunit 3 isoform 2	VBP1	7411	0.7	19	4	8	4	233	26.5	0.314157671
XP_005263233.1	methylsterol monooxygenase 1 isoform X1	MSMO1	6307	2.174	5	2	8	2	293	35.2	0.000106828
NP_061330.2	brain-specific angiogenesis inhibitor 1-associated protein 2-like protein 1	BAIAP2L1	55971	0.846	10	6	11	6	511	56.8	0.031635171
NP_001091981.1	kinesin-like protein KIF2A isoform 2	KIF2A	3796	0.557	5	4	7	4	744	84	0.393198751
NP_056277.2	nucleolar protein 11 isoform 1	NOL11	25926	1.354	5	3	10	3	719	81.1	0.516174389
NP_002023.2	ferritin heavy chain	FTH1	2495	1.048	22	3	5	3	183	21.2	0.866677095
NP_055419.1	tax1-binding protein 3 isoform 1	TAX1BP3	30851	0.441	14	1	10	1	124	13.7	0.013458515
NP_002789.1	proteasome subunit beta type-6 isoform 1 precursor	PSMB6	5694	0.929	13	3	17	3	239	25.3	0.727935818
NP_775882.2	protein FAM98B	FAM98B	283742	0.887	15	4	9	3	433	45.5	0.711484118
XP_016857522.1	protein S100-A6 isoform X1	S100A6	6277	0.466	43	5	32	5	90	10.2	0.00292636
NP_113619.2	conserved oligomeric Golgi complex subunit 3	COG3	83548	0.625	5	2	7	2	828	94	0.57809324
XP_011508153.1	E3 ubiquitin-protein ligase RING2 isoform X1	RNF2	6045	1.078	13	3	5	3	336	37.6	0.894470296
XP_011532532.1	oxidoreductase NAD-binding domain-containing protein 1 isoform X1	OXNAD1	92106	1.326	8	3	5	3	385	42.9	0.343185961
NP_003038.2	sodium/hydrogen exchanger 1	SLC9A1	6548	1.407	3	2	5	2	815	90.7	0.182855384
XP_005268523.1	RNA-binding protein 27 isoform X1	RBM27	54439	0.998	3	2	7	1	1061	118.7	0.755962661
NP_006519.1	tissue factor pathway inhibitor 2 isoform 1 precursor	TFPI2	7980	0.752	10	2	8	2	235	26.9	0.03934365
NP_057049.5	NADH dehydrogenase	NDUFA13	51079	1.196	26	3	8	3	144	16.7	0.656968312
NP_001019907.1	CD276 antigen isoform a precursor	CD276	80381	1.306	5	1	2	1	534	57.2	0.993868757
NP_001171904.1	protein kinase C and casein kinase substrate in neurons protein 3	PACSIN3	29763	0.611	9	3	5	3	424	48.5	0.254440359
NP_009144.1	PRA1 family protein 2	PRAF2	11230	1.36	16	2	14	2	178	19.2	0.076092491
NP_892021.1	DBIRD complex subunit ZNF326 isoform 1	ZNF326	284695	1.597	3	1	6	1	582	65.6	0.053949503
NP_789788.1	glutamyl-tRNA(Gln) amidotransferase subunit C, mitochondrial	GATC	283459	1.602	27	3	7	3	136	15.1	0.033400212
XP_005250699.1	protein-tyrosine sulfotransferase 1 isoform X1	TPST1	8460	1.612	12	3	8	3	376	42.8	0.178909356
NP_066018.1	nuclear receptor coactivator 5 isoform 1	NCOA5	57727	1.007	8	3	7	3	579	65.5	0.94341065
XP_016872205.1	calcium/calmodulin-dependent protein kinase type II subunit gamma isoform X1	CAMK2G	818	1.171	9	3	6	1	615	67.7	0.577456043
NP_803173.1	syntaxin-12	STX12	23673	1.617	9	2	12	2	276	31.6	4.53E-05
XP_011538066.1	tumor necrosis factor receptor superfamily member 6 isoform X1	FAS	355	1.25	13	4	7	4	389	43.6	0.45729129
NP_001034937.1	phospholipid hydroperoxide glutathione peroxidase isoform C	GPX4	2879	0.77	20	4	8	4	234	27.1	0.579235431
NP_619634.1	protein phosphatase 1 regulatory subunit 14B	PPP1R14B	26472	0.315	31	2	9	2	147	15.9	0.015075057
NP_057736.4	crooked neck-like protein 1 isoform a	CRNKL1	51340	1.08	6	5	9	5	848	100.4	0.792273484
NP_003679.2	peripheral plasma membrane protein CASK isoform 1	CASK	8573	1.234	6	3	8	3	921	104.5	0.621709709
NP_003126.1	signal recognition particle 19 kDa protein isoform 1	SRP19	6728	0.871	38	3	7	3	144	16.1	0.730503071
NP_945327.2	negative elongation factor C/D	NELFCD	51497	0.772	5	2	7	2	599	67.3	0.525921274
NP_004882.1	39S ribosomal protein L33, mitochondrial isoform a	MRPL33	9553	1.839	31	4	7	4	65	7.6	0.099652253
NP_008818.3	mRNA decay activator protein ZFP36L2	ZFP36L2	678	0.713	7	3	8	3	494	51	0.676726793
NP_114429.2	coiled-coil domain-containing protein 8	CCDC8	83987	1.12	9	2	7	2	538	59.3	0.568755435
NP_002480.1	cytochrome c oxidase subunit NDUFA4	NDUFA4	4697	0.625	35	3	11	3	81	9.4	0.026613381
NP_005810.1	syntaxin-6 isoform 1	STX6	10228	1.738	9	2	6	2	255	29.2	0.001337263
XP_011513026.1	28S ribosomal protein S10, mitochondrial isoform X1	MRPS10	55173	1.665	12	3	7	3	211	24	0.049691572
NP_002015.1	synaptic functional regulator FMR1 isoform ISO1	FMR1	2332	0.783	5	3	7	2	632	71.1	0.726343418
NP_002743.3	mitogen-activated protein kinase 9 isoform alpha2	MAPK9	5601	0.369	8	3	7	2	424	48.1	0.277165733
NP_064520.2	ATPase WRNIP1 isoform 1	WRNIP1	56897	1.176	7	4	8	4	665	72.1	0.668583427
NP_073572.2	Golgi resident protein GCP60	ACBD3	64746	1.181	9	3	8	3	528	60.6	0.000617234
NP_005720.1	peptidyl-prolyl cis-trans isomerase F, mitochondrial precursor	PPIF	10105	1.76	15	4	10	3	207	22	0.33407173
XP_016884289.1	arylsulfatase A isoform X1	ARSA	410	0.955	7	2	8	2	547	57.7	0.998991469
XP_024306443.1	clustered mitochondria protein homolog isoform X1	CLUH	23277	0.486	3	3	6	2	1583	174.5	0.003469582
NP_056285.1	rab11 family-interacting protein 5	RAB11FIP5	26056	0.709	7	2	5	2	653	70.4	0.663927441
NP_057104.2	dimethyladenosine transferase 1, mitochondrial isoform 1	TFB1M	51106	1.223	10	3	7	3	346	39.5	0.781613499
XP_011511214.1	DCN1-like protein 1 isoform X2	DCUN1D1	54165	0.633	14	3	8	3	280	32.8	0.406327471
NP_006169.2	vesicle-fusing ATPase	NSF	4905	1.424	6	4	7	4	744	82.5	0.122742317
NP_006058.1	ER membrane protein complex subunit 8 isoform 1	EMC8	10328	1.711	14	3	8	3	210	23.8	0.145156417
NP_004634.1	polyadenylate-binding protein 2 isoform 1	PABPN1	8106	1.594	18	2	6	2	306	32.7	0.000603161
NP_258411.2	tripartite motif-containing protein 47	TRIM47	91107	0.999	6	3	6	3	638	69.5	0.891555765
NP_000567.1	interleukin-1 beta proprotein	IL1B	3553	0.789	9	2	7	2	269	30.7	0.722593746
NP_003681.1	interferon-inducible double-stranded RNA-dependent protein kinase activator A isoform 1	PRKRA	8575	0.566	10	2	6	2	313	34.4	0.47951963
NP_005923.2	mitochondrial intermediate peptidase	MIPEP	4285	1.092	9	4	8	4	713	80.6	0.83479642
NP_001128251.1	importin-11 isoform 1	IPO11	51194	0.482	5	4	9	4	1015	116.9	0.563007312
NP_005427.2	coiled-coil domain-containing protein 6	CCDC6	8030	0.799	10	4	5	4	474	53.3	0.916873889
XP_016868173.1	armadillo repeat-containing protein 10 isoform X1	ARMC10	83787	1.92	15	4	6	4	352	38.6	0.128971282
NP_060898.2	U3 small nucleolar RNA-associated protein 6 homolog	UTP6	55813	1.233	5	4	8	4	597	70.1	0.652532318
NP_056197.3	E3 ubiquitin-protein ligase HECTD1	HECTD1	25831	0.402	2	3	7	3	2610	289.2	0.00117405
NP_001316043.1	BRISC and BRCA1-A complex member 2 isoform 5	BABAM2	9577	0.85	7	3	9	3	472	53.1	0.637956768
NP_005473.1	GPI-anchor transamidase precursor	PIGK	10026	1.415	8	3	9	3	395	45.2	0.285585974
XP_011519396.1	UDP-glucose:glycoprotein glucosyltransferase 2 isoform X1	UGGT2	55757	1.464	2	3	6	2	1590	182.8	0.955286501
NP_001351048.1	AP-3 complex subunit sigma-1 isoform 7	AP3S1	1176	0.466	14	3	7	3	209	23.5	0.027811698
XP_005248599.1	phosphatidylinositol 3-kinase regulatory subunit alpha isoform X1	PIK3R1	5295	0.446	6	2	14	2	724	83.5	0.000555994
NP_115700.1	cancer-related nucleoside-triphosphatase isoform 1	NTPCR	84284	0.976	32	4	6	4	190	20.7	0.956190586
NP_852136.1	replication factor C subunit 2 isoform 1	RFC2	5982	1.053	12	3	7	3	354	39.1	0.739015103
NP_001116078.1	lysosome-associated membrane glycoprotein 2 isoform C precursor	LAMP2	3920	0.615	5	2	5	2	411	45.1	0.845071216
XP_011525359.1	protein Smaug homolog 2 isoform X1	SAMD4B	55095	0.787	2	1	5	1	709	77	0.153974665
NP_006708.2	spindlin-1	SPIN1	10927	1.14	6	1	3	1	262	29.6	0.938015055
NP_001768.1	leukocyte surface antigen CD47 isoform 1 precursor	CD47	961	1.09	6	2	13	2	323	35.2	0.155836686
NP_002694.3	amidophosphoribosyltransferase precursor	PPAT	5471	0.745	8	4	12	4	517	57.4	0.411146377
NP_001290194.1	peptidyl-tRNA hydrolase ICT1, mitochondrial isoform 2 precursor	MRPL58	3396	1.224	24	4	9	4	229	25.6	0.731703834
NP_005990.1	translin-associated protein X	TSNAX	7257	0.667	16	3	10	3	290	33.1	0.00011233
NP_002422.1	CDK-activating kinase assembly factor MAT1 isoform 1	MNAT1	4331	1.145	12	3	7	3	309	35.8	0.877418716
XP_016855540.1	peptidyl-prolyl cis-trans isomerase E isoform X1	PPIE	10450	1.02	5	3	10	2	314	35	0.921305137
NP_057494.3	charged multivesicular body protein 5 isoform 1	CHMP5	51510	0.753	11	3	8	3	219	24.6	0.68060481
XP_005273739.1	set1/Ash2 histone methyltransferase complex subunit ASH2 isoform X1	ASH2L	9070	1.076	5	2	5	2	634	69.2	0.932911659
NP_001278910.1	receptor-type tyrosine-protein phosphatase kappa isoform c precursor	PTPRK	5796	1.295	4	4	7	4	1462	164.5	0.588387442
NP_001287758.1	cold-inducible RNA-binding protein isoform 3	CIRBP	1153	0.864	11	2	6	2	297	31.9	0.507458027
NP_056290.3	protein FAM98A isoform 1	FAM98A	25940	0.787	14	4	8	3	518	55.2	0.20190585
XP_024307582.1	proteasomal ubiquitin receptor ADRM1 isoform X1	ADRM1	11047	0.628	6	2	9	2	470	48.4	0.002969514
NP_079120.1	nuclear pore complex protein Nup85 isoform 1	NUP85	79902	1.398	6	4	6	4	656	75	0.703150901
XP_006713854.1	leucine-rich repeat and calponin homology domain-containing protein 3 isoform X3	LRCH3	84859	0.54	6	3	8	3	777	86	0.297529948
NP_005029.1	peptidyl-prolyl cis-trans isomerase D	PPID	5481	0.57	11	5	10	5	370	40.7	0.008727207
NP_001264890.1	stromal interaction molecule 1 isoform 1 precursor	STIM1	6786	1.458	3	2	6	2	791	88.6	0.653568017
NP_003752.2	vesicle-associated membrane protein 8	VAMP8	8673	1.737	24	2	6	2	100	11.4	0.354198163
NP_005063.1	solute carrier family 12 member 4 isoform a	SLC12A4	6560	1.052	4	4	9	4	1085	120.6	0.977072701
NP_612461.1	dehydrogenase/reductase SDR family member 1	DHRS1	115817	1.293	14	3	6	3	313	33.9	0.971020966
XP_005246708.1	peptidyl-prolyl cis-trans isomerase-like 3 isoform X1	PPIL3	53938	0.799	12	1	4	1	165	18.6	0.723710041
NP_077297.2	RNA-binding protein 42 isoform 1	RBM42	79171	0.996	13	3	6	3	480	50.4	0.931773515
NP_001295140.1	probable glutamate--tRNA ligase, mitochondrial isoform 2	EARS2	124454	1.071	8	3	5	3	534	60	0.898159385
NP_003578.2	pre-mRNA-splicing factor ATP-dependent RNA helicase DHX16 isoform 1	DHX16	8449	1.115	4	3	6	3	1041	119.2	0.919847904
XP_024309132.1	coiled-coil domain-containing protein 12 isoform X4	CCDC12	151903	1.23	25	3	8	3	179	20.5	0.684410777
NP_001035112.1	tyrosine-protein phosphatase non-receptor type substrate 1 isoform 1 precursor	SIRPA	140885	1.739	8	3	5	3	504	54.9	0.385067486
NP_612410.1	multivesicular body subunit 12A isoform 1	MVB12A	93343	0.777	11	2	5	2	273	28.8	0.684670449
NP_149351.1	surfeit locus protein 4 isoform 1	SURF4	6836	1.04	9	2	18	2	269	30.4	0.781035445
NP_001253975.1	FERM domain-containing protein 6 isoform 2	FRMD6	122786	1.387	6	3	5	3	622	72	0.130939687
NP_006411.2	brefeldin A-inhibited guanine nucleotide-exchange protein 2	ARFGEF2	10564	0.61	2	3	9	1	1785	201.9	0.033502691
NP_001091896.1	SWI/SNF-related matrix-associated actin-dependent regulator of chromatin subfamily D member 2 isoform 1	SMARCD2	6603	1.146	8	2	5	2	531	58.9	0.607975375
NP_005779.1	protein arginine N-methyltransferase 3 isoform 1	PRMT3	10196	0.512	7	3	6	3	531	59.9	0.003608352
XP_005246698.1	glia-derived nexin isoform X1	SERPINE2	5270	1.684	9	3	6	3	410	45.2	0.476182219
NP_002220.1	transcription factor jun-B	JUNB	3726	1.39	13	4	14	3	347	35.9	0.359839977
XP_011533233.1	poly	PARP4	143	0.67	2	4	11	4	1771	197.3	0.117732175
NP_001308427.1	septin-10 isoform 4	SEPT10	151011	0.833	6	3	12	3	544	62.9	0.019527476
XP_024308741.1	MOB kinase activator 1A isoform X1	MOB1A	55233	0.674	4	1	6	1	258	29.9	0.214448275
NP_001174.2	V-type proton ATPase subunit S1 precursor	ATP6AP1	537	1.821	6	2	6	2	470	52	0.104717047
NP_001167459.1	rab-like protein 6 isoform 3	RABL6	55684	0.875	8	4	11	4	730	79.6	0.66851882
XP_011508441.1	rho guanine nucleotide exchange factor 2 isoform X1	ARHGEF2	9181	0.69	5	3	9	3	1139	127.7	0.75349417
NP_001316929.1	membrane magnesium transporter 1 precursor	MMGT1	93380	1.432	18	1	5	1	131	14.7	0.698887484
NP_001035754.1	PPAN-P2RY11 protein isoform 1	PPAN-P2RY11	692312	1.116	6	2	5	2	794	87.9	0.894297873
NP_001340828.1	katanin p60 ATPase-containing subunit A-like 2 isoform 1	KATNAL2	83473	0.493	7	2	12	1	564	64.3	0.478257374
NP_001015880.1	bifunctional 3'-phosphoadenosine 5'-phosphosulfate synthase 2 isoform b	PAPSS2	9060	0.935	4	2	6	1	619	69.9	0.567827417
NP_001121631.1	vacuolar protein sorting-associated protein 53 homolog isoform 1	VPS53	55275	0.611	8	3	6	3	832	94.3	0.398768058

Patil et al,2020. Proteomic and phosphoproteomic profiling of shammah-induced signaling in oral keratinocytes

Supplementary Table S1: List of proteins identified and quantified in OKF6/TERT1-Shammah and OKF6/TERT1-Parental total proteome using TMT-based quantitative proteomic approach

Accession	Protein Description	Gene Symbol	Gene ID	Fold Change	Coverage [%]	# Peptides	# PSMs	# Unique Peptides	# AAs	MW [kDa]	p-value
NP_004704.2	nuclear export mediator factor NEMF isoform 1	NEMF	9147	0.884	4	4	8	4	1076	122.9	0.874574784
XP_024304529.1	AH receptor-interacting protein isoform X1	AIP	9049	0.856	4	1	6	1	330	37.6	0.259596188
NP_115721.1	partner of Y14 and mago isoform 1	PYM1	84305	0.818	29	3	7	3	204	22.6	0.533201811
NP_002446.3	metaxin-1 isoform 1	MTX1	4580	1.384	12	4	10	4	466	51.4	0.597913952
NP_064539.1	ER membrane protein complex subunit 7 precursor	EMC7	56851	1.331	12	2	6	2	242	26.5	0.007127669
XP_011529315.1	SH3 domain-binding glutamic acid-rich-like protein isoform X1	SH3BGRL	6451	0.42	18	2	4	2	176	20.2	0.072740426
NP_078849.1	protein-glutamate O-methyltransferase isoform a	ARMT1	79624	0.249	13	4	8	4	441	51.1	0.000220534
NP_115812.1	chromosome alignment-maintaining phosphoprotein 1	CHAMP1	283489	1.208	5	3	8	3	812	89	0.255985282
XP_005246274.1	abl interactor 2 isoform X1	ABI2	10152	1.033	7	3	5	2	542	58.7	0.960106886
NP_057111.1	endoribonuclease LACTB2	LACTB2	51110	1.072	13	3	7	3	288	32.8	0.749583868
XP_011543890.1	patatin-like phospholipase domain-containing protein 4 isoform X1	PNPLA4	8228	1.058	13	2	4	2	253	28	0.974161983
NP_056271.3	negative elongation factor B	NELFB	25920	1.353	7	4	6	4	628	70	0.667707202
NP_001240804.1	UDP-glucuronic acid decarboxylase 1 isoform 1	UXS1	80146	1.347	10	2	5	2	425	48.1	0.451633289
XP_024306413.1	neuron navigator 1 isoform X8	NAV1	89796	0.757	4	4	6	4	1900	205.3	0.616320563
NP_059133.1	cleavage and polyadenylation specificity factor subunit 2 isoform 1	CPSF2	53981	1.128	7	4	7	4	782	88.4	0.628646021
NP_005857.1	sigma non-opioid intracellular receptor 1 isoform 1	SIGMAR1	10280	0.509	20	2	6	2	223	25.1	0.225458634
XP_024308136.1	cytochrome c-type heme lyase isoform X1	HCCS	3052	0.985	10	3	7	3	288	33.5	0.991875575
NP_002204.2	integrin beta-5 isoform a precursor	ITGB5	3693	1.31	7	4	8	4	799	88	0.671827055
XP_016868509.1	cytochrome c oxidase subunit 6C isoform X1	COX6C	1345	0.936	23	3	13	3	75	8.8	0.164925445
NP_001336377.1	kynurenine--oxoglutarate transaminase 3 isoform 1	KYAT3	56267	1.163	12	3	5	3	454	51.4	0.775716035
NP_116021.2	poly(A) polymerase alpha isoform 1	PAPOLA	10914	1.224	7	4	6	4	745	82.8	0.76219324
NP_001273015.1	uncharacterized protein C11orf98	C11orf98	102288414	1.256	17	2	9	2	123	14.2	0.013219492
XP_024303241.1	45 kDa calcium-binding protein isoform X1	SDF4	51150	2.267	11	3	5	3	407	46.3	0.001923649
NP_115686.3	caspase recruitment domain-containing protein 19 isoform 1	CARD19	84270	1.301	19	3	5	3	183	20.6	0.964101124
XP_005255597.1	SUMO-conjugating enzyme UBC9 isoform X1	UBE2I	7329	0.595	13	3	6	3	184	20.4	0.168439245
NP_001129115.1	dynactin subunit 4 isoform a	DCTN4	51164	0.897	10	2	6	2	467	53.1	0.004395231
XP_005257071.1	RNA-binding protein Musashi homolog 2 isoform X1	MSI2	124540	1.205	12	3	6	3	346	36.9	0.886671046
NP_115758.3	conserved oligomeric Golgi complex subunit 8	COG8	84342	0.982	10	4	10	4	612	68.4	0.913365528
NP_002487.1	NADH dehydrogenase	NDUFS8	4728	0.894	13	2	5	2	210	23.7	0.843565991
XP_011525496.1	splicing factor, arginine/serine-rich 19 isoform X1	SCAF1	58506	1.224	2	2	4	2	1315	139.5	0.158238786
NP_001290162.1	interferon-stimulated gene 20 kDa protein isoform a	ISG20	3669	0.436	15	3	9	3	181	20.4	0.224006886
NP_789764.1	39S ribosomal protein L43, mitochondrial isoform d	MRPL43	84545	1.372	9	2	6	2	235	25.8	0.054851337
XP_016858234.1	translation initiation factor eIF-2B subunit gamma isoform X1	EIF2B3	8891	0.545	9	3	7	3	457	51.1	0.018662029
NP_000987.2	60S ribosomal protein L35a	RPL35A	6165	1.23	16	4	16	4	110	12.5	0.393147076
NP_003944.1	myelin protein zero-like protein 1 isoform a precursor	MPZL1	9019	1.361	12	3	8	3	269	29.1	0.136888696
NP_001269951.1	GMP reductase 2 isoform 4	GMPR2	51292	0.822	8	3	6	3	427	46.9	0.056624814
XP_006718549.1	EH domain-binding protein 1-like protein 1 isoform X1	EHBP1L1	254102	0.681	2	2	5	2	1535	163	0.456431867
XP_005248869.1	phenylalanine--tRNA ligase, mitochondrial isoform X1	FARS2	10667	1.451	6	3	10	3	451	52.3	0.025796126
NP_001306086.1	dynein light chain roadblock-type 1 isoform d	DYNLRB1	83658	0.697	23	2	6	2	121	13.4	0.131798257
NP_037397.2	vacuolar protein sorting-associated protein 51 homolog	VPS51	738	0.601	4	2	5	2	782	86	0.045990735
NP_001099040.1	nucleoside diphosphate-linked moiety X motif 19 precursor	NUDT19	390916	1.301	9	3	7	3	375	42.2	0.98677256
NP_001295561.1	DNA polymerase delta catalytic subunit isoform 2	POLD1	5424	0.771	7	6	12	5	1133	126.3	0.439969958
NP_057395.1	ATPase inhibitor, mitochondrial isoform 1 precursor	ATP5IF1	93974	2.895	15	3	15	3	106	12.2	0.001577232
XP_016871599.1	ankyrin-3 isoform X1	ANK3	288	1.149	1	4	7	4	4504	494.1	0.846323178
NP_060855.2	large subunit GTPase 1 homolog	LSG1	55341	1.26	5	1	2	1	658	75.2	0.774754517
NP_001317317.1	EKC/KEOPS complex subunit TPRKB isoform b	TPRKB	51002	0.343	31	4	7	4	175	19.6	0.421638894
NP_055182.3	protein FAM162A	FAM162A	26355	1.962	17	3	13	3	154	17.3	0.076342127
NP_057053.1	28S ribosomal protein S17, mitochondrial	MRPS17	51373	1.448	29	2	4	2	130	14.5	0.284370294
NP_057121.2	probable U3 small nucleolar RNA-associated protein 11	UTP11	51118	1.459	15	5	10	5	253	30.4	0.554799934
NP_085056.2	cytochrome b5 type B	CYB5B	80777	1.35	31	2	7	2	150	16.7	0.168650908
NP_954592.1	histone-arginine methyltransferase CARM1	CARM1	10498	1.044	7	3	5	3	608	65.8	0.898933773
XP_011508501.1	zinc finger CCCH domain-containing protein 11A isoform X1	ZC3H11A	9877	1.2	5	3	8	3	810	89.1	0.70317344
NP_031383.1	conserved oligomeric Golgi complex subunit 2 isoform 1	COG2	22796	0.953	7	4	5	4	738	83.2	0.94978919
XP_011542487.1	lamin-B receptor isoform X1	LBR	3930	1.143	7	4	10	4	615	70.7	0.021058513
NP_001182484.1	clathrin interactor 1 isoform 1	CLINT1	9685	0.938	7	4	7	4	643	70.3	0.964488071
NP_056449.1	KIF1-binding protein	KIF1BP	26128	0.65	7	3	8	3	621	71.8	0.045305777
XP_011507962.1	protein odr-4 homolog isoform X1	ODR4	54953	1.545	10	3	5	3	454	51.1	0.099158778
XP_024303159.1	long-chain fatty acid transport protein 4 isoform X1	SLC27A4	10999	1.289	7	4	11	4	643	72	0.037498296
NP_068598.1	FAST kinase domain-containing protein 5, mitochondrial	FASTKD5	60493	1.346	4	3	8	3	764	86.5	0.337267995
XP_005252377.1	tripartite motif-containing protein 14 isoform X1	TRIM14	9830	0.654	6	2	6	2	442	49.7	0.027091919
XP_005274252.1	syntaxin-3 isoform X3	STX3	6809	1.449	5	2	6	2	289	33.1	0.00084564
NP_079170.2	nucleolar protein 10 isoform 1	NOL10	79954	1.602	4	2	5	2	688	80.3	0.314241871
NP_061138.3	vacuolar protein sorting-associated protein 33B isoform 1	VPS33B	26276	0.721	4	3	6	3	617	70.5	0.619547354
XP_024308560.1	endoplasmic reticulum lectin 1 isoform X1	ERLEC1	27248	2.258	7	4	10	4	524	59.3	0.22092806
NP_004230.2	thyroid receptor-interacting protein 11 isoform 1	TRIP11	9321	0.931	3	3	6	3	1979	227.4	0.894905953
NP_006775.1	WD repeat-containing protein 3	WDR3	10885	1.11	6	4	8	4	943	106	0.678877843
NP_065813.1	SLIT-ROBO Rho GTPase-activating protein 1 isoform 1	SRGAP1	57522	0.674	3	3	6	3	1085	124.2	0.476381042
XP_024305736.1	COMM domain-containing protein 4 isoform X1	COMMD4	54939	0.676	17	3	6	3	252	27.2	0.647050636
NP_001010867.1	putative transferase CAF17, mitochondrial isoform 1 precursor	IBA57	200205	0.57	13	2	4	2	356	38.1	0.634882879
NP_001098057.1	schlafen family member 11	SLFN11	91607	0.749	4	3	5	2	901	102.8	0.389940034
NP_612393.2	mitochondrial ribosome-associated GTPase 1	MTG1	92170	1.184	9	2	2	2	334	37.2	0.846522234
NP_003860.3	cocaine esterase isoform 1 precursor	CES2	8824	1.115	10	3	7	3	559	61.8	0.251450593
NP_115786.1	protein Hook homolog 3	HOOK3	84376	0.982	5	3	7	3	718	83.1	0.880992518
XP_005262942.1	ELMO domain-containing protein 2 isoform X1	ELMOD2	255520	1.767	10	3	10	3	305	36.4	0.005887032
XP_016862214.1	protein polybromo-1 isoform X1	PBRM1	55193	1.198	3	5	11	5	1734	198	0.31309492
NP_001136270.1	chromatin complexes subunit BAP18 isoform 1	C17orf49	124944	1.027	7	1	6	1	192	19.9	0.411158124
NP_001288784.1	SAP30-binding protein isoform 1	SAP30BP	29115	1.142	13	3	8	3	342	37.6	0.804213176
NP_671713.1	quinone oxidoreductase PIG3 isoform 1	TP53I3	9540	0.588	15	4	9	4	332	35.5	0.01930428
NP_001180414.1	LIM and senescent cell antigen-like-containing domain protein 1 isoform a	LIMS1	3987	0.959	9	3	5	3	387	44.4	0.933437146
NP_612455.1	mitochondrial assembly of ribosomal large subunit protein 1	MALSU1	115416	0.817	24	2	6	2	234	26.2	0.582032278
XP_024301814.1	mothers against decapentaplegic homolog 5 isoform X1	SMAD5	4090	0.943	7	3	7	3	465	52.2	0.992355927
NP_002124.1	heme oxygenase 1	HMOX1	3162	1.643	8	1	2	1	288	32.8	0.985725698
NP_057124.3	transmembrane emp24 domain-containing protein 5 isoform 1 precursor	TMED5	50999	1.127	10	2	9	2	229	26	0.39034056
XP_016873329.1	platelet-activating factor acetylhydrolase IB subunit beta isoform X1	PAFAH1B2	5049	0.599	17	3	10	3	242	27.1	0.194985217
NP_001335.1	dolichyl-diphosphooligosaccharide--protein glycosyltransferase subunit DAD1	DAD1	1603	0.929	11	1	6	1	113	12.5	0.500279124
XP_011509935.1	anaphase-promoting complex subunit 1 isoform X1	ANAPC1	64682	0.721	2	2	6	2	1948	216.8	0.001135613
NP_002659.1	proteolipid protein 2	PLP2	5355	1.044	10	1	5	1	152	16.7	0.988753752
NP_005774.2	thioredoxin domain-containing protein 9	TXNDC9	10190	0.68	17	4	8	4	226	26.5	0.025292419
NP_036233.2	tripartite motif-containing protein 29 isoform 1	TRIM29	23650	1.268	6	4	10	4	588	65.8	0.607598329
NP_004136.2	unconventional myosin-IXb isoform 1	MYO9B	4650	0.396	2	4	9	4	2157	243.2	0.111369186
XP_011541142.1	serine-protein kinase ATM isoform X1	ATM	472	0.836	1	3	6	3	3056	350.5	0.77958557
NP_002613.2	prefoldin subunit 1	PFDN1	5201	0.706	24	3	8	3	122	14.2	0.330789395
NP_001489.1	glutamate--cysteine ligase catalytic subunit isoform a	GCLC	2729	0.613	4	3	10	3	637	72.7	0.189713741
NP_073616.6	probable ATP-dependent RNA helicase DDX31 isoform 1	DDX31	64794	1.191	3	3	8	3	851	94	0.563282374
NP_612444.2	protein FAM83F	FAM83F	113828	1.23	5	1	3	1	500	55.5	0.276372685
NP_001790.1	cyclin-dependent kinase 7 isoform 1	CDK7	1022	1.052	11	3	6	3	346	39	0.963470565
NP_001351.2	7-dehydrocholesterol reductase	DHCR7	1717	2.026	11	4	13	4	475	54.5	0.003264597
NP_001305965.1	cytochrome c oxidase subunit 7A-related protein, mitochondrial isoform a	COX7A2L	9167	1.133	44	3	5	3	114	12.6	0.945693688
NP_055506.1	RNA helicase aquarius	AQR	9716	1.368	2	3	6	3	1485	171.2	0.691964154
NP_056183.2	pannexin-1	PANX1	24145	0.831	13	3	8	3	426	48	0.302928189
NP_006006.3	AT-rich interactive domain-containing protein 1A isoform a	ARID1A	8289	1.027	2	3	4	3	2285	241.9	0.741871765
NP_757345.2	tapasin isoform 2 precursor	TAPBP	6892	1.187	6	3	11	3	504	53.9	0.00174175
NP_071898.2	E3 ubiquitin-protein ligase RNF25	RNF25	64320	0.564	7	2	6	2	459	51.2	0.002495492
NP_777576.1	E3 ubiquitin-protein ligase UBR1	UBR1	197131	0.803	2	2	5	2	1749	200.1	0.46452267
NP_071904.4	exportin-4	XPO4	64328	0.622	3	3	5	3	1151	130.1	0.595270025
NP_001258851.1	signal peptidase complex catalytic subunit SEC11A isoform 3	SEC11A	23478	0.902	18	4	10	4	185	21.4	0.592629427
XP_016858338.1	ras GTPase-activating protein nGAP isoform X1	RASAL2	9462	0.834	2	3	6	3	1419	160	0.708084208
NP_005317.2	hydroxyacylglutathione hydrolase, mitochondrial isoform 1 precursor	HAGH	3029	1.12	9	3	8	3	308	33.8	0.599289416
NP_957516.1	melanoma-associated antigen D2	MAGED2	10916	0.756	11	4	8	4	606	64.9	0.068298839
XP_024308225.1	RNA-binding protein 10 isoform X1	RBM10	8241	1.372	4	4	9	3	1029	113.9	0.510198187
XP_006716996.1	structural maintenance of chromosomes protein 2 isoform X1	SMC2	10592	0.634	5	4	8	4	1197	135.6	0.089495018
NP_116139.1	protein ABHD14B isoform 1	ABHD14B	84836	0.416	11	2	6	2	210	22.3	0.091610793
XP_016873413.1	phosphatidylinositol 4-phosphate 3-kinase C2 domain-containing subunit alpha isoform X1	PIK3C2A	5286	1.046	3	4	9	4	1686	190.6	0.398914118
NP_066284.2	dehydrogenase/reductase SDR family member 4 isoform 1	DHRS4	10901	1.642	18	4	9	4	278	29.5	0.067597987
NP_463460.1	peptidyl-prolyl cis-trans isomerase FKBP1A isoform a	FKBP1A	2280	0.703	13	1	8	1	108	11.9	0.583984355
XP_006717936.1	activating signal cointegrator 1 complex subunit 1 isoform X6	ASCC1	51008	0.585	8	4	7	4	357	41.2	0.339580655
NP_001306125.1	protein S100-A8 isoform a	S100A8	6279	0.993	19	2	6	2	117	13.5	0.708128374
NP_001273117.1	protein SCAF8 isoform a	SCAF8	22828	1.021	4	5	9	2	1349	148.8	0.593711206
NP_002956.1	protein S100-A9	S100A9	6280	1.205	26	3	5	3	114	13.2	0.889473474
XP_016855574.1	thioredoxin-interacting protein isoform X1	TXNIP	10628	0.702	7	3	7	3	395	44.2	0.108114847
NP_003012.1	small glutamine-rich tetratricopeptide repeat-containing protein alpha	SGTA	6449	0.68	16	4	10	4	313	34	0.018553544
NP_055487.2	mitochondrial ribonuclease P catalytic subunit isoform 1 precursor	KIAA0391	9692	0.598	5	3	5	3	583	67.3	0.677494234
NP_004323.2	valacyclovir hydrolase isoform 1 precursor	BPHL	670	1.524	13	5	12	5	291	32.5	0.103703698
NP_076426.1	39S ribosomal protein L34, mitochondrial	MRPL34	64981	1.471	25	2	5	2	92	10.2	0.4164479
NP_057033.2	Golgi to ER traffic protein 4 homolog	GET4	51608	0.806	8	2	4	2	327	36.5	0.863991264
NP_002660.1	pleiotropic regulator 1 isoform 1	PLRG1	5356	1.214	10	3	5	3	514	57.2	0.079243571
NP_115865.1	28S ribosomal protein S6, mitochondrial	MRPS6	64968	1.023	23	3	5	3	125	14.2	0.931072653
NP_001350777.1	thymidine kinase, cytosolic isoform 3	TK1	7083	0.291	7	1	6	1	267	28.6	0.002637162
XP_005256095.1	protein VAC14 homolog isoform X1	VAC14	55697	0.534	3	2	6	2	790	88.6	0.082019113
NP_055467.3	importin-13	IPO13	9670	0.881	5	3	5	3	963	108.1	0.885556657
NP_015561.1	ran-binding protein 3 isoform RANBP3-d	RANBP3	8498	0.916	5	2	6	2	567	60.2	0.899906294
NP_536355.1	selenoprotein M precursor	SELENOM	140606	1.479	19	2	3	2	145	16.2	0.673401
XP_005253966.1	5'-AMP-activated protein kinase subunit beta 1 isoform X1	PRKAB1	5564	0.648	21	3	4	3	270	30.4	0.333775143
NP_056235.5	DDB1- and CUL4-associated factor 13	DCAF13	25879	1.103	9	3	6	3	445	51.4	0.889298851
NP_005773.3	THO complex subunit 4	ALYREF	10189	1.426	4	1	9	1	264	27.5	0.160589982
NP_000172.2	beta-glucuronidase isoform 1 precursor	GUSB	2990	1.372	5	3	8	3	651	74.7	0.066416209
NP_001107563.1	cell division cycle protein 27 homolog isoform 1	CDC27	996	0.92	6	3	4	3	830	92.6	0.89707596
NP_006288.2	DNA repair protein XRCC1	XRCC1	7515	1.089	6	4	9	4	633	69.5	0.642474319
NP_001275584.1	dephospho-CoA kinase domain-containing protein	DCAKD	79877	1.171	16	3	6	3	231	26.5	0.875602731
XP_011517524.1	guanine deaminase isoform X1	GDA	9615	0.72	6	3	6	3	509	57.1	0.225949338
NP_003329.1	ubiquitin-conjugating enzyme E2 D1 isoform 1	UBE2D1	7321	0.65	26	2	6	2	147	16.6	7.11E-05
XP_011540628.1	dedicator of cytokinesis protein 7 isoform X1	DOCK7	85440	1.166	1	3	7	3	2140	242.4	0.926138119
NP_775487.2	keratin, type II cytoskeletal 78 isoform 1	KRT78	196374	1.129	4	4	25	1	520	56.8	0.558076794
NP_036614.1	zinc finger protein 281 isoform 1	ZNF281	23528	0.788	6	3	7	3	895	96.9	0.540277524
XP_006717354.1	bromodomain-containing protein 3 isoform X1	BRD3	8019	1.75	4	3	8	1	726	79.5	0.908495915
NP_938408.1	endoplasmic reticulum-Golgi intermediate compartment protein 3 isoform a	ERGIC3	51614	1.543	4	2	8	2	388	43.7	0.003677949
NP_005841.1	splicing factor 3B subunit 4	SF3B4	10262	2.058	3	1	3	1	424	44.4	0.698461174
NP_037425.1	transformer-2 protein homolog alpha isoform 1	TRA2A	29896	1.357	7	2	6	2	282	32.7	0.002138616
NP_002056.2	glutamine synthetase	GLUL	2752	0.557	11	4	6	4	373	42	0.601143304
NP_061882.2	toll-interacting protein isoform 1	TOLLIP	54472	0.912	8	2	4	2	274	30.3	0.91689495
NP_001029366.1	exosome complex component RRP45 isoform 1	EXOSC9	5393	1.029	6	2	7	2	456	50.8	0.931284888
NP_115605.2	SLIT and NTRK-like protein 6 precursor	SLITRK6	84189	3.326	3	2	5	2	841	95	0.0853407
NP_055410.1	5'(3')-deoxyribonucleotidase, cytosolic type isoform 1	NT5C	30833	0.533	12	1	5	1	201	23.4	0.205360548
XP_006718779.1	liprin-alpha-1 isoform X1	PPFIA1	8500	0.911	2	3	6	3	1306	147	0.748472581
NP_612208.1	glucosamine-6-phosphate isomerase 2 isoform 1	GNPDA2	132789	0.47	12	2	7	1	276	31.1	0.230966852
NP_003895.1	zinc finger protein ZPR1 isoform 1	ZPR1	8882	0.635	7	3	7	3	459	50.9	0.02319799
NP_005763.3	RNA 3'-terminal phosphate cyclase-like protein isoform a	RCL1	10171	0.734	15	5	11	5	373	40.8	0.102281803
NP_004283.2	ras and Rab interactor 1 isoform 1	RIN1	9610	0.9	3	2	9	2	783	84	0.180945487
XP_016884760.1	MICOS complex subunit MIC27 isoform X1	APOOL	139322	1.433	13	2	8	2	276	29.9	0.034089182
NP_002804.2	26S proteasome non-ATPase regulatory subunit 9 isoform 1	PSMD9	5715	0.548	9	2	4	2	223	24.7	0.520047421
XP_024308581.1	isoamyl acetate-hydrolyzing esterase 1 homolog isoform X1	IAH1	285148	0.56	19	3	5	3	272	30.6	0.474432753
XP_011515603.1	gasdermin-D isoform X1	GSDMD	79792	0.749	7	3	6	3	695	74.8	0.151410942
XP_005272541.1	angiopoietin-related protein 4 isoform X1	ANGPTL4	51129	3.104	8	3	10	3	424	47	0.000294921
XP_016883294.1	mitochondrial ribosome-associated GTPase 2 isoform X1	MTG2	26164	1.527	7	2	5	2	434	46.8	0.78938088
NP_006396.2	transmembrane 9 superfamily member 1 isoform a precursor	TM9SF1	10548	1.025	6	2	3	2	606	68.8	0.933432672
NP_001349967.1	integrator complex subunit 9 isoform 4	INTS9	55756	1.23	7	3	4	3	676	75.7	0.92592773
NP_001122082.1	ubiquitin carboxyl-terminal hydrolase 8 isoform a	USP8	9101	0.386	3	2	9	2	1118	127.4	1.46E-05
NP_055983.1	zinc finger CCCH domain-containing protein 4	ZC3H4	23211	0.787	4	3	8	3	1303	140.2	0.807629477
NP_001002010.2	cytosolic 5'-nucleotidase 3A isoform 1	NT5C3A	51251	0.665	9	3	4	3	331	37.4	0.746247704
NP_671717.1	F-box only protein 22 isoform a	FBXO22	26263	0.629	7	3	7	3	403	44.5	0.441850085
XP_006712115.1	E3 ubiquitin-protein ligase KCMF1 isoform X2	KCMF1	56888	0.554	4	1	5	1	389	42.8	0.226419001
XP_005277780.1	alpha-1,2-mannosyltransferase ALG9 isoform X1	ALG9	79796	1.287	8	3	6	3	629	72.2	0.842682892
NP_689478.1	transcription factor BTF3 homolog 4 isoform 1	BTF3L4	91408	0.977	42	3	7	3	158	17.3	0.792008045
NP_004851.2	fragile X mental retardation syndrome-related protein 2	FXR2	9513	1.121	5	3	6	1	673	74.2	0.90701343
XP_016873212.1	fatty acid desaturase 3 isoform X1	FADS3	3995	1.525	8	2	3	2	500	56.8	0.852000619
XP_024304073.1	protein enabled homolog isoform X1	ENAH	55740	0.529	4	3	7	3	1104	119.1	0.185020038
NP_004389.2	probable ATP-dependent RNA helicase DDX10	DDX10	1662	1.564	5	3	7	3	875	100.8	0.852599878
NP_000475.1	amyloid-beta A4 protein isoform a precursor	APP	351	2.356	3	1	3	1	770	86.9	0.000164094
XP_005247295.1	1-phosphatidylinositol 4,5-bisphosphate phosphodiesterase eta-1 isoform X1	PLCH1	23007	1.578	1	2	19	1	1705	190.8	0.086883041
XP_024306499.1	ARF GTPase-activating protein GIT1 isoform X1	GIT1	28964	0.628	8	4	9	3	809	89	0.006184836
NP_004587.1	U1 small nuclear ribonucleoprotein A	SNRPA	6626	0.586	16	3	10	1	282	31.3	0.142192561
XP_016866507.1	exocyst complex component 2 isoform X1	EXOC2	55770	0.67	5	4	6	4	924	104	0.108714803
NP_001104792.1	ATP-dependent RNA helicase DDX54 isoform 1	DDX54	79039	0.93	3	2	6	2	882	98.6	0.915711717
XP_011542222.1	cyclin-dependent kinase 16 isoform X1	CDK16	5127	0.866	7	3	11	2	571	63.3	0.899143813
NP_003898.2	translation initiation factor eIF-2B subunit epsilon	EIF2B5	8893	0.518	4	4	11	4	721	80.3	0.014295604
XP_006723234.1	striatin-4 isoform X1	STRN4	29888	0.61	3	2	4	2	775	83	0.567678395
NP_683725.1	cytochrome b5 isoform 1	CYB5A	1528	1.541	32	3	6	3	134	15.3	0.494913304
XP_024309648.1	la-related protein 7 isoform X1	LARP7	51574	1.378	7	3	6	3	595	68.4	0.419000278
NP_112570.2	28S ribosomal protein S15, mitochondrial	MRPS15	64960	1.72	13	4	8	4	257	29.8	0.087557271
NP_003007.2	serine/arginine-rich splicing factor 2	SRSF2	6427	1.171	11	2	11	2	221	25.5	0.304262656
NP_112483.1	ribosomal biogenesis protein LAS1L isoform 1	LAS1L	81887	0.793	5	3	7	3	734	83	0.0263225
NP_835227.1	reticulophagy regulator 3	RETREG3	162427	0.964	3	1	6	1	466	51.4	0.849261976
NP_001299602.1	choline-phosphate cytidylyltransferase A	PCYT1A	5130	1.045	9	2	6	2	367	41.7	0.805685553
NP_803882.1	cytochrome P450 20A1	CYP20A1	57404	1.159	6	3	6	3	462	52.4	0.706819882
NP_001096617.1	GRB10-interacting GYF protein 2 isoform a	GIGYF2	26058	0.871	3	4	7	4	1320	152.3	0.833847515
NP_001243546.1	gamma-tubulin complex component 2 isoform 1	TUBGCP2	10844	0.905	5	4	6	4	930	105.6	0.710220866
NP_004543.1	NADH dehydrogenase	NDUFS5	4725	1.543	11	1	6	1	106	12.5	0.027724731
NP_219482.1	zinc finger protein 622	ZNF622	90441	0.736	5	2	7	2	477	54.2	0.26451145
NP_064528.1	RNA-binding protein PNO1 isoform 1	PNO1	56902	0.988	12	3	11	3	252	27.9	0.922116644
NP_002895.3	negative elongation factor E	NELFE	7936	1.057	10	2	9	2	380	43.2	0.443697161
NP_060961.3	COBW domain-containing protein 1 isoform 1	CBWD1	55871	0.797	11	3	5	3	395	44	0.761131346
NP_055871.1	ribosomal RNA processing protein 1 homolog B	RRP1B	23076	1.108	6	4	7	4	758	84.4	0.467292858
NP_066930.1	28S ribosomal protein S12, mitochondrial precursor	MRPS12	6183	1.688	33	3	8	3	138	15.2	0.002457856
NP_008841.2	E3 ubiquitin-protein ligase RBBP6 isoform 1	RBBP6	5930	1.446	2	3	8	3	1792	201.4	0.134371917
XP_011527350.1	dnaJ homolog subfamily C member 5 isoform X1	DNAJC5	80331	1.667	16	2	6	2	198	22.1	0.000511998
NP_002711.1	serine/threonine-protein phosphatase 4 catalytic subunit isoform 1	PPP4C	5531	1.315	13	3	6	3	307	35.1	0.814788137
NP_060828.2	ATP-binding cassette sub-family F member 3 isoform 1	ABCF3	55324	0.582	2	1	6	1	709	79.7	0.000451799
NP_071935.1	DNA-directed RNA polymerase I subunit RPA49 isoform 1	POLR1E	64425	1.307	6	3	4	3	419	47.2	0.468909522
XP_011537045.1	vacuolar protein sorting-associated protein 37B isoform X1	VPS37B	79720	0.372	10	2	5	2	344	37.7	0.145471401
NP_690866.1	TIP41-like protein isoform 1	TIPRL	261726	0.526	18	4	12	4	272	31.4	0.002629218
XP_011541982.1	U3 small nucleolar RNA-associated protein 15 homolog isoform X1	UTP15	84135	1.13	6	3	9	3	518	58.4	0.040487946
XP_016884978.1	immunoglobulin-binding protein 1 isoform X1	IGBP1	3476	0.714	7	1	1	1	339	39.2	0.688419738
XP_005264597.1	serine/threonine-protein kinase VRK2 isoform X1	VRK2	7444	0.962	7	3	5	3	508	58.1	0.973419505
NP_001005743.1	protein numb homolog isoform 1	NUMB	8650	1.092	6	4	9	4	651	70.8	0.946267853
NP_060283.3	inositol monophosphatase 3	IMPAD1	54928	1.941	14	3	6	3	359	38.7	0.00946841
XP_011513560.1	oxysterol-binding protein-related protein 3 isoform X1	OSBPL3	26031	1.25	6	4	9	4	887	101.2	0.117538056
XP_006723890.1	extracellular sulfatase Sulf-2 isoform X1	SULF2	55959	1.935	4	3	7	3	871	100.5	0.031339618
NP_003668.2	density-regulated protein	DENR	8562	0.608	23	4	8	4	198	22.1	0.110193346
NP_001350700.1	ADP-ribosylation factor-binding protein GGA1 isoform 6	GGA1	26088	0.903	7	3	6	2	656	72.2	0.033897373
NP_001161819.1	probable 28S rRNA (cytosine-C(5))-methyltransferase isoform 3	NSUN5	55695	0.868	6	2	6	2	470	51	0.160556858
NP_291025.3	integrator complex subunit 4	INTS4	92105	0.817	3	2	6	2	963	108.1	0.046266859
NP_066923.3	cysteine desulfurase, mitochondrial isoform a precursor	NFS1	9054	1.283	5	2	6	2	457	50.2	0.163718786
XP_011537850.1	wings apart-like protein homolog isoform X1	WAPL	23063	1.109	4	3	6	3	1196	133.5	0.826944983
NP_001303903.1	protein LZIC isoform b	LZIC	84328	0.43	14	2	6	2	211	23.9	3.07E-05
XP_016885310.1	zinc finger protein 185 isoform X1	ZNF185	7739	1.011	8	4	5	4	766	81.4	0.876324818
XP_024305500.1	stonin-2 isoform X1	STON2	85439	0.848	3	2	16	2	977	108.9	0.263765175
XP_011514385.1	WD repeat-containing protein 91 isoform X1	WDR91	29062	0.599	4	3	8	3	792	88.3	0.267095481
NP_001274069.1	tumor protein D52 isoform 4	TPD52	7163	0.469	19	2	6	2	247	26.9	2.24E-05
NP_003163.1	surfeit locus protein 1 isoform 1	SURF1	6834	1.016	14	2	7	2	300	33.3	0.944398917
NP_003067.3	SWI/SNF-related matrix-associated actin-dependent regulator of chromatin subfamily D member 1 isoform a	SMARCD1	6602	0.97	5	3	5	3	515	58.2	0.99634671
NP_001164185.1	breast cancer anti-estrogen resistance protein 1 isoform 1	BCAR1	9564	0.825	5	3	9	3	916	97.8	0.74257496
NP_004690.1	protein FAM50A	FAM50A	9130	0.694	6	2	4	2	339	40.2	0.155766968
NP_057130.1	exosome complex component CSL4 isoform a	EXOSC1	51013	1.194	14	2	5	2	195	21.4	0.899158185
NP_001288763.1	protein C10 isoform 1	C12orf57	113246	0.62	29	2	5	2	126	13.2	0.402563403
NP_037519.2	cytochrome b-c1 complex subunit 9 isoform a	UQCR10	29796	1.216	38	2	6	2	63	7.3	0.002180584
NP_001217.2	caspase-6 isoform alpha precursor	CASP6	839	0.699	10	3	8	3	293	33.3	0.363509954
XP_011526372.1	DNA-directed RNA polymerases I, II, and III subunit RPABC1 isoform X1	POLR2E	5434	0.952	15	2	4	2	211	24.6	0.751954837
NP_001316607.1	protein PAXX isoform 1	PAXX	286257	0.687	12	2	6	2	223	23.8	0.270974705
NP_005756.2	renin receptor precursor	ATP6AP2	10159	1.19	10	2	4	2	350	39	0.874592556
NP_001269701.1	DNA-directed RNA polymerase I subunit RPA2 isoform 3	POLR1B	84172	0.901	1	1	6	1	1173	132.2	0.892773237
XP_016855611.1	tudor and KH domain-containing protein isoform X1	TDRKH	11022	0.865	8	3	3	3	561	62	0.984634943
NP_689940.3	copine-2	CPNE2	221184	0.662	6	3	10	2	548	61.2	0.029889544
NP_003724.1	2'-5'-oligoadenylate synthase-like protein isoform a	OASL	8638	0.84	7	3	6	3	514	59.2	0.756088708
XP_005264301.1	lysocardiolipin acyltransferase 1 isoform X1	LCLAT1	253558	1.26	9	3	4	3	414	48.9	0.719494707
NP_006629.2	ribonuclease P protein subunit p40 isoform a	RPP40	10799	0.752	9	2	4	2	363	41.8	0.565964284
XP_011527203.1	ADP-ribosylation factor GTPase-activating protein 1 isoform X1	ARFGAP1	55738	1.127	6	2	7	2	416	45.9	0.52967923
NP_001308264.1	selenoprotein H	SELENOH	280636	1.124	16	2	6	2	122	13.4	0.752432758
NP_061331.2	solute carrier family 25 member 40	SLC25A40	55972	0.905	12	2	4	2	338	38.1	0.320811281
NP_004796.1	DNA-directed RNA polymerase II subunit RPB4	POLR2D	5433	0.964	22	2	4	2	142	16.3	0.835731437
XP_024303791.1	lysosomal acid lipase/cholesteryl ester hydrolase isoform X1	LIPA	3988	0.364	5	3	6	3	399	45.4	0.283619483
NP_076931.1	ribosomal protein 63, mitochondrial	MRPL57	78988	1.555	14	2	4	2	102	12.3	0.245193775
NP_078796.2	m-AAA protease-interacting protein 1, mitochondrial precursor	MAIP1	79568	1.388	9	3	8	3	291	32.5	0.284444522
NP_004837.1	eukaryotic translation initiation factor 4E type 2 isoform A	EIF4E2	9470	0.6	11	2	6	2	245	28.3	0.002009022
NP_079428.2	iron-sulfur protein NUBPL isoform 1 precursor	NUBPL	80224	1.586	5	1	6	1	319	34.1	0.00336323
XP_011535460.1	calmin isoform X1	CLMN	79789	0.784	1	1	3	1	1033	115.3	0.044994381
XP_016863161.1	U6 snRNA-associated Sm-like protein LSm6 isoform X1	LSM6	11157	1.177	25	2	6	2	80	9.1	0.184596475
NP_031388.2	nuclear cap-binding protein subunit 2 isoform 1	NCBP2	22916	1.206	19	3	7	3	156	18	0.782517587
XP_016875895.1	ephrin-B2 isoform X1	EFNB2	1948	1.156	7	1	3	1	335	37.5	0.876971724
NP_057673.2	complex I assembly factor TIMMDC1, mitochondrial precursor	TIMMDC1	51300	1.387	12	2	4	2	285	32.2	0.084418226
NP_055217.2	cytochrome b-c1 complex subunit 8	UQCRQ	27089	1.316	30	3	11	3	82	9.9	0.28804763
NP_001072113.1	cell division cycle protein 16 homolog isoform 1	CDC16	8881	0.99	5	3	4	3	620	71.6	0.946809836
XP_011507899.1	RRP15-like protein isoform X1	RRP15	51018	1.271	8	3	7	3	368	40.7	0.722592081
NP_001166958.1	NF-kappa-B-repressing factor isoform 1	NKRF	55922	1.249	4	2	4	2	705	79.4	0.708471892
NP_054891.2	14 kDa phosphohistidine phosphatase isoform 3	PHPT1	29085	0.815	26	3	4	3	125	13.8	0.904708547
NP_006357.1	adenylyl cyclase-associated protein 2 isoform 1	CAP2	10486	0.57	8	3	15	1	477	52.8	0.00032408
NP_000294.1	phosphomannomutase 2	PMM2	5373	0.439	11	3	7	3	246	28.1	0.001356346
NP_001932.2	desmocollin-3 isoform Dsc3a preproprotein	DSC3	1825	1.453	5	3	7	3	896	99.9	0.631394455
NP_056518.2	ribosomal RNA-processing protein 7 homolog A	RRP7A	27341	1.465	12	2	5	2	280	32.3	0.020396406
NP_079477.2	pleckstrin homology domain-containing family O member 2 isoform 1	PLEKHO2	80301	0.657	5	1	3	1	490	53.3	0.001149788
NP_001002836.2	zinc finger protein 787 isoform 1	ZNF787	126208	1.366	3	1	1	1	382	40.4	0.758935617
NP_001185883.1	RNF103-CHMP3 protein	RNF103-CHMP3	100526767	0.342	5	1	6	1	251	28.4	7.54E-06
XP_011507514.1	BRO1 domain-containing protein BROX isoform X2	BROX	148362	0.675	7	3	10	3	432	48.2	0.13581024
NP_001304051.1	protein argonaute-1 isoform 1x	AGO1	26523	1.03	5	3	3	3	891	101.1	0.821081219
XP_005263588.1	uncharacterized protein KIAA2013 isoform X1	KIAA2013	90231	0.879	3	2	4	2	667	72.6	0.997383105
NP_001633.1	amyloid-like protein 2 isoform 1 precursor	APLP2	334	1.672	3	2	4	2	763	86.9	0.848090106
XP_024303296.1	bis(5'-nucleosyl)-tetraphosphatase	NUDT2	318	0.604	22	3	6	3	147	16.8	0.480475286
NP_001191676.1	replication factor C subunit 1 isoform 2	RFC1	5981	0.802	3	3	7	3	1148	128.2	0.791187721
NP_056139.1	ribosomal RNA-processing protein 8	RRP8	23378	1.379	5	2	6	2	456	50.7	0.002220271
XP_011542759.1	kinesin-like protein KIF13B isoform X1	KIF13B	23303	0.641	2	2	6	1	1847	205.1	0.003164833

Patil et al,2020. Proteomic and phosphoproteomic profiling of shammah-induced signaling in oral keratinocytes

Supplementary Table S1: List of proteins identified and quantified in OKF6/TERT1-Shammah and OKF6/TERT1-Parental total proteome using TMT-based quantitative proteomic approach

Accession	Protein Description	Gene Symbol	Gene ID	Fold Change	Coverage [%]	# Peptides	# PSMs	# Unique Peptides	# AAs	MW [kDa]	p-value
XP_006724806.1	four and a half LIM domains protein 1 isoform X1	FHL1	2273	0.488	7	2	5	2	339	37.9	0.226303175
XP_005247145.1	programmed cell death protein 10 isoform X1	PDCD10	11235	0.75	12	2	3	2	212	24.7	0.98910009
XP_005259627.3	zinc finger and BTB domain-containing protein 7A isoform X1	ZBTB7A	51341	1.015	5	2	4	2	634	66.9	0.920403091
XP_024306736.1	ras-related protein Rab-34 isoform X1	RAB34	83871	0.623	11	3	5	3	408	44.6	0.592045077
XP_011538818.1	interferon-induced protein 44 isoform X1	IFI44	10561	0.347	7	3	7	3	457	52	0.117794415
NP_001161339.1	distal membrane-arm assembly complex protein 2 isoform 1	DMAC2	55101	1.046	7	1	3	1	263	30.2	0.545039567
NP_001317455.1	putative hydroxypyruvate isomerase isoform 5	HYI	81888	0.773	7	1	6	1	302	33	0.07686935
NP_036332.2	galactosylgalactosylxylosylprotein 3-beta-glucuronosyltransferase 3 isoform 1	B3GAT3	26229	0.995	7	2	5	2	335	37.1	0.80766201
NP_115976.2	caspase recruitment domain-containing protein 6	CARD6	84674	0.633	3	2	5	2	1037	116.4	0.406102285
XP_016879276.2	putative oxidoreductase GLYR1 isoform X2	GLYR1	84656	1.349	6	3	6	3	580	63.1	0.732943499
XP_005260061.1	protein Wiz isoform X1	WIZ	58525	0.861	2	3	5	3	1889	203.4	0.977405112
XP_005273419.1	acyl-CoA:lysophosphatidylglycerol acyltransferase 1 isoform X1	LPGAT1	9926	1.314	6	2	5	2	403	46.6	0.260640761
NP_006611.1	HBS1-like protein isoform 1	HBS1L	10767	0.396	6	3	5	3	684	75.4	0.142654521
NP_073581.2	RUS1 family protein C16orf58	C16orf58	64755	1.157	5	1	5	1	468	51	0.638877405
XP_016885689.1	pyridoxal-dependent decarboxylase domain-containing protein 1 isoform X1	LOC102724985	102724985	0.82	3	2	5	2	835	91.6	0.67284384
XP_006715898.1	cyclin-dependent kinase 6 isoform X1	CDK6	1021	0.712	12	3	11	2	326	36.9	0.661411255
NP_057591.2	cyclin-dependent kinase 12 isoform 1	CDK12	51755	1.034	2	2	10	1	1490	164.1	0.604675889
XP_024302744.1	proteasome assembly chaperone 3 isoform X1	PSMG3	84262	0.474	12	1	3	1	122	13.1	0.008740253
NP_004672.2	eukaryotic translation initiation factor 1A, Y-chromosomal isoform 1	EIF1AY	9086	0.717	15	2	6	2	144	16.4	0.280230113
NP_002055.1	glutaredoxin-1	GLRX	2745	0.923	11	2	4	2	106	11.8	0.994824136
NP_001012533.1	ferrochelatase, mitochondrial isoform a precursor	FECH	2235	1.587	9	4	11	4	429	48.6	0.37932801
NP_004539.1	NADH dehydrogenase	NDUFB10	4716	1.241	12	2	6	2	172	20.8	0.045915739
NP_112192.2	protein unc-93 homolog B1	UNC93B1	81622	0.543	2	1	4	1	597	66.6	0.021867836
NP_078799.2	GRIP and coiled-coil domain-containing protein 1	GCC1	79571	1.079	3	1	3	1	775	87.8	0.774586922
NP_004614.3	tetratricopeptide repeat protein 4 isoform 1	TTC4	7268	0.513	7	2	5	2	387	44.7	0.270643585
NP_758869.1	membrane cofactor protein isoform 2 precursor	CD46	4179	1.666	10	3	7	3	399	44.2	0.352094214
XP_011517323.1	histone-lysine N-methyltransferase EHMT1 isoform X1	EHMT1	79813	1.483	3	3	4	3	1301	142	0.766947015
XP_024304353.1	WD repeat-containing protein 74 isoform X1	WDR74	54663	1.058	6	2	4	2	477	52.6	0.915545045
NP_055889.2	kinesin-like protein KIF1B isoform b	KIF1B	23095	1.38	2	3	6	1	1770	199.1	0.96574882
NP_077002.2	uncharacterized protein C1orf50	C1orf50	79078	0.595	8	1	3	1	199	21.9	0.000592913
NP_057542.2	protein HGH1 homolog	HGH1	51236	0.936	6	1	6	1	390	42.1	0.411123136
XP_011522899.1	protein TANC2 isoform X1	TANC2	26115	0.722	1	2	6	2	2075	228.8	0.124304071
XP_011537972.1	fucose mutarotase isoform X1	FUOM	282969	0.553	18	2	4	2	176	19.3	0.194409898
NP_056145.4	cytospin-A isoform 1	SPECC1L	23384	0.906	4	4	8	4	1117	124.5	0.718323053
NP_001257330.1	retinoic acid receptor RXR-beta isoform 1	RXRB	6257	1.279	6	2	6	1	537	57.3	0.401780285
XP_011513778.1	tensin-3 isoform X2	TNS3	64759	0.628	2	1	4	1	1561	167.9	0.582397031
NP_115716.1	ubiquinol-cytochrome-c reductase complex assembly factor 2	UQCC2	84300	1.369	15	2	8	2	126	14.9	0.429383698
XP_024302717.1	protein ABHD11 isoform X3	ABHD11	83451	0.854	5	2	6	2	434	47.3	0.238569901
NP_006266.2	serine/arginine-rich splicing factor 6	SRSF6	6431	1.286	7	3	12	2	344	39.6	0.03088344
NP_057688.2	lysine-specific demethylase 3B	KDM3B	51780	0.679	2	3	7	3	1761	191.5	0.827968321
XP_024307304.1	inositol-3-phosphate synthase 1 isoform X1	ISYNA1	51477	0.641	4	2	7	2	685	74.7	0.204638612
NP_060809.3	riboflavin kinase	RFK	55312	0.564	13	3	6	3	155	17.6	0.403628336
NP_004474.2	glycine cleavage system H protein, mitochondrial precursor	GCSH	2653	1.595	17	2	6	2	173	18.9	0.423331598
NP_060295.1	poly(ADP-ribose) glycohydrolase ARH3	ADPRHL2	54936	0.75	7	2	4	2	363	38.9	0.405693342
NP_000066.1	cyclin-dependent kinase 4	CDK4	1019	0.518	11	3	11	2	303	33.7	0.366161123
NP_001317003.1	WASH complex subunit 2C isoform 4	WASHC2C	253725	0.555	4	2	5	2	1341	147.1	0.367581657
XP_011540617.1	pre-mRNA-splicing factor 38A isoform X1	PRPF38A	84950	1.336	6	2	6	2	319	38.3	0.00123671
NP_004773.1	synaptosomal-associated protein 29	SNAP29	9342	0.609	12	3	7	3	258	29	0.143720717
NP_003867.1	transmembrane protein 11, mitochondrial	TMEM11	8834	1.234	21	4	10	4	192	21.5	0.100722642
NP_076933.3	prenylcysteine oxidase-like isoform 1 precursor	PCYOX1L	78991	0.84	5	3	5	3	494	54.6	0.734705964
NP_003070.3	SWI/SNF-related matrix-associated actin-dependent regulator of chromatin subfamily E member 1	SMARCE1	6605	0.937	4	1	6	1	411	46.6	0.078624141
NP_004290.2	ATP-binding cassette sub-family B member 7, mitochondrial isoform 1	ABCB7	22	1.593	8	3	6	3	753	82.7	0.199183057
XP_005260924.1	mitochondrial genome maintenance exonuclease 1 isoform X1	MGME1	92667	0.937	8	2	3	2	359	41.2	0.524316856
XP_005265804.1	exocyst complex component 1 isoform X1	EXOC1	55763	0.84	3	3	8	3	912	104.1	0.75176813
NP_075567.2	ubiquitin-conjugating enzyme E2 Z	UBE2Z	65264	0.818	8	3	5	3	354	38.2	0.754213013
NP_003950.1	huntingtin-interacting protein 1-related protein isoform 1	HIP1R	9026	1.071	3	2	2	2	1068	119.3	0.796106155
XP_011521281.1	group XV phospholipase A2 isoform X1	PLA2G15	23659	0.957	6	2	8	2	444	50.1	0.870715569
XP_016855889.1	zinc transporter 7 isoform X1	SLC30A7	148867	2.253	7	2	6	2	376	41.6	0.043371362
NP_061955.1	probable ATP-dependent RNA helicase DDX56 isoform 1	DDX56	54606	1.512	8	3	7	3	547	61.6	0.002105609
NP_631918.3	sn1-specific diacylglycerol lipase beta isoform 1	DAGLB	221955	0.971	5	2	4	2	672	73.7	0.952584999
XP_016867804.1	MAGUK p55 subfamily member 6 isoform X1	MPP6	51678	1.913	7	3	6	3	540	61.1	0.420740906
XP_024304767.1	vesicle transport protein GOT1B isoform X1	GOLT1B	51026	0.575	6	1	3	1	236	25.6	0.0208604
NP_005096.1	RNA-binding protein 8A	RBM8A	9939	0.912	16	3	11	3	174	19.9	0.680038217
NP_079021.3	SHC SH2 domain-binding protein 1 isoform 1	SHCBP1	79801	0.696	3	2	3	2	672	75.6	0.63874995
NP_057034.2	39S ribosomal protein L2, mitochondrial isoform 1	MRPL2	51069	1.264	7	2	4	2	305	33.3	0.584838557
NP_060557.3	nucleoporin NDC1 isoform 1	NDC1	55706	1.344	3	2	7	2	674	76.3	0.453412608
NP_004619.3	DNA repair protein complementing XP-C cells	XPC	7508	1.068	3	2	4	2	940	105.9	0.682295696
XP_005256923.1	nuclear receptor corepressor 1 isoform X1	NCOR1	9611	0.789	2	2	4	2	2524	279	0.038233372
NP_001224.1	caveolin-2 isoform a	CAV2	858	0.846	10	1	5	1	162	18.3	0.816210313
NP_005429.1	fos-related antigen 1 isoform 1	FOSL1	8061	1.064	14	3	4	3	271	29.4	0.883575926
NP_060851.2	repressor of yield of DENV protein isoform 1	C19orf66	55337	1.001	10	2	4	2	291	33.1	0.772692584
XP_011544166.1	leucine carboxyl methyltransferase 1 isoform X1	LCMT1	51451	1.078	7	3	5	3	357	41.1	0.903398002
XP_005271584.1	thymocyte nuclear protein 1 isoform X1	THYN1	29087	0.902	9	2	5	2	266	29.8	0.842917036
NP_060334.2	integrator complex subunit 8	INTS8	55656	1.103	4	2	3	2	995	113	0.77860998
XP_011526137.1	rho guanine nucleotide exchange factor 18 isoform X1	ARHGEF18	23370	0.79	2	2	6	1	1426	158.7	0.42586325
NP_001034229.1	UPF0687 protein C20orf27 isoform 1	C20orf27	54976	0.73	7	1	7	1	199	21.6	0.085602047
NP_001017980.1	vacuolar ATPase assembly integral membrane protein VMA21 isoform 1	VMA21	203547	1.027	12	1	5	1	101	11.3	0.958675091
NP_001307256.1	coiled-coil-helix-coiled-coil-helix domain-containing protein 2 precursor isoform 1	CHCHD2	51142	1.277	23	2	7	2	168	17.7	0.472647864
XP_016861535.1	arf-GAP with coiled-coil, ANK repeat and PH domain-containing protein 2 isoform X1	ACAP2	23527	0.857	3	2	5	2	848	96.1	0.771309862
NP_001265045.1	neural cell adhesion molecule L1 isoform 1 precursor	L1CAM	3897	1.581	4	3	6	3	1257	139.9	0.032730378
NP_004580.1	protein SCO1 homolog, mitochondrial	SCO1	6341	0.95	8	2	6	2	301	33.8	0.297054464
NP_005524.2	interferon-induced 35 kDa protein isoform 1	IFI35	3430	0.549	10	3	6	3	288	31.8	0.296918431
NP_001335007.1	lon protease homolog 2, peroxisomal isoform 3	LONP2	83752	1.694	3	2	6	2	906	100.9	0.357853459
NP_115725.1	tudor-interacting repair regulator protein isoform 1	NUDT16L1	84309	1.012	13	2	5	2	211	23.3	0.832488503
NP_003633.1	histone acetyltransferase type B catalytic subunit	HAT1	8520	0.915	7	2	5	2	419	49.5	0.559953991
NP_057444.2	translational activator of cytochrome c oxidase 1	TACO1	51204	1.2	20	3	7	3	297	32.5	0.995753295
XP_005270614.1	glycogen debranching enzyme isoform X1	AGL	178	0.705	3	3	5	3	1532	174.7	0.772951824
XP_016868047.1	serine/threonine-protein kinase B-raf isoform X1	BRAF	673	0.735	3	2	4	2	807	89.1	0.785030583
NP_000988.1	60S ribosomal protein L37	RPL37	6167	0.601	23	3	11	3	97	11.1	0.046949244
NP_071761.1	dimethyladenosine transferase 2, mitochondrial	TFB2M	64216	1.215	6	2	5	2	396	45.3	0.808294355
XP_016869464.1	nicotinate phosphoribosyltransferase isoform X1	NAPRT	93100	1.057	5	2	5	2	690	73.1	0.722144037
NP_001161403.1	interleukin-1 receptor accessory protein isoform 3 precursor	IL1RAP	3556	1.519	2	2	7	2	687	78.6	0.497206662
XP_024307962.1	tuftelin-interacting protein 11 isoform X1	TFIP11	24144	1.133	4	3	4	3	837	96.8	0.843769084
NP_001129107.1	UPF0696 protein C11orf68 isoform 1	C11orf68	83638	0.593	8	2	6	2	293	31.5	0.43644052
NP_659731.1	dual specificity mitogen-activated protein kinase kinase 3 isoform B	MAP2K3	5606	0.605	10	3	9	3	347	39.3	0.113866409
NP_068805.1	ragulator complex protein LAMTOR3 isoform 1	LAMTOR3	8649	0.693	27	2	3	2	124	13.6	0.873043293
NP_006720.1	protein diaphanous homolog 2 isoform 156	DIAPH2	1730	0.976	4	3	6	3	1101	125.5	0.947464231
NP_006237.1	serine/threonine-protein phosphatase 2A 56 kDa regulatory subunit epsilon isoform isoform a	PPP2R5E	5529	0.572	5	2	7	2	467	54.7	0.080437381
NP_001032354.1	geranylgeranyl pyrophosphate synthase	GGPS1	9453	0.525	7	2	4	2	300	34.8	0.004758838
NP_036453.1	U6 snRNA-associated Sm-like protein LSm4 isoform 1	LSM4	25804	1.092	20	2	7	2	139	15.3	0.174374743
NP_001243399.1	pleckstrin homology domain-containing family A member 5 isoform 4	PLEKHA5	54477	1.045	3	3	5	3	1282	146.8	0.910494511
NP_001121172.1	alpha-1-antitrypsin precursor	SERPINA1	5265	1.118	8	3	8	3	418	46.7	0.425178867
XP_024303669.1	cytosolic purine 5'-nucleotidase isoform X1	NT5C2	22978	0.754	9	3	6	3	569	65.8	0.226340429
NP_004522.1	molybdopterin synthase catalytic subunit large subunit MOCS2B	MOCS2	4338	0.366	8	1	4	1	188	20.9	0.001119863
XP_006712933.1	GRIP and coiled-coil domain-containing protein 2 isoform X1	GCC2	9648	0.966	2	3	6	2	1687	196.1	0.813202336
XP_005273819.1	carnitine O-palmitoyltransferase 1, liver isoform isoform X1	CPT1A	1374	1.322	3	2	5	2	805	91.8	0.76265025
NP_057155.1	28S ribosomal protein S33, mitochondrial	MRPS33	51650	1.392	20	2	6	2	106	12.6	0.009156933
NP_689633.1	carnosine N-methyltransferase isoform 1	CARNMT1	138199	0.543	5	2	6	2	409	47.2	0.000608113
NP_004779.2	ubiquitin conjugation factor E4 A isoform 1	UBE4A	9354	0.643	3	2	2	2	1073	123.4	0.204776679
NP_036606.2	uridine-cytidine kinase 2 isoform 1	UCK2	7371	0.751	10	2	5	2	261	29.3	0.511180875
NP_060822.2	translation machinery-associated protein 16	TMA16	55319	0.988	19	3	4	3	203	23.8	0.560150009
NP_001189476.1	SYNJ2BP-COX16 protein isoform 1	SYNJ2BP-COX16	100529257	1.424	11	2	5	2	191	21.3	0.610653732
XP_016867448.1	integrator complex subunit 1 isoform X1	INTS1	26173	1.088	2	3	4	3	2251	250.3	0.882072108
NP_689753.2	sec1 family domain-containing protein 2	SCFD2	152579	1.58	3	2	5	2	684	75.1	0.625705979
NP_006744.2	surfeit locus protein 6 isoform 1	SURF6	6838	1.347	10	3	7	3	361	41.4	0.274318636
NP_789782.1	charged multivesicular body protein 4b	CHMP4B	128866	0.806	14	3	5	3	224	24.9	0.667415207
NP_620137.2	F-BAR domain only protein 2 isoform a	FCHO2	115548	0.551	3	1	2	1	810	88.9	0.047803282
NP_001288005.1	AP-2 complex subunit sigma isoform 3	AP2S1	1175	0.869	17	3	10	3	158	18.9	0.560769374
NP_950241.1	diphosphoinositol polyphosphate phosphohydrolase 2 isoform beta	NUDT4	11163	0.676	13	2	2	2	181	20.4	0.733564907
NP_001341605.1	protein kinase C delta type isoform a	PRKCD	5580	0.639	5	3	5	3	695	79.6	0.613237089
NP_061961.2	RNA polymerase II-associated factor 1 homolog isoform 1	PAF1	54623	1.012	5	2	6	2	531	59.9	0.895488523
XP_011518702.1	fatty acyl-CoA reductase 1 isoform X1	FAR1	84188	0.85	5	2	3	2	518	59.8	0.622494265
NP_004330.1	pituitary tumor-transforming gene 1 protein-interacting protein isoform 1 precursor	PTTG1IP	754	0.706	10	1	4	1	180	20.3	0.227804196
XP_005265056.1	protein TASOR isoform X1	FAM208A	23272	1.04	2	4	6	4	1670	188.9	0.906310165
XP_011537724.1	dedicator of cytokinesis protein 1 isoform X1	DOCK1	1793	0.792	1	2	2	2	1908	219.8	0.830651088
NP_037466.2	mannose-1-phosphate guanyltransferase beta isoform 1	GMPPB	29925	0.927	10	3	5	3	387	42.6	0.863277971
NP_057651.1	BRCA2 and CDKN1A-interacting protein isoform BCCIPalpha	BCCIP	56647	0.655	10	2	7	2	322	36.2	0.003370761
NP_006236.1	serine/threonine-protein phosphatase 2A 56 kDa regulatory subunit delta isoform isoform 1	PPP2R5D	5528	0.208	3	2	6	2	602	69.9	1.42E-05
XP_011534642.1	gephyrin isoform X1	GPHN	10243	0.656	3	2	6	2	825	89.8	0.363256213
XP_011517276.1	TNF receptor-associated factor 2 isoform X1	TRAF2	7186	0.653	6	3	6	3	501	55.8	0.703372117
XP_005247653.2	mitofusin-1 isoform X1	MFN1	55669	1.263	3	2	6	1	741	84.1	0.227432907
NP_009096.2	splicing factor 3A subunit 2	SF3A2	8175	0.546	8	3	6	3	464	49.2	0.692097188
NP_056525.2	ribosome biogenesis protein NOP53	NOP53	29997	0.879	6	2	5	2	478	54.4	0.929050922
XP_011508705.1	arf-GAP with SH3 domain, ANK repeat and PH domain-containing protein 2 isoform X1	ASAP2	8853	0.707	3	3	7	3	1020	113.4	0.819924479
NP_001193541.1	chromatin target of PRMT1 protein isoform 2	CHTOP	26097	1.363	12	2	7	2	249	26.5	0.04064156
XP_011537005.1	signal transducer and activator of transcription 6 isoform X1	STAT6	6778	0.47	2	2	6	2	865	96.2	1.95E-05
NP_001157683.1	leucine-rich repeat and calponin homology domain-containing protein 1 isoform 1	LRCH1	23143	0.843	2	1	5	1	763	84.6	0.603793484
NP_001161206.1	valine--tRNA ligase, mitochondrial isoform 1	VARS2	57176	1.262	3	2	4	2	1093	121.5	0.604311048
XP_005258803.1	charged multivesicular body protein 2a isoform X1	CHMP2A	27243	0.354	10	2	5	2	265	29.6	0.002099937
NP_001186752.1	NEDD8-MDP1 protein	NEDD8-MDP1	100528064	0.484	7	1	5	1	193	22	0.005784536
XP_016871476.1	la-related protein 4B isoform X1	LARP4B	23185	1.272	2	1	2	1	1273	138.3	0.682416021
NP_001035521.1	cytochrome c oxidase assembly factor 3 homolog, mitochondrial	COA3	28958	1.152	19	2	5	2	106	11.7	0.800819657
NP_004626.1	MAP kinase-activated protein kinase 3	MAPKAPK3	7867	0.779	10	3	6	2	382	43	0.755352416
NP_001274.1	AP-1 complex subunit sigma-1A	AP1S1	1174	1.112	16	2	5	2	158	18.7	0.888342969
NP_055203.4	digestive organ expansion factor homolog	UTP25	27042	1.346	2	2	6	2	756	87	0.000617777
NP_079436.4	WD repeat-containing protein 26 isoform a	WDR26	80232	0.687	3	2	4	2	661	72.1	0.354066176
XP_011527824.1	C2 domain-containing protein 2 isoform X1	C2CD2	25966	1.195	2	1	4	1	707	76.6	0.48450736
NP_060017.1	FAD-dependent oxidoreductase domain-containing protein 1	FOXRED1	55572	1.075	5	2	9	2	486	53.8	0.133477285
NP_057715.2	PAX3- and PAX7-binding protein 1 isoform 1	PAXBP1	94104	1.037	3	2	6	2	917	104.7	0.943224604
NP_001092253.1	ribonuclease P protein subunit p14	RPP14	11102	0.792	13	1	4	1	124	13.7	0.361056855
XP_011521015.1	methyltransferase-like 26 isoform X1	METTL26	84326	0.592	9	2	4	2	271	29.5	0.017061998
NP_003155.2	syntaxin-5 isoform 1	STX5	6811	0.68	7	2	7	2	355	39.6	0.276927667
NP_057181.1	immediate early response 3-interacting protein 1 precursor	IER3IP1	51124	0.811	24	1	3	1	82	9	0.006866031
XP_016874894.1	cyclin-dependent kinase 17 isoform X1	CDK17	5128	1.09	5	3	12	2	523	59.5	0.882926105
NP_009135.4	probable ATP-dependent RNA helicase DDX20	DDX20	11218	0.632	5	2	8	2	824	92.2	0.695870856
NP_055870.2	switch-associated protein 70 isoform 1	SWAP70	23075	1.155	5	3	6	3	585	69	0.59409737
NP_001120664.1	cellular nucleic acid-binding protein isoform 1	CNBP	7555	0.981	8	1	9	1	179	19.7	0.601602712
XP_011512750.1	N(G),N(G)-dimethylarginine dimethylaminohydrolase 2 isoform X1	DDAH2	23564	1.071	17	3	5	3	285	29.6	0.909945725
NP_115688.1	protein YIPF4	YIPF4	84272	1.474	4	1	4	1	244	27.1	0.189087173
NP_006658.1	membrane-associated progesterone receptor component 1 isoform 1	PGRMC1	10857	1.475	21	3	8	3	195	21.7	0.004467824
NP_004854.1	serine palmitoyltransferase 2	SPTLC2	9517	1.114	8	3	5	3	562	62.9	0.883720519
NP_002026.1	3-ketodihydrosphingosine reductase precursor	KDSR	2531	2.173	8	2	3	2	332	36.2	0.95634159
NP_054885.1	mediator of RNA polymerase II transcription subunit 4 isoform 1	MED4	29079	0.917	10	2	4	2	270	29.7	0.916929669
NP_001161212.1	histone-lysine N-methyltransferase SMYD3 isoform 1	SMYD3	64754	0.714	8	2	5	2	428	49.1	0.823822373
XP_011508194.1	SHC-transforming protein 1 isoform X2	SHC1	6464	1.011	4	2	6	2	593	63.8	0.71922371
NP_061888.1	chondroitin sulfate glucuronyltransferase isoform 1 precursor	CHPF2	54480	1.193	2	1	3	1	772	85.9	0.03134701
XP_016866721.1	transcription factor AP-2-alpha isoform X1	TFAP2A	7020	0.997	7	2	3	2	519	55.6	0.954332294
NP_060416.1	peptidyl-prolyl cis-trans isomerase FKBP14 precursor	FKBP14	55033	1.358	6	2	4	2	211	24.2	0.753330323
NP_001026882.2	tRNA (guanine(10)-N2)-methyltransferase homolog isoform a	TRMT11	60487	0.837	6	2	5	2	463	53.4	0.547753424
NP_060919.3	ubiquitin-associated protein 2 isoform 1	UBAP2	55833	0.5	3	2	4	2	1119	117.1	0.386809134
NP_714916.3	KDEL motif-containing protein 2 isoform 1 precursor	KDELC2	143888	0.813	6	2	5	2	507	58.5	0.170849526
NP_443749.5	paralemmin-2 isoform a	PALM2	114299	1.63	10	3	7	1	411	45.4	0.63236951
NP_001296373.1	SPARC isoform 3 precursor	SPARC	6678	1.683	9	2	6	2	341	39.1	0.005445661
XP_016866973.1	WD repeat-containing protein 46 isoform X1	WDR46	9277	1.44	4	3	8	3	754	83.2	0.466274002
NP_612452.1	HAUS augmin-like complex subunit 1	HAUS1	115106	0.6	13	2	3	2	278	31.8	0.244725943
XP_011522362.1	rab GTPase-binding effector protein 1 isoform X1	RABEP1	9135	0.562	2	1	2	1	884	101.8	0.011921971
XP_005256652.2	dehydrogenase/reductase SDR family member 7B isoform X1	DHRS7B	25979	0.855	4	1	3	1	372	40.4	0.46253855
NP_001310552.1	nucleolar GTP-binding protein 2 isoform 2	GNL2	29889	0.823	3	2	4	2	799	91.2	0.911059754
NP_001311.3	casein kinase II subunit beta isoform 1	CSNK2B	1460	1.459	19	3	6	3	215	24.9	0.374001097
NP_000098.1	DNA damage-binding protein 2 isoform WT	DDB2	1643	1.754	5	2	4	2	427	47.8	0.047190022
NP_055321.1	torsin-1B isoform 1 precursor	TOR1B	27348	1.053	4	1	3	1	336	38	0.377171297
XP_011510679.1	monoglyceride lipase isoform X1	MGLL	11343	0.604	10	2	6	2	339	37.6	0.043371788
NP_060176.2	WD repeat-containing protein 55	WDR55	54853	1.07	6	2	5	2	383	42.1	0.830517754
NP_001349460.1	creatine kinase B-type isoform 2	CKB	1152	0.791	8	2	6	2	405	44.9	0.494014977
XP_016880222.1	rabankyrin-5 isoform X1	ANKFY1	51479	0.466	2	2	5	2	1273	138.7	0.07111672
NP_001180291.1	exonuclease 3'-5' domain-containing protein 2 isoform 1	EXD2	55218	1.535	5	2	4	2	621	70.3	0.252138764
XP_005256724.1	gem-associated protein 4 isoform X1	GEMIN4	50628	0.773	4	3	5	3	1062	120.4	0.774111473
NP_009208.2	exocyst complex component 3	EXOC3	11336	1.038	5	3	7	3	745	85.5	0.989486428
XP_011542237.1	GRIP1-associated protein 1 isoform X1	GRIPAP1	56850	0.922	5	3	5	3	851	97.1	0.847319406
XP_016877241.1	metastasis-associated protein MTA1 isoform X1	MTA1	9112	1.685	3	2	8	2	799	89.6	0.644214346
NP_060831.2	1-acyl-sn-glycerol-3-phosphate acyltransferase epsilon	AGPAT5	55326	1.245	9	2	5	2	364	42	0.020518166
NP_001290385.1	DNA-directed RNA polymerase III subunit RPC3 isoform 2	POLR3C	10623	0.799	3	1	3	1	547	62.1	0.005267675
NP_060193.2	torsin-4A	TOR4A	54863	1.602	7	3	6	3	423	46.9	0.038581596
NP_001307539.1	MICOS complex subunit MIC25 isoform 1	CHCHD6	84303	2.064	8	2	4	2	236	26.6	0.588206492
NP_065757.1	splicing factor, arginine/serine-rich 15 isoform 1	SCAF4	57466	1.502	5	4	9	1	1147	125.8	0.621977466
NP_064709.2	anamorsin isoform 1	CIAPIN1	57019	0.836	13	3	7	3	312	33.6	0.8994443
NP_005852.2	E3 ubiquitin-protein ligase CHIP isoform a	STUB1	10273	1.337	11	3	6	3	303	34.8	0.368100262
NP_036440.1	lysine-specific demethylase 2A isoform a	KDM2A	22992	1.134	2	2	5	2	1162	132.7	0.834274679
XP_016878181.1	synaptosomal-associated protein 23 isoform X1	SNAP23	8773	1.994	6	1	3	1	224	25.1	0.028184466
NP_006490.3	galectin-8 isoform a	LGALS8	3964	1.408	6	2	6	2	359	40.4	0.004846728
NP_001339.1	death-associated protein kinase 3	DAPK3	1613	1.005	4	2	6	2	454	52.5	0.973350925
NP_057383.2	heat shock 70 kDa protein 14 isoform 1	HSPA14	51182	0.474	4	2	5	2	509	54.8	0.785111013
NP_001288160.1	high mobility group protein B3 isoform b	HMGB3	3149	0.804	6	1	3	1	220	25.5	0.340229031
NP_061849.2	sodium-coupled neutral amino acid transporter 2 isoform 1	SLC38A2	54407	1.015	4	1	3	1	506	56	0.842899898
NP_001648.1	amphiregulin preproprotein	AREG	374	1.197	4	1	4	1	252	27.9	0.324467739
NP_000205.1	protein jagged-1 precursor	JAG1	182	2.56	4	3	7	3	1218	133.7	0.188081259
XP_005260739.1	leucine-rich repeat transmembrane protein FLRT3 isoform X1	FLRT3	23767	1.386	6	2	4	2	649	73	0.732345744
NP_005860.2	spliceosome-associated protein CWC27 homolog isoform 1	CWC27	10283	0.756	3	1	6	1	472	53.8	0.655479011
NP_057284.1	LSM8 homolog, U6 small nuclear RNA associated	LSM8	51691	0.969	17	1	3	1	96	10.4	0.97015293
XP_005263971.1	metal transporter CNNM4 isoform X1	CNNM4	26504	1.199	4	2	3	2	796	88.8	0.922803157
NP_073587.1	poly	PARP12	64761	0.958	5	3	8	3	701	79	0.933440747
NP_002406.1	macrophage migration inhibitory factor	MIF	4282	0.387	14	2	15	2	115	12.5	0.000277471
XP_016883731.1	double-stranded RNA-specific editase 1 isoform X1	ADARB1	104	2.618	5	3	4	3	790	86.6	0.132388363
XP_005245573.1	interferon-stimulated 20 kDa exonuclease-like 2 isoform X1	ISG20L2	81875	1.613	6	2	5	2	353	39.1	0.649945648
XP_011514525.1	hepatocyte growth factor receptor isoform X1	MET	4233	1.284	2	3	10	2	1409	157.6	0.009026335
NP_057322.2	anaphase-promoting complex subunit 7 isoform a	ANAPC7	51434	0.562	4	2	6	2	599	66.8	0.010252211
NP_004865.1	BAG family molecular chaperone regulator 4 isoform 1	BAG4	9530	0.913	5	2	7	1	457	49.6	0.555213609
XP_005274240.1	kinesin light chain 2 isoform X1	KLC2	64837	0.736	5	3	6	1	622	68.9	0.000300038
NP_055105.2	tudor domain-containing protein 7 isoform 1	TDRD7	23424	0.722	3	3	5	3	1098	123.5	0.164280429
NP_006223.2	DNA-directed RNA polymerases I, II, and III subunit RPABC3 isoform 2	POLR2H	5437	0.955	18	2	3	2	150	17.1	0.932643781
NP_079481.2	mediator of RNA polymerase II transcription subunit 28	MED28	80306	1.142	15	1	1	1	178	19.5	0.547449092
NP_060850.2	probable ATP-dependent RNA helicase DDX28	DDX28	55794	0.6	4	1	3	1	540	59.5	0.006471499
NP_056999.2	atlastin-1 isoform a	ATL1	51062	1.128	5	2	6	1	558	63.5	0.326050236
NP_055929.1	ankyrin repeat and LEM domain-containing protein 2	ANKLE2	23141	1.276	3	3	9	3	938	104.1	0.021355297
XP_016869077.1	pyruvate dehyrogenase phosphatase catalytic subunit 1 isoform X1	PDP1	54704	1.047	5	3	6	3	591	66.4	0.835996102
NP_061326.1	NADH dehydrogenase	NDUFA12	55967	1.067	17	2	6	2	145	17.1	0.940711818
NP_778236.2	ATP-dependent RNA helicase DDX51	DDX51	317781	1.504	4	2	4	2	666	72.4	0.692623907
NP_057538.1	ER membrane protein complex subunit 4 isoform a	EMC4	51234	0.847	13	2	6	2	183	20.1	0.141033886
NP_006075.3	interferon regulatory factor 9	IRF9	10379	0.87	7	2	6	2	393	43.7	0.869395889
NP_001305436.1	cytoplasmic tRNA 2-thiolation protein 2 isoform 3	CTU2	348180	0.557	6	2	4	2	586	63.6	0.961843995
NP_787117.3	UPF0600 protein C5orf51	C5orf51	285636	0.614	5	1	5	1	294	33.6	0.4145768
NP_689520.2	tRNA (adenine(58)-N(1))-methyltransferase catalytic subunit TRMT61A	TRMT61A	115708	0.916	6	1	3	1	289	31.4	0.064015928
NP_065823.1	nuclear fragile X mental retardation-interacting protein 2	NUFIP2	57532	0.717	5	2	3	2	695	76.1	0.891210223
NP_001092046.1	bax inhibitor 1 isoform 2	TMBIM6	7009	1.209	3	2	7	2	295	32.7	0.364155067
NP_005629.1	vesicle-associated membrane protein 7 isoform 1	VAMP7	6845	0.519	13	2	2	2	220	24.9	0.369674635
NP_067017.2	zinc transporter 1	SLC30A1	7779	1.242	5	2	6	2	507	55.3	0.010560221
NP_057151.1	28S ribosomal protein S18c, mitochondrial isoform 1	MRPS18C	51023	0.652	13	1	3	1	142	15.8	0.006190343
XP_005273547.1	inhibitor of nuclear factor kappa-B kinase subunit beta isoform X1	IKBKB	3551	0.698	2	2	5	2	805	91.8	0.204644659
NP_055449.1	protein phosphatase 1F	PPM1F	9647	0.592	7	2	8	2	454	49.8	0.004194495
XP_016865661.1	high mobility group nucleosome-binding domain-containing protein 4 isoform X1	HMGN4	10473	1.931	27	2	5	2	90	9.5	0.4943146
NP_000135.2	frataxin, mitochondrial isoform 1 preproprotein	FXN	2395	1.662	6	1	5	1	210	23.1	0.196637548
XP_024308819.1	mitotic-spindle organizing protein 2A isoform X4	MZT2A	653784	0.477	7	2	5	2	269	28	0.449609383
NP_006496.4	poliovirus receptor isoform alpha precursor	PVR	5817	1.573	5	2	5	2	417	45.3	0.486987663
NP_055054.1	translation initiation factor eIF-2B subunit beta	EIF2B2	8892	0.584	5	1	4	1	351	39	0.023101799
NP_071348.3	thyroid adenoma-associated protein isoform a	THADA	63892	0.983	2	2	5	2	1953	219.5	0.801898043
XP_005264506.2	baculoviral IAP repeat-containing protein 6 isoform X1	BIRC6	57448	0.588	1	2	3	2	4884	533	0.937610228
NP_071760.2	dnaJ homolog subfamily C member 1 precursor	DNAJC1	64215	1.019	5	2	4	2	554	63.8	0.985253899
NP_001188505.1	phospholipid scramblase 3	PLSCR3	57048	0.933	11	2	5	2	295	31.6	0.947883758
XP_011535987.1	protein sprouty homolog 4 isoform X1	SPRY4	81848	1.414	7	2	4	2	322	34.9	0.890498407
NP_976225.1	zinc finger Ran-binding domain-containing protein 2 isoform 1	ZRANB2	9406	1.067	10	3	6	3	330	37.4	0.932489985
NP_115890.2	acetyl-coenzyme A synthetase 2-like, mitochondrial isoform 1 precursor	ACSS1	84532	1.527	3	2	6	2	689	74.8	0.00644551
XP_011529011.1	myotubularin-related protein 5 isoform X1	SBF1	6305	0.755	2	2	3	2	1903	211.9	0.122652308
NP_055318.2	small subunit processome component 20 homolog	UTP20	27340	1.215	1	4	4	4	2785	318.2	0.815093477
NP_001350894.1	rab-like protein 3 isoform 1	RABL3	285282	1.583	17	2	3	2	250	28.1	0.787935886
XP_011513884.1	uncharacterized protein C7orf50 isoform X3	C7orf50	84310	0.997	16	3	6	3	225	25.2	0.772579568
NP_001026871.1	5'-nucleotidase domain-containing protein 3	NT5DC3	51559	1.57	4	2	5	2	548	63.4	0.349215513
NP_001271271.1	vitamin K epoxide reductase complex subunit 1-like protein 1 isoform 2	VKORC1L1	154807	1.156	6	1	3	1	177	19.7	0.524615484
NP_660304.1	mitochondrial import inner membrane translocase subunit TIM14 isoform 1	DNAJC19	131118	1.29	19	2	4	2	116	12.5	0.56082168
NP_057230.1	trafficking protein particle complex subunit 4 isoform 1	TRAPPC4	51399	0.753	9	2	5	2	219	24.3	0.665198029
NP_115570.1	ribosome production factor 2 homolog isoform 1	RPF2	84154	1.017	11	3	7	3	306	35.6	0.834441165
NP_071327.2	stromal cell-derived factor 2-like protein 1 precursor	SDF2L1	23753	1.507	10	2	7	2	221	23.6	0.085003883
NP_065101.1	something about silencing protein 10	UTP3	57050	1.402	4	2	7	2	479	54.5	0.000776337
XP_011543847.1	CTP synthase 2 isoform X1	CTPS2	56474	0.538	4	2	5	1	628	70.5	0.331571763
NP_000654.2	4-aminobutyrate aminotransferase, mitochondrial precursor	ABAT	18	1.332	3	1	6	1	500	56.4	0.007429933
XP_016869764.1	carnitine O-acetyltransferase isoform X1	CRAT	1384	2.024	4	2	6	2	627	71.1	0.00048367
NP_078801.3	tetratricopeptide repeat protein 13 isoform a	TTC13	79573	1.114	4	2	2	2	860	96.8	0.913006211
XP_016872786.1	probable methyltransferase-like protein 15 isoform X1	METTL15	196074	1.027	4	1	3	1	407	46.1	0.907616868
NP_005116.1	copper chaperone for superoxide dismutase	CCS	9973	1.078	8	2	6	2	274	29	0.148988113
NP_004540.1	NADH dehydrogenase	NDUFC2	4718	1.733	16	2	4	2	119	14.2	0.466049025
XP_011527050.1	activity-dependent neuroprotector homeobox protein isoform X1	ADNP	23394	1.471	2	1	6	1	1111	124.6	0.002435438
NP_076955.1	DET1- and DDB1-associated protein 1	DDA1	79016	1.17	31	3	6	3	102	11.8	0.754036009
NP_065939.2	uncharacterized protein KIAA1522 isoform 1	KIAA1522	57648	1.095	3	2	5	2	1094	113	0.75086609
NP_060060.3	zinc finger CCCH domain-containing protein 7B	ZC3H7B	23264	0.647	3	2	6	2	977	109.8	0.020317813
NP_954580.1	transmembrane protein 189 isoform 1	TMEM189	387521	1.555	8	2	5	1	270	31.1	0.015226608
NP_055288.1	probable dimethyladenosine transferase isoform 1	DIMT1	27292	0.833	17	3	4	3	313	35.2	0.794280788
XP_016874530.1	protein MON2 homolog isoform X1	MON2	23041	0.487	2	2	5	2	1718	190.4	0.357589906

Patil et al,2020. Proteomic and phosphoproteomic profiling of shammah-induced signaling in oral keratinocytes

Supplementary Table S1: List of proteins identified and quantified in OKF6/TERT1-Shammah and OKF6/TERT1-Parental total proteome using TMT-based quantitative proteomic approach

Accession	Protein Description	Gene Symbol	Gene ID	Fold Change	Coverage [%]	# Peptides	# PSMs	# Unique Peptides	# AAs	MW [kDa]	p-value
XP_016868782.1	zinc transporter ZIP14 isoform X1	SLC39A14	23516	1.186	6	2	3	2	502	55.2	0.857421146
NP_001308062.1	Golgi SNAP receptor complex member 2 isoform D	GOSR2	9570	1.16	11	2	4	2	257	29.8	0.657084091
NP_859052.3	sulfhydryl oxidase 2 precursor	QSOX2	169714	1.08	2	1	4	1	698	77.5	0.77251905
NP_004844.1	syntaxin-8	STX8	9482	1.042	8	1	2	1	236	26.9	0.319539477
NP_006318.1	mitochondrial import inner membrane translocase subunit Tim23	TIMM23	100287932	1.021	8	1	4	1	209	21.9	0.895949189
XP_005274808.1	lys-63-specific deubiquitinase BRCC36 isoform X2	BRCC3	79184	0.756	8	2	4	2	317	36.1	0.000333868
NP_006799.1	protein transport protein Sec61 subunit beta	SEC61B	10952	0.797	22	2	4	2	96	10	0.628556844
XP_011510535.1	FERM, ARHGEF and pleckstrin domain-containing protein 2 isoform X1	FARP2	9855	1.67	2	2	3	2	1061	120.6	0.573556435
NP_000047.1	2-oxoisovalerate dehydrogenase subunit beta, mitochondrial isoform 1 precursor	BCKDHB	594	0.984	9	2	4	2	392	43.1	0.932067461
NP_004242.1	ras-related protein Rab-9A	RAB9A	9367	0.799	7	2	2	2	201	22.8	0.877364053
NP_004121.2	glycogenin-1 isoform 1	GYG1	2992	0.664	5	2	6	2	350	39.4	0.117425613
NP_055779.2	epsin-2 isoform b	EPN2	22905	1.072	3	2	7	1	641	68.4	0.833590274
NP_055426.1	midasin	MDN1	23195	1.858	1	3	4	3	5596	632.4	0.780609404
NP_689939.1	calcium uptake protein 2, mitochondrial	MICU2	221154	1.235	4	1	6	1	434	49.6	0.048835743
NP_000782.1	dihydrofolate reductase isoform 1	DHFR	1719	0.372	12	2	5	2	187	21.4	0.0194811
XP_005249035.1	mitochondrial carrier homolog 1 isoform X1	MTCH1	23787	1.313	5	2	5	2	427	46.3	0.159288243
NP_001158312.1	LYR motif-containing protein 4 isoform 2	LYRM4	57128	1.996	11	1	1	1	130	15.2	0.969544749
XP_011543091.1	serine/threonine-protein kinase MARK2 isoform X1	MARK2	2011	0.683	2	2	8	1	808	89.8	0.123627097
NP_037458.3	regulator of MON1-CCZ1 complex isoform 1	RMC1	29919	0.697	2	1	3	1	657	74.9	0.022359956
NP_004364.2	cytochrome c oxidase subunit 6A1, mitochondrial	COX6A1	1337	0.748	27	1	3	1	109	12.1	0.948836805
NP_001478.2	biogenesis of lysosome-related organelles complex 1 subunit 1	BLOC1S1	2647	0.814	26	2	4	2	153	17.3	0.584605705
NP_536724.1	phosphatidylinositol glycan anchor biosynthesis class U protein precursor	PIGU	128869	0.88	6	2	4	2	435	50	0.916742431
NP_112196.3	TRMT1-like protein isoform 1	TRMT1L	81627	0.648	5	2	4	2	733	81.7	0.791647449
XP_011514891.1	E3 ubiquitin-protein ligase TRIM56 isoform X1	TRIM56	81844	0.755	4	2	2	2	755	81.4	0.969447043
XP_011507753.1	ceramide synthase 2 isoform X1	CERS2	29956	0.989	4	1	4	1	400	47	0.780658363
NP_056157.1	peptidylprolyl isomerase domain and WD repeat-containing protein 1 isoform 1	PPWD1	23398	0.956	5	2	4	2	646	73.5	0.983792125
XP_005262198.1	armadillo repeat-containing X-linked protein 3 isoform X1	ARMCX3	51566	2.081	5	2	6	2	379	42.5	3.06E-06
XP_016864892.1	general transcription factor IIH subunit 2 isoform X1	GTF2H2	2966	1.291	2	1	3	1	395	44.4	0.823618823
NP_061176.3	probable 2-oxoglutarate dehydrogenase E1 component DHKTD1, mitochondrial	DHTKD1	55526	1.416	3	2	5	2	919	103	0.870586488
NP_116204.3	NFATC2-interacting protein	NFATC2IP	84901	0.519	6	2	7	2	419	45.8	0.093701447
XP_024304347.1	multifunctional methyltransferase subunit TRM112-like protein isoform X1	TRMT112	51504	0.611	33	3	3	3	125	14.2	0.720547045
XP_011524074.1	THO complex subunit 1 isoform X1	THOC1	9984	1.222	5	2	2	2	664	76.4	0.739401845
NP_002481.3	NADH dehydrogenase	NDUFA6	4700	1.28	14	2	6	2	128	15.1	0.216537834
NP_065956.1	retinol dehydrogenase 14	RDH14	57665	1.455	7	3	7	3	336	36.8	0.508331751
XP_016862206.1	anoctamin-10 isoform X1	ANO10	55129	1.491	1	1	3	1	699	80.6	0.719616842
NP_001248340.1	splicing factor, suppressor of white-apricot homolog isoform 1	SFSWAP	6433	1.185	2	2	2	2	1003	109.9	0.923425872
NP_001265478.1	[Pyruvate dehydrogenase (acetyl-transferring)] kinase isozyme 1, mitochondrial isoform 1 precursor	PDK1	5163	1.363	3	1	3	1	456	51.6	0.02688297
NP_660213.1	39S ribosomal protein L30, mitochondrial precursor	MRPL30	51263	1.201	12	2	6	2	161	18.5	0.035116651
NP_001340295.1	nucleolar MIF4G domain-containing protein 1 isoform 2	NOM1	64434	1.267	3	2	5	2	861	96.3	0.007438203
NP_001273053.1	serine/threonine-protein kinase MARK1 isoform 1	MARK1	4139	1.038	2	2	8	1	796	89	0.818560097
NP_055859.1	ADP-ribosylation factor-binding protein GGA2	GGA2	23062	0.708	6	2	5	1	613	67.1	0.702368046
NP_068839.1	integral membrane protein 2B	ITM2B	9445	1.369	14	2	2	2	266	30.3	0.893776094
NP_001352193.1	epithelial splicing regulatory protein 2 isoform 2	ESRP2	80004	0.704	5	2	4	1	727	78.4	0.842044438
NP_536859.1	tyrosine-protein phosphatase non-receptor type 6 isoform 3	PTPN6	5777	0.592	5	2	6	2	624	70.1	0.326602247
NP_060932.2	protein BRICK1	BRK1	55845	0.843	27	2	6	2	75	8.7	0.033740626
XP_005245737.1	rab GTPase-activating protein 1-like isoform X1	RABGAP1L	9910	0.418	3	2	4	1	1051	119.4	1.16E-05
NP_036580.2	signal transducer and activator of transcription 5B	STAT5B	6777	0.662	2	1	3	1	787	89.8	0.169021136
XP_005246217.1	hippocalcin-like protein 1 isoform X1	HPCAL1	3241	1.797	9	2	6	2	193	22.3	3.67E-06
NP_054896.2	mitochondrial import inner membrane translocase subunit Tim21 precursor	TIMM21	29090	1.247	6	1	2	1	248	28.2	0.737204631
NP_060404.4	PH-interacting protein	PHIP	55023	0.89	2	3	6	3	1821	206.6	0.899650829
NP_071396.4	kinesin-like protein KIF13A isoform a	KIF13A	63971	0.541	1	1	4	1	1805	202.2	0.366694611
NP_077003.1	coiled-coil domain-containing protein 86	CCDC86	79080	1.144	6	1	2	1	360	40.2	0.969263616
XP_005246794.1	RNA-binding motif, single-stranded-interacting protein 1 isoform X1	RBMS1	5937	1.231	7	2	5	2	422	46.3	0.31390156
NP_001350422.1	solute carrier family 12 member 9 isoform 1	SLC12A9	56996	0.848	5	3	4	3	914	96	0.498552353
XP_016874541.1	ELKS/Rab6-interacting/CAST family member 1 isoform X1	ERC1	23085	0.915	3	2	6	2	1141	131	0.91052484
XP_011527345.1	alpha-tocopherol transfer protein-like isoform X1	TTPAL	79183	0.79	9	2	3	2	342	38.5	0.692169088
NP_056201.2	conserved oligomeric Golgi complex subunit 4 isoform 1	COG4	25839	0.781	2	1	3	1	789	89.4	0.174281335
NP_073613.2	oxysterol-binding protein-related protein 11	OSBPL11	114885	0.589	3	2	7	1	747	83.6	0.660333338
NP_055186.3	A-kinase anchor protein 8-like isoform 1	AKAP8L	26993	1.052	2	2	3	2	646	71.6	0.920154408
XP_005258127.1	Golgi SNAP receptor complex member 1 isoform X1	GOSR1	9527	1.158	7	2	5	2	300	34.5	0.98267561
XP_005264880.1	ubiquitin carboxyl-terminal hydrolase 19 isoform X1	USP19	10869	0.654	1	1	3	1	1423	156.3	0.684811677
NP_057541.2	serine/threonine-protein kinase D2 isoform A	PRKD2	25865	0.743	5	3	5	3	878	96.7	0.477820123
NP_001130010.1	acyl-CoA dehydrogenase family member 10 isoform a	ACAD10	80724	0.74	2	2	6	2	1090	122.3	0.002075454
NP_001265210.1	glycosyltransferase 8 domain-containing protein 1	GLT8D1	55830	1.223	6	2	5	2	371	41.9	0.841848646
NP_008955.1	transmembrane protein 115	TMEM115	11070	1.219	5	1	2	1	351	38.2	0.901299139
XP_024301960.1	bromodomain-containing protein 9 isoform X1	BRD9	65980	2.991	4	2	4	2	617	69.4	0.014969358
NP_001333158.1	lisH domain and HEAT repeat-containing protein KIAA1468 isoform a	RELCH	57614	0.613	2	2	5	2	1250	138.7	0.254720561
XP_016880808.1	cytospin-B isoform X3	SPECC1	92521	1.011	1	1	6	1	1076	119.4	0.882814885
NP_115717.3	protein DDI1 homolog 2	DDI2	84301	0.742	5	1	2	1	399	44.5	0.673938176
NP_000276.2	xaa-Pro dipeptidase isoform 1	PEPD	5184	0.495	10	2	4	2	493	54.5	0.521118554
NP_002344.2	tumor-associated calcium signal transducer 2 precursor	TACSTD2	4070	1.423	10	2	3	2	323	35.7	0.701273512
NP_003087.1	small nuclear ribonucleoprotein G isoform a	SNRPG	6637	1.27	26	2	10	2	76	8.5	0.135493785
XP_005270993.1	E3 ubiquitin-protein ligase TRIM33 isoform X1	TRIM33	51592	1.026	1	1	2	1	1151	125.3	0.878013756
NP_006849.1	transmembrane emp24 domain-containing protein 1 precursor	TMED1	11018	1.164	11	2	4	2	227	25.2	0.856090595
NP_003667.1	sphingolipid delta(4)-desaturase DES1 isoform 1	DEGS1	8560	0.894	5	1	5	1	323	37.8	0.550853839
XP_024306436.1	exocyst complex component 7 isoform X1	EXOC7	23265	0.772	3	2	4	2	763	86.2	0.898659434
NP_055976.1	ADP-ribosylation factor-like protein 6-interacting protein 1 isoform 1	ARL6IP1	23204	1.323	5	1	4	1	203	23.3	0.212369941
NP_001007271.1	gem-associated protein 7	GEMIN7	79760	1.156	21	1	1	1	131	14.5	0.298455164
NP_055134.2	inner nuclear membrane protein Man1 isoform 1	LEMD3	23592	1.59	4	2	4	2	911	99.9	0.122013312
NP_004271.1	eukaryotic translation elongation factor 1 epsilon-1 isoform 1	EEF1E1	9521	0.699	13	2	6	2	174	19.8	0.01626797
NP_001004125.1	tumor suppressor candidate gene 1 protein	TUSC1	286319	0.943	11	2	3	2	212	23.4	0.51625129
NP_689929.2	protein PAT1 homolog 1	PATL1	219988	0.599	5	2	5	2	770	86.8	0.160346719
NP_008879.3	heat shock 70 kDa protein 13 precursor	HSPA13	6782	1.628	2	1	6	1	471	51.9	0.00130661
XP_011541659.1	3-hydroxy-3-methylglutaryl-Coenzyme A reductase isoform X1	HMGCR	3156	1.301	3	2	4	2	908	99.7	0.647727604
XP_024307296.1	nitric oxide synthase-interacting protein isoform X1	NOSIP	51070	0.498	4	1	5	1	348	37.9	0.003125939
NP_851320.1	integral membrane protein GPR180 precursor	GPR180	160897	1.391	6	2	5	2	440	49.4	0.566448301
NP_002399.1	alpha-1,6-mannosyl-glycoprotein 2-beta-N-acetylglucosaminyltransferase	MGAT2	4247	0.697	5	2	4	2	447	51.5	0.43950064
NP_038476.2	bromodomain adjacent to zinc finger domain protein 1A isoform a	BAZ1A	11177	0.676	2	2	3	2	1556	178.6	0.70456985
NP_001847.3	collagen alpha-1(XVI) chain precursor	COL16A1	1307	0.837	2	2	4	2	1604	157.7	0.927072725
XP_016860992.1	RNA-binding protein 5 isoform X1	RBM5	10181	0.884	3	2	6	1	815	92.1	0.058200762
XP_005267280.1	mediator of RNA polymerase II transcription subunit 23 isoform X1	MED23	9439	1.283	3	3	8	3	1374	157	0.039189924
NP_001003891.1	mediator of RNA polymerase II transcription subunit 15 isoform a	MED15	51586	0.946	5	2	4	2	788	86.7	0.641855077
NP_001231.2	cyclin-T1 isoform a	CCNT1	904	1.489	4	3	5	3	726	80.6	0.831212486
NP_057297.2	activating signal cointegrator 1 isoform 1	TRIP4	9325	0.661	5	3	6	3	581	66.1	0.007217299
NP_056380.2	E3 ubiquitin-protein ligase listerin isoform 1	LTN1	26046	0.494	1	1	2	1	1812	205	0.045767653
XP_016867667.1	integrin beta-8 isoform X1	ITGB8	3696	2.17	2	1	5	1	769	85.6	0.005188221
XP_011530785.1	STAM-binding protein isoform X1	STAMBP	10617	0.767	4	1	4	1	424	48	0.579781982
NP_006360.3	leucine-rich repeat-containing protein 41	LRRC41	10489	0.892	4	1	4	1	812	88.6	0.854137301
XP_016855991.1	2-(3-amino-3-carboxypropyl)histidine synthase subunit 2 isoform X1	DPH2	1802	0.606	9	2	4	2	495	52.9	0.223656934
NP_001298240.1	vitamin K epoxide reductase complex subunit 1 isoform 3 precursor	VKORC1	79001	1.996	7	1	3	1	191	21.5	0.158013724
NP_115664.2	UAP56-interacting factor isoform 1	FYTTD1	84248	1.342	4	1	6	1	318	35.8	0.068648128
NP_055580.1	mitochondrial import receptor subunit TOM20 homolog	TOMM20	9804	2.582	16	2	6	2	145	16.3	0.000150598
NP_060441.2	39S ribosomal protein L20, mitochondrial isoform 1	MRPL20	55052	1.346	14	2	5	2	149	17.4	0.096036002
NP_001366.1	deoxyribonuclease-2-alpha precursor	DNASE2	1777	1.243	5	2	4	2	360	39.6	0.717209666
NP_073557.3	microprocessor complex subunit DGCR8 isoform 1	DGCR8	54487	0.722	3	2	4	2	773	86	0.350658583
NP_542161.1	RNA-binding protein with serine-rich domain 1 isoform a	RNPS1	10921	1.185	9	2	6	2	305	34.2	0.108511808
NP_001035919.1	transforming growth factor beta-1-induced transcript 1 protein isoform 1	TGFB1I1	7041	0.601	6	2	4	2	461	49.8	0.371142656
XP_005247639.1	presenilins-associated rhomboid-like protein, mitochondrial isoform X1	PARL	55486	0.77	7	2	5	2	394	43.4	0.643230308
NP_004076.1	mitochondrial import inner membrane translocase subunit Tim8 A isoform 1	TIMM8A	1678	1.611	23	2	6	2	97	11	0.002778144
NP_057185.1	60S ribosome subunit biogenesis protein NIP7 homolog isoform 1	NIP7	51388	0.658	16	2	6	2	180	20.4	0.000113528
XP_006717199.1	28S ribosomal protein S2, mitochondrial isoform X1	MRPS2	51116	1.4	5	2	6	2	319	35.5	0.006339527
NP_001304712.1	GDP-L-fucose synthase isoform 1	TSTA3	7264	0.529	6	1	3	1	327	36.6	0.61995537
NP_659400.2	protein FAM91A1 isoform 1	FAM91A1	157769	0.887	4	3	6	3	838	93.9	0.753141908
XP_006721594.1	misshapen-like kinase 1 isoform X1	MINK1	50488	0.828	3	3	5	3	1361	153.4	0.740162824
NP_060116.2	tRNA dimethylallyltransferase isoform 1 precursor	TRIT1	54802	0.826	3	1	3	1	467	52.7	0.082199355
XP_016861135.1	CKLF-like MARVEL transmembrane domain-containing protein 7 isoform X2	CMTM7	112616	1.079	18	1	3	1	179	19.5	0.38025993
NP_004888.2	multiple inositol polyphosphate phosphatase 1 isoform 1 precursor	MINPP1	9562	1.671	2	1	4	1	487	55	0.001264163
NP_001123917.1	sperm-specific antigen 2 isoform 1	ITPRID2	6744	0.965	1	1	4	1	1259	138.3	0.881686409
XP_024307330.1	coiled-coil and C2 domain-containing protein 1A isoform X1	CC2D1A	54862	1.078	2	1	2	1	982	107.3	0.920147151
NP_031376.3	pleckstrin homology-like domain family A member 1	PHLDA1	22822	0.619	2	1	5	1	401	45	0.449353473
NP_078895.2	ketosamine-3-kinase	FN3KRP	79672	0.591	8	2	2	2	309	34.4	0.546418094
NP_001317672.1	choline/ethanolaminephosphotransferase 1	CEPT1	10390	1.425	3	1	7	1	416	46.5	0.168440084
NP_037453.1	sorting nexin-8	SNX8	29886	1.18	3	1	2	1	465	52.5	0.997380253
NP_004255.2	mediator of RNA polymerase II transcription subunit 21 isoform 1	MED21	9412	1.256	10	1	2	1	144	15.6	0.385882448
NP_079047.2	N-alpha-acetyltransferase 40 isoform 1	NAA40	79829	0.625	5	1	5	1	237	27.2	0.243071505
NP_056224.3	E1A-binding protein p400	EP400	57634	1.165	0	1	3	1	3123	339.6	0.062155527
XP_005265513.1	sarcolemmal membrane-associated protein isoform X1	SLMAP	7871	1.616	6	3	5	3	847	97.1	0.377163443
NP_001289979.1	microtubule-associated protein RP/EB family member 3	MAPRE3	22924	0.92	7	2	5	2	281	32	0.784972854
NP_777569.1	protein kish-A precursor	TMEM167A	153339	0.73	25	2	5	2	72	8.1	0.516233917
NP_683685.1	39S ribosomal protein L10, mitochondrial isoform b	MRPL10	124995	1.029	7	1	3	1	271	30.3	0.544840518
XP_011521075.1	telomere length regulation protein TEL2 homolog isoform X1	TELO2	9894	0.759	4	2	4	2	899	98.6	0.355070992
NP_149075.2	Fanconi anemia group D2 protein isoform a	FANCD2	2177	0.935	1	1	1	1	1471	166.4	0.514376448
NP_002938.1	replication protein A 14 kDa subunit	RPA3	6119	0.611	14	1	4	1	121	13.6	0.134891245
XP_005246910.1	secernin-3 isoform X1	SCRN3	79634	0.332	2	1	3	1	444	50.7	0.013947286
NP_002964.3	ataxin-2 isoform 1	ATXN2	6311	0.784	1	1	1	1	1313	140.2	0.791369653
NP_056464.1	interferon regulatory factor 2-binding protein 1	IRF2BP1	26145	1.318	4	2	5	2	584	61.6	0.041082508
NP_068810.3	transcription factor p65 isoform 1	RELA	5970	0.869	4	2	4	2	551	60.2	0.82003698
NP_001335361.1	zinc finger protein 148 isoform a	ZNF148	7707	0.837	2	1	3	1	794	88.9	0.113976278
XP_024308987.1	U3 small nucleolar ribonucleoprotein protein IMP4 isoform X1	IMP4	92856	1.789	6	2	5	2	319	36.7	0.21336277
NP_002484.1	NADH dehydrogenase	NDUFB6	4712	1.023	22	2	4	2	128	15.5	0.864832555
NP_060680.2	ATP-dependent (S)-NAD(P)H-hydrate dehydratase isoform a	NAXD	55739	0.765	3	1	2	1	390	41.3	0.977300062
NP_077001.1	dCTP pyrophosphatase 1	DCTPP1	79077	0.764	14	2	5	2	170	18.7	0.113213995
NP_008924.1	nucleoplasmin-3	NPM3	10360	0.619	12	1	5	1	178	19.3	0.018658319
XP_011540741.1	rho GTPase-activating protein 29 isoform X1	ARHGAP29	9411	0.471	1	1	3	1	1261	142	1.56E-05
NP_006400.2	actin-related protein 2/3 complex subunit 1A isoform 1	ARPC1A	10552	0.992	5	2	6	2	370	41.5	0.98558054
NP_001310260.1	mitogen-activated protein kinase 8 isoform alpha2	MAPK8	5599	0.45	5	2	6	1	427	48.3	8.30E-05
NP_079160.1	L-2-hydroxyglutarate dehydrogenase, mitochondrial precursor	L2HGDH	79944	1.588	6	3	6	3	463	50.3	0.231531047
XP_006717584.1	neuropilin-1 isoform X1	NRP1	8829	1.565	2	1	3	1	924	103.1	0.003085768
XP_005261794.1	cysteine-rich with EGF-like domain protein 2 isoform X1	CRELD2	79174	1.275	4	1	2	1	403	43.6	0.371367847
NP_149103.1	ribosomal RNA processing protein 36 homolog isoform 1	RRP36	88745	1.547	6	1	2	1	259	29.8	0.455814421
NP_932070.1	BH3-interacting domain death agonist isoform 1	BID	637	0.794	6	1	3	1	241	26.8	0.783448688
NP_689481.2	probable proline--tRNA ligase, mitochondrial precursor	PARS2	25973	0.79	9	2	5	2	475	53.2	0.64859264
NP_057143.1	peptidyl-prolyl cis-trans isomerase-like 1	PPIL1	51645	1.039	19	3	9	3	166	18.2	0.678876519
NP_001091977.1	BUB3-interacting and GLEBS motif-containing protein ZNF207 isoform c	ZNF207	7756	1.088	7	3	7	3	494	52.7	0.937529496
NP_055640.2	nucleolar pre-ribosomal-associated protein 1	URB1	9875	0.404	2	2	4	2	2271	254.2	0.292442467
NP_001310960.1	semaphorin-4B isoform 4	SEMA4B	10509	2.101	2	1	4	1	890	98	0.000351608
NP_597677.2	nipped-B-like protein isoform A	NIPBL	25836	0.849	1	2	4	2	2804	315.9	0.738156654
NP_073568.2	nuclear ubiquitous casein and cyclin-dependent kinase substrate 1	NUCKS1	64710	0.386	15	3	5	3	243	27.3	0.086208287
NP_542417.2	protein PRRC2A	PRRC2A	7916	0.831	1	1	1	1	2157	228.7	0.893077272
NP_068579.3	exocyst complex component 4 isoform a	EXOC4	60412	0.808	4	3	3	3	974	110.4	0.791426259
NP_001350747.1	metaxin-3 isoform 3	MTX3	345778	1.236	9	3	6	3	312	35.1	0.538966304
NP_005054.3	acyl-CoA desaturase	SCD	6319	0.538	4	1	2	1	359	41.5	0.620275714
XP_005245072.1	protein PRRC2C isoform X1	PRRC2C	23215	1.071	1	3	5	3	2903	317.5	0.777638662
NP_055647.2	TBC1 domain family member 4 isoform 1	TBC1D4	9882	0.711	2	2	3	2	1298	146.5	0.805865791
NP_001308832.1	very-long-chain (3R)-3-hydroxyacyl-CoA dehydratase 4 isoform 3	HACD4	401494	1.592	4	2	5	2	283	33.1	0.046122334
XP_011507381.1	influenza virus NS1A-binding protein isoform X1	IVNS1ABP	10625	1.113	3	2	3	2	649	72.4	0.881450026
NP_002426.1	mannose-6-phosphate isomerase isoform 1	MPI	4351	0.657	3	1	3	1	423	46.6	0.116110769
NP_031372.2	opioid growth factor receptor	OGFR	11054	0.79	2	1	5	1	677	73.3	0.602962684
XP_016863819.1	COMM domain-containing protein 8 isoform X1	COMMD8	54951	0.534	6	1	3	1	189	21.7	0.003807013
NP_006414.2	prenylated Rab acceptor protein 1	RABAC1	10567	1.295	12	2	5	2	185	20.6	0.617609167
XP_005245231.2	microsomal glutathione S-transferase 3 isoform X1	MGST3	4259	1.233	16	1	3	1	152	16.5	0.900764297
XP_005270952.4	nuclear transcription factor Y subunit gamma isoform X2	NFYC	4802	1.175	4	2	3	2	544	59.9	0.765730504
NP_001153245.1	soluble calcium-activated nucleotidase 1	CANT1	124583	0.939	7	2	6	2	401	44.8	0.81355598
XP_005268894.1	amphoterin-induced protein 2 isoform X1	AMIGO2	347902	1.56	3	1	3	1	522	57.9	0.011192815
NP_056089.1	epididymis-specific alpha-mannosidase isoform 1 precursor	MAN2B2	23324	0.948	2	1	4	1	1009	113.9	0.939320787
NP_065715.1	peroxisomal 2,4-dienoyl-CoA reductase	DECR2	26063	1.198	7	2	5	2	292	30.8	0.754641215
XP_011542963.1	dedicator of cytokinesis protein 5 isoform X1	DOCK5	80005	0.644	2	3	4	3	1877	215.9	0.777255604
NP_060501.3	WD repeat-containing protein 6 isoform 1	WDR6	11180	0.934	3	2	2	2	1151	124.9	0.685210245
NP_976043.1	coiled-coil-helix-coiled-coil-helix domain-containing protein 1	CHCHD1	118487	0.816	25	2	6	2	118	13.5	0.1046326
XP_006711555.1	Golgi pH regulator A isoform X1	GPR89A	653519	0.877	6	1	2	1	455	52.9	0.571422098
NP_001018494.1	pumilio homolog 1 isoform 1	PUM1	9698	1.137	2	2	4	2	1188	126.6	0.834832383
NP_079239.3	F-box/LRR-repeat protein 18 isoform 2	FBXL18	80028	0.751	3	2	3	2	718	78.9	0.886947353
NP_776152.1	PDZ domain-containing protein 8	PDZD8	118987	1.29	3	3	8	3	1154	128.5	0.231222372
NP_001129514.2	PTGES3L-AARSD1 protein isoform 1	PTGES3L-AARSD1	100885850	0.885	5	2	3	2	586	65.7	0.678166892
XP_011513022.1	p21-activated protein kinase-interacting protein 1 isoform X1	PAK1IP1	55003	1.173	6	2	4	2	415	46.5	0.835472253
NP_002948.1	retinoic acid receptor RXR-alpha isoform a	RXRA	6256	1.003	7	2	4	1	462	50.8	0.817909943
NP_005006.3	mitochondrial inner membrane protein OXA1L	OXA1L	5018	1.524	4	2	7	2	495	55.2	0.006593871
NP_001511.2	general transcription factor 3C polypeptide 1 isoform 1	GTF3C1	2975	0.934	1	2	5	2	2109	238.7	0.972235176
XP_024306633.1	(E3-independent) E2 ubiquitin-conjugating enzyme isoform X1	UBE2O	63893	0.654	2	2	2	2	1329	145.1	0.966635442
XP_016856545.1	glutamate--cysteine ligase regulatory subunit isoform X1	GCLM	2730	1.025	11	2	5	2	275	30.4	0.920899673
XP_006713912.1	macrophage erythroblast attacher isoform X1	MAEA	10296	0.758	7	2	2	2	450	50.2	0.542159365
NP_060115.3	HAUS augmin-like complex subunit 6 isoform 1	HAUS6	54801	0.662	3	2	4	2	955	108.6	0.581687567
XP_005265227.2	macrophage-stimulating protein receptor isoform X1	MST1R	4486	1.51	1	2	7	1	1401	152.3	0.062141924
NP_005121.1	fibroblast growth factor-binding protein 1 precursor	FGFBP1	9982	0.525	6	1	3	1	234	26.2	0.00633635
NP_001273743.1	tumor necrosis factor alpha-induced protein 8 isoform c	TNFAIP8	25816	0.553	10	1	3	1	210	24.4	0.001347713
NP_076952.1	ADP-ribose pyrophosphatase, mitochondrial isoform a precursor	NUDT9	53343	0.858	9	3	4	3	350	39.1	0.657590925
NP_003334.2	ubiquitin-conjugating enzyme E2 G2 isoform 1	UBE2G2	7327	0.975	15	1	5	1	165	18.6	0.971023793
NP_005633.2	transcription initiation factor TFIID subunit 7	TAF7	6879	0.849	7	2	2	2	349	40.2	0.599313001
NP_005265.1	guanine nucleotide-binding protein G(I)/G(S)/G(O) subunit gamma-5 precursor	GNG5	2787	3.944	16	1	1	1	68	7.3	0.697445159
NP_060832.1	protein lin-7 homolog C	LIN7C	55327	1.245	13	3	9	3	197	21.8	0.027542131
NP_071344.1	39S ribosomal protein L17, mitochondrial	MRPL17	63875	1.63	13	3	10	3	175	20	0.105783814
NP_055486.2	ubiquitin-protein ligase E3C	UBE3C	9690	0.712	3	2	3	2	1083	123.8	0.90314323
NP_054722.2	pre-mRNA-splicing factor ATP-dependent RNA helicase PRP16	DHX38	9785	0.972	2	2	2	2	1227	140.4	0.983049612
NP_077719.2	neurogenic locus notch homolog protein 2 isoform 1 preproprotein	NOTCH2	4853	1.336	1	2	5	2	2471	265.2	0.268099486
NP_002638.2	phosphatidylinositol 3-kinase catalytic subunit type 3 isoform 1	PIK3C3	5289	0.756	3	2	5	2	887	101.5	0.654969003
XP_016873363.1	UBX domain-containing protein 1 isoform X2	UBXN1	51035	0.465	11	2	6	2	297	33.3	0.049678698
NP_005872.2	[3-methyl-2-oxobutanoate dehydrogenase [lipoamide]] kinase, mitochondrial isoform a precursor	BCKDK	10295	1.778	5	1	3	1	412	46.3	0.013203028
XP_006720438.1	calcium and integrin-binding protein 1 isoform X1	CIB1	10519	1.897	6	1	3	1	264	29.4	9.07E-05
NP_001018119.1	pirin	PIR	8544	0.333	4	1	4	1	290	32.1	0.044917561
NP_689968.1	protein canopy homolog 4 precursor	CNPY4	245812	1.335	14	2	2	2	248	28.3	0.734331977
NP_443109.2	mediator of RNA polymerase II transcription subunit 8 isoform 3	MED8	112950	0.874	5	1	2	1	301	32.8	0.96474573
XP_011541400.1	ribosome biogenesis protein NSA2 homolog isoform X1	NSA2	10412	1.523	7	2	4	2	271	31.2	0.373904098
NP_001243671.1	CCR4-NOT transcription complex subunit 10 isoform 3	CNOT10	25904	0.638	1	1	4	1	804	89.1	0.347396166
XP_016885057.1	PDZ domain-containing protein 11 isoform X1	PDZD11	51248	1.073	21	2	5	2	172	19.6	0.944218629
NP_078812.2	chondroitin sulfate synthase 2 isoform 1	CHPF	79586	0.893	4	2	3	2	775	85.4	0.969491446
NP_001306146.1	cytochrome P450 1A1 isoform 1	CYP1A1	1543	1.264	3	1	3	1	512	58.1	0.02209135
NP_001078816.1	prosalusin isoform a precursor	TOR2A	27433	1.01	4	1	9	1	321	35.7	0.612630587
XP_016867990.1	fidgetin-like protein 1 isoform X1	FIGNL1	63979	0.876	3	2	4	1	674	74	0.617641065
NP_057137.1	WASH complex subunit 3 isoform 1	WASHC3	51019	0.596	7	1	6	1	194	21.2	0.004030703
NP_001116827.1	serine/threonine-protein phosphatase 6 catalytic subunit isoform a	PPP6C	5537	0.768	9	2	4	2	342	38.9	0.784237139
NP_001284505.1	astrocytic phosphoprotein PEA-15 isoform a	PEA15	8682	0.552	14	2	4	2	151	17.3	0.370700895
XP_011509745.1	sphingomyelin phosphodiesterase 4 isoform X1	SMPD4	55627	0.792	3	1	4	1	944	105.7	0.74710195
XP_005251773.1	cathepsin L1 isoform X1	CTSL	1514	2.556	3	2	5	2	333	37.5	0.140178134
XP_005254344.1	transmembrane protein 87A isoform X1	TMEM87A	25963	1.105	3	2	5	2	556	63.5	0.585419421
NP_037469.2	mitochondrial import inner membrane translocase subunit Tim22	TIMM22	29928	0.987	8	1	3	1	194	20	0.908386348
NP_009121.1	SEC23-interacting protein	SEC23IP	11196	0.991	3	3	11	2	1000	111	0.791841081
NP_113642.1	selenoprotein O	SELENOO	83642	0.747	3	1	6	1	669	73.4	0.000967041
XP_011526907.1	protein LSM14 homolog B isoform X1	LSM14B	149986	1.048	2	1	3	1	486	53	0.911804242
NP_060842.3	ligand-dependent nuclear receptor-interacting factor 1 isoform 1	LRIF1	55791	0.958	3	2	4	2	769	84.5	0.837062667
NP_001129101.1	protein phosphatase 1 regulatory subunit 21 isoform 1	PPP1R21	129285	0.841	2	1	3	1	780	88.3	0.245630951
NP_002544.1	origin recognition complex subunit 5 isoform 1	ORC5	5001	0.678	2	1	6	1	435	50.3	0.250077875
NP_872282.1	ubiquinone biosynthesis monooxygenase COQ6, mitochondrial isoform a	COQ6	51004	1.142	4	2	7	2	468	50.8	0.128717188
NP_116759.2	caspase-10 isoform 1 preproprotein	CASP10	843	0.636	3	1	2	1	522	59	0.294694936
XP_006719842.1	E3 ubiquitin-protein ligase MYCBP2 isoform X1	MYCBP2	23077	0.934	1	2	6	2	4797	526.2	0.152054877
NP_057038.2	deoxyribose-phosphate aldolase isoform 1	DERA	51071	0.652	11	2	5	2	318	35.2	0.544283762
NP_004036.1	copper transport protein ATOX1	ATOX1	475	0.6	25	3	4	3	68	7.4	0.924368745
NP_001014432.1	RAC-alpha serine/threonine-protein kinase	AKT1	207	1.054	3	2	6	1	480	55.7	0.497570342
XP_005278336.1	CCR4-NOT transcription complex subunit 3 isoform X1	CNOT3	4849	0.922	7	2	4	2	771	83.8	0.900021695
XP_016884402.1	smoothelin isoform X1	SMTN	6525	0.689	2	2	5	2	1002	108	0.689015084
NP_072097.2	vacuolar protein sorting-associated protein 16 homolog isoform 1	VPS16	64601	0.791	3	2	5	2	839	94.6	0.523152866
NP_077309.1	fibronectin type III and SPRY domain-containing protein 1 isoform 1	FSD1	79187	0.588	8	2	3	2	496	55.8	0.101411899
NP_862820.1	origin recognition complex subunit 3 isoform 1	ORC3	23595	0.836	3	2	5	2	712	82.3	0.142381063
NP_005680.1	ATP-binding cassette sub-family B member 6, mitochondrial isoform 1	ABCB6	10058	0.958	2	1	3	1	842	93.8	0.981918997
NP_061929.2	NADH dehydrogenase	NDUFB11	54539	1.731	7	1	6	1	163	18.4	7.44E-05
NP_006388.2	ribonuclease H2 subunit A	RNASEH2A	10535	0.992	9	2	2	2	299	33.4	0.967520701
NP_001026883.1	mitochondrial calcium uniporter regulator 1	MCUR1	63933	1.143	6	2	3	2	359	39.7	0.130020908
NP_004116.2	DNAJC25-GNG10 protein precursor	DNAJC25-GNG10	552891	1.669	12	2	4	2	153	16.5	0.238208669
XP_016873816.1	glutamine and serine-rich protein 1 isoform X1	QSER1	79832	0.915	2	2	3	2	1888	205.5	0.789044244
NP_061982.3	chitobiosyldiphosphodolichol beta-mannosyltransferase isoform 1	ALG1	56052	1.331	5	2	5	2	464	52.5	0.338629814
NP_002805.1	26S proteasome non-ATPase regulatory subunit 10 isoform 1	PSMD10	5716	0.58	12	1	1	1	226	24.4	0.491491131
NP_001336644.1	enoyl-	MECR	51102	1.847	4	2	4	2	408	44.4	0.268193655
XP_016884904.1	G-patch domain and KOW motifs-containing protein isoform X1	GPKOW	27238	0.714	9	2	4	2	491	53.9	0.768239529
XP_006723900.1	protein NDRG3 isoform X3	NDRG3	57446	0.59	4	1	1	1	388	42.8	0.855005416
NP_064708.1	ubiquinone biosynthesis protein COQ9, mitochondrial precursor	COQ9	57017	1.142	12	2	3	2	318	35.5	0.825426455
XP_011543255.1	glucose 1,6-bisphosphate synthase isoform X1	PGM2L1	283209	0.844	4	2	4	2	643	72.8	0.136323933
NP_056170.2	polycomb protein SUZ12 isoform 1	SUZ12	23512	0.997	3	2	4	2	739	83	0.634210983
XP_006724817.1	E3 ubiquitin-protein ligase XIAP isoform X1	XIAP	331	0.454	3	1	5	1	497	56.6	0.180440237
NP_001269580.1	UDP-galactose translocator isoform h	SLC35A2	7355	1.335	3	1	5	1	421	44.1	0.327963794
NP_004194.3	membrane-associated tyrosine- and threonine-specific cdc2-inhibitory kinase isoform 1	PKMYT1	9088	0.742	3	1	4	1	499	54.5	0.826106156
NP_001305406.1	tsukushin isoform a precursor	TSKU	25987	1.245	4	1	3	1	367	39.3	0.01916215
NP_006218.1	phospholipid transfer protein isoform a precursor	PLTP	5360	0.841	3	1	3	1	493	54.7	0.152076006
NP_660308.1	coiled-coil domain-containing protein 127	CCDC127	133957	1.258	8	2	4	2	260	30.8	0.010218481
XP_016882257.2	NADH dehydrogenase	NDUFS7	374291	1.45	5	2	6	2	294	32	0.032852809
NP_001317240.1	segment polarity protein dishevelled homolog DVL-1 isoform 1	DVL1	1855	0.816	4	1	3	1	695	75.1	0.014613396
NP_001159711.1	cell growth regulator with EF hand domain protein 1 isoform a precursor	CGREF1	10669	3.012	11	1	3	1	318	33.4	2.86E-06
XP_005271435.1	transcriptional coactivator YAP1 isoform X1	YAP1	10413	0.976	5	2	4	2	510	55.1	0.396921817
NP_001272368.1	rapamycin-insensitive companion of mTOR isoform 2	RICTOR	253260	0.595	2	2	4	2	1732	194.9	0.835489245

Patil et al,2020. Proteomic and phosphoproteomic profiling of shammah-induced signaling in oral keratinocytes

Supplementary Table S1: List of proteins identified and quantified in OKF6/TERT1-Shammah and OKF6/TERT1-Parental total proteome using TMT-based quantitative proteomic approach

Accession	Protein Description	Gene Symbol	Gene ID	Fold Change	Coverage [%]	# Peptides	# PSMs	# Unique Peptides	# AAs	MW [kDa]	p-value
XP_016855760.1	lysophospholipase-like protein 1 isoform X1	LYPLAL1	127018	0.843	4	1	3	1	246	27.2	0.533119453
NP_114152.3	arf-GAP with GTPase, ANK repeat and PH domain-containing protein 3 isoform a	AGAP3	116988	0.412	2	1	4	1	911	97.9	0.001852955
XP_006713279.1	3-oxoacyl-	OXSM	54995	1.16	4	1	2	1	459	48.8	0.896816629
NP_001265874.1	lymphokine-activated killer T-cell-originated protein kinase isoform 2	PBK	55872	0.531	4	1	2	1	333	37.2	0.660842923
XP_024304258.1	glutamine amidotransferase-like class 1 domain-containing protein 1 isoform X1	GATD1	347862	0.624	4	1	4	1	229	24.2	0.228076977
XP_005262677.1	syndecan-1 isoform X1	SDC1	6382	0.704	11	2	6	2	334	34.6	0.022881506
NP_741999.3	iron-sulfur cluster co-chaperone protein HscB, mitochondrial isoform 1 precursor	HSCB	150274	1.07	8	2	4	2	235	27.4	0.864572542
NP_003932.3	neural Wiskott-Aldrich syndrome protein	WASL	8976	0.582	3	2	3	2	505	54.8	0.647918048
NP_758455.1	39S ribosomal protein L54, mitochondrial	MRPL54	116541	1.518	26	2	4	2	138	15.8	0.909599422
NP_002475.2	cytosolic Fe-S cluster assembly factor NUBP1 isoform 1	NUBP1	4682	0.659	8	2	4	2	320	34.5	0.57325264
XP_016884163.1	2-amino-3-ketobutyrate coenzyme A ligase, mitochondrial isoform X1	GCAT	23464	0.986	7	2	3	2	521	56.3	0.930793874
NP_001035787.1	ubiquitin-conjugating enzyme E2 variant 3 isoform a	UEVLD	55293	0.478	4	2	4	2	471	52.2	0.660576448
NP_060890.2	solute carrier family 22 member 15	SLC22A15	55356	1.426	2	1	2	1	547	60.5	0.673399257
XP_024309076.1	E3 ubiquitin-protein ligase ARIH2 isoform X1	ARIH2	10425	0.695	3	1	3	1	516	60.5	0.004381771
NP_003128.3	SRSF protein kinase 1	SRPK1	6732	0.846	4	3	7	3	655	74.3	0.746627662
NP_001026897.1	methylthioribose-1-phosphate isomerase isoform 1	MRI1	84245	0.383	4	1	3	1	369	39.1	9.34E-05
NP_055278.1	U6 snRNA-associated Sm-like protein LSm3	LSM3	27258	1.326	12	1	6	1	102	11.8	0.062035186
NP_004126.1	isocitrate dehydrogenase	IDH3G	3421	2.003	7	3	8	3	393	42.8	0.000180037
NP_004318.3	breakpoint cluster region protein isoform 1	BCR	613	0.416	3	2	4	2	1271	142.7	0.448722417
XP_024307071.1	AP-1 complex subunit mu-2 isoform X1	AP1M2	10053	0.706	9	3	5	3	503	56.6	0.569802905
XP_011537894.1	probable E3 ubiquitin-protein ligase HERC4 isoform X1	HERC4	26091	0.893	3	2	3	2	1081	121.1	0.744649271
NP_001121699.1	bifunctional UDP-N-acetylglucosamine 2-epimerase/N-acetylmannosamine kinase isoform 1	GNE	10020	0.508	2	2	4	2	753	83	0.392718335
NP_001269429.1	tetratricopeptide repeat protein 1	TTC1	7265	0.774	8	2	5	2	292	33.5	0.492312513
NP_003376.2	visinin-like protein 1	VSNL1	7447	0.843	14	2	2	2	191	22.1	0.257381385
XP_005247023.1	peptidyl-prolyl cis-trans isomerase G isoform X1	PPIG	9360	1.133	2	1	3	1	754	88.6	0.379250485
NP_003711.1	proteasome assembly chaperone 1 isoform a	PSMG1	8624	0.659	11	2	6	2	288	32.8	0.0002769
NP_001070666.1	autophagy-related protein 9A	ATG9A	79065	1.078	1	1	5	1	839	94.4	0.424338404
XP_016872373.1	acyl-CoA-binding domain-containing protein 5 isoform X1	ACBD5	91452	1.334	4	2	4	2	609	68.3	0.431151854
NP_005958.1	NGFI-A-binding protein 2 isoform 1	NAB2	4665	0.648	7	2	3	2	525	56.6	0.573656914
NP_037372.4	hemK methyltransferase family member 2 isoform 1	N6AMT1	29104	0.549	6	1	5	1	214	22.9	0.148537662
NP_005124.1	CAAX prenyl protease 2 isoform 1	RCE1	9986	0.766	4	1	1	1	329	35.8	0.874929916
NP_001002032.1	jupiter microtubule associated homolog 1 isoform 2	JPT1	51155	0.673	14	2	4	2	181	19.9	0.562026995
NP_079508.2	receptor expression-enhancing protein 4 isoform 1 precursor	REEP4	80346	1.233	8	2	4	2	257	29.4	0.683624129
NP_001617.1	RAC-beta serine/threonine-protein kinase isoform 1	AKT2	208	0.573	3	2	6	1	481	55.7	0.002557814
NP_006656.2	heparanase isoform 1 preproprotein	HPSE	10855	0.642	5	2	4	2	543	61.1	0.456203542
NP_001243114.1	sorting nexin-12 isoform 1	SNX12	29934	0.796	10	2	11	1	162	18.9	0.050188508
NP_005693.1	GTPase Era, mitochondrial isoform 1	ERAL1	26284	1.171	4	1	2	1	437	48.3	0.847151563
NP_872319.1	kinetochore protein Spc24 isoform 1	SPC24	147841	0.669	9	1	3	1	197	22.4	0.230538883
NP_068813.1	suppressor of tumorigenicity 14 protein	ST14	6768	1.177	2	1	3	1	855	94.7	0.067502921
NP_001284519.1	DNA-directed RNA polymerase I subunit RPA34 isoform 1	CD3EAP	10849	0.948	7	2	5	2	512	55.1	0.67382977
XP_024303020.1	maestro heat-like repeat-containing protein family member 1 isoform X1	MROH1	727957	0.602	1	1	3	1	1764	193.6	0.016560083
XP_016856939.1	ubiquitin-fold modifier-conjugating enzyme 1 isoform X1	UFC1	51506	0.764	11	2	3	2	167	19.4	0.830728954
XP_016871432.1	selenide, water dikinase 1 isoform X1	SEPHS1	22929	0.682	4	1	3	1	392	42.9	0.133890121
NP_001035546.1	rhomboid domain-containing protein 2 isoform a	RHBDD2	57414	1.003	4	1	3	1	364	39.2	0.873644874
NP_036465.2	C-Myc-binding protein	MYCBP	26292	1.158	19	2	3	2	103	12	0.958902829
NP_006306.2	polycomb group RING finger protein 3	PCGF3	10336	2.15	9	1	3	1	242	28.1	0.004365132
NP_006434.1	2'-deoxynucleoside 5'-phosphate N-hydrolase 1 isoform 1	DNPH1	10591	0.875	15	2	4	2	174	19.1	0.294182971
NP_001244289.1	AMP deaminase 2 isoform 1	AMPD2	271	0.561	3	3	7	3	879	100.6	0.097038564
NP_004985.2	matrix metalloproteinase-9 preproprotein	MMP9	4318	2.013	2	1	3	1	707	78.4	0.000539393
XP_011541108.1	interleukin-18 isoform X2	IL18	3606	0.407	9	2	5	2	193	22.3	0.08392701
NP_004555.1	glutamyl-tRNA(Gln) amidotransferase subunit B, mitochondrial isoform 1 precursor	GATB	5188	0.439	11	2	4	2	557	61.8	0.116224002
NP_114403.1	28S ribosomal protein S24, mitochondrial precursor	MRPS24	64951	1.089	10	2	2	2	167	19	0.879479716
NP_055953.3	RNA polymerase-associated protein RTF1 homolog	RTF1	23168	1.195	3	2	6	2	710	80.3	0.000365257
NP_006120.1	bone morphogenetic protein 1 isoform 3 precursor	BMP1	649	1.147	3	2	2	2	986	111.2	0.603966171
NP_001974.1	DNA excision repair protein ERCC-1 isoform 2	ERCC1	2067	0.653	11	2	4	2	297	32.5	0.551212947
NP_060915.2	selenoprotein S isoform 1	SELENOS	55829	1.63	15	2	3	2	189	21.2	0.054381808
NP_060129.2	glutaminyl-peptide cyclotransferase-like protein isoform 1	QPCTL	54814	1.58	5	2	5	2	382	42.9	0.495469901
XP_005245370.1	proline-rich protein PRCC isoform X1	PRCC	5546	0.536	8	1	3	1	523	56	0.018181712
NP_061845.2	ganglioside-induced differentiation-associated protein 1 isoform a	GDAP1	54332	1.457	7	2	5	2	358	41.3	0.159600088
NP_694574.3	NEDD4-binding protein 1	N4BP1	9683	0.634	3	2	2	2	896	100.3	0.896736345
NP_060372.2	hypoxia-inducible factor 1-alpha inhibitor	HIF1AN	55662	1.157	9	2	2	2	349	40.3	0.886859543
NP_065177.2	putative monooxygenase p33MONOX isoform a	KIAA1191	57179	0.972	13	2	4	2	305	33.2	0.821210902
NP_060104.2	BTB/POZ domain-containing protein KCTD9	KCTD9	54793	0.686	5	1	3	1	389	42.5	0.013767763
XP_011509738.1	pre-mRNA 3' end processing protein WDR33 isoform X1	WDR33	55339	1.353	1	2	3	2	1336	145.8	0.633709065
NP_775930.2	mitochondrial mRNA pseudouridine synthase RPUSD3 isoform 1	RPUSD3	285367	1.09	4	1	3	1	351	38.4	0.510580247
NP_001230695.1	protein O-linked-mannose beta-1,2-N-acetylglucosaminyltransferase 1 isoform 2	POMGNT1	55624	1.424	2	1	3	1	748	84.6	0.41382694
NP_001013725.2	eIF-2-alpha kinase GCN2	EIF2AK4	440275	0.702	2	2	4	2	1649	186.8	0.516237082
NP_115714.1	protein LLP homolog	LLPH	84298	1.215	11	1	1	1	129	15.2	0.716545166
NP_001127965.1	mitochondrial import receptor subunit TOM6 homolog	TOMM6	100188893	1.204	19	1	3	1	74	8	0.382951628
XP_016876625.1	alpha-(1,6)-fucosyltransferase isoform X1	FUT8	2530	1.17	3	2	5	2	609	70.3	0.263745981
NP_001121121.1	peroxisome assembly protein 26 isoform a	PEX26	55670	0.851	12	2	3	2	305	33.9	0.940944444
NP_006107.1	TGF-beta-activated kinase 1 and MAP3K7-binding protein 1 isoform alpha	TAB1	10454	0.571	5	2	4	2	504	54.6	0.002240452
XP_011514418.1	huntingtin-interacting protein 1 isoform X1	HIP1	3092	1.064	4	2	5	2	1037	116.1	0.94200278
NP_002496.1	nuclear transcription factor Y subunit alpha isoform 1	NFYA	4800	0.867	5	1	2	1	347	36.9	0.872313566
NP_066979.2	thioredoxin-related transmembrane protein 4 precursor	TMX4	56255	1.064	4	1	3	1	349	38.9	0.384003205
XP_011533426.1	M-phase phosphoprotein 8 isoform X1	MPHOSPH8	54737	1.094	2	1	3	1	879	98.9	0.579945663
NP_001026904.2	ferredoxin-2, mitochondrial precursor	FDX2	112812	0.792	17	2	3	2	186	19.9	0.759265675
NP_006836.2	kinesin-like protein KIF2C isoform 1	KIF2C	11004	0.636	4	2	4	2	725	81.3	0.719782837
XP_006714471.1	probable ATP-dependent RNA helicase DDX60-like isoform X1	DDX60L	91351	0.663	1	1	5	1	1707	197.6	0.4013851
NP_689518.1	protein O-glucosyltransferase 1 precursor	POGLUT1	56983	1.365	5	2	4	2	392	46.2	0.012927964
NP_001350105.1	transmembrane protein 68 isoform 1	TMEM68	137695	1.383	6	1	2	1	324	37.4	0.888013947
NP_001275655.1	elongator complex protein 4 isoform 3	ELP4	26610	0.554	3	1	1	1	535	58.8	0.191295779
XP_005256295.1	adhesion G-protein coupled receptor G1 isoform X1	ADGRG1	9289	2.027	2	1	2	1	698	78.1	0.707314465
NP_065120.2	ras-related protein Rab-25 precursor	RAB25	57111	1.338	9	2	6	1	213	23.5	0.002050664
XP_016883578.1	E3 ubiquitin-protein ligase Itchy homolog isoform X1	ITCH	83737	1.042	3	2	4	2	903	102.7	0.842632249
NP_001736.1	calcium signal-modulating cyclophilin ligand	CAMLG	819	0.551	7	1	3	1	296	32.9	0.081136073
XP_011544175.1	rho GTPase-activating protein 17 isoform X1	ARHGAP17	55114	1.532	2	1	1	1	913	98.9	0.645330398
XP_011513184.1	epithelial discoidin domain-containing receptor 1 isoform X1	DDR1	780	0.863	2	2	6	2	937	103.6	0.31429743
XP_024307260.1	interferon regulatory factor 3 isoform X1	IRF3	3661	0.697	6	2	5	2	480	51.9	0.685406431
NP_005819.3	DDB1- and CUL4-associated factor 7	DCAF7	10238	0.929	8	2	6	2	342	38.9	0.90780629
XP_005264262.1	EGF-containing fibulin-like extracellular matrix protein 1 isoform X1	EFEMP1	2202	1.639	6	2	5	2	543	59.3	0.766992046
NP_056342.3	TBC1 domain family member 10B	TBC1D10B	26000	1.044	4	2	4	2	808	87.1	0.993519708
XP_006715197.1	39S ribosomal protein S18a, mitochondrial isoform X1	MRPS18A	55168	1.681	7	2	6	2	270	30.5	0.063079617
XP_016869094.1	armadillo repeat-containing protein 1 isoform X1	ARMC1	55156	1.329	10	2	2	2	282	31.3	0.857759636
XP_006719985.1	dedicator of cytokinesis protein 9 isoform X5	DOCK9	23348	0.82	1	1	2	1	2141	244.5	0.863433188
XP_005258447.1	endothelial lipase isoform X1	LIPG	9388	1.639	3	1	3	1	536	60.5	0.002375178
NP_078881.3	rho GTPase-activating protein 10	ARHGAP10	79658	0.835	3	2	4	2	786	89.3	0.111856387
XP_011521469.1	chromosome transmission fidelity protein 8 homolog isoform X1	CHTF8	54921	0.986	5	2	3	2	524	51.4	0.908352491
XP_011517122.1	syntaxin-17 isoform X1	STX17	55014	1.5	8	2	3	2	302	33.4	0.576829497
NP_001268382.1	methyl-CpG-binding domain protein 3 isoform 1	MBD3	53615	0.826	5	1	3	1	291	32.8	0.015775157
XP_011518164.1	Hermansky-Pudlak syndrome 5 protein isoform X1	HPS5	11234	0.718	1	1	5	1	1156	130.1	0.584259526
NP_003306.3	dnaJ homolog subfamily C member 7 isoform 1	DNAJC7	7266	0.665	4	2	3	2	494	56.4	0.950598767
NP_067045.1	exopolyphosphatase PRUNE1 isoform 1	PRUNE1	58497	0.765	3	1	3	1	453	50.2	0.114498108
XP_005252033.1	protein prenyltransferase alpha subunit repeat-containing protein 1 isoform X1	PTAR1	375743	0.674	6	2	4	2	429	49.2	0.531016285
NP_000352.1	thrombomodulin precursor	THBD	7056	1.137	13	2	3	2	575	60.3	0.414788901
NP_065941.2	protein CIP2A	CIP2A	57650	0.732	2	2	2	2	905	102.1	0.73985059
XP_005250131.1	TRPM8 channel-associated factor 1 isoform X1	TCAF1	9747	0.771	3	2	6	2	921	102.1	0.005375301
NP_079413.3	spatacsin isoform 1	SPG11	80208	0.679	1	2	2	2	2443	278.7	0.70446856
XP_016879874.1	ADP-ribosylation factor-binding protein GGA3 isoform X1	GGA3	23163	0.736	4	3	4	2	733	79.2	0.981486362
NP_001005781.1	small ubiquitin-related modifier 1 isoform a precursor	SUMO1	7341	0.994	19	2	4	2	101	11.6	0.932661784
XP_005270469.1	target of EGR1 protein 1 isoform X1	TOE1	114034	1.016	4	2	3	2	516	57.2	0.949609794
XP_011535422.1	coiled-coil domain-containing protein 175 isoform X1	CCDC175	729665	0.804	1	1	9	1	794	93.7	0.13218496
XP_005260495.1	1-phosphatidylinositol 4,5-bisphosphate phosphodiesterase gamma-1 isoform X1	PLCG1	5335	0.988	2	2	5	2	1330	152.7	0.942239966
NP_036336.2	general transcription factor 3C polypeptide 4	GTF3C4	9329	1.342	2	1	3	1	822	91.9	0.157552104
XP_016858797.1	nucleoporin NUP53 isoform X1	NUP35	129401	1.343	9	2	2	2	329	35.2	0.863746473
XP_016856038.1	transmembrane protein 201 isoform X1	TMEM201	199953	1.586	3	2	2	2	765	82.9	0.961048683
NP_001138637.1	deoxyhypusine hydroxylase	DOHH	83475	0.95	7	1	2	1	302	32.9	0.936899173
NP_060755.1	U3 small nucleolar ribonucleoprotein protein IMP3	IMP3	55272	1.296	9	2	5	2	184	21.8	0.208905278
XP_024309954.1	kinesin-associated protein 3 isoform X1	KIFAP3	22920	0.438	3	2	2	1	807	92.6	0.897535278
NP_808800.1	Golgi membrane protein 1	GOLM1	51280	1.261	5	2	7	2	401	45.3	0.091250315
NP_071446.2	39S ribosomal protein L46, mitochondrial	MRPL46	26589	1.112	12	3	8	3	279	31.7	0.738300811
NP_064551.3	methylcrotonoyl-CoA carboxylase subunit alpha, mitochondrial isoform 1 precursor	MCCC1	56922	1.044	2	1	3	1	725	80.4	0.225698188
NP_945192.1	7,8-dihydro-8-oxoguanine triphosphatase isoform p22	NUDT1	4521	0.576	11	3	4	3	179	20.3	0.273581924
NP_940851.1	embigin precursor	EMB	133418	1.108	7	2	3	2	327	36.9	0.907326833
NP_115609.2	histone-lysine N-methyltransferase setd3 isoform a	SETD3	84193	0.574	2	1	1	1	594	67.2	0.207868678
XP_016856320.1	ubiquitin carboxyl-terminal hydrolase 24 isoform X1	USP24	23358	0.543	1	2	6	2	2640	296.3	0.255517204
XP_016870320.1	aprataxin isoform X1	APTX	54840	1.028	5	2	3	2	423	47.7	0.981013236
NP_004746.2	ribosomal protein S6 kinase alpha-5 isoform a	RPS6KA5	9252	0.926	2	2	4	2	802	89.8	0.839421565
XP_011537861.1	dynamin-binding protein isoform X1	DNMBP	23268	0.545	2	2	4	2	1577	177.2	0.387679988
NP_004926.1	cyclin-dependent-like kinase 5 isoform 1	CDK5	1020	0.29	5	2	10	1	292	33.3	0.000228357
NP_060650.2	putative pre-mRNA-splicing factor ATP-dependent RNA helicase DHX32	DHX32	55760	0.487	3	2	4	2	743	84.4	0.478509372
NP_036455.1	transcription factor MafF isoform a	MAFF	23764	1.707	14	2	5	2	164	17.7	1.50E-05
NP_003482.1	N-alpha-acetyltransferase 10 isoform 1	NAA10	8260	0.477	7	2	5	2	235	26.4	0.049752651
XP_005245271.1	deaminated glutathione amidase isoform X1	NIT1	4817	0.448	5	1	3	1	344	37.9	0.019872542
XP_006715037.1	histone-lysine N-methyltransferase EHMT2 isoform X1	EHMT2	10919	1.937	1	1	3	1	1274	139.5	0.995684905
NP_004658.3	E3 ubiquitin-protein ligase HERC2	HERC2	8924	0.939	1	2	3	2	4834	526.9	0.250813181
NP_003833.4	tumor necrosis factor receptor superfamily member 10B isoform 1 precursor	TNFRSF10B	8795	0.797	4	1	4	1	440	47.8	0.641839736
NP_008982.1	FAS-associated factor 1	FAF1	11124	0.563	3	2	5	2	650	73.9	0.014355961
NP_005853.2	cohesin subunit SA-1	STAG1	10274	0.898	2	2	2	1	1258	144.3	0.547225341
NP_001171564.1	branched-chain-amino-acid aminotransferase, cytosolic isoform 4	BCAT1	586	0.224	5	1	3	1	398	44.1	0.004151558
XP_024302154.1	NAD-dependent protein deacylase sirtuin-5, mitochondrial isoform X1	SIRT5	23408	1.549	9	2	6	2	310	33.9	0.000628589
NP_001004127.2	GDP-Man:Man(3)GlcNAc(2)-PP-Dol alpha-1,2-mannosyltransferase	ALG11	440138	1.508	4	2	5	2	492	55.6	0.840833308
NP_060950.3	complex I assembly factor TMEM126B, mitochondrial isoform a	TMEM126B	55863	0.622	8	1	3	1	230	25.9	0.002483708
NP_078942.3	alpha- and gamma-adaptin-binding protein p34 isoform 1	AAGAB	79719	0.639	4	1	2	1	315	34.6	0.505906402
NP_065801.1	exportin-5	XPO5	57510	0.444	2	1	2	1	1204	136.2	0.021797734
XP_016866864.1	reticulon-4-interacting protein 1, mitochondrial isoform X1	RTN4IP1	84816	1.73	3	1	3	1	401	44	0.001278326
XP_011519705.1	vam6/Vps39-like protein isoform X1	VPS39	23339	0.961	3	2	5	2	907	104.3	0.541604598
XP_024309306.1	cytoplasmic protein NCK1 isoform X1	NCK1	4690	0.637	5	2	5	2	377	42.8	0.124413916
XP_006721880.1	histone acetyltransferase KAT2A isoform X1	KAT2A	2648	0.973	2	2	2	1	838	94	0.576054869
NP_001034779.1	chromosome transmission fidelity protein 8 homolog	CHTF8	54921	0.69	11	1	2	1	121	13.3	0.604432858
NP_000811.1	growth arrest-specific protein 6 precursor	GAS6	2621	0.814	4	1	3	1	678	74.9	0.014011082
NP_054880.2	39S ribosomal protein L18, mitochondrial isoform 1	MRPL18	29074	1.467	12	2	5	2	180	20.6	0.142474178
NP_115553.2	phosphorylated adapter RNA export protein	PHAX	51808	0.466	7	2	3	2	394	44.4	0.494922362
XP_005261855.1	THO complex subunit 5 homolog isoform X1	THOC5	8563	1.358	2	2	5	2	694	79.7	0.424456734
NP_055363.1	tropomodulin-2 isoform a	TMOD2	29767	2.493	5	2	6	1	351	39.6	7.70E-05
NP_112496.1	haloacid dehalogenase-like hydrolase domain-containing protein 3	HDHD3	81932	1.357	10	2	4	2	251	28	0.135689754
XP_016858039.1	transcription termination factor 2 isoform X1	TTF2	8458	0.557	1	1	3	1	1190	132.5	0.005358794
NP_061846.2	dolichol-phosphate mannosyltransferase subunit 3 isoform 1	DPM3	54344	2.211	8	1	3	1	122	13.3	0.006427417
XP_016880525.1	tether containing UBX domain for GLUT4 isoform X1	ASPSCR1	79058	0.655	2	1	2	1	660	71.3	0.720825863
NP_004879.1	V-type proton ATPase subunit G 1	ATP6V1G1	9550	0.757	12	1	1	1	118	13.7	0.733897082
XP_016858490.1	glycosaminoglycan xylosylkinase isoform X1	FAM20B	9917	1.196	6	2	5	2	432	49.3	0.963117907
XP_011509194.1	SH3 domain-binding protein 4 isoform X1	SH3BP4	23677	0.699	3	2	4	2	963	107.4	0.889634168
NP_001929.1	protein Dr1	DR1	1810	0.783	7	1	3	1	176	19.4	0.100856968
NP_653337.1	protein arginine methyltransferase NDUFAF7, mitochondrial isoform 1	NDUFAF7	55471	1.128	4	1	1	1	441	49.2	0.342984386
NP_060481.3	arginine and glutamate-rich protein 1	ARGLU1	55082	2.124	6	2	6	2	273	33.2	0.006221012
NP_061901.2	palmitoyltransferase ZDHHC13 isoform 1	ZDHHC13	54503	1.24	2	1	4	1	622	70.8	0.476356084
NP_689608.2	U8 snoRNA-decapping enzyme isoform 2	NUDT16	131870	0.428	11	1	1	1	195	21.3	0.01326179
NP_079157.3	solute carrier family 35 member E1	SLC35E1	79939	1.245	3	1	2	1	410	44.7	0.79959247
NP_085151.1	histone-lysine N-methyltransferase SETD7 isoform 1	SETD7	80854	0.383	5	1	3	1	366	40.7	6.92E-06
NP_004220.2	mediator of RNA polymerase II transcription subunit 14	MED14	9282	0.958	2	2	3	2	1454	160.5	0.900658256
NP_113651.4	inactive hydroxysteroid dehydrogenase-like protein 1 isoform a	HSDL1	83693	1.427	5	2	5	2	330	37	0.400029679
NP_056943.1	transcription initiation factor IIA subunit 1 isoform 1	GTF2A1	2957	0.99	7	2	3	2	376	41.5	0.761694272
NP_001293009.1	LIM domain only protein 7 isoform 3	LMO7	4008	1.086	1	1	1	1	1631	186.1	0.735936253
NP_065881.1	WD repeat and FYVE domain-containing protein 1	WDFY1	57590	1.118	4	1	1	1	410	46.3	0.771331655
NP_003464.1	signal transducing adapter molecule 1 isoform a	STAM	8027	0.849	4	2	2	2	540	59.1	0.954447908
NP_005597.3	legumain isoform 1 preproprotein	LGMN	5641	0.91	4	1	1	1	433	49.4	0.607938433
NP_001177921.1	acetyl-coenzyme A transporter 1 isoform 1	SLC33A1	9197	0.534	2	1	3	1	549	60.9	0.03732039
NP_003174.3	disintegrin and metalloproteinase domain-containing protein 17 preproprotein	ADAM17	6868	0.609	3	2	2	2	824	93	0.954771099
NP_000480.3	transcriptional regulator ATRX isoform 1	ATRX	546	0.795	1	2	4	1	2492	282.4	0.075158793
NP_003086.1	small nuclear ribonucleoprotein F	SNRPF	6636	1.801	15	1	3	1	86	9.7	0.658253447
XP_011520726.1	DNA repair endonuclease XPF isoform X1	ERCC4	2072	0.762	2	1	5	1	962	109.6	0.290262177
NP_005362.3	tRNA (guanine-N(7)-)-methyltransferase isoform a	METTL1	4234	0.708	5	1	3	1	276	31.5	0.000214131
NP_060838.3	probable lysosomal cobalamin transporter	LMBRD1	55788	1.392	3	1	1	1	540	61.3	0.958909426
NP_001035207.1	arf-GAP with Rho-GAP domain, ANK repeat and PH domain-containing protein 1 isoform c	ARAP1	116985	0.59	1	1	2	1	1450	162.1	0.305605438
NP_005048.2	retinoblastoma-binding protein 5 isoform 1	RBBP5	5929	1.293	9	2	4	2	538	59.1	0.126925626
XP_016863041.1	TBC1 domain family member 5 isoform X1	TBC1D5	9779	0.796	2	2	3	2	817	91.2	0.510510927
XP_011524990.1	E3 ubiquitin-protein ligase CBL-C isoform X1	CBLC	23624	0.558	2	1	5	1	493	54.6	0.418366513
NP_001093096.1	mitochondrial fission regulator 1-like isoform a	MTFR1L	56181	1.194	10	2	4	2	292	31.9	0.776013764
NP_001345239.1	D-glutamate cyclase, mitochondrial isoform b precursor	DGLUCY	80017	1.702	4	2	4	2	621	66.9	0.652093352
NP_060205.3	tetratricopeptide repeat protein 27 isoform 1	TTC27	55622	0.648	2	1	1	1	843	96.6	0.638350891
NP_003161.2	transcription elongation factor SPT6	SUPT6H	6830	1.108	1	2	4	2	1726	198.9	0.753537948
XP_011534929.1	pleckstrin homology domain-containing family G member 3 isoform X4	PLEKHG3	26030	0.609	1	1	3	1	1391	152.9	0.00836606
NP_114172.1	G2/mitotic-specific cyclin-B1 isoform 1	CCNB1	891	0.605	4	1	1	1	433	48.3	0.642414864
NP_001350632.1	maleylacetoacetate isomerase isoform 4	GSTZ1	2954	0.936	9	1	5	1	217	24.2	0.85794697
XP_005247986.1	transforming acidic coiled-coil-containing protein 3 isoform X1	TACC3	10460	0.187	3	1	3	1	842	90.8	0.003450219
NP_001273685.1	anaphase-promoting complex subunit 4 isoform 1	ANAPC4	29945	0.696	2	1	2	1	809	92.2	0.011560151
XP_016865149.1	teneurin-2 isoform X1	TENM2	57451	1.542	1	1	3	1	2774	307.6	0.214085708
NP_001243459.1	translocator protein	TSPO	706	1.59	9	2	6	2	169	18.8	0.000720482
NP_443102.2	sorting nexin-18 isoform b	SNX18	112574	0.921	3	1	3	1	628	68.9	0.228594663
NP_001349806.1	SWI/SNF-related matrix-associated actin-dependent regulator of chromatin subfamily B member 1 isoform d	SMARCB1	6598	1.324	7	2	3	2	403	46	0.924230509
XP_016866442.1	chloride intracellular channel protein 5 isoform X1	CLIC5	53405	0.689	6	2	4	1	410	46.5	0.488647381
XP_016856867.1	nuclear valosin-containing protein-like isoform X1	NVL	4931	0.82	1	1	3	1	917	101.3	0.633696448
XP_024308698.1	intersectin-2 isoform X1	ITSN2	50618	0.925	1	1	2	1	1697	193.3	0.227682571
NP_004505.2	forkhead box protein K2	FOXK2	3607	1.117	3	2	2	1	660	69	0.876425196
XP_006723006.1	homer protein homolog 3 isoform X1	HOMER3	9454	1.208	3	1	2	1	361	39.8	0.975240171
NP_003722.2	Sjoegren syndrome nuclear autoantigen 1	SSNA1	8636	0.47	11	1	3	1	119	13.6	0.013643745
XP_011529987.1	methylmalonic aciduria type A protein, mitochondrial isoform X1	MMAA	166785	1.283	4	1	3	1	418	46.5	0.163985148
XP_011537286.1	protein SCAF11 isoform X4	SCAF11	9169	0.943	2	1	4	1	1479	166.5	0.862272262
NP_443183.1	deoxynucleotidyltransferase terminal-interacting protein 1	DNTTIP1	116092	1.274	5	1	3	1	329	37	0.007074044
NP_981951.1	metallo-beta-lactamase domain-containing protein 2	MBLAC2	153364	1.185	8	2	4	2	279	31.3	0.010268722
XP_005260519.1	ral GTPase-activating protein subunit beta isoform X1	RALGAPB	57148	0.812	2	2	3	2	1507	168.2	0.995234835
NP_001350300.1	spliceosome-associated protein CWC15 homolog	CWC15	51503	1.686	5	1	3	1	229	26.6	0.050432445
NP_061848.2	telomeric repeat-binding factor 2-interacting protein 1	TERF2IP	54386	1.19	5	1	2	1	399	44.2	0.889048868
NP_001338307.1	enhancer of mRNA-decapping protein 3 isoform 1	EDC3	80153	0.717	5	1	3	1	508	56	0.890219221
NP_060228.3	RNA demethylase ALKBH5	ALKBH5	54890	0.65	7	1	2	1	394	44.2	0.025333662
NP_001026847.1	ras-related protein Rab-24	RAB24	53917	1.428	8	1	3	1	203	23.1	0.187884092
NP_001316485.1	receptor expression-enhancing protein 6 isoform 1	REEP6	92840	0.564	6	1	3	1	211	23.4	0.033114523
XP_016864388.1	uncharacterized protein LOC100506514 isoform X1	LOC100506514	100506514	2.35	2	2	6	2	488	56.6	0.000100523
NP_055563.1	sorting nexin-17 isoform 1	SNX17	9784	0.574	4	1	1	1	470	52.9	0.345161856
NP_001273540.1	ralBP1-associated Eps domain-containing protein 1 isoform c	REPS1	85021	1.042	3	2	3	2	796	86.6	0.802463715
NP_001265319.1	sodium-coupled neutral amino acid transporter 1 isoform 2	SLC38A1	81539	1.1	3	2	4	2	503	56.2	0.405818563
NP_001349932.1	intraflagellar transport protein 27 homolog isoform 1	IFT27	11020	0.703	9	1	2	1	186	20.5	0.262178729
NP_009107.1	probable ergosterol biosynthetic protein 28	ERG28	11161	1.271	12	2	5	2	140	15.9	0.656925887
NP_113640.1	RNMT-activating mRNA cap methyltransferase subunit	RAMAC	83640	1.029	13	1	2	1	118	14.4	0.966298814
XP_024308643.1	inositol polyphosphate 1-phosphatase isoform X1	INPP1	3628	0.5	3	1	3	1	399	44	0.001896256
NP_003675.3	MAP kinase-interacting serine/threonine-protein kinase 1 isoform 1	MKNK1	8569	0.625	3	1	3	1	453	49.9	0.143496414
XP_016857969.1	breast cancer anti-estrogen resistance protein 3 isoform X1	BCAR3	8412	1.119	2	1	3	1	825	92.5	0.282619459
NP_005834.4	signal transducing adapter molecule 2	STAM2	10254	0.846	4	2	3	2	525	58.1	0.730002989
NP_443157.1	leukocyte receptor cluster member 8	LENG8	114823	1.142	4	2	4	2	800	88.1	0.587953202
NP_005185.2	CCAAT/enhancer-binding protein beta isoform a	CEBPB	1051	1.494	3	1	3	1	345	36.1	0.775772967
NP_054905.2	COMM domain-containing protein 9 isoform 1	COMMD9	29099	0.871	18	2	5	2	198	21.8	0.882800066
NP_002745.1	mitogen-activated protein kinase 13	MAPK13	5603	0.811	5	2	4	1	365	42.1	0.992424376
XP_005248348.1	ribonuclease 3 isoform X1	DROSHA	29102	0.38	1	1	6	1	1374	159.2	0.756859429
NP_078888.4	probable ATP-dependent RNA helicase DHX40 isoform 1	DHX40	79665	0.657	2	1	2	1	779	88.5	0.178094443
NP_003189.2	elongin-A	ELOA	6924	0.603	2	1	3	1	798	89.9	0.00648882
NP_003354.2	ubiquitin carboxyl-terminal hydrolase 4 isoform a	USP4	7375	0.478	2	1	3	1	963	108.5	0.002179334
NP_859067.2	centromere protein V	CENPV	201161	0.983	5	1	3	1	272	29.7	0.979277432
NP_006729.4	A-kinase anchor protein 13 isoform 1	AKAP13	11214	0.882	0	1	3	1	2817	307.7	0.808899972
XP_016862083.1	NCK-interacting protein with SH3 domain isoform X1	NCKIPSD	51517	0.675	2	1	1	1	762	82.9	0.941239935
NP_079470.1	inositol-trisphosphate 3-kinase C	ITPKC	80271	0.747	2	1	2	1	683	75.2	0.906729649
NP_000936.1	calcineurin subunit B type 1	PPP3R1	5534	0.962	23	2	2	2	170	19.3	0.663596141
NP_001124160.1	high mobility group protein B2	HMGB2	3148	0.673	9	3	4	2	209	24	0.790147298
XP_005251676.1	GPI ethanolamine phosphate transferase 3 isoform X1	PIGO	84720	0.886	3	2	5	2	1089	118.6	0.938618523
XP_016866842.1	F-box only protein 30 isoform X1	FBXO30	84085	0.803	2	1	3	1	745	82.3	0.172263872
XP_016856949.1	diphthine methyl ester synthase isoform X1	DPH5	51611	0.652	7	1	1	1	285	31.6	0.39751547
NP_055209.2	growth hormone-inducible transmembrane protein	GHITM	27069	1.721	6	2	6	2	345	37.2	0.001291424
XP_016860395.1	polypeptide N-acetylgalactosaminyltransferase 14 isoform X1	GALNT14	79623	0.811	4	2	3	2	563	65.1	0.325500722
XP_011543423.1	anoctamin-1 isoform X1	ANO1	55107	1.13	2	2	2	2	1008	116.5	0.625980197
XP_016858754.1	oxysterol-binding protein-related protein 6 isoform X1	OSBPL6	114880	0.687	1	1	2	1	981	111.6	0.438672798
NP_005913.2	mitogen-activated protein kinase kinase kinase 4 isoform a	MAP3K4	4216	0.373	1	1	3	1	1608	181.6	0.000261566
XP_016877890.1	dnaJ homolog subfamily C member 17 isoform X1	DNAJC17	55192	0.37	4	1	3	1	308	35	0.002773966
XP_006715051.1	serine/threonine-protein kinase 38 isoform X1	STK38	11329	1.024	6	2	3	2	465	54.2	0.987205671
XP_016866497.1	F-actin-uncapping protein LRRC16A isoform X1	CARMIL1	55604	0.837	2	2	4	2	1431	158.1	0.814755065
XP_011529475.1	myotubularin isoform X3	MTM1	4534	0.751	3	1	4	1	603	69.9	0.001882488
NP_001034859.2	transmembrane protein with metallophosphoesterase domain isoform 1	TMPPE	643853	1.265	2	1	3	1	453	49.4	0.332144811
XP_016871517.1	proline and serine-rich protein 2 isoform X1	PROSER2	254427	0.645	5	2	5	2	435	45.8	0.103075873
XP_005255528.1	chromosome transmission fidelity protein 18 homolog isoform X5	CHTF18	63922	0.821	1	1	1	1	1003	110.1	0.867350731
XP_011530693.1	type II inositol 3,4-bisphosphate 4-phosphatase isoform X1	INPP4B	8821	0.667	1	1	3	1	947	106.8	0.043745583
XP_006717090.1	choline transporter-like protein 1 isoform X1	SLC44A1	23446	1.157	2	1	2	1	737	82.9	0.935216608
NP_057342.2	YTH domain-containing family protein 2 isoform 1	YTHDF2	51441	0.78	2	1	2	1	579	62.3	0.70703045
XP_005246697.1	mitogen-activated protein kinase kinase kinase 20 isoform X1	MAP3K20	51776	0.71	1	1	3	1	800	91.1	0.005231867
NP_057272.1	actin-like protein 6B	ACTL6B	51412	0.636	3	2	3	1	426	46.8	0.800087059
NP_057562.3	nuclear-interacting partner of ALK isoform 1	ZC3HC1	51530	1.574	4	2	4	2	502	55.2	0.000461214
NP_071919.2	MAGUK p55 subfamily member 5 isoform 1	MPP5	64398	0.821	2	1	3	1	675	77.2	0.026459765
NP_060939.3	testis-expressed protein 2 isoform 1	TEX2	55852	0.759	1	1	3	1	1134	125.9	0.765048776
XP_011531236.1	HEAT repeat-containing protein 5B isoform X4	HEATR5B	54497	0.433	1	1	3	1	2071	224.2	0.076461361
NP_056173.1	MORC family CW-type zinc finger protein 3 isoform 1	MORC3	23515	0.756	1	1	3	1	939	107	0.33484554
XP_006718715.1	cell cycle checkpoint control protein RAD9A isoform X1	RAD9A	5883	0.766	5	1	3	1	402	43.9	0.208453647
NP_002068.1	golgin subfamily A member 1	GOLGA1	2800	0.705	2	1	2	1	767	88.1	0.633694677
NP_005917.2	microfibrillar-associated protein 1	MFAP1	4236	1.535	5	2	3	2	439	51.9	0.243186792

Patil et al,2020. Proteomic and phosphoproteomic profiling of shammah-induced signaling in oral keratinocytes

Supplementary Table S1: List of proteins identified and quantified in OKF6/TERT1-Shammah and OKF6/TERT1-Parental total proteome using TMT-based quantitative proteomic approach

Accession	Protein Description	Gene Symbol	Gene ID	Fold Change	Coverage [%]	# Peptides	# PSMs	# Unique Peptides	# AAs	MW [kDa]	p-value
NP_444278.1	39S ribosomal protein L53, mitochondrial	MRPL53	116540	1.609	9	1	3	1	112	12.1	0.053248819
XP_011530445.1	integrator complex subunit 12 isoform X1	INTS12	57117	1.739	3	1	3	1	462	48.8	0.004093632
NP_004595.2	syntaxin-4 isoform 3	STX4	6810	1.507	10	2	4	2	297	34.2	0.399639145
NP_000290.2	plakophilin-1 isoform 1b	PKP1	5317	1.437	2	1	2	1	747	82.8	0.75857342
XP_005265078.1	THUMP domain-containing protein 3 isoform X1	THUMPD3	25917	0.592	7	2	4	2	507	57	0.398169833
NP_001304978.1	epidermal retinol dehydrogenase 2 isoform 1	SDR16C5	195814	1.378	6	1	1	1	318	35.1	0.790772176
NP_002310.2	leucine-rich repeat and calponin homology domain-containing protein 4 isoform 1	LRCH4	4034	1.054	2	1	3	1	683	73.4	0.043753797
NP_004482.4	rho GTPase-activating protein 35	ARHGAP35	2909	0.412	2	2	5	2	1499	170.4	0.096438027
XP_005245324.2	cytosolic phospholipase A2 isoform X1	PLA2G4A	5321	1.209	2	1	2	1	754	85.6	0.9657565
XP_006724446.1	cadherin EGF LAG seven-pass G-type receptor 1 isoform X1	CELSR1	9620	0.995	1	1	5	1	3019	329.7	0.912258988
NP_059119.2	N-acetylgalactosaminyltransferase 7	GALNT7	51809	0.988	3	2	6	2	657	75.3	0.718197105
NP_001271267.1	E3 ubiquitin-protein ligase NEDD4 isoform 3	NEDD4	4734	0.455	1	1	3	1	1319	149	0.002985571
NP_002483.1	NADH dehydrogenase	NDUFB5	4711	1.373	12	3	6	3	189	21.7	0.923794392
NP_001073141.1	fibronectin type-III domain-containing protein 3A isoform 1	FNDC3A	22862	0.981	2	2	3	2	1198	131.8	0.917686615
NP_000391.1	general transcription and DNA repair factor IIH helicase subunit XPD isoform 1	ERCC2	2068	0.931	2	1	1	1	760	86.9	0.967639089
NP_001252524.1	prefoldin subunit 6	PFDN6	10471	0.548	16	2	4	2	129	14.6	0.678438529
NP_001177636.1	ALS2 C-terminal-like protein isoform 1	ALS2CL	259173	0.539	1	1	1	1	953	107.7	0.757989687
XP_011534401.1	dual specificity protein kinase TTK isoform X1	TTK	7272	0.803	2	1	1	1	857	97	0.725426869
NP_056018.2	regulation of nuclear pre-mRNA domain-containing protein 2 isoform 1	RPRD2	23248	1.071	1	1	3	1	1461	155.9	0.423631524
NP_001191819.1	ISY1-RAB43 protein	ISY1-RAB43	100534599	1.058	6	1	4	1	331	37.5	0.866993276
NP_001186125.1	armadillo repeat-containing protein 6 isoform 1	ARMC6	93436	0.696	4	1	1	1	501	54.1	0.907454223
XP_016874280.1	solute carrier family 15 member 4 isoform X1	SLC15A4	121260	0.765	2	1	3	1	635	67.4	0.186781268
XP_016872264.1	redox-regulatory protein FAM213A isoform X1	PRXL2A	84293	1.55	5	1	3	1	236	26.3	0.069735698
NP_115737.1	THO complex subunit 3	THOC3	84321	0.976	5	2	7	2	351	38.7	0.752254432
NP_001139400.1	histone deacetylase complex subunit SAP130 isoform a	SAP130	79595	1.551	2	2	3	2	1083	113.9	0.960376919
XP_011520489.1	protein regulator of cytokinesis 1 isoform X1	PRC1	9055	0.938	3	2	4	1	621	72	0.616150661
NP_003102.1	transcription factor Sp3 isoform 1	SP3	6670	1.458	2	1	1	1	781	81.9	0.67342273
XP_011536085.1	citron Rho-interacting kinase isoform X1	CIT	11113	0.818	1	1	3	1	2084	238.2	0.476045804
NP_001273120.1	protein CUSTOS isoform a	C12orf43	64897	0.75	7	1	3	1	293	31.7	0.091800243
XP_005254465.1	neogenin isoform X3	NEO1	4756	1.151	1	1	4	1	1481	161.9	0.580043815
NP_001137464.1	telomerase Cajal body protein 1	WRAP53	55135	0.786	6	2	4	2	548	59.3	0.551714156
NP_663304.1	mitogen-activated protein kinase kinase kinase 7 isoform B	MAP3K7	6885	0.619	3	1	1	1	606	67.2	0.127030395
XP_011515960.1	WASH complex subunit 1 isoform X1	WASHC1	100287171	0.629	3	1	2	1	479	51.7	0.52575857
XP_006713348.1	E3 ubiquitin-protein ligase RNF123 isoform X1	RNF123	63891	0.634	1	2	5	1	1314	148.4	0.049409395
XP_016868469.1	RNA-binding protein with multiple splicing isoform X1	RBPMS	11030	0.596	5	1	3	1	264	28.9	6.59E-06
NP_004868.1	glia maturation factor gamma isoform 1	GMFG	9535	0.841	6	1	3	1	142	16.8	0.303257275
NP_060686.3	pantothenate kinase 4	PANK4	55229	0.539	1	1	3	1	773	85.9	0.030203016
XP_011516670.1	fukutin isoform X1	FKTN	2218	1.196	3	1	2	1	488	56.4	0.617092469
NP_115689.1	nitric oxide-associated protein 1	NOA1	84273	0.702	2	1	4	1	698	78.4	0.225572561
XP_011511221.1	5'-3' exoribonuclease 1 isoform X1	XRN1	54464	1.578	1	2	3	2	1707	194.1	0.917130481
NP_066951.1	DNA-directed RNA polymerases I, II, and III subunit RPABC5	POLR2L	5441	0.989	13	1	4	1	67	7.6	0.213049412
NP_919299.3	beta-1,3-glucosyltransferase precursor	B3GLCT	145173	0.688	3	1	2	1	498	56.5	0.842508008
NP_001284.1	methylosome subunit pICln isoform a	CLNS1A	1207	0.868	5	1	3	1	237	26.2	0.91916032
NP_054829.2	nuclear inhibitor of protein phosphatase 1 isoform alpha	PPP1R8	5511	1.04	3	1	2	1	351	38.5	0.983761116
NP_005496.4	scavenger receptor class B member 1 isoform 1	SCARB1	949	0.941	6	3	4	3	509	56.9	0.825312121
NP_006756.2	tumor suppressor candidate 3 isoform a precursor	TUSC3	7991	1.339	3	1	3	1	348	39.7	0.028986171
NP_055925.2	structural maintenance of chromosomes protein 5	SMC5	23137	1.047	1	1	2	1	1101	128.7	0.842351152
XP_016869756.1	probable low affinity copper uptake protein 2 isoform X1	SLC31A2	1318	0.553	18	1	1	1	199	22.2	0.828920567
XP_016867663.1	inhibin beta A chain isoform X1	INHBA	3624	1.608	2	1	3	1	548	60.7	6.47E-05
NP_003623.1	contactin-associated protein 1 precursor	CNTNAP1	8506	0.681	2	2	6	2	1384	156.2	0.723808145
NP_004347.1	CD81 antigen isoform 1	CD81	975	0.639	7	1	3	1	236	25.8	0.002334054
NP_061910.1	exosome complex component RRP41	EXOSC4	54512	1.126	7	2	6	2	245	26.4	0.12736963
NP_001284487.1	replication protein A 32 kDa subunit isoform 3	RPA2	6118	0.578	5	1	3	1	278	30.1	0.062656551
NP_001036100.1	neuroguidin isoform 1	NGDN	25983	1.919	8	2	2	2	315	35.9	0.969446745
XP_005270851.1	immunoglobulin superfamily member 3 isoform X3	IGSF3	3321	1.376	1	1	3	1	1199	135.6	0.00833452
NP_005689.2	secretory carrier-associated membrane protein 3 isoform 1	SCAMP3	10067	1.36	9	2	2	2	347	38.3	0.824894421
NP_005783.2	M-phase phosphoprotein 6	MPHOSPH6	10200	0.839	14	2	2	2	160	19	0.973276845
NP_060671.3	TBC1 domain family member 13 isoform a	TBC1D13	54662	0.611	4	1	2	1	400	46.5	0.7495567
XP_011526999.1	targeting protein for Xklp2 isoform X1	TPX2	22974	1.23	2	1	1	1	747	85.6	0.966809475
XP_024303692.1	aurora kinase A-interacting protein isoform X2	AURKAIP1	54998	1.72	4	1	3	1	258	28.5	0.000951746
NP_002815.3	parathymosin isoform 2	PTMS	5763	0.757	23	2	5	2	102	11.5	0.690411441
NP_004536.2	NADH dehydrogenase	NDUFB1	4707	1.836	10	1	3	1	105	11.9	2.73E-05
NP_005472.2	mediator of RNA polymerase II transcription subunit 16	MED16	10025	1.482	2	2	5	2	877	96.7	0.028723633
XP_024308127.1	putative tRNA (cytidine(32)/guanosine(34)-2'-O)-methyltransferase isoform X1	FTSJ1	24140	0.747	5	1	2	1	329	36.1	0.71855524
NP_002848.1	peroxisomal biogenesis factor 19 isoform a	PEX19	5824	0.899	5	1	4	1	299	32.8	0.314777843
NP_060830.2	gamma-taxilin isoform 1	TXLNG	55787	0.923	4	2	5	2	528	60.5	0.786396938
NP_001193570.2	cytochrome c oxidase assembly factor 6 homolog isoform 2	COA6	388753	0.883	9	1	3	1	155	18	0.94546213
XP_011538453.1	UPF0668 protein C10orf76 isoform X1	ARMH3	79591	0.483	2	1	3	1	689	78.7	0.020414001
NP_116558.1	DNA-directed RNA polymerase II subunit RPB3	POLR2C	5432	0.655	5	1	3	1	275	31.4	0.055133308
XP_005248399.1	excitatory amino acid transporter 1 isoform X1	SLC1A3	6507	0.686	2	1	2	1	542	59.5	0.483555308
NP_006776.1	mucosa-associated lymphoid tissue lymphoma translocation protein 1 isoform a	MALT1	10892	0.298	1	1	2	1	824	92.2	0.225726179
NP_775889.1	INO80 complex subunit E isoform 1	INO80E	283899	0.641	9	1	1	1	244	26.5	0.141144548
NP_000240.1	DNA mismatch repair protein Mlh1 isoform 1	MLH1	4292	0.776	3	2	5	2	756	84.5	0.1126078
NP_569711.2	collagen alpha-1(XVIII) chain isoform 3 preproprotein	COL18A1	80781	1.42	2	2	4	2	1751	177.8	0.158986619
NP_003644.2	COP9 signalosome complex subunit 3 isoform 1	COPS3	8533	0.789	3	1	3	1	423	47.8	0.018639838
NP_115751.3	proline-rich AKT1 substrate 1 isoform a	AKT1S1	84335	0.799	5	1	2	1	276	29.2	0.783633215
NP_001129037.1	pseudouridine-5'-phosphatase isoform a	PUDP	8226	0.696	14	2	5	2	251	27.6	0.584604422
NP_037414.3	E3 ubiquitin-protein ligase UHRF1 isoform 2	UHRF1	29128	0.999	2	1	3	1	806	91	0.852841556
NP_783859.1	phosphatidylglycerophosphatase and protein-tyrosine phosphatase 1 isoform 1	PTPMT1	114971	1.86	6	1	3	1	201	22.8	0.021972447
NP_001127703.1	5'-nucleotidase domain-containing protein 2 isoform 1	NT5DC2	64943	1.526	4	2	2	2	557	64.1	0.847939168
NP_938018.1	glucose-6-phosphate exchanger SLC37A2 isoform 1	SLC37A2	219855	1.257	2	1	3	1	505	54.8	0.354363406
NP_115736.1	acyl-CoA-binding domain-containing protein 6	ACBD6	84320	0.563	4	1	3	1	282	31.1	0.011048746
NP_001104490.1	transcription elongation factor SPT5 isoform a	SUPT5H	6829	1.155	5	2	2	2	1087	120.9	0.896533462
NP_150648.2	vacuolar protein sorting-associated protein 13A isoform A	VPS13A	23230	1.307	0	1	3	1	3174	360	0.233598735
XP_011544439.1	F-actin-monooxygenase MICAL3 isoform X1	MICAL3	57553	0.497	1	1	1	1	2209	247.5	0.592461649
NP_000776.1	25-hydroxyvitamin D-1 alpha hydroxylase, mitochondrial	CYP27B1	1594	0.805	3	1	3	1	508	56.5	0.647697051
NP_001278.1	H(+)/Cl(-) exchange transporter 7 isoform a	CLCN7	1186	1.263	2	2	5	2	805	88.6	0.541228068
NP_036273.1	integrator complex subunit 6 isoform a	INTS6	26512	1.005	2	1	2	1	887	100.3	0.439312381
NP_060616.1	rRNA methyltransferase 3, mitochondrial isoform 1 precursor	MRM3	55178	1.201	9	2	6	2	420	47	0.210259355
XP_011507955.1	POU domain, class 2, transcription factor 1 isoform X1	POU2F1	5451	1.105	2	2	4	2	769	78.9	0.869797711
NP_068597.2	coiled-coil domain-containing protein 90B, mitochondrial isoform 1 precursor	CCDC90B	60492	1.661	7	1	2	1	254	29.5	0.988158753
NP_004137.2	NADH dehydrogenase	NDUFB7	4713	1.288	7	1	4	1	137	16.4	0.715581092
XP_016865929.1	lysine-specific histone demethylase 1B isoform X1	KDM1B	221656	1.268	1	1	3	1	832	93.2	0.121947638
XP_006712192.1	sodium-dependent multivitamin transporter isoform X1	SLC5A6	8884	0.633	2	1	3	1	635	68.6	0.343982104
NP_076956.1	gamma-glutamylcyclotransferase isoform 1	GGCT	79017	0.432	11	2	4	2	188	21	0.064211782
NP_006393.2	regulator complex protein LAMTOR5	LAMTOR5	10542	0.425	12	1	2	1	173	18.1	0.395897938
XP_006719627.1	TBC1 domain family member 15 isoform X1	TBC1D15	64786	0.953	3	2	3	2	699	80.6	0.798314196
NP_062564.1	interleukin-36 gamma isoform 1	IL36G	56300	0.729	11	1	1	1	169	18.7	0.193033915
XP_011524671.1	kallikrein-11 isoform X1	KLK11	11012	0.83	2	1	8	1	307	33.7	0.36915174
NP_001274532.1	zinc finger and BTB domain-containing protein 17 isoform 3	ZBTB17	7709	1.563	2	1	3	1	810	88.5	0.020933181
XP_016875753.1	C2 domain-containing protein 5 isoform X1	C2CD5	9847	0.693	2	1	2	1	1161	127.9	0.543128243
NP_733746.1	sialate O-acetylesterase isoform 1 precursor	SIAE	54414	0.605	3	1	3	1	523	58.3	0.079499056
NP_690856.1	E3 ubiquitin-protein ligase UHRF2	UHRF2	115426	1.015	3	2	4	2	802	89.9	0.89492639
XP_016864434.1	TNFAIP3-interacting protein 1 isoform X1	TNIP1	10318	0.747	2	1	2	1	698	78.4	0.521115676
NP_001243055.1	ceramide synthase 6 isoform 1	CERS6	253782	1.267	3	2	4	2	392	45.8	0.615127527
XP_016866759.1	protein tyrosine phosphatase type IVA 1 isoform X1	PTP4A1	7803	0.463	9	1	3	1	173	19.8	0.004645912
NP_001014832.1	serine/threonine-protein kinase PAK 4 isoform 1	PAK4	10298	0.9	4	2	2	2	591	64	0.886766643
XP_011513480.1	BRCA1-associated ATM activator 1 isoform X1	BRAT1	221927	0.501	2	1	3	1	909	97.4	0.017309147
XP_006722125.1	formin-like protein 1 isoform X1	FMNL1	752	0.734	1	1	1	1	1164	128.9	0.160306089
XP_016857247.1	REST corepressor 3 isoform X1	RCOR3	55758	0.687	3	1	3	1	586	64.5	0.00149199
XP_005271230.1	low-density lipoprotein receptor-related protein 8 isoform X1	LRP8	7804	1.281	3	2	2	2	976	107.1	0.793671574
NP_612450.2	cyclic GMP-AMP synthase	CGAS	115004	1.485	2	1	3	1	522	58.8	0.020138627
NP_004609.1	DNA topoisomerase 3-alpha isoform 1	TOP3A	7156	0.919	1	1	3	1	1001	112.3	0.973535606
NP_057256.2	ubiquitin-associated domain-containing protein 1	UBAC1	10422	0.44	9	2	4	2	405	45.3	0.081292058
NP_036525.1	phosphoribosylformylglycinamidine synthase	PFAS	5198	0.708	2	2	5	2	1338	144.6	0.706653764
NP_490647.1	dnaJ homolog subfamily B member 6 isoform a	DNAJB6	10049	0.777	5	1	3	1	326	36.1	0.07027059
NP_001337459.1	mesoderm induction early response protein 1 isoform k	MIER1	57708	0.899	6	2	2	2	602	67.6	0.810410965
XP_016859651.1	translation initiation factor IF-2, mitochondrial isoform X1	MTIF2	4528	1.295	3	2	4	2	727	81.3	0.78518325
NP_112211.1	protein phosphatase 1 regulatory subunit 14C	PPP1R14C	81706	0.431	10	1	3	1	165	17.8	0.000778621
NP_689729.1	COMM domain-containing protein 1 isoform 1	COMMD1	150684	0.777	9	1	3	1	190	21.2	0.126679559
NP_001306593.1	transmembrane protein 263 isoform a	TMEM263	90488	1.124	13	1	1	1	116	11.7	0.894058064
NP_056156.2	condensin complex subunit 2 isoform 1	NCAPH	23397	0.727	5	2	4	2	741	82.5	0.000302968
XP_016857596.1	nicotinamide/nicotinic acid mononucleotide adenylyltransferase 1 isoform X1	NMNAT1	64802	0.949	6	1	3	1	279	31.9	0.728251171
XP_016884791.1	uncharacterized protein CXorf38 isoform X2	CXorf38	159013	0.803	4	1	1	1	330	38.2	0.859817056
NP_001035982.1	protein diaphanous homolog 3 isoform a	DIAPH3	81624	0.865	2	2	2	2	1193	136.8	0.637943778
NP_002102.4	huntingtin	HTT	3064	0.59	1	1	2	1	3144	347.6	0.38331006
NP_733936.1	succinate-semialdehyde dehydrogenase, mitochondrial isoform 1 precursor	ALDH5A1	7915	1.238	5	2	2	2	548	58.6	0.937842396
NP_002827.1	receptor-type tyrosine-protein phosphatase alpha isoform 1 precursor	PTPRA	5786	2.403	1	1	3	1	802	90.7	0.017480542
NP_665860.2	ectodysplasin-A receptor-associated adapter protein isoform A	EDARADD	128178	0.741	8	1	2	1	215	24.8	0.913118259
XP_016862457.1	ras GTPase-activating protein 2 isoform X1	RASA2	5922	0.576	2	1	3	1	872	100	0.050923586
NP_064710.5	UPF0505 protein C16orf62 isoform 1	VPS35L	57020	0.536	1	1	1	1	963	109.5	0.418430852
NP_003575.2	RNA/RNP complex-1-interacting phosphatase	DUSP11	8446	0.723	4	1	3	1	377	43.7	0.025519596
XP_005253240.1	tripartite motif-containing protein 5 isoform X1	TRIM5	85363	0.542	3	1	2	1	493	56.3	0.116495056
NP_998760.1	iron-sulfur cluster assembly enzyme ISCU, mitochondrial isoform 2 precursor	ISCU	23479	1.511	8	2	4	2	167	18	0.121371013
NP_001104262.1	methyl-CpG-binding protein 2 isoform 2	MECP2	4204	1.498	4	2	4	2	498	53.3	0.890397246
XP_016860185.1	HCLS1-binding protein 3 isoform X1	HS1BP3	64342	0.905	5	1	2	1	396	43.3	0.367340565
NP_057017.1	translation machinery-associated protein 7 isoform 1	TMA7	51372	0.53	14	1	3	1	64	7.1	0.001031408
XP_016866981.1	tight junction-associated protein 1 isoform X2	TJAP1	93643	0.939	3	1	3	1	547	60.7	0.672307412
NP_115662.2	mediator of RNA polymerase II transcription subunit 10	MED10	84246	0.972	10	1	2	1	135	15.7	0.483056572
NP_060835.1	meiosis-specific nuclear structural protein 1	MNS1	55329	0.823	3	2	5	2	495	60.5	0.786158527
XP_005273138.2	consortin isoform X1	CNST	163882	1.173	2	1	1	1	725	79.5	0.094060906
XP_016857855.1	dyslexia-associated protein KIAA0319-like protein isoform X1	KIAA0319L	79932	1.52	1	1	2	1	1054	116.3	0.879088411
NP_064695.3	aldo-keto reductase family 1 member B10	AKR1B10	57016	1.451	3	1	3	1	316	36	0.115290775
NP_057616.2	inositol polyphosphate 5-phosphatase K isoform 1	INPP5K	51763	0.726	2	1	3	1	448	51.1	0.239604103
NP_003394.1	transcriptional repressor protein YY1	YY1	7528	0.911	6	2	2	2	414	44.7	0.824684272
XP_006721660.1	mannose-P-dolichol utilization defect 1 protein isoform X1	MPDU1	9526	0.735	4	1	4	1	260	28.2	0.09462237
XP_005265391.1	monoacylglycerol lipase ABHD6 isoform X1	ABHD6	57406	1.684	4	1	3	1	338	38.4	0.174526785
NP_054903.1	protein cornichon homolog 4 isoform 1	CNIH4	29097	1.023	14	1	3	1	139	16.1	0.283346343
XP_024304171.1	DNA methyltransferase 1-associated protein 1 isoform X1	DMAP1	55929	1.208	2	1	3	1	467	53	0.509510161
NP_060379.2	required for meiotic nuclear division protein 1 homolog isoform 1	RMND1	55005	1.496	3	1	1	1	449	51.6	0.805961193
NP_942600.1	acidic fibroblast growth factor intracellular-binding protein isoform a	FIBP	9158	1.046	4	1	3	1	364	41.9	0.876920588
NP_055101.2	neuronal calcium sensor 1 isoform 1	NCS1	23413	0.595	8	1	3	1	190	21.9	0.079526248
XP_016856233.1	pogo transposable element with ZNF domain isoform X1	POGZ	23126	1.209	1	2	4	2	1417	156	0.619253741
XP_011530916.1	regulator of microtubule dynamics protein 2 isoform X1	RMDN2	151393	0.85	2	1	2	1	616	69.8	0.580694197
XP_005253650.1	CAP-Gly domain-containing linker protein 1 isoform X1	CLIP1	6249	0.67	1	2	2	2	1440	162.4	0.915057486
XP_005267921.1	presenilin-1 isoform X1	PSEN1	5663	0.903	4	1	2	1	467	52.6	0.993644425
XP_011529014.2	traB domain-containing protein isoform X1	TRABD	80305	1.155	6	2	6	2	383	43.1	0.004638383
NP_002241.1	intermediate conductance calcium-activated potassium channel protein 4	KCNN4	3783	0.695	4	1	3	1	427	47.7	0.079124478
NP_061870.1	28S ribosomal protein S21, mitochondrial	MRPS21	54460	1.261	14	1	3	1	87	10.7	0.896701863
NP_066990.3	GATA zinc finger domain-containing protein 1	GATAD1	57798	0.752	6	1	3	1	269	28.7	0.008672667
XP_016867003.1	pre-mRNA-splicing regulator WTAP isoform X1	WTAP	9589	1.146	3	1	1	1	413	46.1	0.962101141
NP_037451.1	ubiA prenyltransferase domain-containing protein 1 isoform 1	UBIAD1	29914	1.685	3	1	2	1	338	36.8	0.582102629
XP_011511782.1	syntaxin-18 isoform X1	STX18	53407	0.97	8	2	3	2	366	41.8	0.910113641
NP_004738.3	disks large homolog 5	DLG5	9231	0.82	1	1	3	1	1919	213.7	0.446406802
XP_011510350.1	protein TANC1 isoform X2	TANC1	85461	0.388	1	1	3	1	1885	204.7	0.020498274
NP_005397.1	rho-associated protein kinase 1	ROCK1	6093	0.638	1	1	3	1	1354	158.1	0.081680197
NP_001119601.1	DNA replication complex GINS protein PSF3 isoform a	GINS3	64785	1.641	5	1	1	1	255	28.8	0.835502713
NP_060873.4	mRNA-decapping enzyme 1A isoform a	DCP1A	55802	0.644	2	1	1	1	582	63.2	0.73860168
NP_003185.1	TATA-box-binding protein isoform 1	TBP	6908	0.88	4	1	2	1	339	37.7	0.224221957
NP_068375.3	vacuolar protein sorting-associated protein 11 homolog isoform 1	VPS11	55823	0.818	1	1	1	1	941	107.7	0.648437822
XP_005250875.1	dihydropyrimidinase isoform X1	DPYS	1807	1.237	3	2	4	1	555	60.6	0.997717046
NP_060137.2	syndetin isoform a	VPS50	55610	0.688	3	2	2	2	964	111.1	0.732538587
NP_510966.1	CD97 antigen isoform 1 preproprotein	ADGRE5	976	0.616	2	1	3	1	835	91.8	0.002417147
NP_001106849.1	Fanconi anemia group I protein isoform 1	FANCI	55215	0.829	1	1	1	1	1328	149.2	0.377961716
NP_001180442.1	zinc transporter 6 isoform 1	SLC30A6	55676	0.639	3	1	3	1	501	55.8	0.113564835
XP_016859126.1	cysteine protease ATG4B isoform X2	ATG4B	23192	0.736	3	1	2	1	479	53.2	0.532700226
NP_000118.2	exostosin-1	EXT1	2131	1.071	2	1	2	1	746	86.2	0.594400302
XP_024307057.1	thioredoxin-like protein 1 isoform X1	TXNL1	9352	0.692	8	2	4	2	292	32.7	0.03254479
XP_005263279.1	glutathione S-transferase C-terminal domain-containing protein isoform X1	GSTCD	79807	0.473	2	1	3	1	633	71	0.012676494
NP_005345.3	transcription factor jun-D isoform JunD-FL	JUND	3727	1.299	4	2	5	1	347	35.2	0.977015254
NP_001273166.1	solute carrier family 2, facilitated glucose transporter member 14 isoform d	SLC2A14	144195	2.429	2	1	3	1	535	58.2	0.001872276
XP_016866284.1	dermatan-sulfate epimerase isoform X1	DSE	29940	1.318	2	2	4	2	977	111.8	0.466333225
NP_060311.1	succinate dehydrogenase assembly factor 2, mitochondrial	SDHAF2	54949	1.065	7	1	3	1	166	19.6	0.714053067
XP_024304548.1	CD151 antigen isoform X1	CD151	977	1.718	7	2	6	2	253	28.3	0.084191311
NP_001094284.1	type 1 phosphatidylinositol 4,5-bisphosphate 4-phosphatase isoform 1	PIP4P1	90809	1.601	4	1	2	1	284	30.2	0.541599967
XP_005248178.1	SLAIN motif-containing protein 2 isoform X1	SLAIN2	57606	0.75	4	1	2	1	607	65.5	0.66005832
NP_612206.5	protein FAM122A	FAM122A	116224	1.055	5	1	1	1	287	30.5	0.734412482
NP_001273725.1	N-terminal Xaa-Pro-Lys N-methyltransferase 1 isoform a	NTMT1	28989	0.783	13	2	2	2	223	25.4	0.88492984
XP_024302721.1	transmembrane protein 120A isoform X1	TMEM120A	83862	2.68	2	1	3	1	410	47.1	0.00022656
XP_006715470.1	nesprin-1 isoform X1	SYNE1	23345	0.565	0	1	2	1	8846	1015.9	0.354182659
XP_016870293.1	putative methyltransferase C9orf114 isoform X1	SPOUT1	51490	1.27	5	1	3	1	458	50.6	0.06709239
XP_016879383.1	Na(+)/H(+) exchange regulatory cofactor NHE-RF2 isoform X1	SLC9A3R2	9351	1.993	4	1	2	1	372	38.6	0.710971879
NP_620129.2	R3H domain-containing protein 4	R3HDM4	91300	0.814	6	1	3	1	268	30.3	0.039366031
NP_009041.2	telomerase protein component 1 isoform 1	TEP1	7011	0.582	0	1	3	1	2627	290.3	0.00355243
NP_060479.3	tapasin-related protein precursor	TAPBPL	55080	1.126	3	1	3	1	468	50.2	0.207016878
NP_003622.2	poly(ADP-ribose) glycohydrolase isoform a	PARG	8505	0.613	2	1	3	1	976	111	0.00488997
NP_001268364.1	dual specificity mitogen-activated protein kinase kinase 4 isoform 2	MAP2K4	6416	0.377	4	1	2	1	410	45.6	0.457004966
XP_016881898.1	zinc finger protein 579 isoform X1	ZNF579	163033	1.094	3	1	2	1	640	68.4	0.876024025
NP_733752.1	max-like protein X isoform gamma	MLX	6945	0.898	3	1	1	1	298	33.3	0.378921926
NP_055445.2	zinc finger protein 592	ZNF592	9640	1.127	2	1	4	1	1267	137.4	0.873591776
XP_011541371.1	etoposide-induced protein 2.4 homolog isoform X1	EI24	9538	1.368	3	1	3	1	340	38.9	0.871367604
XP_024308659.1	microtubule-associated protein 2 isoform X1	MAP2	4133	0.599	1	1	1	1	1915	208.7	0.356363384
NP_112202.2	iron-sulfur cluster assembly 1 homolog, mitochondrial precursor	ISCA1	81689	1.28	9	1	2	1	129	14.2	0.688997121
NP_116246.2	serine beta-lactamase-like protein LACTB, mitochondrial isoform a precursor	LACTB	114294	1.76	2	1	3	1	547	60.7	0.001958018
NP_077306.3	long-chain fatty acid transport protein 3 isoform 1 precursor	SLC27A3	11000	0.923	3	1	3	1	683	73.5	0.809078732
NP_891552.1	HEAT repeat-containing protein 3 isoform a	HEATR3	55027	0.658	4	2	3	2	680	74.5	0.369563963
XP_016860402.1	structural maintenance of chromosomes protein 6 isoform X1	SMC6	79677	1.003	4	2	3	2	1110	128.7	0.965436943
NP_000278.3	peroxisome biogenesis factor 6 isoform 1	PEX6	5190	0.981	2	1	3	1	980	104	0.986266853
NP_065095.2	PITH domain-containing protein 1	PITHD1	57095	0.569	10	1	1	1	211	24.2	0.435625776
XP_005248058.1	histone-lysine N-methyltransferase NSD2 isoform X1	NSD2	7468	0.679	1	1	1	1	1365	152.2	0.664546165
NP_997281.2	guanylate-binding protein 7	GBP7	388646	0.815	3	2	4	1	638	72.5	0.990764873
XP_016856050.1	alpha-taxilin isoform X1	TXLNA	200081	0.67	3	2	4	2	579	65.5	0.906140127
XP_016861001.1	nucleoside diphosphate kinase 6 isoform X6	NME6	10201	1.028	5	1	3	1	264	29	0.374238151
NP_001136202.1	BTB/POZ domain-containing protein KCTD1 isoform b	KCTD1	284252	0.789	2	1	1	1	865	96.4	0.441307372
XP_011509661.1	coiled-coil domain-containing protein 93 isoform X1	CCDC93	54520	0.871	2	2	4	2	686	78.3	0.938942792
NP_001121633.1	upstream-binding protein 1 isoform LBP-1b	UBP1	7342	0.704	3	1	2	1	540	60.5	0.034836006
XP_024302623.1	RAD50-interacting protein 1 isoform X1	RINT1	60561	0.849	3	1	1	1	816	93.4	0.460225922
XP_011536701.1	CCR4-NOT transcription complex subunit 2 isoform X1	CNOT2	4848	0.875	3	1	1	1	540	59.7	0.899887996
XP_011521623.1	rho family-interacting cell polarization regulator 1 isoform X2	RIPOR1	79567	0.636	2	1	3	1	1248	135.1	0.123727921
NP_054727.1	coiled-coil domain-containing protein 22	CCDC22	28952	0.723	2	1	2	1	627	70.7	0.62607682
XP_005257700.1	vascular endothelial zinc finger 1 isoform X1	VEZF1	7716	1.093	2	1	3	1	527	57.6	0.893765661
NP_001035088.2	protein FAM83G	FAM83G	644815	0.835	3	1	1	1	823	90.8	0.893841807
NP_550438.1	deoxyguanosine kinase, mitochondrial isoform a precursor	DGUOK	1716	2.102	3	1	2	1	277	32	0.283958626
NP_001184058.1	protein SYS1 homolog isoform a	SYS1	90196	1.422	6	1	3	1	156	17.6	0.001428185
NP_071447.1	tubulointerstitial nephritis antigen-like isoform 1 precursor	TINAGL1	64129	2.948	2	1	3	1	467	52.4	2.94E-05
NP_002687.1	DNA-directed RNA polymerase II subunit RPB7	POLR2G	5436	0.476	11	2	3	2	172	19.3	0.004890215
XP_016858494.1	kinesin-like protein KIF14 isoform X1	KIF14	9928	1.114	1	1	4	1	1648	186.4	0.967965459
NP_001129465.1	tetratricopeptide repeat protein 39C isoform 1	TTC39C	125488	0.49	2	1	2	1	583	65.8	0.395139399
XP_016870772.1	E3 ubiquitin-protein ligase LRSAM1 isoform X1	LRSAM1	90678	0.434	4	1	3	1	723	83.5	0.006017044
XP_011509557.1	origin recognition complex subunit 4 isoform X1	ORC4	5000	0.207	2	2	3	2	436	50.3	0.143682292
NP_001010917.1	golgin subfamily A member 7B	GOLGA7B	401647	1.416	5	1	2	1	167	18.3	0.968409825
NP_115984.3	neurabin-2	PPP1R9B	84687	1.223	2	1	1	1	817	89.3	0.603981875
XP_011536358.1	nuclear envelope integral membrane protein 1 isoform X1	NEMP1	23306	0.93	3	1	1	1	492	55.9	0.564996099
XP_011519572.1	codanin-1 isoform X1	CDAN1	146059	0.591	2	1	3	1	1236	135	0.002706696
NP_114063.1	protocadherin alpha-9 isoform 1 precursor	PCDHA9	9752	1.025	2	1	2	1	950	102.3	0.811359693
XP_024303422.1	6-phosphofructo-2-kinase/fructose-2,6-bisphosphatase 2 isoform X1	PFKFB2	5208	0.651	4	2	2	2	505	58.4	0.419529373
NP_001243764.1	queuine tRNA-ribosyltransferase accessory subunit 2 isoform 2	QTRT2	79691	0.662	4	1	4	1	427	48.2	0.313103761
NP_006055.3	ras-related GTP-binding protein B short isoform	RRAGB	10325	1.3	4	1	2	1	346	40.1	0.646753884
XP_016872802.1	steroid hormone receptor ERR1 isoform X1	ESRRA	2101	0.894	6	1	2	1	433	46.5	0.845551435
NP_001078841.1	ubiquinol-cytochrome-c reductase complex assembly factor 3	UQCC3	790955	1.001	24	1	3	1	93	10.1	0.233905907
NP_064617.2	proteasome assembly chaperone 2 isoform 1	PSMG2	56984	0.382	5	1	5	1	264	29.4	0.000734683
NP_002990.2	syndecan-4 precursor	SDC4	6385	1.444	5	1	1	1	198	21.6	0.913946165
XP_011516268.1	myogenesis-regulating glycosidase isoform X1	MYORG	57462	0.724	3	1	1	1	714	81	0.264016759
XP_005267043.1	mannosyl-oligosaccharide 1,2-alpha-mannosidase IA isoform X1	MAN1A1	4121	0.812	2	1	3	1	736	81.3	0.263665986
NP_055569.1	phosphatidylserine synthase 1 isoform 1	PTDSS1	9791	1.536	5	2	4	2	473	55.5	0.111009625
XP_005267567.1	low-density lipoprotein receptor-related protein 10 isoform X1	LRP10	26020	0.755	6	1	2	1	721	76.8	0.09685438
NP_002504.2	nucleoside diphosphate kinase 3 precursor	NME3	4832	1.663	10	1	3	1	169	19	0.000453996
NP_001037.1	solute carrier family 12 member 2 isoform 1	SLC12A2	6558	1.135	2	1	3	1	1212	131.4	0.872182262
XP_016856246.1	pleckstrin homology domain-containing family M member 2 isoform X1	PLEKHM2	23207	0.772	1	1	1	1	1032	113.9	0.875215514
NP_036442.3	chromosome-associated kinesin KIF4A	KIF4A	24137	0.77	2	2	3	2	1232	139.8	0.98720578
NP_057484.3	huntingtin-interacting protein K isoform 1	HYPK	25764	0.713	12	1	1	1	129	14.7	0.623653207
XP_005256178.1	telomeric repeat-binding factor 2 isoform X1	TERF2	7014	1.023	3	1	2	1	545	59.9	0.992573951
NP_001341968.1	histone deacetylase 3 isoform 1	HDAC3	8841	0.768	4	1	3	1	434	49.4	0.025271661
XP_005257439.1	matrix-remodeling-associated protein 7 isoform X1	MXRA7	439921	1.918	3	1	2	1	247	27.4	0.946647324
NP_115834.1	PHD finger protein 6 isoform 1	PHF6	84295	1.154	4	2	5	2	365	41.3	0.884806217
XP_016866325.1	kinesin-like protein KIFC1 isoform X1	KIFC1	3833	0.907	1	1	3	1	674	74	0.400591123
NP_002425.2	DNA-3-methyladenine glycosylase isoform a	MPG	4350	1.294	8	2	2	2	298	32.8	0.903519398
NP_997227.1	SPRY domain-containing protein 4	SPRYD4	283377	1.293	5	1	3	1	207	23.1	0.001031096
XP_005260807.1	kinesin-like protein KIF16B isoform X1	KIF16B	55614	0.994	1	1	1	1	1883	214	0.541210749
NP_001180246.1	spermatogenesis-defective protein 39 homolog isoform 1	VIPAS39	63894	0.706	6	2	3	2	493	57	0.852409483
NP_612404.1	methionine--tRNA ligase, mitochondrial precursor	MARS2	92935	1.097	3	1	1	1	593	66.5	0.464750352
NP_073732.1	elongation of very long chain fatty acids protein 1 isoform 1	ELOVL1	64834	1.087	4	1	3	1	279	32.6	0.831594708
NP_002632.1	phosphatidylinositol N-acetylglucosaminyltransferase subunit A isoform 1	PIGA	5277	1.649	7	2	4	2	484	54.1	0.06703015
XP_024307243.1	general transcription factor IIF subunit 1 isoform X1	GTF2F1	2962	0.571	3	1	2	1	542	61	0.50278391
NP_859056.2	complex III assembly factor LYRM7 isoform 1	LYRM7	90624	0.951	10	1	2	1	104	11.9	0.7832502
XP_011516267.1	trypsin-3 isoform X1	PRSS3	5646	0.832	2	1	6	1	333	35.7	0.573243605
NP_005899.3	beta-mannosidase precursor	MANBA	4126	1.302	2	2	2	2	879	100.8	0.960013253
XP_006712714.1	band 4.1-like protein 5 isoform X1	EPB41L5	57669	1.145	2	1	1	1	740	82.6	0.845340686
NP_001269360.1	ADP-ribosylation factor-like protein 4C isoform 1	ARL4C	10123	1.328	4	1	3	1	201	22.4	0.005534841
XP_006716357.1	cell division cycle-associated protein 2 isoform X3	CDCA2	157313	0.607	3	1	3	1	1028	113.2	0.006384996
NP_056273.2	myotubularin-related protein 9	MTMR9	66036	0.718	2	1	3	1	549	63.4	0.242325414
XP_011535869.1	putative E3 ubiquitin-protein ligase SH3RF2 isoform X1	SH3RF2	153769	0.756	2	1	3	1	735	80.1	0.009327246
XP_016859807.1	protein yippee-like 5 isoform X1	YPEL5	51646	0.926	8	1	3	1	121	13.8	0.253015668
XP_024307388.1	reticulocalbin-3 isoform X1	RCN3	57333	1.557	4	1	1	1	365	41.4	0.932358256

Patil et al.2020. Proteomic and phosphoproteomic profiling of shammah-induced signaling in oral keratinocytes

Supplementary Table S1: List of proteins identified and quantified in OKF6/TERT1-Shammah and OKF6/TERT1-Parental total proteome using TMT-based quantitative proteomic approach

Accession	Protein Description	Gene Symbol	Gene ID	Fold Change	Coverage [%]	# Peptides	# PSMs	# Unique Peptides	# AAs	MW [kDa]	p-value
XP_011518188.1	LARGE xylosyl- and glucuronyltransferase 2 isoform X1	LARGE2	120071	1.081	3	1	3	1	736	83.4	0.052465594
NP_705902.2	pantothenate kinase 2, mitochondrial isoform 1 precursor	PANK2	80025	0.699	3	2	2	2	570	62.6	0.774314754
NP_001312.1	cysteine and glycine-rich protein 2	CSRP2	1466	0.365	5	1	3	1	193	20.9	0.000928403
XP_011516378.1	maternal embryonic leucine zipper kinase isoform X1	MELK	9833	0.651	3	2	2	2	651	74.6	0.908149427
NP_078908.1	histone deacetylase complex subunit SAP30L isoform 1	SAP30L	79685	1.212	5	1	1	1	183	20.9	0.824292635
NP_115497.4	magnesium transporter protein 1	MAGT1	84061	1.272	2	1	3	1	367	41.5	0.24715004
XP_011524424.1	ectopic P granules protein 5 homolog isoform X1	EPG5	57724	1	1	1	4	1	2589	293.1	0.997358707
XP_005262352.1	transcription initiation factor TFIID subunit 1 isoform X1	TAF1	6872	1.098	1	1	2	1	1927	218.4	0.095951882
NP_001186384.1	bromodomain-containing protein 2 isoform 2	BRD2	6046	1.148	2	2	4	2	836	92	0.917034549
XP_011540518.1	cyclin-L2 isoform X1	CCNL2	81669	0.636	3	1	1	1	553	61.2	0.535723206
XP_016871100.1	inhibitor of nuclear factor kappa-B kinase subunit alpha isoform X1	CHUK	1147	0.739	2	1	3	1	814	92.3	0.134704255
NP_002592.1	retinal rod rhodopsin-sensitive cGMP 3',5'-cyclic phosphodiesterase subunit delta isoform 1	PDE6D	5147	0.766	8	1	2	1	150	17.4	0.386214842
XP_016864269.1	glycerol-3-phosphate acyltransferase 3 isoform X1	GPAT3	84803	1.864	4	1	2	1	526	58.6	0.145728369
XP_005251567.1	plasminogen receptor (KT) isoform X1	PLGRKT	55848	1.427	16	2	2	2	147	17.2	0.566939364
NP_476510.1	ARF GTPase-activating protein GIT2 isoform 1	GIT2	9815	0.626	5	2	4	1	759	84.5	0.418744064
NP_003245.1	metalloproteinase inhibitor 1 precursor	TIMP1	7076	1.205	6	1	3	1	207	23.2	0.018973675
XP_016883368.1	prefoldin subunit 4 isoform X1	PFDN4	5203	0.514	6	1	2	1	181	20.6	0.801839981
NP_060271.1	CKLF-like MARVEL transmembrane domain-containing protein 6	CMTM6	54918	1.369	5	1	5	1	183	20.4	0.011046635
XP_011533688.1	adapter protein CIKS isoform X1	TRAF3IP2	10758	0.784	2	1	3	1	574	64.6	0.032347398
NP_057455.1	3-keto-steroid reductase isoform 1	HSD17B7	51478	1.589	4	1	2	1	341	38.2	0.042647992
NP_057723.1	tumor necrosis factor receptor superfamily member 12A precursor	TNFRSF12A	51330	0.758	8	1	3	1	129	13.9	0.008005034
XP_024308807.1	probable tRNA N6-adenosine threonylcarbamoyltransferase, mitochondrial isoform X1	OSGEPL1	64172	0.999	4	1	1	1	425	46.3	0.224049301
NP_001091995.1	cytoskeleton-associated protein 2 isoform 2	CKAP2	26586	0.42	2	1	3	1	683	76.9	0.000751395
NP_036591.2	mitochondrial import inner membrane translocase subunit Tim8 B	TIMM8B	26521	0.96	8	1	3	1	98	11.1	0.692459754
XP_016864546.1	casein kinase I isoform X1	CSNK1G3	1456	1.7	2	1	2	1	481	55.2	0.946878962
NP_689759.2	serum response factor-binding protein 1	SRFBP1	153443	0.999	4	2	5	2	429	48.6	0.851133259
NP_000937.1	DNA primase small subunit	PRIM1	5557	0.828	3	1	2	1	420	49.9	0.528748122
NP_064703.1	cyclin-L1 isoform 1	CCNL1	57018	1.702	2	1	1	1	526	59.6	0.789349008
XP_005261565.1	thioredoxin, mitochondrial isoform X1	TXN2	25828	1.345	11	1	1	1	197	21.8	0.803137319
NP_067067.1	ras-related GTP-binding protein D	RRAGD	58528	0.63	2	1	1	1	400	45.6	0.821475169
NP_000238.1	MHC class I polypeptide-related sequence A isoform 1 (MICA*001) precursor	MICA	100507436	1.512	3	1	3	1	383	42.9	0.943625386
NP_076970.1	phosducin-like protein 3	PDCL3	79031	0.603	5	1	3	1	239	27.6	0.112265515
NP_149079.2	striatin-interacting protein 1 isoform 1	STRIP1	85369	0.835	2	1	3	1	837	95.5	0.000891996
NP_055290.1	trans-1,2-dihydrobenzene-1,2-diol dehydrogenase	DHDH	27294	0.977	4	1	2	1	334	36.4	0.864456534
NP_001035835.1	insulin, isoform 2 precursor	INS-IGF2	723961	2.318	4	1	8	1	200	21.5	0.023060881
NP_004100.1	adrenodoxin, mitochondrial precursor	FDX1	2230	1.825	5	1	3	1	184	19.4	1.56E-05
NP_116185.2	synapse-associated protein 1	SYAP1	94056	0.512	4	1	1	1	352	39.9	0.93936186
NP_005530.3	type I inositol 1,4,5-trisphosphate 5-phosphatase isoform a	INPP5A	3632	0.628	7	1	1	1	412	47.8	0.737003803
NP_036454.1	U6 snRNA-associated Sm-like protein LSm5 isoform a	LSM5	23658	0.855	25	1	2	1	91	9.9	0.887634355
NP_001708.3	zinc finger protein basonuclin-1 isoform a	BNC1	646	0.672	5	2	2	2	994	110.9	0.500851444
NP_079140.2	rRNA methyltransferase 1, mitochondrial precursor	MRM1	79922	1.203	3	1	2	1	353	38.6	0.718170636
NP_061164.3	ADP-ribosylation factor-like protein 6-interacting protein 4 isoform 1	ARL6IP4	51329	0.825	4	1	3	1	421	44.9	0.503116599
XP_006717122.1	nucleoporin GLE1 isoform X1	GLE1	2733	0.831	2	1	2	1	710	80.5	0.603191089
XP_011542514.1	AT-rich interactive domain-containing protein 4B isoform X1	ARID4B	51742	0.756	2	1	1	1	1312	147.7	0.965950388
NP_116584.2	MAP kinase-activated protein kinase 2 isoform 2	MAPKAPK2	9261	1.056	4	2	2	1	400	45.5	0.927290164
XP_011509532.1	NADH dehydrogenase	NDUFB3	4709	1.266	11	1	3	1	98	11.4	0.01682893
XP_016858358.1	lymphocyte function-associated antigen 3 isoform X1	CD58	965	1.291	4	1	3	1	257	28.9	0.00027941
NP_001252518.1	reticulon-3 isoform e	RTN3	10313	0.906	1	1	4	1	1032	112.5	0.764768948
XP_011536973.1	sin3 histone deacetylase corepressor complex component SDS3 isoform X1	SUDS3	64426	1.045	2	1	2	1	407	46.3	0.916429768
NP_001342172.1	uncharacterized protein NCBP2-AS2	NCBP2-AS2	152217	1.287	14	1	3	1	99	10.9	0.414021986
NP_004815.3	chromodomain Y-like protein isoform a	CDYL	9425	1.188	3	1	1	1	544	60.6	0.683244296
XP_024306631.1	replication protein A 70 kDa DNA-binding subunit isoform X1	RPA1	6117	0.828	5	2	2	2	618	68.9	0.946597142
NP_057321.2	anaphase-promoting complex subunit 5 isoform a	ANAPC5	51433	0.568	2	1	1	1	755	85	0.522963158
NP_057706.2	39S ribosomal protein L35, mitochondrial isoform a	MRPL35	51318	1.13	4	1	2	1	188	21.5	0.876045627
NP_787080.2	coiled-coil domain-containing protein 71L	CCDC71L	168455	1.233	6	1	1	1	235	26.2	0.695652761
NP_001161328.1	protein strawberry notch homolog 1 isoform 1	SBNO1	55206	0.775	2	1	3	1	1393	154.2	0.017051018
NP_001317627.1	YTH domain-containing protein 1 isoform 3	YTHDC1	91746	1.651	2	1	2	1	735	85.5	0.601235716
NP_006653.2	helicase SRCAP	SRCAP	10847	0.481	0	1	2	1	3230	343.3	0.813588864
XP_006715563.1	ubiquinone biosynthesis O-methyltransferase, mitochondrial isoform X1	COQ3	51805	1.577	2	1	2	1	410	45.7	0.923240474
NP_004932.1	ATP-dependent RNA helicase DHX8 isoform 1	DHX8	1659	1.033	1	1	1	1	1220	139.2	0.839572449
NP_001035927.1	trafficking protein particle complex subunit 5	TRAPPC5	126003	0.479	14	1	1	1	188	20.8	0.251691161
NP_077024.2	probable ATP-dependent RNA helicase DHX58	DHX58	79132	1.224	2	1	2	1	678	76.6	0.800682771
NP_149977.2	lipase maturation factor 2 isoform 1	LMF2	91289	1.959	3	1	3	1	707	79.6	0.036926618
NP_001092142.1	hephaestin-like protein 1 precursor	HEPHL1	341208	0.967	1	1	1	1	1159	131.5	0.952899566
NP_076994.2	KDEL motif-containing protein 1 isoform 1 precursor	KDELC1	79070	1.149	4	2	3	2	502	58	0.657449078
NP_009017.1	WD repeat and HMG-box DNA-binding protein 1 isoform 1	WDHD1	11169	0.893	1	1	3	1	1129	125.9	0.042782119
XP_011543457.1	serine/threonine-protein kinase MRCK gamma isoform X1	CDC42BPG	55561	0.554	1	1	3	1	1569	174.2	0.017030412
NP_005430.1	myeloid leukemia factor 2	MLF2	8079	1.31	5	1	2	1	248	28.1	0.830236788
NP_001108072.1	SUZ domain-containing protein 1 isoform 1	SZRD1	26099	0.934	14	1	2	1	152	17	0.671908615
NP_002729.2	protein kinase C beta type isoform 2	PRKCB	5579	0.472	2	1	3	1	673	77	0.204994964
NP_001350643.1	protein kinase C-binding protein 1 isoform s	ZMYND8	23613	0.902	2	1	1	1	1241	137.9	0.743714511
XP_016866484.1	serine/threonine-protein phosphatase 1 regulatory subunit 10 isoform X1	PPP1R10	5514	1.117	2	1	3	1	940	99	0.625291753
NP_071736.1	peptide deformylase, mitochondrial precursor	PDF	64146	1.123	8	1	2	1	243	27	0.463613687
NP_951032.1	NADH dehydrogenase	NDUFAF3	25915	0.993	13	1	3	1	184	20.3	0.887473031
NP_036392.2	2-hydroxyacyl-CoA lyase 1 isoform a	HACL1	26061	0.901	3	1	2	1	578	63.7	0.932881707
XP_011507965.1	protoporphyrinogen oxidase isoform X1	PPOX	5498	0.872	8	2	3	2	567	60.6	0.999927514
NP_001432.2	fatty-acid amide hydrolase 1	FAAH	2166	0.971	4	1	3	1	579	63	0.707024485
XP_005252654.1	threonine synthase-like 1 isoform X1	THNSL1	79896	1.175	2	1	2	1	743	83	0.833147309
XP_005269515.1	arginyl-tRNA--protein transferase 1 isoform X2	ATE1	11101	0.84	3	1	1	1	561	64	0.232176355
NP_060337.2	histone PARylation factor 1	HPF1	54969	0.53	4	1	1	1	346	39.4	0.783965953
XP_005260319.1	centrosome-associated protein CEP250 isoform X1	CEP250	11190	1.409	0	1	1	1	2442	281	0.803461558
NP_057056.1	DNA-directed RNA polymerases I and III subunit RPAC2 isoform 1	POLR1D	51082	0.544	24	2	3	2	133	15.2	0.595152054
NP_057657.2	GEM-interacting protein isoform 1	GMIP	51291	0.618	3	1	3	1	970	106.6	0.007727594
NP_001296122.1	ephrin type-B receptor 2 isoform 4 precursor	EPHB2	2048	1.269	2	1	1	1	1055	117.4	0.671859455
NP_110425.2	RCC1-like G exchanging factor-like protein isoform 1	RCC1L	81554	0.891	4	1	1	1	464	49.9	0.836567845
XP_006712039.1	fos-related antigen 2 isoform X1	FOSL2	2355	0.977	3	1	2	1	343	37.1	0.928336504
NP_055643.1	TOX high mobility group box family member 4 isoform 1	TOX4	9878	1.448	2	1	1	1	621	66.2	0.889966586
NP_001073932.1	cytoplasmic dynein 2 heavy chain 1 isoform 2	DYNC2H1	79659	0.653	0	1	3	1	4314	493.1	0.279195209
NP_000145.1	galactokinase	GALK1	2584	1.406	3	1	2	1	392	42.2	0.799466095
NP_653190.2	catechol O-methyltransferase domain-containing protein 1	COMTD1	118881	1.017	7	1	2	1	262	28.8	0.801603121
NP_115873.1	GH3 domain-containing protein isoform 1 precursor	GHDC	84514	1.353	2	1	3	1	530	57.5	0.065898869
XP_011541522.1	poly(A) RNA polymerase GLD2 isoform X1	TENT2	167153	0.608	3	1	1	1	509	58.7	0.039800637
NP_060222.2	TBC1 domain family member 8B isoform a	TBC1D8B	54885	0.811	2	2	5	1	1120	128.6	0.878930705
NP_001231509.1	transformation/transcription domain-associated protein isoform 1	TRRAP	8295	0.924	0	1	1	1	3859	437.3	0.959786768
NP_001074930.1	bifunctional arginine demethylase and lysyl-hydroxylase JMJD6 isoform 1	JMJD6	23210	0.747	3	1	1	1	414	47.6	0.675810532
NP_689413.1	sorting nexin-5 isoform a	SNX5	27131	0.865	8	2	3	2	404	46.8	0.986373093
NP_057184.1	N-alpha-acetyltransferase 20 isoform a	NAA20	51126	1.034	8	1	2	1	178	20.4	0.780052071
NP_065770.1	proline-rich protein 12	PRR12	57479	0.871	1	1	1	1	2036	210.9	0.302520094
NP_001186652.1	cellular retinoic acid-binding protein 2	CRABP2	1382	0.88	12	2	5	2	138	15.7	0.809540155
NP_001011713.2	N-alpha-acetyltransferase 30	NAA30	122830	0.69	5	1	1	1	362	39.3	0.260434585
NP_775489.2	tryptophan 5-hydroxylase 2	TPH2	121278	1.003	4	1	17	1	490	56	0.969041593
NP_060388.2	calcium uniporter regulatory subunit MCUb, mitochondrial	MCUB	55013	0.825	4	1	2	1	336	39.1	0.867957525
XP_016883917.1	intersectin-1 isoform X1	ITSN1	6453	0.657	1	1	1	1	1721	195.3	0.919931466
NP_068779.1	induced myeloid leukemia cell differentiation protein Mcl-1 isoform 1	MCL1	4170	0.661	6	1	2	1	350	37.3	0.426737571
NP_001273309.1	constitutive coactivator of peroxisome proliferator-activated receptor gamma isoform b	FAM120B	84498	0.87	1	1	3	1	933	106	0.060103759
NP_064628.1	mitochondrial import receptor subunit TOM22 homolog	TOMM22	56993	0.886	25	2	3	2	142	15.5	0.960475467
XP_005250117.1	cullin-1 isoform X1	CUL1	8454	0.987	4	2	4	2	776	89.6	0.74986505
NP_115607.1	MIP18 family protein FAM96A isoform a precursor	CIAO2A	84191	0.603	6	1	3	1	160	18.3	0.371996962
XP_016869765.2	probable peptidyl-tRNA hydrolase isoform X1	PTRH1	138428	1.161	4	1	3	1	246	26.1	0.799636411
NP_006323.2	gamma-interferon-inducible lysosomal thiol reductase preproprotein	IFI30	10437	0.938	10	1	2	1	250	27.9	0.824619018
NP_001099026.1	protein THEMIS2 isoform 3	THEMIS2	9473	0.709	4	1	2	1	643	72	0.026359484
NP_057016.1	proteasome maturation protein	POMP	51371	0.708	11	1	1	1	141	15.8	0.6135666
XP_011537421.1	calcium uptake protein 1, mitochondrial isoform X1	MICU1	10367	0.796	2	1	2	1	532	61.2	0.940360939
NP_002394.1	microfibrillar-associated protein 2 isoform a precursor	MFAP2	4237	2.096	5	1	3	1	183	20.8	0.000272832
NP_078898.3	FAST kinase domain-containing protein 1, mitochondrial isoform 1	FASTKD1	79675	0.704	3	1	1	1	847	97.3	0.024462099
XP_006723226.1	ER membrane protein complex subunit 10 isoform X1	EMC10	284361	1.198	4	1	1	1	403	42.9	0.743536859
NP_001071639.1	retrotransposon Gag-like protein 8B	RTL8C	8933	0.813	11	1	2	1	113	13.2	0.122778057
NP_001182665.4	prostamide/prostaglandin F synthase isoform a	PRXL2B	127281	1.034	4	1	2	1	216	23.3	0.709070184
XP_016864078.1	negative elongation factor A isoform X1	NELFA	7469	0.592	2	1	1	1	567	61.7	0.029771326
NP_857593.1	kunitz-type protease inhibitor 1 isoform 1 precursor	SPINT1	6692	1.84	2	1	3	1	529	58.4	0.000588016
NP_001029345.2	mitochondrial 10-formyltetrahydrofolate dehydrogenase precursor	ALDH1L2	160428	1.653	1	1	5	1	923	101.7	0.047732962
NP_775873.2	disco-interacting protein 2 homolog B	DIP2B	57609	1.21	1	1	2	1	1576	171.4	0.96137122
NP_001165948.1	ribonucleoside-diphosphate reductase subunit M2 B isoform 2	RRM2B	50484	0.358	3	1	2	1	423	48.8	0.094385409
XP_016873478.1	T-complex protein 11-like protein 1 isoform X1	TCP11L1	55346	0.543	3	1	2	1	514	57.6	0.747721197
NP_060263.2	ribonuclease P protein subunit p25	RPP25	54913	1.834	6	1	2	1	199	20.6	0.612597707
XP_011520133.1	stAR-related lipid transfer protein 9 isoform X1	STARD9	57519	0.881	0	1	1	1	4705	516.5	0.442648753
NP_078820.2	mitochondrial ubiquitin ligase activator of NFKB 1	MUL1	79594	1.092	6	1	2	1	352	39.8	0.927225724
NP_001304824.1	poly	PARP10	84875	0.616	2	1	1	1	1037	111.3	0.860835771
XP_005246783.1	pre-mRNA-splicing factor CWC22 homolog isoform X1	CWC22	57703	0.898	2	1	3	1	908	105.4	0.057219681
NP_065717.1	dual specificity protein kinase CLK4	CLK4	57396	0.567	3	1	1	1	481	57.5	0.945570416
NP_001073594.1	caspase-8 isoform G precursor	CASP8	841	0.484	4	1	3	1	538	61.8	0.003975667
NP_001317547.1	cysteine and histidine-rich protein 1 isoform 3	CYHR1	50626	0.734	3	1	2	1	404	43.8	0.897694423
NP_005605.1	GTP-binding protein Rheb	RHEB	6009	0.868	4	1	3	1	184	20.5	0.007369974
NP_957718.1	neuropilin-2 isoform 1 precursor	NRP2	8828	0.585	1	1	1	1	931	104.8	0.144573459
NP_077271.1	derlin-1 isoform a	DERL1	79139	0.776	4	1	2	1	251	28.8	0.874438292
XP_011526990.1	disks large-associated protein 4 isoform X1	DLGAP4	22839	0.705	1	1	3	1	992	107.9	0.088466633
NP_991403.1	lipolysis-stimulated lipoprotein receptor isoform 2	LSR	51599	0.985	2	1	1	1	649	71.4	0.802150669
NP_005104.3	golgin subfamily A member 5	GOLGA5	9950	1.056	2	1	1	1	731	82.9	0.7380073
NP_001505.1	transcription initiation factor IIB	GTF2B	2959	1.093	9	2	2	2	316	34.8	0.784176667
XP_011531316.1	lipid droplet-associated hydrolase isoform X1	LDAH	60526	1.276	3	1	3	1	331	38	0.004769108
NP_060670.1	high mobility group protein 20A isoform a	HMG20A	10363	1.003	3	1	2	1	347	40.1	0.702524359
NP_001003787.1	STE20-related kinase adapter protein alpha isoform 1	STRADA	92335	0.679	3	1	1	1	431	48.3	0.47119416
XP_016856104.1	epidermal growth factor receptor substrate 15 isoform X1	EPS15	2060	0.84	1	1	3	1	933	102.4	0.062291208
NP_116147.1	PHD finger-like domain-containing protein 5A	PHF5A	84844	1.645	7	1	3	1	110	12.4	0.004107094
NP_037374.1	cilia- and flagella-associated protein 20	CFAP20	29105	1.135	4	1	3	1	193	22.8	0.228188347
NP_001841.2	collagen alpha-1(VIII) chain precursor	COL8A1	1295	2.212	1	1	3	1	744	73.3	1.88E-06
NP_071361.2	G patch domain-containing protein 3	GPATCH3	63906	0.65	2	1	4	1	525	59.3	0.344222265
XP_011509096.1	general transcription and DNA repair factor IIH helicase subunit XPB isoform X1	ERCC3	2071	1.204	2	1	3	1	788	89.9	0.085268573
NP_001116295.1	general transcription factor 3C polypeptide 5 isoform 1	GTF3C5	9328	0.907	3	2	3	2	526	60.3	0.961961619
NP_001180304.1	NADH dehydrogenase	NDUFA11	126328	2.02	10	1	2	1	228	23.4	0.664333393
NP_056041.1	protein CLEC16A isoform 1	CLEC16A	23274	0.86	1	1	1	1	1053	117.6	0.279564401
XP_016879247.1	protein spinster homolog 1 isoform X2	SPNS1	83985	0.978	4	2	4	2	578	61.6	0.601355309
NP_057083.2	adiponectin receptor protein 1	ADIPOR1	51094	0.886	3	1	3	1	375	42.6	0.542250744
NP_055299.1	adenylyltransferase and sulfurtransferase MOCS3	MOCS3	27304	0.528	2	1	1	1	460	49.6	0.931333979
NP_001317074.1	synembryn-B isoform 1	RIC8B	55188	0.7	2	1	3	1	560	63.5	0.000435016
XP_024304174.1	condensin-2 complex subunit D3 isoform X1	NCAPD3	23310	0.377	1	1	2	1	1498	168.8	0.119753019
NP_060585.2	protein SDA1 homolog isoform 1	SDAD1	55153	1.247	1	1	1	1	687	79.8	0.714304304
NP_006464.1	TAF6-like RNA polymerase II p300/CBP-associated factor-associated factor 65 kDa subunit 6L	TAF6L	10629	1.232	4	1	3	1	622	67.8	0.069566644
NP_955450.2	lysosomal Pro-X carboxypeptidase isoform 2 precursor	PRCP	5547	1.038	2	1	3	1	517	58.1	0.68029417
NP_001121598.1	AP-4 complex subunit sigma-1 isoform 2	AP4S1	11154	0.394	7	1	1	1	144	17	0.222291088
NP_008881.2	synapsin-1 isoform Ia	SYN1	6853	1.377	3	2	3	2	705	74.1	0.436770616
NP_001186673.1	SAA2-SAA2 protein precursor	SAA2-SAA4	100528017	0.596	7	1	2	1	208	23.3	0.878950862
XP_011517817.1	protein FAM208B isoform X1	FAM208B	54906	0.973	1	1	3	1	2716	301.7	0.826751647
NP_008817.1	ATP synthase subunit epsilon, mitochondrial	ATP5F1E	514	1.588	29	2	4	2	51	5.8	0.16239575
NP_443076.2	WD repeat-containing protein 34	WDR34	89891	0.553	2	1	1	1	536	57.8	0.799796846
XP_011532165.1	glutamine-rich protein 1 isoform X1	QRICH1	54870	0.572	1	1	3	1	776	86.4	0.002330319
XP_016868300.1	PHD finger protein 14 isoform X1	PHF14	9678	1.093	1	1	3	1	958	108.1	0.516757334
NP_055888.1	signal-induced proliferation-associated 1-like protein 3	SIPA1L3	23094	0.838	1	1	1	1	1781	194.5	0.868776556
NP_003780.1	tumor necrosis factor receptor type 1-associated DEATH domain protein	TRADD	8717	0.627	3	1	3	1	312	34.2	8.35E-05
NP_001352009.1	dnaJ homolog subfamily B member 12 isoform 2	DNAJB12	54788	1.293	3	1	2	1	377	42.3	0.941786064
NP_877429.2	tumor protein p63-regulated gene 1-like protein	TPRG1L	127262	0.762	3	1	2	1	272	30.2	0.580088768
XP_011520551.1	inositol hexakisphosphate and diphosphoinositol-pentakisphosphate kinase 1 isoform X1	PPIP5K1	9677	0.652	1	1	1	1	1479	164.3	0.926082974
XP_016874706.1	intraflagellar transport protein 81 homolog isoform X1	IFT81	28981	0.852	2	1	2	1	676	79.7	0.325520065
NP_004727.2	xenotropic and polytropic retrovirus receptor 1 isoform 1	XPR1	9213	0.694	3	1	2	1	696	81.5	0.012314967
NP_001092872.1	cyclin-K	CCNK	8812	0.747	2	1	1	1	580	64.2	0.435051548
XP_005252324.1	GTPase-activating Rap/Ran-GAP domain-like protein 3 isoform X1	GARNL3	84253	1.101	1	1	5	1	1076	119.4	0.832506049
NP_009209.1	gamma-aminobutyric acid receptor-associated protein	GABARAP	11337	0.385	9	1	3	1	117	13.9	0.000263032
NP_057058.2	lambda-crystallin homolog isoform 1	CRYL1	51084	0.417	3	1	2	1	319	35.4	0.371905231
XP_016863700.1	microsomal glutathione S-transferase 2 isoform X1	MGST2	4258	1.625	10	1	3	1	147	16.6	0.014519289
XP_006715085.1	protein FAM83B isoform X1	FAM83B	222584	0.72	4	2	2	2	1011	114.7	0.715204285
NP_065699.1	twisted gastrulation protein homolog 1 precursor	TWSG1	57045	1.031	7	1	1	1	223	25	0.676141513
NP_057088.2	intraflagellar transport protein 52 homolog isoform 1	IFT52	51098	0.693	5	1	3	1	437	49.7	0.015535432
NP_001423.1	proepiregulin preproprotein	EREG	2069	0.901	7	1	1	1	169	19	0.858505569
NP_002731.4	protein kinase C iota type	PRKCI	5584	0.479	5	1	2	1	596	68.2	0.191117936
NP_004984.2	ataxin-3 reference isoform	ATXN3	4287	0.874	3	1	3	1	361	41.2	0.069814481
NP_110426.4	protein YIPF5 isoform a	YIPF5	81555	1.169	4	1	6	1	257	28	0.004568118
XP_016866335.1	centrosomal protein of 85 kDa-like isoform X1	CEP85L	387119	3.348	1	1	3	1	808	92.4	0.80156648
NP_001257823.1	trafficking protein particle complex subunit 3 isoform 2	TRAPPC3	27095	0.411	4	1	3	1	188	21.2	0.000844063
NP_003327.2	ubiquitin-conjugating enzyme E2 A isoform 1	UBE2A	7319	0.799	11	1	2	1	152	17.3	0.875449166
NP_004413.1	segment polarity protein dishevelled homolog DVL-2	DVL2	1856	0.592	2	1	2	1	736	78.9	0.495637502
NP_001350493.1	mth938 domain-containing protein isoform d	AAMDC	28971	0.953	6	1	2	1	141	15.6	0.967665232
XP_016883495.1	protein MANBAL isoform X1	MANBAL	63905	1.569	9	1	3	1	85	9.5	0.000147185
NP_001508.1	general transcription factor IIH subunit 4	GTF2H4	2968	1.052	2	1	3	1	462	52.2	0.078488572
NP_001017536.1	vitamin D3 receptor isoform VDRB1	VDR	7421	0.855	3	1	3	1	477	53.8	0.28499544
NP_079219.1	transmembrane protein 156 isoform 1	TMEM156	80008	0.631	5	1	2	1	296	34.3	0.02613481
NP_116288.2	protein FAM83A isoform a	FAM83A	84985	0.796	3	1	1	1	434	47.4	0.736825417
NP_542388.2	radical S-adenosyl methionine domain-containing protein 2	RSAD2	91543	0.767	5	1	2	1	361	42.1	0.88598875
XP_006724688.1	disks large homolog 3 isoform X1	DLG3	1741	0.938	2	1	2	1	849	94	0.643012862
XP_016866934.1	multidrug resistance-associated protein 7 isoform X1	ABCC10	89845	0.712	1	1	3	1	1558	168.3	0.025925347
NP_031399.2	leucine-rich repeat protein SHOC-2 isoform 1	SHOC2	8036	0.937	2	1	3	1	582	64.8	0.890462913
NP_001290072.1	HAUS augmin-like complex subunit 3	HAUS3	79441	1.012	2	1	2	1	603	69.6	0.789157845
NP_078836.1	acyl-CoA synthetase short-chain family member 3, mitochondrial isoform 1 precursor	ACSS3	79611	1.12	2	1	3	1	686	74.7	0.599442557
NP_073593.2	chromatin modification-related protein MEAF6 isoform 1	MEAF6	64769	1.142	6	1	1	1	201	22.7	0.619077172
NP_001268400.1	inositol hexakisphosphate and diphosphoinositol-pentakisphosphate kinase 2 isoform 3	PPIP5K2	23262	0.637	1	1	3	1	1278	144.3	0.507066242
NP_116249.2	protein LTV1 homolog isoform 1	LTV1	84946	0.5	4	1	3	1	475	54.8	0.005792439
XP_011543886.1	MICOS complex subunit MIC26 isoform X1	APOO	79135	1.472	5	1	1	1	202	22.8	0.736983989
NP_031394.2	ras GTPase-activating protein 3 isoform 1	RASA3	22821	0.999	2	1	1	1	834	95.6	0.617919889
NP_002349.1	mitotic spindle assembly checkpoint protein MAD2A	MAD2L1	4085	0.698	4	1	3	1	205	23.5	0.313713191
NP_060420.2	coiled-coil domain-containing protein 40 isoform 1	CCDC40	55036	1.568	1	1	5	1	1142	130	0.019294575
NP_055956.1	glycerol-3-phosphate dehydrogenase 1-like protein	GPD1L	23171	1.274	3	1	3	1	351	38.4	0.104308032
XP_016883624.1	tumor necrosis factor receptor superfamily member 5 isoform X1	CD40	958	1.99	3	1	3	1	323	35.7	1.48E-05
NP_079330.2	deubiquitinating protein VCIP135	VCPIP1	80124	0.78	2	1	1	1	1222	134.2	0.221454015
NP_060716.2	coiled-coil domain-containing protein 25 isoform 1	CCDC25	55246	0.662	5	1	2	1	208	24.5	0.700252555
NP_003300.1	testis-specific Y-encoded-like protein 1	TSPYL1	7259	0.668	5	1	3	1	437	49.2	0.107036483
XP_005256774.1	nuclear cap-binding protein subunit 3 isoform X1	NCBP3	55421	0.951	2	1	3	1	632	72	0.689854604
XP_024306539.1	alpha-N-acetylglucosaminidase isoform X1	NAGLU	4669	0.728	2	1	1	1	762	84.3	0.186533914
NP_001854.1	cytochrome c oxidase subunit 6B1	COX6B1	1340	0.919	31	1	1	1	86	10.2	0.813883138
NP_996670.1	mortality factor 4-like protein 1 isoform 2	MORF4L1	10933	1.269	2	1	3	1	362	41.4	0.255016592
NP_061977.1	protein lin-37 homolog	LIN37	55957	0.643	5	1	3	1	246	28.4	0.02302657
NP_872353.2	transmembrane emp24 domain-containing protein 4 isoform 1 precursor	TMED4	222068	2.904	4	1	3	1	227	25.9	0.000790701
NP_001316681.1	protein FAM136A isoform 2	FAM136A	84908	1.184	3	1	1	1	245	26.8	0.805596733
NP_852127.2	39S ribosomal protein L55, mitochondrial isoform b	MRPL55	128308	1.724	5	1	6	1	164	18.9	0.001774873
NP_006432.1	5-formyltetrahydrofolate cyclo-ligase isoform a	MTHFS	10588	0.797	6	1	2	1	203	23.2	0.315070156
NP_057240.3	myotubularin-related protein 2 isoform 1	MTMR2	8898	0.577	3	1	3	1	643	73.3	0.003089493
XP_005255334.1	ubinuclein-1 isoform X1	UBN1	29855	1.953	2	1	1	1	1161	124.4	0.465586846
NP_078880.2	RNA polymerase II-associated protein 3 isoform 1	RPAP3	79657	0.551	1	1	1	1	665	75.7	0.943614818
NP_689494.2	RAB6-interacting golgin isoform a precursor	GORAB	92344	1.053	5	1	1	1	394	45	0.495296295
XP_024306203.1	synaptotagmin-like protein 1 isoform X1	SYTL1	84958	1.366	1	1	3	1	645	70.1	0.011598331
NP_689596.4	DIS3-like exonuclease 2 isoform 1	DIS3L2	129563	0.437	1	1	3	1	885	99.2	9.40E-05
NP_001317147.1	cyclin-Y-like protein 1 isoform 3	CCNYL1	151195	0.839	5	1	1	1	359	40.7	0.829032922
NP_055508.3	EEF1AKMT4-ECE2 readthrough transcript protein	EEF1AKMT4-ECE2	110599583	0.84	2	1	1	1	883	99.7	0.960978112
NP_001284636.1	calmodulin-regulated spectrin-associated protein 2 isoform 1	CAMSAP2	23271	1.084	1	2	2	2	1489	168	0.822986271
NP_787072.2	exocyst complex component 8	EXOC8	149371	0.56	5	1	2	1	725	81.7	0.850692962
XP_005269978.1	cyclin-J isoform X5	CCNJ	54619	0.697	4	1	3	1	269	30.7	0.082797512
NP_001287831.1	complement decay-accelerating factor isoform 5 precursor	CD55	1604	2.081	2	1	1	1	444	49.3	0.980135095
NP_008998.1	histone acetyltransferase KAT7 isoform 1	KAT7	11143	0.879	4	1	1	1	611	70.6	0.373064599
NP_060366.1	glucose-induced degradation protein 8 homolog	GID8	54994	0.913	5	1	1	1	228	26.7	0.910879848
NP_060911.2	peroxisomal trans-2-enoyl-CoA reductase	PECR	55825	1.55	3	1	2	1	303	32.5	0.90056648
NP_001137436.1	TBC1 domain family member 7 isoform a	TBC1D7	51256	0.66	6	1	1	1	293	34	0.577305983
NP_037450.2	protein PRRC2B	PRRC2B	84726	0.452	1	1	1	1	2229	242.8	0.084686923
NP_001350870.1	armadillo repeat-containing protein 8 isoform 6	ARMC8	25852	0.649	2	1	1	1	673	75.5	0.787198035
XP_011514708.1	B-cell receptor-associated protein 29 isoform X1	BCAP29	55973	0.869	2	1	3	1	348	40.8	0.906360942
NP_003052.2	slit homolog 1 protein precursor	SLIT1	6585	2.919	1	1	4	1	1534	167.8	0.08065497
NP_057159.2	vacuolar protein-sorting-associated protein 36 isoform 1	VPS36	51028	1.048	3	1	2	1	386	43.8	0.803342179
NP_057107.4	deubiquitinase OTUD6B isoform 1	OTUD6B	51633	0.738	3	1	3	1	293	33.8	0.016255709
NP_078922.1	protein zyg-11 homolog B	ZYG11B	79699	0.866	2	1	1	1	744	83.9	0.943131903
NP_003795.2	receptor-interacting serine/threonine-protein kinase 1 isoform 1	RIPK1	8737	0.672	2	1	2	1	671	75.9	0.089935048
XP_011509886.1	NIF3-like protein 1 isoform X1	NIF3L1	60491	0.641	4	1	1	1	377	41.9	0.991900935
XP_024303103.1	rho guanine nucleotide exchange factor 10 isoform X2	ARHGEF10	9639	0.592	1	1	2	1	1373	151.9	0.607380513
NP_003031.3	anion exchange protein 2 isoform 1	SLC4A2	6522	1.132	1	1	1	1	1241	136.9	0.848825893
XP_011538521.1	tetraspanin-14 isoform X1	TSPAN14	81619	0.814	6	1	1	1	284	32.4	0.877892784
NP_078925.3	Bardet-Biedl syndrome 1 protein	BBS1	582	0.559	3	1	1	1	593	65	0.467285693
XP_016862847.1	calcium-dependent secretion activator 1 isoform X1	CADPS	8618	0.734	1	1	2	1	1433	161.6	0.191205689
XP_016866890.1	mRNA-capping enzyme isoform X1	RNGTT	8732	0.421	2	1	3	1	603	69.4	4.35E-05
NP_478070.1	protein FAM207A isoform 1	FAM207A	85395	1.024	6	1	3	1	230	25.4	0.931533689
NP_060566.2	notchless protein homolog 1 isoform a	NLE1	54475	0.837	2	1	2	1	485	53.3	0.800867813
NP_001304818.1	putative deoxyribonuclease TATDN1 isoform c	TATDN1	83940	0.624	3	1	3	1	333	37.6	0.006161331
NP_001307707.1	secretory carrier-associated membrane protein 2 isoform 1	SCAMP2	10066	1.083	3	1	1	1	372	41.5	0.878183005
NP_001241667.1	rho-related GTP-binding protein RhoE precursor	RND3	390	0.781	4	1	3	1	244	27.4	0.003576389
NP_872294.2	ubiquitin carboxyl-terminal hydrolase 12	USP12	219333	1.031	2	1	2	1	370	42.8	0.854055747
NP_060496.2	phosphofurin acidic cluster sorting protein 1	PACS1	55690	0.761	1	1	2	1	963	104.8	0.4036669
NP_057709.2	serine/Arginine-related protein 53 isoform 1	RSRC1	51319	1.451	4	1	1	1	334	38.7	0.805988361
XP_016882575.1	epidermal growth factor receptor substrate 15-like 1 isoform X1	EPS15L1	58513	1.47	1	1	3	1	926	101.3	9.76E-06
NP_001070736.1	abscission/NoCut checkpoint regulator isoform 1	ZFYVE19	84936	0.91	2	1	1	1	471	51.5	0.97520808
NP_705582.1	protein UXT isoform 1	UXT	8409	0.633	7	1	2	1	169	19.6	0.754881445
NP_001120683.1	shootin-1 isoform a	SHTN1	57698	0.927	2	1	1	1	631	71.6	0.799970116
NP_000308.1	6-pyruvoyl tetrahydrobiopterin synthase	PTS	5805	0.649	6	1	3	1	145	16.4	0.002560754
XP_011540493.1	protein wntless homolog isoform X1	WLS	79971	1.2	2	1	1	1	545	62.7	0.844178038
NP_006755.4	interferon-related developmental regulator 2	IFRD2	7866	0.547	3	1	1	1	506	54.8	0.647224953
NP_006466.2	periostin isoform 1 precursor	POSTN	10631	0.708	2	1	1	1	836	93.3	0.549405903
NP_008950.1	ubiquitin-conjugating enzyme E2 C isoform 1	UBE2C	11065	0.91	9	1	1	1	179	19.6	0.764466998
XP_011514555.1	neuronal cell adhesion molecule isoform X1	NRCAM	4897	0.663	2	1	1	1	1311	144.6	0.245613649
NP_000471.1	AMP deaminase 3 isoform 1A	AMPD3	272	0.343	1	1	2	1	776	89.7	0.303564744
NP_060567.1	HAUS augmin-like complex subunit 2 isoform 1	HAUS2	55142	0.62	7	1	2	1	235	26.9	0.009098543
NP_001350698.1	matrix-remodeling-associated protein 7 isoform 4	MXRA7	439921	1.811	5	1	3	1	213	23.4	0.000828125

Patil et al,2020. Proteomic and phosphoproteomic profiling of shammah-induced signaling in oral keratinocytes

Supplementary Table S1: List of proteins identified and quantified in OKF6/TERT1-Shammah and OKF6/TERT1-Parental total protome using TMT-based quantitative proteomic approach

Accession	Protein Description	Gene Symbol	Gene ID	Fold Change	Coverage [%]	# Peptides	# PSMs	# Unique Peptides	# AAs	MW [kDa]	p-value
XP_011509929.1	protein lifeguard 3 isoform X1	TMBIM1	64114	1.282	5	1	2	1	311	34.6	0.75571377
NP_787103.1	prostaglandin reductase 3 isoform 1	ZADH2	284273	0.956	2	1	3	1	377	40.1	0.665403568
XP_005245867.1	cation-transporting ATPase 13A2 isoform X1	ATP13A2	23400	1.514	1	1	2	1	1201	130.5	0.352183722
NP_079023.2	Hermansky-Pudlak syndrome 6 protein	HPS6	79803	0.815	2	1	3	1	775	82.9	0.819611269
XP_005250783.1	nuclear envelope pore membrane protein POM 121 isoform X1	POM121	9883	1.403	1	1	3	1	1249	127.6	0.033710381
NP_055082.1	small acidic protein	C11orf58	10944	0.432	4	1	6	1	183	20.3	0.000496942
NP_722517.3	CDK2-associated and cullin domain-containing protein 1	CACUL1	143384	0.561	6	1	3	1	369	41	0.829517533
NP_005572.2	basal cell adhesion molecule isoform 1 precursor	BCAM	4059	0.495	2	1	1	1	628	67.4	0.743703018
NP_060738.2	WD repeat-containing protein 41	WDR41	55255	0.588	4	1	3	1	459	51.7	0.008918547
NP_078939.3	probable aminopeptidase NPEPL1 isoform 1	NPEPL1	79716	0.869	2	1	3	1	523	55.8	0.414498831
NP_006433.2	dr1-associated corepressor	DRAP1	10589	0.667	5	1	3	1	205	22.3	5.60E-05
XP_005255532.2	target of rapamycin complex subunit LST8 isoform X1	MLST8	64223	1.278	5	1	1	1	351	38.5	0.7704869
NP_056485.2	sentrin-specific protease 3	SENP3	26168	0.692	2	1	2	1	574	65	0.702140225
NP_954981.1	coiled-coil domain-containing protein 137	CCDC137	339230	1.285	4	1	1	1	289	33.2	0.97432399
NP_071739.2	protein Njmu-R1	C17orf75	64149	0.783	3	1	1	1	396	44.6	0.892182542
XP_005268185.1	breast cancer metastasis-suppressor 1-like protein isoform X1	BRMS1L	84312	1.053	4	1	1	1	330	38.4	0.814154931
NP_001135764.1	VIP36-like protein isoform 1 precursor	LMAN2L	81562	1.172	2	1	1	1	359	41	0.926715116
XP_011527888.1	NADH dehydrogenase	NDUFV3	4731	1.769	3	1	3	1	494	53.3	0.0037446
XP_005248591.1	DNA polymerase kappa isoform X1	POLK	51426	0.555	2	1	3	1	884	100.4	0.001547116
XP_011521726.1	nucleolar protein 3 isoform X1	NOL3	8996	0.545	3	1	2	1	295	32.4	0.454071703
XP_005256393.1	casein kinase I isoform X1	CSNK1D	1453	1.096	3	1	3	1	450	51.5	0.267983593
XP_011544061.1	cardiotrophin-1 isoform X1	CTF1	1489	0.644	8	1	1	1	223	23.6	0.546385184
XP_006721703.1	endoplasmic reticulum protein SC65 isoform X1	P3H4	10609	0.739	2	1	3	1	531	60.1	0.056428022
NP_067033.1	trafficking protein particle complex subunit 1	TRAPPC1	58485	0.664	6	1	2	1	145	16.8	0.461546454
NP_036213.2	RNA polymerase II elongation factor ELL2	ELL2	22936	1.304	1	1	3	1	640	72.3	0.065911899
XP_005271049.1	E3 ubiquitin-protein ligase RNF220 isoform X1	RNF220	55182	0.93	2	1	1	1	592	65.6	0.305559746
NP_060055.2	solute carrier family 2, facilitated glucose transporter member 6 isoform 1	SLC2A6	11182	0.729	2	1	3	1	507	54.5	0.002815058
NP_009216.1	gamma-aminobutyric acid receptor-associated protein-like 2	GABARAPL2	11345	0.811	11	1	1	1	117	13.7	0.947065764
XP_005259114.1	protein SMG9 isoform X1	SMG9	56006	0.531	3	1	1	1	521	57.4	0.042651346
NP_054900.2	galectin-related protein	LGALSL	29094	0.723	6	1	1	1	172	19	0.488858494
NP_001893.2	cystathionine gamma-lyase isoform 1	CTH	1491	0.307	5	1	2	1	405	44.5	0.176334525
NP_078975.2	AN1-type zinc finger protein 1 isoform a	ZFAND1	79752	0.717	4	1	1	1	268	30.8	0.508510062
XP_005273855.1	angiomotin-like protein 1 isoform X1	AMOTL1	154810	0.572	2	1	1	1	985	109.6	0.800204357
NP_004764.1	zinc finger HIT domain-containing protein 3 isoform 1	ZNHIT3	9326	0.974	6	1	1	1	155	17.6	0.459752899
NP_003815.1	FAS-associated death domain protein	FADD	8772	0.496	9	1	3	1	208	23.3	0.00013299
NP_001166038.1	myeloid differentiation primary response protein MyD88 isoform 1	MYD88	4615	0.538	2	1	3	1	317	35.4	0.002197511
XP_005261628.1	desumoylating isopeptidase 1 isoform X1	DESI1	27351	0.746	4	1	3	1	209	23	0.041669901
NP_116198.3	mitoguardin 2 isoform 1	MIGA2	84895	0.579	2	1	3	1	657	72.1	0.131970276
NP_009115.2	nischarin isoform 1	NISCH	11188	1.133	1	1	1	1	1504	166.5	0.831779831
NP_775754.2	cytochrome P450 4F22	CYP4F22	126410	19.422	2	1	1	1	531	61.9	0.207283415
NP_079224.1	ubiquitin carboxyl-terminal hydrolase MINDY-3 isoform a	MINDY3	80013	0.709	2	1	1	1	445	49.7	0.839606952
NP_000629.3	vitronectin precursor	VTN	7448	1.221	3	1	1	1	478	54.3	0.849577074
NP_057108.2	RNA-binding motif protein, X-linked 2	RBMX2	51634	1.134	3	1	3	1	322	37.3	0.618723225
NP_060662.2	prolyl 3-hydroxylase 2 isoform a precursor	P3H2	55214	0.811	2	1	4	1	708	80.9	0.658239987
NP_060599.1	pyridoxine-5'-phosphate oxidase	PNPO	55163	0.62	3	1	1	1	261	30	0.821318265
NP_001159735.1	translation initiation factor IF-3, mitochondrial	MTIF3	219402	1.226	3	1	3	1	278	31.7	0.639523962
NP_060762.3	glutamyl-tRNA(Gln) amidotransferase subunit A, mitochondrial	QRSL1	55278	0.843	3	1	2	1	528	57.4	0.17066715
XP_006714068.1	amyloid-beta A4 precursor protein-binding family B member 2 isoform X1	APBB2	323	1.041	2	1	1	1	761	83.7	0.495593657
NP_001713.2	DNA-directed RNA polymerase III subunit RPC4	POLR3D	661	1.009	4	1	1	1	398	44.4	0.948727304
NP_079232.3	transmembrane protein 62 isoform a	TMEM62	80021	0.88	4	1	1	1	643	73.1	0.288817631
NP_003250.3	intercellular adhesion molecule 5 precursor	ICAM5	7087	1.254	1	1	2	1	924	97.1	0.6050866
NP_115963.1	protein dpy-30 homolog isoform 1	DPY30	84661	0.346	16	1	2	1	99	11.2	0.04936043
NP_006582.1	DNA polymerase delta subunit 3 isoform 1	POLD3	10714	0.968	3	1	1	1	466	51.4	0.584464566
NP_061118.1	H/ACA ribonucleoprotein complex subunit 3	NOP10	55505	2.329	14	1	3	1	64	7.7	0.782112742
XP_011511422.1	probable cation-transporting ATPase 13A3 isoform X1	ATP13A3	79572	1.453	1	1	2	1	1256	141.6	0.688323916
XP_011513863.1	cerebral cavernous malformations 2 protein isoform X1	CCM2	83605	1.385	3	1	1	1	506	55.9	0.772936592
NP_065872.1	vacuolar protein sorting-associated protein 13C isoform 2A	VPS13C	54832	0.601	1	1	3	1	3753	422.1	0.004377277
NP_055385.3	ADP-ribosylation factor GTPase-activating protein 3 isoform 1	ARFGAP3	26286	0.826	2	1	2	1	516	56.9	0.43015903
NP_510870.1	cytochrome c oxidase assembly protein COX15 homolog isoform 1	COX15	1355	0.486	4	1	3	1	410	46	0.005917183
NP_006395.2	endothelial protein C receptor precursor	PROCR	10544	1.794	4	1	1	1	238	26.7	0.872485864
XP_024303217.1	delta-aminolevulinic acid dehydratase isoform X1	ALAD	210	1.161	3	1	1	1	359	39	0.698306978
XP_024309194.1	vacuolar protein sorting-associated protein 8 homolog isoform X1	VPS8	23355	0.918	1	1	3	1	1428	161.7	0.604789227
NP_059140.1	chromatin accessibility complex protein 1	CHRAC1	54108	0.707	20	1	3	1	131	14.7	0.12091827
NP_055037.1	NADH dehydrogenase	NDUFA8	4702	1.132	8	1	1	1	172	20.1	0.934696176
XP_005267269.2	rho GTPase-activating protein 18 isoform X1	ARHGAP18	93663	0.598	2	1	2	1	713	80.5	0.23392279
NP_004765.2	mediator of RNA polymerase II transcription subunit 1	MED1	5469	1.061	1	1	1	1	1581	168.4	0.463992608
NP_055224.1	TAF5-like RNA polymerase II p300/CBP-associated factor-associated factor 65 kDa subunit 5L isoform a	TAF5L	27097	0.514	3	1	1	1	589	66.1	0.042923725
XP_006723899.1	NFX1-type zinc finger-containing protein 1 isoform X1	ZNFX1	57169	0.674	0	1	1	1	1918	220.1	0.892737261
NP_115731.2	vacuolar fusion protein MON1 homolog A isoform a	MON1A	84315	0.596	1	1	3	1	652	72.8	0.001996961
XP_024304204.1	mitochondrial import inner membrane translocase subunit Tim10 isoform X1	TIMM10	26519	1.675	16	1	2	1	90	10.3	0.861218496
NP_060743.2	transmembrane protein 143 isoform a	TMEM143	55260	0.776	2	1	1	1	459	51.7	0.927948607
XP_005261260.1	germinal-center associated nuclear protein isoform X1	MCM3AP	8888	0.89	1	1	3	1	1980	218.3	0.217583284
NP_001507.2	general transcription factor IIH subunit 3 isoform a	GTF2H3	2967	0.676	4	1	2	1	308	34.4	0.801331308
NP_076971.1	ERI1 exoribonuclease 3 isoform 1	ERI3	79033	0.83	3	1	2	1	337	37.2	0.7756796
NP_075559.2	spermatogenesis-associated serine-rich protein 2	SPATS2	65244	0.728	2	1	1	1	545	59.5	0.891354171
NP_000259.1	merlin isoform 1	NF2	4771	0.476	3	1	2	1	595	69.6	0.941056551
NP_001185882.1	zinc finger FYVE domain-containing protein 21 isoform 1	ZFYVE21	79038	1.037	8	1	1	1	252	28.5	0.55557293
XP_006719890.1	mothers against decapentaplegic homolog 9 isoform X1	SMAD9	4093	0.723	3	1	1	1	479	53.7	0.353158525
XP_005264329.1	general transcription factor 3C polypeptide 2 isoform X1	GTF3C2	2976	0.982	2	1	1	1	950	105	0.755351505
NP_001271335.1	transmembrane protein 40 isoform a	TMEM40	55287	1.502	4	1	2	1	249	26.9	0.647647986
XP_006717991.1	AMSH-like protease isoform X1	STAMBPL1	57559	0.592	5	1	2	1	436	49.8	0.56089633
NP_037462.1	nucleoside diphosphate kinase 7 isoform a	NME7	29922	0.775	3	1	1	1	376	42.5	0.605922464
XP_016876094.1	propionyl-CoA carboxylase alpha chain, mitochondrial isoform X1	PCCA	5095	0.995	2	1	1	1	744	82.2	0.549502699
NP_001001433.1	syntaxin-16 isoform a	STX16	8675	0.696	3	1	3	1	325	37	0.001445631
XP_005258776.1	protein LSM14 homolog A isoform X1	LSM14A	26065	0.828	4	1	1	1	487	53.4	0.849448594
NP_001185987.1	C15orf38-AP3S2 fusion protein	C15orf38-AP3S2	100526783	1.043	2	1	1	1	394	43.9	0.773619873
XP_024304579.1	polypeptide N-acetylgalactosaminyltransferase 6 isoform X1	GALNT6	11226	0.828	4	1	1	1	622	71.1	0.907323322
XP_011516871.1	rap guanine nucleotide exchange factor 1 isoform X1	RAPGEF1	2889	0.473	1	1	1	1	1278	141.6	0.792037872
NP_653324.1	zinc finger matrin-type protein 2	ZMAT2	153527	1.365	4	1	3	1	199	23.6	0.080335746
NP_061918.3	WD repeat-containing protein 44 isoform 1	WDR44	54521	0.697	1	1	1	1	913	101.3	0.111122582
NP_055388.2	sigma intracellular receptor 2	TMEM97	27346	1.37	5	1	2	1	176	20.8	0.945503999
XP_005247590.1	phospholipase D1 isoform X1	PLD1	5337	0.802	2	1	1	1	1074	124.1	0.972087614
NP_001191335.1	arachidonate 5-lipoxygenase-activating protein isoform 2	ALOX5AP	241	0.544	6	1	1	1	218	24.2	0.904008151
XP_024307647.1	transmembrane protein 230 isoform X1	TMEM230	29058	1.192	6	1	1	1	190	20.3	0.467538873
NP_071409.3	phospholysine phosphohistidine inorganic pyrophosphate phosphatase isoform 1	LHPP	64077	0.448	8	1	2	1	270	29.1	0.060776297
NP_005836.2	multidrug resistance-associated protein 4 isoform 1	ABCC4	10257	1.332	1	1	1	1	1325	149.4	0.88570304
NP_001352253.1	non-canonical poly(A) RNA polymerase PAPD5 isoform d	TENT4B	64282	0.811	2	1	1	1	713	77.6	0.592064449
XP_016874252.1	serine dehydratase-like isoform X1	SDSL	113675	0.489	4	1	1	1	342	36.2	0.323553448
NP_000378.1	mitochondrial carnitine/acylcarnitine carrier protein	SLC25A20	788	1.119	3	1	3	1	301	32.9	0.111864334
XP_016876870.1	protein phosphatase 1A isoform X1	PPM1A	5494	0.908	3	1	1	1	459	51.1	0.946244023
NP_001037770.1	stromal membrane-associated protein 1 isoform A	SMAP1	60682	0.722	3	1	1	1	467	50.4	0.74387097
NP_060717.1	cell cycle control protein 50A isoform 1	TMEM30A	55754	1.199	4	1	1	1	361	40.7	0.884158287
XP_011515149.1	transmembrane protein 65 isoform X1	TMEM65	157378	0.64	4	1	2	1	264	28.2	0.081548245
NP_005350.1	mothers against decapentaplegic homolog 4	SMAD4	4089	0.773	4	1	1	1	552	60.4	0.204033378
XP_011542186.1	polyglutamine-binding protein 1 isoform X1	PQBP1	10084	1.277	3	1	2	1	265	30.5	0.856585092
NP_001265184.1	DNA polymerase epsilon subunit 3	POLE3	54107	0.856	7	1	2	1	147	16.8	0.712488806
NP_665857.2	quinone oxidoreductase-like protein 1	CRYZL1	9946	0.675	6	1	1	1	349	38.7	0.699791747
NP_997263.2	coiled-coil domain-containing protein 9B	CCDC9B	388115	0.822	1	1	1	1	534	57.3	0.741275293
XP_005259637.3	DNA-directed RNA polymerase, mitochondrial isoform X1	POLRMT	5442	0.425	2	1	2	1	1237	139.3	0.17210154
NP_056308.3	KN motif and ankyrin repeat domain-containing protein 2 isoform 1	KANK2	25959	0.91	1	1	2	1	859	91.9	0.6622476
XP_005251168.1	metastasis suppressor protein 1 isoform X1	MTSS1	9788	1.099	1	1	1	1	868	93.9	0.603669439
NP_660309.1	nudC domain-containing protein 2 isoform 1	NUDCD2	134492	0.773	7	1	2	1	157	17.7	0.732383434
NP_148977.2	E3 ubiquitin-protein ligase TRIM4 isoform alpha	TRIM4	89122	1.502	1	1	2	1	500	57.4	0.545012006
NP_001257935.1	centromere protein X isoform 1	CENPX	201254	0.682	11	1	2	1	81	9	0.960747494
XP_011530342.2	OTU domain-containing protein 4 isoform X1	OTUD4	54726	0.635	1	1	1	1	1121	124.8	0.53679734
XP_011538821.1	glucocorticoid modulatory element-binding protein 1 isoform X1	GMEB1	10691	1.13	2	1	1	1	573	62.6	0.905141291
XP_011530588.1	centrosomal protein of 44 kDa isoform X1	CEP44	80817	0.625	3	1	2	1	399	44.7	0.051444519
NP_001153861.1	tRNA 2'-phosphotransferase 1 isoform 3	TRPT1	83707	0.575	9	1	2	1	255	27.9	0.161746994
NP_061836.2	bromodomain and WD repeat-containing protein 1 isoform A	BRWD1	54014	1.451	1	1	5	1	2320	262.8	0.19476254
NP_037470.1	dolichyl-phosphate beta-glucosyltransferase isoform 1	ALG5	29880	1.354	4	1	1	1	324	36.9	0.826943869
NP_001103132.3	probable E3 ubiquitin-protein ligase HECTD4	HECTD4	283450	0.66	0	1	1	1	4284	469.7	0.343219731
XP_011536131.1	la-related protein 4 isoform X1	LARP4	113251	0.87	2	1	1	1	732	81.3	0.937091864
NP_003178.1	transcription initiation factor TFIID subunit 9	TAF9	6880	0.836	8	1	1	1	264	29	0.918471444
NP_001308715.1	E3 ubiquitin-protein ligase CBL-B isoform a	CBLB	868	0.686	2	1	2	1	1010	112.8	0.201259864
NP_001291744.1	protein capicua homolog isoform CIC-L	CIC	23152	1.15	1	1	1	1	2517	257.9	0.314150027
NP_620279.1	guanidinoacetate N-methyltransferase isoform b	GAMT	2593	0.863	8	1	1	1	269	29.4	0.383485356
XP_006714337.1	3-hydroxybutyrate dehydrogenase type 2 isoform X1	BDH2	56898	0.918	5	1	1	1	259	28.4	0.908632141
XP_005265987.1	BRCA1-A complex subunit RAP80 isoform X1	UIMC1	51720	0.892	3	1	2	1	719	79.7	0.399349599
XP_011533470.1	conserved oligomeric Golgi complex subunit 6 isoform X1	COG6	57511	0.901	1	1	1	1	702	78.3	0.646799781
NP_055099.1	neurochondrin isoform 1	NCDN	23154	0.644	2	1	1	1	729	78.8	0.642771768
NP_036221.2	ATP-binding cassette sub-family B member 10, mitochondrial	ABCB10	23456	0.965	2	1	1	1	738	79.1	0.579280969
NP_001341885.1	fibroblast growth factor 1 isoform 1 precursor	FGF1	2246	0.701	8	1	3	1	155	17.4	0.043524556
NP_079093.2	thrombospondin type-1 domain-containing protein 4 isoform 1 precursor	THSD4	79875	1.878	1	1	2	1	1018	112.4	0.976245464
XP_011521056.1	lipopolysaccharide-induced tumor necrosis factor-alpha factor isoform X1	LITAF	9516	1.359	4	1	3	1	191	20.4	0.116137824
XP_011532607.1	IQ motif and SEC7 domain-containing protein 1 isoform X2	IQSEC1	9922	1.187	1	1	1	1	1132	126.4	0.368832509
NP_060342.2	probable tRNA(His) guanylyltransferase isoform 1	THG1L	54974	0.813	4	1	3	1	298	34.8	0.138914632
NP_113676.2	lethal(3)malignant brain tumor-like protein 2	L3MBTL2	83746	1.34	1	1	3	1	705	79.1	0.008932075
NP_057594.5	selenocysteine lyase	SCLY	51540	0.92	2	1	1	1	445	48.1	0.214087031
NP_005328.2	nck-associated protein 1-like isoform 1	NCKAP1L	3071	0.545	1	1	2	1	1127	128.1	0.858625398
NP_001839.2	collagen alpha-1(VI) chain precursor	COL6A1	1291	1.704	1	1	3	1	1028	108.5	1.35E-05
NP_001171846.1	ethanolamine-phosphate cytidylyltransferase isoform 1	PCYT2	5833	0.153	5	1	1	1	407	45.6	0.378770701
NP_110389.1	class E basic helix-loop-helix protein 41	BHLHE41	79365	0.565	2	1	3	1	482	50.5	0.002084335
NP_001037850.1	transmembrane protein 237 isoform a	TMEM237	65062	1.199	4	1	1	1	408	45.5	0.611262051
XP_016873061.1	B-cell CLL/lymphoma 9-like protein isoform X1	BCL9L	283149	1.524	1	1	2	1	1547	162	0.546009196
NP_001265199.1	transcriptional adapter 3 isoform a	TADA3	10474	1.229	5	1	1	1	432	48.9	0.811943997
XP_016862062.1	testis-expressed protein 264 isoform X1	TEX264	51368	0.954	3	1	2	1	352	38.2	0.523969565
NP_036345.2	malonyl-CoA decarboxylase, mitochondrial	MLYCD	23417	0.847	3	1	1	1	493	55	0.345544721
XP_024304405.1	zinc finger protein ubi-d4 isoform X1	DPF2	5977	1.047	4	1	1	1	575	63.7	0.356249521
NP_061969.3	GTP-binding protein 2 isoform a	GTPBP2	54676	0.68	2	1	1	1	602	65.7	0.946445461
NP_291022.2	RCC1 domain-containing protein 1	RCCD1	91433	0.903	3	1	1	1	376	40.1	0.726207356
NP_054886.2	thyroid transcription factor 1-associated protein 26	CCDC59	29080	1.807	5	1	2	1	241	28.7	0.904900122
XP_024302862.1	exostosin-like 3 isoform X1	EXTL3	2137	1.01	2	1	2	1	919	104.7	0.693724333
XP_011517965.1	uncharacterized protein C10orf88 isoform X1	CCDC7	79741	1.289	1	1	1	1	1385	157.4	0.915971505
NP_001310487.1	protein EFR3 homolog A isoform 3	EFR3A	23167	1.558	1	1	3	1	838	94.8	0.041728402
NP_116231.2	transmembrane protein 209 isoform 1	TMEM209	84928	1.536	3	1	1	1	561	62.9	0.65903711
NP_056355.2	RNA polymerase II-associated protein 1	RPAP1	26015	0.797	1	1	2	1	1393	152.7	0.707899592
XP_011510816.1	protein ECT2 isoform X1	ECT2	1894	0.648	1	1	1	1	958	108.5	0.983062493
NP_009186.1	beta-1,4-galactosyltransferase 7	B4GALT7	11285	1.116	2	1	2	1	327	37.4	0.953718688
XP_011543287.1	ankyrin repeat domain-containing protein 13D isoform X1	ANKRD13D	338692	0.56	2	1	3	1	654	73.3	0.141408462
XP_005263764.1	protein IWS1 homolog isoform X1	IWS1	55677	0.981	2	1	1	1	824	92.4	0.699533786
XP_005256203.1	GATOR complex protein WDR59 isoform X1	WDR59	79726	0.59	1	1	1	1	993	112	0.066959023
NP_689657.1	prostaglandin reductase 2	PTGR2	145482	0.641	4	1	4	1	351	38.5	0.281835681
NP_001127911.1	pleckstrin homology-like domain family B member 2 isoform a	PHLDB2	90102	1.099	1	1	1	1	1253	142.1	0.96692884
XP_011531878.1	charged multivesicular body protein 2b isoform X1	CHMP2B	25978	0.754	4	1	3	1	229	25.6	0.00944415
XP_005256351.1	Fanconi anemia group A protein isoform X1	FANCA	2175	0.534	2	1	1	1	1463	163.3	0.951579261
XP_011519666.1	chondroitin sulfate synthase 1 isoform X1	CHSY1	22856	0.849	3	1	1	1	830	94.8	0.503954627
XP_016868510.1	vacuolar protein sorting-associated protein 37A isoform X1	VPS37A	137492	0.773	5	1	1	1	397	44.3	0.469986408
XP_011530104.1	cystine/glutamate transporter isoform X1	SLC7A11	23657	1.465	2	1	1	1	506	56	0.863179651
NP_001129071.1	transforming growth factor beta-2 isoform 1 precursor	TGFB2	7042	0.875	2	1	2	1	442	50.5	0.86307709
NP_001164653.1	centromere protein L isoform 2	CENPL	91687	1.341	3	1	1	1	344	39	0.301390806
XP_024310139.1	transmembrane protein 161B isoform X1	TMEM161B	153396	1.408	1	1	1	1	633	71.6	0.781320382
XP_005265644.1	actin-related protein 8 isoform X1	ACTR8	93973	0.729	3	1	2	1	624	70.4	0.122402348
NP_059107.1	survival motor neuron protein isoform d	SMN2	6607	0.799	3	1	1	1	294	31.8	0.931104307
NP_060552.4	DNA-directed RNA polymerase III subunit RPC2 isoform 1	POLR3B	55703	1.347	1	1	2	1	1133	127.7	0.987377834
XP_011541083.1	ester hydrolase C11orf54 isoform X1	C11orf54	28970	0.547	4	1	1	1	315	35.1	0.102669673
NP_001041675.1	chloride channel CLIC-like protein 1 isoform 1 precursor	CLCC1	23155	1.247	1	1	1	1	551	62	0.864605338
NP_001343945.1	lysosomal acid phosphatase isoform 1x precursor	ACP2	53	1.428	2	1	3	1	434	49.4	0.036457119
NP_071450.2	xylosyltransferase 2	XYLT2	64132	0.724	1	1	3	1	865	96.7	0.001407772
NP_001813.1	N-chimaerin isoform 1	CHN1	1123	0.544	2	1	2	1	459	53.1	0.426488288
NP_542172.2	beta-1,3-galactosyltransferase 6	B3GALT6	126792	1.151	3	1	3	1	329	37.1	0.511789819
NP_001012997.1	transcription elongation factor A protein-like 5	TCEAL5	340543	0.755	8	1	1	1	206	23.3	0.816520886
NP_055215.2	ly6/PLAUR domain-containing protein 3 precursor	LYPD3	27076	2.518	3	1	1	1	346	35.9	0.258088607
NP_060401.2	tetratricopeptide repeat protein 38	TTC38	55020	0.424	2	1	2	1	469	52.7	0.400108284
NP_647537.1	attractin isoform 1 preproprotein	ATRN	8455	1.557	1	1	1	1	1429	158.4	0.63341615
NP_219489.1	sorting nexin-21 isoform a	SNX21	90203	1.01	4	1	1	1	373	41.3	0.30994471
XP_011536363.1	homeobox protein cut-like 2 isoform X1	CUX2	23316	1.357	0	1	4	1	1542	168.1	0.000635097
NP_898880.1	vacuolar protein sorting-associated protein 28 homolog isoform 2	VPS28	51160	0.726	5	1	1	1	233	26.4	0.960266281
NP_055067.1	mitochondrial ornithine transporter 1	SLC25A15	10166	2.157	2	1	2	1	301	32.7	0.672903094
NP_001140170.1	probable RNA-binding protein 19	RBM19	9904	1.161	1	1	2	1	960	107.3	0.798583382
NP_001273510.1	PHD and RING finger domain-containing protein 1 isoform 1	PHRF1	57661	1.631	1	1	1	1	1649	178.6	0.629365114
NP_057114.5	trafficking protein particle complex subunit 12	TRAPPC12	51112	0.94	1	1	1	1	735	79.3	0.575452386
XP_011539331.1	WD repeat-containing protein 47 isoform X1	WDR47	22911	0.539	1	1	2	1	929	103.1	0.250961704
XP_005267136.1	BEN domain-containing protein 3 isoform X1	BEND3	57673	0.891	3	1	1	1	828	94.4	0.438233863
NP_001254.2	phosphatidate cytidylyltransferase 1	CDS1	1040	0.974	6	1	2	1	461	53.3	0.890883925
XP_024309015.1	probable cytosolic iron-sulfur protein assembly protein CIAO1 isoform X1	CIAO1	9391	0.616	6	1	2	1	372	41.5	0.172236889
NP_004459.2	four and a half LIM domains protein 3 isoform 1	FHL3	2275	1.282	4	1	2	1	280	31.2	0.970449408
NP_001289617.1	apolipoprotein E isoform a precursor	APOE	348	1.585	5	1	2	1	343	39	0.429690822
NP_065184.2	selenoprotein N isoform 2	SELENON	57190	1.578	1	1	3	1	590	65.8	0.270489152
NP_004272.2	BAG family molecular chaperone regulator 3	BAG3	9531	0.66	1	1	1	1	575	61.6	0.665630289
NP_003646.2	E3 SUMO-protein ligase CBX4	CBX4	8535	1.742	4	1	2	1	560	61.3	0.339341383
NP_004533.1	NADH dehydrogenase	NDUFA3	4696	1.177	42	1	3	1	84	9.3	0.030711359
NP_005940.1	metallothionein-1F isoform 1	MT1F	4494	0.14	21	1	2	1	61	6.1	0.1079307
NP_061139.2	tRNA (guanine-N(7)-)-methyltransferase non-catalytic subunit WDR4 isoform 1	WDR4	10785	0.871	3	1	3	1	412	45.5	0.37477863
NP_055778.2	protein strawberry notch homolog 2 isoform 1	SBNO2	22904	0.763	1	1	2	1	1366	150.2	0.080971686
XP_011526370.1	peptidyl-prolyl cis-trans isomerase NIMA-interacting 1 isoform X1	PIN1	5300	0.683	5	1	3	1	168	18.8	0.004635047
XP_011527964.1	1-acyl-sn-glycerol-3-phosphate acyltransferase gamma isoform X4	AGPAT3	56894	1.007	3	1	2	1	463	52.2	0.973581595
NP_003322.3	non-receptor tyrosine-protein kinase TYK2	TYK2	7297	1.254	1	1	3	1	1187	133.6	0.002080743
NP_001092907.1	zinc finger protein 30 isoform a	ZNF30	90075	1.431	5	1	2	1	624	71.4	0.991360617
NP_073741.2	speckle targeted PIP5K1A-regulated poly(A) polymerase	TUT1	64852	0.493	2	1	1	1	912	98.4	0.223789666
NP_001243711.1	septin-4 isoform 5	SEPT4	5414	1.087	2	1	3	1	493	56.6	0.048937616
XP_005258027.1	DNA ligase 3 isoform X1	LIG3	3980	0.77	1	1	2	1	1018	114	0.721844119
NP_899050.1	ras-related protein Rab-23	RAB23	51715	1.318	7	1	1	1	237	26.6	0.193753828
NP_001984.1	tissue factor isoform 1 precursor	F3	2152	1.518	7	1	3	1	295	33	0.00940669
NP_006570.1	3-beta-hydroxysteroid-Delta(8),Delta(7)-isomerase	EBP	10682	1.668	4	1	1	1	230	26.3	0.508889579
NP_006246.2	protein kinase C eta type	PRKCH	5583	0.541	2	1	1	1	683	77.8	0.787569369
NP_060628.2	kanadaptin	SLC4A1AP	22950	0.739	1	1	1	1	796	88.8	0.493056331
XP_011536103.1	bromodomain adjacent to zinc finger domain protein 2A isoform X1	BAZ2A	11176	0.843	0	1	1	1	1912	211.8	0.314882212
XP_016857675.1	transcription initiation factor TFIID subunit 12 isoform X2	TAF12	6883	1.321	3	1	1	1	241	27	0.553104391
XP_011536228.1	contactin-1 isoform X1	CNTN1	1272	0.975	1	1	1	1	1018	113.2	0.802813063
NP_001034230.1	TRIO and F-actin-binding protein isoform 6	TRIOBP	11078	0.958	0	1	1	1	2365	261.2	0.867877081
NP_006556.1	transcriptional repressor CTCF isoform 1	CTCF	10664	1.483	1	1	3	1	727	82.7	0.151163967
NP_001287783.1	dermcidin isoform 2 preproprotein	DCD	117159	3.384	7	1	1	1	121	12.4	0.800085447
XP_016868179.1	mitotic spindle assembly checkpoint protein MAD1 isoform X1	MAD1L1	8379	1.686	1	1	1	1	782	90.3	0.970222732
NP_620128.1	solute carrier family 25 member 46 isoform 1	SLC25A46	91137	1.042	3	1	1	1	418	46.1	0.765133156
NP_002817.2	sulfhydryl oxidase 1 isoform a precursor	QSOX1	5768	1.274	3	1	3	1	747	82.5	0.064952719
NP_115685.1	coiled-coil-helix-coiled-coil-helix domain-containing protein 5 isoform a	CHCHD5	84269	1.108	12	1	1	1	110	12.4	0.752622019
NP_001333746.1	thiopurine S-methyltransferase isoform 1	TPMT	7172	0.65	3	1	1	1	245	28.2	0.859191087
XP_011543439.1	serine/threonine-protein phosphatase 6 regulatory subunit 3 isoform X1	PPP6R3	55291	0.488	1	1	3	1	889	99.4	0.000160036
NP_008910.2	zinc transporter SLC39A7 isoform 1 precursor	SLC39A7	7922	2.667	3	1	3	1	469	50.1	6.45E-05
NP_001334.2	disabled homolog 2 isoform 1	DAB2	1601	1.201	1	1	2	1	770	82.4	0.806797821
XP_016880672.1	nuclear distribution protein nudE-like 1 isoform X1	NDEL1	81565	0.739	3	1	2	1	390	43.7	0.656424539
NP_065189.1	SPRY domain-containing protein 7 isoform 1	SPRYD7	57213	1.261	4	1	3	1	196	21.7	0.039304646
NP_078916.3	yrdC domain-containing protein, mitochondrial precursor	YRDC	79693	1.012	3	1	3	1	279	29.3	0.890368634
XP_016874923.1	periphilin-1 isoform X1	PPHLN1	51535	1.618	3	1	1	1	507	58.1	0.972621322
NP_523353.2	male-specific lethal 3 homolog isoform a	MSL3	10943	0.741	3	1	2	1	521	59.8	0.568544839
NP_690852.1	leucine-rich repeat and WD repeat-containing protein 1 isoform 1	LRWD1	222229	1.079	1	1	3	1	647	70.8	0.155041728
NP_065099.3	X-linked retinitis pigmentosa GTPase regulator-interacting protein 1	RPGRIP1	57096	0.16	1	1	2	1	1286	146.6	0.411705291
NP_112577.1	splicing factor 3B subunit 5	SF3B5	83443	0.922	10	1	1	1	86	10.1	0.815462775
NP_001337791.1	sorting nexin-13 isoform a	SNX13	23161	0.903	1	1	3	1	968	112.1	6.14E-05
XP_011535239.1	rho guanine nucleotide exchange factor 40 isoform X1	ARHGEF40	55701	0.801	0	1	2	1	1523	164.9	0.518222147
NP_001254479.2	titin isoform IC	TTN	7273	0.819	0	1	3	1	35991	3992.2	0.141594002
XP_005264306.1	CB1 cannabinoid receptor-interacting protein 1 isoform X1	CNRIP1	25927	1.065	14	1	2	1	164	18.6	0.923665342
NP_114110.1	FERM domain-containing protein 8 isoform 1	FRMD8	83786	1.307	3	1	1	1	464	51.2	0.620455094
NP_060368.2	mitochondrial amidoxime reducing component 2 isoform a precursor	MARC2	54996	2.091	2	1	3	1	335	38	0.406235063
NP_001243092.1	baculoviral IAP repeat-containing protein 2 isoform 1	BIRC2	329	0.775	1	1	1	1	618	69.9	0.481426787
XP_005251892.1	zinc finger and BTB domain-containing protein 43 isoform X1	ZBTB43	23099	1.023	3	1	2	1	467	52.6	0.425495056
XP_011513891.1	hyccin isoform X1	FAM126A	84668	0.775	4	1	2	1	521	57.6	0.014324951
NP_065145.2	charged multivesicular body protein 1b	CHMP1B	57132	0.455	3	1	3	1	199	22.1	0.006571309
NP_060549.4	S1 RNA-binding domain-containing protein 1	SRBD1	55133	0.991	1	1	3	1	995	111.7	0.851124401
NP_065111.1	N-acetyltransferase 14	NAT14	57106	0.929	4	1	3	1	206	21.6	0.777654993
NP_001305855.1	ribulose-phosphate 3-epimerase isoform 6	RPE	6120	0.568	3	1	3	1	246	26.9	0.010611691
NP_443091.1	protein RFT1 homolog	RFT1	91869	1.043	2	1	1	1	541	60.3	0.643487427
XP_005255770.1	rab11 family-interacting protein 3 isoform X1	RAB11FIP3	9727	4.111	1	1	1	1	801	87.5	0.748349425
XP_011541651.1	sorting nexin-24 isoform X1	SNX24	28966	0.353	4	1	2	1	246	28.8	0.059390001
NP_054907.1	RNA polymerase II subunit A C-terminal domain phosphatase SSU72	SSU72	29101	0.775	8	1	2	1	194	22.6	0.509794811
XP_005249902.1	uncharacterized protein C7orf26 isoform X1	C7orf26	79034	0.443	3	1	1	1	589	64.4	0.484327974
NP_004642.2	ubiquitin carboxyl-terminal hydrolase 11	USP11	8237	0.613	1	1	2	1	963	109.7	0.707151186
XP_016866640.1	costars family protein ABRACL isoform X1	ABRACL	58527	0.832	20	1	1	1	81	9.1	0.283634444
NP_002059.3	guanine nucleotide-binding protein subunit alpha-15	GNA15	2769	0.887	2	1	3	1	374	43.5	0.637298203
XP_005262461.1	HIV Tat-specific factor 1 isoform X1	HTATSF1	27336	1.378	1	1	4	1	755	85.8	0.743028781
XP_016862903.1	sorting nexin-4 isoform X1	SNX4	8723	0.711	1	1	3	1	482	55.9	0.017008661
XP_005254336.1	N-acetylgalactosamine kinase isoform X1	GALK2	2585	0.685	2	1	3	1	462	50.8	0.290470926
NP_001073590.1	major prion protein preproprotein Prp precursor	PRNP	5621	1.721	5	1	1	1	253	27.6	0.593395912
NP_001165694.1	MOB kinase activator 2 isoform 1	MOB2	81532	0.734	3	1	2	1	268	30.2	0.980293255
NP_001119594.1	mitochondrial thiamine pyrophosphate carrier	SLC25A19	60386	1.59	3	1	1	1	320	35.5	0.829872214
NP_000812.2	vitamin K-dependent gamma-carboxylase isoform 1	GGCX	2677	0.939	1	1	3	1	758	87.5	0.585148598
NP_473447.1	olfactory receptor 5AC2	OR5AC2	81050	0.578	2	1	3	1	309	35.3	0.023431649
XP_011531375.1	striatin isoform X1	STRN	6801	1.02	4	1	1	1	809	89.3	0.855026495
NP_001017989.2	optic atrophy 3 protein isoform a	OPA3	80207	0.362	4	1	3	1	180	20.2	0.000852721
NP_003497.2	frizzled-6 isoform a precursor	FZD6	8323	1.46	1	1	2	1	706	79.2	0.810663514
NP_055160.2	zinc finger protein 318	ZNF318	24149	1.093	1	1	1	1	2279	251	0.588961956
NP_127497.1	NACHT, LRR and PYD domains-containing protein 1 isoform 1	NLRP1	22861	0.838	1	1	1	1	1473	165.8	0.621024289
XP_024308593.1	bromodomain adjacent to zinc finger domain protein 2B isoform X1	BAZ2B	29994	0.649	1	1	2	1	2259	250.1	0.608965299
XP_006714567.1	ropporin-1-like protein isoform X1	ROPN1L	83853	1.695	4	1	1	1	257	29.1	0.742277118
XP_011541349.1	centrosomal protein of 295 kDa isoform X1	CEP295	85459	1.986	1	1	2	1	2645	300	0.559323903
NP_057386.2	protein cereblon isoform 1	CRBN	51185	0.742	5	1	1	1	442	50.5	0.464359709
NP_996925.1	alpha-endosulfine isoform 1	ENSA	2029	0.533	13	1	1	1	137	15.3	0.942459061
NP_631908.1	probable tRNA pseudouridine synthase 1	TRUB1	142940	1.077	6	1	2	1	349	37.2	0.983215973
XP_016878760.1	3-phosphoinositide-dependent protein kinase 1 isoform X1	PDPK1	5170	0.885	1	1	1	1	600	67.8	0.561377403
NP_542193.3	BRI3-binding protein precursor	BRI3BP	140707	1.739	3	1	3	1	251	27.8	0.018302568
XP_011508084.1	OTU domain-containing protein 7B isoform X1	OTUD7B	56957	1.005	2	1	1	1	852	93.4	0.89556502
NP_689895.2	RWD domain-containing protein 4 isoform 1	RWDD4	201965	0.647	5	1	2	1	188	21.2	0.495716772
NP_004995.1	NADH dehydrogenase	NDUFB8	4714	1.536	12	1	2	1	186	21.8	0.243317794
NP_689794.1	motile sperm domain-containing protein 2 isoform 1	MOSPD2	158747	1.355	2	1	1	1	518	59.7	0.77637715
NP_001350540.1	choline transporter-like protein 2 isoform 3	SLC44A2	57153	0.643	2	1	1	1	711	80.7	0.501762376

Patil et al,2020. Proteomic and phosphoproteomic profiling of shammah-induced signaling in oral keratinocytes

Supplementary Table S1: List of proteins identified and quantified in OKF6/TERT1-Shammah and OKF6/TERT1-Parental total proteome using TMT-based quantitative proteomic approach

Accession	Protein Description	Gene Symbol	Gene ID	Fold Change	Coverage [%]	# Peptides	# PSMs	# Unique Peptides	# AAs	MW [kDa]	p-value
NP_068602.2	twinkle protein, mitochondrial isoform A	TWNK	56652	0.766	4	1	1	1	684	77.1	0.166089032
NP_057306.2	probable ATP-dependent RNA helicase DDX41 isoform 1	DDX41	51428	1.378	1	1	3	1	622	69.8	0.002413197
NP_009133.2	A-kinase anchor protein 10, mitochondrial isoform 1 precursor	AKAP10	11216	0.367	2	1	1	1	662	73.8	0.935285513
NP_031390.1	transmembrane emp24 domain-containing protein 3 isoform a precursor	TMED3	23423	1.676	4	1	2	1	217	24.8	0.687249214
XP_006711514.1	histone-lysine N-methyltransferase ASH1L isoform X1	ASH1L	55870	0.128	0	1	2	1	2964	332	0.060278962
NP_699160.2	aldehyde dehydrogenase family 16 member A1 isoform 1	ALDH16A1	126133	0.484	1	1	1	1	802	85.1	0.761642603
XP_011518252.1	exostosin-2 isoform X1	EXT2	2132	1.101	1	1	1	1	764	87.3	0.488142529
NP_005253.3	FAD-linked sulfhydryl oxidase ALR	GFER	2671	1.518	7	1	1	1	205	23.4	0.963624482
NP_001309431.1	alpha-1A adrenergic receptor isoform 5	ADRA1A	148	0.477	2	1	3	1	372	40.6	0.000177668
NP_060672.2	macoilin isoform a	MACO1	55219	1.046	2	1	1	1	664	76.1	0.471229736
NP_061932.1	mitochondrial import receptor subunit TOM7 homolog	TOMM7	54543	0.976	31	1	1	1	55	6.2	0.812348226
NP_005406.3	sodium-dependent phosphate transporter 1	SLC20A1	6574	1.074	1	1	1	1	679	73.7	0.972900779
NP_001036068.1	lysine-specific demethylase 5A	KDM5A	5927	1.144	1	1	1	1	1690	192	0.378098093
XP_006719435.1	RILP-like protein 1 isoform X1	RILPL1	353116	0.605	2	1	2	1	411	47.9	0.56047649
XP_011510975.1	vesicle-trafficking protein SEC22a isoform X1	SEC22A	26984	1.548	4	1	1	1	307	34.9	0.371690374
NP_001138244.1	protein FAN isoform 2	NSMAF	8439	0.571	2	1	1	1	948	107.2	0.020065993
XP_005269064.1	kinesin-like protein KIF21A isoform X1	KIF21A	55605	0.437	1	1	1	1	1675	187.2	0.421537119
NP_004698.3	ubiquitin-like protein ATG12 isoform 1	ATG12	9140	0.525	5	1	2	1	140	15.1	0.702229459
NP_056230.1	mitochondrial pyruvate carrier 2	MPC2	25874	0.75	8	1	3	1	127	14.3	0.017865334
XP_005252892.1	NAD-dependent protein deacetylase sirtuin-3, mitochondrial isoform X1	SIRT3	23410	1.336	3	1	1	1	417	45.5	0.839358333
XP_016883228.1	nuclear receptor coactivator 6 isoform X1	NCOA6	23054	0.734	1	1	1	1	2087	221.8	0.553982773
NP_000779.1	deoxycytidine kinase	DCK	1633	0.524	5	1	1	1	260	30.5	0.766225443
NP_115515.2	ankyrin repeat domain-containing protein 27	ANKRD27	84079	0.897	1	1	3	1	1050	116.9	0.035236034
XP_005263981.1	uncharacterized protein KIAA1211-like isoform X2	KIAA1211L	343990	0.877	2	1	1	1	990	105.4	0.692341584
NP_057097.2	complex I intermediate-associated protein 30, mitochondrial precursor	NDUFAF1	51103	1.703	4	1	1	1	327	37.7	0.994096492
XP_011520054.2	uveal autoantigen with coiled-coil domains and ankyrin repeats isoform X1	UACA	55075	0.747	1	1	2	1	1436	164.9	0.343942335
XP_024308062.1	protein-tyrosine sulfotransferase 2 isoform X1	TPST2	8459	1.449	2	1	2	1	447	49.1	0.887068606
XP_011536420.1	single-strand selective monofunctional uracil DNA glycosylase isoform X1	SMUG1	23583	0.555	4	1	1	1	270	29.8	0.997769569
NP_065908.1	vacuolar protein sorting-associated protein 18 homolog	VPS18	57617	1.036	1	1	1	1	973	110.1	0.57737634
NP_001257870.1	janus kinase and microtubule-interacting protein 2 isoform 1	JAKMIP2	9832	1.509	1	1	2	1	820	96.2	0.827565145
NP_114143.1	TBC1 domain family member 10A isoform 2	TBC1D10A	83874	0.45	5	1	1	1	508	57.1	0.220758209
XP_011521472.1	protein BANP isoform X1	BANP	54971	1.548	2	1	1	1	536	58.5	0.911582648
NP_003835.3	tumor necrosis factor receptor superfamily member 10A	TNFRSF10A	8797	1.179	3	1	1	1	468	50.1	0.65850172
NP_443134.3	serine/threonine-protein kinase 11-interacting protein	STK11IP	114790	0.893	1	1	1	1	1088	120.2	0.882961388
NP_002373.3	protein max isoform a	MAX	4149	1.009	6	1	1	1	160	18.3	0.651795441
NP_001284694.1	centrin-3 isoform 1	CETN3	1070	1.015	4	1	1	1	191	22.4	0.942986923
NP_001164672.1	ubiquitin-associated protein 1 isoform 2	UBAP1	51271	0.726	4	1	1	1	566	61.8	0.344344789
XP_016868012.1	transmembrane protein 168 isoform X1	TMEM168	64418	1.144	2	1	1	1	697	79.7	0.560318833
NP_056075.1	paired amphipathic helix protein Sin3b isoform 1	SIN3B	23309	0.755	1	1	2	1	1162	133	0.710233387
NP_001124436.1	1-phosphatidylinositol 4,5-bisphosphate phosphodiesterase delta-1 isoform 1	PLCD1	5333	0.887	3	1	1	1	777	88.1	0.996290311
NP_056972.1	protein Hook homolog 1	HOOK1	51361	0.484	2	1	1	1	728	84.6	0.332944852
XP_024303153.1	serine/threonine-protein kinase Nek6 isoform X1	NEK6	10783	0.867	2	1	2	1	371	42	0.945001799
NP_001317183.1	SCY1-like protein 2 isoform 2	SCYL2	55681	0.541	1	1	3	1	933	104	0.002363455
NP_001164979.1	multiple coagulation factor deficiency protein 2 isoform A precursor	MCFD2	90411	1.422	12	1	1	1	146	16.4	0.734949225
NP_003657.1	golgin-45 isoform 1	BLZF1	8548	0.825	3	1	1	1	400	44.9	0.76272379
NP_001304986.1	complement factor I isoform 1 preproprotein	CFI	3426	0.979	2	1	2	1	591	66.6	0.89381385
NP_000285.1	phosphorylase b kinase gamma catalytic chain, liver/testis isoform isoform 1	PHKG2	5261	0.776	4	1	1	1	406	46.4	0.857542053
NP_009084.2	zinc finger protein 208 isoform 1	ZNF208	7757	1.197	2	1	1	1	1280	147.4	0.953099057
XP_006719498.1	PRKC apoptosis WT1 regulator protein isoform X1	PAWR	5074	0.501	3	1	2	1	340	36.5	0.788690831
NP_078959.3	transcription elongation factor, mitochondrial	TEFM	79736	1.002	2	1	2	1	360	41.7	0.9746556
XP_011516423.1	laminin subunit gamma-3 isoform X1	LAMC3	10319	1.352	1	1	2	1	1581	171.6	0.933438377
NP_001600.1	short/branched chain specific acyl-CoA dehydrogenase, mitochondrial isoform 1 precursor	ACADSB	36	0.655	5	1	1	1	432	47.5	0.051673998
NP_057146.1	mitotic spindle-associated MMXD complex subunit MIP18	CIAO2B	51647	0.595	4	1	3	1	163	17.7	0.003068569
NP_004996.1	NADH dehydrogenase	NDUFB9	4715	1.661	5	1	3	1	179	21.8	0.002775819
NP_001305713.1	inositol polyphosphate 5-phosphatase OCRL 1 isoform c	OCRL	4952	1.047	1	1	1	1	902	104.3	0.544868764
NP_001310832.1	RNA polymerase-associated protein LEO1 isoform 3	LEO1	123169	0.987	1	1	3	1	688	78.3	0.803388158
XP_016884746.1	H(+)/Cl(-) exchange transporter 5 isoform X1	CLCN5	1184	0.67	1	1	2	1	820	91.1	0.801689499
NP_001333181.1	ubiquitin carboxyl-terminal hydrolase 28 isoform c	USP28	57646	0.77	1	1	1	1	1139	129.2	0.028295195
XP_005247115.1	multidrug resistance-associated protein 5 isoform X1	ABCC5	10057	0.797	0	1	1	1	1437	160.6	0.715651688
NP_078970.3	androglobin	ADGB	79747	1.876	1	1	3	1	1667	189.6	1.07E-05
XP_005254312.1	dmX-like protein 2 isoform X1	DMXL2	23312	0.695	0	1	1	1	3058	341.9	0.962178045
XP_011511158.1	neprilysin isoform X1	MME	4311	0.564	1	1	1	1	750	85.5	0.791345963
NP_006042.3	amyloid-beta A4 precursor protein-binding family B member 3 isoform d	APBB3	10307	0.668	4	1	4	1	493	53.2	0.084206925
XP_011510207.1	lisH domain-containing protein ARMC9 isoform X1	ARMC9	80210	0.547	1	1	2	1	927	102.4	0.532088322
NP_054781.1	RNA-binding protein NOB1	NOB1	28987	0.644	2	1	1	1	412	46.6	0.774142652
XP_016867433.1	amiloride-sensitive amine oxidase	AOC1	26	0.496	3	1	2	1	770	87.2	0.013678186
XP_011532415.1	coiled-coil domain-containing protein 51 isoform X1	CCDC51	79714	1.058	3	1	1	1	431	48.1	0.775561913
XP_011528441.1	tRNA (uracil-5-)-methyltransferase homolog A isoform X1	TRMT2A	27037	1.318	1	1	2	1	643	70.8	0.764482959
NP_001034761.1	protein YIF1B isoform 5	YIF1B	90522	2.71	5	1	1	1	314	34.4	0.768030093
NP_001491.1	GDP-mannose 4,6 dehydratase isoform 1	GMDS	2762	0.857	5	1	1	1	372	41.9	0.749848589
NP_060293.2	dual specificity protein phosphatase 23	DUSP23	54935	0.448	5	1	1	1	150	16.6	0.311729129
XP_005273592.1	DNA polymerase beta isoform X1	POLB	5423	1.292	2	1	1	1	370	41.9	0.742521622
NP_062565.2	partitioning defective 3 homolog isoform 1	PARD3	56288	0.897	1	1	3	1	1356	151.3	0.193924772
NP_078954.4	probable asparagine--tRNA ligase, mitochondrial isoform 1 precursor	NARS2	79731	0.913	1	1	2	1	477	54.1	0.622494433
NP_057330.2	17-beta-hydroxysteroid dehydrogenase 14	HSD17B14	51171	1.068	11	1	3	1	270	28.3	0.526357239
NP_065207.2	polypeptide N-acetylgalactosaminyltransferase 1	GALNT1	2589	1.177	3	1	1	1	559	64.2	0.741183763
XP_005270242.2	tankyrase-2 isoform X1	TNKS2	80351	0.392	1	1	1	1	1190	129.6	0.079077504
XP_005252184.1	N-alpha-acetyltransferase 35, NatC auxiliary subunit isoform X1	NAA35	60560	0.847	1	1	1	1	725	83.6	0.922621897
NP_001077362.1	striatin-3 isoform 1	STRN3	29966	0.644	5	1	1	1	797	87.2	0.699807095
NP_003792.1	glycosylphosphatidylinositol anchor attachment 1 protein	GPAA1	8733	1.174	2	1	3	1	621	67.6	0.083720629
XP_006713861.1	beta-1,4-galactosyltransferase 4 isoform X1	B4GALT4	8702	1.595	2	1	2	1	344	40	0.826338042
NP_001405.1	translation initiation factor eIF-2B subunit alpha	EIF2B1	1967	0.553	2	1	1	1	305	33.7	0.421003279
NP_066011.2	protein GPR107 isoform 3	GPR107	57720	1.042	1	1	1	1	552	61.9	0.67003691
NP_061153.1	E3 ubiquitin-protein ligase RNF114	RNF114	55905	0.683	3	1	1	1	228	25.7	0.39019715
NP_001129204.1	target of Myb protein 1 isoform 2	TOM1	10043	1.016	5	1	3	1	493	53.8	0.566284875
NP_001528.1	heat shock factor-binding protein 1	HSBP1	3281	0.479	25	1	1	1	76	8.5	0.445932105
XP_011536780.1	histone deacetylase 7 isoform X1	HDAC7	51564	0.396	3	1	1	1	1035	111.3	0.884821431
NP_149015.2	protein piccolo isoform 1	PCLO	27445	0.393	0	1	5	1	5142	560.4	0.021142883
NP_001801.1	major centromere autoantigen B	CENPB	1059	1.422	1	1	3	1	599	65.1	0.000587977
XP_011511196.1	phosphatidylinositol 4,5-bisphosphate 3-kinase catalytic subunit alpha isoform isoform X1	PIK3CA	5290	0.594	1	1	2	1	1068	124.2	0.544788479
NP_056651.1	tryptophan--tRNA ligase, mitochondrial isoform 1 precursor	WARS2	10352	0.911	4	1	1	1	360	40.1	0.837949532
NP_001177984.1	endogenous retrovirus group V member 2 Env polyprotein precursor	ERVV-2	100271846	0.876	4	1	3	1	535	59.3	0.94713065
NP_002716.1	prolargin precursor	PRELP	5549	1.839	2	1	3	1	382	43.8	0.018623496
NP_006452.3	sperm-associated antigen 5	SPAG5	10615	0.759	1	1	1	1	1193	134.3	0.512254766
NP_002430.3	DNA mismatch repair protein Msh3	MSH3	4437	0.91	1	1	1	1	1137	127.3	0.995019102
XP_024302274.1	uncharacterized protein C1orf167 isoform X1	C1orf167	284498	1.315	1	1	2	1	1534	169.6	0.923314665
NP_005859.1	BET1 homolog isoform 1	BET1	10282	1.125	9	1	1	1	118	13.3	0.998948084
NP_001002755.1	NFU1 iron-sulfur cluster scaffold homolog, mitochondrial isoform 2	NFU1	27247	0.961	5	1	3	1	254	28.4	0.706229473
NP_612430.2	protein SAAL1	SAAL1	113174	0.445	2	1	1	1	474	53.5	0.462057894
NP_443108.1	nucleus accumbens-associated protein 1	NACC1	112939	0.994	1	1	1	1	527	57.2	0.844635234
XP_016863377.1	long-chain-fatty-acid--CoA ligase 1 isoform X2	ACSL1	2180	1.569	1	1	2	1	698	77.9	0.607059051
XP_016863522.1	zinc finger protein 330 isoform X1	ZNF330	27309	0.786	2	1	1	1	320	36.2	0.580481434
NP_000018.2	N(4)-(beta-N-acetylglucosaminyl)-L-asparaginase isoform 1 preproprotein	AGA	175	1.618	2	1	2	1	346	37.2	0.939156068
XP_006712758.1	ras-associated and pleckstrin homology domains-containing protein 1 isoform X1	RAPH1	65059	0.629	2	1	1	1	1302	141.1	0.999451428
NP_001129970.1	CDGSH iron-sulfur domain-containing protein 3, mitochondrial precursor	CISD3	284106	1.629	6	1	1	1	127	14.2	0.861461787
XP_016865452.1	G-protein coupled receptor 98 isoform X1	ADGRV1	84059	1.05	0	1	1	1	6313	693.4	0.187207517
NP_057329.3	estradiol 17-beta-dehydrogenase 11 precursor	HSD17B11	51170	0.799	5	1	2	1	300	32.9	0.128197974
XP_006716444.1	protein FAM160B2 isoform X1	FAM160B2	64760	0.58	3	1	1	1	753	83.4	0.517233924
NP_001337182.1	tRNA (guanine(37)-N1)-methyltransferase isoform 1	TRMT5	57570	1.258	1	1	2	1	537	61.3	0.954761648
XP_005263423.1	microfibrillar-associated protein 3-like isoform X1	MFAP3L	9848	1.003	1	1	1	1	442	49.2	0.484834538
XP_005253073.1	tetratricopeptide repeat protein 17 isoform X1	TTC17	55761	0.712	1	1	1	1	1198	135.9	0.406221185
XP_016857341.1	atypical kinase COQ8A, mitochondrial isoform X1	COQ8A	56997	1.071	1	1	2	1	647	71.9	0.930259798
XP_011509364.1	aldehyde oxidase isoform X1	AOX1	316	1.339	1	1	3	1	1350	149.1	0.042045314
NP_733832.1	ras association domain-containing protein 1 isoform D	RASSF1	11186	1.017	3	1	1	1	344	39.2	0.833337866
NP_001128522.1	protein turtle homolog A isoform a precursor	IGSF9	57549	4.89	2	1	2	1	1179	126.5	0.081893599
NP_006484.1	ceroid-lipofuscinosis neuronal protein 5	CLN5	1203	0.859	2	1	2	1	407	46.3	0.551477345
NP_001941.2	transcription factor E2F4	E2F4	1874	0.561	4	1	1	1	413	43.9	0.885844562
XP_016874718.1	sentrin-specific protease 1 isoform X1	SENP1	29843	1.144	1	1	1	1	707	79.9	0.794190051
NP_001310011.1	phytanoyl-CoA dioxygenase, peroxisomal isoform c precursor	PHYH	5264	1.377	3	1	1	1	340	38.7	0.820682365
NP_055446.2	SH3 and PX domain-containing protein 2A isoform 1	SH3PXD2A	9644	0.862	1	1	1	1	1105	121.8	0.994665295
XP_011535944.1	rho guanine nucleotide exchange factor 37 isoform X3	ARHGEF37	389337	0.575	3	1	1	1	686	77.6	0.960462304
XP_005271830.1	phosphoserine phosphatase isoform X1	PSPH	5723	0.638	5	1	1	1	225	25	0.753580478
NP_003096.1	sortilin-related receptor preproprotein	SORL1	6653	0.776	1	1	1	1	2214	248.3	0.043742948
XP_006716329.1	vinexin isoform X1	SORBS3	10174	1.03	1	1	1	1	710	79.5	0.563534289
XP_011539949.1	integrator complex subunit 11 isoform X1	INTS11	54973	0.545	2	1	1	1	660	73.7	0.723948546
NP_001339115.1	ankyrin repeat and sterile alpha motif domain-containing protein 1B isoform m	ANKS1B	56899	0.878	1	1	2	1	1285	142.1	0.510295085
NP_443164.2	porimin precursor	TMEM123	114908	1.422	4	1	2	1	208	21.5	0.970610971
NP_113615.2	dynein light chain 1, axonemal isoform 1	DNAL1	83544	0.823	11	1	1	1	190	21.5	0.959228305
XP_016873901.1	probable RNA-binding protein EIF1AD isoform X1	EIF1AD	84285	1.471	10	1	1	1	165	19	0.877598377
NP_001127956.1	mitochondrial import receptor subunit TOM5 homolog isoform 3	TOMM5	401505	1.504	8	1	2	1	92	10	0.79366521
XP_016882231.1	myb-related transcription factor, partner of profilin isoform X1	MYPOP	339344	0.754	4	1	1	1	542	57.4	0.499959925
XP_011536491.1	zinc finger protein GLI1 isoform X1	GLI1	2735	0.961	2	1	1	1	1106	117.8	0.375222778
XP_016883947.1	E3 ubiquitin-protein ligase TTC3 isoform X1	TTC3	7267	1.051	1	1	1	1	2081	236	0.899497472
NP_006234.1	serine/threonine-protein phosphatase 2A 56 kDa regulatory subunit alpha isoform isoform 1	PPP2R5A	5525	0.934	3	1	1	1	486	56.2	0.682159541
NP_060190.2	signal-transducing adaptor protein 2 isoform 1	STAP2	55620	0.719	3	1	1	1	449	49.6	0.643309584
NP_689947.2	bombesin receptor-activated protein C6orf89 isoform a	C6orf89	221477	1.112	2	1	1	1	354	40.7	0.904346586
NP_001273644.1	cytosolic carboxypeptidase 1 isoform a	AGTPBP1	23287	0.837	2	1	1	1	1278	143.1	0.953520608
NP_690848.1	collagen alpha-1(XXII) chain precursor	COL22A1	169044	1.065	1	1	1	1	1626	161	0.854548489
NP_758438.2	transcription factor AP-2-delta	TFAP2D	83741	0.905	2	1	1	1	452	49.5	0.912845334
NP_443107.1	vacuolar protein sorting-associated protein 26B	VPS26B	112936	1.073	3	1	1	1	336	39.1	0.916541597
NP_004612.2	short transient receptor potential channel 6	TRPC6	7225	0.948	1	1	3	1	931	106.3	0.087788335
NP_005438.2	ras association domain-containing protein 9	RASSF9	9182	0.849	2	1	2	1	435	50	0.700762888
NP_006235.1	serine/threonine-protein phosphatase 2A 56 kDa regulatory subunit beta isoform	PPP2R5B	5526	0.781	3	1	1	1	497	57.4	0.494079146
XP_005254215.2	methionyl-tRNA formyltransferase, mitochondrial isoform X1	MTFMT	123263	1.148	4	1	1	1	440	49.4	0.011141131
NP_945185.1	protein FAM111B isoform a	FAM111B	374393	0.65	3	1	1	1	734	84.6	0.170985421
XP_005255761.1	meiosis regulator and mRNA stability factor 1 isoform X1	MARF1	9665	1.322	1	1	1	1	1743	192.8	0.53172153
NP_001216.1	caspase-4 isoform alpha precursor	CASP4	837	1.023	3	1	1	1	377	43.2	0.95688272
XP_016881064.1	dystrobrevin alpha isoform X1	DTNA	1837	1.084	2	1	2	1	754	85.4	0.891427217
NP_075384.3	protein KRI1 homolog	KRI1	65095	1.137	1	1	1	1	709	83.2	0.924951397
NP_075383.2	jmjC domain-containing protein 4 isoform 1	JMJD4	65094	1.884	2	1	2	1	463	52.5	0.711389813
NP_064584.1	keratinocyte-associated transmembrane protein 2 precursor	C5orf15	56951	0.92	3	1	1	1	265	29.2	0.543341357
XP_016873254.1	menin isoform X1	MEN1	4221	1.419	2	1	1	1	657	72.9	0.857696571
XP_011531926.1	sulfatase-modifying factor 1 isoform X1	SUMF1	285362	0.559	4	1	1	1	448	49.4	0.841672331
NP_005111.2	mediator of RNA polymerase II transcription subunit 12	MED12	9968	1.17	0	1	1	1	2177	242.9	0.780593016
NP_055714.3	rho-related BTB domain-containing protein 3	RHOBTB3	22836	0.833	2	1	1	1	611	69.4	0.876650771
XP_011534951.1	ATP synthase subunit s, mitochondrial isoform X1	DMAC2L	27109	1.297	4	1	1	1	244	28.1	0.728076039
NP_064711.1	pyridoxal phosphate phosphatase	PDXP	57026	1.058	4	1	2	1	296	31.7	0.655112968
NP_001243758.1	transmembrane protein 265	TMEM265	100862671	1.476	23	1	2	1	108	11.8	0.944061114
XP_006719604.1	RNA-binding motif, single-stranded-interacting protein 2 isoform X1	RBMS2	5939	0.916	3	1	1	1	477	52.3	0.898537038
XP_005260253.1	zinc finger and BTB domain-containing protein 46 isoform X1	ZBTB46	140685	0.866	3	1	1	1	589	64	0.944912708
XP_011531035.1	follicle-stimulating hormone receptor isoform X1	FSHR	2492	0.873	1	1	2	1	729	81.9	0.707230809
NP_001007560.1	protein SSXT isoform 1	SS18	6760	1.297	3	1	1	1	418	45.9	0.882112322
XP_005262835.1	deoxycytidylate deaminase isoform X1	DCTD	1635	0.598	10	1	1	1	189	21.2	0.015871835
NP_001317652.1	sorting nexin-27 isoform 1	SNX27	81609	0.71	2	1	2	1	541	61.2	0.804009687
XP_005264324.1	echinoderm microtubule-associated protein-like 4 isoform X1	EML4	27436	0.682	2	1	1	1	992	110.1	0.025705333
NP_077295.1	multiple myeloma tumor-associated protein 2	C1orf35	79169	0.953	6	1	1	1	263	29.4	0.91917944
XP_005273268.1	signal-induced proliferation-associated 1-like protein 2 isoform X1	SIPA1L2	57568	1.205	2	1	1	1	1722	190.3	0.636340443
XP_011512453.1	ubiquitin thioesterase otulin isoform X1	OTULIN	90268	0.351	7	1	2	1	352	40.2	0.466026151
NP_066925.1	kunitz-type protease inhibitor 2 isoform a precursor	SPINT2	10653	0.859	4	1	1	1	252	28.2	0.928437804
XP_005261860.1	myotubularin-related protein 3 isoform X3	MTMR3	8897	0.615	1	1	2	1	1220	136	0.532066041
NP_001074012.1	cactin	CACTIN	58509	0.874	2	1	2	1	758	88.6	0.690829538
NP_001137434.1	calpain-8	CAPN8	388743	0.889	3	1	1	1	703	79.1	0.566587409
XP_016855687.1	forkhead-associated domain-containing protein 1 isoform X1	FHAD1	114827	3.405	1	1	1	1	1531	176.7	0.85830798
NP_057030.3	protein pelota homolog	PELO	53918	0.562	5	1	1	1	385	43.3	0.807152014
XP_005273996.1	O-acetyl-ADP-ribose deacetylase MACROD1 isoform X1	MACROD1	28992	1.63	3	1	3	1	334	36.4	0.000158223
XP_011536397.1	ATP-binding cassette sub-family B member 9 isoform X1	ABCB9	23457	1.577	2	1	1	1	766	84.4	0.950511068
NP_054747.2	osteopetrosis-associated transmembrane protein 1	OSTM1	28962	1.34	2	1	2	1	334	37.2	0.858677132
XP_005253361.1	FYVE, RhoGEF and PH domain-containing protein 4 isoform X1	FGD4	121512	0.989	1	1	1	1	930	103.7	0.790356906
NP_036228.1	DCC-interacting protein 13-alpha	APPL1	26060	0.475	1	1	3	1	709	79.6	0.000336359
NP_001307732.1	ORM1-like protein 3	ORMDL3	94103	1.107	7	1	2	1	153	17.5	0.887848676
NP_077740.1	desmocollin-2 isoform Dsc2a preproprotein	DSC2	1824	1.303	1	1	2	1	901	99.9	0.924147083
NP_872321.2	zinc finger protein 714	ZNF714	148206	2.873	2	1	2	1	555	64	0.839468643
XP_011518175.1	oxysterol-binding protein-related protein 5 isoform X1	OSBPL5	114879	0.824	1	1	1	1	879	98.6	0.970894743
XP_005253341.1	ATP-binding cassette sub-family C member 9 isoform X1	ABCC9	10060	0.971	0	1	2	1	1549	174.3	0.956002575
XP_005271379.1	nexilin isoform X1	NEXN	91624	0.852	1	1	1	1	676	80.8	0.813493289
NP_001193926.1	uncharacterized protein C4orf22 isoform 1	CFAP299	255119	1.221	6	1	1	1	250	28.8	0.118097508
XP_005251926.1	cell surface hyaluronidase isoform X1	CEMIP2	23670	0.813	1	1	2	1	1383	154.3	0.912638126
NP_110395.1	integrin-linked kinase-associated serine/threonine phosphatase 2C	ILKAP	80895	0.623	2	1	1	1	392	42.9	0.637245219
NP_942093.1	ankyrin repeat and EF-hand domain-containing protein 1 isoform 1	ANKEF1	63926	0.391	1	1	1	1	776	86.6	0.983461226
NP_002824.1	tyrosine-protein phosphatase non-receptor type 9	PTPN9	5780	0.559	1	1	2	1	593	68	0.805649931
NP_055752.1	phosphatidylinositide phosphatase SAC2 isoform 1	INPP5F	22876	0.831	2	1	1	1	1132	128.3	0.599992067
NP_620586.3	interleukin-31 receptor subunit alpha isoform 1	IL31RA	133396	0.529	3	1	1	1	764	86.4	0.785186193
NP_003479.1	A-kinase anchor protein 1, mitochondrial precursor	AKAP1	8165	1.344	2	1	3	1	903	97.3	0.000354094
XP_024304777.1	endoplasmic reticulum-Golgi intermediate compartment protein 2 isoform X1	ERGIC2	51290	0.941	6	1	1	1	377	42.5	0.747022831
NP_001093859.1	transmembrane protein 192	TMEM192	201931	1.202	4	1	2	1	271	30.9	0.978433234
NP_004243.1	Na(+)/H(+) exchange regulatory cofactor NHE-RF1	SLC9A3R1	9368	1.341	3	1	2	1	358	38.8	0.881695753
XP_011526902.1	cleavage stimulation factor subunit 1 isoform X1	CSTF1	1477	1.213	2	1	2	1	431	48.3	0.524921264
XP_011519299.1	serine/threonine-protein kinase WNK1 isoform X1	WNK1	65125	0.62	1	1	1	1	2795	295.2	0.662774726
NP_060937.1	vesicle transport protein USE1	USE1	55850	1.441	3	1	1	1	259	29.3	0.948489026
NP_115875.1	dynactin subunit 5 isoform 1	DCTN5	84516	0.583	4	1	3	1	182	20.1	0.001558907
XP_005262950.1	major facilitator superfamily domain-containing protein 8 isoform X1	MFSD8	256471	1.036	3	1	3	1	518	57.6	0.895864595
NP_005642.1	tryptophan 2,3-dioxygenase	TDO2	6999	0.642	2	1	3	1	406	47.8	0.153225175
NP_689480.2	E3 ubiquitin-protein ligase RNF185 isoform 1	RNF185	91445	1.03	5	1	1	1	192	20.4	0.764801515
XP_011544282.1	B-cell CLL/lymphoma 7 protein family member C isoform X1	BCL7C	9274	1.095	3	1	1	1	293	32.8	0.733089455
NP_036380.2	selenide, water dikinase 2	SEPHS2	22928	0.902	2	1	2	1	448	47.3	0.872285194
NP_001078868.1	RELT-like protein 1 precursor	RELL1	768211	1.161	3	1	2	1	271	29.3	0.93193658
NP_036537.1	protein-S-isoprenylcysteine O-methyltransferase	ICMT	23463	2.278	4	1	1	1	284	31.9	0.920369983
NP_569055.1	ATR-interacting protein isoform 1	ATRIP	84126	1.089	1	1	2	1	791	85.8	0.981247362
XP_005253915.1	histone-lysine N-methyltransferase SETD1B isoform X1	SETD1B	23067	0.875	1	1	1	1	1966	212.7	0.603115195
NP_001269220.1	ATP-binding cassette sub-family B member 8, mitochondrial isoform a	ABCB8	11194	0.585	1	1	1	1	735	79.9	0.653002724
XP_005256213.1	protein FAM192A isoform X1	FAM192A	80011	0.836	2	1	1	1	300	33.7	0.726697498
XP_005259549.1	PDZ domain-containing protein GIPC3 isoform X1	GIPC3	126326	1.082	1	1	1	1	1349	143.1	0.977702608
NP_002913.3	regulator of G-protein signaling 1	RGS1	5996	0.573	8	1	1	1	209	23.8	0.105881375
NP_001013764.1	diacylglycerol kinase kappa	DGKK	139189	0.973	1	1	2	1	1271	141.7	0.738321392
XP_011517313.1	terminal uridylyltransferase 7 isoform X1	TUT7	79670	0.94	1	1	3	1	1551	177.7	0.828789908
NP_066008.2	protocadherin beta-16 precursor	PCDHB16	57717	0.83	2	1	1	1	776	84.9	0.766649417
NP_060003.2	myosin-4	MYH4	4622	1.462	1	1	3	1	1939	222.9	0.017348782
XP_024304527.1	tyrosine-protein phosphatase non-receptor type 14 isoform X1	PTPN14	5784	0.653	1	1	2	1	1187	135.2	0.871257904
XP_005264814.1	contactin-3 isoform X1	CNTN3	5067	0.726	3	1	2	1	1028	112.8	0.970457735
NP_000775.1	sterol 26-hydroxylase, mitochondrial precursor	CYP27A1	1593	0.549	2	1	1	1	531	60.2	0.060818263
NP_060757.4	rho GTPase-activating protein 12 isoform 1	ARHGAP12	94134	0.999	1	1	1	1	846	96.2	0.83009611

Patil et al,2020. Proteomic and phosphoproteomic profiling of shammah-induced signaling in oral keratinocytes

Supplementary Table S2: List of phosphopeptides identified and quantified in OKF6/TERT1-Shammah and OKF6/TERT1-Parental phosphoproteome using TMT/TiO$_2$-based quantitative phosphoproteomic approach

Peptide Sequence	Protein Accessions	Gene Symbol	Gene ID	Positions in Proteins	Modifications	PhosphoRS: Best Site Probabilities	# PSMs	Fold change	p-value
[R].VPSSDEEVVEEPQSR.[R]	578821824	TNKS1BP1	85456	578821824 [1618-1632]	1xTMT6plex [N-Term];2xPhospho [S3(100);S4(100)]	S3(Phospho): 100;S4(Phospho): 100	7	0.283	0.049351
[K].GSSEQAESDNMDVPPEDDSK.[E]	212549553	ILF3	3609	212549553 [55-74]	1xOxidation [M11];1xPhospho [S8(99.9)];2xTMT6plex [N-Term;K20]	S8(Phospho): 99.91	3	0.595	0.048966
[R].KPVTVSPTTPTSPTEGEAS.[-]	166795297	DYNC1LI1	51143	166795297 [505-523]	1xPhospho [T/S];2xTMT6plex [N-Term;K1]	T9(Phospho): 19.95;T11(Phospho): 19.95;S12(Phospho): 19.95;T14(Phospho): 19.95;S19(Phospho): 19.95	16	2.378	0.047738
[R].GLGVGFGSGGGSSSSVK.[F]	119395754	KRT5	3852	119395754 [560-576]	1xPhospho [S];2xTMT6plex [N-Term;K17]	S15(Phospho): 99.99	24	0.503	0.047502
[R].TRSPSPTLGESLAPHK.[G]	767969840	PHLDB1	23187	767969840 [562-577]	2xPhospho [T1(98.6);2xTMT6plex [N-Term;K16];S5(100)]	T1(Phospho): 99.98;S5(Phospho): 99.98	12	0.508	0.047434
[R].AASDPLLSSVSPAVSK.[A]	8659574	TFE3	7030	8659574 [546-561]	2xPhospho [S3(100);2xTMT6plex [N-Term;K16];S11(90.5)]	S3(Phospho): 99.95;S8(Phospho): 33.35;S9(Phospho): 33.35;S11(Phospho): 33.35	2	0.636	0.046722
[R].RADDFPVRDDPSDVTDEDEGPAEPPPPPK.[L]	291219927	RABL6	55684	291219927 [586-614]	2xPhospho [S12(100);2xTMT6plex [N-Term;K29];T15(100)]	S12(Phospho): 100;T15(Phospho): 100	3	0.409	0.045796
[R].ATSSSSGSLSATGR.[L]	388240801	LMNB2	84823	388240801 [417-430]	1xTMT6plex [N-Term];2xPhospho [S4(99);S/T]	S4(Phospho): 98.97;S6(Phospho): 50;S8(Phospho): 50	8	4.259	0.045611
[K].NAEEESESEAEEGD.[-]	333470719	BZW1	9689	333470719 [438-451]	1xTMT6plex [N-Term];2xPhospho [S6(100);S8(100)]	S6(Phospho): 100;S8(Phospho): 100	8	0.491	0.045149
[R].ASVSPMDEPVPDSESPIEK.[V]	153945728	MAP1B	4131	153945728 [1375-1393]	1xOxidation [M6];2xPhospho [S15(97.2);2xTMT6plex [N-Term;K19];S]	S4(Phospho): 98.35;S15(Phospho): 99.97	6	0.381	0.044867
[R].VGDSTPVSEKPVSAAVDANASESP.[-]	29826294	GORASP2	26003	29826294 [429-452]	1xPhospho [S23(93.5)];2xTMT6plex [N-Term;K10]	S23(Phospho): 96.83	3	1.42	0.043671
[K].TPVQYSQQQNSPQK.[H]	103472005	MKI67	4288	103472005 [347-360]	1xPhospho [S11(100)];2xTMT6plex [N-Term;K14]	S11(Phospho): 100	6	0.491	0.043161
[R].KAVPMAPAPASPGSSNDSSAR.[S]	1034608385	MAP1S	55201	1034608385 [749-769]	1xOxidation [M5];1xPhospho [S11(96.9)];2xTMT6plex [N-Term;K1]	S11(Phospho): 96.88	4	0.748	0.042195
[K].LDQPVSAPPSPR.[D]	46852390	CCDC6	8030	46852390 [235-246]	1xTMT6plex [N-Term];2xPhospho [S6(100);S10(100)]	S6(Phospho): 100;S10(Phospho): 100	17	0.604	0.042033
[R].AGSSTPGDAPPAVAEVQGR.[S]	1034673276	HUWE1	10075	1034673276 [3012-3030]	1xTMT6plex [N-Term];2xPhospho [S3(98.8);S/T]	S3(Phospho): 98.8;S4(Phospho): 50.6;T5(Phospho): 50.6	2	0.736	0.041255
[K].EGMNPSYDEYADSDEDQHDAYLER.[M]	1370459980	SSRP1	6749	1370459980 [661-684]	1xOxidation [M3];1xPhospho [S/Y];1xTMT6plex [N-Term]	S6(Phospho): 33.08;Y10(Phospho): 33.08;S13(Phospho): 33.08	9	0.769	0.041014
[R].GLNTSQESDDDILDESSSPEGTQK.[V]	1034557776	ARHGEF16	27237	1034557776 [240-263]	1xPhospho [S/T];2xTMT6plex [N-Term;K24]	S8(Phospho): 99.95	9	0.463	0.040596
[K].SQDADSPGSSGAPENLTFK.[E]	1034650321	AFDN	4301	1034650321 [1782-1800]	1xPhospho [S6(97.5)];2xTMT6plex [N-Term;K19]	S6(Phospho): 99.98	7	0.643	0.040553
[K].FQSQADQDQQASGLQSPPSR.[D]	767905135	CTTNBP2NL	55917	767905135 [466-485]	1xPhospho [S];1xTMT6plex [N-Term]	S19(Phospho): 96.62	3	0.533	0.040015
[R].RGSGDTSSLIDPDTSLSELR.[DE]	767924540	LRRFIP2	9209	767924540 [326-345]	1xPhospho [S3(100)];1xTMT6plex [N-Term]	S3(Phospho): 100	5	0.536	0.039596
[R].RYSPPIQR.[R]	1034554585	SRRM1	10250	1034554585 [634-641]	1xPhospho [Y/S];1xTMT6plex [N-Term]	Y2(Phospho): 50;S3(Phospho): 50	6	1.761	0.038437
[R].ENPPVEDSSDEDDKR.[N]	30578410	STT3B	201595	30578410 [491-505]	2xPhospho [S8(100);2xTMT6plex [N-Term;K14];S9(100)]	S8(Phospho): 100;S9(Phospho): 100	3	1.543	0.037960
[R].LLHEDLDESDDDMDEK.[L]	767913370	ASAP2	8853	767913370 [707-722]	1xOxidation [M13];1xPhospho [S9(100)];2xTMT6plex [N-Term;K16]	S9(Phospho): 100	3	0.388	0.037952
[K].EPTPSIASDISLPIATQELR.[Q]	1370510645	ESYT2	57488	1370510645 [744-763]	1xTMT6plex [N-Term];2xPhospho [S8(87.4);S11(87.4)]	S8(Phospho): 99.76;S11(Phospho): 99.76	3	0.683	0.037552
[R].SQSSDTEQQSPTSGGGK.[V]	664805999	EIF4B	1975	664805999 [500-516]	1xPhospho [S/T];2xTMT6plex [N-Term;K17]	S4(Phospho): 99.08	13	0.357	0.037389
[K].TASESISNLSEAGSIK.[K]	530401713	CLIP1	6249	530401713 [191-206]	2xPhospho [S10(100);2xTMT6plex [N-Term;K16];S14(100)]	S10(Phospho): 100;S14(Phospho): 100	3	0.296	0.037374
[K].YSPTSPTYSPTSPK.[YG]	4505939	POLR2A	5430	4505939 [1909-1922];[1923-1936]	2xPhospho [T/S/Y];2xTMT6plex [N-Term;K14]	T4(Phospho): 49.26;S5(Phospho): 99.18;T7(Phospho): 49.26	15	1.278	0.037282
[R].SQSGEDESMNQPGPIK.[T]	4505725	PEX1	5189	4505725 [1209-1224]	1xOxidation [M9];1xPhospho [S3(100)];2xTMT6plex [N-Term;K16]	S3(Phospho): 99.99	6	1.635	0.037222
[R].SGSAAQAEGLCK.[Q]	5031953	ALG3	10195	5031953 [11-22]	1xCarbamidomethyl [C11];1xPhospho [S];2xTMT6plex [N-Term;K12]	S3(Phospho): 99.44	6	1.461	0.036928
[R].RASPSPPPK.[R]	1034554585	SRRM1	10250	1034554585 [663-671]	2xPhospho [S3(100);2xTMT6plex [N-Term;K9];S5(100)]	S3(Phospho): 100;S5(Phospho): 100	3	1.595	0.036702
[R].RLSQPESAEK.[H]	7662238	ACIN1	22985	7662238 [708-717]	1xPhospho [S3(100)];2xTMT6plex [N-Term;K10]	S3(Phospho): 100	4	2.311	0.036404
[R].GSLASLDSLR.[K]	146231940	CTNND1	1500	146231940 [345-354]	1xTMT6plex [N-Term];2xPhospho [S5(100);S8(100)]	S5(Phospho): 100;S8(Phospho): 100	8	1.649	0.036376
[K].AAAYDISEDEED.[-]	916413046	PCYT1A	5130	916413046 [356-367]	1xPhospho [S7(100)];1xTMT6plex [N-Term]	S7(Phospho): 100	3	0.552	0.036285
[R].YSHSYLSDSDTEAK.[L]	1093953582	MYO18A	399687	1093953582 [2039-2052]	2xPhospho [Y/S/T];2xTMT6plex [N-Term;K14]	Y5(Phospho): 66.66;S7(Phospho): 66.66;S9(Phospho): 66.66	7	0.513	0.035568
[K].AQTPPGPSLSGSK.[S]	118572613	SRRM2	23524	118572613 [1001-1013]	1xPhospho [T3(100)];2xTMT6plex [N-Term;K13]	T3(Phospho): 100	6	1.794	0.034833
[K].LGAGEGGEASVSPEKTSTTSK.[G]	530380665	TCOF1	6949	530380665 [1405-1425]	2xPhospho [S10(100);3xTMT6plex [N-Term;K15;K21];S/T]	S10(Phospho): 100;S12(Phospho): 100	7	0.573	0.034829
[R].TCEERPAEDGSDEEDPDSMEAPTR.[I]	38327642	WDR55	54853	38327642 [4-27]	1xCarbamidomethyl [C2];1xOxidation [M19];1xPhospho [S11(100)];1xTMT6plex [N-Term]	S11(Phospho): 100	3	0.509	0.034402
[R].SAVRPASLNLNR.[S]	767906494	DOCK7	85440	767906494 [882-893]	1xPhospho [S7(100)];1xTMT6plex [N-Term]	S7(Phospho): 100	3	0.56	0.034179
[R].RVNDAEPGSPEAPQGK.[R]	154759257	DDX51	317781	154759257 [75-90]	1xPhospho [S9(100)];2xTMT6plex [N-Term;K16]	S9(Phospho): 100	3	1.29	0.033753
[R].TLENSPISSCDTSDTEGPVPVNSAAVLK.[K]	767929330	AFAP1	60312	767929330 [759-786]	1xCarbamidomethyl [C10];2xPhospho [S/T];2xTMT6plex [N-Term;K28]	S5(Phospho): 94.06;S8(Phospho): 21.16;S9(Phospho): 21.16;T12(Phospho): 21.16;S13(Phospho): 21.16;T15(Phospho): 21.16	4	1.902	0.033585
[R].AIGGGLSSVGGGSSTIK.[Y]	5031839	KRT6A	3853	5031839 [534-550]	1xPhospho [S/T];2xTMT6plex [N-Term;K17]	S7(Phospho): 50;S8(Phospho): 50	13	2.225	0.033053
[R].ENPPVEDSSDEDDKR.[N]	30578410	STT3B	201595	30578410 [491-505]	1xPhospho [S8(99.3)];2xTMT6plex [N-Term;K14]	S8(Phospho): 99.34	6	1.265	0.032968
[R].EVDQDDEENSEEDEMDSGTMVR.[A]	530418351	STK4	6789	530418351 [327-348]	1xPhospho [S10(100)];1xTMT6plex [N-Term];2xOxidation [M15;M20]	S10(Phospho): 99.95	4	0.537	0.032890
[K].ALELPLAASSIPRPRTPSPESHR.[S]	1034608385	MAP1S	55201	1034608385 [623-645]	1xTMT6plex [N-Term];2xPhospho [S9(94.2);S18(91.5)]	S9(Phospho): 94.21;S18(Phospho): 91.46	2	0.44	0.031881
[R].YFQISQDEDESESED.[-]	153791384	RPL22L1	200916	153791384 [108-122]	1xPhospho [S/Y];1xTMT6plex [N-Term]	S11(Phospho): 49.99;S13(Phospho): 49.99	27	0.24	0.031544
[R].SESAPTLHPYSPLSPK.[G]	215820635	PPP1R13L	10848	215820635 [100-115]	2xPhospho [S14(99.9);2xTMT6plex [N-Term;K16];S/Y/T]	S3(Phospho): 97.44;S14(Phospho): 99.98	5	0.374	0.031155
[R].SLSNSNPDISGTPTSPDDEVR.[S]	767906494	DOCK7	85440	767906494 [896-916]	1xPhospho [S5(99.9)];1xTMT6plex [N-Term]	S5(Phospho): 99.98	6	0.609	0.030894
[R].ETPHSPGVEDAPIAK.[V]	767974553	LIMA1	51474	767974553 [487-501]	1xPhospho [S5(100)];2xTMT6plex [N-Term;K15]	S5(Phospho): 100	13	1.607	0.030512
[K].AGMSSNQSISSPVLDAVPR.[T]	118572613	SRRM2	23524	118572613 [1394-1412]	1xOxidation [M3];1xPhospho [S];1xTMT6plex [N-Term]	S10(Phospho): 49.61;S11(Phospho): 49.61	12	1.611	0.030010
[K].TAPSSPLTSPSDTR.[S]	38679909	TEX2	55852	38679909 [262-275]	1xTMT6plex [N-Term];2xPhospho [S/T]	T8(Phospho): 98.31;S9(Phospho): 98.31	9	2.198	0.029682
[K].IDASKNEEDEGHSNSSPR.[H]	14110420	HNRNPD	3184	14110420 [68-85]	2xPhospho [S15(97.8);2xTMT6plex [N-Term;K5];S16(97.8)]	S13(Phospho): 66.67;S15(Phospho): 66.67;S16(Phospho): 66.67	9	0.728	0.029669
[K].DYLLSESEDEGDNDGER.[K]	21361348	UTP14A	10813	21361348 [25-41]	1xTMT6plex [N-Term];2xPhospho [S5(100);S7(100)]	S5(Phospho): 100;S7(Phospho): 100	7	0.645	0.028991
[K].ASAPERTPSPAPK.[R]	23199998	NSUN5	55695	23199998 [436-448]	2xPhospho [T7(100);2xTMT6plex [N-Term;K13];S9(100)]	T7(Phospho): 100;S9(Phospho): 100	8	0.66	0.028821
[K].SASSYSDIEEIATPDSSAPSSPK.[L]	1370455125	NAV1	89796	1370455125 [1256-1278]	2xPhospho [T/S/Y];2xTMT6plex [N-Term;K23]	S20(Phospho): 94.52;S21(Phospho): 99.74	3	0.342	0.028306
[K].VQISPDSGGLPER.[S]	530425477	KHSRP	8570	530425477 [178-190]	1xPhospho [S4(100)];1xTMT6plex [N-Term]	S4(Phospho): 99.98	3	1.65	0.027694
[R].ADRLEGDRSEGSGQENENEDEE.[-]	312176366	DCAF6	55827	312176366 [930-951]	1xTMT6plex [N-Term];2xPhospho [S9(100);S12(100)]	S9(Phospho): 100;S12(Phospho): 100	3	0.652	0.027415
[R].TLSPSSGYSSQSGTPTLPPK.[G]	112734870	KIAA1522	57648	112734870 [577-596]	2xPhospho [S/T/Y];2xTMT6plex [N-Term;K20]	S9(Phospho): 36.1;S10(Phospho): 36.1;S12(Phospho): 36.1;T14(Phospho): 36.1;T16(Phospho): 36.1	3	0.793	0.027175
[R].SASVSPTRNSSNTLPIPK.[K]	119829187	COL17A1	1308	119829187 [170-187]	2xPhospho [T13(95.5);2xTMT6plex [N-Term;K18];S/T]	S5(Phospho): 95.88;S10(Phospho): 49.46;T13(Phospho): 49.46	7	2.213	0.026296
[K].SQDVAVSPQQQQCSK.[S]	1190332452	EDC3	80153	1190332452 [125-139]	1xCarbamidomethyl [C13];1xPhospho [S7(98.3)];2xTMT6plex [N-Term;K15]	S7(Phospho): 100	6	0.392	0.025436
[R].TPEELDDSDFETEDFDVRSR.[T]	1022433203	CTNNA1	1495	1022433203 [634-653]	1xTMT6plex [N-Term];2xPhospho [S19(81.2);S/T]	S8(Phospho): 99.95;S19(Phospho): 100	3	3.517	0.024908
[K].SPGALETPSAAGSQGNTASQGK.[E]	6005709	PALM2-AKAP2	445815	6005709 [624-645]	1xPhospho [S1(100)];2xTMT6plex [N-Term;K22]	S1(Phospho): 100	3	0.799	0.024888
[K].KLEKEEEEGISQESSEEEQ.[-]	22208967	HMGA1	3159	22208967 [89-107]	3xPhospho [S11(100);3xTMT6plex [N-Term;K1;K4];S14(100);S15(100)]	S11(Phospho): 100;S14(Phospho): 100;S15(Phospho): 100	3	0.314	0.023669
[R].GSPQQIDHAK.[Q]	530425477	KHSRP	8570	530425477 [479-488]	1xPhospho [S2(100)];2xTMT6plex [N-Term;K10]	S2(Phospho): 100	3	1.489	0.023579
[K].ALELPLAASSIPRPRTPSPESHRSPAEGSER.[L]	1034608385	MAP1S	55201	1034608385 [623-653]	1xTMT6plex [N-Term];3xPhospho [S/T]	S9(Phospho): 69;S10(Phospho): 69;T16(Phospho): 69;S18(Phospho): 72.96	2	0.412	0.023131
[K].MNEEISSDSESESLAPR.[K]	4759276	RRP9	9136	4759276 [45-61]	1xOxidation [M1];1xTMT6plex [N-Term];3xPhospho [S6(100);S]	S6(Phospho): 99.98;S7(Phospho): 98.7;S9(Phospho): 98.7	8	0.446	0.022312
[K].DGDSYDPYDFSDTEEEMPQVHTPK.[T]	394582093	MCM3	4172	394582093 [746-769]	1xOxidation [M17];2xPhospho [T22(99.8);2xTMT6plex [N-Term;K24];Y/S/T]	S11(Phospho): 49.43;T13(Phospho): 49.43;T22(Phospho): 100	7	0.449	0.022263
[K].VENMSSNQDGNDSDEFM.[-]	108936950	CCDC25	55246	108936950 [192-208]	1xPhospho [S13(97.1)];1xTMT6plex [N-Term];2xOxidation [M4;M17]	S5(Phospho): 33.33;S6(Phospho): 33.33;S13(Phospho): 33.33	15	0.294	0.022101
[R].AGAGMITQHSSNASPINR.[I]	767985304	SLTM	79811	767985304 [1131-1148]	1xOxidation [M5];1xPhospho [S14(100)];1xTMT6plex [N-Term]	S14(Phospho): 100	8	1.621	0.021991
[R].SFSLASSSNSPISQR.[R]	19923592	OSBPL11	114885	19923592 [172-186]	1xPhospho [S];1xTMT6plex [N-Term]	S10(Phospho): 99.99	3	0.52	0.021920
[K].LGVSVSPSR.[A]	661567300	ZC3H18	124245	661567300 [553-561]	1xTMT6plex [N-Term];2xPhospho [S4(100);S6(100)]	S4(Phospho): 100;S6(Phospho): 98.6	5	1.276	0.021399
[R].VAAAAGSGPSPPGSPGHDR.[E]	13559514	PI4K2A	55361	13559514 [38-56]	1xTMT6plex [N-Term];2xPhospho [S10(100);S14(100)]	S10(Phospho): 99.95;S14(Phospho): 100	10	1.167	0.021326
[R].LAESHVESASNMEQLTR.[E]	157419138	LAMC2	3918	157419138 [764-780]	1xOxidation [M12];1xPhospho [S/T];1xTMT6plex [N-Term]	S8(Phospho): 49.97;S10(Phospho): 49.97	7	1.394	0.020770
[K].KPATPAEDDEDDDIDLFGSDNEEEDKEAAQLR.[E]	1370511980	EEF1D	1936	1370511980 [510-541]	1xPhospho [S19(99.8)];3xTMT6plex [N-Term;K1;K26]	S19(Phospho): 100	3	0.179	0.020646
[R].RTRSPSPTLGESLAPHK.[G]	767969840	PHLDB1	23187	767969840 [561-577]	2xPhospho [T2(100);2xTMT6plex [N-Term;K17];S/T]	T2(Phospho): 99.98;S6(Phospho): 98.78	10	0.313	0.020219
[R].LAIQGPEDSPSRQSR.[R]	4885487	MLF2	8079	4885487 [230-244]	1xTMT6plex [N-Term];2xPhospho [S9(98.8);S]	S9(Phospho): 98.72;S14(Phospho): 99.98	9	5.278	0.019821
[R].SNTIQSGPEGSLVNSQSLKSIPEK.[F]	19923729	LRRC8C	84230	19923729 [196-219]	2xPhospho [S/T];3xTMT6plex [N-Term;K19;K24]	S15(Phospho): 65.84;S17(Phospho): 65.84;S20(Phospho): 65.84	7	1.776	0.019527
[R].SCFESSPDPELK.[S]	118572613	SRRM2	23524	118572613 [871-882]	1xCarbamidomethyl [C2];1xPhospho [S];2xTMT6plex [N-Term;K12]	S5(Phospho): 50;S6(Phospho): 50	6	1.19	0.019467
[K].ESLKEEDESDDDNM.[-]	4506183	PSMA3	5684	4506183 [242-255]	1xOxidation [M14];1xPhospho [S9(100)];2xTMT6plex [N-Term;K4]	S9(Phospho): 100	6	0.62	0.019415
[R].KVVDYSQFQESDDADEDYGR.[D]	56118310	NUCKS1	64710	56118310 [9-28]	1xPhospho [S11(99.9)];2xTMT6plex [N-Term;K1]	S11(Phospho): 100	10	0.402	0.019288
[R].SECDSSPEPK.[A]	118572613	SRRM2	23524	118572613 [1478-1487]	1xCarbamidomethyl [C3];2xPhospho [S5(100);2xTMT6plex [N-Term;K10];S6(100)]	S5(Phospho): 100;S6(Phospho): 100	4	2.14	0.019201
[R].RLEISPDSSPER.[A]	148664226	PKP2	5318	148664226 [147-158]	1xTMT6plex [N-Term];2xPhospho [S5(100);S9(100)]	S5(Phospho): 100;S8(Phospho): 50;S9(Phospho): 50	8	0.613	0.018297
[K].EGHSLEMENENLVENGADSDEDDNSFLK.[Q]	767974553	LIMA1	51474	767974553 [669-696]	1xOxidation [M7];1xPhospho [S19(100)];2xTMT6plex [N-Term;K28]	S19(Phospho): 99.96	7	0.577	0.018015
[R].IPESETESTASAPNSPR.[T]	15529996	SOS1	6654	15529996 [1068-1084]	1xTMT6plex [N-Term];2xPhospho [S11(99.9);S15(100)]	S11(Phospho): 100;S15(Phospho): 100	3	0.663	0.017597
[K].TTSQAHSLPLSPASTR.[Q]	1370486990	NFKB1	4790	1370486990 [906-921]	1xTMT6plex [N-Term];2xPhospho [S7(99.6);S11(93.7)]	S7(Phospho): 93.44;S11(Phospho): 95.51	3	0.755	0.017489
[K].TVIRLPSGSGAASPTGSAVDIR.[A]	1079280806	AHNAK	79026	1079280806 [204-225]	1xTMT6plex [N-Term];2xPhospho [S7(96.4);S13(96.1)]	S7(Phospho): 100;S13(Phospho): 99.96	8	2.943	0.017234
[K].LVEQANSPK.[H]	1406928079	SLU7	10569	1406928079 [224-232]	1xPhospho [S7(100)];2xTMT6plex [N-Term;K9]	S7(Phospho): 100	3	0.645	0.017178
[R].TLSNAEDYLDDEDSD.[-]	578817327	OSTF1	26578	578817327 [203-217]	1xPhospho [Y/S/T];1xTMT6plex [N-Term]	S14(Phospho): 100	7	0.217	0.016994
[R].GPGSPPRTPPAPGPPSFEEQLR.[L]	767968057	ANKRD13D	338692	767968057 [598-619]	1xTMT6plex [N-Term];2xPhospho [S4(100);T8(100)]	S4(Phospho): 100;T8(Phospho): 100	3	0.684	0.016962
[R].GRNDSGEENVPLDLTR.[E]	550544217	RICTOR	253260	550544217 [17-32]	1xPhospho [S5(100)];1xTMT6plex [N-Term]	S5(Phospho): 100	3	0.64	0.016635
[R].GEPNVSYICSR.[Y]	49574532	GSK3A	2931	49574532 [273-283]	1xCarbamidomethyl [C9];1xPhospho [S6(97.8)];1xTMT6plex [N-Term]	S6(Phospho): 97.82	5	0.58	0.016613
[R].LCDDGPQLPTSPR.[L]	578815137	SORBS3	10174	578815137 [559-571]	1xCarbamidomethyl [C2];1xPhospho [T/S];1xTMT6plex [N-Term]	T10(Phospho): 50;S11(Phospho): 50	12	0.417	0.015956
[R].GSGGGGGPQVPHQSPPKR.[V]	1370495603	FAM53C	51307	1370495603 [155-172]	1xPhospho [S14(100)];2xTMT6plex [N-Term;K17]	S14(Phospho): 100	3	0.427	0.015908
[R].VGGSDEEASGIPSR.[T]	99028871	PPAN-P2RY11	692312	99028871 [356-369]	1xPhospho [S4(100)];1xTMT6plex [N-Term]	S4(Phospho): 100	3	1.395	0.015870
[K].YVISDEEEEDDD.[-]	20270337	LEO1	123169	20270337 [655-666]	1xPhospho [S4(93.6)];1xTMT6plex [N-Term]	S4(Phospho): 100	1	0.608	0.015198
[R].SGAQASSTPLSPTR.[I]	27436946	LMNA	4000	27436946 [12-25]	1xPhospho [S11(99)];1xTMT6plex [N-Term]	T8(Phospho): 49.99;S11(Phospho): 49.99	8	1.947	0.015036
[R].SPVPSPGSSSPQLQVK.[S]	530420479	MICALL1	85377	530420479 [612-627]	1xPhospho [S5(93.7)];2xTMT6plex [N-Term;K16]	S9(Phospho): 49.69;S10(Phospho): 49.69	10	0.344	0.014995
[R].HGLQLGAQSPGR.[G]	530365132	ZNF687	57592	530365132 [1058-1069]	1xPhospho [S9(100)];1xTMT6plex [N-Term]	S9(Phospho): 100	3	1.671	0.014698
[R].VLGSEGEEEDEALSPAK.[G]	4557719	LIG1	3978	4557719 [63-79]	2xPhospho [S4(100);2xTMT6plex [N-Term;K17];S14(100)]	S4(Phospho): 100;S14(Phospho): 100	5	0.157	0.014117
[R].ASPSPQPSSQPLQIHR.[Q]	530367488	EML4	27436	530367488 [143-158]	1xPhospho [S];1xTMT6plex [N-Term]	S2(Phospho): 50;S4(Phospho): 50	5	0.649	0.014045
[K].AADPPAENSSAPEAEQGGAE.[-]	34098946	YBX1	4904	34098946 [305-324]	1xPhospho [S];1xTMT6plex [N-Term]	S9(Phospho): 50;S10(Phospho): 50	12	0.653	0.013906
[K].SVAAEGALLPQTPPSPR.[N]	578801173	RALGPS2	55103	578801173 [315-331]	1xTMT6plex [N-Term];2xPhospho [T12(100);S15(100)]	T12(Phospho): 100;S15(Phospho): 100	2	0.615	0.013764
[R].SGSSSPDSEITELK.[F]	1370452107	CTPS1	1503	1370452107 [578-591]	2xPhospho [S/T];2xTMT6plex [N-Term;K14]	S4(Phospho): 98.96;S5(Phospho): 99.99	6	0.142	0.013759
[K].AVPMAPAPASPGSSNDSSAR.[S]	1034608385	MAP1S	55201	1034608385 [750-769]	1xOxidation [M4];1xPhospho [S10(100)];1xTMT6plex [N-Term]	S10(Phospho): 100	7	0.58	0.013063
[K].EYVSNDAAQSDDEEKLQSQPTDTDGGR.[L]	45238858	WDR44	54521	45238858 [394-420]	1xPhospho [S/T/Y];2xTMT6plex [N-Term;K15]	S10(Phospho): 95.8	8	0.269	0.013024
[K].NYAESDHSEDEDNDNNSATAEESTK.[K]	530360573	RERE	473	530360573 [49-73]	2xPhospho [S5(97.5);2xTMT6plex [N-Term;K25];S8(99.8)]	S5(Phospho): 97.48;S8(Phospho): 99.85	4	1.445	0.012931
[R].NHSPSPPVTPTGAAPSLASPK.[Q]	767943194	NHSL1	57224	767943194 [1446-1466]	2xPhospho [S3(100);2xTMT6plex [N-Term;K21];S5(99.7)]	S3(Phospho): 100;S5(Phospho): 100	6	0.578	0.012862
[R].LSSPVLHR.[L]	1034643898	DBN1	1627	1034643898 [142-149]	1xPhospho [S3(99.2)];1xTMT6plex [N-Term]	S3(Phospho): 99.99	5	1.195	0.012821
[R].AESPESSAIESTQSTPQKGR.[G]	530402180	PDS5B	23047	530402180 [1357-1376]	2xPhospho [S3(100);2xTMT6plex [N-Term;K18];S/T]	S3(Phospho): 100;S6(Phospho): 89.6	3	1.318	0.012643
[R].YSPSQNSPIHHIPSR.[R]	7661958	BCLAF1	9774	7661958 [284-298]	1xTMT6plex [N-Term];2xPhospho [S/Y]	S2(Phospho): 97.06;S7(Phospho): 99.98	5	0.465	0.012548
[R].GLGVGFGSGGGSSSSVK.[F]	119395754	KRT5	3852	119395754 [560-576]	2xPhospho [S14(95.9);2xTMT6plex [N-Term;K17];S15(95.9)]	S14(Phospho): 95.94;S15(Phospho): 95.94	9	0.571	0.012310
[R].AADSQNSGEGNTGAAESSFSQEVSR.[E]	114155142	TPR	7175	114155142 [2031-2055]	1xPhospho [S/T];1xTMT6plex [N-Term]	S7(Phospho): 98.39	4	1.982	0.012198
[R].LPSGSGAASPTGSAVDIR.[A]	1079280806	AHNAK	79026	1079280806 [208-225]	1xPhospho [T/S];1xTMT6plex [N-Term]	S9(Phospho): 50;T11(Phospho): 50	17	0.875	0.011928
[K].SAYQEYDSDSDVPEELKR.[D]	62865871	VPS50	55610	62865871 [552-569]	2xPhospho [Y/S];2xTMT6plex [N-Term;K17]	Y6(Phospho): 66.67;S8(Phospho): 66.67;S10(Phospho): 66.67	3	0.414	0.011831
[K].AAAAAPASEDEDDEDDEDDEDDDDEEDDSEEEAMETTPAKGK.[K]	55956788	NCL	4691	55956788 [177-219]	1xOxidation [M35];2xPhospho [S/T];3xTMT6plex [N-Term;K41;K43]	S8(Phospho): 50;S30(Phospho): 50;T37(Phospho): 50;T38(Phospho): 50	4	2.453	0.011529
[R].TAFYNEDDSEEEQR.[Q]	1081684143	PHIP	55023	1081684143 [1775-1788]	1xPhospho [S9(100)];1xTMT6plex [N-Term]	S9(Phospho): 100	3	1.41	0.011078
[K].VVFENEQDNSSLTK.[T]	767932824	KIAA1109	84162	767932824 [2745-2758]	1xPhospho [S/T];2xTMT6plex [N-Term;K14]	S10(Phospho): 33.33;S11(Phospho): 33.33;T13(Phospho): 33.33	3	1.858	0.010966
[K].KPAQEETEETSSQESAEED.[-]	4504431	HMGA2	8091	4504431 [91-109]	2xPhospho [T/S];2xTMT6plex [N-Term;K1]	T10(Phospho): 98.11;S12(Phospho): 96.26	70	1.765	0.010529
[K].VKPETPPRQSHSGSISPYPK.[V]	118572613	SRRM2	23524	118572613 [979-998]	2xPhospho [T5(100);3xTMT6plex [N-Term;K2;K20];S/Y]	T5(Phospho): 100;S10(Phospho): 25;S12(Phospho): 25;S14(Phospho): 25;S16(Phospho): 25	8	1.572	0.010251
[R].RTPSDDEEDNLFAPPK.[L]	1052792762	WASHC2C	253725	1052792762 [330-345]	1xPhospho [T/S];2xTMT6plex [N-Term;K16]	T2(Phospho): 50;S4(Phospho): 50	4	0.61	0.009670
[R].KLSGDQPAAR.[T]	530380665	TCOF1	6949	530380665 [1386-1395]	1xPhospho [S3(100)];2xTMT6plex [N-Term;K1]	S3(Phospho): 100	4	1.748	0.009521
[R].TQSSSCEDLPSTTQPK.[G]	768035974	CASK	8573	768035974 [573-588]	1xCarbamidomethyl [C6];1xPhospho [S/T];2xTMT6plex [N-Term;K16]	S5(Phospho): 99.25	9	2.411	0.009355
[R].ATGGGLSSVGGGSSTIK.[Y]	119703753	KRT6B	3854	119703753 [534-550]	1xPhospho [S14(97.8)];2xTMT6plex [N-Term;K17]	S13(Phospho): 99.22	5	1.949	0.009051
[R].AGEEDEGEEDSDSDYEISAK.[A]	41393547	NBAS	51594	41393547 [463-482]	2xPhospho [S11(98.7);2xTMT6plex [N-Term;K20];S/Y]	S11(Phospho): 98.67;S13(Phospho): 98.67	5	1.983	0.008951
[R].SVSGSPEPAAK.[K]	1034554585	SRRM1	10250	1034554585 [791-801]	2xPhospho [S3(98.8);2xTMT6plex [N-Term;K11];S5(100)]	S3(Phospho): 99.99;S5(Phospho): 100	10	2.67	0.008847
[R].SRSGEGEVSGLMR.[K]	1370474190	TRIM28	10155	1370474190 [471-483]	1xOxidation [M12];1xPhospho [S3(98.1)];1xTMT6plex [N-Term]	S3(Phospho): 98.1	3	1.408	0.008793
[R].SSPNVANQPPSPGGK.[S]	1034650321	AFDN	4301	1034650321 [1180-1194]	1xPhospho [S11(100)];2xTMT6plex [N-Term;K15]	S11(Phospho): 100	6	1.515	0.008242
[R].DVYLSPR.[D]	56699409	RBMX	27316	56699409 [204-210]	1xPhospho [S5(100)];1xTMT6plex [N-Term]	S5(Phospho): 100	3	1.322	0.008198
[R].AFSSRSYTSGPGSR.[I]	372466572	KRT8	3856	372466572 [47-60]	1xTMT6plex [N-Term];2xPhospho [S3(98.6);S6(93.3)]	S3(Phospho): 99.97;S6(Phospho): 98.32	3	2.196	0.008072
[R].HRPSPPATPPPK.[T]	1034554585	SRRM1	10250	1034554585 [424-435]	2xPhospho [S4(100);2xTMT6plex [N-Term;K12];T8(100)]	S4(Phospho): 100;T8(Phospho): 100	29	2.069	0.007991
[K].TASTNNIAQAR.[R]	1034560222	GNG12	55970	1034560222 [5-15]	1xPhospho [S3(97.8)];1xTMT6plex [N-Term]	S3(Phospho): 99.99	3	1.751	0.007912
[R].AAKLSEGSQPAEEEEDQETPSR.[N]	7662238	ACIN1	22985	7662238 [236-257]	2xPhospho [S5(100);2xTMT6plex [N-Term;K3];S8(100)]	S5(Phospho): 100;S8(Phospho): 100	4	3.341	0.007813
[R].ALPSLNTGSSSPR.[G]	219879806	TRAFD1	10906	219879806 [317-329]	1xPhospho [T/S];1xTMT6plex [N-Term]	S10(Phospho): 49.33;S11(Phospho): 49.33	13	0.617	0.007715
[R].TLSNAEDYLDDEDSD.[-]	578817327	OSTF1	26578	578817327 [203-217]	1xTMT6plex [N-Term];2xPhospho [S14(95.4);S/Y/T]	S3(Phospho): 99.98;S14(Phospho): 100	7	0.607	0.007691
[R].HYEDGYPGGSDNYGSLSR.[V]	146231940	CTNND1	1500	146231940 [216-233]	1xPhospho [S/Y];1xTMT6plex [N-Term]	S15(Phospho): 49.69;S17(Phospho): 49.69	7	0.462	0.007665
[R].AHSPAEGASVESSSPGPK.[K]	1213953124	FLCN	201163	1213953124 [60-77]	2xPhospho [S3(100);2xTMT6plex [N-Term;K18];S]	S3(Phospho): 100;S9(Phospho): 98.72	3	0.357	0.007655
[R].APQTSSSPPPVR.[R]	1034554585	SRRM1	10250	1034554585 [729-740]	1xPhospho [S/T];1xTMT6plex [N-Term]	S7(Phospho): 98.63	43	1.678	0.007223
[K].AGGSAALSPSK.[K]	5174449	H1FX	8971	5174449 [24-34]	1xPhospho [S8(100)];2xTMT6plex [N-Term;K11]	S8(Phospho): 100	6	0.638	0.007056
[R].TFSLDAVPPDHSPR.[A]	62739179	RTKN	6242	62739179 [518-531]	1xTMT6plex [N-Term];2xPhospho [S3(100);S12(100)]	S3(Phospho): 99.99;S12(Phospho): 100	9	0.56	0.007015
[R].MDFAFPGSTNSLHR.[M]	578830853	ITGB4	3691	578830853 [1482-1495]	1xOxidation [M1];1xPhospho [T/S];1xTMT6plex [N-Term]	S11(Phospho): 98.81	7	0.47	0.006999
[R].AALAPAKESPR.[K]	530380665	TCOF1	6949	530380665 [898-908]	1xPhospho [S9(100)];2xTMT6plex [N-Term;K7]	S9(Phospho): 100	3	0.295	0.006802
[R].SSQPSPTAVPASDSPPTKQEVK.[K]	78042577	MAP7D1	55700	78042577 [112-133]	2xPhospho [S5(97.3);3xTMT6plex [N-Term;K18;K22];S/T]	S5(Phospho): 95.42;S14(Phospho): 99.94	9	0.28	0.006785
[R].ASSPPDRIDIFGR.[T]	134152708	ARGLU1	55082	134152708 [75-87]	1xPhospho [S3(99.1)];1xTMT6plex [N-Term]	S3(Phospho): 99.06	6	1.979	0.006772
[K].STSAPQMSPGSSDNQSSSPQPAQQK.[L]	530365733	UBAP2L	9898	530365733 [471-495]	1xOxidation [M7];2xPhospho [S/T];2xTMT6plex [N-Term;K25]	S17(Phospho): 80.87;S18(Phospho): 80.87	33	0.381	0.006732
[K].IEDVGSDEEDDSGK.[D]	1370507998	HSP90AB1	3326	1370507998 [250-263]	1xPhospho [S6(100)];2xTMT6plex [N-Term;K14]	S6(Phospho): 100	6	0.193	0.006409
[R].LESLSETPGPSSPR.[Q]	50959191	ARHGEF5	7984	50959191 [1115-1128]	1xPhospho [S/T];1xTMT6plex [N-Term]	S11(Phospho): 49.76;S12(Phospho): 49.76	6	0.632	0.006245
[K].EAASGTTPQK.[S]	530380665	TCOF1	6949	530380665 [1302-1311]	1xPhospho [T7(97.6)];2xTMT6plex [N-Term;K10]	T7(Phospho): 99.99	11	0.42	0.006016
[R].VSPASPAGSPSADFAVHGESLGDR.[H]	1034663602	ENTR1	10807	1034663602 [288-311]	1xTMT6plex [N-Term];2xPhospho [S5(98.3);S9(98.3)]	S5(Phospho): 98.33;S9(Phospho): 99.97	3	0.481	0.005724
[K].ASASGSGAQVGGPISSGSSASSVTVTR.[S]	27436946	LMNA	4000	27436946 [598-624]	1xTMT6plex [N-Term];2xPhospho [S/T]	S18(Phospho): 98.15;S21(Phospho): 49.09;S22(Phospho): 49.09	62	2.483	0.005596
[R].TEARSSDEENGPPSSPDLDR.[I]	400153980	AKT1S1	84335	400153980 [218-237]	1xTMT6plex [N-Term];3xPhospho [S5(97.6);S15(97.6);S6(97.6)]	S5(Phospho): 97.55;S6(Phospho): 97.55;S15(Phospho): 97.55	4	0.225	0.005314
[R].VRLQSASPSTR.[W]	119829187	COL17A1	1308	119829187 [144-154]	1xTMT6plex [N-Term];2xPhospho [S5(100);S7(100)]	S5(Phospho): 100;S7(Phospho): 100	6	2.849	0.005286
[R].LLEGEDAHLSSSQFSSGSQSSRDVTSSSR.[Q]	15431310	KRT14	3861	15431310 [418-446]	1xPhospho [S/T];1xTMT6plex [N-Term]	S10(Phospho): 45.29;S11(Phospho): 45.29	21	0.503	0.005280
[R].SLYGLGGSK.[R]	119703753;5031839	KRT6B;KRT6A	3854;3853	119703753 [60-68];5031839 [60-68]	1xPhospho [S1(100)];2xTMT6plex [N-Term;K9]	S1(Phospho): 100	7	1.994	0.005194
[R].TASGSSVTSLDGTR.[S]	767952381	NDRG1	10397	767952381 [345-358]	1xTMT6plex [N-Term];3xPhospho [S/T]	S3(Phospho): 100;S6(Phospho): 97.93;T8(Phospho): 97.93	8	0.45	0.005158
[R].SLSYSPVER.[R]	118572613	SRRM2	23524	118572613 [2690-2698]	1xTMT6plex [N-Term];2xPhospho [S3(98.8);S5(98.8)]	S3(Phospho): 98.93;S5(Phospho): 98.93	9	1.774	0.005009

Patil et al.2020. Proteomic and phosphoproteomic profiling of shammah-induced signaling in oral keratinocytes

Supplementary Table S2: List of phosphopeptides identified and quantified in OKF6/TERT1-Shammah and OKF6/TERT1-Parental phosphoproteome using TMT/TiO_2-based quantitative phosphoproteomic approach

Peptide Sequence	Protein Accessions	Gene Symbol	Gene ID	Positions in Proteins	Modifications	PhosphoRS: Best Site Probabilities	# PSMs	Fold change	p-value
[R].KPSVGVPPPASPSYPR.[A]	112734870	KIAA1522	57648	112734870 [1028-1043]	2xPhospho [S3(100);2xTMT6plex [N-Term;K1];S11(96.5)]	S3(Phospho): 100;S11(Phospho): 96.5	3	1.43	0.004886
[K].AAAAAPASEDEDDEDDEDDEDDDDDEEDDSEEEAMETTPAKGK.[K]	55956788	NCL	4691	55956788 [177-219]	1xOxidation [M35];3xPhospho [T37(96.2);3xTMT6plex [N-Term;K41;K43];S];T38(96.2)]	S8(Phospho): 53.79;S30(Phospho): 53.79;T37(Phospho): 96.21;T38(Phospho): 96.21	8	0.44	0.004630
[R].TLNAETPKSSPLPAK.[G]	47271406	MEPCE	56257	47271406 [208-222]	2xPhospho [T6(100);3xTMT6plex [N-Term;K15];K8;S/T]	T6(Phospho): 99.99;S9(Phospho): 50.01;S10(Phospho): 50.01	3	2.079	0.004455
[R].LLEGEDAHLSSSQFSSGSQSSRDVTSSSR.[Q]	15431310	KRT14	3861	15431310 [418-446]	1xTMT6plex [N-Term];2xPhospho [S/T]	S16(Phospho): 49;S18(Phospho): 49;S20(Phospho): 49.97;S21(Phospho): 49.97	39	0.264	0.004373
[R].SGFSSVSVSR.[S]	5031839	KRT6A	3853	5031839 [31-40]	1xPhospho [S];1xTMT6plex [N-Term]	S7(Phospho): 100	9	2.502	0.004297
[K].GSLGISQEEQ.[-]	7706174	SPG21	51324	7706174 [299-308]	1xPhospho [S6(100)];1xTMT6plex [N-Term]	S6(Phospho): 100	3	0.485	0.004280
[R].ALGSAAADAQR.[A]	157419138	LAMC2	3918	157419138 [996-1006]	1xPhospho [S4(100)];1xTMT6plex [N-Term]	S4(Phospho): 100	3	2.787	0.004246
[R].TDSREDEISPPPPNPVVK.[G]	47132581	PRKAR1A	5573	47132581 [75-92]	1xPhospho [S/T];2xTMT6plex [N-Term;K18]	S3(Phospho): 49.72;S9(Phospho): 49.72	3	0.606	0.004175
[R].SGSSPEVDSK.[S]	118572613	SRRM2	23524	118572613 [1579-1588]	2xPhospho [S3(98.7);2xTMT6plex [N-Term;K10];S4(100)]	S3(Phospho): 99.98;S4(Phospho): 99.98	3	2.009	0.004170
[K].TSDVNETQPPQSE.[-]	767992184	MYH10	4628	767992184 [2025-2037]	1xPhospho [S12(100)];1xTMT6plex [N-Term]	S12(Phospho): 100	3	0.508	0.004153
[R].RTSMGGTQQQFVEGVR.[M]	1370483230	CTNNB1	1499	1370483230 [550-565]	1xOxidation [M4];1xPhospho [T/S];1xTMT6plex [N-Term]	T2(Phospho): 50;S3(Phospho): 50	23	1.574	0.003963
[R].RDSDGVDGFEAEGK.[K]	34101286	ZFR	51663	34101286 [1052-1065]	1xPhospho [S3(100)];2xTMT6plex [N-Term;K14]	S3(Phospho): 100	3	1.805	0.003801
[R].SSFASSSASDASKPSSPR.[G]	14249622	AJUBA	84962	14249622 [122-139]	1xPhospho [S];2xTMT6plex [N-Term;K13]	S12(Phospho): 97.06	7	0.423	0.003694
[R].RYSPSPPPK.[R]	1034554585	SRRM1	10250	1034554585 [642-650]	2xPhospho [S3(99.1);2xTMT6plex [N-Term;K9];S5(100)]	Y2(Phospho): 50;S3(Phospho): 50;S5(Phospho): 100	62	1.656	0.003623
[R].DSQDASAEQSDHDDEVASLASASGGFGTK.[V]	45827771	EDC4	23644	45827771 [870-898]	1xPhospho [S/T];2xTMT6plex [N-Term;K29]	S6(Phospho): 48.19;S10(Phospho): 48.19	5	0.331	0.003510
[R].SSPNVANQPPSPGGK.[S]	1034650321	AFDN	4301	1034650321 [1180-1194]	2xPhospho [S1(100);2xTMT6plex [N-Term;K15];S11(100)]	S1(Phospho): 99.99;S11(Phospho): 100	6	0.558	0.003483
[R].GFSANSARLPGVSR.[S]	119703753;5031839	KRT6B|KRT6A	3854|3853	119703753 [17-30];5031839 [17-30]	1xPhospho [S6(100)];1xTMT6plex [N-Term]	S6(Phospho): 100	3	3.013	0.003475
[R].KGAGDGSDEEVDGK.[A]	1034628998	MYH9	4627	1034628998 [1937-1950]	1xPhospho [S7(100)];3xTMT6plex [N-Term;K1;K14]	S7(Phospho): 100	5	0.264	0.003298
[R].AQISSPNLR.[T]	55749544	SH3PXD2A	9644	55749544 [389-397]	1xPhospho [S];1xTMT6plex [N-Term]	S5(Phospho): 98.84	3	0.625	0.003292
[R].ASSHSSQTQGGGSVTKK.[R]	27436946	LMNA	4000	27436946 [402-418]	2xPhospho [S/T];3xTMT6plex [N-Term;K16;K17]	S3(Phospho): 96.71;S5(Phospho): 33.36;S6(Phospho): 33.36;T8(Phospho): 33.36	3	3.521	0.003253
[K].IGEGTYGVVYK.[AG]	767972772	CDK2	1017	767972772 [10-20]	2xPhospho [T5(100);2xTMT6plex [N-Term;K11];Y6(97.9)]	T5(Phospho): 100;Y6(Phospho): 100	6	0.255	0.003229
[K].LDSSPSVSSTLAAK.[D]	530380665	TCOF1	6949	530380665 [1263-1276]	2xPhospho [S3(98.5);2xTMT6plex [N-Term;K14];S4(98.5)]	S3(Phospho): 98.5;S4(Phospho): 98.5	5	0.68	0.003213
[R].GLVAAYSGESDSEEEQERGGPEREEK.[L]	1370525026	RBM10	8241	1370525026 [826-851]	2xPhospho [S/Y];2xTMT6plex [N-Term;K26]	S10(Phospho): 95.44;S12(Phospho): 95.44	7	4.308	0.003145
[R].SRSPPPVSK.[R]	47271443	SRSF2	6427	47271443 [189-197]	2xPhospho [S1(100);2xTMT6plex [N-Term;K9];S3(100)]	S1(Phospho): 100;S3(Phospho): 100	3	0.425	0.003069
[K].YSPTSPTYSPTTPK.[Y]	4505939	POLR2A	5430	4505939 [1874-1887]	2xPhospho [T/S/Y];2xTMT6plex [N-Term;K14]	T4(Phospho): 50.44;S5(Phospho): 97.99;T7(Phospho): 49.47	13	0.585	0.003028
[R].APSASPLALHASR.[L]	530420479	MICALL1	85377	530420479 [482-494]	1xTMT6plex [N-Term];2xPhospho [S3(100);S5(100)]	S3(Phospho): 100;S5(Phospho): 100	9	0.516	0.002906
[R].AFSRGGSLESR.[S]	1034567534	PROSER2	254427	1034567534 [25-35]	1xTMT6plex [N-Term];2xPhospho [S3(100);S7(100)]	S3(Phospho): 100;S7(Phospho): 100	3	0.277	0.002894
[K].KAENAEGQTPAIGPDGEPLDETSQMSDLPVK.[V]	192807323	SMARCA4	6597	192807323 [588-618]	1xOxidation [M25];2xPhospho [T/S];3xTMT6plex [N-Term;K1;K31]	T22(Phospho): 66.67;S23(Phospho): 66.67;S26(Phospho): 66.67	6	1.443	0.002866
[R].DTYSDRSGSSSPDSEITELK.[F]	1370452107	CTPS1	1503	1370452107 [572-591]	2xPhospho [S7(84.2);2xTMT6plex [N-Term;K20];S9(84.2)]	S7(Phospho): 84.2;S9(Phospho): 84.2	3	0.23	0.002823
[R].RSRSVSPCSNVESR.[L]	118572613	SRRM2	23524	118572613 [949-962]	1xCarbamidomethyl [C8];1xTMT6plex [N-Term];3xPhospho [S2(98.3);S4(98.3);S6(88.6)]	S2(Phospho): 100;S4(Phospho): 100;S6(Phospho): 100	3	0.265	0.002650
[R].TNPPTQKPPSPPMSGR.[G]	61743942	ABI1	10006	61743942 [174-189]	1xOxidation [M13];1xPhospho [S10(98.2)];2xTMT6plex [N-Term;K7]	S10(Phospho): 98.25	4	0.502	0.002606
[R].GDQPAASGDSDDDEPPPLPR.[L]	5729875	PGRMC1	10857	5729875 [48-67]	1xPhospho [S];1xTMT6plex [N-Term]	S10(Phospho): 99.99	13	0.397	0.002551
[R].TASISSSPSEGTPTVGSYGCTPQSLPK.[F]	530380464	LARP1	23367	530380464 [845-871]	1xCarbamidomethyl [C20];2xPhospho [S3(96.1);2xTMT6plex [N-Term;K27];S/T/Y]	S3(Phospho): 96.07;S5(Phospho): 11.17;S6(Phospho): 11.17;S7(Phospho): 11.17;S9(Phospho): 11.17;T12(Phospho): 11.17;T14(Phospho): 11.17;S17(Phospho): 11.17;Y18(Phospho): 11.17;T21(Phospho): 11.17	4	0.384	0.002517
[R].HLAQASQELQ.[-]	31317307	PCSK9	255738	31317307 [683-692]	1xPhospho [S6(100)];1xTMT6plex [N-Term]	S6(Phospho): 100	3	3.282	0.002382
[R].ASSLSESSPPK.[A]	767974553	LIMA1	51474	767974553 [368-378]	2xPhospho [S5(97.7);2xTMT6plex [N-Term;K11];S]	S3(Phospho): 99.19;S5(Phospho): 99.19	9	0.417	0.002285
[K].VVTNARSPGAK.[C]	767985304	SLTM	79811	767985304 [557-567]	1xPhospho [S7(100)];2xTMT6plex [N-Term;K11]	S7(Phospho): 100	3	2.833	0.002141
[R].RLLEGEDAHLSSSQFSSGSQSSR.[D]	15431310	KRT14	3861	15431310 [417-439]	1xPhospho [S];1xTMT6plex [N-Term]	S17(Phospho): 49.68;S19(Phospho): 49.68	37	0.434	0.002137
[K].VGSLTPPSSPK.[T]	148277037	AAK1	22848	148277037 [616-626]	2xPhospho [S8(98.8);2xTMT6plex [N-Term;K11];S9(100)]	S8(Phospho): 100;S9(Phospho): 100	19	0.415	0.002135
[R].SSSDEQGLSYSSLK.[N]	268840316	IL1RAP	3556	268840316 [555-568]	1xPhospho [S/Y];2xTMT6plex [N-Term;K14]	S3(Phospho): 99.15	12	1.481	0.002096
[R].VAAAAGSGPSPPGSPGHDRER.[Q]	13559514	PI4K2A	55361	13559514 [38-58]	1xTMT6plex [N-Term];2xPhospho [S14(97.2);S]	S10(Phospho): 99.96;S14(Phospho): 100	21	1.641	0.002059
[R].AQSGSDSSPEPK.[A]	118572613	SRRM2	23524	118572613 [1614-1625]	2xPhospho [S];2xTMT6plex [N-Term;K12]	S7(Phospho): 99.98;S8(Phospho): 99.98	4	4.24	0.002042
[K].AAPQSPSVPK.[K]	24430149	NUP155	9631	24430149 [988-997]	1xPhospho [S5(100)];2xTMT6plex [N-Term;K10]	S5(Phospho): 100	9	1.815	0.002013
[R].RGFSANSARLPGVSR.[S]	119703753;5031839	KRT6B|KRT6A	3854|3853	119703753 [16-30];5031839 [16-30]	1xPhospho [S7(100)];1xTMT6plex [N-Term]	S7(Phospho): 98.42	3	2.563	0.002002
[R].LLPPASP.[-]	767987920	TELO2	9894	767987920 [893-899]	1xPhospho [S6(100)];1xTMT6plex [N-Term]	S6(Phospho): 100	3	0.318	0.002001
[K].ARSVSPPPK.[R]	20127499	SRSF6	6431	20127499 [312-320]	2xPhospho [S3(100);2xTMT6plex [N-Term;K9];S5(100)]	S3(Phospho): 100;S5(Phospho): 100	5	1.545	0.001937
[K].SNTEPQSPPIASPK.[A]	1394533392	TMF1	7110	1394533392 [66-79]	2xPhospho [S12(98.4);2xTMT6plex [N-Term;K14];S/T]	S7(Phospho): 100;S12(Phospho): 100	3	2.434	0.001826
[R].TAQVPSPPRGK.[I]	7662238	ACIN1	22985	7662238 [999-1009]	1xPhospho [S6(100)];2xTMT6plex [N-Term;K11]	S6(Phospho): 100	5	2.714	0.001818
[K].TGSGSPFAGNSPAR.[E]	126723060	ZC3H4	23211	126723060 [1265-1278]	1xTMT6plex [N-Term];2xPhospho [S5(97.9);S11(100)]	S5(Phospho): 97.93;S11(Phospho): 100	3	0.544	0.001760
[R].FIHQQPQSSSPVYGSSAK.[T]	1034580879	PXN	5829	1034580879 [82-99]	1xPhospho [S8(97.9)];2xTMT6plex [N-Term;K18]	S8(Phospho): 97.41	9	0.249	0.001725
[R].SLMSSPEDLTK.[D]	153945728	MAP1B	4131	153945728 [828-838]	1xOxidation [M3];2xPhospho [T/S];2xTMT6plex [N-Term;K11]	S4(Phospho): 100;S5(Phospho): 100	4	0.231	0.001701
[R].VGGSSVDLHR.[F]	146231940	CTNND1	1500	146231940 [265-274]	1xTMT6plex [N-Term];2xPhospho [S4(100);S5(100)]	S4(Phospho): 100;S5(Phospho): 100	6	2.115	0.001690
[R].SGAQASSTPLSPTR.[I]	27436946	LMNA	4000	27436946 [12-25]	1xTMT6plex [N-Term];2xPhospho [T8(98.4);S11(98.4)]	S7(Phospho): 49.99;T8(Phospho): 49.99;S11(Phospho): 98.02	13	0.401	0.001643
[R].ALQTSPQPPEGPGLPR.[R]	767985868	PPIP5K1	9677	767985868 [956-971]	1xPhospho [T/S];1xTMT6plex [N-Term]	T4(Phospho): 50;S5(Phospho): 50	6	0.59	0.001617
[K].EYIPGQPPLSQSSDSSPTR.[N]	62241042	EPRS	2058	62241042 [871-889]	1xTMT6plex [N-Term];2xPhospho [S/T/Y]	S10(Phospho): 66.12;S12(Phospho): 66.12;S13(Phospho): 66.12	17	0.499	0.001580
[K].ERRTPSDDEEDNLFAPPK.[L]	1052792762	WASHC2C	253725	1052792762 [328-345]	2xPhospho [T4(100);2xTMT6plex [N-Term;K18];S6(100)]	T4(Phospho): 100;S6(Phospho): 100	5	0.235	0.001567
[R].TEARSSDEENGPPSSPDLDR.[I]	400153980	AKT1S1	84335	400153980 [218-237]	1xTMT6plex [N-Term];2xPhospho [S/T]	S5(Phospho): 50;S6(Phospho): 50;S14(Phospho): 50;S15(Phospho): 50	4	0.314	0.001536
[K].VSEEQTQPPSPAGAGMSTAMGR.[S]	1424027871	IFI16	3428	1424027871 [144-165]	1xPhospho [S/T];1xTMT6plex [N-Term];2xOxidation [M16;M20]	T6(Phospho): 49.32;S10(Phospho): 49.32	62	0.696	0.001488
[K].LSSQISAGEEK.[W]	1387845409	MTDH	92140	1387845409 [293-303]	1xPhospho [S6(100)];2xTMT6plex [N-Term;K11]	S6(Phospho): 99.99	3	1.641	0.001475
[R].DNTFFRESPVGR.[K]	1034579978	PPHLN1	51535	1034579978 [133-144]	1xPhospho [S8(100)];1xTMT6plex [N-Term]	S8(Phospho): 100	3	1.801	0.001406
[R].HGSGADSDYENTQSGDPLLGLEGK.[R]	1370470787	GIT1	28964	1370470787 [631-654]	2xPhospho [S3(100);2xTMT6plex [N-Term;K24];S/Y/T]	S3(Phospho): 99.98;S7(Phospho): 99.98	3	0.312	0.001364
[R].RFSLSPSLGPQASR.[F]	1370495603	FAM53C	51307	1370495603 [236-249]	1xTMT6plex [N-Term];2xPhospho [S3(100);S5(100)]	S3(Phospho): 100;S5(Phospho): 100	3	0.4	0.001354
[R].LLQPGGGPDVGTGAPRPGCSPR.[A]	116063568	TOR4A	54863	116063568 [44-65]	1xCarbamidomethyl [C19];1xPhospho [T/S];1xTMT6plex [N-Term]	T12(Phospho): 50;S20(Phospho): 50	5	2.268	0.001337
[K].VVDYSQFQESDDADEDYGR.[D]	56118310	NUCKS1	64710	56118310 [10-28]	1xPhospho [S/Y];1xTMT6plex [N-Term]	S10(Phospho): 100	25	0.369	0.001295
[R].ERSPSPSFR.[W]	916413046	PCYT1A	5130	916413046 [327-335]	1xTMT6plex [N-Term];2xPhospho [S3(100);S]	S3(Phospho): 100;S5(Phospho): 50;S7(Phospho): 50	3	0.449	0.001286
[K].TEESPASDEAGEK.[E]	48255933	HMGN1	3150	48255933 [83-95]	2xPhospho [S4(100);2xTMT6plex [N-Term;K13];S7(100)]	S4(Phospho): 100;S7(Phospho): 100	3	0.72	0.001217
[R].ASSHSSQTQGGGSVTK.[K]	27436946	LMNA	4000	27436946 [402-417]	2xPhospho [S/T];2xTMT6plex [N-Term;K16]	S3(Phospho): 98.05;S6(Phospho): 98.05	73	2.118	0.001192
[R].LRLSPSPTSQR.[S]	27436946	LMNA	4000	27436946 [387-397]	1xPhospho [S4(100)];1xTMT6plex [N-Term]	S4(Phospho): 100	6	2.576	0.001135
[K].ASVSGPNSPSETRR.[E]	4557753	MID1	4281	4557753 [89-102]	1xTMT6plex [N-Term];2xPhospho [S4(97);S8(94.3)]	S4(Phospho): 99.95;S8(Phospho): 99.95	3	0.392	0.001083
[K].RTEARSSDEENGPPSSPDLDR.[I]	400153980	AKT1S1	84335	400153980 [217-237]	1xTMT6plex [N-Term];2xPhospho [T2(96);S15(98)]	S15(Phospho): 93.16;S16(Phospho): 93.16	3	0.394	0.001003
[K].SGTSSPQSPVFR.[H]	767929330	AFAP1	60312	767929330 [745-756]	1xPhospho [S8(100)];1xTMT6plex [N-Term]	S8(Phospho): 100	3	2.45	0.000956
[R].AASPYSQRPASPTAIR.[R]	767920201	PKP4	8502	767920201 [271-286]	1xTMT6plex [N-Term];2xPhospho [S3(100);T13(100)]	S3(Phospho): 99.94;T13(Phospho): 98.24	6	0.705	0.000954
[R].LAIQGPEDSPSR.[Q]	4885487	MLF2	8079	4885487 [230-241]	1xPhospho [S];1xTMT6plex [N-Term]	S9(Phospho): 99.23	6	2.362	0.000932
[K].KSLDSDESEDEEDDYQQK.[R]	7657441	PDAP1	11333	7657441 [56-73]	2xPhospho [S5(95.9);3xTMT6plex [N-Term;K1;K18];S/Y]	S5(Phospho): 98.04;S8(Phospho): 99.96	3	0.35	0.000930
[R].SFLSEPSSPGR.[T]	33946282	VIRMA	25962	33946282 [1572-1582]	1xPhospho [S];1xTMT6plex [N-Term]	S7(Phospho): 49.72;S8(Phospho): 49.72	5	1.478	0.000904
[R].LQPERGSPK.[K]	7662238	ACIN1	22985	7662238 [723-731]	1xPhospho [S7(100)];2xTMT6plex [N-Term;K9]	S7(Phospho): 100	3	1.831	0.000897
[K].TLSVAAAFNEDEDSEPEEMPPEAK.[M]	9966827	PCNP	57092	9966827 [106-129]	1xOxidation [M19];1xPhospho [S14(92.8)];2xTMT6plex [N-Term;K24]	S14(Phospho): 100	6	0.441	0.000890
[K].GTEKRESPSPAPKPR.[K]	1034554585	SRRM1	10250	1034554585 [469-483]	2xPhospho [S7(97.3);3xTMT6plex [N-Term;K13];K4;S9(97.3)]	S7(Phospho): 96.93;S9(Phospho): 96.93	3	2.396	0.000872
[K].TPASTPVSGTPQASPMIER.[S]	32171238	BAIAP2L1	55971	32171238 [248-266]	1xOxidation [M16];1xTMT6plex [N-Term];2xPhospho [S14(99.9);S/T]	T10(Phospho): 97.88;S14(Phospho): 100	11	0.531	0.000816
[R].SPSPAHLPDDPK.[V]	1034567413	LARP4B	23185	1034567413 [1099-1110]	1xPhospho [S3(99.2)];2xTMT6plex [N-Term;K12]	S3(Phospho): 99.16	3	0.373	0.000812
[R].NLDNVSPK.[D]	1034626887	ADARB1	104	1034626887 [70-77]	1xPhospho [S6(100)];2xTMT6plex [N-Term;K8]	S6(Phospho): 100	4	2.55	0.000809
[R].SQSLPNSLDYTQTSDPGR.[H]	1021589337	RMDN3	55177	1021589337 [44-61]	1xPhospho [S3(98.5)];1xTMT6plex [N-Term]	S3(Phospho): 99.97	6	3.182	0.000791
[K].RVPSPTPAPK.[E]	118572613	SRRM2	23524	118572613 [2578-2587]	1xPhospho [S4(100)];2xTMT6plex [N-Term;K10]	S4(Phospho): 100	3	1.692	0.000789
[R].LRLSPSPTSQR.[S]	27436946	LMNA	4000	27436946 [387-397]	1xTMT6plex [N-Term];2xPhospho [S4(100);S/T]	S4(Phospho): 100;T8(Phospho): 49.58;S9(Phospho): 49.58	47	2.012	0.000785
[R].ARSTPSPVLQPR.[D]	530425459	DOT1L	84444	530425459 [897-908]	1xTMT6plex [N-Term];2xPhospho [S6(100);S/T]	S3(Phospho): 50;T4(Phospho): 50;S6(Phospho): 100	6	1.57	0.000769
[K].ISEGLPTPTKMTPR.[S]	108773787	RB1	5925	108773787 [815-828]	1xOxidation [M11];2xPhospho [T7(100);2xTMT6plex [N-Term;K10];T12(100)]	T7(Phospho): 100;T12(Phospho): 100	4	0.206	0.000676
[R].SNSSSEAVLGQEELSAQAK.[V]	24308075	RAB11FIP5	26056	24308075 [393-411]	1xPhospho [S3(97.3)];2xTMT6plex [N-Term;K19]	S3(Phospho): 97.26	8	1.939	0.000625
[K].DEGLCSANVTR.[Y]	5730091	TFPI2	7980	5730091 [163-173]	1xCarbamidomethyl [C5];1xPhospho [S6(100)];1xTMT6plex [N-Term]	S6(Phospho): 100	3	0.449	0.000590
[R].VSDQNSPVLPK.[K]	1034557138	USP24	23358	1034557138 [2062-2072]	1xPhospho [S6(100)];2xTMT6plex [N-Term;K11]	S6(Phospho): 100	6	0.47	0.000558
[K].LGASNSPGQPNSVKR.[K]	55741709	RBM25	58517	55741709 [672-686]	1xPhospho [S6(100)];2xTMT6plex [N-Term;K14]	S6(Phospho): 99.98	3	1.819	0.000490
[R].NLSSTTDDEAPR.[L]	20070205	LAD1	3898	20070205 [36-47]	1xTMT6plex [N-Term];2xPhospho [S3(100);S4(91.7)]	S3(Phospho): 100;S4(Phospho): 99.99	10	8.524	0.000489
[R].TASPPPPPKR.[R]	1034554585	SRRM1	10250	1034554585 [653-662]	2xPhospho [T1(100);2xTMT6plex [N-Term;K9];S3(100)]	T1(Phospho): 100;S3(Phospho): 100	24	1.689	0.000471
[R].GGLSPANDTGAK.[K]	10835073	NMT1	4836	10835073 [44-55]	1xPhospho [S4(100)];2xTMT6plex [N-Term;K12]	S4(Phospho): 100	3	0.24	0.000408
[R].TDGSISGDRQPVTVADYISR.[A]	259013556	FXR2	9513	259013556 [598-617]	1xTMT6plex [N-Term];2xPhospho [S4(97.8);S6(100)]	S4(Phospho): 99.97;S6(Phospho): 100	6	0.354	0.000400
[R].GGSLLAGGGGFGGGSLSGGGGSR.[S]	767994709	KRT15	3866	767994709 [20-42]	1xPhospho [S15(100)];1xTMT6plex [N-Term]	S15(Phospho): 99.99	7	2.54	0.000365
[R].AFSSRSYTSGPGSR.[I]	372466572	KRT8	3856	372466572 [47-60]	1xPhospho [S6(100)];1xTMT6plex [N-Term]	S6(Phospho): 99.98	3	4.135	0.000310
[R].GNDPLTSSPGR.[S]	33356547	MCM2	4171	33356547 [20-30]	1xPhospho [S8(98.7)];1xTMT6plex [N-Term]	S8(Phospho): 98.72	5	0.512	0.000275
[R].SQSDLDDQHDYDSVASDEDTDQEPLR.[S]	1370470787	GIT1	28964	1370470787 [414-439]	1xTMT6plex [N-Term];2xPhospho [S/Y/T]	S13(Phospho): 98.03;S16(Phospho): 98.03	3	0.351	0.000262
[K].RPESPSEISPIK.[G]	767962262	WAPL	23063	767962262 [218-229]	2xPhospho [S4(100);2xTMT6plex [N-Term;K12];S9(100)]	S4(Phospho): 100;S9(Phospho): 100	4	0.6	0.000256
[R].LELQGPRGSPNAR.[S]	55956788	NCL	4691	55956788 [555-567]	1xPhospho [S9(100)];1xTMT6plex [N-Term]	S9(Phospho): 100	3	2.928	0.000231
[K].VGSTENIK.[H]	1370484102	MAP4	4134	1370484102 [2113-2120]	1xPhospho [S3(100)];2xTMT6plex [N-Term;K8]	S3(Phospho): 100	3	0.263	0.000230
[R].ISISTSGGSFR.[N]	119395754	KRT5	3852	119395754 [74-84]	1xPhospho [S/T];1xTMT6plex [N-Term]	S9(Phospho): 100	3	0.351	0.000206
[R].SRSPESQVIGENTK.[Q]	42741682	ZRANB2	9406	42741682 [305-318]	2xPhospho [S1(100);2xTMT6plex [N-Term;K14];S3(100)]	S1(Phospho): 100;S3(Phospho): 100	3	0.272	0.000200
[R].LNQPGTPTR.[T]	13489054	MAP2K2	5605	13489054 [389-397]	1xPhospho [T6(99.2)];1xTMT6plex [N-Term]	T6(Phospho): 100	3	0.589	0.000185
[R].SGKNSQEDSEDSEDKDVK.[T]	56118310	NUCKS1	64710	56118310 [50-67]	2xPhospho [S5(100);4xTMT6plex [N-Term;K15;K18];K3;S9(97.6)]	S9(Phospho): 100;S12(Phospho): 100	4	0.469	0.000169
[K].LGVSVSPSR.[A]	661567300	ZC3H18	124245	661567300 [553-561]	1xPhospho [S6(100)];1xTMT6plex [N-Term]	S6(Phospho): 99.23	3	1.514	0.000149
[R].LLEGEDAHLSSSQFSSGSQSSR.[D]	15431310	KRT14	3861	15431310 [418-439]	1xPhospho [S18(100)];1xTMT6plex [N-Term]	S18(Phospho): 100	113	0.165	0.000133
[K].EIPSATQSPISK.[K]	157694494	MYBBP1A	10514	157694494 [1156-1167]	1xPhospho [S8(97.3)];2xTMT6plex [N-Term;K12]	S8(Phospho): 99	11	0.51	0.000108
[R].TGSNAAQYK.[Y]	767929330	AFAP1	60312	767929330 [630-638]	1xPhospho [S3(100)];2xTMT6plex [N-Term;K9]	S3(Phospho): 100	3	1.775	0.000101
[K].LTGSTSSLNK.[L]	530376797	EXOC1	55763	530376797 [485-494]	2xPhospho [S4(98.2);2xTMT6plex [N-Term;K10];S7(99.1)]	S4(Phospho): 97.89;S7(Phospho): 98.92	6	0.32	0.000092
[K].LVTSGAESGNLNTSPSSNQTR.[N]	530399758	NEDD1	121441	530399758 [510-530]	1xPhospho [S14(98.8)];1xTMT6plex [N-Term]	S14(Phospho): 98.79	10	0.418	0.000070
[R].DQTASAPATPLVNK.[H]	510937001	COBLL1	22837	510937001 [318-331]	2xPhospho [S5(100);2xTMT6plex [N-Term;K14];T9(100)]	S5(Phospho): 99.99;T9(Phospho): 100	3	0.218	0.000061
[R].SLYNLGGSK.[R]	119395754	KRT5	3852	119395754 [64-72]	1xPhospho [S1(100)];2xTMT6plex [N-Term;K9]	S1(Phospho): 100	6	0.549	0.000041
[R].SLYGLGGSKR.[I]	119703753;5031839	KRT6B|KRT6A	3854|3853	119703753 [60-69];5031839 [60-69]	1xPhospho [S1(100)];2xTMT6plex [N-Term;K9]	S1(Phospho): 99.98	5	3.239	0.000040
[R].GLLYDSDEEDEERPAR.[K]	33356547	MCM2	4171	33356547 [134-149]	1xPhospho [S/Y];1xTMT6plex [N-Term]	S6(Phospho): 100	3	0.469	0.000038
[R].SLPTTVPESPNYR.[N]	530380464	LARP1	23367	530380464 [766-778]	1xPhospho [S9(100)];1xTMT6plex [N-Term]	S9(Phospho): 99.97	7	0.358	0.000029
[K].HTGPNSPDTANDGFVR.[L]	767938350	HNRNPH1	3187	767938350 [99-114]	1xPhospho [S/T];1xTMT6plex [N-Term]	S6(Phospho): 100	9	2.245	0.000027
[R].TVSASSTGDLPK.[A]	1370510834	SRPK2	6733	1370510834 [540-551]	2xPhospho [S3(100);2xTMT6plex [N-Term;K12];S5(100)]	S3(Phospho): 99.99;S5(Phospho): 99.06	8	0.639	0.000021
[R].GSPATSPHLGR.[R]	68989256	RIN1	9610	68989256 [332-342]	1xTMT6plex [N-Term];2xPhospho [S2(98.9);T/S]	S2(Phospho): 98.91;S6(Phospho): 99.99	7	0.366	0.000011
[R].SPSFGDPQLSPEARPR.[CV]	1370457682	SVIL	6840	1370457682 [261-276]	1xTMT6plex [N-Term];2xPhospho [S3(94.8);S10(99.4)]	S3(Phospho): 98.87;S10(Phospho): 100	3	3.847	0.000006
[K].LARVDSEGDFSENDDAAGDFR.[S]	5031579	AKAP8	10270	5031579 [318-338]	1xTMT6plex [N-Term];2xPhospho [S6(100);S11(100)]	S6(Phospho): 100;S11(Phospho): 100	3	2.828	0.000001
[R].LDYVPSSASREVR.[H]	1034659998	FAM83H	286077	1034659998 [709-721]	1xTMT6plex [N-Term];2xPhospho [S/Y]	S6(Phospho): 98.32;S9(Phospho): 99.97	9	7.003	0.000000
[R].GPPSPPAPVMHSPSR.[K]	974005398	SNW1	22938	974005398 [221-235]	1xOxidation [M10];1xTMT6plex [N-Term];2xPhospho [S4(100);S]	S4(Phospho): 100;S12(Phospho): 50;S14(Phospho): 50	12	1	0.999587
[K].EGDVLRRSEEHSPPR.[G]	530389014	RBM12B	389677	530389014 [243-257]	1xTMT6plex [N-Term];2xPhospho [S8(100);S12(100)]	S8(Phospho): 100;S12(Phospho): 100	4	0.958	0.999326
[K].SGTPPRQGSITSPQANEQSVTPQR.[R]	118572613	SRRM2	23524	118572613 [846-869]	1xTMT6plex [N-Term];2xPhospho [S/T]	T3(Phospho): 88.25;S9(Phospho): 33.23;T11(Phospho): 33.23;S12(Phospho): 33.23	1	2.208	0.998828
[R].MLPHAPGVQMQAIPEDAVHEDSGDEDGEDPDKR.[I]	293336691	HDAC2	3066	293336691 [373-405]	1xPhospho [S22(100)];2xOxidation [M1;2xTMT6plex [N-Term;K32];M10]	S22(Phospho): 100	4	0.999	0.998566
[R].DLAFEVPERGSRPASPAAK.[L]	767918807	KANSL3	55683	767918807 [501-519]	2xPhospho [S11(100);2xTMT6plex [N-Term;K19];S15(100)]	S11(Phospho): 100;S15(Phospho): 100	1	0.367	0.998033
[R].SVASNQSEMEFSSLQDMPK.[E]	24432106	CCAR2	57805	24432106 [675-693]	1xPhospho [S];2xOxidation [M9;2xTMT6plex [N-Term;K19];M17]	S1(Phospho): 48.3;S4(Phospho): 48.3	1	1.516	0.997964
[R].YEPSDKDRQSPPPAK.[R]	661567300	ZC3H18	124245	661567300 [857-871]	1xPhospho [S10(100)];3xTMT6plex [N-Term;K15];K6	S10(Phospho): 99.98	2	0.958	0.997528
[R].VDDDSLGEFPVTNSR.[A]	1370459882	DPF2	5977	1370459882 [138-152]	1xPhospho [S5(99.9)];1xTMT6plex [N-Term]	S5(Phospho): 99.93	1	1.363	0.997084
[K].ALIQTCLNSPDSPPRSDPTTDQR.[M]	4507205	SRF	6722	4507205 [213-235]	1xCarbamidomethyl [C6];1xPhospho [T/S];1xTMT6plex [N-Term]	T5(Phospho): 33.11;S9(Phospho): 33.11;S12(Phospho): 33.11	7	1.334	0.996196
[K].NLATSADTPPSTVPGTGK.[S]	530407763	SMG1	23049	530407763 [3594-3611]	2xPhospho [T/S];2xTMT6plex [N-Term;K18]	S5(Phospho): 98.14;T8(Phospho): 98.06	1	0.792	0.995976
[R].EVQAEQPSSSSPR.[R]	118600973	RBMX2	51634	118600973 [178-190]	1xPhospho [S];1xTMT6plex [N-Term]	S8(Phospho): 33.24;S9(Phospho): 33.24;S10(Phospho): 33.24	20	0.999	0.995516
[K].SFSKEELMSSDLEETAGSTSIPK.[R]	32483374	NOP56	10528	32483374 [511-533]	1xOxidation [M8];1xPhospho [S/T];3xTMT6plex [N-Term;K23];K4	S9(Phospho): 50;S10(Phospho): 50	2	1.172	0.994662
[R].SSPPLRTPDVLESSGPAVR.[S]	4506735	RPS6KA4	8986	4506735 [681-699]	1xTMT6plex [N-Term];2xPhospho [S2(97.7);T7(97.7)]	S2(Phospho): 97.7;T7(Phospho): 97.7	1	0.632	0.993994
[R].LDSSPVSSPR.[K]	87116683	ZFC3H1	196441	87116683 [946-955]	1xTMT6plex [N-Term];2xPhospho [S]	S3(Phospho): 49.83;S4(Phospho): 49.83;S7(Phospho): 50.17;S8(Phospho): 50.17	3	0.864	0.993923
[K].AKSPVPKSPVEEK.[G]	157738649	NEFM	4741	157738649 [613-625]	2xPhospho [S3(100);4xTMT6plex [N-Term;K13];K2;K7;S8(100)]	S3(Phospho): 100;S8(Phospho): 100	1	4.599	0.993573
[R].GVQKPAGPSTSPDGNSR.[C]	578815063	PRAG1	157285	578815063 [138-154]	1xPhospho [S/T];2xTMT6plex [N-Term;K4]	T10(Phospho): 49.36;S11(Phospho): 49.36	1	0.699	0.992802
[K].KVVEAVNSDSDSEFGIPK.[K]	1061214220	TOP2B	7155	1061214220 [1515-1532]	2xPhospho [S];3xTMT6plex [N-Term;K1;K18]	S8(Phospho): 50.4;S10(Phospho): 99.2;S12(Phospho): 50.4	8	0.996	0.991488
[R].SATDGNTSTTPPTSAK.[K]	530422563	CUL4B	8450	530422563 [40-55]	2xPhospho [T7(98.8);2xTMT6plex [N-Term;K16];S14(100)]	T7(Phospho): 98.83;S14(Phospho): 99.99	1	0.831	0.991344
[K].RKAEDSDSEPEPEDNVR.[L]	964750564	XRN2	22803	964750564 [572-588]	2xPhospho [S6(100);2xTMT6plex [N-Term;K2];S8(100)]	S6(Phospho): 100;S8(Phospho): 100	2	0.724	0.990699
[R].RFSEGVLQSPSQDQEK.[L]	578821824	TNKS1BP1	85456	578821824 [427-442]	1xPhospho [S3(100)];2xTMT6plex [N-Term;K16]	S3(Phospho): 100	1	0.458	0.990377
[K].ELSNSPLR.[E]	118572613	SRRM2	23524	118572613 [1316-1323]	1xPhospho [S5(99)];1xTMT6plex [N-Term]	S5(Phospho): 99.99	2	0.891	0.990162
[K].KETQSPEQVK.[S]	7661958	BCLAF1	9774	7661958 [492-501]	1xPhospho [S/T];3xTMT6plex [N-Term;K1;K10]	S5(Phospho): 99.99	2	1.105	0.989946
[R].LQAKESPQR.[K]	157743245	WDR43	23160	157743245 [72-80]	1xPhospho [S6(100)];2xTMT6plex [N-Term;K4]	S6(Phospho): 100	1	1.567	0.989572
[R].VPAEDETQSIDSEDSFVPGR.[R]	62865649	RFFL	117584	62865649 [218-237]	1xTMT6plex [N-Term];2xPhospho [T/S]	T7(Phospho): 50;S9(Phospho): 50;S12(Phospho): 50;S15(Phospho): 50	3	1.85	0.989496
[K].KAEGEPQEESPLK.[S]	7661958	BCLAF1	9774	7661958 [168-180]	1xPhospho [S10(100)];3xTMT6plex [N-Term;K1;K13]	S10(Phospho): 100	2	0.964	0.989441
[K].EGALSRVSDESLSKVQEAESPVFK.[E]	530388000	SLC20A2	6575	530388000 [249-272]	2xPhospho [S8(97.3);3xTMT6plex [N-Term;K14;K24];S]	S8(Phospho): 97.31;S11(Phospho): 49.32;S13(Phospho): 49.32	1	1.062	0.989313
[K].NEEPSEEEIDAPKPK.[K]	50659095	DDX21	9188	50659095 [117-131]	1xPhospho [S5(100)];3xTMT6plex [N-Term;K13;K15]	S5(Phospho): 100	2	1.131	0.988945
[R].SRTSVQTEDDQLIAGQSAR.[A]	1022433203	CTNNA1	1495	1022433203 [652-670]	1xPhospho [S/T];1xTMT6plex [N-Term]	S4(Phospho): 97.54	4	1.182	0.988853
[K].RIDFIPVSPAPSPTR.[G]	1433354118	FAM122A	116224	1433354118 [136-150]	1xTMT6plex [N-Term];2xPhospho [S8(100);S12(98.5)]	S8(Phospho): 100;S12(Phospho): 98.53	2	0.855	0.986201
[R].SITSTPLSGK.[E]	1034644516	RAI14	26064	1034644516 [299-308]	2xPhospho [S1(100);2xTMT6plex [N-Term;K10];T/S]	S1(Phospho): 99.99;T3(Phospho): 98.06	4	1.009	0.985352
[K].TEEPLGSPHPSGTVESPGEGPQDTR.[I]	8923582	ZNF446	55663	8923582 [131-155]	1xTMT6plex [N-Term];2xPhospho [S/T]	S7(Phospho): 65.71;S11(Phospho): 65.72;T13(Phospho): 65.72	3	1.006	0.983368
[K].AVSPPPSPR.[A]	1034657213	TNRC18	84629	1034657213 [989-997]	1xTMT6plex [N-Term];2xPhospho [S3(100);S7(100)]	S3(Phospho): 100;S7(Phospho): 100	2	1.185	0.983336
[K].RRSPPADAIPK.[S]	114796644	RCC1	1104	114796644 [9-19]	1xPhospho [S3(100)];2xTMT6plex [N-Term;K11]	S3(Phospho): 100	1	1.756	0.983289
[R].TASGSSVTSLDGTRSR.[S]	767952381	NDRG1	10397	767952381 [345-360]	1xTMT6plex [N-Term];2xPhospho [S3(87.3);T/S]	S3(Phospho): 99.96;T13(Phospho): 50;S15(Phospho): 50	1	0.73	0.983046

Patil et al,2020. Proteomic and phosphoproteomic profiling of shammah-induced signaling in oral keratinocytes

Supplementary Table S2: List of phosphopeptides identified and quantified in OKF6/TERT1-Shammah and OKF6/TERT1-Parental phosphoproteome using TMT/TiO_2-based quantitative phosphoproteomic approach

Peptide Sequence	Protein Accessions	Gene Symbol	Gene ID	Positions in Proteins	Modifications	PhosphoRS: Best Site Probabilities	# PSMs	Fold change	p-value
[R].VASPSQGQVGSSSPKR.[S]	767920201	PKP4	8502	767920201 [325-340]	2xPhospho [S11(95);2xTMT6plex [N-Term;K15];S12(95)]	S11(Phospho): 94.95;S12(Phospho): 94.95	2	1.448	0.982965
[K].IQPLEPDSPTGLSENPTPATEK.[L]	1034627249	ZBTB21	49854	1034627249 [996-1017]	1xPhospho [S/T];2xTMT6plex [N-Term;K22]	S8(Phospho): 24.8;T10(Phospho): 24.8;S13(Phospho): 24.8;T17(Phospho): 24.8	4	1.313	0.982721
[R].TVNSGGSSEPSPTEVDVSR.[Q]	1370506597	FBXO38	81545	1370506597 [730-748]	1xTMT6plex [N-Term];2xPhospho [S/T]	S4(Phospho): 33.69;S7(Phospho): 33.69;S8(Phospho): 33.69;S11(Phospho): 49.41;T13(Phospho): 49.41	1	1.299	0.981664
[R].AVVVSPKEENK.[A]	150417989	CCNK	8812	150417989 [336-346]	1xPhospho [S5(100)];3xTMT6plex [N-Term;K11];K7	S5(Phospho): 100	1	1.427	0.981340
[K].NSSNTSVGSPSNTIGR.[T]	1034612655	CLASP1	23332	1034612655 [1088-1103]	1xPhospho [S/T];1xTMT6plex [N-Term]	S9(Phospho): 90.2	2	0.873	0.981124
[R].EAANEAGDSSQDEAEDDVK.[Q]	8922477	POLR3E	55718	8922477 [153-171]	2xPhospho [S9(100);2xTMT6plex [N-Term;K19];S10(100)]	S9(Phospho): 100;S10(Phospho): 100	6	1.004	0.980831
[K].RKSLSDSESDDSK.[S]	530384472	CBX3	11335	530384472 [91-103]	2xPhospho [S3(97.8);3xTMT6plex [N-Term;K13];K2;S]	S3(Phospho): 100;S5(Phospho): 99.96	1	0.894	0.980788
[R].TSSKESSPIPSPTSDR.[K]	530368012	SPTBN1	6711	530368012 [2159-2174]	2xTMT6plex [N-Term;3xPhospho [S7(98.4);K4];S/T];S11(96.7)	S2(Phospho): 49.51;S3(Phospho): 49.51;S6(Phospho): 49.51;S7(Phospho): 49.51;S11(Phospho): 94.41	1	0.941	0.978527
[R].EREAANEAGDSSQDEAEDDVK.[Q]	8922477	POLR3E	55718	8922477 [151-171]	2xPhospho [S11(100);2xTMT6plex [N-Term;K21];S12(100)]	S11(Phospho): 100;S12(Phospho): 100	1	1.04	0.978401
[K].AETSEGSGSAPAVPEASASPK.[Q]	160707950	MECP2	4204	160707950 [74-94]	1xPhospho [S19(100)];2xTMT6plex [N-Term;K21]	S19(Phospho): 100	2	1.028	0.977331
[R].GYQSLSDSPPGSR.[V]	1022436959	SEMA4B	10509	1022436959 [839-851]	1xPhospho [S8(96.7)];1xTMT6plex [N-Term]	S8(Phospho): 100	2	1.047	0.977302
[K].TQEISRPNSPSEGEGESSDSR.[S]	530360573	RERE	473	530360573 [671-691]	1xTMT6plex [N-Term];2xPhospho [S5(99.9);S9(99.9)]	S5(Phospho): 99.94;S9(Phospho): 99.94	2	1.119	0.976782
[R].ERSPALKSPLQSVVVR.[R]	167234419	THRAP3	9967	167234419 [246-261]	2xPhospho [S3(100);2xTMT6plex [N-Term;K7];S]	S3(Phospho): 100;S8(Phospho): 100	3	1.428	0.976042
[R].VPEASSEPFDTSSPQAGR.[Q]	530417965	ZHX3	23051	530417965 [934-951]	1xPhospho [S6(90)];1xTMT6plex [N-Term]	T11(Phospho): 33.33;S12(Phospho): 33.33;S13(Phospho): 33.33	10	1.017	0.975049
[K].TEMDKSPFNSPSPQDSPR.[L]	1034608029	NFIC	4782	1034608029 [328-345]	1xOxidation [M3];2xPhospho [T1(96.2);2xTMT6plex [N-Term;K5];S]	S6(Phospho): 50;S10(Phospho): 50;S12(Phospho): 98.19	1	0.758	0.974289
[R].SQSLPTTLLSPVR.[V]	194363771	ITPRID2	6744	194363771 [737-749]	1xPhospho [S3(98.6)];1xTMT6plex [N-Term]	S3(Phospho): 98.57	1	1.632	0.974244
[K].RASFAEK.[T]	530389151	PLEC	5339	530389151 [1587-1593]	1xPhospho [S3(100)];2xTMT6plex [N-Term;K7]	S3(Phospho): 100	1	1.801	0.974208
[K].ATQASQEY.[-]	4504253	H2AFX	3014	4504253 [136-143]	1xPhospho [S5(100)];1xTMT6plex [N-Term]	S5(Phospho): 100	1	2.189	0.973990
[K].LSSSPPRASPVR.[M]	767956109	USP20	10868	767956109 [405-416]	1xTMT6plex [N-Term];2xPhospho [S9(91.7);S]	S3(Phospho): 50;S4(Phospho): 50;S9(Phospho): 98.89	1	1.302	0.973789
[R].NNSVSGLSVK.[S]	256223453	DDX20	11218	256223453 [498-507]	1xPhospho [S3(100)];2xTMT6plex [N-Term;K10]	S3(Phospho): 100	2	0.584	0.973472
[K].TSPLKDNPSPEPQLDDIKR.[E]	38569495	EAF1	85403	38569495 [157-175]	2xPhospho [S9(100);3xTMT6plex [N-Term;K18];K5;S/T]	T1(Phospho): 50;S2(Phospho): 50;S9(Phospho): 100	4	0.959	0.972971
[R].HVSPVTPPREVPIYANR.[F]	1034661881	ADAM9	8754	1034661881 [758-774]	1xTMT6plex [N-Term];2xPhospho [S3(100);T6(96.8)]	S3(Phospho): 98.31;T6(Phospho): 50.84;Y14(Phospho): 50.84	1	1.864	0.972608
[R].SDGSGESAQPPEDSSPPASSESSSTR.[D]	1034673276	HUWE1	10075	1034673276 [3031-3056]	1xPhospho [S/T];1xTMT6plex [N-Term]	S14(Phospho): 49.93;S15(Phospho): 49.93	4	0.638	0.972451
[R].ASEDTTSGSPPKK.[S]	219283152	API5	8539	219283152 [456-468]	1xPhospho [T/S];3xTMT6plex [N-Term;K12;K13]	S9(Phospho): 100	3	0.812	0.972066
[R].SVGKVEPSSQSPGR.[S]	1034578688	ERC1	23085	1034578688 [7-20]	1xPhospho [S];2xTMT6plex [N-Term;K4]	S9(Phospho): 49.51;S11(Phospho): 49.51	1	0.458	0.971192
[K].LSEEAECPNPSTPSK.[A]	126362971	STK10	6793	126362971 [941-955]	1xCarbamidomethyl [C7];1xPhospho [T12(98.6)];2xTMT6plex [N-Term;K15]	T12(Phospho): 98.55	3	0.682	0.971155
[R].SAGGSSPEGGEDSDREDGNYCPPVKR.[E]	6466466	RANBP3	8498	6466466 [96-121]	1xCarbamidomethyl [C21];2xPhospho [S/Y];2xTMT6plex [N-Term;K25]	S5(Phospho): 66.52;S6(Phospho): 66.52;S13(Phospho): 66.42	3	1.069	0.970267
[R].DSENLASPSEYPENGER.[F]	1435761100	NUP98	4928	1435761100 [600-616]	1xPhospho [S/Y];1xTMT6plex [N-Term]	S7(Phospho): 33.33;S9(Phospho): 33.33;Y11(Phospho): 33.33	3	1.393	0.969977
[K].SSKASLGSLEGEAEAEASSPK.[G]	1079280806	AHNAK	79026	1079280806 [5745-5765]	2xPhospho [S];3xTMT6plex [N-Term;K21];K3	S1(Phospho): 33.35;S2(Phospho): 33.35;S5(Phospho): 33.35;S8(Phospho): 99.96	9	1.028	0.969642
[R].HAYEGSSSGNSSPEYPRK.[E]	119829187	COL17A1	1308	119829187 [107-124]	2xPhospho [S/Y];2xTMT6plex [N-Term;K18]	S6(Phospho): 97.94;S8(Phospho): 33.13;S11(Phospho): 33.13;S12(Phospho): 33.13	7	0.946	0.969144
[R].KASGSENEGDYNPGRK.[T]	1061214220	TOP2B	7155	1061214220 [1548-1563]	2xPhospho [S3(100);3xTMT6plex [N-Term;K1;K16];S5(100)]	S3(Phospho): 100;S5(Phospho): 100	2	1.305	0.969095
[R].SQSIEQESQEK.[Q]	166235169	PRPF38B	55119	166235169 [527-537]	1xPhospho [S];2xTMT6plex [N-Term;K11]	S3(Phospho): 100	3	1.006	0.968415
[R].SVSPPPK.[R]	20127499	SRSF6	6431	20127499 [314-320]	2xPhospho [S1(100);2xTMT6plex [N-Term;K7];S3(100)]	S1(Phospho): 100;S3(Phospho): 100	3	1.202	0.967916
[R].KASVVDPSTESSPAPQEGSEQPASPASPLSSR.[Q]	45597175	TBC1D9B	23061	45597175 [409-440]	2xPhospho [S3(100);2xTMT6plex [N-Term;K1];S27(96.1)]	S3(Phospho): 100;S27(Phospho): 92.76	2	1.083	0.966653
[K].SRSPGSPVGEGTGSPPK.[W]	530375995	ADD1	118	530375995 [353-369]	2xPhospho [S3(98.7);2xTMT6plex [N-Term;K17];S14(97.4)]	S3(Phospho): 98.69;S14(Phospho): 97.42	1	1.046	0.966343
[R].SRPTSEGSDIESTEPQK.[Q]	388490152	LARP7	51574	388490152 [261-277]	1xPhospho [S8(100)];2xTMT6plex [N-Term;K17]	S8(Phospho): 100	2	1.194	0.965954
[R].GTDDSPKDSQEDLQER.[H]	61175239	TCEAL5	340543	61175239 [123-138]	2xPhospho [S5(100);2xTMT6plex [N-Term;K7];S9(100)]	S5(Phospho): 99.96;S9(Phospho): 100	1	1.579	0.965615
[R].ETRSSSESLIFSGNQGR.[G]	157738639	TNS4	84951	157738639 [193-209]	1xTMT6plex [N-Term];2xPhospho [S8(75.9);T/S]	T2(Phospho): 33.57;S4(Phospho): 33.57;S5(Phospho): 32.9;S8(Phospho): 97.22	3	0.903	0.964558
[R].NHSGSRTPPVALNSSR.[M]	118572613	SRRM2	23524	118572613 [2098-2113]	1xTMT6plex [N-Term];2xPhospho [S5(98.4);T7(100)]	S5(Phospho): 98.63;T7(Phospho): 99.98	2	0.635	0.964145
[R].DAGDPSPPNK.[M]	18379328	WAC	51322	18379328 [48-57]	1xPhospho [S6(100)];2xTMT6plex [N-Term;K10]	S6(Phospho): 100	2	1.04	0.964054
[R].SSSESYTQSFQSR.[K]	13559514	PI4K2A	55361	13559514 [460-472]	1xPhospho [S/Y/T];1xTMT6plex [N-Term]	S2(Phospho): 49.65;S3(Phospho): 49.65	2	2.661	0.963105
[R].LGAGGGSPEKSPSAQELK.[E]	56181387	STUB1	10273	56181387 [13-30]	2xPhospho [S7(100);3xTMT6plex [N-Term;K10;K18];S]	S7(Phospho): 99.95;S11(Phospho): 50.02;S13(Phospho): 50.02	1	0.808	0.962611
[R].LTNLSSNSDV.[-]	262205665	TPBG	7162	262205665 [411-420]	1xPhospho [S8(98.8)];1xTMT6plex [N-Term]	S8(Phospho): 98.8	1	1.89	0.962147
[R].RQQDPSPGSNLGGGDDLK.[L]	13124881	CBFB	865	13124881 [168-185]	1xPhospho [S6(98.8)];2xTMT6plex [N-Term;K18]	S6(Phospho): 98.82	4	1.487	0.961899
[K].KADRDQSPFSK.[I]	530371189	PPIG	9360	530371189 [681-691]	1xPhospho [S7(100)];3xTMT6plex [N-Term;K1;K11]	S7(Phospho): 99.98	1	1.849	0.961074
[R].AQSPSYVISTGVSPSR.[G]	767920201	PKP4	8502	767920201 [219-234]	1xTMT6plex [N-Term];2xPhospho [S3(97.6);S/Y/T]	S3(Phospho): 97.62;S5(Phospho): 48.36;S13(Phospho): 49.36	1	0.746	0.960123
[R].GPPSPPAPVMHSPSRK.[M]	974005398	SNW1	22938	974005398 [221-236]	1xOxidation [M10];2xPhospho [S4(100);2xTMT6plex [N-Term;K16];S12(98.2)]	S4(Phospho): 100;S12(Phospho): 98.26	6	1.049	0.959886
[K].AVQEKSSSPPPR.[K]	167234419	THRAP3	9967	167234419 [528-539]	2xPhospho [S6(99.1);2xTMT6plex [N-Term;K5];S8(99.1)]	S6(Phospho): 99.06;S8(Phospho): 99.06	3	1.25	0.958819
[R].DSSFTEVPRSPK.[H]	1370457682	SVIL	6840	1370457682 [236-247]	1xPhospho [S10(98.8)];2xTMT6plex [N-Term;K12]	S10(Phospho): 99.99	1	1.869	0.958646
[R].RGSPTTGFIEQK.[G]	1034659998	FAM83H	286077	1034659998 [1091-1102]	1xPhospho [S3(100)];2xTMT6plex [N-Term;K12]	S3(Phospho): 99.98	2	1.598	0.958312
[R].ELAGNTSSPPLSPGRPSPMQR.[L]	767914691	BCL11A	53335	767914691 [321-341]	1xOxidation [M19];1xTMT6plex [N-Term];2xPhospho [T6(93.8);S]	T6(Phospho): 33.76;S7(Phospho): 33.76;S8(Phospho): 33.76;S12(Phospho): 49.35;S17(Phospho): 49.35	3	0.922	0.958271
[R].SPASPLNNQGIPTPAQLTK.[S]	218505694	KCTD1	284252	218505694 [617-635]	1xPhospho [S4(94.7)];2xTMT6plex [N-Term;K19]	S4(Phospho): 98.71	2	0.852	0.958127
[K].TSAAACAVTDLSDDSDFDEKAK.[L]	9966799	UTP3	57050	9966799 [354-375]	1xCarbamidomethyl [C6];2xPhospho [S15(87.4);3xTMT6plex [N-Term;K20;K22];S/T]	S12(Phospho): 99.95;S15(Phospho): 99.95	1	1.331	0.956996
[R].LDYVPSSASR.[E]	1034659998	FAM83H	286077	1034659998 [709-718]	1xTMT6plex [N-Term];2xPhospho [S9(99.2);S/Y]	S6(Phospho): 50.46;S7(Phospho): 50.46;S9(Phospho): 99.07	2	1.684	0.956823
[K].AVSPPHLDGPPSPRSPVIGSEVFLPNSNHVASGAGEAEER.[V]	67089149	PML	5371	67089149 [516-555]	1xTMT6plex [N-Term];3xPhospho [S3(100);S]	S3(Phospho): 100;S12(Phospho): 99.86;S15(Phospho): 48.87;S20(Phospho): 48.87	6	1.023	0.956685
[K].RESEDDPESEPDDHEK.[R]	530368018	GCFC2	6936	530368018 [180-195]	2xPhospho [S3(100);2xTMT6plex [N-Term;K16];S9(100)]	S3(Phospho): 100;S9(Phospho): 100	1	0.437	0.956600
[R].ESGVVAVSPEKSESPQKEDGLSSQLK.[S]	14790190	SPEN	23013	14790190 [2113-2138]	2xPhospho [S];4xTMT6plex [N-Term;K11;K17;K26]	S8(Phospho): 64.36;S12(Phospho): 64.36;S14(Phospho): 64.36	3	0.718	0.956580
[R].GSGTASDDEFENLR.[I]	1034673276	HUWE1	10075	1034673276 [2029-2042]	1xPhospho [S6(99.1)];1xTMT6plex [N-Term]	S6(Phospho): 99.99	4	0.88	0.956559
[R].AAAAAAAAAAAPPAPPEGASPGDSARQKLEETSACLAAALQAVEEK.[I]	18079216	CASKIN1	57524	18079216 [1345-1390]	1xCarbamidomethyl [C35];2xPhospho [S/T];3xTMT6plex [N-Term;K28;K46]	S20(Phospho): 50;S24(Phospho): 50;T32(Phospho): 50;S33(Phospho): 50	4	2.152	0.956532
[R].AEQPSPPNSDSGQDAHPDPDANPDAAR.[M]	767967520	SPINDOC	144097	767967520 [144-170]	1xTMT6plex [N-Term];2xPhospho [S5(98.7);S]	S5(Phospho): 98.7;S9(Phospho): 50.65;S11(Phospho): 50.65	3	0.984	0.956086
[R].SITSPPSTSTTK.[R]	530414871	NFIX	4784	530414871 [324-335]	2xPhospho [S1(89.5);2xTMT6plex [N-Term;K12];T/S]	S1(Phospho): 99.95;S4(Phospho): 96.03	1	1.567	0.955890
[K].ILLVDSPGMGNADDEQQEEGTSSK.[Q]	156119605	GTF3C4	9329	156119605 [606-629]	1xOxidation [M9];1xPhospho [S6(100)];2xTMT6plex [N-Term;K24]	S6(Phospho): 100	5	1.034	0.955884
[R].VNLEESSGVENSPAGARPK.[R]	33469966	SCFD1	23256	33469966 [292-310]	1xPhospho [S];2xTMT6plex [N-Term;K19]	S6(Phospho): 33.33;S7(Phospho): 33.33;S12(Phospho): 33.33	3	1.478	0.955851
[R].SRSLGSSPVK.[-]	256773222	KIAA1551	55196	256773222 [1738-1747]	2xPhospho [S3(99.1);2xTMT6plex [N-Term;K10];S]	S3(Phospho): 99.13;S6(Phospho): 50;S7(Phospho): 50	2	1.593	0.955471
[R].MEREDSSEEEEEEIDDEEIERR.[R]	50726968	MFAP1	4236	50726968 [127-148]	1xOxidation [M1];1xTMT6plex [N-Term];2xPhospho [S6(100);S7(100)]	S6(Phospho): 100;S7(Phospho): 100	2	1.09	0.954505
[R].SRSPLELEPEAK.[K]	4557515	DDB2	1643	4557515 [24-35]	2xPhospho [S1(100);2xTMT6plex [N-Term;K12];S3(100)]	S1(Phospho): 100;S3(Phospho): 100	2	1.23	0.954472
[K].EAEDSDSDDNIK.[R]	530369278	IWS1	55677	530369278 [512-523]	2xPhospho [S5(100);2xTMT6plex [N-Term;K12];S7(100)]	S5(Phospho): 100;S7(Phospho): 100	2	0.626	0.953845
[R].AYLGYQAVPSGSSGSLSTSSSSPPGTPSPADAK.[A]	28872796	CEBPB	1051	28872796 [209-242]	1xPhospho [T/S/Y];2xTMT6plex [N-Term;K34]	T27(Phospho): 76.49	21	0.684	0.953752
[R].AETASQSQRSPISDNSGCDAPGNSNPSLSVPSSAESEK.[Q]	767975924	SCAF11	9169	767975924 [345-382]	1xCarbamidomethyl [C18];1xPhospho [S/T];2xTMT6plex [N-Term;K38]	S5(Phospho): 33.07;S7(Phospho): 33.07;S10(Phospho): 33.07	15	1.459	0.953341
[R].IGSPLSPK.[K]	1034640316	LARP1B	55132	1034640316 [539-546]	2xPhospho [S3(100);2xTMT6plex [N-Term;K8];S6(100)]	S3(Phospho): 100;S6(Phospho): 100	1	0.511	0.953169
[R].RSTSPIIGSPPVR.[A]	189217919	PATL1	219988	189217919 [176-188]	1xTMT6plex [N-Term];2xPhospho [S9(100);T/S]	T3(Phospho): 49.6;S4(Phospho): 49.6;S9(Phospho): 100	4	0.675	0.953127
[R].RSPSLSSK.[H]	1034554585	SRRM1	10250	1034554585 [691-698]	2xPhospho [S2(100);2xTMT6plex [N-Term;K8];S4(98.3)]	S2(Phospho): 100;S4(Phospho): 98.47	3	0.983	0.952841
[R].YRSPYSGPK.[F]	35493811	RBM39	9584	35493811 [95-103]	2xPhospho [Y/S];2xTMT6plex [N-Term;K9]	S3(Phospho): 98.65;Y5(Phospho): 50;S6(Phospho): 50	2	1.23	0.952783
[R].VEQMPQASPGLAPR.[T]	530420479	MICALL1	85377	530420479 [571-584]	1xOxidation [M4];1xPhospho [S8(100)];1xTMT6plex [N-Term]	S8(Phospho): 100	4	1.027	0.950144
[K].AVDSSSEEIEVEVPVVDRR.[N]	530379249	FAM169A	26049	530379249 [632-650]	1xTMT6plex [N-Term];2xPhospho [S4(100);S5(98.3)]	S4(Phospho): 99.98;S5(Phospho): 98.66	4	0.905	0.950041
[K].AAQQAASSSGQGQQAQTPTGK.[Q]	68303575	CSNK1A1	1452	68303575 [333-353]	1xPhospho [T17(97.7)];2xTMT6plex [N-Term;K21]	T17(Phospho): 100	20	0.961	0.949968
[R].LLESSLSSSEGEEPVEYK.[S]	1017029567	ITGA5	3678	1017029567 [120-137]	1xPhospho [S/Y];2xTMT6plex [N-Term;K18]	S7(Phospho): 33.21;S8(Phospho): 33.21;S9(Phospho): 33.21	9	0.977	0.949944
[R].RRSPSPYYSR.[GY]	9558733	TRA2A	29896	9558733 [258-267]	1xTMT6plex [N-Term];2xPhospho [S3(100);S5(96.9)]	S3(Phospho): 100;S5(Phospho): 100	5	0.94	0.949498
[K].SDSQASLTVPLSPQVVR.[S]	146260268	SMCR8	140775	146260268 [487-503]	1xTMT6plex [N-Term];2xPhospho [S/T]	S1(Phospho): 50;S3(Phospho): 50;S6(Phospho): 97.79	1	0.779	0.949289
[R].AESGPDLR.[Y]	58530840	DSP	1832	58530840 [20-27]	1xPhospho [S3(100)];1xTMT6plex [N-Term]	S3(Phospho): 100	1	1.03	0.948818
[R].SVGGSGGGSFGDNLVTR.[S]	27436946	LMNA	4000	27436946 [628-644]	1xPhospho [S/T];1xTMT6plex [N-Term]	S1(Phospho): 49.97;S5(Phospho): 49.97	2	1.067	0.947751
[K].SSSISEEKGDSDDEKPR.[K]	7662238	ACIN1	22985	7662238 [206-222]	1xPhospho [S];3xTMT6plex [N-Term;K15];K8	S11(Phospho): 99.98	1	1.481	0.947749
[K].TVSSPPTSPRPGSAATVSASTSNIIPPR.[H]	109638749	SRP72	6731	109638749 [618-645]	1xTMT6plex [N-Term];2xPhospho [S/T]	S3(Phospho): 49.49;S4(Phospho): 49.49;T7(Phospho): 33.66;S8(Phospho): 33.66;S13(Phospho): 33.66	12	0.741	0.947519
[R].RPDPDSDEDEDYER.[E]	224549005	RBM17	84991	224549005 [150-163]	1xPhospho [S6(100)];1xTMT6plex [N-Term]	S6(Phospho): 100	3	1.054	0.947428
[K].GYYSPYSVSGSGSTAGSRTGSR.[T]	530389151	PLEC	5339	530389151 [4478-4499]	1xTMT6plex [N-Term];2xPhospho [S/T/Y]	S7(Phospho): 32.4;S9(Phospho): 32.4;S11(Phospho): 32.6;S13(Phospho): 20.32;T14(Phospho): 20.32;S17(Phospho): 20.32;T19(Phospho): 20.32;S21(Phospho): 20.32	5	0.965	0.945953
[K].YSDEQLNSGRQSPSQNER.[W]	1237938059	APC	324	1237938059 [1049-1066]	1xPhospho [S8(98.4)];1xTMT6plex [N-Term]	S8(Phospho): 99.97	2	0.829	0.945913
[K].AEAPSSPDVAPAGKEDSPSASGR.[V]	557440888	PHRF1	57661	557440888 [1355-1377]	2xPhospho [S];2xTMT6plex [N-Term;K14]	S6(Phospho): 97.72;S17(Phospho): 97.66	3	1.416	0.945532
[R].SRSLSPICPR.[S]	21687119	SPATA18	132671	21687119 [505-514]	1xCarbamidomethyl [C8];1xTMT6plex [N-Term];2xPhospho [S5(100);S]	S1(Phospho): 50;S3(Phospho): 50;S5(Phospho): 100	1	0.802	0.944922
[R].VKDSSPGEK.[S]	767975924	SCAF11	9169	767975924 [914-922]	1xPhospho [S5(99.3)];3xTMT6plex [N-Term;K2;K9]	S5(Phospho): 99.34	1	1.651	0.944901
[K].KAQEAEAQSEDDDEDTEEEQGEEKEK.[G]	1214884389	NOM1	64434	1214884389 [272-297]	2xPhospho [S9(100);4xTMT6plex [N-Term;K1;K24;K26];T16(100)]	S9(Phospho): 100;T16(Phospho): 100	1	1.301	0.944259
[R].IEVLPVDTGAGGYSGNSGSPK.[N]	578818661	USP6NL	9712	578818661 [726-746]	1xPhospho [Y/S/T];2xTMT6plex [N-Term;K21]	S19(Phospho): 97.89	7	0.696	0.944096
[R].AISAPTSPTR.[L]	752507721	MIEF1	54471	752507721 [53-62]	1xTMT6plex [N-Term];2xPhospho [S3(100);S7(98.6)]	S3(Phospho): 100;S7(Phospho): 99.98	2	1.593	0.943709
[R].GESPPTPPGQPPISPK.[K]	64085167	INPP5D	3635	64085167 [958-973]	2xPhospho [T6(98);2xTMT6plex [N-Term;K16];S14(100)]	T6(Phospho): 98.03;S14(Phospho): 100	2	0.872	0.943379
[R].TSSKESSPIPSPTSDRK.[A]	530368012	SPTBN1	6711	530368012 [2159-2175]	2xPhospho [S/T];3xTMT6plex [N-Term;K17];K4	S6(Phospho): 48.58;S7(Phospho): 48.58;S11(Phospho): 33.33;T13(Phospho): 33.33;S14(Phospho): 33.33	1	1.935	0.942505
[R].LARSSPEQPRPSTSK.[A]	67089149	PML	5371	67089149 [501-515]	2xPhospho [S/T];2xTMT6plex [N-Term;K15]	S5(Phospho): 96.67;S12(Phospho): 49.15;T13(Phospho): 49.15	2	1.64	0.941736
[R].SPVSTRPLPSASQK.[A]	530416134	LSM14A	26065	530416134 [216-229]	1xPhospho [S1(100)];2xTMT6plex [N-Term;K14]	S1(Phospho): 100	1	0.561	0.941424
[R].FVSEGDGGR.[L]	21735596	PDCD4	27250	21735596 [455-463]	1xPhospho [S3(100)];1xTMT6plex [N-Term]	S3(Phospho): 100	2	1.028	0.941392
[R].LMRSSTDSLPGPISR.[Q]	15147335	NCOA5	57727	15147335 [374-388]	1xOxidation [M2];1xTMT6plex [N-Term];2xPhospho [S8(97.3);S/T]	S4(Phospho): 49.37;S5(Phospho): 49.37;S8(Phospho): 97.34	1	1.54	0.941316
[R].NSSTPGLQVPVSPTVPIQNQK.[Y]	767970167	KMT2A	4297	767970167 [3061-3081]	2xPhospho [T14(97.4);2xTMT6plex [N-Term;K21];S/T]	S3(Phospho): 64.96;T4(Phospho): 64.96;T14(Phospho): 64.96	5	1.446	0.941028
[K].GKASPFEEDQNR.[D]	530367033	PUM2	23369	530367033 [133-144]	1xPhospho [S4(100)];2xTMT6plex [N-Term;K2]	S4(Phospho): 100	2	0.863	0.940861
[K].VDNLTYRTSPDTLR.[R]	47271443	SRSF2	6427	47271443 [18-31]	1xPhospho [T/Y/S];1xTMT6plex [N-Term]	T5(Phospho): 24.92;Y6(Phospho): 24.92;T8(Phospho): 24.92;S9(Phospho): 24.92	4	0.929	0.940495
[R].SSSPVTELASRSPIRQDR.[G]	118572613	SRRM2	23524	118572613 [1101-1118]	1xTMT6plex [N-Term];2xPhospho [S/T]	S3(Phospho): 97.28;T6(Phospho): 97.36	2	2.082	0.938521
[R].NLSSTTDDEAPR.[L]	20070205	LAD1	3898	20070205 [36-47]	1xPhospho [S3(100)];1xTMT6plex [N-Term]	S3(Phospho): 100	1	3.274	0.937969
[R].QGDDSSDEEDKER.[V]	20149560	STX4	6810	20149560 [10-22]	2xPhospho [S5(100);2xTMT6plex [N-Term;K11];S6(100)]	S5(Phospho): 100;S6(Phospho): 100	1	2.289	0.936626
[R].KAPPPPISPTQLSDVSSPR.[S]	1034644516	RAI14	26064	1034644516 [280-298]	2xPhospho [S/T];2xTMT6plex [N-Term;K1]	S8(Phospho): 49.54;T10(Phospho): 49.54;S13(Phospho): 33.64;S16(Phospho): 33.64;S17(Phospho): 33.64	7	1.44	0.935514
[K].SLSDSESDDSKSK.[K]	530384472	CBX3	11335	530384472 [93-105]	2xPhospho [S3(96.7);3xTMT6plex [N-Term;K11;K13];S]	S3(Phospho): 100;S5(Phospho): 50;S7(Phospho): 50	1	1.048	0.935213
[R].SSSSPTEATEK.[N]	110624774	MRC2	9902	110624774 [1455-1465]	1xPhospho [S2(95.9)];2xTMT6plex [N-Term;K11]	S2(Phospho): 95.86	3	2.047	0.935087
[R].ADSGPTQPPLSLSPAPETK.[R]	530426853	SEC16A	9919	530426853 [2071-2089]	2xPhospho [S/T];2xTMT6plex [N-Term;K19]	S11(Phospho): 66.64;S13(Phospho): 66.64;T18(Phospho): 66.64	1	0.922	0.935039
[R].RGEGDAPFSEPGTTSTQRPSSPETATK.[Q]	118572613	SRRM2	23524	118572613 [303-329]	2xPhospho [T26(90.2);2xTMT6plex [N-Term;K27];S/T]	T13(Phospho): 25.44;T14(Phospho): 25.44;S15(Phospho): 24.88;T16(Phospho): 24.88;S21(Phospho): 94.62	14	0.963	0.933533
[K].NSSPAPPQPAPGK.[V]	157817023	CDK12	51755	157817023 [1081-1093]	1xPhospho [S];2xTMT6plex [N-Term;K13]	S2(Phospho): 50;S3(Phospho): 50	2	0.696	0.932509
[R].SLSSPPSSK.[R]	530389915	NFIB	4781	530389915 [262-270]	2xPhospho [S3(98.5);2xTMT6plex [N-Term;K9];S4(98.5)]	S3(Phospho): 99.98;S4(Phospho): 99.98	1	0.916	0.932309
[K].SSTSFANIQENSN.[-]	30025026	OSTM1	28962	30025026 [322-334]	1xPhospho [S/T];1xTMT6plex [N-Term]	T3(Phospho): 49.74;S4(Phospho): 49.74	3	1.086	0.932289
[R].RPSASSPNNNTAAK.[G]	530391243	PHF2	5253	530391243 [1055-1068]	2xPhospho [S3(100);2xTMT6plex [N-Term;K14];S5(98.5)]	S3(Phospho): 100;S5(Phospho): 98.5	2	1.493	0.932255
[K].RENPPVEDSSDEDDKR.[N]	30578410	STT3B	201595	30578410 [490-505]	2xPhospho [S9(100);2xTMT6plex [N-Term;K15];S10(100)]	S9(Phospho): 100;S10(Phospho): 100	2	1.017	0.932055
[K].NSSQSGGKPGSSPITK.[H]	28559039	MED1	5469	28559039 [1145-1160]	1xPhospho [S/T];3xTMT6plex [N-Term;K16];K8	S2(Phospho): 24.89;S3(Phospho): 24.89;S11(Phospho): 24.89;S12(Phospho): 24.89	2	0.876	0.931269
[K].NNTAAETEDDESDGEDRGGGTSGSLR.[R]	47578105	NIPBL	25836	47578105 [2661-2686]	1xTMT6plex [N-Term];2xPhospho [T/S]	T7(Phospho): 99.9;S12(Phospho): 100	2	1.671	0.931171
[K].RKTSSDDESEEDEDDLLQR.[T]	118344456	UTP18	51096	118344456 [202-220]	2xPhospho [S9(100);2xTMT6plex [N-Term;K2];T/S]	T3(Phospho): 33.33;S4(Phospho): 33.33;S5(Phospho): 33.33;S9(Phospho): 100	2	1.059	0.931120
[K].YGLQDSDEEEEEHPSK.[T]	1435761100	NUP98	4928	1435761100 [866-881]	1xPhospho [S6(97.9)];2xTMT6plex [N-Term;K16]	S6(Phospho): 100	3	1.072	0.931111
[R].TLSDPPSPLPHGPPNK.[G]	578815980	ASAP1	50807	578815980 [840-855]	2xPhospho [S3(98.6);2xTMT6plex [N-Term;K16];S7(100)]	S3(Phospho): 99.95;S7(Phospho): 100	2	0.897	0.929908
[R].GTAQYSSQKSVEER.[S]	148664226	PKP2	5318	148664226 [126-139]	1xPhospho [S10(99)];2xTMT6plex [N-Term;K9]	S10(Phospho): 98.99	1	0.775	0.928752
[R].SSFASSSASDASKPSSPR.[G]	14249622	AJUBA	84962	14249622 [122-139]	2xPhospho [S];2xTMT6plex [N-Term;K13]	S9(Phospho): 98.97;S12(Phospho): 33.97;S15(Phospho): 33.5;S16(Phospho): 33.5	1	0.558	0.928038
[K].GVPEKSPVLEK.[S]	20070205	LAD1	3898	20070205 [172-182]	1xPhospho [S6(100)];3xTMT6plex [N-Term;K11];K5	S6(Phospho): 100	1	1.874	0.928032
[R].VGDTEKPEPERSPPNR.[K]	7706557	C9orf78	51759	7706557 [250-265]	1xPhospho [S12(100)];2xTMT6plex [N-Term;K6]	S12(Phospho): 100	2	0.632	0.927112
[K].DLDRPESQSPK.[R]	530367499	GTF3C2	2976	530367499 [198-208]	1xPhospho [S9(98.6)];2xTMT6plex [N-Term;K11]	S9(Phospho): 98.57	1	1.296	0.926536
[R].QQAAYYAQTSPQGMPQHPPAPQGQ.[-]	742670570	FUBP1	8880	742670570 [642-665]	1xOxidation [M14];1xPhospho [Y/T/S];1xTMT6plex [N-Term]	T9(Phospho): 49.99;S10(Phospho): 49.99	4	1.258	0.926375
[R].VSDSESEGPQKGPASDSETEDASR.[H]	530369278	IWS1	55677	530369278 [304-327]	2xPhospho [S/T];2xTMT6plex [N-Term;K11]	S15(Phospho): 91.67;S17(Phospho): 49.83;T19(Phospho): 49.83	1	0.948	0.926038
[K].AAAAPASEDEDDEDDEDDEDDDDDEEDDSEEEAMETTPAK.[G]	55956788	NCL	4691	55956788 [177-217]	1xOxidation [M35];2xTMT6plex [N-Term;3xPhospho [T38(94.5);K41];S/T]	S8(Phospho): 51.18;S30(Phospho): 51.18;T37(Phospho): 97.75;T38(Phospho): 99.88	10	0.815	0.924488
[R].ILIPSDIESSKSSTR.[R]	1370484230	NKTR	4820	1370484230 [459-473]	2xPhospho [S5(100);2xTMT6plex [N-Term;K11];S/T]	S5(Phospho): 100;S9(Phospho): 24.87;S10(Phospho): 24.87;S12(Phospho): 24.87;S13(Phospho): 24.87	1	1.053	0.924041
[R].YRRSPSPYYSR.[Y]	544346329	TRA2A	29896	544346329 [155-165]	1xTMT6plex [N-Term];2xPhospho [S4(98.1);S6(98)]	S4(Phospho): 99.97;S6(Phospho): 100	2	1.222	0.923382
[R].SFSASQSTDREGASPVTEVR.[I]	1034627249	ZBTB21	49854	1034627249 [409-428]	1xTMT6plex [N-Term];2xPhospho [S3(98.4);S14(100)]	S3(Phospho): 97.82;S14(Phospho): 99.95	7	1.373	0.923272
[K].AGEAPTENPAPPTQQSSAE.[-]	224586882	YBX3	8531	224586882 [354-372]	1xPhospho [T/S];1xTMT6plex [N-Term]	T13(Phospho): 33.33;S16(Phospho): 33.33;S17(Phospho): 33.33	3	0.78	0.922910
[K].SLQKVDEEDSDEESHHDEMSEQEEELEDDPTVVK.[N]	1034650453	C6orf203	51250	1034650453 [127-160]	1xOxidation [M19];2xPhospho [S/T];3xTMT6plex [N-Term;K34];K4	S10(Phospho): 100;S20(Phospho): 96.76	3	0.92	0.921995
[K].ARSVSPPPKR.[A]	20127499	SRSF6	6431	20127499 [312-321]	2xPhospho [S3(100);2xTMT6plex [N-Term;K9];S5(100)]	S3(Phospho): 100;S5(Phospho): 100	3	0.971	0.921587
[R].EGSTQQLQTTSPK.[V]	767977944	RBM26	64062	767977944 [621-633]	1xPhospho [T/S];2xTMT6plex [N-Term;K13]	T9(Phospho): 33.33;T10(Phospho): 33.33;S11(Phospho): 33.33	3	1.448	0.920702
[R].VTSFSTPPTPER.[N]	57863293	KIAA0930	23313	57863293 [290-301]	1xTMT6plex [N-Term];2xPhospho [T9(100);T/S]	S3(Phospho): 49.63;S5(Phospho): 49.63;T9(Phospho): 98.5	4	0.923	0.920630
[R].SQTTTERDSDTDVEEEELPVENR.[E]	530382821	MDC1	9656	530382821 [466-488]	1xTMT6plex [N-Term];2xPhospho [S9(93.8);T11(99.7)]	S9(Phospho): 93.75;T11(Phospho): 99.71	2	0.904	0.920216
[K].MALPPQEDATASPPR.[Q]	118572613	SRRM2	23524	118572613 [1168-1182]	1xOxidation [M1];1xPhospho [T/S];1xTMT6plex [N-Term]	S12(Phospho): 98.96	2	0.791	0.920098
[R].RVSQNSLGEMTK.[L]	1149123001	KIDINS220	57498	1149123001 [881-892]	1xOxidation [M10];2xPhospho [S3(100);2xTMT6plex [N-Term;K12];S6(100)]	S3(Phospho): 100;S6(Phospho): 100	2	0.828	0.919765
[R].AASSSSPGSPVASSPSR.[L]	578819967	GBF1	8729	578819967 [1810-1826]	1xTMT6plex [N-Term];2xPhospho [S]	S5(Phospho): 99.96;S9(Phospho): 99.98	2	1.016	0.919529
[R].AGAAAGDSDEESRADDK.[G]	1034608146	ZBTB7A	51341	1034608146 [380-396]	1xPhospho [S8(100)];2xTMT6plex [N-Term;K17]	S8(Phospho): 100	2	1.199	0.918726
[R].SPSDSSTASTPVAEQIER.[A]	1034643898	DBN1	1627	1034643898 [385-402]	1xTMT6plex [N-Term];2xPhospho [S/T]	S5(Phospho): 39.66;S6(Phospho): 39.66;T7(Phospho): 39.66;S9(Phospho): 39.66;T10(Phospho): 39.66	4	2.448	0.918315
[R].APSPAPRPVPGSPAR.[D]	1370455393	CROCC	9696	1370455393 [1583-1597]	1xTMT6plex [N-Term];2xPhospho [S3(100);S12(100)]	S3(Phospho): 100;S12(Phospho): 100	1	1.384	0.918129
[R].MSCFSRPSMSPTPLDR.[C]	118572613	SRRM2	23524	118572613 [2114-2129]	1xCarbamidomethyl [C3];1xTMT6plex [N-Term];2xOxidation [M1;2xPhospho [S8(96.2);M9];S10(96.3)]	S2(Phospho): 91.12;S5(Phospho): 91.12	2	1.631	0.918057
[K].LRSPRGSPDGSLQTGKPSAPK.[K]	503774453	PELP1	27043	503774453 [525-545]	2xPhospho [T14(97);3xTMT6plex [N-Term;K16;K21];S]	S3(Phospho): 99.9;S7(Phospho): 97.12	1	1.025	0.917313
[K].AGLESGAEPGDGDSDTTKK.[K]	4503337	DKC1	1736	4503337 [481-499]	2xPhospho [S5(100);3xTMT6plex [N-Term;K18;K19];S14(95.9)]	S5(Phospho): 100;S14(Phospho): 96.14	2	1.326	0.917252
[K].LKAESISEEADSEPGR.[S]	16445436	BRWD1	54014	16445436 [1782-1797]	2xPhospho [S7(98.2);2xTMT6plex [N-Term;K2];S12(100)]	S7(Phospho): 98.16;S12(Phospho): 100	1	1.063	0.916696
[K].DSNELSDSAGEEDSADLKR.[A]	768023947	MORC2	22880	768023947 [777-795]	2xPhospho [S6(93.1);2xTMT6plex [N-Term;K18];S8(93.1)]	S6(Phospho): 97.02;S8(Phospho): 97.02	1	1.241	0.916368
[R].RLSSASTGKPPLSVEDDFEK.[L]	767911028	ZC3H11A	9877	767911028 [756-775]	2xPhospho [S3(98.2);3xTMT6plex [N-Term;K20];K9;S6(96.2)]	S3(Phospho): 98.22;S6(Phospho): 95.03	5	1.227	0.916140
[R].NSSNTLPIPK.[K]	119829187	COL17A1	1308	119829187 [178-187]	1xPhospho [S/T];2xTMT6plex [N-Term;K10]	T5(Phospho): 99.99	6	0.969	0.915781
[K].SSSKDSRPSQAAGDNQGDEAK.[E]	167234419	THRAP3	9967	167234419 [182-202]	1xPhospho [S];3xTMT6plex [N-Term;K21];K4	S1(Phospho): 32.93;S2(Phospho): 32.93;S3(Phospho): 32.93	4	0.999	0.915740
[K].SPSVPKSPTPKSPPSR.[K]	1337306379	PARVA	55742	1337306379 [8-23]	2xPhospho [S7(100);3xTMT6plex [N-Term;K11];K6;T/S]	S7(Phospho): 100;T9(Phospho): 49.99;S12(Phospho): 49.99	2	1.157	0.913849
[K].KVEEEQEADEEDVSEEEAESK.[E]	151101292	TMX1	81542	151101292 [234-254]	1xPhospho [S14(99.9)];3xTMT6plex [N-Term;K1;K21]	S14(Phospho): 100	1	1.499	0.912908
[R].IKTEPSSPLSDPSDIIR.[V]	1034627249	ZBTB21	49854	1034627249 [429-445]	2xPhospho [T3(99);2xTMT6plex [N-Term;K2];S7(94.5)]	T3(Phospho): 100;S6(Phospho): 50;S7(Phospho): 50	2	1.559	0.912224
[R].LQRLPSSSSEMGSQDGSPLR.[E]	109148539	GGT7	2686	109148539 [67-86]	1xOxidation [M11];1xPhospho [S6(91.3)];1xTMT6plex [N-Term]	S6(Phospho): 91.29	1	1.86	0.911626
[R].KQQQEPTGEPSPK.[R]	4504431	HMGA2	8091	4504431 [34-46]	1xPhospho [S11(98.1)];3xTMT6plex [N-Term;K1;K13]	S11(Phospho): 99.98	5	1.657	0.911389

Patil et al,2020. Proteomic and phosphoproteomic profiling of shammah-induced signaling in oral keratinocytes

Supplementary Table S2: List of phosphopeptides identified and quantified in OKF6/TERT1-Shammah and OKF6/TERT1-Parental phosphoproteome using TMT/TiO_2-based quantitative phosphoproteomic approach

Peptide Sequence	Protein Accessions	Gene Symbol	Gene ID	Positions in Proteins	Modifications	PhosphoRS: Best Site Probabilities	# PSMs	Fold change	p-value
[R].KADGEDAGAESNEEAPGEPSAGSSEEAPGEPSAGSSEEAPGER.[S]	154759257	DDX51	317781	154759257 [93-135]	1xPhospho [S];2xTMT6plex [N-Term;K1]	S11(Phospho): 100	5	1.015	0.911150
[R].LSSLRASTSK.[S]	17158044	RPS6	6194	17158044 [234-243]	2xPhospho [S3(98.2);2xTMT6plex [N-Term;K10];S7(98.2)]	S3(Phospho): 100;S7(Phospho): 98.84	2	1.68	0.909886
[K].IPSEMPSPNFGK.[N]	33354281	SLC6A15	55117	33354281 [681-692]	1xOxidation [M5];1xPhospho [S];2xTMT6plex [N-Term;K12]	S7(Phospho): 100	1	1.508	0.909863
[R].ASSRVSGSFPEDSSK.[E]	170932494	STX7	8417	170932494 [124-138]	2xPhospho [S];2xTMT6plex [N-Term;K15]	S2(Phospho): 50.3;S3(Phospho): 50.3;S6(Phospho): 99.39	1	1.45	0.909498
[R].KNSSTDQGSDEEGSLQK.[E]	101943240	GTF3C1	2975	101943240 [1060-1076]	2xPhospho [S3(94);3xTMT6plex [N-Term;K1;K17];S9(95.4)]	S3(Phospho): 33.89;S4(Phospho): 33.89;T5(Phospho): 33.89;S9(Phospho): 96.61	1	1.364	0.909350
[R].VSGRTSPPLLDR.[A]	118572613	SRRM2	23524	118572613 [2393-2404]	1xPhospho [S6(98.7)];1xTMT6plex [N-Term]	S6(Phospho): 98.58	3	1.842	0.908909
[R].IGDSSQGDNNLQK.[L]	19718766	UBE3A	7337	19718766 [214-226]	1xPhospho [S4(99.1)];2xTMT6plex [N-Term;K13]	S4(Phospho): 99.11	4	1.483	0.908454
[K].SLSDSESDDSK.[S]	530384472	CBX3	11335	530384472 [93-103]	1xPhospho [S];2xTMT6plex [N-Term;K11]	S3(Phospho): 100	2	0.691	0.908439
[K].GEIKDSSPSSSPSPK.[G]	289547524	RAB11FIP1	80223	289547524 [333-347]	2xPhospho [S6(98.5);3xTMT6plex [N-Term;K15];K4;S13(98.4)]	S6(Phospho): 98.51;S13(Phospho): 98.43	1	1.454	0.908325
[K].FMEQSRSPSVSPSKQPVSTSSK.[T]	62865871	VPS50	55610	62865871 [488-509]	1xOxidation [M2];2xPhospho [S/T];3xTMT6plex [N-Term;K14;K22]	S5(Phospho): 50;S7(Phospho): 50;S9(Phospho): 49.2;S11(Phospho): 49.2	1	0.42	0.908146
[K].LPASPSGSEDLSSVSSSPTSSPK.[T]	767918807	KANSL3	55683	767918807 [520-542]	2xPhospho [S4(96.9);2xTMT6plex [N-Term;K23];S/T]	S4(Phospho): 96.87;S12(Phospho): 31.66;S13(Phospho): 31.66;S15(Phospho): 31.66	9	1.205	0.907565
[R].VKVDGPRSPSYGR.[S]	5902076	SRSF1	6426	5902076 [192-204]	2xPhospho [S8(100)];2xTMT6plex [N-Term;K2]	S8(Phospho): 100;S10(Phospho): 98.78	2	1.21	0.907450
[R].RPIERSQSPVHLR.[R]	14790190	SPEN	23013	14790190 [720-732]	1xTMT6plex [N-Term];2xPhospho [S6(100);S8(100)]	S6(Phospho): 100;S8(Phospho): 100	1	0.654	0.907319
[R].RNSLTGEEGQLAR.[V]	13540569	SGPP1	81537	13540569 [110-122]	1xPhospho [S3(100)];1xTMT6plex [N-Term]	S3(Phospho): 100	2	0.95	0.907263
[K].GADNDGSGSESGYTTPK.[K]	32698730	NUFIP2	57532	32698730 [206-222]	1xPhospho [S7(97.8)];2xTMT6plex [N-Term;K17]	S7(Phospho): 97.83	10	0.994	0.907165
[K].IAAPELHKGDSDSEEDEPTKK.[K]	530365024	PRCC	5546	530365024 [147-167]	2xPhospho [S11(100);4xTMT6plex [N-Term;K20;K21];K8;S13(100)]	S11(Phospho): 99.95;S13(Phospho): 99.95	2	0.694	0.906840
[R].MGGAGGEGSDDDTSLT.[-]	116812600	STARD10	10809	116812600 [276-291]	1xOxidation [M1];1xPhospho [T/S];1xTMT6plex [N-Term]	S14(Phospho): 48.9;T16(Phospho): 48.9	6	0.881	0.906394
[K].NDKSEEEQSSSSVK.[K]	117190192	HNRNPC	3183	117190192 [230-243]	2xPhospho [S];3xTMT6plex [N-Term;K14];K3	S4(Phospho): 99.98;S9(Phospho): 49.67;S10(Phospho): 49.68	1	0.893	0.905760
[K].NMPNKQPESSL.[-]	52352803	SLC30A1	7779	52352803 [497-507]	1xOxidation [M2];1xPhospho [S10(98.5)];2xTMT6plex [N-Term;K5]	S10(Phospho): 98.78	1	0.923	0.905493
[R].GESAPTLSTSPSPSSPSPTSPSPTLGR.[R]	110681719	TMEM245	23731	110681719 [311-337]	1xTMT6plex [N-Term];2xPhospho [S22(97.8);S/T]	S17(Phospho): 49.43;T19(Phospho): 49.43;S22(Phospho): 97.85	5	1.035	0.904979
[R].SRSPSSPELNNK.[C]	118572613	SRRM2	23524	118572613 [1497-1508]	2xPhospho [S1(100);2xTMT6plex [N-Term;K12];S3(100)]	S1(Phospho): 100;S3(Phospho): 100	1	1.733	0.903277
[R].KRSPSPSPTPEAK.[K]	1057867268	SMARCC2	6601	1057867268 [300-312]	2xPhospho [S3(98.1);3xTMT6plex [N-Term;K1;K13];S/T]	S3(Phospho): 100;S5(Phospho): 97.24	2	0.953	0.903178
[R].TPESQPDTPPGTPLVSQDEK.[R]	31543419	PNKP	11284	31543419 [111-130]	1xPhospho [T8(97.5)];2xTMT6plex [N-Term;K20]	T8(Phospho): 97.52	2	0.517	0.902993
[R].SPGSPVGEGTGSPPK.[W]	530375995	ADD1	118	530375995 [355-369]	1xPhospho [S4(93.7)];2xTMT6plex [N-Term;K15]	S4(Phospho): 98.61	5	0.959	0.902980
[R].RGFSDSGGGPPAK.[Q]	33356174	PNN	5411	33356174 [63-75]	1xPhospho [S4(98.7)];2xTMT6plex [N-Term;K13]	S4(Phospho): 98.72	2	1.423	0.901507
[K].TEIKEEEDQPSTSATQSSPAPGQSK.[K]	50345997	EP300	2033	50345997 [1021-1045]	1xPhospho [S/T];3xTMT6plex [N-Term;K25];K4	S11(Phospho): 16.66;T12(Phospho): 16.66;S13(Phospho): 16.66;T15(Phospho): 16.66;S17(Phospho): 16.66;S18(Phospho): 16.66	13	1.111	0.901291
[K].GPPDFSSDEEREPTPVLGSGAAAAGR.[S]	4507555	TMPO	7112	4507555 [61-86]	1xTMT6plex [N-Term];3xPhospho [S6(100);S7(100);T14(100)]	S6(Phospho): 100;S7(Phospho): 100;T14(Phospho): 100	2	0.879	0.901099
[K].NAEEESESEAEEGD.[-]	333470719	BZW1	9689	333470719 [438-451]	1xPhospho [S6(100)];1xTMT6plex [N-Term]	S6(Phospho): 99.97	1	0.845	0.900464
[R].GFSANSAR.[L]	119703753;5031839	KRT6B|KRT6A	3854|3853	119703753 [17-24];5031839 [17-24]	1xTMT6plex [N-Term];2xPhospho [S3(100);S6(100)]	S3(Phospho): 100;S6(Phospho): 100	2	1.352	0.900178
[R].RTSSSSDIPK.[S]	767939877	HIVEP1	3096	767939877 [811-820]	2xPhospho [S4(96.9);2xTMT6plex [N-Term;K10];T/S]	T2(Phospho): 50.29;S3(Phospho): 50.29;S4(Phospho): 96.99	1	2.332	0.899234
[R].RLSSLRASTSK.[S]	17158044	RPS6	6194	17158044 [233-243]	2xPhospho [S3(99.9);2xTMT6plex [N-Term;K11];S4(97.4)]	S3(Phospho): 50;S4(Phospho): 50;S8(Phospho): 98.67	1	1.567	0.898442
[R].HGGSPQPLATTPLSQEPVNPPSEASPTR.[D]	118572613	SRRM2	23524	118572613 [374-401]	1xTMT6plex [N-Term];3xPhospho [S4(100);S/T]	S4(Phospho): 100;T10(Phospho): 33.33;T11(Phospho): 33.33;S14(Phospho): 33.33;S22(Phospho): 49.2;S25(Phospho): 49.2	2	0.946	0.898023
[R].ALPDSQQVPSSPASK.[H]	578808211	RGS12	6002	578808211 [840-854]	1xPhospho [S11(100)];2xTMT6plex [N-Term;K15]	S11(Phospho): 99.99	2	0.991	0.897707
[R].GPSPSSPTPPAAAAPAEQAPR.[A]	55742678	GLCCI1	113263	55742678 [103-123]	1xTMT6plex [N-Term];2xPhospho [S3(95.5);S/T]	S3(Phospho): 95.5;S5(Phospho): 34.83;S6(Phospho): 34.83;T8(Phospho): 34.83	1	0.581	0.897245
[R].SRDATPPVSPINMEDQER.[I]	4504809	JUNB	3726	4504809 [251-268]	1xOxidation [M13];1xTMT6plex [N-Term];3xPhospho [S1(100);S9(100)];T5(100)	S1(Phospho): 100;T5(Phospho): 100;S9(Phospho): 100	2	0.877	0.896783
[K].KEQSEVSVSPR.[A]	530401729	RSRC2	65117	530401729 [24-34]	2xPhospho [S7(98);2xTMT6plex [N-Term;K1];S9(98)]	S7(Phospho): 100;S9(Phospho): 100	2	1.041	0.896777
[K].TVTPASSAKTSPAK.[Q]	308818195	DPYSL2	1808	308818195 [617-630]	2xPhospho [T/S];3xTMT6plex [N-Term;K14];K9	T10(Phospho): 96.28;S11(Phospho): 96.28	2	0.441	0.896428
[R].VVSSVSSSPR.[Q]	110618244	KDM5A	5927	110618244 [1324-1333]	1xTMT6plex [N-Term];2xPhospho [S]	S3(Phospho): 49.62;S4(Phospho): 49.62;S6(Phospho): 33.58;S7(Phospho): 33.58;S8(Phospho): 33.58	2	1.242	0.895512
[R].LQDSSDPDTGSEEEGSSRLSPPHSPR.[DG]	767910855	ARHGEF2	9181	767910855 [1080-1105]	1xTMT6plex [N-Term];2xPhospho [S24(93.3);T/S]	T9(Phospho): 90.41;S24(Phospho): 99.71	11	0.956	0.895402
[R].LIPGSNLDSEPR.[I]	29826289	CASC4	113201	29826289 [324-335]	1xPhospho [S];1xTMT6plex [N-Term]	S5(Phospho): 98.18	5	1.215	0.895231
[R].RSSTSGDTEEEEEEEVVPFSSDEQKR.[SR]	1034675221	ZNF185	7739	1034675221 [151-176]	2xPhospho [S/T];2xTMT6plex [N-Term;K25]	S2(Phospho): 50;S3(Phospho): 50;S5(Phospho): 49.53;T8(Phospho): 49.53	6	0.981	0.895149
[R].GNIETTSEDGQVFSPK.[K]	530402186	ZC3H13	23091	530402186 [1008-1023]	1xPhospho [S14(96.9)];2xTMT6plex [N-Term;K16]	S14(Phospho): 99.99	2	1.294	0.894739
[K].EELEQQTDGDCEEDEEEENDGETPK.[S]	4507525	SEC62	7095	4507525 [369-393]	1xCarbamidomethyl [C11];1xPhospho [T7(100)];2xTMT6plex [N-Term;K25]	T7(Phospho): 100	5	1.063	0.894471
[R].SSSVTSIDKESR.[E]	189217915	STXBP5	134957	189217915 [780-791]	2xPhospho [S/T];2xTMT6plex [N-Term;K9]	T5(Phospho): 98.86;S6(Phospho): 100	1	0.552	0.893828
[R].GAPSSPATGVLPSPQGK.[S]	62955803	NUP188	23511	62955803 [1705-1721]	1xPhospho [S/T];2xTMT6plex [N-Term;K17]	S4(Phospho): 49.07;S5(Phospho): 49.07	2	1.072	0.892471
[K].AAPEASSPPASPLQHLLPGK.[A]	767958073	FAM129B	64855	767958073 [716-735]	2xPhospho [S];2xTMT6plex [N-Term;K20]	S6(Phospho): 50.51;S7(Phospho): 50.51;S11(Phospho): 98.98	7	1.057	0.892016
[R].SQSSHSYDDSTLPLIDR.[N]	146231940	CTNND1	1500	146231940 [859-875]	1xTMT6plex [N-Term];2xPhospho [S/Y/T]	S6(Phospho): 96.18;S10(Phospho): 50;T11(Phospho): 50	5	1.514	0.891889
[R].VAAAAGSGPSPPGSPGHDR.[E]	13559514	PI4K2A	55361	13559514 [38-56]	1xPhospho [S10(100)];1xTMT6plex [N-Term]	S10(Phospho): 99.98	9	1.028	0.891023
[K].DIKEESDEEEEDDEESGR.[L]	530387240	RBM33	155435	530387240 [240-257]	1xPhospho [S6(100)];2xTMT6plex [N-Term;K3]	S6(Phospho): 100	1	0.981	0.890888
[R].RSSSDEQGLSYSSLK.[N]	268840316	IL1RAP	3556	268840316 [554-568]	2xPhospho [S/Y];2xTMT6plex [N-Term;K15]	S2(Phospho): 50;S3(Phospho): 50;S4(Phospho): 99.99	3	1.488	0.890787
[K].EEEEGISQESSEEEQ.[-]	22208967	HMGA1	3159	22208967 [93-107]	1xTMT6plex [N-Term];2xPhospho [S7(100);S]	S10(Phospho): 98.26;S11(Phospho): 98.26	9	0.881	0.890078
[K].NDKSEEEQSSSSVK.[K]	117190192	HNRNPC	3183	117190192 [230-243]	1xPhospho [S4(100)];3xTMT6plex [N-Term;K14];K3	S4(Phospho): 100	1	1.081	0.889925
[R].YTGSPDSLR.[S]	530366268	PLXNA2	5362	530366268 [1642-1650]	1xTMT6plex [N-Term];2xPhospho [S4(97.4);S7(100)]	S4(Phospho): 97.36;S7(Phospho): 100	1	0.746	0.889357
[K].GISSRISGQDSPPR.[V]	157074214	KCNU1	157855	157074214 [643-656]	1xTMT6plex [N-Term];2xPhospho [S]	S7(Phospho): 94.07;S11(Phospho): 94.07	1	0.974	0.888973
[R].NDSVIVADQTPTPTR.[F]	22538422	ATF2	1386	22538422 [60-74]	1xTMT6plex [N-Term];2xPhospho [T10(100);T12(98.2)]	T10(Phospho): 99.98;T12(Phospho): 98.58	5	1.056	0.888923
[R].LKSEDGVEGDLGETQSR.[T]	1079280806	AHNAK	79026	1079280806 [133-149]	1xPhospho [S3(100)];2xTMT6plex [N-Term;K2]	S3(Phospho): 100	2	2.619	0.886592
[R].HGSDPAFAPGPR.[G]	1034659998	FAM83H	286077	1034659998 [722-733]	1xPhospho [S3(100)];1xTMT6plex [N-Term]	S3(Phospho): 100	2	1.186	0.885437
[R].LSTTPSPTSSLHEDGVEDFRR.[Q]	7662274	TOX4	9878	7662274 [173-193]	1xTMT6plex [N-Term];2xPhospho [S/T]	S2(Phospho): 42.13;T3(Phospho): 14.72;T4(Phospho): 28.44;S6(Phospho): 29.43;T8(Phospho): 28.43;S9(Phospho): 28.43;S10(Phospho): 28.43	1	0.732	0.885161
[R].EPVVTPQDLEGPGRAESKESVGSR.[D]	1034608385	MAP1S	55201	1034608385 [456-479]	2xPhospho [T/S];2xTMT6plex [N-Term;K18]	T5(Phospho): 50;S17(Phospho): 50;S20(Phospho): 50;S23(Phospho): 50	1	0.66	0.883000
[R].GGPGAAPGGASPASSSSAASSPSSGR.[A]	13994287	KLF16	83855	13994287 [89-114]	1xPhospho [S];1xTMT6plex [N-Term]	S11(Phospho): 14.04;S14(Phospho): 14.04;S15(Phospho): 14.04;S16(Phospho): 14.04;S17(Phospho): 14.04;S20(Phospho): 14.04;S21(Phospho): 14.04	6	1.077	0.882942
[K].SNTAGSQSQVETEA.[-]	4503729	FKBP4	2288	4503729 [446-459]	1xPhospho [S6(96.1)];1xTMT6plex [N-Term]	S6(Phospho): 100	1	1.342	0.882759
[R].LEPVYSPPGSPPPGDPR.[I]	4506735	RPS6KA4	8986	4506735 [338-354]	1xTMT6plex [N-Term];2xPhospho [Y/S]	Y5(Phospho): 66.67;S6(Phospho): 66.67;S10(Phospho): 66.67	2	1.745	0.881846
[K].EKQSSEEEEKETR.[G]	117553608	SLC39A7	7922	117553608 [272-284]	2xPhospho [S4(100);3xTMT6plex [N-Term;K10];K2;S5(100)]	S4(Phospho): 100;S5(Phospho): 100	1	2.104	0.879917
[K].SEAAAPHTDAGGGLSSDEEEGTSSQAEAAR.[I]	224809295	XPC	7508	224809295 [869-898]	1xTMT6plex [N-Term];2xPhospho [S/T]	S15(Phospho): 100;S16(Phospho): 100	12	1.134	0.879792
[R].ALAAGADSPKTEEARPSPAPGPGTPTGTPTR.[T]	167614488	TBC1D10B	26000	167614488 [125-155]	2xPhospho [T24(84.1);2xTMT6plex [N-Term;K10];S/T]	S8(Phospho): 34.49;T11(Phospho): 34.49;S17(Phospho): 34.49;T24(Phospho): 84.06	2	1.383	0.879704
[R].SGSPSDNSGAEEMEVSLAKPK.[H]	62241015	AKT1	207	62241015 [122-142]	1xOxidation [M13];2xPhospho [S];3xTMT6plex [N-Term;K19;K21]	S3(Phospho): 49.99;S5(Phospho): 49.99;S8(Phospho): 99.96	2	0.494	0.879587
[R].DSHSSEEDEASSQTDLSQTISKK.[T]	767908953	TOR1AIP1	26092	767908953 [153-175]	2xPhospho [S/T];3xTMT6plex [N-Term;K22;K23]	S4(Phospho): 100;S5(Phospho): 100	3	0.992	0.878369
[K].ISQRDPSPESNK.[K]	530375304	PHC3	80012	530375304 [269-280]	1xPhospho [S7(100)];2xTMT6plex [N-Term;K12]	S7(Phospho): 100	1	1.139	0.878014
[R].ENYSDSEEEDDDDVASSR.[Q]	7706607	LEMD3	23592	7706607 [256-273]	1xTMT6plex [N-Term];2xPhospho [S4(98.4);S6(98.4)]	Y3(Phospho): 50.01;S4(Phospho): 50.01;S6(Phospho): 99.97	3	0.899	0.877307
[K].YAEISSDEDNDSDEAFESSR.[K]	47578105;530378760	NIPBL|NIPBL	25836|25836	47578105 [1085-1104];530378760 [1085-1104]	1xTMT6plex [N-Term];3xPhospho [S/Y]	S5(Phospho): 100;S6(Phospho): 100;S12(Phospho): 100	3	1.857	0.876541
[K].ADREVQAEQPSSSSPR.[R]	118600973	RBMX2	51634	118600973 [175-190]	1xPhospho [S];1xTMT6plex [N-Term]	S11(Phospho): 49.43;S12(Phospho): 49.43	6	1.485	0.876406
[R].RNSSSPVSPASVPGQR.[R]	33457336	IRF2BPL	64207	33457336 [655-670]	1xTMT6plex [N-Term];2xPhospho [S3(100);S5(100)]	S3(Phospho): 99.98;S4(Phospho): 98.55	6	1.116	0.875815
[K].GQKSPGALETPSAAGSQGNTASQGK.[E]	6005709	PALM2-AKAP2	445815	6005709 [621-645]	1xPhospho [S4(100)];3xTMT6plex [N-Term;K25];K3	S4(Phospho): 100	3	1.321	0.875520
[K].KVLSDSEDEEKDADVPGTSTR.[K]	1081684143	PHIP	55023	1081684143 [1278-1298]	2xPhospho [S4(100);3xTMT6plex [N-Term;K1;K11];S6(100)]	S4(Phospho): 100;S6(Phospho): 100	1	0.88	0.874864
[R].KAQAVSEEEEEEEGKSSSPK.[K]	9966805	DDX24	57062	9966805 [77-96]	2xPhospho [S6(100);4xTMT6plex [N-Term;K1;K15;K20];S]	S6(Phospho): 100;S17(Phospho): 49.38;S18(Phospho): 49.38	2	0.56	0.874554
[K].AEDGATPSPSNETPKK.[K]	153070260	MARCKS	4082	153070260 [138-153]	1xPhospho [S/T];3xTMT6plex [N-Term;K15;K16]	S8(Phospho): 33.12;S10(Phospho): 33.12;T13(Phospho): 33.12	3	0.914	0.874136
[R].GAVAAEGASDTEREEPTESQGLAAR.[L]	557440888	PHRF1	57661	557440888 [907-931]	1xPhospho [S9(100)];1xTMT6plex [N-Term]	S9(Phospho): 99.97	7	1.663	0.872544
[R].HGGSPQPLATTPLSQEPVNPPSEASPTR.[D]	118572613	SRRM2	23524	118572613 [374-401]	1xTMT6plex [N-Term];2xPhospho [S4(100);S25(96)]	S4(Phospho): 100;S25(Phospho): 97.79	9	0.924	0.871886
[R].SFSTASAITPSVSR.[T]	119395754	KRT5	3852	119395754 [16-29]	1xTMT6plex [N-Term];2xPhospho [T/S]	S1(Phospho): 33.76;S3(Phospho): 33.76;T4(Phospho): 33.14;S6(Phospho): 99.33	1	0.78	0.871874
[R].VPGPAEGPAEPAAEASDEAER.[R]	1034574337	TMEM132A	54972	1034574337 [528-548]	1xPhospho [S16(100)];1xTMT6plex [N-Term]	S16(Phospho): 100	2	0.855	0.869431
[R].ALVEFESNPEETREPGSPPSVQR.[A]	13129104	CCDC86	79080	13129104 [31-53]	1xPhospho [T/S];1xTMT6plex [N-Term]	S17(Phospho): 96.85	10	0.882	0.868272
[R].SSSLIQLTSQNSSPNQQR.[T]	767946724	CPSF4	10898	767946724 [205-222]	1xPhospho [S/T];1xTMT6plex [N-Term]	S13(Phospho): 95.76	15	1.073	0.868253
[R].SRLTPVSPESSSTEEK.[S]	4505571	SQSTM1	8878	4505571 [266-281]	2xPhospho [S7(97.2);2xTMT6plex [N-Term;K16];S/T]	T4(Phospho): 100;S7(Phospho): 100	2	0.799	0.867749
[K].AEDSDSEPEPEDNVR.[L]	964750564	XRN2	22803	964750564 [574-588]	1xTMT6plex [N-Term];2xPhospho [S4(100);S6(100)]	S4(Phospho): 100;S6(Phospho): 100	5	0.883	0.867724
[R].SGSSPEVK.[D]	118572613	SRRM2	23524	118572613 [1598-1605]	2xPhospho [S3(100);2xTMT6plex [N-Term;K8];S4(100)]	S3(Phospho): 99.99;S4(Phospho): 100	2	1.793	0.867722
[R].DTSPSSGSAVSSSK.[V]	1034579978	PPHLN1	51535	1034579978 [210-223]	1xPhospho [T/S];2xTMT6plex [N-Term;K14]	T2(Phospho): 50;S3(Phospho): 50	5	0.966	0.867499
[R].RVSHSPPPK.[Q]	1034554585	SRRM1	10250	1034554585 [673-681]	2xPhospho [S3(100);2xTMT6plex [N-Term;K9];S5(100)]	S3(Phospho): 100;S5(Phospho): 100	2	1.174	0.867454
[K].AALLKASPK.[K]	78000181	RPL14	9045	78000181 [133-141]	1xPhospho [S7(100)];3xTMT6plex [N-Term;K5;K9]	S7(Phospho): 100	1	0.346	0.866806
[R].KTSAVSSPLLDQQR.[N]	530402186	ZC3H13	23091	530402186 [236-249]	1xPhospho [S/T];2xTMT6plex [N-Term;K1]	S6(Phospho): 50;S7(Phospho): 50	3	1.245	0.866723
[K].GLSPAMSPALQR.[L]	530426297	ESS2	8220	530426297 [390-401]	1xOxidation [M6];1xTMT6plex [N-Term];2xPhospho [S3(100);S7(100)]	S3(Phospho): 100;S7(Phospho): 100	1	1.074	0.866127
[K].LIPITGGNARSPEDQLGK.[H]	530372165	FAM208A	23272	530372165 [917-934]	1xPhospho [S/T];2xTMT6plex [N-Term;K18]	S11(Phospho): 98.35	6	1.272	0.865721
[K].GSLAPQDSDSEVSQNR.[S]	767957960	PTPN3	5774	767957960 [439-454]	1xPhospho [S8(100)];1xTMT6plex [N-Term]	S8(Phospho): 98.85	3	0.992	0.865551
[K].TSAAACAVTDLSDDSDFDEK.[A]	9966799	UTP3	57050	9966799 [354-373]	1xCarbamidomethyl [C6];2xPhospho [T/S];2xTMT6plex [N-Term;K20]	T9(Phospho): 66.67;S12(Phospho): 66.67;S15(Phospho): 66.67	2	1.244	0.864074
[R].LATNTSAPDLK.[N]	1370484102	MAP4	4134	1370484102 [2097-2107]	1xPhospho [T/S];2xTMT6plex [N-Term;K11]	T5(Phospho): 50;S6(Phospho): 50	2	0.439	0.863992
[R].RAGGGGGLGAGSPALSGGQGR.[R]	106507164	RAB12	201475	106507164 [10-30]	1xPhospho [S12(100)];1xTMT6plex [N-Term]	S12(Phospho): 100	2	1.003	0.863398
[R].HDTPDPSPLR.[G]	14249338	BUD13	84811	14249338 [157-166]	1xTMT6plex [N-Term];2xPhospho [T3(100);S7(100)]	T3(Phospho): 100;S7(Phospho): 100	2	1.944	0.862431
[K].SPGSTPTTPTSSQAPQK.[L]	33946327	NUP214	8021	33946327 [430-446]	2xPhospho [S4(98.2);2xTMT6plex [N-Term;K17];T8(93.3)]	S4(Phospho): 98.63;T7(Phospho): 49.67;T8(Phospho): 49.67	22	1.051	0.862346
[R].SDELLGSDDSHDGESESNAK.[V]	237681119	BRCA1	672	237681119 [389-408]	2xPhospho [S7(100);2xTMT6plex [N-Term;K20];S10(97.7)]	S7(Phospho): 100;S10(Phospho): 97.68	1	0.764	0.862078
[K].AGEAPTENPAPPTQQSSAE.[-]	224586882	YBX3	8531	224586882 [354-372]	1xTMT6plex [N-Term];2xPhospho [T13(98);S16(98)]	T13(Phospho): 97.97;S16(Phospho): 97.97	2	1.014	0.861881
[R].HNQGDSDDDVEETHPMDGNDSDYDPKK.[E]	1034560064	RCOR3	55758	1034560064 [242-268]	1xOxidation [M16];2xPhospho [S/T/Y];3xTMT6plex [N-Term;K26;K27]	S6(Phospho): 99.98;T13(Phospho): 33.34;S21(Phospho): 33.34;Y23(Phospho): 33.34	1	0.629	0.861559
[R].AGGPATPLSPTR.[L]	388240801	LMNB2	84823	388240801 [29-40]	1xTMT6plex [N-Term];2xPhospho [T6(100);S/T]	T6(Phospho): 100;S9(Phospho): 99.98	2	1.18	0.861461
[R].NRSAEEGELAESK.[S]	578821824	TNKS1BP1	85456	578821824 [1664-1676]	1xPhospho [S3(100)];2xTMT6plex [N-Term;K13]	S3(Phospho): 100	2	0.977	0.861008
[R].ANNLHSGDNFQLNDSEIER.[Q]	1034577824	OSBPL8	114882	1034577824 [323-341]	1xPhospho [S15(98.1)];1xTMT6plex [N-Term]	S15(Phospho): 100	2	0.939	0.859394
[R].NESEDNKFSDDSDDDFVQPR.[K]	47419911	TRIM24	8805	47419911 [1017-1036]	2xPhospho [S];2xTMT6plex [N-Term;K7]	S9(Phospho): 99.92;S12(Phospho): 100	1	1.66	0.858778
[R].SQSESSDEVTELDLSHGKK.[D]	1034582490	C2CD5	9847	1034582490 [767-785]	2xPhospho [S/T];3xTMT6plex [N-Term;K18;K19]	S1(Phospho): 49.34;S3(Phospho): 49.34;S6(Phospho): 94.67	2	1.197	0.857352
[R].AAVGQESPGGLEAGNAK.[A]	767930270	PDS5A	23244	767930270 [1299-1315]	1xPhospho [S7(100)];2xTMT6plex [N-Term;K17]	S7(Phospho): 100	5	1.118	0.857072
[K].MPLLELGGETTPPLSTERSPEAVGSECPSR.[V]	530383409	CRYBG1	202	530383409 [923-952]	1xCarbamidomethyl [C27];1xOxidation [M1];1xTMT6plex [N-Term];2xPhospho [S15(93.4);T/S]	T10(Phospho): 50;T11(Phospho): 50;S15(Phospho): 93.43	2	1.16	0.856798
[K].GSFSDTGLGDGK.[M]	167234419	THRAP3	9967	167234419 [376-387]	1xPhospho [S/T];2xTMT6plex [N-Term;K12]	S4(Phospho): 98.53	1	1.341	0.855590
[R].EGNTTEDDFPSSPGNGNK.[S]	1034652351	WTAP	9589	1034652351 [312-329]	1xPhospho [S/T];2xTMT6plex [N-Term;K18]	S11(Phospho): 49.99;S12(Phospho): 49.99	2	0.823	0.855402
[R].LLEDSEESSEETVSR.[A]	255982614	DHX16	8449	255982614 [99-113]	1xTMT6plex [N-Term];2xPhospho [S5(100);S/T]	S5(Phospho): 100;S8(Phospho): 100	23	1.036	0.855109
[R].RPDPDSDEDEDYERER.[R]	224549005	RBM17	84991	224549005 [150-165]	1xPhospho [S6(100)];1xTMT6plex [N-Term]	S6(Phospho): 100	2	0.817	0.854994
[K].EAEEGPTGASESQDSPR.[K]	55749544	SH3PXD2A	9644	55749544 [505-521]	1xPhospho [S15(98.7)];1xTMT6plex [N-Term]	S15(Phospho): 99.15	7	0.958	0.853139
[R].SGSESSVDQK.[T]	118572613	SRRM2	23524	118572613 [1517-1526]	1xPhospho [S3(97.7)];2xTMT6plex [N-Term;K10]	S3(Phospho): 49.74;S6(Phospho): 49.74	1	2.316	0.852978
[R].DVTNFTVGGFAPMSPR.[I]	161169023	SUPT5H	6829	161169023 [653-668]	1xOxidation [M13];1xPhospho [T/S];1xTMT6plex [N-Term]	T6(Phospho): 49.99;S14(Phospho): 49.99	2	0.969	0.852877
[K].EIGSSTSSSSETVVK.[L]	388490221	FRMD6	122786	388490221 [503-517]	2xPhospho [S/T];2xTMT6plex [N-Term;K15]	T6(Phospho): 19.94;S7(Phospho): 19.94;S8(Phospho): 19.94;S9(Phospho): 19.94;S10(Phospho): 19.94;T12(Phospho): 99.93	10	0.829	0.852804
[K].EYPSPPPSPLR.[R]	767994242	TANC2	26115	767994242 [1612-1622]	1xTMT6plex [N-Term];2xPhospho [S4(98.4);S8(100)]	S4(Phospho): 98.38;S8(Phospho): 100	4	1.051	0.851906
[R].AADTEEMDIEMDSGDEA.[-]	4505117	MBD2	8932	4505117 [395-411]	1xPhospho [S13(100)];1xTMT6plex [N-Term];2xOxidation [M7;M11]	S13(Phospho): 100	4	1.122	0.851096
[R].RNSFSENEK.[H]	530370697	CWC22	57703	530370697 [827-835]	1xPhospho [S3(100)];2xTMT6plex [N-Term;K9]	S3(Phospho): 100	1	1.236	0.850945
[R].GRSECDSSPEPK.[A]	118572613	SRRM2	23524	118572613 [1476-1487]	1xCarbamidomethyl [C5];2xTMT6plex [N-Term;3xPhospho [S3(100);K12];S7(100);S8(100)]	S3(Phospho): 100;S7(Phospho): 100;S8(Phospho): 100	1	0.56	0.850900
[R].DTQSNELIEINPQSEGK.[V]	100913220	COL16A1	1307	100913220 [257-273]	1xPhospho [S4(98.7)];2xTMT6plex [N-Term;K17]	S4(Phospho): 98.69	3	1.38	0.850129
[K].LFGAPEPSTIARSLPTTVPESPNYR.[N]	530380464	LARP1	23367	530380464 [754-778]	1xTMT6plex [N-Term];2xPhospho [T/S/Y]	S13(Phospho): 99.84;T16(Phospho): 48.93;S21(Phospho): 48.96	10	0.834	0.849254
[R].KPFSVSSTPTMSR.[S]	1394533392	TMF1	7110	1394533392 [925-937]	1xOxidation [M11];2xPhospho [S/T];2xTMT6plex [N-Term;K1]	S7(Phospho): 94.22;T8(Phospho): 94.22	1	0.956	0.848852
[R].LVREDENDASDDEDDDEKR.[R]	22035565	PAXBP1	94104	22035565 [253-271]	1xPhospho [S10(100)];2xTMT6plex [N-Term;K18]	S10(Phospho): 100	2	1.361	0.848512
[R].GGSSGATGEPGDSSDREPGRPPGDR.[A]	807045813	FBRS	64319	807045813 [177-201]	1xTMT6plex [N-Term];2xPhospho [S13(100);S14(100)]	S13(Phospho): 99.98;S14(Phospho): 99.98	1	0.953	0.847359
[R].VTNDISPESSPGVGRR.[R]	767950881	PCM1	5108	767950881 [60-75]	1xTMT6plex [N-Term];2xPhospho [S9(98.4);S10(98.4)]	S9(Phospho): 98.54;S10(Phospho): 98.54	3	0.954	0.845730
[R].VTAEDKGTGNK.[N]	16507237	HSPA5	3309	16507237 [511-521]	1xPhospho [T8(100)];3xTMT6plex [N-Term;K11];K6	T8(Phospho): 100	1	4.826	0.845290
[K].TPSPKEEDEEPESPPEKK.[T]	56118310	NUCKS1	64710	56118310 [202-219]	2xPhospho [S3(98);4xTMT6plex [N-Term;K17;K18];K5;S13(100)]	S3(Phospho): 97.95;S13(Phospho): 100	1	0.562	0.845127
[R].HSSISPVRLPLNSSLGAELSR.[K]	157817023	CDK12	51755	157817023 [381-401]	1xTMT6plex [N-Term];2xPhospho [S]	S3(Phospho): 96.65;S5(Phospho): 96.65	1	0.713	0.844299
[R].AKNSPPQAPSTR.[D]	806638628	STK11IP	114790	806638628 [758-769]	1xPhospho [S4(100)];2xTMT6plex [N-Term;K2]	S4(Phospho): 100	3	1.153	0.843276
[K].FTGYQAIQQQSSSETEGEGGNTADASSEEEGDRVEEDGK.[G]	578827046	HDGFL3	50810	578827046 [96-134]	2xPhospho [S/T/Y];2xTMT6plex [N-Term;K39]	S11(Phospho): 28.57;S12(Phospho): 28.57;S13(Phospho): 28.57;T15(Phospho): 28.57;T22(Phospho): 28.57;S26(Phospho): 28.57;S27(Phospho): 28.57	5	0.978	0.843102
[R].ALEEGDGSVSGSSPR.[S]	971059218	FNBP4	23360	971059218 [422-436]	1xPhospho [S];1xTMT6plex [N-Term]	S8(Phospho): 25;S10(Phospho): 25;S12(Phospho): 25;S13(Phospho): 25	8	1.029	0.841968
[K].TNQGSMENLCPGK.[A]	767985868	PPIP5K1	9677	767985868 [936-948]	1xCarbamidomethyl [C10];1xOxidation [M6];1xPhospho [S5(100)];2xTMT6plex [N-Term;K13]	S5(Phospho): 99.98	1	1.052	0.841285
[R].AGDTSDQEGHSLEEK.[A]	530422376	ELF4	2000	530422376 [147-161]	1xPhospho [T/S];2xTMT6plex [N-Term;K15]	T4(Phospho): 49.56;S5(Phospho): 49.56	4	1.041	0.841063
[K].VLVQNAAGSQEK.[L]	223029410	TLN1	7094	223029410 [2032-2043]	1xPhospho [S9(100)];2xTMT6plex [N-Term;K12]	S9(Phospho): 100	2	0.818	0.840422
[K].AGMSSNQSISSPVLDAVPRTPSR.[E]	118572613	SRRM2	23524	118572613 [1394-1416]	1xOxidation [M3];1xTMT6plex [N-Term];2xPhospho [T20(97);S]	S8(Phospho): 33.33;S10(Phospho): 33.33;S11(Phospho): 33.33;T20(Phospho): 98.3	3	0.991	0.840303
[K].RGSIGENQGEEK.[G]	1370511010	CALD1	800	1370511010 [200-211]	1xPhospho [S3(100)];2xTMT6plex [N-Term;K12]	S3(Phospho): 100	1	0.685	0.839090
[R].TVSPGSVSPIHGQGQVVENLK.[A]	1370477823	ITSN2	50618	1370477823 [882-902]	2xPhospho [S3(97.6);2xTMT6plex [N-Term;K21];S6(97.6)]	S3(Phospho): 97.58;S6(Phospho): 97.58	1	0.824	0.838991
[R].NNSFTAPSTVGK.[R]	4505611	PARN	5073	4505611 [555-566]	1xPhospho [S3(98.7)];2xTMT6plex [N-Term;K12]	S3(Phospho): 98.45	3	0.517	0.838964
[K].GGEFDEFVNDDTDDDLPISK.[K]	7661950	CTR9	9646	7661950 [914-933]	1xPhospho [T12(100)];2xTMT6plex [N-Term;K20]	T12(Phospho): 100	1	1.372	0.838176
[K].SEVQQPVHPKPLSPDSR.[A]	767974553	LIMA1	51474	767974553 [351-367]	1xPhospho [S13(100)];2xTMT6plex [N-Term;K10]	S13(Phospho): 99.98	2	0.852	0.838009
[K].ALEQQEAESDSSDTEEKDDDDDEEDVGKR.[E]	31543091	MAK16	84549	31543091 [189-218]	2xPhospho [S/T];3xTMT6plex [N-Term;K17;K29]	S9(Phospho): 50;S11(Phospho): 50;S12(Phospho): 50;T14(Phospho): 50	6	0.64	0.837668
[K].AAQQAASSSGQGQQAQTPTGF.[-]	414144878	CSNK1A1	1452	414144878 [305-325]	1xPhospho [S/T];1xTMT6plex [N-Term]	T17(Phospho): 96.51	35	1.099	0.837488
[R].AVSLDSPVSVGSSPPVK.[N]	32307126	NCOA3	8202	32307126 [855-871]	2xPhospho [S3(98.8);2xTMT6plex [N-Term;K17];S13(100)]	S3(Phospho): 98.8;S13(Phospho): 99.95	3	1.006	0.836960
[R].KASPEPEGEAAGK.[M]	24308115	IRF2BP1	26145	24308115 [382-394]	1xPhospho [S3(100)];3xTMT6plex [N-Term;K1;K13]	S3(Phospho): 100	2	1.157	0.836665
[R].MAPTPIPTRSPSDSSTASTPVAEQIER.[A]	1034643898	DBN1	1627	1034643898 [376-402]	1xOxidation [M1];1xPhospho [T/S];1xTMT6plex [N-Term]	T4(Phospho): 31.78;T8(Phospho): 31.78;S10(Phospho): 31.78	9	1.282	0.835204
[R].YLEEDNSDESDAEGEHGDGAEEEAPPAGPRPGPEPAGLGR.[R]	45580709	UNC93B1	81622	45580709 [541-580]	1xTMT6plex [N-Term];2xPhospho [S7(99.8);S10(99.8)]	S7(Phospho): 100;S10(Phospho): 100	5	1.097	0.834824
[R].TSSFAEPGGGGGGGGGPGGSASGPGGTGGGK.[A]	49574532	GSK3A	2931	49574532 [19-50]	1xPhospho [S/T];2xTMT6plex [N-Term;K32]	S3(Phospho): 98.56	4	0.727	0.832980
[K].EKLQEEGGGSDEEETGSPSEDGMQSAR.[T]	5902034	PWP1	11137	5902034 [41-67]	1xOxidation [M23];2xPhospho [T/S];2xTMT6plex [N-Term;K2]	S10(Phospho): 100;T15(Phospho): 49.39;S17(Phospho): 49.39	2	0.708	0.832832
[K].GVPAGNSDTEGGQPGR.[K]	7662238	ACIN1	22985	7662238 [832-847]	1xPhospho [S7(100)];1xTMT6plex [N-Term]	S7(Phospho): 100	2	2.074	0.832267
[K].AQTPPGPSLSGSKSPCPQEK.[S]	118572613	SRRM2	23524	118572613 [1001-1020]	1xCarbamidomethyl [C16];1xPhospho [S/T];3xTMT6plex [N-Term;K13;K20]	S8(Phospho): 33;S10(Phospho): 33;S12(Phospho): 33	3	1.629	0.831708
[R].ESRQESDPEDDDVK.[K]	33356174	PNN	5411	33356174 [95-108]	1xPhospho [S];2xTMT6plex [N-Term;K14]	S6(Phospho): 100	3	0.937	0.831492
[K].VHAYFAPVTPPPSVGGSR.[Q]	767996256	FBXL20	84961	767996256 [446-463]	1xTMT6plex [N-Term];2xPhospho [T9(100);S/Y]	T9(Phospho): 100;S17(Phospho): 98.4	3	1.066	0.830006
[R].RFSDSEGEETVPEPR.[L]	4502889	CLN3	1201	4502889 [10-24]	1xTMT6plex [N-Term];2xPhospho [S3(100);S5(100)]	S3(Phospho): 100;S5(Phospho): 100	3	0.937	0.829901
[R].RSPSPYYSR.[GY]	9558733;544346329	#N/A	#N/A	9558733 [259-267];544346329 [157-165]	1xTMT6plex [N-Term];2xPhospho [S2(100);S4(99.9)]	S2(Phospho): 100;S4(Phospho): 49.6;S8(Phospho): 49.6	1	0.977	0.827694
[K].EHYPVSSPSSPSPPAQPGGVSR.[N]	38683807	ANKRD17	26057	38683807 [2036-2057]	1xTMT6plex [N-Term];2xPhospho [S/Y]	S6(Phospho): 39.76;S7(Phospho): 39.76;S9(Phospho): 39.76;S10(Phospho): 39.76;S12(Phospho): 39.76	9	0.949	0.827554
[K].IQNNDNVGKPKSRSPSR.[H]	21361282	SRSF4	6429	21361282 [277-293]	2xPhospho [S12(98);3xTMT6plex [N-Term;K11];K9;S14(98)]	S12(Phospho): 97.18;S14(Phospho): 97.18	1	0.98	0.827200

Patil et al,2020. Proteomic and phosphoproteomic profiling of shammah-induced signaling in oral keratinocytes

Supplementary Table S2: List of phosphopeptides identified and quantified in OKF6/TERT1-Shammah and OKF6/TERT1-Parental phosphoproteome using TMT/TiO_2-based quantitative phosphoproteomic approach

Peptide Sequence	Protein Accessions	Gene Symbol	Gene ID	Positions in Proteins	Modifications	PhosphoRS: Best Site Probabilities	# PSMs	Fold change	p-value
[R].SRPTSEGSDIESTEPQK.[Q]	388490152	LARP7	51574	388490152 [261-277]	2xPhospho [T/S];2xTMT6plex [N-Term;K17]	T4(Phospho): 49.59;S5(Phospho): 49.59;S8(Phospho): 99.98	4	1.386	0.826957
[K].ETSSPGTDDVFTPAPSDSPSSQR.[I]	27777632	SLC9A1	6548	27777632 [768-790]	1xPhospho [S/T];1xTMT6plex [N-Term]	S18(Phospho): 49.33;S21(Phospho): 49.33	5	0.999	0.826617
[R].LPSTSGSEGVPFR.[T]	32967311	EPHA2	1969	32967311 [895-907]	1xTMT6plex [N-Term];2xPhospho [S3(98.6);S7(100)]	S3(Phospho): 98.58;S7(Phospho): 99.98	11	2.002	0.826321
[K].AGEQQLSEPEDMEMEAGDTDDPPR.[I]	1034595923	USP7	7874	1034595923 [12-35]	1xPhospho [S7(100)];1xTMT6plex [N-Term];2xOxidation [M12;M14]	S7(Phospho): 100	1	0.799	0.825923
[R].KVTSPSSSSSSSSDSESDDEADVSEVTPR.[V]	768020849	NDUFV3	4731	768020849 [147-176]	1xPhospho [S/T];2xTMT6plex [N-Term;K1]	S8(Phospho): 23.58;S9(Phospho): 23.58;S10(Phospho): 23.58;S11(Phospho): 23.58	4	1.09	0.825842
[K].EVELVSE.[-]	4503337	DKC1	1736	4503337 [508-514]	1xPhospho [S6(100)];1xTMT6plex [N-Term]	S6(Phospho): 100	3	1.12	0.824209
[R].TCPSLSPTSPLNNK.[G]	767985566	PIAS1	8554	767985566 [489-502]	1xCarbamidomethyl [C2];1xPhospho [S9(98.7)];2xTMT6plex [N-Term;K14]	S9(Phospho): 98.9	4	0.968	0.823498
[K].YYSDSDDELTVEQR.[R]	767929747	BOD1L1	259282	767929747 [480-493]	1xTMT6plex [N-Term];2xPhospho [S3(98.7);S5(100)]	S3(Phospho): 98.95;S5(Phospho): 98.95	5	1.191	0.822838
[K].AEQGSEEEGEGEEEEEEGGESK.[A]	69354671	ABCF1	23	69354671 [224-245]	1xPhospho [S5(100)];2xTMT6plex [N-Term;K22]	S5(Phospho): 100	2	0.439	0.821364
[K].RQSPLPPQK.[K]	1034670962	SET	6418	1034670962 [5-13]	1xPhospho [S3(100)];2xTMT6plex [N-Term;K9]	S3(Phospho): 100	5	0.908	0.821196
[K].LTVSDGESGEEKK.[T]	530788276	ATRX	546	530788276 [1345-1357]	2xPhospho [S4(100);3xTMT6plex [N-Term;K12;K13];S8(100)]	S4(Phospho): 99.96;S8(Phospho): 100	1	2.531	0.820290
[R].LMRSSTDSLPGELR.[G]	1130413426	NCOA5	57727	1130413426 [374-387]	1xOxidation [M2];1xTMT6plex [N-Term];2xPhospho [S8(100);S/T]	S5(Phospho): 97.59;S8(Phospho): 99.94	2	1.67	0.820258
[K].SPSPPDGSPAATPEIR.[V]	530369146	BIN1	274	530369146 [311-326]	1xTMT6plex [N-Term];2xPhospho [S3(96.7);S/T]	S3(Phospho): 96.67;S8(Phospho): 50.03;T12(Phospho): 50.03	1	0.481	0.819667
[K].IEDVGSDEEDDSGKDKK.[K]	1370507998	HSP90AB1	3326	1370507998 [250-266]	1xPhospho [S6(98.1)];4xTMT6plex [N-Term;K14;K16;K17]	S6(Phospho): 97.59	1	0.359	0.819483
[R].GTQSTEGSPVSK.[M]	1006599006	FAM83D	81610	1006599006 [455-466]	2xPhospho [S4(96.6);2xTMT6plex [N-Term;K12];S8(100)]	S4(Phospho): 50;T5(Phospho): 50;S8(Phospho): 99.97	3	0.511	0.819337
[K].LEGNSPQGSNQGVK.[I]	16933567	RAB8A	4218	16933567 [177-190]	1xPhospho [S9(100)];2xTMT6plex [N-Term;K14]	S9(Phospho): 100	2	1.521	0.819006
[R].EISSSPTSK.[N]	118572613	SRRM2	23524	118572613 [452-460]	1xPhospho [S/T];2xTMT6plex [N-Term;K9]	S5(Phospho): 99.19	3	1.602	0.818970
[R].GEGDAPFSEPGTTSTQRPSSPETATK.[Q]	118572613	SRRM2	23524	118572613 [304-329]	2xPhospho [T/S];2xTMT6plex [N-Term;K26]	T12(Phospho): 25.37;T13(Phospho): 25.37;S14(Phospho): 25.37;T15(Phospho): 25.37;S19(Phospho): 46.22;S20(Phospho): 46.22	3	0.857	0.818879
[K].GTMDDISQEEGSSQGEDSVSGSQR.[I]	30581135	SMC1A	8243	30581135 [945-968]	1xOxidation [M3];1xPhospho [S7(97.2)];1xTMT6plex [N-Term]	S7(Phospho): 33.33;S12(Phospho): 33.33;S13(Phospho): 33.33	6	1.61	0.818403
[R].AQQELVPPQQQASPPQLPK.[A]	530426853	SEC16A	9919	530426853 [1057-1075]	1xPhospho [S13(100)];2xTMT6plex [N-Term;K19]	S13(Phospho): 100	9	0.822	0.818369
[R].ESRQESDPEDDDVKKPALQSSVVATSK.[E]	33356174	PNN	5411	33356174 [95-121]	1xPhospho [S/T];4xTMT6plex [N-Term;K14;K15;K27]	S2(Phospho): 50;S6(Phospho): 50	4	1.454	0.818167
[K].RPASPSSPEHLPATPAESPAQR.[F]	767975125	SUDS3	64426	767975125 [310-331]	1xTMT6plex [N-Term];2xPhospho [S4(100);S/T]	S4(Phospho): 99.98;S6(Phospho): 50.01;S7(Phospho): 50.01	10	1.056	0.817891
[R].EVLYDSEGLSGEER.[G]	768010386	SCAF1	58506	768010386 [732-745]	1xTMT6plex [N-Term];2xPhospho [S6(100);S10(100)]	S6(Phospho): 100;S10(Phospho): 100	5	0.951	0.817367
[K].VPVPGPGSGGNGPER.[E]	1034659998	FAM83H	286077	1034659998 [841-855]	1xPhospho [S8(100)];1xTMT6plex [N-Term]	S8(Phospho): 100	2	0.879	0.816636
[K].SSSNDSVDEETAESDTSPVLEK.[E]	767975924	SCAF11	9169	767975924 [416-437]	1xPhospho [S/T];2xTMT6plex [N-Term;K22]	S1(Phospho): 25;S2(Phospho): 25;S3(Phospho): 25;S6(Phospho): 25	12	1.038	0.815853
[K].TAVPPLSEGDGYSSENTSR.[A]	767923054	SETD2	29072	767923054 [1840-1858]	1xTMT6plex [N-Term];2xPhospho [Y/S/T]	T17(Phospho): 87.33;S18(Phospho): 87.33	2	0.775	0.813934
[K].MLAESDESGDEESVSQTDK.[T]	4505531	OSBP	5007	4505531 [186-204]	1xOxidation [M1];2xPhospho [S/T];2xTMT6plex [N-Term;K19]	S5(Phospho): 50.65;S8(Phospho): 50.65;S13(Phospho): 95.87	2	0.635	0.813732
[R].STPLASPSPSPGRSPQR.[L]	578802190	AHCTF1	25909	578802190 [1244-1260]	1xTMT6plex [N-Term];2xPhospho [S6(97.3);S/T]	S8(Phospho): 49.97;S10(Phospho): 50;S14(Phospho): 98.76	4	1.642	0.813697
[R].DATPPVSPINMEDQER.[I]	4504809	JUNB	3726	4504809 [253-268]	1xOxidation [M11];1xTMT6plex [N-Term];2xPhospho [T3(100);S7(100)]	T3(Phospho): 100;S7(Phospho): 100	3	1.041	0.813659
[K].AQLEPVASPAK.[K]	530389151	PLEC	5339	530389151 [1296-1306]	1xPhospho [S8(100)];2xTMT6plex [N-Term;K11]	S8(Phospho): 100	5	1.048	0.812943
[R].AQTLPTSVVTITSESSPGKR.[E]	530368012	SPTBN1	6711	530368012 [2326-2345]	1xPhospho [S/T];2xTMT6plex [N-Term;K19]	S7(Phospho): 16.6;T10(Phospho): 16.6;T12(Phospho): 16.6;S13(Phospho): 16.6;S15(Phospho): 16.6;S16(Phospho): 16.6	5	1.001	0.811654
[R].SRSSSVGSSSSYPISPAVSR.[T]	530389151	PLEC	5339	530389151 [4250-4269]	1xTMT6plex [N-Term];2xPhospho [S/Y]	S1(Phospho): 48.68;S3(Phospho): 48.68;S4(Phospho): 48.68;S5(Phospho): 47.26	6	0.921	0.810600
[R].AKTQTPPVSPAPQPTEER.[L]	296080746	CTTN	2017	296080746 [360-377]	2xPhospho [T5(100);2xTMT6plex [N-Term;K2];S9(100)]	T5(Phospho): 99.97;S9(Phospho): 100	8	1.176	0.809497
[R].SRTSVQTEDDQLIAGQSAR.[A]	1022433203	CTNNA1	1495	1022433203 [652-670]	1xTMT6plex [N-Term];2xPhospho [S1(96.7);T7(98.5)]	S1(Phospho): 96.69;T7(Phospho): 100	2	3.338	0.809226
[R].NRENSPSSQSAGLSSINK.[E]	530406292	ZNF106	64397	530406292 [1298-1315]	1xPhospho [S5(99.8)];2xTMT6plex [N-Term;K18]	S5(Phospho): 99.84	1	1.482	0.807928
[R].APSTYGGGLSVSSSR.[F]	15431310	KRT14	3861	15431310 [42-56]	1xPhospho [S3(92.4)];1xTMT6plex [N-Term]	S3(Phospho): 92.43	1	1.548	0.807864
[K].AGLESGAEPGDGDSDTTKK.[K]	4503337	DKC1	1736	4503337 [481-499]	1xPhospho [S14(97.4)];3xTMT6plex [N-Term;K18;K19]	S14(Phospho): 99.98	2	1.816	0.807662
[R].EGSPIPHDPEFGSK.[L]	82546824	FOXK1	221937	82546824 [443-456]	1xPhospho [S3(100)];2xTMT6plex [N-Term;K14]	S3(Phospho): 100	2	1.258	0.807043
[R].SYSPDGKESPSDKK.[S]	62750354	MATR3	9782	62750354 [596-609]	1xPhospho [S/Y];4xTMT6plex [N-Term;K13;K14];K7	S3(Phospho): 98.28	1	1.116	0.806372
[R].RRGDSESEEDEQDSEEVR.[LA]	7706557	C9orf78	51759	7706557 [11-28]	1xTMT6plex [N-Term];2xPhospho [S5(100);S]	S5(Phospho): 99.97;S7(Phospho): 50.01;S14(Phospho): 50.01	1	0.245	0.805552
[R].SSGHSSSELSPDAVEK.[A]	118572613	SRRM2	23524	118572613 [1378-1393]	2xPhospho [S7(97.9);2xTMT6plex [N-Term;K16];S]	S5(Phospho): 98.66;S6(Phospho): 49.67;S7(Phospho): 50.33	8	0.823	0.805070
[K].AGLESGAEPGDGDSDTTKKK.[K]	4503337	DKC1	1736	4503337 [481-500]	1xPhospho [S/T];4xTMT6plex [N-Term;K18;K19;K20]	S14(Phospho): 32.8;T16(Phospho): 32.8;T17(Phospho): 32.8	1	1.376	0.804486
[R].APESPRVPSPPPSPR.[L]	388240778	PLEKHG5	57449	388240778 [922-936]	1xTMT6plex [N-Term];3xPhospho [S4(100);S13(100);S9(100)]	S4(Phospho): 100;S9(Phospho): 100;S13(Phospho): 100	2	0.775	0.804028
[K].GYYSPYSVSGSGSTAGSR.[T]	530389151	PLEC	5339	530389151 [4478-4495]	1xPhospho [T/S/Y];1xTMT6plex [N-Term]	S9(Phospho): 48.88;S11(Phospho): 48.88	32	1.185	0.803311
[R].GGQGDPAVPTQQPADPSTPEQQNSPSGSEQFVR.[R]	1034675221	ZNF185	7739	1034675221 [573-605]	1xTMT6plex [N-Term];2xPhospho [T10(93.4);S24(96.2)]	T10(Phospho): 93.41;S24(Phospho): 99.6	10	0.884	0.802508
[K].ESSPIPSPTSDR.[K]	530368012	SPTBN1	6711	530368012 [2163-2174]	1xTMT6plex [N-Term];2xPhospho [S3(100);S7(98.4)]	S3(Phospho): 99.97;S7(Phospho): 98.42	1	0.687	0.802351
[R].EAPGSPPLSPR.[G]	1034573119	BCL9L	283149	1034573119 [65-75]	1xTMT6plex [N-Term];2xPhospho [S5(100);S9(100)]	S5(Phospho): 100;S9(Phospho): 100	4	1.061	0.802291
[R].TPGSRQASPTEVVER.[L]	530367033	PUM2	23369	530367033 [175-189]	1xTMT6plex [N-Term];2xPhospho [S/T]	S4(Phospho): 99.97;S8(Phospho): 99.97	3	0.969	0.800480
[R].GGGTPDANSLAPPGK.[A]	23510333	PYGO2	90780	23510333 [299-313]	1xPhospho [T4(100)];2xTMT6plex [N-Term;K15]	T4(Phospho): 100	1	0.597	0.799452
[R].RSPSPYSLK.[C]	530381792	BRPF3	27154	530381792 [16-24]	2xPhospho [S2(100);2xTMT6plex [N-Term;K9];S4(96.5)]	S2(Phospho): 100;S4(Phospho): 100	1	0.522	0.799387
[R].NTDVAQSPEAPKQEAPAK.[K]	110611218	RRBP1	6238	110611218 [179-196]	1xPhospho [S7(98.6)];3xTMT6plex [N-Term;K12;K18]	S7(Phospho): 98.64	1	1.563	0.799385
[R].AGDPGEMPQSPTGLGQPK.[R]	767972932	BAZ2A	11176	767972932 [1388-1405]	1xOxidation [M7];1xPhospho [S/T];2xTMT6plex [N-Term;K18]	S10(Phospho): 50;T12(Phospho): 50	4	1.272	0.798606
[K].YADSPGASSPEQPKR.[K]	27363484	ELF1	1997	27363484 [160-174]	2xPhospho [S/Y];2xTMT6plex [N-Term;K14]	S4(Phospho): 66.67;S8(Phospho): 66.67;S9(Phospho): 66.67	1	0.722	0.798118
[K].VSNLQVSPK.[S]	1034574239	PIK3C2A	5286	1034574239 [253-261]	1xPhospho [S7(100)];2xTMT6plex [N-Term;K9]	S7(Phospho): 100	2	1.676	0.796799
[R].KVVDYSQFQESDDADEDYGRDSGPPTK.[K]	56118310	NUCKS1	64710	56118310 [9-35]	1xPhospho [T26(95.6)];3xTMT6plex [N-Term;K1;K27]	S11(Phospho): 99.94	2	1.273	0.796072
[R].LLSSNEDDANILSSPTDR.[S]	1057503224	MED24	9862	1057503224 [879-896]	1xTMT6plex [N-Term];2xPhospho [S/T]	S3(Phospho): 49.98;S4(Phospho): 49.98;S13(Phospho): 49.42;S14(Phospho): 49.42	1	2.047	0.795322
[R].GSGGSSGDELREDDEPVK.[K]	1131345319	HDGFL2	84717	1131345319 [389-406]	2xPhospho [S5(97);2xTMT6plex [N-Term;K18];S6(99.9)]	S5(Phospho): 96.99;S6(Phospho): 99.89	1	2.762	0.795119
[K].SPAVATSTAAPPPPSSPLPSK.[S]	530365733	UBAP2L	9898	530365733 [450-470]	1xPhospho [S/T];2xTMT6plex [N-Term;K21]	S7(Phospho): 19.89;T8(Phospho): 19.89;S15(Phospho): 19.89;S16(Phospho): 19.89;S20(Phospho): 19.89	18	0.693	0.794957
[K].LSVPTSDEEDEVPAPKPR.[G]	69354671	ABCF1	23	69354671 [104-121]	1xPhospho [T/S];2xTMT6plex [N-Term;K16]	T5(Phospho): 98.51	3	0.757	0.793572
[K].SRSPVDLR.[G]	1034652055	PRPF4B	8899	1034652055 [292-299]	1xTMT6plex [N-Term];2xPhospho [S1(100);S3(100)]	S1(Phospho): 100;S3(Phospho): 100	1	1.038	0.793079
[R].TASQGPQTDSVIQNSENIK.[A]	7662088	ARHGEF12	23365	7662088 [1286-1304]	1xPhospho [T/S];2xTMT6plex [N-Term;K19]	T1(Phospho): 50;S3(Phospho): 50	2	0.891	0.791927
[R].DSHSSEEDEASSQTDLSQTISK.[K]	767908953	TOR1AIP1	26092	767908953 [153-174]	1xPhospho [S/T];2xTMT6plex [N-Term;K22]	S4(Phospho): 49.72;S5(Phospho): 49.72	4	0.948	0.790786
[K].FLESPSRSSSPANQR.[A]	578807557	YEATS2	55689	578807557 [115-129]	1xTMT6plex [N-Term];2xPhospho [S]	S6(Phospho): 97.05;S8(Phospho): 33.82;S9(Phospho): 33.82;S10(Phospho): 33.82	1	1.185	0.790114
[K].LSSSDRYSDASDDSFSEPR.[I]	768032583	CTPS2	56474	768032583 [603-621]	1xTMT6plex [N-Term];2xPhospho [S/Y]	Y7(Phospho): 48.65;S8(Phospho): 48.65;S14(Phospho): 49.54;S16(Phospho): 49.54	3	0.775	0.788874
[K].FASDDEHDEHDENGATGPVK.[R]	10835067	SSB	6741	10835067 [364-383]	1xPhospho [S3(100)];2xTMT6plex [N-Term;K20]	S3(Phospho): 100	2	0.666	0.788045
[K].ESSTSEGADQKMSSPEDDSDTKR.[L]	321267469	SAFB	6294	321267469 [371-393]	1xOxidation [M12];2xPhospho [S/T];3xTMT6plex [N-Term;K11;K22]	S13(Phospho): 98.98;S14(Phospho): 98.98	1	1.792	0.787095
[R].AVLSPGSVFSPGR.[G]	194272161	ITPKB	3707	194272161 [40-52]	1xTMT6plex [N-Term];2xPhospho [S4(98.8);S]	S4(Phospho): 98.81;S7(Phospho): 50.59;S10(Phospho): 50.59	2	1.082	0.787019
[K].TSQVGAASAPAKESPR.[K]	530380665	TCOF1	6949	530380665 [368-383]	1xPhospho [S14(100)];2xTMT6plex [N-Term;K12]	S14(Phospho): 100	2	0.434	0.786702
[R].SRSGSSPEVDSK.[S]	118572613	SRRM2	23524	118572613 [1577-1588]	2xPhospho [S1(99);2xTMT6plex [N-Term;K12];S]	S1(Phospho): 98.99;S5(Phospho): 50;S6(Phospho): 50	1	2.311	0.786454
[R].SSSVGSSSSYPISPAVSR.[T]	530389151	PLEC	5339	530389151 [4252-4269]	1xTMT6plex [N-Term];3xPhospho [S/Y]	S3(Phospho): 97.74;S6(Phospho): 99.95;S13(Phospho): 100	5	1.219	0.785902
[K].QKSDAEEDGGTVSQEEEDR.[K]	767938662	CANX	821	767938662 [606-624]	2xPhospho [S3(100);2xTMT6plex [N-Term;K2];T/S]	S3(Phospho): 100;T11(Phospho): 50;S13(Phospho): 50	1	1.015	0.784968
[R].VVSSTSEEEEAFTEK.[F]	309384265	OXR1	55074	309384265 [199-213]	2xPhospho [S/T];2xTMT6plex [N-Term;K15]	S3(Phospho): 50;S4(Phospho): 50;T5(Phospho): 49.99;S6(Phospho): 49.99	1	1.182	0.783671
[R].RYPSSISSSPQK.[D]	530365733	UBAP2L	9898	530365733 [612-623]	1xPhospho [S/Y];2xTMT6plex [N-Term;K12]	S9(Phospho): 98.67	2	0.325	0.783577
[K].RGSIGENQIKDEK.[I]	1034656986	CALD1	800	1034656986 [200-212]	1xPhospho [S3(100)];3xTMT6plex [N-Term;K10;K13]	S3(Phospho): 100	1	0.696	0.783549
[K].GPPDFSSDEEREPTPVLGSGAAAAGR.[S]	4507555	TMPO	7112	4507555 [61-86]	1xPhospho [T14(100)];1xTMT6plex [N-Term]	T14(Phospho): 100	1	1.614	0.783239
[R].GAEAFGDSEEDGEDVFEVEK.[I]	767977796	MPHOSPH8	54737	767977796 [44-63]	1xPhospho [S8(100)];2xTMT6plex [N-Term;K20]	S8(Phospho): 100	1	1.837	0.783197
[K].ALTDDSDENEEEDAFTDQK.[I]	767901811	PATJ	10207	767901811 [1207-1225]	2xPhospho [T3(99.9);2xTMT6plex [N-Term;K19];S6(95.1)]	T3(Phospho): 99.93;S6(Phospho): 97.63	2	1.007	0.782945
[R].SQSESSDEVTELDLSHGK.[K]	1034582490	C2CD5	9847	1034582490 [767-784]	2xPhospho [S/T];2xTMT6plex [N-Term;K18]	S3(Phospho): 99.96;S5(Phospho): 50.01;S6(Phospho): 50.01	7	1.074	0.782568
[R].DIEISTEEEKDTGDLK.[D]	388490152	LARP7	51574	388490152 [340-355]	2xPhospho [S/T];3xTMT6plex [N-Term;K10;K16]	S5(Phospho): 50.32;T6(Phospho): 50.32;T12(Phospho): 99.36	2	0.624	0.782003
[K].TSQPPVPQGEAEEDSQGKEGPSGSGDSQLSASSR.[S]	101943240	GTF3C1	2975	101943240 [725-758]	1xPhospho [S/T];2xTMT6plex [N-Term;K18]	S15(Phospho): 49.26;S22(Phospho): 49.26	4	0.771	0.780354
[K].AASDDGSLK.[S]	1093953582	MYO18A	399687	1093953582 [2000-2008]	2xPhospho [S3(100);2xTMT6plex [N-Term;K9];S7(100)]	S3(Phospho): 100;S7(Phospho): 100	2	0.556	0.780265
[K].EEEEGISQESSEEEQ.[-]	22208967	HMGA1	3159	22208967 [93-107]	1xTMT6plex [N-Term];3xPhospho [S7(100);S10(100);S11(100)]	S7(Phospho): 100;S10(Phospho): 100;S11(Phospho): 100	4	0.568	0.779141
[K].KIPDPDSDDVSEVDAR.[H]	192807323	SMARCA4	6597	192807323 [689-704]	2xPhospho [S7(100);2xTMT6plex [N-Term;K1];S11(100)]	S7(Phospho): 100;S11(Phospho): 100	1	1.257	0.778082
[R].KSPSGPVKSPPLSPVGTTPVK.[L]	1370478960	C2orf49	79074	1370478960 [181-201]	2xPhospho [S13(91.4);4xTMT6plex [N-Term;K1;K21];K8;S/T]	S2(Phospho): 34.69;S4(Phospho): 34.69;S9(Phospho): 34.69;S13(Phospho): 91.43	1	1.735	0.777778
[R].TLQPPESPR.[L]	767969840	PHLDB1	23187	767969840 [483-491]	1xPhospho [S7(100)];1xTMT6plex [N-Term]	S7(Phospho): 100	1	0.586	0.777632
[R].SGFSSVSVSR.[S]	5031839	KRT6A	3853	5031839 [31-40]	1xTMT6plex [N-Term];2xPhospho [S4(98.7);S7(100)]	S4(Phospho): 99.98;S7(Phospho): 100	2	3.125	0.776870
[K].AEDGATPSPSNETPK.[K]	153070260	MARCKS	4082	153070260 [138-152]	2xPhospho [S/T];2xTMT6plex [N-Term;K15]	T6(Phospho): 33.34;S8(Phospho): 33.34;S10(Phospho): 33.34;T13(Phospho): 99.99	4	0.575	0.776634
[R].DSALQDTDDSDDDPVLIPGAR.[Y]	312176366	DCAF6	55827	312176366 [725-745]	1xTMT6plex [N-Term];2xPhospho [T/S]	T7(Phospho): 100;S10(Phospho): 100	3	0.951	0.775682
[R].SGSSQELDVKPSASPQER.[S]	118572613	SRRM2	23524	118572613 [1539-1556]	2xPhospho [S];2xTMT6plex [N-Term;K10]	S3(Phospho): 49.6;S4(Phospho): 49.6;S12(Phospho): 50;S14(Phospho): 50	2	2.044	0.775554
[R].GEFSASPMLK.[S]	118572613	SRRM2	23524	118572613 [1119-1128]	1xOxidation [M8];1xPhospho [S6(98.9)];2xTMT6plex [N-Term;K10]	S6(Phospho): 98.86	2	1.571	0.774931
[R].NADSEIKHSTPSPTR.[Y]	530368768	RANBP2	5903	530368768 [770-784]	2xPhospho [S12(97);2xTMT6plex [N-Term;K7];S/T]	S9(Phospho): 59.98;T10(Phospho): 59.98;S12(Phospho): 40;T14(Phospho): 40	5	0.585	0.774767
[K].YSPTSPTYSPTSPVYTPTSPK.[Y]	4505939	POLR2A	5430	4505939 [1888-1908]	2xPhospho [T/S/Y];2xTMT6plex [N-Term;K21]	Y1(Phospho): 18.1;S2(Phospho): 18.1;T4(Phospho): 18.1;S5(Phospho): 18.1;T7(Phospho): 18.1;Y8(Phospho): 18.1;S9(Phospho): 18.1;T11(Phospho): 18.1;S12(Phospho): 18.1;Y15(Phospho): 18.1;T16(Phospho): 18.1	1	1.238	0.773707
[R].LSSETGGMGSS.[-]	14141168	PCBP2	5094	14141168 [356-366]	1xOxidation [M8];1xPhospho [S/T];1xTMT6plex [N-Term]	S10(Phospho): 50;S11(Phospho): 50	2	0.971	0.772813
[R].TASNPKVENEDEPVR.[L]	767909551	PI4KB	5298	767909551 [304-318]	1xPhospho [S3(100)];2xTMT6plex [N-Term;K6]	S3(Phospho): 99.99	1	0.537	0.771889
[K].SVNEILGLAESSPNEPK.[A]	262527223	CAAP1	79886	262527223 [301-317]	1xPhospho [S];2xTMT6plex [N-Term;K17]	S11(Phospho): 50;S12(Phospho): 50	2	2.593	0.771369
[K].ADKLAEEHSS.[-]	32189394	ATP5F1B	506	32189394 [520-529]	1xPhospho [S];2xTMT6plex [N-Term;K3]	S9(Phospho): 50;S10(Phospho): 50	2	2.445	0.768887
[R].TPESQPDTPPGTPLVSQDEKR.[D]	31543419	PNKP	11284	31543419 [111-131]	2xPhospho [T8(97.9);2xTMT6plex [N-Term;K20];T12(97.9)]	T8(Phospho): 100;T12(Phospho): 100	3	1.181	0.768412
[K].SFNYSPNSSTSEVSSTSASK.[A]	183396804	RPRD2	23248	183396804 [589-608]	1xPhospho [Y/S/T];2xTMT6plex [N-Term;K20]	S5(Phospho): 98.66	5	1.09	0.767828
[K].KETESEAEDNLDDLEK.[H]	1034554585	SRRM1	10250	1034554585 [909-924]	1xPhospho [T/S];3xTMT6plex [N-Term;K1;K16]	T3(Phospho): 50;S5(Phospho): 50	2	2.385	0.766688
[K].RTGSNISGASSDISLDEQYK.[H]	4505531	OSBP	5007	4505531 [376-395]	2xPhospho [S7(98.2);2xTMT6plex [N-Term;K20];T/S/Y]	T2(Phospho): 50.73;S4(Phospho): 50.73;S7(Phospho): 98.21	1	0.359	0.766505
[R].VAHEPVAPPEDKESESEAKVDGETASDSESR.[A]	20143480	CDS2	8760	20143480 [8-38]	2xPhospho [S/T];3xTMT6plex [N-Term;K12;K19]	S14(Phospho): 50;S16(Phospho): 50;T24(Phospho): 49.96;S26(Phospho): 49.96	3	1.22	0.766443
[R].TDRGGDSIGETPTPGASK.[R]	54112117	SF3B1	23451	54112117 [316-333]	2xPhospho [T11(97.7);2xTMT6plex [N-Term;K18];T13(99.9)]	T11(Phospho): 97.71;T13(Phospho): 99.94	1	1.653	0.766275
[R].SLGGQQGSPK.[H]	767924770	IQSEC1	9922	767924770 [414-423]	1xPhospho [S8(97.6)];2xTMT6plex [N-Term;K10]	S8(Phospho): 100	2	0.816	0.765120
[R].EGAGSPAAPGTLPLEK.[V]	5902106	SOX15	6665	5902106 [33-48]	1xPhospho [T11(97.6)];2xTMT6plex [N-Term;K16]	S5(Phospho): 50;T11(Phospho): 50	1	0.538	0.764941
[R].SRSLSNSNPDISGTPTSPDDEVR.[S]	767906494	DOCK7	85440	767906494 [894-916]	1xTMT6plex [N-Term];2xPhospho [S3(98.1);S5(98.1)]	S3(Phospho): 97.91;S5(Phospho): 99.95	3	0.973	0.764881
[R].TSSFTEQLDEGTPNR.[E]	530372318	GOLGA4	2803	530372318 [39-53]	1xPhospho [S/T];1xTMT6plex [N-Term]	S3(Phospho): 96.52	4	0.793	0.764659
[R].KAAVLSDSEDEEK.[A]	530369278	IWS1	55677	530369278 [398-410]	2xPhospho [S6(100);3xTMT6plex [N-Term;K1;K13];S8(100)]	S6(Phospho): 100;S8(Phospho): 100	1	0.667	0.764574
[R].SASPYPSHSLSSPQR.[K]	530402186	ZC3H13	23091	530402186 [370-384]	1xTMT6plex [N-Term];3xPhospho [S/Y]	S1(Phospho): 100;S3(Phospho): 99.98;S11(Phospho): 50;S12(Phospho): 50	1	0.608	0.764172
[R].LSSSSSNDSGNK.[V]	1079280806	AHNAK	79026	1079280806 [5862-5873]	1xPhospho [S];2xTMT6plex [N-Term;K12]	S3(Phospho): 99.26	1	0.953	0.764091
[R].EQGTESRSSTPLPTISSSAENTR.[Q]	4507555	TMPO	7112	4507555 [151-173]	1xTMT6plex [N-Term];2xPhospho [S/T]	S8(Phospho): 65.37;S9(Phospho): 65.37;T10(Phospho): 65.37	2	0.613	0.763744
[R].STPLASPSPSPGRSPQR.[L]	578802190	AHCTF1	25909	578802190 [1244-1260]	1xPhospho [T/S];1xTMT6plex [N-Term]	S14(Phospho): 99.93	6	1.022	0.763520
[R].RSSSDEQGLSYSSLK.[N]	268840316	IL1RAP	3556	268840316 [554-568]	1xPhospho [S/Y];2xTMT6plex [N-Term;K15]	S2(Phospho): 33.33;S3(Phospho): 33.33;S4(Phospho): 33.33	6	2.192	0.763507
[K].KRPSLPSSPSPGLPK.[A]	109638745	TGFB1I1	7041	109638745 [134-148]	2xPhospho [S4(100);3xTMT6plex [N-Term;K1;K15];S]	S4(Phospho): 99.98;S7(Phospho): 50.01;S8(Phospho): 50.01	1	0.618	0.763072
[K].SELGNQSPSTSSR.[Q]	767908953	TOR1AIP1	26092	767908953 [325-337]	1xPhospho [S7(77.8)];1xTMT6plex [N-Term]	S7(Phospho): 99.98	2	0.904	0.762430
[R].RSSQPSPTAVPASDSPPTK.[Q]	78042577	MAP7D1	55700	78042577 [111-129]	2xTMT6plex [N-Term;3xPhospho [S/T];K19]	S2(Phospho): 49.96;S3(Phospho): 49.96;S6(Phospho): 49.96;T8(Phospho): 49.96;S13(Phospho): 49.06;S15(Phospho): 49.06	3	0.839	0.762300
[R].EREVDEDSEPEREVR.[A]	56550051	USP39	10713	56550051 [75-89]	1xPhospho [S8(100)];1xTMT6plex [N-Term]	S8(Phospho): 100	1	1.818	0.761639
[K].TAQALSSGSGSQETK.[I]	767924844	OXSR1	9943	767924844 [470-484]	1xPhospho [T14(99.3)];2xTMT6plex [N-Term;K15]	S11(Phospho): 50;T14(Phospho): 50	6	0.953	0.761417
[K].AELLQSDENGIPSSPQK.[V]	767968032	SLC29A2	3177	767968032 [239-255]	2xPhospho [S6(97.3);2xTMT6plex [N-Term;K17];S13(97.3)]	S6(Phospho): 97.23;S13(Phospho): 97.23	1	1.557	0.760750
[R].LTPVSPESSSTEEK.[S]	4505571	SQSTM1	8878	4505571 [268-281]	2xPhospho [S5(95.4);2xTMT6plex [N-Term;K14];S/T]	S5(Phospho): 98.35;S8(Phospho): 25.01;S9(Phospho): 25.01;S10(Phospho): 25.01;T11(Phospho): 25.01	2	0.866	0.760281
[R].TASPPPPPKR.[R]	1034554585	SRRM1	10250	1034554585 [653-662]	1xPhospho [S/T];2xTMT6plex [N-Term;K9]	S3(Phospho): 99.14	3	1.274	0.760088
[R].KASSPSPLTIGTPESQR.[K]	291327466	DCP1A	55802	291327466 [520-536]	2xPhospho [S3(100);2xTMT6plex [N-Term;K1];S/T]	S3(Phospho): 98.19;S6(Phospho): 99.93	3	0.726	0.759883
[R].SLDSPTSSPGAGTR.[Q]	91208437	GMIP	51291	91208437 [434-447]	1xTMT6plex [N-Term];2xPhospho [S4(100);T/S]	S4(Phospho): 99.96;T6(Phospho): 33.14;S7(Phospho): 33.14;S8(Phospho): 33.14	2	0.684	0.759880
[R].TGDLGIPPNPEDRSPSPEPIYNSEGK.[R]	295842330	SF1	7536	295842330 [192-217]	2xPhospho [S/Y/T];2xTMT6plex [N-Term;K26]	S14(Phospho): 65.46;S16(Phospho): 65.46;Y21(Phospho): 65.46	2	0.786	0.759752
[R].KGAGDGSDEEVDGKADGAEAKPAE.[-]	1034628998	MYH9	4627	1034628998 [1937-1960]	1xPhospho [S7(100)];4xTMT6plex [N-Term;K1;K14;K21]	S7(Phospho): 100	2	0.392	0.756551
[K].GPPDFSSDEEREPTPVLGSGAAAAGR.[S]	4507555	TMPO	7112	4507555 [61-86]	1xTMT6plex [N-Term];2xPhospho [S7(97.7);T14(99.9)]	S7(Phospho): 97.8;T14(Phospho): 99.95	9	0.824	0.756225
[R].GTGSGGQLQDLDCSSSDDEGAAQNSTKPSATK.[G]	23308697	SRPRA	6734	23308697 [283-314]	1xCarbamidomethyl [C13];1xPhospho [S/T];3xTMT6plex [N-Term;K27;K32]	S14(Phospho): 31.9;S15(Phospho): 31.9;S16(Phospho): 31.9	3	2.122	0.755889
[R].EAAAESDRELNEDDSTDCDDDVQR.[E]	21314654	RNF139	11236	21314654 [620-643]	1xCarbamidomethyl [C18];1xTMT6plex [N-Term];3xPhospho [S6(100);S15(100);T16(100)]	S6(Phospho): 100;S15(Phospho): 100;T16(Phospho): 100	3	0.926	0.754668
[R].STLQDSDEYSNPAPLPLDQHSR.[K]	221316659	LPXN	9404	221316659 [19-40]	1xPhospho [S/T/Y];1xTMT6plex [N-Term]	S1(Phospho): 48.38;S6(Phospho): 48.38	1	0.6	0.754382
[K].VVSQSQPGSRSSSPGK.[L]	1034612655	CLASP1	23332	1034612655 [683-698]	2xPhospho [S];2xTMT6plex [N-Term;K16]	S9(Phospho): 49.57;S11(Phospho): 49.57;S12(Phospho): 49.57;S13(Phospho): 49.57	2	0.352	0.753987
[K].LKFSDEEDGRDSDEEGAEGHR.[D]	149158690	PRRC2A	7916	149158690 [339-359]	2xPhospho [S4(100);2xTMT6plex [N-Term;K2];S12(100)]	S4(Phospho): 100;S12(Phospho): 100	4	0.337	0.753752
[K].AAEEQGDDQDSEKSKPAGSDGERR.[G]	118601081	HNRNPUL2	221092	118601081 [175-198]	1xPhospho [S];3xTMT6plex [N-Term;K13;K15]	S11(Phospho): 33.33;S14(Phospho): 33.33;S19(Phospho): 33.33	3	1.599	0.753154
[R].GRLTPSPDIIVLSDNEASSPR.[S]	530365128	GATAD2B	57459	530365128 [117-137]	1xTMT6plex [N-Term];3xPhospho [T4(99.8);S];S6(92.7)	T4(Phospho): 99.82;S6(Phospho): 92.66;S18(Phospho): 50.08;S19(Phospho): 50.08	1	1.383	0.753010
[K].ATVTPSPVKGK.[G]	56118310	NUCKS1	64710	56118310 [176-186]	1xPhospho [S6(100)];3xTMT6plex [N-Term;K11];K9	S6(Phospho): 100	1	0.272	0.752212
[K].VQIPVSRPDPEPVSDNEEDSYDEEIHDPR.[S]	1034591669	TJP1	7082	1034591669 [205-233]	1xTMT6plex [N-Term];2xPhospho [S/Y]	S14(Phospho): 66.67;S20(Phospho): 66.67;Y21(Phospho): 66.67	11	0.538	0.752087
[K].ETQSPEQVK.[S]	7661958	BCLAF1	9774	7661958 [493-501]	1xPhospho [S4(100)];2xTMT6plex [N-Term;K9]	S4(Phospho): 100	7	1.028	0.751491
[R].ASSRVSGSFPEDSSK.[E]	170932494	STX7	8417	170932494 [124-138]	1xPhospho [S2(98.5)];2xTMT6plex [N-Term;K15]	S2(Phospho): 98.48	1	1.386	0.750860
[K].TSRDTSPSSGSAVSSSK.[V]	1034579978	PPHLN1	51535	1034579978 [207-223]	2xPhospho [S2(93.8);2xTMT6plex [N-Term;K17];S/T]	S2(Phospho): 98.06;T5(Phospho): 49.53;S6(Phospho): 49.53	6	0.765	0.750684
[K].AGPLGGSSYEEEEEEEEGGGGER.[K]	42560237	NECTIN1	5818	42560237 [428-451]	1xTMT6plex [N-Term];2xPhospho [S7(100);S8(98)]	S7(Phospho): 98.03;S8(Phospho): 98.03	2	2.109	0.750299
[K].GHYEVTGSDDETGK.[L]	1079280806	AHNAK	79026	1079280806 [5834-5847]	1xPhospho [T/S/Y];2xTMT6plex [N-Term;K14]	S8(Phospho): 99.17	2	0.465	0.750248
[R].FVSSGSGGGYGGGMR.[V]	767994709	KRT15	3866	767994709 [51-65]	1xOxidation [M14];1xPhospho [S6(92.5)];1xTMT6plex [N-Term]	S4(Phospho): 33.07;S6(Phospho): 33.07;Y10(Phospho): 33.07	1	2.532	0.750039
[K].ELSNSPLRENSFGSPLEFR.[N]	118572613	SRRM2	23524	118572613 [1316-1334]	1xTMT6plex [N-Term];2xPhospho [S]	S5(Phospho): 65.16;S11(Phospho): 66.25;S14(Phospho): 66.25	1	1.245	0.749435
[K].TPTPSTPGDTQPNTPAPVPPAEDGIK.[I]	1370461044	CHD4	1108	1370461044 [1568-1593]	2xPhospho [T/S];2xTMT6plex [N-Term;K26]	S5(Phospho): 49.32;T6(Phospho): 49.32;T10(Phospho): 49.98;T14(Phospho): 49.98	2	0.971	0.748782
[R].YSHSGSSSPDTK.[V]	118572613	SRRM2	23524	118572613 [967-978]	2xPhospho [S/T/Y];2xTMT6plex [N-Term;K12]	S4(Phospho): 99.98;S6(Phospho): 99.97	13	1.123	0.748494
[R].TASGSSVTSLDGTR.[S]	767952381	NDRG1	10397	767952381 [345-358]	1xTMT6plex [N-Term];2xPhospho [S/T]	S5(Phospho): 98.69;S6(Phospho): 99.98	22	0.584	0.747888
[R].NLSNEELTK.[S]	226371723	FCHO2	115548	226371723 [401-409]	1xPhospho [S3(100)];2xTMT6plex [N-Term;K9]	S3(Phospho): 100	2	0.407	0.746738
[K].KGNSPNSEPPTPK.[T]	149158690	PRRC2A	7916	149158690 [377-389]	1xPhospho [S4(100)];3xTMT6plex [N-Term;K1;K13]	S4(Phospho): 100	2	0.841	0.746413
[R].TPSSSPPITPPASETK.[I]	555943897	DTL	51514	555943897 [508-523]	2xPhospho [T/S];2xTMT6plex [N-Term;K16]	S4(Phospho): 65.95;S5(Phospho): 65.95;T9(Phospho): 65.95	3	0.918	0.744782
[R].LRLSPSPTSQRSR.[G]	27436946	LMNA	4000	27436946 [387-399]	1xTMT6plex [N-Term];2xPhospho [S4(100);S/T]	S4(Phospho): 100;S6(Phospho): 99.98	1	2.97	0.743390
[K].KLEGNSPQGSNQGVK.[I]	16933567	RAB8A	4218	16933567 [176-190]	1xPhospho [S];3xTMT6plex [N-Term;K1;K15]	S10(Phospho): 97.32	1	1.512	0.743383
[R].ISISTSGGSFR.[N]	119395754	KRT5	3852	119395754 [74-84]	1xTMT6plex [N-Term];2xPhospho [S4(100);S9(100)]	S4(Phospho): 99.98;S9(Phospho): 100	1	0.535	0.743221
[K].RPESPSEISPIK.[G]	767962262	WAPL	23063	767962262 [218-229]	1xPhospho [S4(98.5)];2xTMT6plex [N-Term;K12]	S4(Phospho): 99.1	1	0.969	0.743172
[K].SDDSKSSSPELVTHLK.[W]	767965879	ARHGAP1	392	767965879 [44-59]	2xPhospho [S/T];3xTMT6plex [N-Term;K16];K5	S4(Phospho): 33.78;S6(Phospho): 33.78;S7(Phospho): 33.78;S8(Phospho): 98.65	8	0.74	0.742371
[K].ATVTPSPVK.[G]	56118310	NUCKS1	64710	56118310 [176-184]	2xPhospho [T4(100);2xTMT6plex [N-Term;K9];S6(100)]	T4(Phospho): 99.97;S6(Phospho): 100	1	0.216	0.742314
[R].LPSTSGSEGVPFR.[T]	32967311	EPHA2	1969	32967311 [895-907]	1xPhospho [S/T];1xTMT6plex [N-Term]	S7(Phospho): 96.76	1	1.496	0.741689
[K].TSTSPPPEKSGDEGSEDEAPSGED.[-]	56118310	NUCKS1	64710	56118310 [220-243]	2xPhospho [S21(97.4);2xTMT6plex [N-Term;K9];S/T]	S2(Phospho): 24.79;T3(Phospho): 24.79;S4(Phospho): 24.79;S10(Phospho): 25.7;S15(Phospho): 49.49;S21(Phospho): 49.49	1	1.399	0.740735
[K].ASQSRPNSSALETLGGEK.[L]	126362971	STK10	6793	126362971 [447-464]	1xPhospho [S/T];2xTMT6plex [N-Term;K18]	S8(Phospho): 49.23;S9(Phospho): 49.23	1	0.369	0.739845
[R].LQQGAGLESPQGQPEPGAASPQR.[Q]	13129104	CCDC86	79080	13129104 [72-94]	1xPhospho [S20(100)];1xTMT6plex [N-Term]	S20(Phospho): 100	9	0.879	0.739632
[R].SHDSDDSDYASSK.[H]	767994135	GPATCH8	23131	767994135 [937-949]	2xPhospho [S7(98.8);2xTMT6plex [N-Term;K13];S/Y]	S1(Phospho): 50;S4(Phospho): 50;S7(Phospho): 98.85	1	0.648	0.738480
[R].LQSASPSTR.[W]	119829187	COL17A1	1308	119829187 [146-154]	1xTMT6plex [N-Term];2xPhospho [S3(100);S5(100)]	S3(Phospho): 100;S5(Phospho): 98.59	4	0.89	0.738141
[K].GSEGYLAATYPTVGQTSPR.[A]	109826564	NF1	4763	109826564 [2499-2517]	1xPhospho [T/S/Y];1xTMT6plex [N-Term]	T12(Phospho): 32.88;T16(Phospho): 32.88;S17(Phospho): 32.88	3	1.133	0.736105

Patil et al,2020. Proteomic and phosphoproteomic profiling of shammah-induced signaling in oral keratinocytes

Supplementary Table S2: List of phosphopeptides identified and quantified in OKF6/TERT1-Shammah and OKF6/TERT1-Parental phosphoproteome using TMT/TiO_2-based quantitative phosphoproteomic approach

Peptide Sequence	Protein Accessions	Gene Symbol	Gene ID	Positions in Proteins	Modifications	PhosphoRS: Best Site Probabilities	# PSMs	Fold change	p-value
[K].RGTSPRPPEGGLGYSQLGDDDLK.[E]	767906898	CDK11B	984	767906898 [758-780]	2xPhospho [T3(100);2xTMT6plex [N-Term;K23];S/Y]	T3(Phospho): 100;S4(Phospho): 100	3	0.644	0.734780
[R].DSSFTEVPRSPK.[H]	1370457682	SVIL	6840	1370457682 [236-247]	2xPhospho [S3(91.3);2xTMT6plex [N-Term;K12];T/S]	S3(Phospho): 98.85;S10(Phospho): 100	1	1.374	0.734572
[K].ASQSRPNSSALETLGGEK.[L]	126362971	STK10	6793	126362971 [447-464]	2xPhospho [S/T];2xTMT6plex [N-Term;K18]	S4(Phospho): 97.47;S8(Phospho): 97.53	6	0.28	0.732487
[R].VHGEPTSDLSDID.[-]	1034628669	BRD1	23774	1034628669 [1177-1189]	1xTMT6plex [N-Term];2xPhospho [T/S]	T6(Phospho): 100;S7(Phospho): 99.98	2	1.21	0.731467
[K].KPIETGSPK.[T]	530363610	SRSF11	9295	530363610 [450-458]	1xPhospho [S7(99.3)];3xTMT6plex [N-Term;K1;K9]	S7(Phospho): 99.29	2	1.702	0.731089
[R].RPSVGSQSNQAGQGK.[R]	768036920	PHF8	23133	768036920 [1019-1033]	1xPhospho [S3(94.3)];2xTMT6plex [N-Term;K15]	S3(Phospho): 100	1	0.699	0.729790
[R].LDADSDSGDDSDKR.[S]	41393559	KIF1B	23095	41393559 [659-672]	2xPhospho [S11(98);2xTMT6plex [N-Term;K13];S]	S5(Phospho): 100;S7(Phospho): 100	2	0.327	0.726260
[K].VNFSEEGETEEDDQDSSHSSVTTVK.[A]	767908953	TOR1AIP1	26092	767908953 [213-237]	2xPhospho [S/T];2xTMT6plex [N-Term;K25]	S4(Phospho): 99.93;T9(Phospho): 33.35;S16(Phospho): 33.35;S17(Phospho): 33.35	3	0.85	0.724263
[R].THSDASDDEAFTTSK.[T]	71834863	ARHGAP5	394	71834863 [1171-1185]	2xPhospho [S/T];2xTMT6plex [N-Term;K15]	S3(Phospho): 99.99;S6(Phospho): 100	2	0.482	0.724219
[R].LNTSDFQK.[L]	400153980	AKT1S1	84335	400153980 [264-271]	1xPhospho [T3(100)];2xTMT6plex [N-Term;K8]	T3(Phospho): 100	2	0.373	0.724149
[K].THTTALAGRSPSPASGR.[R]	118572613	SRRM2	23524	118572613 [286-302]	1xTMT6plex [N-Term];2xPhospho [S10(100);S12(98.2)]	T1(Phospho): 33.15;T3(Phospho): 33.15;T4(Phospho): 33.15;S12(Phospho): 98.39	2	1.571	0.723883
[K].FTDKDQQPSGSEGEDDDAEAALKK.[E]	751868065	THUMPD1	55623	751868065 [78-101]	2xPhospho [S9(100);4xTMT6plex [N-Term;K23;K24];K4;S11(100)]	S9(Phospho): 100;S11(Phospho): 100	1	0.582	0.723152
[K].IDSNISPK.[K]	1034625197	ESF1	51575	1034625197 [148-155]	1xPhospho [S];2xTMT6plex [N-Term;K8]	S6(Phospho): 99.99	1	1.416	0.723028
[K].VDGPRSPSYGR.[S]	5902076	SRSF1	6426	5902076 [194-204]	1xTMT6plex [N-Term];2xPhospho [S6(99.1);S/Y]	S6(Phospho): 99.12;S8(Phospho): 50.44;Y9(Phospho): 50.44	2	1.163	0.722938
[R].LDTGPQSLSGKSTPQPPSGK.[T]	1370474986	GTF2F1	2962	1370474986 [450-469]	2xPhospho [S/T];3xTMT6plex [N-Term;K11;K20]	S7(Phospho): 49.7;S9(Phospho): 49.7;S12(Phospho): 49.7;T13(Phospho): 49.7	2	0.425	0.722770
[R].MLPHAPGVQMQAIPEDAIPEESGDEDEDDPDKR.[I]	13128860	HDAC1	3065	13128860 [372-404]	1xPhospho [S22(100)];2xOxidation [M1;2xTMT6plex [N-Term;K32];M10]	S22(Phospho): 100	4	1.191	0.721768
[R].YSPSQNSPIHHIPSRR.[S]	7661958	BCLAF1	9774	7661958 [284-299]	1xTMT6plex [N-Term];2xPhospho [S2(96.2);S14(100)]	S2(Phospho): 65.99;S4(Phospho): 66.49;S7(Phospho): 66.49	4	0.466	0.720678
[R].KDSLTQAQEQGNLLN.[-]	578812080	TJAP1	93643	578812080 [543-557]	1xPhospho [S3(100)];2xTMT6plex [N-Term;K1]	S3(Phospho): 99.97	1	0.64	0.719046
[K].APILIATDVASR.[G]	148613856	DDX17	10521	148613856 [469-480]	1xTMT6plex [N-Term];2xPhospho [T7(100);S11(100)]	T7(Phospho): 100;S11(Phospho): 100	1	1.463	0.718961
[R].KQGGSPDEPDSK.[A]	612407827	AKAP8L	26993	612407827 [279-290]	1xPhospho [S5(100)];3xTMT6plex [N-Term;K1;K12]	S5(Phospho): 100	2	1.87	0.716999
[K].AGMSSNQSISSPVLDAVPRTPSRER.[S]	118572613	SRRM2	23524	118572613 [1394-1418]	1xOxidation [M3];1xTMT6plex [N-Term];2xPhospho [S/T]	S10(Phospho): 49.99;S11(Phospho): 49.99;T20(Phospho): 49.99;S22(Phospho): 49.99	3	1.39	0.715801
[K].KLEKEEEEGISQESSEEEQ.[-]	22208967	HMGA1	3159	22208967 [89-107]	2xPhospho [S];3xTMT6plex [N-Term;K1;K4]	S14(Phospho): 99.91;S15(Phospho): 100	8	0.917	0.715546
[R].SEEISESESEETNAPK.[K]	55750041	ATN1	1822	55750041 [73-88]	2xPhospho [S/T];2xTMT6plex [N-Term;K16]	S5(Phospho): 50;S7(Phospho): 50;S9(Phospho): 50;T12(Phospho): 50	3	1.062	0.714625
[R].ERDHSPTPSVFNSDEER.[Y]	530376841	FIP1L1	81608	530376841 [497-513]	1xTMT6plex [N-Term];2xPhospho [S5(100);S13(100)]	S5(Phospho): 100;S13(Phospho): 100	3	0.732	0.713854
[K].ASMSEFLESEDGEVEQQR.[T]	197333809	SUZ12	23512	197333809 [538-555]	1xOxidation [M3];1xPhospho [S9(97.7)];1xTMT6plex [N-Term]	S9(Phospho): 99.92	2	1.074	0.713414
[R].SVPTAYRVSRSPGASR.[K]	387157914	SFSWAP	6433	387157914 [876-891]	1xTMT6plex [N-Term];2xPhospho [T/Y/S]	Y6(Phospho): 65.77;S9(Phospho): 65.77;S11(Phospho): 65.77	2	2.47	0.712378
[K].SLHGEFSASDSSLTDIQETR.[R]	1238789350	SIPA1L1	26037	1238789350 [1639-1658]	1xTMT6plex [N-Term];2xPhospho [S/T]	S11(Phospho): 95.23;S12(Phospho): 95.23	10	1.098	0.711861
[R].LTSVSSSVDFDQR.[D]	530382212	TMEM63B	55362	530382212 [118-130]	1xTMT6plex [N-Term];2xPhospho [S3(97.8);S/T]	S3(Phospho): 97.85;S5(Phospho): 50.35;S6(Phospho): 50.35	3	2.237	0.710803
[R].GNVVPSPLPTR.[R]	1034594161	CARHSP1	23589	1034594161 [57-67]	1xPhospho [S6(100)];1xTMT6plex [N-Term]	S6(Phospho): 100	2	0.357	0.710543
[R].SSLGQSASETEEDTVSVSKK.[E]	55749531	SF3B2	10992	55749531 [302-321]	2xPhospho [S/T];3xTMT6plex [N-Term;K19;K20]	S6(Phospho): 65.93;S8(Phospho): 65.93;T10(Phospho): 65.93	1	1.257	0.710360
[R].GPPASSPAPAPK.[F]	1034656800	ZYX	7791	1034656800 [313-324]	1xPhospho [S];2xTMT6plex [N-Term;K12]	S5(Phospho): 50;S6(Phospho): 50	2	1.257	0.710024
[K].AAAAAPASEDEDDEDDEDDEDDDDDEEDDSEEEAMETTPAK.[G]	55956788	NCL	4691	55956788 [177-217]	2xPhospho [S/T];2xTMT6plex [N-Term;K41]	S8(Phospho): 50;S30(Phospho): 50;T37(Phospho): 50;T38(Phospho): 50	4	0.952	0.709798
[R].DHSPTPSVFNSDEER.[Y]	530376841	FIP1L1	81608	530376841 [499-513]	1xPhospho [S3(100)];1xTMT6plex [N-Term]	S3(Phospho): 100	3	0.9	0.709468
[R].IACDEEFSDSEDEGEGGRR.[N]	293336691	HDAC2	3066	293336691 [415-433]	1xCarbamidomethyl [C3];1xTMT6plex [N-Term];2xPhospho [S8(100);S10(100)]	S8(Phospho): 100;S10(Phospho): 100	7	1.246	0.708601
[R].SQDATFSPGSEQAEKSPGPIVSR.[T]	66348045	ZC3HC1	51530	66348045 [329-351]	2xPhospho [S16(87.8);2xTMT6plex [N-Term;K15];T/S]	S7(Phospho): 97.84;S16(Phospho): 99.95	4	0.971	0.708569
[R].DSPLQGSGQQNSQAGQRNSTSSIEPR.[L]	530368861	MAP4K4	9448	530368861 [715-740]	1xPhospho [S/T];1xTMT6plex [N-Term]	S7(Phospho): 16.67;S12(Phospho): 16.67;S19(Phospho): 16.67;T20(Phospho): 16.67;S21(Phospho): 16.67;S22(Phospho): 16.67	10	0.688	0.707428
[R].IACEEEFSDSEEEGEGGRK.[N]	13128860	HDAC1	3065	13128860 [414-432]	1xCarbamidomethyl [C3];2xPhospho [S8(100);2xTMT6plex [N-Term;K19];S10(100)]	S8(Phospho): 100;S10(Phospho): 100	8	1.126	0.707231
[R].SQSSDTEQQSPTSGGGK.[V]	664805999	EIF4B	1975	664805999 [500-516]	2xPhospho [S/T];2xTMT6plex [N-Term;K17]	S4(Phospho): 98.81;T6(Phospho): 98.82	1	0.386	0.707166
[K].IYHLPDAESDEDEDFKEQTR.[L]	1370477773	37500	4735	1370477773 [277-296]	1xPhospho [S9(100)];2xTMT6plex [N-Term;K16]	S9(Phospho): 100	4	0.687	0.706755
[R].KVTSPSSSSSSSSDSESDDEADVSEVTPR.[V]	768020849	NDUFV3	4731	768020849 [147-176]	2xPhospho [S/T];2xTMT6plex [N-Term;K1]	S11(Phospho): 32.51;S12(Phospho): 32.51;S13(Phospho): 32.51;S14(Phospho): 32.51;S16(Phospho): 32.51;S18(Phospho): 32.51	5	1.359	0.705280
[K].NNTAAETEDDESDGEDRGGGTSGVR.[R]	530378760	NIPBL	25836	530378760 [2661-2685]	1xTMT6plex [N-Term];2xPhospho [T7(99.9);S12(100)]	T7(Phospho): 99.93;S12(Phospho): 100	1	1.188	0.703352
[R].NSVLSDPGLDSPRTSPVIMAR.[V]	578801799	ARHGEF11	9826	578801799 [297-317]	1xOxidation [M19];1xTMT6plex [N-Term];2xPhospho [S/T]	S11(Phospho): 66.64;T14(Phospho): 66.64;S15(Phospho): 66.64	2	0.493	0.703308
[R].RPDHSGGSPSPPTSEPAR.[S]	21314720	SNIP1	79753	21314720 [45-62]	1xTMT6plex [N-Term];3xPhospho [S10(98.8);S/T];S14(97.6)	S5(Phospho): 50.62;S8(Phospho): 50.62;S10(Phospho): 98.76;S14(Phospho): 97.55	1	0.854	0.702970
[R].SASEPSLNR.[A]	1034656683	BRAF	673	1034656683 [767-775]	1xPhospho [S];1xTMT6plex [N-Term]	S3(Phospho): 100	2	0.618	0.702475
[R].DYSTLTSVSSHDSR.[L]	578830853	ITGB4	3691	578830853 [1544-1557]	1xTMT6plex [N-Term];2xPhospho [S/T/Y]	S9(Phospho): 96.24;S10(Phospho): 96.24	4	1.355	0.701828
[R].NSATCHSEDSDLEID.[-]	88758594	PDCL	5082	88758594 [287-301]	1xCarbamidomethyl [C5];1xTMT6plex [N-Term];2xPhospho [S7(99.8);S10(99.8)]	S7(Phospho): 100;S10(Phospho): 100	1	0.391	0.701185
[R].LGAGGGSPEKSPSAQELK.[E]	56181387	STUB1	10273	56181387 [13-30]	1xPhospho [S7(98.5)];3xTMT6plex [N-Term;K10;K18]	S7(Phospho): 98.48	3	0.656	0.700563
[R].GNDPLTSSPGR.[S]	33356547	MCM2	4171	33356547 [20-30]	1xTMT6plex [N-Term];2xPhospho [S7(98.2);S8(98.2)]	T6(Phospho): 66.67;S7(Phospho): 66.67;S8(Phospho): 66.67	1	0.442	0.699592
[R].RSLSVEHLETK.[S]	767957960	PTPN3	5774	767957960 [377-387]	2xPhospho [S2(100);2xTMT6plex [N-Term;K11];S4(100)]	S2(Phospho): 100;S4(Phospho): 100	1	0.666	0.699551
[R].SGDETPGSEVPGDK.[A]	118601081	HNRNPUL2	221092	118601081 [161-174]	2xPhospho [S/T];2xTMT6plex [N-Term;K14]	T5(Phospho): 100;S8(Phospho): 100	2	0.904	0.699254
[R].VTQHESDNENEIQIQNK.[L]	530363589	ZMYM4	9202	530363589 [120-136]	1xPhospho [S6(100)];2xTMT6plex [N-Term;K17]	S6(Phospho): 100	5	0.925	0.699178
[R].RYPSSISSSPQK.[D]	530365733	UBAP2L	9898	530365733 [612-623]	2xPhospho [S5(93.7);2xTMT6plex [N-Term;K12];S9(96.8)]	S5(Phospho): 65.91;S7(Phospho): 33.63;S8(Phospho): 33.19;S9(Phospho): 66.36	4	0.428	0.696988
[R].IDEDGENTQIEDTEPMSPVLNSK.[F]	213972636	TP53BP1	7158	213972636 [541-563]	1xOxidation [M16];2xPhospho [S17(100);2xTMT6plex [N-Term;K23];T/S]	T8(Phospho): 50.02;T13(Phospho): 50.02;S17(Phospho): 99.97	1	1.732	0.696889
[K].AVPMAPAPASPGSSNDSSARSQER.[A]	1034608385	MAP1S	55201	1034608385 [750-773]	1xOxidation [M4];1xTMT6plex [N-Term];2xPhospho [S]	S10(Phospho): 94.87;S17(Phospho): 32.79;S18(Phospho): 32.79;S21(Phospho): 32.79	2	0.453	0.696545
[K].AEIHAQPSDKER.[L]	119395742	ITGA6	3655	119395742 [1057-1068]	1xPhospho [S8(100)];2xTMT6plex [N-Term;K10]	S8(Phospho): 100	2	2.716	0.696369
[R].ISSKSPGHMVILDQTK.[G]	767985304	SLTM	79811	767985304 [691-706]	1xOxidation [M9];2xPhospho [S/T];3xTMT6plex [N-Term;K16];K4	S2(Phospho): 66.67;S3(Phospho): 66.67;S5(Phospho): 66.67	4	0.789	0.696349
[K].AVSPPPSPRASPVAALK.[A]	1034657213	TNRC18	84629	1034657213 [989-1005]	2xTMT6plex [N-Term;3xPhospho [S3(100);K17];S11(100)];S7(100)	S3(Phospho): 100;S7(Phospho): 100;S11(Phospho): 100	1	1.315	0.696044
[K].TQTPPVSPAPQPTEERLPSSPVYEDAASFK.[A]	296080746	CTTN	2017	296080746 [362-391]	2xTMT6plex [N-Term;3xPhospho [T/S/Y];K30]	T3(Phospho): 97.19;S7(Phospho): 97.19;S19(Phospho): 49.36;S20(Phospho): 49.36	4	1.553	0.695711
[K].AKPAMPQDSVPSPR.[S]	740086846	ACLY	47	740086846 [524-537]	1xOxidation [M5];1xPhospho [S12(98.5)];2xTMT6plex [N-Term;K2]	S12(Phospho): 98.52	4	0.793	0.694731
[R].EPALPLEPDSGGNTSPGVTANGEAR.[T]	767910855	ARHGEF2	9181	767910855 [825-849]	1xPhospho [S/T];1xTMT6plex [N-Term]	S10(Phospho): 25;T14(Phospho): 25;S15(Phospho): 25;T19(Phospho): 25	9	0.928	0.694674
[R].SLTNSHLEK.[K]	508083059	SLC38A1	81539	508083059 [52-60]	2xPhospho [S1(100);2xTMT6plex [N-Term;K9];S5(100)]	S1(Phospho): 99.97;S5(Phospho): 100	2	0.505	0.694590
[R].SKEGHSLEMENENLVENGADSDEDDNSFLK.[Q]	767974553	LIMA1	51474	767974553 [667-696]	1xOxidation [M9];1xPhospho [S];3xTMT6plex [N-Term;K2;K30]	S21(Phospho): 50;S27(Phospho): 50	2	0.567	0.694478
[R].SAGGSSPEGGEDSDREDGNYCPPVK.[R]	6466466	RANBP3	8498	6466466 [96-120]	1xCarbamidomethyl [C21];2xPhospho [S/Y];2xTMT6plex [N-Term;K25]	S5(Phospho): 88.52;S6(Phospho): 88.52	3	0.638	0.694319
[R].GNIETTSEDGQVFSPK.[K]	530402186	ZC3H13	23091	530402186 [1008-1023]	2xPhospho [S/T];2xTMT6plex [N-Term;K16]	T6(Phospho): 49.56;S7(Phospho): 49.56;S14(Phospho): 99.98	3	1.072	0.694179
[R].IHQDSESGDELSSSSTEQIR.[A]	26986534	ARHGAP12	94134	26986534 [209-228]	1xPhospho [S/T];1xTMT6plex [N-Term]	S5(Phospho): 50;S7(Phospho): 50	4	0.926	0.693839
[R].SSYSSPTSLSPR.[A]	311771772	PDLIM2	64236	311771772 [370-381]	1xTMT6plex [N-Term];2xPhospho [S/T/Y]	S4(Phospho): 48.78;S5(Phospho): 48.78;T7(Phospho): 33.34;S8(Phospho): 33.34;S10(Phospho): 33.34	1	0.4	0.692520
[K].LREQGTESRSSTPLPTISSSAENTR.[Q]	4507555	TMPO	7112	4507555 [149-173]	1xTMT6plex [N-Term];2xPhospho [S/T]	S8(Phospho): 39.89;S10(Phospho): 39.89;S11(Phospho): 59.55;T12(Phospho): 59.55	5	1.141	0.692314
[K].SLKESEQESEEEILAQK.[K]	21396500	HIRIP3	8479	21396500 [219-235]	2xPhospho [S5(98.6);3xTMT6plex [N-Term;K17];K3;S9(100)]	S5(Phospho): 99.99;S9(Phospho): 100	2	0.481	0.691833
[K].HIEDTGSTPSIGENDLK.[F]	550544217	RICTOR	253260	550544217 [1168-1184]	2xPhospho [S7(100);2xTMT6plex [N-Term;K17];S10(98.6)]	S7(Phospho): 98.82;S10(Phospho): 98.83	4	0.57	0.691140
[K].SESPKEPEQLR.[K]	530400152	HNRNPA1	3178	530400152 [4-14]	1xPhospho [S3(100)];2xTMT6plex [N-Term;K5]	S3(Phospho): 99.99	7	1.85	0.689849
[R].TQDPSSPGTTPPQAR.[Q]	1034627629	CHAF1B	8208	1034627629 [424-438]	1xPhospho [S/T];1xTMT6plex [N-Term]	S5(Phospho): 50;S6(Phospho): 50	2	0.729	0.689152
[R].TASFSESR.[A]	740086846	ACLY	47	740086846 [507-514]	1xPhospho [S3(100)];1xTMT6plex [N-Term]	S3(Phospho): 99.99	1	0.208	0.687390
[K].EYPSPPPSPLRR.[G]	767994242	TANC2	26115	767994242 [1612-1623]	1xTMT6plex [N-Term];2xPhospho [S4(98.9);S8(100)]	S4(Phospho): 98.89;S8(Phospho): 100	2	0.905	0.687352
[K].AQSVSPVQAPPPGGSAQLLPGK.[V]	756140883	CIC	23152	756140883 [1809-1830]	1xPhospho [S5(97.6)];2xTMT6plex [N-Term;K22]	S5(Phospho): 97.56	1	1.012	0.686106
[R].IDEDGENTQIEDTEPMSPVLNSK.[F]	213972636	TP53BP1	7158	213972636 [541-563]	1xOxidation [M16];1xPhospho [S17(98.5)];2xTMT6plex [N-Term;K23]	S17(Phospho): 98.53	3	0.773	0.685867
[R].VTNDISPESSPGVGR.[R]	767950881	PCM1	5108	767950881 [60-74]	1xTMT6plex [N-Term];2xPhospho [S10(95.9);S/T]	S9(Phospho): 97.03;S10(Phospho): 97.03	3	0.738	0.683050
[K].NKPGPNIESGNEDDDASFK.[I]	84043963	EIF5B	9669	84043963 [206-224]	1xPhospho [S9(99.9)];3xTMT6plex [N-Term;K19];K2	S9(Phospho): 98.02	1	0.508	0.682391
[R].SESPPPLSDPK.[Q]	578817903	BRD3	8019	578817903 [261-271]	1xPhospho [S];2xTMT6plex [N-Term;K11]	S3(Phospho): 98.75	3	1.443	0.681072
[K].AGIPQHHPPMAQNLQYPDDSDDEK.[K]	42560237	NECTIN1	5818	42560237 [403-426]	1xOxidation [M10];1xPhospho [Y/S];2xTMT6plex [N-Term;K24]	Y16(Phospho): 50;S20(Phospho): 50	1	1.11	0.678676
[R].YHGHSMSDPGVSYR.[T]	291084742	PDHA1	5160	291084742 [327-340]	1xOxidation [M6];1xTMT6plex [N-Term];2xPhospho [S5(100);S12(99)]	S5(Phospho): 99.97;S12(Phospho): 98.3	10	0.944	0.678432
[R].ADLNQGIGEPQSPSRR.[V]	20149675	EFHD2	79180	20149675 [63-78]	1xPhospho [S];1xTMT6plex [N-Term]	S12(Phospho): 50;S14(Phospho): 50	2	1.095	0.677805
[R].GTLDEEDEEADSDTDDIDHR.[V]	1465729081	GPN1	11321	1465729081 [327-346]	1xPhospho [T14(98.6)];1xTMT6plex [N-Term]	T14(Phospho): 98.63	2	0.657	0.677400
[R].GGGSGGGEESEGEEVDED.[-]	15147219	PURB	5814	15147219 [295-312]	1xTMT6plex [N-Term];2xPhospho [S4(100);S10(100)]	S4(Phospho): 100;S10(Phospho): 100	1	0.779	0.677373
[R].SPAGPAATPAQAQAASTPR.[K]	530380665	TCOF1	6949	530380665 [1004-1022]	1xPhospho [S/T];1xTMT6plex [N-Term]	S16(Phospho): 49.45;T17(Phospho): 49.45	2	0.846	0.676453
[K].AAVLSDSEDEEKASAK.[K]	530369278	IWS1	55677	530369278 [399-414]	2xPhospho [S5(100);3xTMT6plex [N-Term;K12;K16];S7(97.5)]	S5(Phospho): 100;S7(Phospho): 100	3	1.247	0.675816
[K].GLNVIGASDQSPLQSPSNLR.[D]	374349211	PLEKHA5	54477	374349211 [1089-1108]	1xTMT6plex [N-Term];2xPhospho [S15(97.8);S]	S8(Phospho): 50.01;S11(Phospho): 50.01;S15(Phospho): 97.83	2	0.987	0.675690
[R].SAGEEEDGPVLTDEQK.[S]	507834119	ARL6IP4	51329	507834119 [332-347]	1xPhospho [S1(100)];2xTMT6plex [N-Term;K16]	S1(Phospho): 100	1	0.564	0.675203
[K].GAHPSGGADDVAK.[K]	19920317	CKAP4	10970	19920317 [22-34]	1xPhospho [S5(100)];2xTMT6plex [N-Term;K13]	S5(Phospho): 100	1	3.912	0.674782
[K].APILIATDVASR.[G]	148613856	DDX17	10521	148613856 [469-480]	1xPhospho [T/S];1xTMT6plex [N-Term]	T7(Phospho): 50;S11(Phospho): 50	2	1.614	0.674675
[R].GEGAGQPSTSAQGQPAAPAPQKR.[G]	4504431	HMGA2	8091	4504431 [5-27]	1xPhospho [S/T];2xTMT6plex [N-Term;K22]	S8(Phospho): 33.33;T9(Phospho): 33.33;S10(Phospho): 33.33	6	2.152	0.674643
[R].KGSDDAPYSPTAR.[V]	530391243	PHF2	5253	530391243 [898-910]	2xPhospho [S3(100);2xTMT6plex [N-Term;K1];Y/S/T]	S3(Phospho): 100;S9(Phospho): 50;T11(Phospho): 50	4	3.215	0.672249
[K].ADVQLFMDDDSYSHHSGLEYADPEK.[F]	4557413	CAV2	858	4557413 [8-32]	1xOxidation [M7];2xPhospho [S/Y];2xTMT6plex [N-Term;K25]	S11(Phospho): 40;Y12(Phospho): 40;S13(Phospho): 40;S16(Phospho): 40;Y20(Phospho): 40	12	0.923	0.671071
[R].TASRPDDIPDSPSSPK.[V]	1034650743	CARMIL1	55604	1034650743 [1288-1303]	2xPhospho [S14(95.8);2xTMT6plex [N-Term;K16];T/S]	S11(Phospho): 50.54;S13(Phospho): 50.54;S14(Phospho): 98.92	2	0.892	0.670621
[K].FADLSEAANR.[N]	578818565	VIM	7431	578818565 [295-304]	1xPhospho [S5(100)];1xTMT6plex [N-Term]	S5(Phospho): 100	1	5.722	0.668322
[R].GGDSIGETPTPGASK.[R]	54112117	SF3B1	23451	54112117 [319-333]	2xPhospho [T8(100);2xTMT6plex [N-Term;K15];T10(100)]	T8(Phospho): 100;T10(Phospho): 100	3	0.928	0.667809
[R].SSGSSGCPSPTPQSSDPDDTPPK.[L]	46852392	CBX6	23466	46852392 [272-294]	1xCarbamidomethyl [C7];1xPhospho [S/T];2xTMT6plex [N-Term;K23]	S1(Phospho): 19.63;S2(Phospho): 19.63;S4(Phospho): 19.63;S5(Phospho): 19.63;S9(Phospho): 19.63	3	1.281	0.667584
[K].SVTPASTLTK.[S]	1034650321	AFDN	4301	1034650321 [1336-1345]	2xPhospho [T3(100);2xTMT6plex [N-Term;K10];S/T]	T3(Phospho): 99.96;T7(Phospho): 97.36	1	0.247	0.667006
[K].HIKEEPLSEEEPCTSTAIASPEK.[K]	7706254	NOP58	51602	7706254 [495-517]	1xCarbamidomethyl [C13];2xPhospho [S20(98.1);3xTMT6plex [N-Term;K23];K3;S/T]	S15(Phospho): 49.19;T16(Phospho): 49.19;S20(Phospho): 98.14	2	0.655	0.666943
[K].STYFSDEEELSD.[-]	51243065	ZCRB1	85437	51243065 [206-217]	1xTMT6plex [N-Term];2xPhospho [S5(99.9);S11(100)]	S5(Phospho): 99.98;S11(Phospho): 100	20	1.056	0.666922
[K].RSSDTSGNEASEIESVK.[I]	767932631	CENPU	79682	767932631 [136-152]	2xPhospho [S/T];2xTMT6plex [N-Term;K17]	S2(Phospho): 40;S3(Phospho): 40;T5(Phospho): 40;S6(Phospho): 40;S11(Phospho): 40	3	1.239	0.666433
[R].IDISPSTLR.[K]	7661958	BCLAF1	9774	7661958 [655-663]	1xPhospho [S/T];1xTMT6plex [N-Term]	S4(Phospho): 33.33;S6(Phospho): 33.33;T7(Phospho): 33.33	2	1.326	0.666166
[R].GPAGEAGASPPVR.[R]	5803177	SNTB2	6645	5803177 [102-114]	1xPhospho [S9(100)];1xTMT6plex [N-Term]	S9(Phospho): 100	4	1.282	0.665617
[R].AAKPGPAEAPSPTASPSGDASPPATAPYDPR.[V]	126723060	ZC3H4	23211	126723060 [1094-1124]	2xPhospho [S/T/Y];2xTMT6plex [N-Term;K3]	S11(Phospho): 33.06;T13(Phospho): 33.06;S15(Phospho): 33.06;S17(Phospho): 45.99;S21(Phospho): 47.38	4	1.613	0.665504
[R].VGSGSLDNLGR.[F]	578821804	PPFIA1	8500	578821804 [689-699]	1xPhospho [S3(98.8)];1xTMT6plex [N-Term]	S3(Phospho): 98.82	1	0.969	0.665415
[R].HQQQLLASPGSSTVDNK.[M]	148886661	XPO4	64328	148886661 [514-530]	1xPhospho [S/T];2xTMT6plex [N-Term;K17]	S8(Phospho): 33.19;S11(Phospho): 33.19;S12(Phospho): 33.19	6	0.729	0.665006
[R].KPGPPLSPEIRSPAGSPELR.[K]	41281612	CHAMP1	283489	41281612 [421-440]	2xPhospho [S7(100);2xTMT6plex [N-Term;K1];S12(99.9)]	S7(Phospho): 100;S12(Phospho): 98.09	2	1.578	0.664530
[R].DSHSSEEDEASSQTDLSQTISK.[K]	767908953	TOR1AIP1	26092	767908953 [153-174]	2xPhospho [S5(98.2);2xTMT6plex [N-Term;K22];S/T]	S4(Phospho): 99.94;S5(Phospho): 99.94	13	0.813	0.663928
[R].SSTDSLPGELR.[G]	1130413426	NCOA5	57727	1130413426 [377-387]	1xTMT6plex [N-Term];2xPhospho [S/T]	S2(Phospho): 66.13;T3(Phospho): 66.13;S5(Phospho): 66.13	2	1.169	0.661949
[R].RTHSDASDDEAFTTSK.[T]	71834863	ARHGAP5	394	71834863 [1170-1185]	2xPhospho [S7(96.7);2xTMT6plex [N-Term;K16];T/S]	S4(Phospho): 98.6;S7(Phospho): 100	2	0.509	0.661457
[R].AHTPTPGIYMGRPTHSGGGGGGGGGGGGGGGR.[R]	9558733;544346329	#N/A	#N/A	9558733 [200-232];544346329 [99-131]	1xOxidation [M10];1xPhospho [T3(79.1)];1xTMT6plex [N-Term]	T3(Phospho): 77.53	1	1.021	0.661357
[R].TPRTPRTPQLK.[D]	530380464	LARP1	23367	530380464 [782-792]	2xPhospho [T4(100);2xTMT6plex [N-Term;K11];T7(100)]	T4(Phospho): 100;T7(Phospho): 100	4	0.238	0.661222
[K].GLNPDGTPALSTLGGFSPASKPSSPR.[E]	9945320	CCNL1	57018	9945320 [319-344]	2xPhospho [S20(96.1);2xTMT6plex [N-Term;K21];S/T]	S17(Phospho): 98.15;S20(Phospho): 96.37	9	0.812	0.660534
[K].ISYIPDEEVSSPSPPQR.[A]	631226390	IGF2BP2	10644	631226390 [158-174]	1xPhospho [S/Y];1xTMT6plex [N-Term]	S11(Phospho): 98.89	7	0.853	0.660313
[R].LRSADSENALSVQER.[N]	42794767	MAP3K3	4215	42794767 [366-380]	1xTMT6plex [N-Term];2xPhospho [S3(100);S6(100)]	S3(Phospho): 100;S6(Phospho): 100	1	1.469	0.659651
[K].YADSPGASSPEQPK.[R]	27363484	ELF1	1997	27363484 [160-173]	2xPhospho [S4(98.5);2xTMT6plex [N-Term;K14];S9(98.5)]	S4(Phospho): 99.98;S8(Phospho): 50;S9(Phospho): 50	6	1.024	0.659425
[R].LQSETSV.[-]	20373171	VANGL1	81839	20373171 [518-524]	1xTMT6plex [N-Term];2xPhospho [S3(98.6);S6(98.5)]	S3(Phospho): 98.47;S6(Phospho): 98.47	1	1.497	0.657792
[R].DKAPVQPQQSPAAAPGGTDEKPSGK.[E]	25777602	PSMD2	5708	25777602 [7-31]	1xPhospho [S10(96.5)];4xTMT6plex [N-Term;K2;K21;K25]	S10(Phospho): 100	4	1.084	0.657542
[R].TVAISDAAQLPHDYCTTPGGTLFSTTPGGTR.[I]	4758260	EIF4EBP2	1979	4758260 [21-51]	1xCarbamidomethyl [C15];1xTMT6plex [N-Term];2xPhospho [Y/T/S]	T25(Phospho): 57.94;T26(Phospho): 57.94;T30(Phospho): 57.94	9	0.278	0.657424
[R].ADYDTLSLRSLR.[L]	732663005	PKP3	11187	732663005 [189-200]	1xTMT6plex [N-Term];2xPhospho [T/S/Y]	T5(Phospho): 66.67;S7(Phospho): 66.67;S10(Phospho): 66.67	2	0.919	0.657307
[R].KTSAVSSPLLDQQR.[N]	530402186	ZC3H13	23091	530402186 [236-249]	2xPhospho [T/S];2xTMT6plex [N-Term;K1]	T2(Phospho): 50;S3(Phospho): 50;S6(Phospho): 50;S7(Phospho): 50	4	2.648	0.657272
[R].VMTIPYQPMPASSPVICAGGQDR.[C]	222352151	PCBP1	5093	222352151 [178-200]	1xCarbamidomethyl [C17];1xPhospho [Y/S/T];1xTMT6plex [N-Term];2xOxidation [M2;M9]	Y6(Phospho): 33.31;S12(Phospho): 33.31;S13(Phospho): 33.31	3	0.68	0.656767
[K].SLPQAVGQGSSSPPGGK.[H]	124430752	KIF26B	55083	124430752 [1762-1778]	1xPhospho [S];2xTMT6plex [N-Term;K17]	S12(Phospho): 98.42	5	0.902	0.656413
[R].LTPSPDIIVLSDNEASSPR.[S]	530365128	GATAD2B	57459	530365128 [119-137]	1xTMT6plex [N-Term];2xPhospho [S11(100);S16(98.2)]	S11(Phospho): 99.96;S16(Phospho): 98.19	8	1.174	0.653937
[R].LDSQPQETSPELPR.[R]	219879806	TRAFD1	10906	219879806 [407-420]	1xPhospho [T/S];1xTMT6plex [N-Term]	T8(Phospho): 50;S9(Phospho): 50	6	0.474	0.652606
[R].VQEKPDSPGGSTQIQR.[Y]	33356170	MYO9B	4650	33356170 [1284-1299]	1xPhospho [S7(100)];2xTMT6plex [N-Term;K4]	S7(Phospho): 99.96	3	0.501	0.651666
[R].SSSPTQYGLTK.[N]	530370525	MAP3K20	51776	530370525 [635-645]	1xPhospho [S2(97.8)];2xTMT6plex [N-Term;K11]	S2(Phospho): 97.85	2	0.707	0.650470
[K].FPSPHPSPAK.[L]	767970787	EI24	9538	767970787 [324-333]	2xPhospho [S3(100);2xTMT6plex [N-Term;K10];S7(100)]	S3(Phospho): 100;S7(Phospho): 100	1	1.129	0.650120
[R].ASPAPGSGHPEGPGAHLDMNSLDR.[A]	54607135	TOMM70	9868	54607135 [90-113]	1xOxidation [M19];1xPhospho [S2(100)];1xTMT6plex [N-Term]	S2(Phospho): 100	1	0.829	0.647763
[R].KAEDSDSEPEPEDNVR.[L]	964750564	XRN2	22803	964750564 [573-588]	2xPhospho [S5(100);2xTMT6plex [N-Term;K1];S7(100)]	S5(Phospho): 100;S7(Phospho): 100	19	1.052	0.647416
[K].EIQNGNLHESDSESVPR.[D]	118498356	KTN1	3895	118498356 [66-82]	1xPhospho [S10(99.9)];1xTMT6plex [N-Term]	S10(Phospho): 99.89	1	0.55	0.643908
[R].RSSTSGDTEEEEEEEVVPFSSDEQK.[R]	1034675221	ZNF185	7739	1034675221 [151-175]	2xPhospho [S2(87.2);2xTMT6plex [N-Term;K25];S3(87.2)]	S2(Phospho): 63;S3(Phospho): 63;T4(Phospho): 63	1	0.55	0.643576
[K].IDGATQSSPAEPK.[S]	212276096	LRRFIP1	9208	212276096 [707-719]	1xPhospho [T/S];2xTMT6plex [N-Term;K13]	T5(Phospho): 33.33;S7(Phospho): 33.33;S8(Phospho): 33.33	4	0.646	0.642635
[R].ASATSTSKPNSPR.[V]	120587027	USP31	57478	120587027 [1168-1180]	2xPhospho [S/T];2xTMT6plex [N-Term;K8]	T6(Phospho): 94.26;S7(Phospho): 94.26	3	0.49	0.641778
[K].RPNEDSDEDEEK.[G]	4557495	CSTF3	1479	4557495 [686-697]	1xPhospho [S6(100)];2xTMT6plex [N-Term;K12]	S6(Phospho): 100	1	0.842	0.641258
[K].KYPSPQK.[T]	530380592	RBM27	54439	530380592 [117-123]	1xPhospho [S4(100)];3xTMT6plex [N-Term;K1;K7]	S4(Phospho): 100	1	1.273	0.641060
[R].KPISDNSFSSDEEQSTGPIK.[Y]	87116683	ZFC3H1	196441	87116683 [1295-1314]	2xPhospho [S7(97.4);3xTMT6plex [N-Term;K1;K20];S/T]	S9(Phospho): 99.85;S10(Phospho): 99.85	9	0.952	0.640035
[R].SNSFNNPLGNR.[A]	1034652229	LATS1	9113	1034652229 [462-472]	1xPhospho [S3(100)];1xTMT6plex [N-Term]	S3(Phospho): 100	1	0.417	0.639314
[K].KAEQGSEEEGEGEEEEEEGGESK.[A]	69354671	ABCF1	23	69354671 [223-245]	1xPhospho [S6(100)];3xTMT6plex [N-Term;K1;K23]	S6(Phospho): 100	1	0.512	0.639232
[R].NSSSPVSPASVPGQR.[R]	33457336	IRF2BPL	64207	33457336 [656-670]	1xTMT6plex [N-Term];2xPhospho [S7(100);S]	S4(Phospho): 98.43;S7(Phospho): 50;S10(Phospho): 50	3	0.957	0.639007
[R].TKDQPDGSSLSPAQSPSQSQPPAASSLR.[E]	1417435944	MPRIP	23164	1417435944 [242-269]	2xPhospho [S/T];2xTMT6plex [N-Term;K2]	S11(Phospho): 49.91;S15(Phospho): 49.91;S17(Phospho): 49.91;S19(Phospho): 49.91	4	0.996	0.638761
[K].TPSPKEEDEEPESPPEKK.[T]	56118310	NUCKS1	64710	56118310 [202-219]	1xPhospho [S13(100)];4xTMT6plex [N-Term;K17;K18];K5	S13(Phospho): 99.96	1	0.402	0.638043
[R].KSLSDSESDDSK.[S]	530384472	CBX3	11335	530384472 [92-103]	2xPhospho [S];3xTMT6plex [N-Term;K1;K12]	S2(Phospho): 100;S4(Phospho): 99.98	2	0.536	0.637636
[R].RIDISPSTFR.[K]	167234419	THRAP3	9967	167234419 [678-687]	1xPhospho [S5(95)];1xTMT6plex [N-Term]	S5(Phospho): 100	1	1.117	0.637490
[R].GGQGDPAVPAQQPADPSTPERQSSPSGSEQLVR.[R]	1034675221	ZNF185	7739	1034675221 [463-495]	1xTMT6plex [N-Term];2xPhospho [S/T]	S17(Phospho): 49.91;T18(Phospho): 49.91;S23(Phospho): 93.42	6	0.794	0.637399
[K].ASLVALPEQTASEEETPPPLLTK.[E]	7662238	ACIN1	22985	7662238 [399-421]	2xPhospho [T/S];2xTMT6plex [N-Term;K23]	T10(Phospho): 66.67;S12(Phospho): 66.67;T16(Phospho): 66.67	2	2.267	0.637247
[R].GTAQYSSQK.[S]	148664226	PKP2	5318	148664226 [126-134]	1xPhospho [Y/S/T];2xTMT6plex [N-Term;K9]	S7(Phospho): 99.27	2	0.535	0.636184
[R].SDSRAQAVSEDAGGNEGR.[A]	767945997	EIF3B	8662	767945997 [117-134]	1xPhospho [S3(100)];1xTMT6plex [N-Term]	S3(Phospho): 98.34	4	0.456	0.634596
[K].IEDVGSDEEDDSGKDKK.[K]	1370507998	HSP90AB1	3326	1370507998 [250-266]	2xPhospho [S6(100);4xTMT6plex [N-Term;K14;K16;K17];S12(100)]	S6(Phospho): 100;S12(Phospho): 100	1	0.238	0.634305
[K].DTSATSQSVNGSPQAEQPSLESTSK.[E]	66348045	ZC3HC1	51530	66348045 [51-75]	1xPhospho [S12(92)];2xTMT6plex [N-Term;K25]	S12(Phospho): 91.99	3	1.261	0.633298
[R].VVVISSSEDSDAENSSSR.[E]	67089149	PML	5371	67089149 [556-573]	1xTMT6plex [N-Term];2xPhospho [S]	S5(Phospho): 32.66;S6(Phospho): 32.66;S7(Phospho): 32.66;S16(Phospho): 48.45;S17(Phospho): 48.45	4	0.696	0.632617
[R].APSDSSLGTPSDGRPELR.[G]	578833732	TRIP10	9322	578833732 [294-311]	1xPhospho [S3(96.2)];1xTMT6plex [N-Term]	S3(Phospho): 96.22	2	0.412	0.631828
[R].TSSGTSLSAMHSSGSSGK.[G]	213972636	TP53BP1	7158	213972636 [1320-1337]	1xOxidation [M10];2xPhospho [T/S];2xTMT6plex [N-Term;K18]	T1(Phospho): 33.32;S2(Phospho): 33.32;S3(Phospho): 33.32;T5(Phospho): 33.32;S6(Phospho): 33.32;S8(Phospho): 33.32	4	2.045	0.631656
[R].RNSSEASSGDFLDLK.[GK]	50345294	JPT1	51155	50345294 [85-99]	1xPhospho [S3(93.2)];2xTMT6plex [N-Term;K15]	S3(Phospho): 25;S4(Phospho): 25;S7(Phospho): 25;S8(Phospho): 25	1	0.201	0.631460
[K].VVSQSQPGSR.[S]	1034612655	CLASP1	23332	1034612655 [683-692]	1xTMT6plex [N-Term];2xPhospho [S9(99);S]	S3(Phospho): 50.01;S5(Phospho): 50.01;S9(Phospho): 99.98	1	1.071	0.630135

Patil et al,2020. Proteomic and phosphoproteomic profiling of shammah-induced signaling in oral keratinocytes

Supplementary Table S2: List of phosphopeptides identified and quantified in OKF6/TERT1-Shammah and OKF6/TERT1-Parental phosphoproteome using TMT/TiO$_2$-based quantitative phosphoproteomic approach

Peptide Sequence	Protein Accessions	Gene Symbol	Gene ID	Positions in Proteins	Modifications	PhosphoRS: Best Site Probabilities	# PSMs	Fold change	p-value
[R].NASVPNLR.[G]	153251766	MTFR1L	56181	153251766 [101-108]	1xPhospho [S3(100)];1xTMT6plex [N-Term]	S3(Phospho): 100	2	2.611	0.628581
[K].KAPAGQEEPGTPPSSPLSAEQLDR.[I]	19718751	UNG	7374	19718751 [50-73]	2xPhospho [T/S];2xTMT6plex [N-Term;K1]	T11(Phospho): 98.46;S14(Phospho): 50;S15(Phospho): 50	2	0.715	0.626904
[R].EVEEELATSGGQSPTGEQIPQFQQR.[Q]	530362268	MACF1	23499	530362268 [5849-5873]	1xPhospho [T/S];1xTMT6plex [N-Term]	T8(Phospho): 25;S9(Phospho): 25;S13(Phospho): 25;T15(Phospho): 25	4	0.955	0.625658
[R].GLVAAYSGESDSEEEQER.[G]	1370525026	RBM10	8241	1370525026 [826-843]	1xTMT6plex [N-Term];2xPhospho [S/Y]	S10(Phospho): 100;S12(Phospho): 100	26	1.252	0.625640
[K].GLSLSPQSPPERR.[G]	118421085	TICRR	90381	118421085 [1426-1438]	1xTMT6plex [N-Term];2xPhospho [S5(98.6);S8(100)]	S5(Phospho): 98.62;S8(Phospho): 100	3	1.097	0.624060
[R].GLVAAYSGDSDNEEELVER.[L]	1034630608	RBM5	10181	1034630608 [615-633]	1xPhospho [Y/S];1xTMT6plex [N-Term]	Y6(Phospho): 33.33;S7(Phospho): 33.33;S10(Phospho): 33.33	6	1.073	0.623944
[R].SMVSPVPSPTGTISVPNSCPASPR.[G]	82546824	FOXK1	221937	82546824 [236-259]	1xCarbamidomethyl [C19];1xOxidation [M2];1xTMT6plex [N-Term];2xPhospho [S/T]	S14(Phospho): 87.67;S18(Phospho): 50.6;S22(Phospho): 50.6	1	0.719	0.623436
[R].ARHDTPDPSPLR.[G]	14249338	BUD13	84811	14249338 [155-166]	1xTMT6plex [N-Term];2xPhospho [T5(100);S9(100)]	T5(Phospho): 100;S9(Phospho): 100	3	1.315	0.623435
[K].SNTEPQSPPIASPK.[A]	1394533392	TMF1	7110	1394533392 [66-79]	1xPhospho [S12(98.2)];2xTMT6plex [N-Term;K14]	S12(Phospho): 98.21	1	0.791	0.623130
[K].TNQAILSQLYQSPNR.[R]	1034664209	ELAVL2	1993	1034664209 [242-256]	1xPhospho [S12(97.9)];1xTMT6plex [N-Term]	S12(Phospho): 97.89	4	0.409	0.622364
[K].ESAAPASPAPSPAPSPTPAPPQK.[E]	78042577	MAP7D1	55700	78042577 [538-560]	2xTMT6plex [N-Term;3xPhospho [S/T];K23]	S7(Phospho): 73.42;S11(Phospho): 73.42;S15(Phospho): 73.42;T17(Phospho): 73.42	4	0.615	0.621853
[K].DTSATSQSVNGSPQAEQPSLESTSK.[E]	66348045	ZC3HC1	51530	66348045 [51-75]	2xPhospho [S/T];2xTMT6plex [N-Term;K25]	S8(Phospho): 81.37;S12(Phospho): 91.94	1	1.203	0.621370
[R].TGSNISGASSDISLDEQYK.[H]	4505531	OSBP	5007	4505531 [377-395]	2xPhospho [T/S/Y];2xTMT6plex [N-Term;K19]	T1(Phospho): 59.83;S3(Phospho): 59.83;S6(Phospho): 59.83	1	0.331	0.620246
[R].SFSISPVR.[L]	520261969	SON	6651	520261969 [2009-2016]	1xTMT6plex [N-Term];2xPhospho [S1(100);S3(98.5)]	S1(Phospho): 100;S3(Phospho): 98.25	1	2.072	0.619180
[K].APAPQPPSLPDRSPRPQR.[H]	578810794	SYNPO	11346	578810794 [513-530]	1xTMT6plex [N-Term];2xPhospho [S8(100);S13(100)]	S8(Phospho): 100;S13(Phospho): 100	1	1.082	0.618024
[K].YTPTSPSYSPSSPEYTPTSPK.[Y]	4505939	POLR2A	5430	4505939 [1839-1859]	2xPhospho [T/S/Y];2xTMT6plex [N-Term;K21]	T4(Phospho): 48.56;S5(Phospho): 48.56;S7(Phospho): 17.06;Y8(Phospho): 17.06;S9(Phospho): 17.06;S11(Phospho): 17.06;S12(Phospho): 17.06;Y15(Phospho): 17.06	3	0.801	0.617625
[R].VIGQDHDFSESSEEEAPAEASSGALR.[S]	122939208	SP100	6672	122939208 [399-424]	1xTMT6plex [N-Term];2xPhospho [S9(100);S]	S9(Phospho): 98.62;S11(Phospho): 50.69;S12(Phospho): 50.69	7	1.187	0.616331
[R].DLPAQRGEESSEEEEK.[G]	21396500	HIRIP3	8479	21396500 [150-165]	2xPhospho [S10(100);2xTMT6plex [N-Term;K16];S11(100)]	S10(Phospho): 100;S11(Phospho): 100	2	0.502	0.616264
[R].VYSLPDGTFSSDEDEEEEEEEEEEEEEEET.[-]	4503523	EIF3D	8664	4503523 [519-548]	1xTMT6plex [N-Term];2xPhospho [T30(90.5);T/S/Y]	S10(Phospho): 96.16;S11(Phospho): 96.16	32	0.699	0.615212
[R].SASASHQADIK.[E]	767912421	LBR	3930	767912421 [97-107]	1xPhospho [S];2xTMT6plex [N-Term;K11]	S3(Phospho): 98.91	3	0.933	0.614893
[K].AVGMPSPVSPK.[L]	530379849	AFF4	27125	530379849 [1050-1060]	1xOxidation [M4];2xPhospho [S6(100);2xTMT6plex [N-Term;K11];S9(100)]	S6(Phospho): 100;S9(Phospho): 100	5	1.203	0.614650
[R].AGGGGGLGAGSPALSGGQGR.[R]	106507164	RAB12	201475	106507164 [11-30]	1xPhospho [S11(100)];1xTMT6plex [N-Term]	S11(Phospho): 100	6	0.921	0.614376
[R].KTSSVSSISQVSPER.[G]	348041302	PI4KA	5297	348041302 [254-268]	2xPhospho [S6(96.3);2xTMT6plex [N-Term;K1];T/S]	T2(Phospho): 95.33;S6(Phospho): 49.98;S7(Phospho): 49.98	3	2.963	0.612810
[R].SRSPLELEPEAK.[K]	4557515	DDB2	1643	4557515 [24-35]	1xPhospho [S];2xTMT6plex [N-Term;K12]	S1(Phospho): 50;S3(Phospho): 50	2	2.299	0.611558
[K].NENTEGSPQEDGVELEGLK.[Q]	19913406	TOP2A	7153	19913406 [1241-1259]	1xPhospho [S7(100)];2xTMT6plex [N-Term;K19]	S7(Phospho): 99.96	1	1.114	0.611099
[R].VASPSQGQVGSSSPK.[R]	767920201	PKP4	8502	767920201 [325-339]	1xPhospho [S5(94.6)];2xTMT6plex [N-Term;K15]	S13(Phospho): 99.09	8	0.902	0.610389
[K].DFQDYMEPEEGCQGSPQR.[R]	5453634	DYNC1LI2	1783	5453634 [180-197]	1xCarbamidomethyl [C12];1xOxidation [M6];1xPhospho [S15(100)];1xTMT6plex [N-Term]	S15(Phospho): 100	2	0.485	0.610206
[K].SREDLSAQPVQTK.[F]	1034591669	TJP1	7082	1034591669 [710-722]	1xPhospho [S1(100)];2xTMT6plex [N-Term;K13]	S1(Phospho): 99.99	2	0.598	0.609564
[R].AAEESASQIQSSAQR.[L]	530366188	LAMB3	3914	530366188 [855-869]	1xPhospho [S];1xTMT6plex [N-Term]	S11(Phospho): 50;S12(Phospho): 50	4	2.075	0.609095
[R].IGDEYAEDSSDEEDIR.[N]	21327667	BOP1	23246	21327667 [118-133]	1xTMT6plex [N-Term];2xPhospho [S/Y]	S9(Phospho): 100;S10(Phospho): 100	10	0.807	0.609020
[K].VVEAVNSDSDSEFGIPK.[K]	1061214220	TOP2B	7155	1061214220 [1516-1532]	2xPhospho [S];2xTMT6plex [N-Term;K17]	S7(Phospho): 99.26;S9(Phospho): 50.37;S11(Phospho): 50.37	12	1.099	0.607511
[K].NPDEIDETTELSS.[-]	116295258	ITGA2	3673	116295258 [1169-1181]	1xPhospho [S/T];1xTMT6plex [N-Term]	S12(Phospho): 49.98;S13(Phospho): 49.98	4	1.081	0.607430
[R].RGEGDAPFSEPGTTSTQRPSSPETATK.[Q]	118572613	SRRM2	23524	118572613 [303-329]	1xPhospho [T/S];2xTMT6plex [N-Term;K27]	S21(Phospho): 98.14	10	1.413	0.607008
[K].KVQIPVSRPDPEPVSDNEEDSYDEEIHDPR.[S]	1034591669	TJP1	7082	1034591669 [204-233]	2xPhospho [S/Y];2xTMT6plex [N-Term;K1]	S15(Phospho): 98.01;S21(Phospho): 50.99;Y22(Phospho): 50.99	3	0.483	0.606680
[K].SRSPIIHSPK.[R]	1075637959	PCF11	51585	1075637959 [487-496]	2xPhospho [S3(99.2);2xTMT6plex [N-Term;K10];S8(100)]	S3(Phospho): 99.99;S8(Phospho): 100	6	1.036	0.606637
[R].SASVSPTRNSSNTLPIPK.[K]	119829187	COL17A1	1308	119829187 [170-187]	2xTMT6plex [N-Term;3xPhospho [S3(96.4);K18];S5(96.3);T/S]	S3(Phospho): 94.24;S5(Phospho): 94.05;T7(Phospho): 34.66;S10(Phospho): 34.66;S11(Phospho): 34.66	2	0.836	0.606245
[K].AAVLSDSEDEEK.[A]	530369278	IWS1	55677	530369278 [399-410]	2xPhospho [S5(100);2xTMT6plex [N-Term;K12];S7(100)]	S5(Phospho): 100;S7(Phospho): 100	3	0.581	0.606081
[R].RREEGPPPPSPDGASSDAEPEPPSGR.[T]	767925441	SMC4	10051	767925441 [13-38]	1xTMT6plex [N-Term];2xPhospho [S10(100);S]	S10(Phospho): 99.89;S15(Phospho): 50.05;S16(Phospho): 50.05	2	0.664	0.605651
[K].FHSSDSEEEEHKK.[Q]	530369278	IWS1	55677	530369278 [365-377]	2xPhospho [S6(99.4);3xTMT6plex [N-Term;K12;K13];S]	S3(Phospho): 50.32;S4(Phospho): 50.32;S6(Phospho): 99.35	1	0.415	0.604881
[R].AASPPASASDLIEQQQK.[R]	56790935	STRIP1	85369	56790935 [333-349]	1xPhospho [S3(96.4)];2xTMT6plex [N-Term;K17]	S3(Phospho): 96.23	2	0.793	0.603860
[R].SCTPSPDQISHR.[A]	1407380404	ZC3HAV1	56829	1407380404 [271-282]	1xCarbamidomethyl [C2];1xTMT6plex [N-Term];2xPhospho [S5(96.6);T/S]	T3(Phospho): 99.11;S5(Phospho): 100	3	0.929	0.602406
[R].RSSQPSPTAVPASDSPPTKQEVK.[K]	78042577	MAP7D1	55700	78042577 [111-133]	2xPhospho [S6(99.9);3xTMT6plex [N-Term;K19;K23];S15(99.9)]	S6(Phospho): 99.86;S15(Phospho): 99.9	2	0.417	0.601544
[R].TSFTSVSR.[S]	119395754	KRT5	3852	119395754 [30-37]	1xPhospho [S7(100)];1xTMT6plex [N-Term]	S7(Phospho): 100	3	1.103	0.600822
[K].KPATPAEDDEDDDIDLFGSDNEEEDKEAAQLREER.[L]	1370511980	EEF1D	1936	1370511980 [510-544]	1xPhospho [S19(100)];3xTMT6plex [N-Term;K1;K26]	S19(Phospho): 100	3	0.393	0.600554
[K].KGDVEGSQSQDEGEGSGESER.[G]	4885399	SMC3	9126	4885399 [1059-1079]	1xPhospho [S];2xTMT6plex [N-Term;K1]	S9(Phospho): 100	5	1.371	0.600446
[R].GHTDTEGRPPSPPPTSTPEK.[C]	530389521	HSF1	3297	530389521 [353-372]	2xPhospho [S16(90.6);2xTMT6plex [N-Term;K20];T17(96.1)]	S16(Phospho): 90.55;T17(Phospho): 96.07	3	0.5	0.599027
[K].ALDISLSSGEEDEGDEEDSTAGTTK.[Q]	194097365	FTSJ3	117246	194097365 [329-353]	2xPhospho [S/T];2xTMT6plex [N-Term;K25]	S7(Phospho): 65.88;S8(Phospho): 65.88;S19(Phospho): 65.88	8	0.88	0.597636
[R].ENPPVEDSSDEDDKRNQGNLYDK.[A]	30578410	STT3B	201595	30578410 [491-513]	2xPhospho [S8(99.9);3xTMT6plex [N-Term;K14;K23];S9(99.9)]	S8(Phospho): 99.84;S9(Phospho): 99.84	3	2.431	0.597220
[R].GGSISVQVNSIKFDSE.[-]	530394600	NOLC1	9221	530394600 [695-710]	1xPhospho [S];2xTMT6plex [N-Term;K12]	S15(Phospho): 99.95	1	1.117	0.596983
[R].ASAPSPNAQVACDHCLK.[E]	68160937	TRIM25	7706	68160937 [96-112]	1xPhospho [S5(96.2)];2xCarbamidomethyl [C12;2xTMT6plex [N-Term;C15];K17]	S5(Phospho): 100	1	0.696	0.596418
[K].MSSPPSSPQK.[C]	118918395	FNDC3A	22862	118918395 [201-210]	1xOxidation [M1];1xPhospho [S];2xTMT6plex [N-Term;K10]	S2(Phospho): 49.99;S3(Phospho): 49.99	2	0.962	0.596026
[K].APASPGAGNPPGTPK.[G]	530380053	ZNF608	57507	530380053 [624-638]	2xPhospho [S4(100);2xTMT6plex [N-Term;K15];T13(100)]	S4(Phospho): 100;T13(Phospho): 100	3	0.8	0.596019
[R].TAPATGQLPGRSSPAGSPR.[T]	191252782	PAK6	56924	191252782 [335-353]	1xTMT6plex [N-Term];2xPhospho [T5(97.4);S17(100)]	S13(Phospho): 97.37;S17(Phospho): 100	3	0.954	0.595463
[R].AHSPASTLPNSPGSTFERK.[T]	119829187	COL17A1	1308	119829187 [83-101]	2xPhospho [S/T];2xTMT6plex [N-Term;K19]	S11(Phospho): 99.93;T15(Phospho): 98.59	11	1.199	0.595322
[K].REVLYDSEGLSGEER.[G]	768010386	SCAF1	58506	768010386 [731-745]	1xTMT6plex [N-Term];2xPhospho [S7(100);S11(100)]	S7(Phospho): 100;S11(Phospho): 100	3	0.976	0.594287
[R].FPSGNQGGAGPSQGSGGGTGGSVYTEDNDDDLYG.[-]	6005942	VCP	7415	6005942 [773-806]	1xPhospho [S/T/Y];1xTMT6plex [N-Term]	S12(Phospho): 24.65;S15(Phospho): 24.65;T19(Phospho): 24.65;S22(Phospho): 24.65	24	2.436	0.593749
[K].VGSLDNVGHLPAGGAVK.[TI]	1370484102	MAP4	4134	1370484102 [2245-2261]	1xPhospho [S3(100)];2xTMT6plex [N-Term;K17]	S3(Phospho): 100	1	0.48	0.593195
[K].KDDSHSAEDSEDEKEDHK.[N]	56118310	NUCKS1	64710	56118310 [70-87]	2xPhospho [S];4xTMT6plex [N-Term;K1;K14;K18]	S6(Phospho): 98.27;S10(Phospho): 100	2	0.148	0.592455
[K].APAGQEEPGTPPSSPLSAEQLDR.[I]	19718751	UNG	7374	19718751 [51-73]	1xTMT6plex [N-Term];2xPhospho [S13(92.9);S14(92.9)]	S13(Phospho): 92.94;S14(Phospho): 92.94	1	0.704	0.591613
[R].ALSGRASPVPAPSSGLHAAVR.[L]	112734870	KIAA1522	57648	112734870 [915-935]	1xTMT6plex [N-Term];2xPhospho [S3(100);S7(100)]	S3(Phospho): 100;S7(Phospho): 99.99	1	0.705	0.591537
[R].ASVRGSSEEDEDAR.[T]	31044432	LEMD2	221496	31044432 [133-146]	1xTMT6plex [N-Term];2xPhospho [S6(100);S7(100)]	S6(Phospho): 100;S7(Phospho): 100	3	0.728	0.589500
[K].GLPSPYNMSSAPGSR.[S]	58530840	DSP	1832	58530840 [2812-2826]	1xOxidation [M8];1xPhospho [Y/S];1xTMT6plex [N-Term]	S14(Phospho): 92.24	1	0.456	0.589424
[K].IVRGDQPAASGDSDDDEPPPLPR.[L]	5729875	PGRMC1	10857	5729875 [45-67]	1xPhospho [S];1xTMT6plex [N-Term]	S10(Phospho): 50;S13(Phospho): 50	6	1.041	0.589347
[K].IEDVGSDEEDDSGKDK.[K]	1370507998	HSP90AB1	3326	1370507998 [250-265]	1xPhospho [S12(98.2)];3xTMT6plex [N-Term;K14;K16]	S6(Phospho): 100	1	0.262	0.589253
[R].AVSEGCASEDEVEGEA.[-]	389565509	TBC1D2	55357	389565509 [913-928]	1xCarbamidomethyl [C6];1xTMT6plex [N-Term];2xPhospho [S3(100);S8(100)]	S3(Phospho): 100;S8(Phospho): 100	1	1.209	0.588871
[K].DDSHSAEDSEDEKEDHK.[N]	56118310	NUCKS1	64710	56118310 [71-87]	1xPhospho [S];3xTMT6plex [N-Term;K13;K17]	S5(Phospho): 97.83	1	0.468	0.587464
[K].GPGEPDSPTPLHPPTPPILSTDR.[S]	767970167	KMT2A	4297	767970167 [1867-1889]	1xTMT6plex [N-Term];2xPhospho [S7(97.3);T15(99.9)]	S7(Phospho): 97.27;T15(Phospho): 99.91	2	1.28	0.587260
[R].SRSHTSEGAHLDITPNSGAAGNSAGPK.[S]	767952381	NDRG1	10397	767952381 [379-405]	2xPhospho [S1(96.8);2xTMT6plex [N-Term;K27];S3(99.9)]	S1(Phospho): 96.81;S3(Phospho): 99.89	6	0.287	0.586965
[K].GAGDGSDEEVDGK.[A]	1034628998	MYH9	4627	1034628998 [1938-1950]	1xPhospho [S6(100)];2xTMT6plex [N-Term;K13]	S6(Phospho): 100	1	0.212	0.586567
[K].SPPREGSQGELTPANSQSR.[M]	1370470744	GPS1	2873	1370470744 [594-612]	1xPhospho [S7(100)];1xTMT6plex [N-Term]	S7(Phospho): 99.98	4	1.21	0.585585
[R].FLETDSEEEQEEVNEK.[K]	578817611	NOL8	55035	578817611 [885-900]	2xPhospho [T4(100);2xTMT6plex [N-Term;K16];S6(100)]	T4(Phospho): 100;S6(Phospho): 100	4	1.233	0.585446
[R].DIVVEPMGGYMYWTDWGASPK.[I]	157384998	LRP4	4038	157384998 [863-883]	1xOxidation [M11];2xPhospho [Y10(100);2xTMT6plex [N-Term;K21];S19(91)]	Y10(Phospho): 99.98;Y12(Phospho): 93.39	1	0.276	0.585272
[R].SAGGGAGPGGAAGGGVGGGDEPGSPAQGK.[R]	4507741	TWIST1	7291	4507741 [45-73]	1xPhospho [S24(95.9)];2xTMT6plex [N-Term;K29]	S24(Phospho): 95.87	1	2.353	0.585052
[K].KMSQPGSPSPK.[T]	1370461044	CHD4	1108	1370461044 [1557-1567]	1xOxidation [M2];2xPhospho [S3(100);3xTMT6plex [N-Term;K1;K11];S7(100)]	S3(Phospho): 100;S7(Phospho): 99.95	3	0.839	0.577429
[R].AGEPNSPDAEEANSPDVTAGCDPAGVHPPR.[-]	39995082	NSUN2	54888	39995082 [738-767]	1xCarbamidomethyl [C21];1xTMT6plex [N-Term];2xPhospho [S6(100);S14(97.7)]	S6(Phospho): 100;S14(Phospho): 97.74	8	1.103	0.576690
[R].TPAAAAAMNLASPR.[T]	118572613	SRRM2	23524	118572613 [2261-2274]	1xOxidation [M8];1xPhospho [S12(100)];1xTMT6plex [N-Term]	S12(Phospho): 100	3	1.159	0.575908
[R].DGGRSSPGGQDEGGFMAQGK.[T]	1034580879	PXN	5829	1034580879 [794-813]	1xOxidation [M16];1xPhospho [S];2xTMT6plex [N-Term;K20]	S5(Phospho): 50;S6(Phospho): 50	6	0.431	0.575813
[R].EVMESAVGNSSGSGQNEESPR.[K]	7662158	FBXO28	23219	7662158 [326-346]	1xOxidation [M3];1xPhospho [S];1xTMT6plex [N-Term]	S19(Phospho): 98.91	10	1.107	0.575122
[K].KLSVPTSDEEDEVPAPKPR.[G]	69354671	ABCF1	23	69354671 [103-121]	3xPhospho [S3(100);3xTMT6plex [N-Term;K1;K17];S7(100)];T6(100)	S3(Phospho): 100;T6(Phospho): 100;S7(Phospho): 100	2	0.434	0.573718
[K].ISMQDVDLSLGSPK.[L]	1079280806	AHNAK	79026	1079280806 [500-513]	1xOxidation [M3];1xPhospho [S12(100)];2xTMT6plex [N-Term;K14]	S12(Phospho): 99.98	1	0.88	0.572443
[K].AGLESGAEPGDGDSDTTK.[K]	4503337	DKC1	1736	4503337 [481-498]	2xPhospho [S5(100);2xTMT6plex [N-Term;K18];T/S]	S5(Phospho): 99.96;S14(Phospho): 49.52;T17(Phospho): 49.54	1	0.903	0.572259
[K].GADNDGSGSESGYTTPK.[K]	32698730	NUFIP2	57532	32698730 [206-222]	2xTMT6plex [N-Term;3xPhospho [T14(99.6);K17];S/Y];T15(99.6)	S7(Phospho): 47.93;S9(Phospho): 47.93;T14(Phospho): 99.33;T15(Phospho): 99.33	4	0.574	0.571772
[R].SSGTASSVAFTPLQGLEIVNPQAAEK.[K]	221136939	PRPF31	26121	221136939 [445-470]	2xPhospho [S/T];2xTMT6plex [N-Term;K26]	S1(Phospho): 33.33;S2(Phospho): 33.33;T4(Phospho): 33.33;S6(Phospho): 33.33;S7(Phospho): 33.33;T11(Phospho): 33.33	1	2.612	0.571525
[R].DGQVINETSQHHDDLE.[-]	578818565	VIM	7431	578818565 [451-466]	1xPhospho [S/T];1xTMT6plex [N-Term]	S9(Phospho): 98.81	5	1.124	0.570239
[R].APQTSSSPPPVRR.[G]	1034554585	SRRM1	10250	1034554585 [729-741]	1xPhospho [T4(98.3)];1xTMT6plex [N-Term]	T4(Phospho): 98.33	1	1.429	0.570047
[K].ATNESEDEIPQLVPIGK.[K]	118498359	RSL1D1	26156	118498359 [357-373]	1xPhospho [S/T];2xTMT6plex [N-Term;K17]	S5(Phospho): 99.97	6	1.338	0.569329
[K].SLPAPVAQRPDSPGGGLQAPGQK.[R]	157676331	HABP4	22927	157676331 [97-119]	1xPhospho [S12(100)];2xTMT6plex [N-Term;K23]	S12(Phospho): 100	3	0.577	0.567817
[K].MESEGGADDSAEEGDLLDDDDNEDR.[G]	117190192	HNRNPC	3183	117190192 [251-275]	1xOxidation [M1];1xPhospho [S];1xTMT6plex [N-Term]	S10(Phospho): 95.89	1	1.67	0.565900
[K].EVGDVIALSDITSSGAADRSQEPSPVGSR.[R]	768010208	GRAMD1A	57655	768010208 [331-359]	1xTMT6plex [N-Term];2xPhospho [S24(94.9);T/S]	T12(Phospho): 25.3;S13(Phospho): 25.3;S14(Phospho): 25.3;S20(Phospho): 25.3;S24(Phospho): 48.99;S28(Phospho): 48.99	3	1.043	0.564616
[R].RGSSPGSLEIPK.[D]	578825674	RALGAPA1	253959	578825674 [1364-1375]	2xPhospho [S3(100);2xTMT6plex [N-Term;K12];S4(100)]	S3(Phospho): 100;S4(Phospho): 100	2	0.459	0.564601
[R].KPVTVSPTTPTSPTEGEAS.[-]	166795297	DYNC1LI1	51143	166795297 [505-523]	2xPhospho [S/T];2xTMT6plex [N-Term;K1]	S6(Phospho): 100;T8(Phospho): 97.84	9	0.763	0.564589
[K].STGSPTQETQAPFIAK.[R]	1034675221	ZNF185	7739	1034675221 [203-218]	1xPhospho [S/T];2xTMT6plex [N-Term;K16]	S1(Phospho): 32.9;T2(Phospho): 32.9;S4(Phospho): 32.9	3	0.481	0.563033
[R].VRGGAPDPSPGATATPGAPAQPSSPDAR.[R]	1034608146	ZBTB7A	51341	1034608146 [553-580]	1xTMT6plex [N-Term];2xPhospho [S9(95.8);T/S]	S9(Phospho): 91.97;S23(Phospho): 47.97;S24(Phospho): 47.97	1	1.445	0.562763
[R].LPRSPSPYSR.[R]	1034657270	CDK13	8621	1034657270 [355-364]	1xTMT6plex [N-Term];2xPhospho [S4(100);S6(100)]	S4(Phospho): 100;S6(Phospho): 100	3	1.093	0.562672
[R].SIGSAVDQGNESIVAK.[T]	1370461749	RACGAP1	29127	1370461749 [222-237]	2xPhospho [S1(100);2xTMT6plex [N-Term;K16];S4(100)]	S1(Phospho): 100;S4(Phospho): 100	2	2.143	0.561350
[R].GNSRPGTPSAEGGSTSSTLR.[A]	1370474986	GTF2F1	2962	1370474986 [408-427]	1xTMT6plex [N-Term];2xPhospho [S3(100);T7(100)]	S3(Phospho): 100;T7(Phospho): 100	2	0.402	0.561297
[R].VSPPAPGSAPETPEDK.[S]	33356170	MYO9B	4650	33356170 [1260-1275]	2xPhospho [S8(95);2xTMT6plex [N-Term;K16];T12(95)]	S8(Phospho): 99.98;T12(Phospho): 100	2	0.452	0.559846
[R].ANSGGVDLDSSGEFASIEK.[M]	51173737	RREB1	6239	51173737 [1165-1183]	2xPhospho [S3(98.3);2xTMT6plex [N-Term;K19];S]	S3(Phospho): 98.26;S10(Phospho): 50;S11(Phospho): 50	1	0.949	0.558804
[R].RPAEATSSPTSPERPR.[H]	13236559	C1orf35	79169	13236559 [210-225]	1xTMT6plex [N-Term];2xPhospho [S11(96.7);S/T]	S7(Phospho): 49.99;S8(Phospho): 49.99;S11(Phospho): 99.97	10	1.612	0.558688
[R].GAESPFEEK.[S]	153945728	MAP1B	4131	153945728 [1424-1432]	1xPhospho [S4(100)];2xTMT6plex [N-Term;K9]	S4(Phospho): 100	2	0.436	0.558504
[R].LEISPDSSPER.[A]	148664226	PKP2	5318	148664226 [148-158]	1xTMT6plex [N-Term];2xPhospho [S4(100);S]	S4(Phospho): 99.99;S7(Phospho): 50;S8(Phospho): 50	16	0.756	0.557895
[R].RGSFDATGSGFSMTFSSSSYSSSGYGR.[R]	530389151	PLEC	5339	530389151 [4508-4534]	1xOxidation [M13];1xTMT6plex [N-Term];2xPhospho [S3(100);S/T/Y]	S3(Phospho): 100;S12(Phospho): 16.37;T14(Phospho): 16.37;S16(Phospho): 16.37;S17(Phospho): 16.37;S18(Phospho): 16.37;S19(Phospho): 16.37	1	0.746	0.557524
[K].AGLESGAEPGDGDSDTTK.[K]	4503337	DKC1	1736	4503337 [481-498]	1xPhospho [S/T];2xTMT6plex [N-Term;K18]	S14(Phospho): 99.99	18	1.264	0.557261
[K].NSYNNSQAPSPGLGSK.[A]	530379849	AFF4	27125	530379849 [1034-1049]	1xPhospho [S10(100)];2xTMT6plex [N-Term;K16]	S10(Phospho): 100	5	1.366	0.556257
[R].SNSLNNGQPK.[S]	41393559	KIF1B	23095	41393559 [1051-1060]	1xPhospho [S3(100)];2xTMT6plex [N-Term;K10]	S3(Phospho): 99.99	2	0.473	0.555978
[R].RGSDPASGEVEASQLR.[R]	548923663	TMEM40	55287	548923663 [151-166]	1xPhospho [S3(100)];1xTMT6plex [N-Term]	S3(Phospho): 100	4	0.789	0.555780
[K].ADTTTPTPTAILAPGSPASPPGSLEPK.[A]	313747417	BRD2	6046	313747417 [283-309]	2xPhospho [T/S];2xTMT6plex [N-Term;K27]	T9(Phospho): 63.03;S16(Phospho): 63.03;S19(Phospho): 63.03	2	0.828	0.555405
[R].SLYASSPGGVYATR.[S]	578818565	VIM	7431	578818565 [51-64]	1xPhospho [S/Y/T];1xTMT6plex [N-Term]	S5(Phospho): 49.99;S6(Phospho): 49.99	10	1.049	0.555286
[R].VNDAEPGSPEAPQGK.[R]	154759257	DDX51	317781	154759257 [76-90]	1xPhospho [S8(100)];2xTMT6plex [N-Term;K15]	S8(Phospho): 100	6	1.173	0.553473
[R].APESPRVPSPPPSPR.[L]	388240778	PLEKHG5	57449	388240778 [922-936]	1xTMT6plex [N-Term];2xPhospho [S13(100);S]	S9(Phospho): 100;S13(Phospho): 100	3	1.067	0.552351
[K].EGAASPAPETPQPTSPETSPK.[E]	7661690	CCDC9	26093	7661690 [372-392]	1xPhospho [T/S];2xTMT6plex [N-Term;K21]	T10(Phospho): 20;T14(Phospho): 20;S15(Phospho): 20;T18(Phospho): 20;S19(Phospho): 20	5	1.158	0.552235
[R].VADAKGDSESEEDEDLEVPVPSR.[F]	767923834	PRKAR2A	5576	767923834 [71-93]	2xPhospho [S8(100);2xTMT6plex [N-Term;K5];S]	S8(Phospho): 100;S10(Phospho): 100	8	0.806	0.551664
[R].GAVAAEGASDTEREEPTESQGLAAR.[L]	557440888	PHRF1	57661	557440888 [907-931]	1xTMT6plex [N-Term];2xPhospho [S9(100);T11(94.4)]	S9(Phospho): 99.99;T11(Phospho): 99.85	2	1.109	0.548788
[K].GFGFGQGAGALVHSE.[-]	4758086	CSRP1	1465	4758086 [179-193]	1xPhospho [S14(100)];1xTMT6plex [N-Term]	S14(Phospho): 100	3	0.495	0.548474
[K].AGGSPASYHGSTSPR.[V]	156671217	EPN2	22905	156671217 [189-203]	1xTMT6plex [N-Term];2xPhospho [S4(100);S/Y/T]	S4(Phospho): 100;S7(Phospho): 98.89	4	1.304	0.545735
[K].TSSVSNPQDSVGSPCSR.[V]	1034580879	PXN	5829	1034580879 [100-116]	1xCarbamidomethyl [C15];1xPhospho [S13(96.8)];1xTMT6plex [N-Term]	S13(Phospho): 98.84	3	0.893	0.545496
[R].STPESGDSDKESVGSSSTSNEGGR.[R]	767909928	OTUD7B	56957	767909928 [469-492]	2xPhospho [S/T];2xTMT6plex [N-Term;K10]	S5(Phospho): 96.8;S8(Phospho): 93.63	2	0.34	0.544581
[K].ELVSSSSSGSDSDSEVDKK.[L]	1034643399	SUB1	10923	1034643399 [6-24]	2xPhospho [S];3xTMT6plex [N-Term;K18;K19]	S4(Phospho): 25;S5(Phospho): 25;S6(Phospho): 25;S7(Phospho): 25;S8(Phospho): 25;S10(Phospho): 25;S12(Phospho): 25;S14(Phospho): 25	7	0.432	0.541723
[R].LGNALGMIGVAGGLAATLGVLKPGPELLAQMSGAMALGGTIGLTIAK.[R]	530378748	NNT	23530	530378748 [646-692]	1xOxidation [M];1xPhospho [T/S];3xTMT6plex [N-Term;K22;K47]	T40(Phospho): 46.65;T44(Phospho): 46.65	9	0.972	0.541563
[R].IASDEEIQGTK.[D]	767931464	PALLD	23022	767931464 [959-969]	1xPhospho [S3(100)];2xTMT6plex [N-Term;K11]	S3(Phospho): 100	4	1.134	0.539249
[K].GYYSPYSVSGSGSTAGSR.[T]	530389151	PLEC	5339	530389151 [4478-4495]	1xTMT6plex [N-Term];2xPhospho [S4(91.9);S/Y/T]	S4(Phospho): 99.98;S9(Phospho): 98.78	46	1.118	0.539082
[K].GLAEVQQDGEAEEGATSDGEK.[K]	1034652357	AKAP12	9590	1034652357 [582-602]	1xPhospho [T/S];2xTMT6plex [N-Term;K21]	S17(Phospho): 98.49	11	0.689	0.537875
[R].SVASNQSEMEFSSLQDMPK.[E]	24432106	CCAR2	57805	24432106 [675-693]	2xOxidation [M9;2xPhospho [S4(100);2xTMT6plex [N-Term;K19];M17];S7(100)]	S4(Phospho): 99.97;S7(Phospho): 99.97	3	1.292	0.537153
[K].APVSSTESVIQSNTPTPPPSQPLNETAEEESR.[I]	530367488	EML4	27436	530367488 [895-926]	1xTMT6plex [N-Term];2xPhospho [S/T]	S12(Phospho): 65.75;T14(Phospho): 65.75;T16(Phospho): 65.75	5	0.828	0.534898
[R].TGSVQTPPDLESSEEVDAAEGAPEVVGPK.[S]	194363771	ITPRID2	6744	194363771 [1163-1191]	2xPhospho [T6(87.3);2xTMT6plex [N-Term;K29];S/T]	T6(Phospho): 91.76;S12(Phospho): 50.72;S13(Phospho): 50.72	4	0.971	0.532827
[K].LMLSTSEYSQSPK.[M]	213972636	TP53BP1	7158	213972636 [520-532]	1xOxidation [M2];2xPhospho [S11(96.9);2xTMT6plex [N-Term;K13];T/S/Y]	T5(Phospho): 33.03;S6(Phospho): 33.03;Y8(Phospho): 33.03;S11(Phospho): 99.98	4	1.359	0.531954
[K].STPQPPSGKTTPNSGDVQVTEDAVR.[R]	1370474986	GTF2F1	2962	1370474986 [461-485]	2xPhospho [S/T];2xTMT6plex [N-Term;K9]	S7(Phospho): 48.95;T10(Phospho): 48.95;T11(Phospho): 48.95;S14(Phospho): 48.95	4	0.703	0.531604
[R].ASSHSSQTQGGGSVTK.[K]	27436946	LMNA	4000	27436946 [402-417]	1xPhospho [S/T];2xTMT6plex [N-Term;K16]	S2(Phospho): 49.71;S3(Phospho): 49.71	3	2.171	0.531356
[R].RPPEEDFRHSPEEDFRQSPQEHFR.[R]	530389014	RBM12B	389677	530389014 [701-724]	1xTMT6plex [N-Term];2xPhospho [S10(100);S18(100)]	S10(Phospho): 100;S18(Phospho): 100	2	0.561	0.529596
[K].NFYESDDDQKEK.[T]	32484979	AP3B1	8546	32484979 [272-283]	1xPhospho [Y/S];3xTMT6plex [N-Term;K10;K12]	Y3(Phospho): 50;S5(Phospho): 50	2	0.22	0.529352
[R].GRSIDQDYER.[A]	282165810	TJP2	9414	282165810 [273-282]	1xPhospho [S3(100)];1xTMT6plex [N-Term]	S3(Phospho): 100	2	0.314	0.529245
[R].NSADDEELTNDSLTLSQSK.[S]	1034657484	PHF14	9678	1034657484 [279-297]	2xPhospho [T9(100);2xTMT6plex [N-Term;K19];S12(98.2)]	T9(Phospho): 49.62;S12(Phospho): 98.45;T14(Phospho): 50.38	3	1.35	0.528347
[K].AQSLVISPPAPSPR.[K]	103472005	MKI67	4288	103472005 [573-586]	1xTMT6plex [N-Term];2xPhospho [S12(100);S]	S7(Phospho): 99.95;S12(Phospho): 100	2	0.885	0.528024
[R].TNTNVNCPIECFMPLDVQADREDSRE.[-]	47132557	FN1	2335	47132557 [2452-2477]	1xOxidation [M13];1xPhospho [S24(100)];1xTMT6plex [N-Term];2xCarbamidomethyl [C7;C11]	S24(Phospho): 100	1	1.638	0.527387
[R].ALPNNTSSSPQPK.[K]	120407068	TP53	7157	120407068 [307-319]	1xPhospho [S/T];2xTMT6plex [N-Term;K13]	S8(Phospho): 50;S9(Phospho): 50	5	0.9	0.526840
[R].LVGATATSSPPPK.[A]	42716295	FYTTD1	84248	42716295 [8-20]	1xPhospho [T/S];2xTMT6plex [N-Term;K13]	T7(Phospho): 33.25;S8(Phospho): 33.25;S9(Phospho): 33.25	3	0.718	0.526668
[R].VLDTSSLTQSAPASPTNK.[G]	22094987	RPTOR	57521	22094987 [850-867]	1xPhospho [S14(99)];2xTMT6plex [N-Term;K18]	S14(Phospho): 99.99	3	0.711	0.526621
[R].LIIESPSNTSSTEPA.[-]	4758024	COIL	8161	4758024 [562-576]	1xPhospho [S5(98.5)];1xTMT6plex [N-Term]	T12(Phospho): 90.74	9	1.522	0.526280
[K].EVAENQQNQSSDPEEEKGSQPPAAESQSSLR.[R]	1370507498	EPB41L2	2037	1370507498 [29-60]	1xPhospho [S];2xTMT6plex [N-Term;K17]	S10(Phospho): 33.33;S11(Phospho): 33.33;S19(Phospho): 33.33	9	2.047	0.526009
[K].SKSPSPPRLTEDR.[K]	7662238	ACIN1	22985	7662238 [384-396]	2xPhospho [S3(97.6);2xTMT6plex [N-Term;K2];S5(99.9)]	S3(Phospho): 99.97;S5(Phospho): 100	5	1.868	0.524464
[R].TFSATVR.[A]	1034594161	CARHSP1	23589	1034594161 [71-77]	1xPhospho [S3(100)];1xTMT6plex [N-Term]	S3(Phospho): 100	1	0.326	0.522543
[K].EFGYDSPHDLDSD.[-]	269315880	SLC7A6OS	84138	269315880 [297-309]	1xTMT6plex [N-Term];2xPhospho [S6(100);S12(100)]	S6(Phospho): 99.97;S12(Phospho): 100	2	0.36	0.522124
[K].ELKTDSSPNQAR.[A]	530425325	PTBP1	5725	530425325 [137-148]	1xPhospho [S/T];2xTMT6plex [N-Term;K3]	S6(Phospho): 50;S7(Phospho): 50	4	3.662	0.522065
[R].ARLSASTASELSPK.[S]	78042577	MAP7D1	55700	78042577 [449-462]	2xPhospho [S4(100);2xTMT6plex [N-Term;K14];S12(100)]	S4(Phospho): 99.98;S6(Phospho): 49.41;S12(Phospho): 49.42	1	0.704	0.521826
[R].REEQTDTSDGESVTHHIR.[R]	530381804	GNL1	2794	530381804 [44-61]	1xTMT6plex [N-Term];2xPhospho [T7(99.4);T/S]	T5(Phospho): 100;T7(Phospho): 50;S8(Phospho): 50	4	0.433	0.520911
[R].DALGDSLQVPVSPSSTTSSR.[C]	6005709	PALM2-AKAP2	445815	6005709 [372-391]	1xPhospho [S12(88.1)];1xTMT6plex [N-Term]	S12(Phospho): 99.99	6	1.165	0.520330
[K].LIHEQEQQSSS.[-]	948549834	S100A16	140576	948549834 [93-103]	1xPhospho [S];1xTMT6plex [N-Term]	S10(Phospho): 49.82;S11(Phospho): 49.82	16	1.137	0.519898
[K].SHVEDGDIAGAPASSPEAPPAEQDPVQLK.[T]	110611218	RRBP1	6238	110611218 [830-858]	2xPhospho [S1(99.6);2xTMT6plex [N-Term;K29];S]	S14(Phospho): 100;S15(Phospho): 100	1	1.755	0.518631
[K].KPSPQPSSPR.[Q]	150417989	CCNK	8812	150417989 [322-331]	2xPhospho [S3(100);2xTMT6plex [N-Term;K1];S]	S3(Phospho): 100;S7(Phospho): 50;S8(Phospho): 50	7	1.835	0.518538
[R].SSPPASPTIPR.[L]	767980601	MAP3K9	4293	767980601 [547-558]	1xTMT6plex [N-Term];2xPhospho [S2(98.4);S6(96.8)]	S1(Phospho): 50.01;S2(Phospho): 50.01;S6(Phospho): 98.25	3	0.937	0.517621
[R].GAASTLVPGVSETSASPGSPSVR.[S]	146219843	SRCAP	10847	146219843 [2772-2794]	1xTMT6plex [N-Term];2xPhospho [S/T]	S11(Phospho): 87.54;T13(Phospho): 87.54	5	0.962	0.517361
[R].ELVGDTGSQEGDHEPSGSETEEDTSSSPHR.[I]	207440661	SMIM13	221710	207440661 [43-72]	1xTMT6plex [N-Term];2xPhospho [T/S]	T6(Phospho): 39.74;S8(Phospho): 39.74;S16(Phospho): 39.74;S18(Phospho): 39.74;T20(Phospho): 39.74	3	1.277	0.516810
[R].EATGLSPQAAQEK.[D]	767948692	ZKSCAN1	7586	767948692 [8-20]	1xPhospho [S6(98)];2xTMT6plex [N-Term;K13]	S6(Phospho): 98.88	2	0.745	0.516548
[R].AFVEDSEDEDGAGEGGSSLLQK.[R]	145580615	KRI1	65095	145580615 [172-193]	1xPhospho [S6(100)];2xTMT6plex [N-Term;K22]	S6(Phospho): 100	13	1.676	0.516053
[K].AASPSPQSVR.[R]	1034554585	SRRM1	10250	1034554585 [775-784]	1xTMT6plex [N-Term];2xPhospho [S3(100);S5(100)]	S3(Phospho): 100;S5(Phospho): 100	3	1.382	0.514156
[R].DTMDLESSSSEEEKEDDDDALVPDSK.[Q]	1022433638	MIA3	375056	1022433638 [399-424]	1xOxidation [M3];2xPhospho [T/S];3xTMT6plex [N-Term;K14;K26]	S8(Phospho): 65.55;S9(Phospho): 65.55;S10(Phospho): 65.55	2	0.901	0.514127

Patil et al,2020. Proteomic and phosphoproteomic profiling of shammah-induced signaling in oral keratinocytes

Supplementary Table S2: List of phosphopeptides identified and quantified in OKF6/TERT1-Shammah and OKF6/TERT1-Parental phosphoproteome using TMT/TiO$_2$-based quantitative phosphoproteomic approach

Peptide Sequence	Protein Accessions	Gene Symbol	Gene ID	Positions in Proteins	Modifications	PhosphoRS: Best Site Probabilities	# PSMs	Fold change	p-value
[K].TLTDEVNSPDSDRR.[D]	1057867268	SMARCC2	6601	1057867268 [276-289]	1xTMT6plex [N-Term];2xPhospho [S8(100);S11(100)]	S8(Phospho): 100;S11(Phospho): 100	3	1.169	0.513913
[K].AGMSSNQSISSPVLDAVPR.[T]	118572613	SRRM2	23524	118572613 [1394-1412]	1xOxidation [M3];1xTMT6plex [N-Term];2xPhospho [S]	S10(Phospho): 88.46;S11(Phospho): 88.46	1	1.173	0.512722
[R].RGAPSSPATGVLPSPQGK.[S]	62955803	NUP188	23511	62955803 [1704-1721]	2xPhospho [S14(100);2xTMT6plex [N-Term;K18];S/T]	S5(Phospho): 49.99;S6(Phospho): 49.99;S14(Phospho): 100	1	1.526	0.512279
[K].KKEPAITSQNSPEAR.[E]	5803191	TCEA1	6917	5803191 [90-104]	1xPhospho [S11(100)];3xTMT6plex [N-Term;K1;K2]	S11(Phospho): 100	2	0.337	0.511726
[R].EAARSPDKPGGSPSASR.[R]	157694511	NOC2L	26155	157694511 [45-61]	2xPhospho [S5(100);2xTMT6plex [N-Term;K8];S12(96)]	S5(Phospho): 100;S12(Phospho): 95.97	1	0.625	0.511715
[K].RPPTPCSDPER.[K]	21396500	HIRIP3	8479	21396500 [81-91]	1xCarbamidomethyl [C6];1xTMT6plex [N-Term];2xPhospho [T4(100);S7(100)]	T4(Phospho): 100;S7(Phospho): 100	6	0.612	0.511589
[K].VSPSKSPSLSPSPPSPLEK.[T]	153945728	MAP1B	4131	153945728 [1251-1269]	2xPhospho [S];3xTMT6plex [N-Term;K19];K5	S4(Phospho): 19.93;S6(Phospho): 19.93;S8(Phospho): 19.94;S10(Phospho): 19.94;S12(Phospho): 19.94;S15(Phospho): 99.92	6	0.458	0.508157
[K].GADNDGSGSESGYTTPK.[K]	32698730	NUFIP2	57532	32698730 [206-222]	2xPhospho [S7(100);2xTMT6plex [N-Term;K17];S9(94.5)]	S7(Phospho): 97.2;S9(Phospho): 94.52	12	0.828	0.505934
[R].KVEEEGSPGDPDHEASTQGR.[T]	33286446	OGFR	11054	33286446 [309-328]	1xPhospho [S7(100)];2xTMT6plex [N-Term;K1]	S7(Phospho): 100	1	0.358	0.505340
[K].KLEKEEEEGISQESSEEEQ.[-]	22208967	HMGA1	3159	22208967 [89-107]	1xPhospho [S];3xTMT6plex [N-Term;K1;K4]	S14(Phospho): 50;S15(Phospho): 50	4	3.124	0.504661
[R].ASVSPMDEPVPDSESPIEK.[V]	153945728	MAP1B	4131	153945728 [1375-1393]	1xOxidation [M6];1xPhospho [S4(98.5)];2xTMT6plex [N-Term;K19]	S4(Phospho): 98.5	3	0.509	0.503140
[K].IISPGSSTPSSTRSPPPGRDESYPR.[E]	183396804	RPRD2	23248	183396804 [756-780]	1xTMT6plex [N-Term];2xPhospho [S/T/Y]	S6(Phospho): 24.55;S7(Phospho): 24.55;T8(Phospho): 24.55;S10(Phospho): 24.55;S14(Phospho): 85.27	4	0.591	0.503130
[R].SSTPLPTISSSAENTR.[Q]	4507555	TMPO	7112	4507555 [158-173]	1xPhospho [T3(98.8)];1xTMT6plex [N-Term]		1	0.751	0.502810
[R].YESQEPLAGQESPLPLATR.[E]	578821824	TNKS1BP1	85456	578821824 [590-608]	1xPhospho [S12(100)];1xTMT6plex [N-Term]	S12(Phospho): 100	3	0.684	0.501825
[R].TNPEDIYPSNPTDDDVSSGSSSER.[S]	530396004	CD44	960	530396004 [163-186]	1xTMT6plex [N-Term];2xPhospho [S/Y/T]	S17(Phospho): 91.07;S18(Phospho): 33.91;S20(Phospho): 33.91;S21(Phospho): 33.91	13	1.385	0.501392
[R].RLLEGEDAHLSSSQFSSGSQSSRDVTSSSR.[Q]	15431310	KRT14	3861	15431310 [417-446]	1xTMT6plex [N-Term];2xPhospho [S/T]	S11(Phospho): 88.63;S12(Phospho): 15.9;S13(Phospho): 15.9;S16(Phospho): 15.9;S17(Phospho): 15.9;S19(Phospho): 15.9;S21(Phospho): 15.9;S22(Phospho): 15.9	23	0.316	0.500169
[K].IDASKNEEDEGHSNSSPR.[H]	14110420	HNRNPD	3184	14110420 [68-85]	1xPhospho [S];2xTMT6plex [N-Term;K5]	S15(Phospho): 49.62;S16(Phospho): 49.62	19	1.63	0.498984
[R].SSPGGQDEGGFMAQGK.[T]	1034580879	PXN	5829	1034580879 [798-813]	1xOxidation [M12];1xPhospho [S];2xTMT6plex [N-Term;K16]	S2(Phospho): 98.96	1	0.426	0.498289
[K].TSSDDESEEDEDDLLQR.[T]	118344456	UTP18	51096	118344456 [204-220]	1xTMT6plex [N-Term];2xPhospho [T/S]	T1(Phospho): 34.2;S2(Phospho): 34.2;S3(Phospho): 34.2;S7(Phospho): 97.4	1	0.936	0.497257
[R].IIYDSDSESEETLQVK.[N]	325296984	RFC1	5981	325296984 [65-80]	2xPhospho [S/T/Y];2xTMT6plex [N-Term;K16]	S5(Phospho): 99.98;S7(Phospho): 100	14	0.887	0.495582
[K].SPGSTPTTPTSSQAPQK.[L]	33946327	NUP214	8021	33946327 [430-446]	1xPhospho [S/T];2xTMT6plex [N-Term;K17]	S4(Phospho): 97.85	5	0.685	0.494263
[K].EYIPGQPPLSQSSDSSPTR.[N]	62241042	EPRS	2058	62241042 [871-889]	1xPhospho [S/T/Y];1xTMT6plex [N-Term]	S15(Phospho): 49.65;S16(Phospho): 49.65	42	0.56	0.492327
[R].RPPSPDVIVLSDNEQPSSPR.[V]	1034608303	GATAD2A	54815	1034608303 [116-135]	1xTMT6plex [N-Term];2xPhospho [S4(100);S]	S4(Phospho): 100;S17(Phospho): 50;S18(Phospho): 50	9	1.22	0.490730
[R].NAPAAVDEGSISPR.[T]	324715041	BIVM-ERCC5	100533467	324715041 [827-840]	1xPhospho [S12(98.4)];1xTMT6plex [N-Term]	S12(Phospho): 98.44	2	1.842	0.490658
[R].RDTQSNELIEINPQSEGK.[V]	100913220	COL16A1	1307	100913220 [256-273]	1xPhospho [S5(98.6)];2xTMT6plex [N-Term;K18]	S5(Phospho): 98.65	1	1.262	0.490127
[R].SLSNSNPDISGTPTSPDDEVR.[S]	767906494	DOCK7	85440	767906494 [896-916]	1xTMT6plex [N-Term];2xPhospho [S/T]	S1(Phospho): 32.82;S3(Phospho): 32.82;S5(Phospho): 33.12;S10(Phospho): 25.31;T12(Phospho): 25.31;T14(Phospho): 25.31;S15(Phospho): 25.31	1	1.032	0.490052
[R].TSSDPVLLK.[A]	530425057	SH2D3A	10045	530425057 [178-186]	1xPhospho [S/T];2xTMT6plex [N-Term;K9]	S2(Phospho): 50;S3(Phospho): 50	2	0.53	0.488457
[R].RGFSANSARLPGVSR.[S]	119703753;5031839	KRT6B|KRT6A	3854|3853	119703753 [16-30];5031839 [16-30]	1xTMT6plex [N-Term];2xPhospho [S4(100);S14(97.5)]	S4(Phospho): 100;S7(Phospho): 100	5	2.108	0.486682
[K].VPSPLEGSEGDGDTD.[-]	70166645	PUS1	80324	70166645 [413-427]	1xPhospho [T14(100)];1xTMT6plex [N-Term]	T14(Phospho): 100	3	0.814	0.484140
[R].EISSSPTSK.[N]	118572613	SRRM2	23524	118572613 [452-460]	2xPhospho [S4(100);2xTMT6plex [N-Term;K9];S/T]	S3(Phospho): 50.25;S4(Phospho): 50.25;S5(Phospho): 98.48	6	1.81	0.484048
[R].AASPAKPSSLDLVPNLPK.[G]	578810794	SYNPO	11346	578810794 [831-848]	2xPhospho [S3(98.8);3xTMT6plex [N-Term;K18];K6;S]	S3(Phospho): 98.82;S8(Phospho): 50.59;S9(Phospho): 50.59	4	1.072	0.483476
[K].GEEGSDDDETENGPKPK.[K]	7661950	CTR9	9646	7661950 [966-982]	1xPhospho [S/T];3xTMT6plex [N-Term;K15;K17]	S5(Phospho): 100	1	0.607	0.483420
[K].ALVVPEPEPDSDSNQER.[K]	767957892	RNF20	56254	767957892 [126-142]	1xPhospho [S13(98.4)];1xTMT6plex [N-Term]	S13(Phospho): 98.69	16	0.937	0.483415
[K].QTSIQSPSSYGNSSPPLNK.[M]	31543818	TP63	8626	31543818 [450-468]	1xPhospho [S/Y/T];2xTMT6plex [N-Term;K19]	S8(Phospho): 19.95;S9(Phospho): 19.95;Y10(Phospho): 19.95;S13(Phospho): 19.95;S14(Phospho): 19.95	10	1.765	0.481663
[R].ACASPSAQVEGSPVAGSDGSQPAVK.[L]	23097240	LRWD1	222229	23097240 [248-272]	1xCarbamidomethyl [C2];2xPhospho [S12(94.7);2xTMT6plex [N-Term;K25];S]	S4(Phospho): 50.02;S6(Phospho): 50.02;S12(Phospho): 94.66	1	1.09	0.481249
[R].RNSSSPVSPASVPGQR.[R]	33457336	IRF2BPL	64207	33457336 [655-670]	1xTMT6plex [N-Term];3xPhospho [S3(100);S5(98.1);S8(100)]	S3(Phospho): 100;S5(Phospho): 98.08;S8(Phospho): 100	3	0.717	0.480399
[R].SKETSSPGTDDVFTPAPSDSPSSQR.[I]	27777632	SLC9A1	6548	27777632 [766-790]	2xPhospho [T/S];2xTMT6plex [N-Term;K2]	S5(Phospho): 48.28;S6(Phospho): 48.28;S18(Phospho): 48.34;S20(Phospho): 48.34	1	3.237	0.479773
[R].AESPESSAIESTQSTPQK.[G]	530402180	PDS5B	23047	530402180 [1357-1374]	1xPhospho [S3(100)];2xTMT6plex [N-Term;K18]	S3(Phospho): 100	7	1.528	0.479738
[K].RSSDGSLSHEEDLAK.[V]	578821804	PPFIA1	8500	578821804 [237-251]	2xPhospho [S6(96);2xTMT6plex [N-Term;K15];S8(96)]	S2(Phospho): 50;S3(Phospho): 50;S6(Phospho): 99.98	5	0.666	0.479316
[K].SDISPLTPR.[E]	153945728	MAP1B	4131	153945728 [1782-1790]	1xTMT6plex [N-Term];2xPhospho [S4(100);T7(100)]	S4(Phospho): 99.98;T7(Phospho): 100	1	0.322	0.479077
[K].IAAPELHKGDSDSEEDEPTK.[K]	530365024	PRCC	5546	530365024 [147-166]	2xPhospho [S11(99.9);3xTMT6plex [N-Term;K20];K8;S13(99.9)]	S11(Phospho): 99.89;S13(Phospho): 99.89	2	0.755	0.478753
[K].ALVVPEPEPDSDSNQER.[K]	767957892	RNF20	56254	767957892 [126-142]	1xTMT6plex [N-Term];2xPhospho [S11(100);S13(100)]	S11(Phospho): 100;S13(Phospho): 100	4	1.205	0.478101
[R].RPPSPEPSTK.[V]	530368012	SPTBN1	6711	530368012 [2099-2108]	1xPhospho [S4(100)];2xTMT6plex [N-Term;K10]	S4(Phospho): 100	3	1.307	0.477893
[K].LLSSGSRYLIR.[S]	1370513191	ARHGEF10	9639	1370513191 [637-647]	1xTMT6plex [N-Term];2xPhospho [S6(89.8);Y8(89.8)]	S6(Phospho): 89.75;Y8(Phospho): 89.75	1	0.785	0.476695
[R].HVDSLSQRSPK.[A]	164698494	40057	10801	164698494 [77-87]	1xPhospho [S9(98.8)];2xTMT6plex [N-Term;K11]	S9(Phospho): 98.79	1	0.46	0.475604
[K].VEMYSGSDDDDDFNKLPK.[K]	84043963	EIF5B	9669	84043963 [131-148]	1xOxidation [M3];2xPhospho [Y/S];3xTMT6plex [N-Term;K15;K18]	Y4(Phospho): 66.67;S5(Phospho): 66.67;S7(Phospho): 66.67	7	0.239	0.474532
[R].AFQLEEGEETEPDCK.[Y]	4758024	COIL	8161	4758024 [113-127]	1xCarbamidomethyl [C14];1xPhospho [T10(100)];2xTMT6plex [N-Term;K15]	T10(Phospho): 100	1	1.682	0.471143
[K].HSLDSDEEEDDDDGGSSK.[Y]	5174409	CD2BP2	10421	5174409 [45-62]	1xPhospho [S5(100)];2xTMT6plex [N-Term;K18]	S5(Phospho): 99.98	2	0.769	0.468771
[R].KTSSDDESEEDEDDLLQR.[T]	118344456	UTP18	51096	118344456 [203-220]	2xPhospho [S/T];2xTMT6plex [N-Term;K1]	S4(Phospho): 97.84;S8(Phospho): 100	10	1.374	0.466451
[R].RGSLSNAGDPEIVK.[S]	156071450	NRDC	4898	156071450 [92-105]	1xPhospho [S3(100)];2xTMT6plex [N-Term;K14]	S3(Phospho): 100	2	0.315	0.466045
[R].SIDDEITEAKSGTATPQR.[S]	94557299	PRKAA1	5562	94557299 [491-508]	2xPhospho [S11(98.6);2xTMT6plex [N-Term;K10];T15(100)]	S11(Phospho): 98.62;T15(Phospho): 99.98	5	0.731	0.465600
[R].LQRLPSSSSEMGSQDGSPLR.[E]	109148539	GGT7	2686	109148539 [67-86]	1xOxidation [M11];1xTMT6plex [N-Term];2xPhospho [S6(97.8);S]	S6(Phospho): 97.75;S7(Phospho): 34.08;S8(Phospho): 34.08;S9(Phospho): 34.08	2	2.89	0.464723
[R].SASQSSLDKLDQELK.[E]	530413055	SPAG9	9043	530413055 [728-742]	2xPhospho [S3(98.5);3xTMT6plex [N-Term;K15];K9;S]	S3(Phospho): 98.53;S5(Phospho): 50.69;S6(Phospho): 50.69	2	0.232	0.464282
[R].AGDLLEDSPK.[R]	984290516	HDGF	3068	984290516 [181-190]	1xPhospho [S8(100)];2xTMT6plex [N-Term;K10]	S8(Phospho): 100	2	0.487	0.464248
[K].KESESEDSSDDEPLIK.[K]	1034651579	DEK	7913	1034651579 [299-314]	2xPhospho [S8(99.8);3xTMT6plex [N-Term;K1;K16];S9(99.8)]	S8(Phospho): 99.85;S9(Phospho): 99.85	1	2.51	0.461332
[R].LSGGLGAGSCR.[L]	4557701	KRT17	3872	4557701 [31-41]	1xCarbamidomethyl [C10];1xPhospho [S9(100)];1xTMT6plex [N-Term]	S9(Phospho): 100	2	2.549	0.460689
[K].HIVNHDDVFEESEELSSDEEMK.[M]	530379403	ERBIN	55914	530379403 [587-608]	1xOxidation [M21];2xPhospho [S];2xTMT6plex [N-Term;K22]	S12(Phospho): 66.67;S16(Phospho): 66.67;S17(Phospho): 66.67	2	0.529	0.459864
[K].GAGDGSDEEVDGKADGAEAKPAE.[-]	1034628998	MYH9	4627	1034628998 [1938-1960]	1xPhospho [S6(100)];3xTMT6plex [N-Term;K13;K20]	S6(Phospho): 100	1	0.234	0.459635
[R].SSQPSPTAVPASDSPPTK.[Q]	78042577	MAP7D1	55700	78042577 [112-129]	2xPhospho [S5(96.9);2xTMT6plex [N-Term;K18];S14(98.4)]	S5(Phospho): 96.92;S14(Phospho): 98.45	1	0.41	0.459221
[R].NMGGPYGGGNYGPGGSGGSGGYGGR.[S]	530384720	HNRNPA2B1	3181	530384720 [326-350]	1xOxidation [M2];1xPhospho [Y22(93.9)];1xTMT6plex [N-Term]	Y22(Phospho): 99.98	7	1.198	0.458893
[R].RVESEESGDEEGKK.[H]	4506013	PPP1R7	5510	4506013 [21-34]	2xPhospho [S4(100);3xTMT6plex [N-Term;K13;K14];S7(100)]	S4(Phospho): 100;S7(Phospho): 100	1	0.126	0.457697
[R].KGGSYSQAASSDSAQGSDVSLTACKV.[-]	1370525687	HLA-A	3105	1370525687 [401-426]	1xCarbamidomethyl [C24];2xPhospho [S/T/Y];3xTMT6plex [N-Term;K1;K25]	S13(Phospho): 50.65;S17(Phospho): 49.11;S20(Phospho): 93.61	7	0.74	0.454976
[K].TSSAFVGKTPEASPEPK.[D]	25777600	PSMD1	5707	25777600 [303-319]	2xPhospho [T/S];3xTMT6plex [N-Term;K17];K8	T9(Phospho): 100;S13(Phospho): 100	4	1.225	0.454520
[R].NNSPPTVGAFGHTR.[C]	148664230	ANKLE2	23141	148664230 [660-673]	1xPhospho [S3(100)];1xTMT6plex [N-Term]	S3(Phospho): 100	2	1.168	0.453713
[K].SATRPSPSPERSSTGPEPPAPTPLLAER.[H]	118572613	SRRM2	23524	118572613 [346-373]	1xTMT6plex [N-Term];3xPhospho [S6(99.6);S/T];S8(99.8)]	S6(Phospho): 95.67;S8(Phospho): 99.4;S12(Phospho): 33.44;S13(Phospho): 33.44;T14(Phospho): 33.44	7	1.29	0.450924
[R].DSPLQGSGQQNSQAGQRNSTSSIEPR.[L]	530368861	MAP4K4	9448	530368861 [715-740]	1xTMT6plex [N-Term];2xPhospho [S/T]	S7(Phospho): 33.32;S12(Phospho): 33.32;S19(Phospho): 33.32;T20(Phospho): 33.32;S21(Phospho): 33.32;S22(Phospho): 33.32	10	0.404	0.448522
[R].LTSIGSDEDEETETYQEK.[V]	110224449	SLC12A6	9990	110224449 [1027-1044]	2xPhospho [T/S/Y];2xTMT6plex [N-Term;K18]	T2(Phospho): 48.95;S3(Phospho): 48.95;S6(Phospho): 48.95;T12(Phospho): 48.95	3	2.798	0.447635
[R].IDFTPVSPAPSPTR.[G]	768038902	FAM122B	159090	768038902 [132-145]	1xTMT6plex [N-Term];2xPhospho [S11(98);S/T]	S7(Phospho): 99.97;S11(Phospho): 98.43	3	0.598	0.447461
[R].GLRDSHSSEEDEASSQTDLSQTISK.[K]	767908953	TOR1AIP1	26092	767908953 [150-174]	2xPhospho [S/T];2xTMT6plex [N-Term;K25]	S7(Phospho): 95.49;S14(Phospho): 33.06;S15(Phospho): 33.06;T17(Phospho): 33.06	10	1.559	0.446925
[R].TPSPNSNEQQK.[S]	145312241	DEF6	50619	145312241 [595-605]	1xPhospho [S3(100)];2xTMT6plex [N-Term;K11]	S3(Phospho): 99.97	2	0.303	0.444660
[R].SCFESSPDPELK.[S]	118572613	SRRM2	23524	118572613 [871-882]	1xCarbamidomethyl [C2];2xPhospho [S1(99.4)];2xTMT6plex [N-Term;K12]	S5(Phospho): 99.99;S6(Phospho): 99.99	3	0.825	0.442727
[K].AESSDSGAESEEEEAQEEVK.[G]	1034570219	ACBD5	91452	1034570219 [257-276]	2xPhospho [S10(96.6);2xTMT6plex [N-Term;K20];S]	S6(Phospho): 95.76;S10(Phospho): 99.84	4	0.636	0.441999
[K].ATRPASTSQPDPSPDRSANTSPESSSR.[L]	1034608877	BCL3	602	1034608877 [604-630]	1xTMT6plex [N-Term];2xPhospho [S13(94.6);S/T]	S13(Phospho): 94.62;S17(Phospho): 34.7;T20(Phospho): 34.7;S21(Phospho): 34.7	2	0.01	0.441450
[R].VNQSALEAVTPSPSFQQR.[H]	6912242	APPL1	26060	6912242 [390-407]	1xPhospho [S/T];1xTMT6plex [N-Term]	S12(Phospho): 99.05	4	0.904	0.441216
[K].IIPELVGSPTQSTSSR.[T]	16445436	BRWD1	54014	16445436 [1468-1483]	1xPhospho [S/T];1xTMT6plex [N-Term]	S8(Phospho): 48.01;T10(Phospho): 48.01	8	0.909	0.440296
[R].AHSPASTLPNSPGSTFER.[K]	119829187	COL17A1	1308	119829187 [83-100]	1xPhospho [S/T];1xTMT6plex [N-Term]	S11(Phospho): 97.3	12	0.744	0.440050
[R].AGSSTPGDAPPAVAEVQGR.[S]	1034673276	HUWE1	10075	1034673276 [3012-3030]	1xPhospho [S/T];1xTMT6plex [N-Term]	S3(Phospho): 33.33;S4(Phospho): 33.33;T5(Phospho): 33.33	3	0.909	0.439368
[K].GNAEGSSDEEGKLVIDEPAKEK.[N]	984290516	HDGF	3068	984290516 [150-171]	2xPhospho [S6(100);4xTMT6plex [N-Term;K12;K20;K22];S7(100)]	S6(Phospho): 100;S7(Phospho): 100	4	0.233	0.439283
[R].IQEQESSGEEDSDLSPEER.[EG]	1186517946	PPP1R2B	153743	1186517946 [116-134]	1xTMT6plex [N-Term];2xPhospho [S6(100);S7(98)]	S6(Phospho): 100;S7(Phospho): 100	6	0.448	0.438811
[R].ASRVPSSDEEVVEEPQSR.[R]	578821824	TNKS1BP1	85456	578821824 [1615-1632]	1xTMT6plex [N-Term];2xPhospho [S7(99.1);S]	S6(Phospho): 100;S7(Phospho): 100	12	0.599	0.437910
[R].GSLSPRSPVSSLQIR.[Y]	1370457836	MLLT10	8028	1370457836 [717-731]	1xTMT6plex [N-Term];2xPhospho [S4(100);S7(100)]	S4(Phospho): 99.98;S7(Phospho): 100	4	1.297	0.437269
[K].ISYIPDEEVSSPSPPQR.[A]	631226390	IGF2BP2	10644	631226390 [158-174]	1xTMT6plex [N-Term];2xPhospho [S/Y]	S10(Phospho): 50.49;S11(Phospho): 50.49;S13(Phospho): 99	4	0.946	0.437259
[K].EDLPAENGETKTEESPASDEAGEKEAK.[S]	48255933	HMGN1	3150	48255933 [72-98]	2xPhospho [S15(92.3);4xTMT6plex [N-Term;K11;K24;K27];S18(100)]	S15(Phospho): 94.82;S18(Phospho): 100	4	1.624	0.436379
[R].GGGSGGGEESEGEEVDED.[-]	15147219	PURB	5814	15147219 [295-312]	1xPhospho [S10(100)];1xTMT6plex [N-Term]	S10(Phospho): 100	3	0.851	0.435362
[K].DQSPPPSPPPSYHPPPPPTK.[K]	112734870	KIAA1522	57648	112734870 [726-745]	2xPhospho [S3(100);2xTMT6plex [N-Term;K20];S/Y/T]	S3(Phospho): 100;S7(Phospho): 96.42	13	0.928	0.433834
[R].RADLNQGIGEPQSPSR.[R]	20149675	EFHD2	79180	20149675 [62-77]	1xPhospho [S13(98.8)];1xTMT6plex [N-Term]	S13(Phospho): 98.75	3	0.828	0.432022
[K].TFNKDTVIIVSEPSEDEESQGLPTMAR.[R]	1370487333	SMARCAD1	56916	1370487333 [114-140]	1xOxidation [M25];2xPhospho [S19(87.9);2xTMT6plex [N-Term;K4];T24(87.9)]	S11(Phospho): 97.72;S14(Phospho): 51.07;S19(Phospho): 51.07	5	0.862	0.431420
[R].DSRSLSYSPVER.[R]	118572613	SRRM2	23524	118572613 [2687-2698]	1xTMT6plex [N-Term];3xPhospho [S4(100);S6(100);S8(100)]	S4(Phospho): 99.96;S6(Phospho): 100;S8(Phospho): 99.96	2	0.441	0.428984
[K].SGDEGSEDEAPSGED.[-]	56118310	NUCKS1	64710	56118310 [229-243]	1xTMT6plex [N-Term];2xPhospho [S6(100);S12(100)]	S6(Phospho): 100;S12(Phospho): 100	2	0.364	0.428087
[R].SGEQITSSPVSPK.[S]	527317388	PPIP5K2	23262	527317388 [1021-1033]	2xPhospho [S11(94.2);2xTMT6plex [N-Term;K13];S/T]	S1(Phospho): 100;S11(Phospho): 100	2	0.622	0.427821
[R].VPGPAEGPAEPAAEASDEAERR.[A]	1034574337	TMEM132A	54972	1034574337 [528-549]	1xPhospho [S16(100)];1xTMT6plex [N-Term]	S16(Phospho): 100	3	1.123	0.426961
[K].DYLLSESEDEGDNDGERK.[H]	21361348	UTP14A	10813	21361348 [25-42]	2xPhospho [S5(100);2xTMT6plex [N-Term;K18];S7(100)]	S5(Phospho): 100;S7(Phospho): 100	2	0.576	0.425301
[R].KHSPSPPPPTPTESR.[K]	188536047	SMARCC1	6599	188536047 [326-340]	2xPhospho [S3(100);2xTMT6plex [N-Term;K1];S5(100)]	S3(Phospho): 100;S5(Phospho): 100	10	0.752	0.425025
[R].SAPASPTHPGLMSPR.[S]	82546824	FOXK1	221937	82546824 [416-430]	1xOxidation [M12];1xTMT6plex [N-Term];2xPhospho [S5(100);S13(100)]	S1(Phospho): 100;S5(Phospho): 99.98	15	0.724	0.424658
[R].ASSHSSQTQGGGSVTK.[K]	27436946	LMNA	4000	27436946 [402-417]	2xTMT6plex [N-Term;3xPhospho [S/T];K16]	S2(Phospho): 50;S3(Phospho): 50;S5(Phospho): 49.47;S6(Phospho): 49.47;S13(Phospho): 49.47;T15(Phospho): 49.47	1	0.828	0.423279
[R].GSSGSPAHAESYSSGGGGQQK.[F]	578802800	FOSL2	2355	578802800 [15-35]	1xPhospho [S/Y];2xTMT6plex [N-Term;K21]	S2(Phospho): 33.31;S3(Phospho): 33.31;S5(Phospho): 33.31	4	0.258	0.423241
[R].MEREDSSEEEEEEIDDEEIER.[R]	50726968	MFAP1	4236	50726968 [127-147]	1xOxidation [M1];1xTMT6plex [N-Term];2xPhospho [S6(100);S7(100)]	S6(Phospho): 100;S7(Phospho): 100	3	1.137	0.422959
[R].VTNDISPESSPGVGR.[R]	767950881	PCM1	5108	767950881 [60-74]	1xPhospho [S6(97.7)];1xTMT6plex [N-Term]	S6(Phospho): 100	1	0.717	0.421916
[R].NSASATTPLSGNSSR.[R]	767924540	LRRFIP2	9209	767924540 [311-325]	1xPhospho [T/S];1xTMT6plex [N-Term]	T7(Phospho): 24.91;S10(Phospho): 24.91;S13(Phospho): 24.91;S14(Phospho): 24.91	6	0.334	0.421432
[K].AQEAEAQSEDDDEDTEEEQGEEK.[E]	1214884389	NOM1	64434	1214884389 [273-295]	2xPhospho [S8(100);2xTMT6plex [N-Term;K23];T15(100)]	S8(Phospho): 100;T15(Phospho): 100	3	1.077	0.421389
[R].TQDPSSPGTTPPQAR.[Q]	1034627629	CHAF1B	8208	1034627629 [424-438]	1xTMT6plex [N-Term];2xPhospho [T10(98.2);S/T]	S5(Phospho): 50.34;S6(Phospho): 50.34;T10(Phospho): 97.93	4	0.676	0.421344
[K].TSTSPPPEKSGDEGSEDEAPSGED.[-]	56118310	NUCKS1	64710	56118310 [220-243]	2xTMT6plex [N-Term;3xPhospho [S/T];K9]	S2(Phospho): 88.3;S10(Phospho): 91.76;S15(Phospho): 91.76	1	0.453	0.421083
[R].LDIDSPPITAR.[N]	332164775	PKM	5315	332164775 [107-117]	1xPhospho [S5(100)];1xTMT6plex [N-Term]	S5(Phospho): 98.39	2	0.456	0.420603
[R].GQTPLTEGSEDLDGHSDPEESFAR.[D]	30023820	RETREG3	162427	30023820 [305-328]	1xTMT6plex [N-Term];2xPhospho [T/S]	S9(Phospho): 99.96;S16(Phospho): 100	5	1.434	0.419345
[R].SGSPSDNSGAEEMEVSLAKPK.[H]	62241015	AKT1	207	62241015 [122-142]	1xOxidation [M13];1xPhospho [S];3xTMT6plex [N-Term;K19;K21]	S3(Phospho): 32.77;S5(Phospho): 32.77;S16(Phospho): 32.77	2	0.451	0.418377
[K].EAEDSDSDDNIKR.[G]	530369278	IWS1	55677	530369278 [512-524]	2xPhospho [S5(100);2xTMT6plex [N-Term;K12];S7(100)]	S5(Phospho): 100;S7(Phospho): 100	3	0.648	0.416682
[R].EPSVPASCAVSATYERSPSPAHLPDDPK.[V]	1034567413	LARP4B	23185	1034567413 [1083-1110]	1xCarbamidomethyl [C8];2xPhospho [S/T/Y];2xTMT6plex [N-Term;K28]	S7(Phospho): 33.31;S11(Phospho): 33.31;T13(Phospho): 33.31;Y14(Phospho): 33.31;S17(Phospho): 33.31;S19(Phospho): 33.31	10	0.892	0.415375
[R].SELSQDAEPAGSQETK.[D]	21361348	UTP14A	10813	21361348 [434-449]	1xPhospho [S/T];2xTMT6plex [N-Term;K16]	S12(Phospho): 99.16	4	0.841	0.413377
[R].AELGMGDSTSQSPPIKR.[S]	768017601	ACSS2	55902	768017601 [269-285]	1xOxidation [M5];1xPhospho [S/T];2xTMT6plex [N-Term;K16]	S8(Phospho): 25;T9(Phospho): 25;S10(Phospho): 25;S12(Phospho): 25	2	0.505	0.409551
[R].LKLSPSPSSR.[V]	5031877;388240801	#N/A	#N/A	5031877 [388-397];388240801 [402-411]	2xPhospho [S4(100);2xTMT6plex [N-Term;K2];S6(100)]	S4(Phospho): 99.96;S6(Phospho): 98.24	4	1.666	0.409527
[R].GGQGDPAVPTQQPADPSTPEQQNSPSGSEQFVR.[R]	1034675221	ZNF185	7739	1034675221 [573-605]	1xPhospho [T/S];1xTMT6plex [N-Term]	T10(Phospho): 33.32;S17(Phospho): 33.32;T18(Phospho): 33.32	13	0.904	0.409171
[K].STFREESPLR.[I]	7661958	BCLAF1	9774	7661958 [525-534]	1xPhospho [S7(100)];1xTMT6plex [N-Term]	S7(Phospho): 100	3	1.275	0.409098
[R].RVESEESGDEEGK.[K]	4506013	PPP1R7	5510	4506013 [21-33]	2xPhospho [S4(100);2xTMT6plex [N-Term;K13];S7(100)]	S4(Phospho): 100;S7(Phospho): 100	2	0.217	0.408897
[R].DDGVFVQEVTQNSPAAR.[T]	1079280806	AHNAK	79026	1079280806 [29-45]	1xPhospho [T10(98.9)];1xTMT6plex [N-Term]	T10(Phospho): 98.73	4	0.728	0.408255
[K].VPSPLEGSEGDGDTD.[-]	70166645	PUS1	80324	70166645 [413-427]	1xTMT6plex [N-Term];2xPhospho [S8(100);T14(100)]	S8(Phospho): 100;T14(Phospho): 100	2	0.609	0.407394
[K].SGTPPRQGSITSPQANEQSVTPQRR.[S]	118572613	SRRM2	23524	118572613 [846-870]	1xTMT6plex [N-Term];2xPhospho [T/S]	T3(Phospho): 48.49;S9(Phospho): 48.49;T21(Phospho): 97.02	3	3.303	0.406087
[R].RPDHSGGSPSPPTSEPAR.[S]	21314720	SNIP1	79753	21314720 [45-62]	1xTMT6plex [N-Term];2xPhospho [S/T]	S8(Phospho): 100;S10(Phospho): 100	9	1.364	0.403784
[R].SLPSSPLLTHQSISVR.[L]	767994242	TANC2	26115	767994242 [84-99]	1xTMT6plex [N-Term];2xPhospho [S1(100);S/T]	S1(Phospho): 100;S4(Phospho): 50;S5(Phospho): 50	4	0.708	0.401199
[R].LIVENLSSR.[VC]	20127499;21361282	SRSF6|SRSF4	6431|6429	20127499 [112-120];21361282 [106-114]	1xPhospho [S];1xTMT6plex [N-Term]	S7(Phospho): 50;S8(Phospho): 50	2	1.956	0.400624
[K].FNDSEGDDTEETEDYR.[Q]	7661958	BCLAF1	9774	7661958 [394-409]	1xTMT6plex [N-Term];2xPhospho [S4(100);T/Y]	S4(Phospho): 100;T9(Phospho): 98.39	2	0.604	0.400237
[R].AAFTTPDHAPLSPQSSVASSGSEQTEEQGSSR.[N]	768009744	SAMD4B	55095	768009744 [260-291]	1xPhospho [S/T];1xTMT6plex [N-Term]	S22(Phospho): 23.41;T25(Phospho): 23.41;S30(Phospho): 23.41;S31(Phospho): 23.41	9	0.575	0.399329
[K].FNDSEGDDTEETEDYR.[Q]	7661958	BCLAF1	9774	7661958 [394-409]	1xPhospho [S4(100)];1xTMT6plex [N-Term]	S4(Phospho): 100	6	1.059	0.399264
[K].LEPVSPPSPPHTDPELELVPPR.[L]	331284178	NCOR2	9612	331284178 [145-166]	1xTMT6plex [N-Term];2xPhospho [S5(100);S/T]	S5(Phospho): 99.97;S8(Phospho): 50.02;T12(Phospho): 50.02	1	1.475	0.398321
[K].TEESPASDEAGEKEAK.[S]	48255933	HMGN1	3150	48255933 [83-98]	2xPhospho [S4(100);3xTMT6plex [N-Term;K13;K16];S7(100)]	S4(Phospho): 100;S7(Phospho): 100	3	1.416	0.395722
[R].RLPRSPSPYSR.[R]	1034657270	CDK13	8621	1034657270 [354-364]	1xTMT6plex [N-Term];2xPhospho [S5(100);S10(98.2)]	S5(Phospho): 100;S7(Phospho): 95.58	3	1.068	0.395451
[K].TIASDSEEEAGK.[E]	530369278	IWS1	55677	530369278 [440-451]	2xPhospho [S4(100);2xTMT6plex [N-Term;K12];S6(100)]	S4(Phospho): 100;S6(Phospho): 100	4	0.525	0.394055
[R].DRSPAAETPPLQR.[R]	530416759	EPS8L1	54869	530416759 [180-192]	1xTMT6plex [N-Term];2xPhospho [S3(100);T8(100)]	S3(Phospho): 100;T8(Phospho): 100	1	0.32	0.393414
[R].GLMAGGRPEGQYSEDEDTDTDEYKEAK.[A]	27883866	RIC8A	60626	27883866 [430-456]	1xOxidation [M3];2xPhospho [Y/S/T];3xTMT6plex [N-Term;K24;K27]	Y12(Phospho): 50.03;S13(Phospho): 50.03;T18(Phospho): 96.66	8	0.418	0.391643
[R].RAHSPASTLPNSPGSTFER.[K]	119829187	COL17A1	1308	119829187 [82-100]	1xTMT6plex [N-Term];2xPhospho [S4(100);S7(97.4)]	S4(Phospho): 100;S12(Phospho): 99.96	23	0.739	0.387377
[R].VANAYFLCLLILQLIPEISSLTWFTTIVPLVLVITMTAVK.[D]	767985358	ATP8B4	79895	767985358 [92-131]	1xCarbamidomethyl [C8];2xPhospho [T/Y/S];2xTMT6plex [N-Term;K40]	Y5(Phospho): 25;S19(Phospho): 25;S20(Phospho): 25;T22(Phospho): 25;T25(Phospho): 25;T26(Phospho): 25;T35(Phospho): 25;T37(Phospho): 25	10	0.724	0.386847
[R].KGPGQPSSPQRLDR.[L]	5174409	CD2BP2	10421	5174409 [188-201]	1xPhospho [S];2xTMT6plex [N-Term;K1]	S7(Phospho): 50;S8(Phospho): 50	5	1.338	0.385568
[K].ASASGSGAQVGGPISSGSSASSVTVTR.[S]	27436946	LMNA	4000	27436946 [598-624]	1xPhospho [S/T];1xTMT6plex [N-Term]	S21(Phospho): 95.31	61	1.446	0.385153
[R].SLSYSPVERR.[R]	118572613	SRRM2	23524	118572613 [2690-2699]	1xTMT6plex [N-Term];2xPhospho [Y/S]	S3(Phospho): 99.27;Y4(Phospho): 50.35;S5(Phospho): 50.35	2	2.751	0.384880
[R].DPAQPMSPGEATQSGARPADR.[Y]	157694494	MYBBP1A	10514	157694494 [5-25]	1xOxidation [M6];1xPhospho [S7(95.8)];1xTMT6plex [N-Term]	S7(Phospho): 95.75	1	0.333	0.384517
[R].AESPESSAIESTQSTPQK.[G]	530402180	PDS5B	23047	530402180 [1357-1374]	2xPhospho [S3(100);2xTMT6plex [N-Term;K18];S/T]	S3(Phospho): 99.96;S14(Phospho): 48.67;T15(Phospho): 48.67	4	0.639	0.383650
[K].SGTPPRQGSITSPQANEQSVTPQRR.[S]	118572613	SRRM2	23524	118572613 [846-870]	1xTMT6plex [N-Term];3xPhospho [T3(99.7);S19(99.7);T21(100)]	T3(Phospho): 99.4;S9(Phospho): 48.58;T11(Phospho): 48.58;T21(Phospho): 94.1	4	2.059	0.379525
[K].SSFFSDRGSGSR.[G]	87196351	DDX3X	1654	87196351 [82-93]	1xPhospho [S];1xTMT6plex [N-Term]	S9(Phospho): 49.5;S11(Phospho): 49.5	1	0.136	0.377350
[R].LVPYSCRENYSDSEEEDDDDVASSR.[Q]	7706607	LEMD3	23592	7706607 [249-273]	1xCarbamidomethyl [C6];1xTMT6plex [N-Term];2xPhospho [S/Y]	Y4(Phospho): 39.81;S5(Phospho): 39.81;Y10(Phospho): 39.81;S11(Phospho): 39.81;S13(Phospho): 39.81	1	4.91	0.373336
[R].RRGSDPASGEVEASQLR.[R]	548923663	TMEM40	55287	548923663 [150-166]	1xPhospho [S4(100)];1xTMT6plex [N-Term]	S4(Phospho): 100	3	1.119	0.372567
[K].VEAKEESEESDEDMGFGLFD.[-]	16933546	RPLP0	6175	16933546 [298-317]	1xOxidation [M14];2xPhospho [S7(100);2xTMT6plex [N-Term;K4];S10(100)]	S7(Phospho): 100;S10(Phospho): 100	1	0.22	0.372111
[K].SNSQSDSHDEEVSPTPPNPVVK.[A]	258613969	PRKAR1B	5575	258613969 [71-92]	2xPhospho [S/T];2xTMT6plex [N-Term;K22]	S3(Phospho): 32.88;S5(Phospho): 32.88;S7(Phospho): 32.88;S13(Phospho): 49.96;T15(Phospho): 49.96	7	0.472	0.370657
[K].FASDDEHDEHDENGATGPVKR.[A]	10835067	SSB	6741	10835067 [364-384]	1xPhospho [S3(100)];2xTMT6plex [N-Term;K20]	S3(Phospho): 100	3	0.682	0.370469
[R].VQEHEDSGDSEVENEAK.[G]	118344456	UTP18	51096	118344456 [115-131]	2xPhospho [S7(100);2xTMT6plex [N-Term;K17];S10(100)]	S7(Phospho): 100;S10(Phospho): 100	6	1.426	0.370038
[R].KDDDSDDESQSSHTGK.[K]	24308039	UFL1	23376	24308039 [454-469]	1xPhospho [S/T];3xTMT6plex [N-Term;K1;K16]	S5(Phospho): 100	6	1.349	0.369652
[K].ILEGDNGMDSDMEEEADDGSK.[M]	262527223	CAAP1	79886	262527223 [194-214]	1xPhospho [S10(100)];2xOxidation [M8;2xTMT6plex [N-Term;K21];M12]	S10(Phospho): 100	3	1.123	0.369017
[K].RPGTQGHLGPEKEEEESDGEPEDSSTS.[-]	134288890	DIS3L2	129563	134288890 [859-885]	1xPhospho [S/T];2xTMT6plex [N-Term;K12]	S17(Phospho): 47.65;S25(Phospho): 47.65	2	0.331	0.368324
[R].ELDPSLVSANDSPSGMQTR.[C]	530370575	RIF1	55183	530370575 [2150-2168]	1xOxidation [M16];1xPhospho [S/T];1xTMT6plex [N-Term]	S5(Phospho): 24.84;S8(Phospho): 24.84;S12(Phospho): 24.84;S14(Phospho): 24.84	3	0.768	0.367465
[K].RVIGQDHDFSESSEEEAPAEASSGALR.[S]	122939208	SP100	6672	122939208 [398-424]	1xTMT6plex [N-Term];2xPhospho [S10(97.6);S]	S10(Phospho): 97.62;S12(Phospho): 49;S13(Phospho): 49	3	1.238	0.367226
[R].LEGLGSSEADQDGLASTVR.[S]	19920317	CKAP4	10970	19920317 [455-473]	1xPhospho [S6(97.8)];1xTMT6plex [N-Term]	S6(Phospho): 97.81	4	0.762	0.364760
[K].FEEESKEPVADEEEEDSDDDVEPITEFR.[F]	4502891	CLNS1A	1207	4502891 [86-113]	1xPhospho [S17(100)];2xTMT6plex [N-Term;K6]	S17(Phospho): 100	4	0.459	0.361633
[K].TPTSSPASSPLVAK.[K]	57863269	RRP1B	23076	57863269 [728-741]	2xPhospho [S/T];2xTMT6plex [N-Term;K14]	S8(Phospho): 99.97;S9(Phospho): 100	9	1.356	0.360963
[K].SEVQQPVHPKPLSPDSRASSLSESSPPK.[A]	767974553	LIMA1	51474	767974553 [351-378]	3xPhospho [S];3xTMT6plex [N-Term;K10;K28]	S20(Phospho): 66.03;S22(Phospho): 66.03;S24(Phospho): 66.03;S25(Phospho): 66.03	4	0.564	0.359731

Patil et al,2020. Proteomic and phosphoproteomic profiling of shammah-induced signaling in oral keratinocytes

Supplementary Table S2: List of phosphopeptides identified and quantified in OKF6/TERT1-Shammah and OKF6/TERT1-Parental phosphoproteome using TMT/TiO_2-based quantitative phosphoproteomic approach

Peptide Sequence	Protein Accessions	Gene Symbol	Gene ID	Positions in Proteins	Modifications	PhosphoRS: Best Site Probabilities	# PSMs	Fold change	p-value
[K].GSETPGATPGSK.[I]	54112117	SF3B1	23451	54112117 [241-252]	2xPhospho [T8(99.9);2xTMT6plex [N-Term;K12];S/T]	T4(Phospho): 99.99;T8(Phospho): 100	7	1.139	0.358674
[R].GGGAGGNSDSDNLDSTDCNSESSSGGK.[S]	37620163	ANKHD1-EIF4EBP3	404734	37620163 [1584-1610]	1xCarbamidomethyl [C18];1xPhospho [S8(90.9)];2xTMT6plex [N-Term;K27]	S8(Phospho): 90.88	6	0.29	0.358032
[R].SFGTRPLSSGFSPEEAQQQDEEFEK.[K]	578821824	TNKS1BP1	85456	578821824 [976-1000]	1xPhospho [S/T];2xTMT6plex [N-Term;K25]	S1(Phospho): 24.83;T4(Phospho): 24.83;S8(Phospho): 24.83;S9(Phospho): 24.83	4	0.471	0.356940
[K].DDSHSAEDSEDEKEDHKNVR.[Q]	56118310	NUCKS1	64710	56118310 [71-90]	2xPhospho [S5(96.8);3xTMT6plex [N-Term;K13;K17];S9(99.9)]	S5(Phospho): 96.77;S9(Phospho): 99.87	1	0.436	0.356831
[K].GGSGSGPTIEEVD.[-]	167466173	HSPA1A	3303	167466173 [629-641]	1xPhospho [S/T];1xTMT6plex [N-Term]	S3(Phospho): 50;S5(Phospho): 50	2	1.843	0.356433
[R].IPMPPSSPQPR.[G]	215820635	PPP1R13L	10848	215820635 [352-362]	1xOxidation [M3];1xPhospho [S];1xTMT6plex [N-Term]	S6(Phospho): 50;S7(Phospho): 50	2	1.286	0.355845
[K].AEEDEILNRSPR.[N]	767938662	CANX	821	767938662 [628-639]	1xPhospho [S10(100)];1xTMT6plex [N-Term]	S10(Phospho): 100	18	1.285	0.355736
[R].RPSQEQSASASSGQPQAPLNR.[E]	150010552	FKBP15	23307	150010552 [954-974]	1xPhospho [S3(100)];1xTMT6plex [N-Term]	S3(Phospho): 100	12	0.661	0.355321
[K].AVSPPHLDGPPSPR.[S]	67089149	PML	5371	67089149 [516-529]	1xTMT6plex [N-Term];2xPhospho [S3(100);S12(100)]	S3(Phospho): 100;S12(Phospho): 100	23	0.937	0.354434
[K].RIDFTPVSPAPSPTR.[G]	768038902	FAM122B	159090	768038902 [131-145]	1xTMT6plex [N-Term];2xPhospho [S8(100);S12(98.4)]	S8(Phospho): 99.97;S12(Phospho): 98.38	9	0.77	0.354313
[R].ADHRSSPNVANQPPSPGGK.[S]	1034650321	AFDN	4301	1034650321 [1176-1194]	2xPhospho [S15(99.9);2xTMT6plex [N-Term;K19];S]	S5(Phospho): 98.5;S15(Phospho): 100	8	0.53	0.350976
[R].AEAESPGGASESDQDGGHESPPK.[K]	768009782	PNMA8A	55228	768009782 [324-346]	2xPhospho [S];2xTMT6plex [N-Term;K23]	S5(Phospho): 66.59;S10(Phospho): 66.59;S12(Phospho): 66.59	1	0.962	0.347354
[R].RLSLGQGDSTEAATEER.[G]	1034659998	FAM83H	286077	1034659998 [1202-1218]	1xPhospho [S3(100)];1xTMT6plex [N-Term]	S3(Phospho): 100	3	0.879	0.345822
[R].ASAVSELSPR.[E]	167234419	THRAP3	9967	167234419 [236-245]	1xPhospho [S8(100)];1xTMT6plex [N-Term]	S8(Phospho): 100	4	1.696	0.345698
[R].DALGDSLQVPVSPSSTTSSR.[C]	6005709	PALM2-AKAP2	445815	6005709 [372-391]	1xTMT6plex [N-Term];2xPhospho [S/T]	S12(Phospho): 97.29;S15(Phospho): 19.93;T16(Phospho): 19.93;T17(Phospho): 19.93;S18(Phospho): 19.93;S19(Phospho): 19.93	4	0.997	0.345009
[R].YSNQNLEDLMPSRPPSPR.[E]	578810809	FAT2	2196	578810809 [4243-4260]	1xOxidation [M10];1xPhospho [S/Y];1xTMT6plex [N-Term]	S12(Phospho): 49.25;S16(Phospho): 49.25	2	0.547	0.342747
[R].KQSQIQNQQGEDSGSDPEDTY.[-]	4557719	LIG1	3978	4557719 [899-919]	2xPhospho [S/T/Y];2xTMT6plex [N-Term;K1]	S13(Phospho): 66.35;S15(Phospho): 66.35;T20(Phospho): 65.5	2	0.449	0.342643
[R].HAYEGSSSGNSSPEYPR.[K]	119829187	COL17A1	1308	119829187 [107-123]	1xTMT6plex [N-Term];2xPhospho [S/Y]	S6(Phospho): 95.63;S12(Phospho): 99.97	33	0.672	0.341104
[R].LTPVSPESSSTEEK.[S]	4505571	SQSTM1	8878	4505571 [268-281]	1xPhospho [S5(98.4)];2xTMT6plex [N-Term;K14]	S5(Phospho): 99.99	3	0.866	0.341031
[R].GPSPAPASSPK.[R]	768010386	SCAF1	58506	768010386 [720-730]	2xPhospho [S3(100);2xTMT6plex [N-Term;K11];S]	S3(Phospho): 100;S9(Phospho): 99.97	7	0.939	0.339987
[R].SKETSSPGTDDVFTPAPSDSPSSQR.[I]	27777632	SLC9A1	6548	27777632 [766-790]	1xPhospho [S/T];2xTMT6plex [N-Term;K2]	S18(Phospho): 49.23;S20(Phospho): 49.23	11	1.94	0.337660
[R].VDHGAEIITQSPGRSSVASPR.[R]	1370477686	MAP2	4133	1370477686 [1860-1880]	1xTMT6plex [N-Term];3xPhospho [T9(96.3);S];S11(96.3)	T9(Phospho): 96.29;S11(Phospho): 96.29;S15(Phospho): 35.8;S16(Phospho): 35.8;S19(Phospho): 35.8	5	0.417	0.336961
[R].DVATSPISPTENNTTPPDALTR.[N]	767968303	PAK1	5058	767968303 [216-237]	1xPhospho [S/T];1xTMT6plex [N-Term]	T4(Phospho): 24.83;S5(Phospho): 24.83;S8(Phospho): 24.83;T10(Phospho): 24.83	12	0.78	0.336900
[R].DNLLDTYSADQGDSSEGGTLAR.[G]	145580631	QSOX2	169714	145580631 [565-586]	1xPhospho [T/Y/S];1xTMT6plex [N-Term]	S14(Phospho): 48.83;S15(Phospho): 48.83	16	0.561	0.331401
[K].LSVPTSDEEDEVPAPKPR.[G]	69354671	ABCF1	23	69354671 [104-121]	2xPhospho [T5(99.9);2xTMT6plex [N-Term;K16];S6(100)]	T5(Phospho): 99.95;S6(Phospho): 99.95	8	0.649	0.329018
[K].GLPSPYNMSSAPGSR.[S]	58530840	DSP	1832	58530840 [2812-2826]	1xOxidation [M8];1xTMT6plex [N-Term];2xPhospho [S14(95.1);Y/S]	S10(Phospho): 98.48;S14(Phospho): 100	13	0.76	0.327267
[K].GLPSPYNMSSAPGSRSGSR.[S]	58530840	DSP	1832	58530840 [2812-2830]	1xOxidation [M8];1xTMT6plex [N-Term];2xPhospho [S14(94.9);S16(94.9)]	S9(Phospho): 49.42;S10(Phospho): 49.42;S14(Phospho): 49.42;S16(Phospho): 49.42	3	0.414	0.324519
[R].TPHSPPLAK.[E]	157738639	TNS4	84951	157738639 [347-355]	1xPhospho [S4(99.2)];2xTMT6plex [N-Term;K9]	S4(Phospho): 99.15	1	0.511	0.324012
[K].GNVFSSPTAAGTPNKETAGLK.[V]	1034656986;1370511010	CALD1|CALD1	800|800	1034656986 [464-484];1370511010 [490-510]	2xPhospho [T8(92.6);3xTMT6plex [N-Term;K15;K21];T12(95.9)]	S6(Phospho): 97.23;T8(Phospho): 94.7	2	0.27	0.323762
[R].LASPSGSTSSGLEVVAPEGTSAPGGGPGTLDDSATICR.[V]	1370474190	TRIM28	10155	1370474190 [592-629]	1xCarbamidomethyl [C37];1xTMT6plex [N-Term];2xPhospho [S3(96.7);S/T]	S3(Phospho): 96.74;S7(Phospho): 93.47	2	1.002	0.323056
[K].GIAPASPMLGNASNPNK.[A]	731441413	MARK3	4140	731441413 [464-480]	1xOxidation [M8];1xPhospho [S6(100)];2xTMT6plex [N-Term;K17]	S6(Phospho): 100	1	0.394	0.320716
[R].VQAYEEPSVASSPNGK.[E]	225007648	POP1	10940	225007648 [719-734]	1xPhospho [S/Y];2xTMT6plex [N-Term;K16]	S8(Phospho): 33.33;S11(Phospho): 33.33;S12(Phospho): 33.33	3	0.799	0.319831
[K].DFDIAEQNESSDEESLRK.[E]	21361912	DNAJC1	64215	21361912 [470-487]	2xPhospho [S10(100);2xTMT6plex [N-Term;K18];S]	S10(Phospho): 99.96;S11(Phospho): 99.96	3	0.845	0.319206
[R].RNSFTPLSSSNTIR.[R]	1370453465	PFKFB2	5208	1370453465 [464-477]	1xPhospho [S3(98.5)];1xTMT6plex [N-Term]	S3(Phospho): 97.78	1	0.566	0.319172
[R].MQNNSSPSISPNTSFTSDGSPSPLGGIK.[R]	964750564	XRN2	22803	964750564 [544-571]	1xOxidation [M1];1xPhospho [S/T];2xTMT6plex [N-Term;K28]	S5(Phospho): 11.07;S6(Phospho): 11.07;S8(Phospho): 11.07;S10(Phospho): 11.07;T13(Phospho): 11.07;S14(Phospho): 11.07;T16(Phospho): 11.07;S17(Phospho): 11.07;S20(Phospho): 11.07	9	0.771	0.315971
[K].IPSKEEEADMSSPTQR.[T]	20070205	LAD1	3898	20070205 [345-360]	1xOxidation [M10];1xPhospho [S12(96.1)];2xTMT6plex [N-Term;K4]	S12(Phospho): 98.4	11	1.904	0.314469
[R].VHASRPASLDSGR.[T]	530367810	CCDC88A	55704	530367810 [1580-1592]	1xTMT6plex [N-Term];2xPhospho [S4(100);S8(97.2)]	S4(Phospho): 100;S8(Phospho): 99.97	2	0.421	0.313375
[K].AAEEQGDDQDSEKSKPAGSDGER.[R]	118601081	HNRNPUL2	221092	118601081 [175-197]	1xPhospho [S];3xTMT6plex [N-Term;K13;K15]	S14(Phospho): 49.47;S19(Phospho): 49.47	5	1.688	0.311252
[K].LTVSDGESGEEK.[K]	530788276	ATRX	546	530788276 [1345-1356]	2xPhospho [S4(98.8);2xTMT6plex [N-Term;K12];S8(100)]	S4(Phospho): 98.84;S8(Phospho): 100	5	2.1	0.305086
[R].SRGSGGLGGACGGAGFGSR.[S]	119703753;5031839	KRT6B|KRT6A	3854|3853	119703753 [41-59];5031839 [41-59]	1xCarbamidomethyl [C11];1xPhospho [S4(98.1)];1xTMT6plex [N-Term]	S4(Phospho): 99.99	4	1.995	0.304890
[K].TGVTSTSDSEEEGDDQEGEK.[K]	190014588	PSIP1	11168	190014588 [267-286]	2xPhospho [T/S];2xTMT6plex [N-Term;K20]	T6(Phospho): 65.54;S7(Phospho): 65.54;S9(Phospho): 65.54	1	0.668	0.304693
[R].LPSGSGAASPTGSAVDIR.[A]	1079280806	AHNAK	79026	1079280806 [208-225]	1xTMT6plex [N-Term];2xPhospho [S/T]	S5(Phospho): 98.34;S9(Phospho): 100	19	0.775	0.303619
[K].ALSSLHGDDQDSEDEVLTIPEVK.[V]	119964726	IGF2R	3482	119964726 [2398-2420]	1xPhospho [S12(89.1)];2xTMT6plex [N-Term;K23]	S12(Phospho): 98.39	1	2.964	0.302029
[R].LQDSSSEEEDVTEETDHR.[N]	578817611	NOL8	55035	578817611 [1079-1096]	1xTMT6plex [N-Term];3xPhospho [S6(91.7);S/T]	S4(Phospho): 100;S5(Phospho): 100;S6(Phospho): 100	2	0.558	0.300595
[K].AASADSTTEGTPADGFTVLSTK.[S]	767936777	H2AFY	9555	767936777 [168-189]	2xPhospho [T21(92.1);2xTMT6plex [N-Term;K22];T/S]	S6(Phospho): 49.53;T7(Phospho): 49.53;T11(Phospho): 99.88	13	1.493	0.298766
[K].EVAENQQNQSSDPEEEKGSQPPAAESQSSLRR.[Q]	1370507498	EPB41L2	2037	1370507498 [29-61]	1xPhospho [S];2xTMT6plex [N-Term;K17]	S27(Phospho): 33.33;S29(Phospho): 33.33;S30(Phospho): 33.33	9	0.399	0.298527
[K].LLTMSRQASKTSGGGSQGFSGR.[S]	1370461858	KRT3	3850	1370461858 [86-107]	1xOxidation [M4];2xPhospho [S12(85.4);2xTMT6plex [N-Term;K10];S/T]	T3(Phospho): 24.76;S5(Phospho): 24.76;S9(Phospho): 24.76;T11(Phospho): 24.76;S16(Phospho): 48.75;S20(Phospho): 48.75	2	0.226	0.296197
[K].TGNESGSNLSDSGSVK.[R]	116008447	CLIP2	7461	116008447 [198-213]	2xPhospho [S10(98.4);2xTMT6plex [N-Term;K16];S/T]	S5(Phospho): 50;S7(Phospho): 50;S10(Phospho): 98.4	2	0.326	0.295100
[R].DLEPGEVPSDSDEDGEHK.[S]	14790190	SPEN	23013	14790190 [1372-1389]	2xPhospho [S9(100);2xTMT6plex [N-Term;K18];S11(100)]	S9(Phospho): 100;S11(Phospho): 100	3	0.771	0.290711
[K].QVAEQGGDLSPAANR.[S]	126362971	STK10	6793	126362971 [429-443]	1xPhospho [S10(100)];1xTMT6plex [N-Term]	S10(Phospho): 100	2	0.763	0.290473
[K].SEDSDVELSD.[-]	530363589	ZMYM4	9202	530363589 [1542-1551]	1xTMT6plex [N-Term];2xPhospho [S4(98.6);S9(100)]	S4(Phospho): 98.56;S9(Phospho): 100	1	0.987	0.288540
[R].LQQGAGLESPQGQPEPGAASPQR.[Q]	13129104	CCDC86	79080	13129104 [72-94]	1xTMT6plex [N-Term];2xPhospho [S9(100);S20(100)]	S9(Phospho): 100;S20(Phospho): 100	3	0.827	0.288338
[K].GNAEGSSDEEGKLVIDEPAK.[E]	984290516	HDGF	3068	984290516 [150-169]	2xPhospho [S6(100);3xTMT6plex [N-Term;K12;K20];S7(100)]	S6(Phospho): 100;S7(Phospho): 100	3	0.347	0.286686
[R].YQDEVFGGFVTEPQEESEEEVEEPEER.[Q]	38327552	G3BP1	10146	38327552 [133-159]	1xPhospho [T/S/Y];1xTMT6plex [N-Term]	T11(Phospho): 50;S17(Phospho): 50	4	0.332	0.286186
[R].SLPTTVPESPNYR.[N]	530380464	LARP1	23367	530380464 [766-778]	1xTMT6plex [N-Term];2xPhospho [S1(100);S9(94.6)]	S1(Phospho): 100;S9(Phospho): 99.98	6	0.443	0.285074
[R].SLTRSPPAIR.[R]	118572613	SRRM2	23524	118572613 [2067-2076]	1xTMT6plex [N-Term];2xPhospho [T/S]	T3(Phospho): 99.98;S5(Phospho): 100	4	1.5	0.283369
[K].AQEAEAQSEDDDEDTEEEQGEEKEK.[G]	1214884389	NOM1	64434	1214884389 [273-297]	2xPhospho [S8(100);3xTMT6plex [N-Term;K23;K25];T15(100)]	S8(Phospho): 100;T15(Phospho): 100	9	1.384	0.282337
[R].RGSQKSTDSPGADAELPESAAR.[D]	530383409	CRYBG1	202	530383409 [422-443]	2xPhospho [S3(97.3);2xTMT6plex [N-Term;K5];S/T]	S3(Phospho): 97.32;S6(Phospho): 34.23;T7(Phospho): 34.23;S9(Phospho): 34.23	2	0.375	0.282118
[R].TSSEDNLYLAVLR.[A]	530389151	PLEC	5339	530389151 [19-31]	1xPhospho [T/S/Y];1xTMT6plex [N-Term]	S2(Phospho): 49.66;S3(Phospho): 49.66	4	1.476	0.281758
[K].DLFDLNSSEEDDTEGFSER.[G]	157694511	NOC2L	26155	157694511 [666-684]	1xTMT6plex [N-Term];2xPhospho [S/T]	S7(Phospho): 100;S8(Phospho): 100	16	0.816	0.281584
[R].KQAREESEESEAEPVQR.[T]	21396500	HIRIP3	8479	21396500 [190-206]	2xPhospho [S7(100);2xTMT6plex [N-Term;K1];S10(100)]	S7(Phospho): 100;S10(Phospho): 100	3	0.371	0.281395
[K].TQTPPVSPAPQPTEER.[L]	296080746	CTTN	2017	296080746 [362-377]	1xTMT6plex [N-Term];2xPhospho [T3(100);S7(100)]	T3(Phospho): 98.97;S7(Phospho): 100	8	0.727	0.279845
[R].ERSPSPLRGNVVPSPLPTR.[R]	1034594161	CARHSP1	23589	1034594161 [49-67]	1xTMT6plex [N-Term];2xPhospho [S3(99.3);S5(90.2)]	S3(Phospho): 100;S5(Phospho): 100	1	0.182	0.279809
[K].SRVSVSPGRTSGK.[V]	1034554585	SRRM1	10250	1034554585 [451-463]	2xPhospho [S4(100);2xTMT6plex [N-Term;K13];S6(100)]	S4(Phospho): 99.98;S6(Phospho): 100	4	3.66	0.279213
[K].KLSVPTSDEEDEVPAPKPR.[G]	69354671	ABCF1	23	69354671 [103-121]	2xPhospho [S3(99.9);3xTMT6plex [N-Term;K1;K17];T/S]	T6(Phospho): 100;S7(Phospho): 100	6	0.58	0.278695
[K].ASLGSLEGEAEAEASSPK.[G]	1079280806	AHNAK	79026	1079280806 [5748-5765]	2xPhospho [S5(98.2);2xTMT6plex [N-Term;K18];S]	S5(Phospho): 100;S16(Phospho): 98.58	8	0.739	0.275240
[R].LTRYSQGDDDGSSSSGGSSVAGSQSTLFK.[D]	1370482149	SUN2	25777	1370482149 [43-71]	1xPhospho [T/Y/S];2xTMT6plex [N-Term;K29]	T2(Phospho): 33.22;Y4(Phospho): 33.22;S5(Phospho): 33.22	7	5.484	0.272230
[K].LQEEGGGSDEEETGSPSEDGMQSAR.[T]	5902034	PWP1	11137	5902034 [43-67]	1xOxidation [M21];1xPhospho [S8(99.9)];1xTMT6plex [N-Term]	S8(Phospho): 99.86	2	0.691	0.271713
[K].LEEPPELNRQSPNPR.[R]	116734704	IRF2BP2	359948	116734704 [165-179]	1xPhospho [S11(100)];1xTMT6plex [N-Term]	S11(Phospho): 100	10	1.168	0.271665
[R].TPEELDDSDFETEDFDVR.[S]	1022433203	CTNNA1	1495	1022433203 [634-651]	1xPhospho [S8(98.8)];1xTMT6plex [N-Term]	S8(Phospho): 98.81	1	1.288	0.271442
[K].NLNNSNLFSPVNR.[D]	1435761100	NUP98	4928	1435761100 [587-599]	1xPhospho [S9(100)];1xTMT6plex [N-Term]	S9(Phospho): 100	6	1.133	0.270464
[K].VATLNSEEESDPPTYK.[D]	1370477456	HDLBP	3069	1370477456 [26-41]	1xPhospho [S6(100)];2xTMT6plex [N-Term;K16]	S6(Phospho): 99.99	4	1.059	0.268195
[K].LNQSSPDNVTDTK.[G]	663071131	CAMSAP2	23271	663071131 [595-607]	1xPhospho [S/T];2xTMT6plex [N-Term;K13]	S5(Phospho): 99.06	7	0.474	0.267545
[R].ALPLPQSSTC.[-]	1034590453	IGF1R	3480	1034590453 [1383-1392]	1xCarbamidomethyl [C10];1xPhospho [T9(98)];1xTMT6plex [N-Term]	T9(Phospho): 98.01	6	1.286	0.266873
[K].LPQPPEGQCYSN.[-]	40806190	TMEM189-UBE2V1	387522	40806190 [359-370]	1xCarbamidomethyl [C9];1xPhospho [S/Y];1xTMT6plex [N-Term]	S11(Phospho): 98.68	3	0.689	0.266013
[K].FNNDSHSPK.[V]	26986534	ARHGAP12	94134	26986534 [159-167]	1xPhospho [S7(100)];2xTMT6plex [N-Term;K9]	S7(Phospho): 100	1	0.397	0.264730
[K].STSAPQMSPGSSDNQSSSPQPAQQK.[L]	530365733	UBAP2L	9898	530365733 [471-495]	1xOxidation [M7];1xPhospho [S8(97.2)];2xTMT6plex [N-Term;K25]	S8(Phospho): 97.23	89	0.464	0.263986
[R].IFAISQQQQQQSK.[S]	1034650032	DSE	29940	1034650032 [812-824]	1xPhospho [S5(100)];2xTMT6plex [N-Term;K13]	S5(Phospho): 100	4	2.111	0.261934
[K].MPAPEPGAAPSNEEDDSDDDDVLAPSGATAAGAGDEGDGQTTGST.[-]	6466466	RANBP3	8498	6466466 [523-567]	1xOxidation [M1];1xPhospho [S/T];1xTMT6plex [N-Term]	S11(Phospho): 14.16;S17(Phospho): 14.16;S26(Phospho): 14.16;T29(Phospho): 14.16;T41(Phospho): 14.16;T42(Phospho): 14.16;S44(Phospho): 14.16	17	0.688	0.261588
[K].GGVTGSPEASISGSK.[G]	1079280806	AHNAK	79026	1079280806 [5726-5740]	1xPhospho [S6(99)];2xTMT6plex [N-Term;K15]	S6(Phospho): 99.1	5	0.668	0.261354
[R].SRTASGSSVTSLDGTR.[S]	767952381	NDRG1	10397	767952381 [343-358]	1xTMT6plex [N-Term];2xPhospho [T/S]	T3(Phospho): 100;S5(Phospho): 100	12	0.707	0.258912
[K].YVDAETSL.[-]	1007367579	SLC4A7	9497	1007367579 [1252-1259]	1xPhospho [S7(99)];1xTMT6plex [N-Term]	S7(Phospho): 99.12	3	1.254	0.258662
[R].SRSRDSGDENEPIQER.[F]	767931464	PALLD	23022	767931464 [1201-1216]	1xTMT6plex [N-Term];3xPhospho [S1(100);S3(100);S6(100)]	S1(Phospho): 100;S3(Phospho): 100;S6(Phospho): 100	2	0.195	0.258020
[R].LSPSPTSQR.[S]	27436946	LMNA	4000	27436946 [389-397]	1xPhospho [S4(100)];1xTMT6plex [N-Term]	S4(Phospho): 99.99	5	2.564	0.257917
[R].SVSPPPKR.[A]	20127499	SRSF6	6431	20127499 [314-321]	2xPhospho [S1(100);2xTMT6plex [N-Term;K7];S3(100)]	S1(Phospho): 100;S3(Phospho): 100	8	0.776	0.257822
[R].FSTYSQSPPDTPSLR.[E]	767990521	RIPOR1	79567	767990521 [370-384]	1xPhospho [S/Y/T];1xTMT6plex [N-Term]	S5(Phospho): 48.8;S7(Phospho): 48.8	2	0.783	0.256698
[R].GPAGEAGASPPVRR.[V]	5803177	SNTB2	6645	5803177 [102-115]	1xPhospho [S9(100)];1xTMT6plex [N-Term]	S9(Phospho): 100	3	1.436	0.255029
[K].AEVPGATGGDSPHLQPAEPPGEPR.[R]	33620747	MYEF2	50804	33620747 [7-30]	1xPhospho [T7(96.6)];1xTMT6plex [N-Term]	T7(Phospho): 96.65	1	1.372	0.254499
[R].RASPGLSMPSSSPPIK.[K]	156151371	KLF3	51274	156151371 [90-105]	1xOxidation [M8];2xPhospho [S];2xTMT6plex [N-Term;K16]	S3(Phospho): 100;S7(Phospho): 98.02	7	0.844	0.253828
[R].RSDSASSEPVGIYQGFEK.[K]	1237937706	PRKCD	5580	1237937706 [320-337]	2xPhospho [Y13(99.9);2xTMT6plex [N-Term;K18];S]	S4(Phospho): 33.02;S6(Phospho): 33.02;S7(Phospho): 33.02;Y13(Phospho): 99.87	1	0.309	0.253409
[R].GSSRQGSPGSVSK.[Q]	767944776	IGF2BP3	10643	767944776 [183-195]	2xPhospho [S];2xTMT6plex [N-Term;K13]	S2(Phospho): 50;S3(Phospho): 50;S7(Phospho): 100	3	1.752	0.253354
[R].EFDELNPSAQR.[D]	24638454	ATP2A2	488	24638454 [656-666]	1xPhospho [S8(100)];1xTMT6plex [N-Term]	S8(Phospho): 100	2	1.616	0.252023
[R].APQTSSSPPPVRR.[G]	1034554585	SRRM1	10250	1034554585 [729-741]	1xTMT6plex [N-Term];2xPhospho [S/T]	T4(Phospho): 97.76;S5(Phospho): 93.62	8	2.304	0.251211
[K].AAAGPLDMSLPSTPDIK.[I]	530399638	ATF7	11016	530399638 [89-105]	1xOxidation [M8];2xPhospho [S/T];2xTMT6plex [N-Term;K17]	S12(Phospho): 98.82;T13(Phospho): 100	4	1.257	0.250835
[R].AHSPASTLPNSPGSTFER.[K]	119829187	COL17A1	1308	119829187 [83-100]	1xTMT6plex [N-Term];2xPhospho [S3(100);S11(96.8)]	S3(Phospho): 100;S11(Phospho): 100	58	0.857	0.250162
[R].ASEDTTSGSPPK.[K]	219283152	API5	8539	219283152 [456-467]	1xPhospho [T/S];2xTMT6plex [N-Term;K12]	S9(Phospho): 100	8	0.675	0.247947
[K].AVPIAVADEGESESEDDDLKPR.[G]	767956109	USP20	10868	767956109 [121-142]	2xPhospho [S12(100);2xTMT6plex [N-Term;K20];S14(100)]	S12(Phospho): 100;S14(Phospho): 100	3	1.349	0.247718
[K].GVDFESSEDDDDDPFMNTSSLR.[R]	1034573783	MRE11	4361	1034573783 [683-704]	1xOxidation [M16];1xTMT6plex [N-Term];2xPhospho [S6(98.9);S/T]	S6(Phospho): 98.91;S7(Phospho): 98.91	3	0.594	0.245116
[R].ADAPDAGAQSDSELPSYHQNDVSLDR.[G]	578819967	GBF1	8729	578819967 [1323-1348]	1xPhospho [S/Y];1xTMT6plex [N-Term]	S10(Phospho): 50;S12(Phospho): 50	19	0.621	0.245033
[R].DSAIPVESDTDDEGAPR.[I]	995970200	NMD3	51068	995970200 [461-477]	1xTMT6plex [N-Term];2xPhospho [S8(100);T10(100)]	S8(Phospho): 100;T10(Phospho): 100	6	0.36	0.244857
[R].SRSDIDVNAAASAK.[S]	1034612655	CLASP1	23332	1034612655 [598-611]	1xPhospho [S];2xTMT6plex [N-Term;K14]	S1(Phospho): 50;S3(Phospho): 50	6	0.812	0.242361
[R].RPPSPDVIVLSDNEQPSSPR.[V]	1034608303	GATAD2A	54815	1034608303 [116-135]	1xTMT6plex [N-Term];3xPhospho [S4(100);S]	S4(Phospho): 99.9;S11(Phospho): 66.7;S17(Phospho): 66.7;S18(Phospho): 66.7	2	0.893	0.241557
[K].SLDSDESEDEEDDYQQK.[R]	7657441	PDAP1	11333	7657441 [57-73]	2xPhospho [S/Y];2xTMT6plex [N-Term;K17]	S4(Phospho): 99.98;S7(Phospho): 100	11	0.334	0.241548
[R].TQPSSGVDSAVGTLPATSPQSTSVQAK.[G]	530380665	TCOF1	6949	530380665 [1131-1157]	1xPhospho [S/T];2xTMT6plex [N-Term;K27]	T13(Phospho): 33.33;T17(Phospho): 33.33;S18(Phospho): 33.33	15	0.764	0.240837
[R].VKEEPPSPPQSPR.[V]	530389521	HSF1	3297	530389521 [297-309]	2xPhospho [S7(100);2xTMT6plex [N-Term;K2];S11(100)]	S7(Phospho): 100;S11(Phospho): 100	3	0.606	0.240412
[R].AQPGSPESSGQPK.[G]	767940349	TRERF1	55809	767940349 [487-499]	1xPhospho [S5(100)];2xTMT6plex [N-Term;K13]	S5(Phospho): 100	6	1.148	0.237967
[R].GSLLLDGAGAGGAGGSRPCSNR.[T]	14249622	AJUBA	84962	14249622 [140-161]	1xCarbamidomethyl [C19];1xPhospho [S16(97.8)];1xTMT6plex [N-Term]	S16(Phospho): 97.83	1	0.288	0.237467
[K].FGSTGSTPPVSPTPSER.[S]	970259824	MRTFA	57591	970259824 [479-495]	1xTMT6plex [N-Term];2xPhospho [T/S]	S6(Phospho): 98.62;S11(Phospho): 99.98	5	0.536	0.235375
[R].GRLTPSPDIIVLSDNEASSPR.[S]	530365128	GATAD2B	57459	530365128 [117-137]	1xTMT6plex [N-Term];2xPhospho [T4(99.9);S]	T4(Phospho): 99.86;S13(Phospho): 32.96;S18(Phospho): 32.96;S19(Phospho): 32.96	1	1.764	0.233108
[K].SISSSNPDLAVAPGSVDDEVSR.[I]	530415042	DOCK6	57572	530415042 [880-901]	1xPhospho [S];1xTMT6plex [N-Term]	S4(Phospho): 49.42;S5(Phospho): 49.42	4	0.738	0.233038
[R].NFSDNQLQEGK.[N]	469608410	TAGLN2	8407	469608410 [182-192]	1xPhospho [S3(100)];2xTMT6plex [N-Term;K11]	S3(Phospho): 100	3	1.197	0.230723
[R].LFSQGQDVSNK.[V]	1034650321	AFDN	4301	1034650321 [1805-1815]	1xPhospho [S3(100)];2xTMT6plex [N-Term;K11]	S3(Phospho): 100	4	0.572	0.230346
[K].DDSHSAEDSEDEKEDHK.[N]	56118310	NUCKS1	64710	56118310 [71-87]	2xPhospho [S5(100);3xTMT6plex [N-Term;K13;K17];S9(100)]	S5(Phospho): 97.87;S9(Phospho): 100	3	0.168	0.230302
[R].RSSLSPPSSAYER.[G]	767923054	SETD2	29072	767923054 [2086-2098]	1xTMT6plex [N-Term];2xPhospho [S5(100);S/Y]	S3(Phospho): 99.97;S5(Phospho): 100	3	1.198	0.229381
[K].SQDSYPGSPSLSPR.[H]	530424156	RANBP10	57610	530424156 [358-371]	1xTMT6plex [N-Term];2xPhospho [Y5(95.7);S]	S8(Phospho): 96.26;S12(Phospho): 98.09	5	0.69	0.229318
[R].YSSSGSPANSFHFK.[E]	1034594464	EEF2K	29904	1034594464 [69-82]	2xPhospho [Y1(95.6);2xTMT6plex [N-Term;K14];S]	Y1(Phospho): 95.59;S2(Phospho): 20.88;S3(Phospho): 20.88;S4(Phospho): 20.88;S6(Phospho): 20.88;S10(Phospho): 20.88	5	0.363	0.227476
[K].ALSSAVQASPTSPGGSPSSPSSGQR.[S]	767970167	KMT2A	4297	767970167 [3536-3560]	1xTMT6plex [N-Term];2xPhospho [S16(97.2);S/T]	T11(Phospho): 49.48;S12(Phospho): 49.48;S19(Phospho): 97.93	4	0.751	0.227197
[R].KSVAAEGALLPQTPPSPR.[N]	578801173	RALGPS2	55103	578801173 [314-331]	2xPhospho [T13(100);2xTMT6plex [N-Term;K1];S16(100)]	T13(Phospho): 100;S16(Phospho): 100	4	0.578	0.223501
[K].DLPSPDSR.[N]	578837375	TCF20	6942	578837375 [1319-1326]	1xPhospho [S4(100)];1xTMT6plex [N-Term]	S4(Phospho): 100	3	1.334	0.222150
[K].EQSEVSVSPR.[A]	530401729	RSRC2	65117	530401729 [25-34]	1xTMT6plex [N-Term];2xPhospho [S6(100);S8(100)]	S6(Phospho): 100;S8(Phospho): 100	3	1.25	0.217659
[R].AQSPGSPLHAR.[G]	4502175	SHROOM2	357	4502175 [1034-1044]	1xTMT6plex [N-Term];2xPhospho [S3(100);S6(100)]	S3(Phospho): 100;S6(Phospho): 100	3	0.717	0.217030
[R].VTRSPPTQVAISSDSAR.[K]	155029552	CCDC9B	388115	155029552 [198-214]	1xPhospho [T/S];1xTMT6plex [N-Term]	T2(Phospho): 50;S4(Phospho): 50	2	1.076	0.216729
[R].GLSGQRTPSPPR.[R]	1370455393	CROCC	9696	1370455393 [620-631]	1xTMT6plex [N-Term];2xPhospho [S3(99.3);T7(98)]	T7(Phospho): 98.01;S9(Phospho): 98.01	3	0.702	0.216560
[R].TNPEDIYPSNPTDDDVSSGSSSER.[S]	530396004	CD44	960	530396004 [163-186]	1xPhospho [S/Y/T];1xTMT6plex [N-Term]	S21(Phospho): 98.33	41	1.761	0.216103
[R].ILENGVNVTVSQMTSVLNGR.[T]	1034673322	TENM1	10178	1034673322 [2636-2655]	1xTMT6plex [N-Term];3xPhospho [S11(96.7);S15(96.7)];T14(96.7)	S11(Phospho): 96.55;T14(Phospho): 96.55;S15(Phospho): 96.55	6	0.248	0.213845
[R].ESPRPLQLPGAEGPAISDGEEGGGEPGAGGGAAGAAGAGR.[R]	530380464	LARP1	23367	530380464 [74-113]	1xPhospho [S];1xTMT6plex [N-Term]	S2(Phospho): 50;S17(Phospho): 50	1	0.299	0.211949
[R].LPDSDDDEDEETAIQR.[V]	116268103	ZFYVE19	84936	116268103 [351-366]	1xPhospho [S4(100)];1xTMT6plex [N-Term]	S4(Phospho): 100	4	0.433	0.211274
[R].IHIDPEIQDGSPTTSR.[R]	19923181	PDLIM4	8572	19923181 [102-117]	1xPhospho [S11(100)];1xTMT6plex [N-Term]	S11(Phospho): 99.99	20	0.751	0.206538
[R].GVGSGPHPPDTQQPSPSK.[A]	767945696	TNS3	64759	767945696 [762-779]	1xPhospho [S15(98.3)];2xTMT6plex [N-Term;K18]	S15(Phospho): 98.31	2	0.324	0.205812
[K].APVPGTPDSLSSGSSR.[D]	7305503	STOML2	30968	7305503 [322-337]	1xTMT6plex [N-Term];2xPhospho [T6(99.9);S]	T6(Phospho): 94.5;S9(Phospho): 21.1;S11(Phospho): 21.1;S12(Phospho): 21.1;S14(Phospho): 21.1;S15(Phospho): 21.1	1	0.639	0.205036
[R].SRDATPPVSPINMEDQER.[I]	4504809	JUNB	3726	4504809 [251-268]	1xOxidation [M13];1xTMT6plex [N-Term];2xPhospho [T5(100);S9(100)]	T5(Phospho): 100;S9(Phospho): 100	3	1.73	0.204916
[K].LASDDRPSPPR.[G]	1370525026	RBM10	8241	1370525026 [815-825]	1xPhospho [S8(100)];1xTMT6plex [N-Term]	S8(Phospho): 100	3	1.232	0.204412
[R].RSLGSSPCPK.[Q]	118572613	SRRM2	23524	118572613 [777-787]	1xCarbamidomethyl [C9];2xPhospho [S6(100);2xTMT6plex [N-Term;K11];S7(100)]	S6(Phospho): 99.99;S7(Phospho): 99.99	3	1.658	0.204147
[R].TPESQPDTPPGTPLVSQDEK.[R]	31543419	PNKP	11284	31543419 [111-130]	2xPhospho [S4(97.6);2xTMT6plex [N-Term;K20];T/S]	S4(Phospho): 97.29;T8(Phospho): 97.37	6	1.34	0.203087
[K].TIQNSSVSPTSSSSSSSTGETQTQSSSR.[L]	32698730	NUFIP2	57532	32698730 [369-397]	1xPhospho [S/T];1xTMT6plex [N-Term]	S8(Phospho): 69.79	13	1.58	0.203041
[K].AVIVSSPK.[A]	1034613097	ZNF638	27332	1034613097 [1502-1509]	1xPhospho [S];2xTMT6plex [N-Term;K8]	S6(Phospho): 99.06	5	1.192	0.202310
[R].LVEPHSPSPSSK.[F]	71725360	ZNF609	23060	71725360 [571-582]	2xPhospho [S6(100);2xTMT6plex [N-Term;K12];S8(100)]	S6(Phospho): 100;S8(Phospho): 100	10	1.395	0.202289
[K].SIGSPESTPK.[N]	1370455125	NAV1	89796	1370455125 [780-789]	1xPhospho [S/T];2xTMT6plex [N-Term;K10]	S4(Phospho): 49.74;T8(Phospho): 49.74	3	0.702	0.200094
[K].TDNSVASSPSSAISTATPSPK.[S]	768033368	KDM6A	7403	768033368 [863-883]	2xPhospho [S8(86.8);2xTMT6plex [N-Term;K21];S19(99.5)]	S7(Phospho): 49.75;S8(Phospho): 49.75;S19(Phospho): 84.67	4	1.265	0.198554
[K].GVDFESSEDDDDDPFMNTSSLRR.[N]	1034573783	MRE11	4361	1034573783 [683-705]	1xOxidation [M16];1xTMT6plex [N-Term];2xPhospho [S6(96.8);S7(96.8)]	S6(Phospho): 96.82;S7(Phospho): 96.82	3	1.165	0.197901
[R].GDEASEEGQNGSSPK.[S]	530375995	ADD1	118	530375995 [453-467]	1xPhospho [S];2xTMT6plex [N-Term;K15]	S12(Phospho): 49.64;S13(Phospho): 49.64	4	0.468	0.197831
[K].TVDSQGPTPVCTPTFLER.[R]	222352111	NIFK	84365	222352111 [227-244]	1xCarbamidomethyl [C11];1xTMT6plex [N-Term];2xPhospho [T8(100);T12(98.1)]	T8(Phospho): 100;T12(Phospho): 98.08	2	1.044	0.197482
[R].ISIGGGSCAISGGYGSR.[A]	119703753;5031839	KRT6B|KRT6A	3854|3853	119703753 [70-86];5031839 [70-86]	1xCarbamidomethyl [C8];1xPhospho [S/Y];1xTMT6plex [N-Term]	Y14(Phospho): 49.8;S16(Phospho): 49.8	17	1.94	0.196225
[R].STPFIVPSSPTEQEGR.[Q]	213972636	TP53BP1	7158	213972636 [377-392]	1xPhospho [S/T];1xTMT6plex [N-Term]	S9(Phospho): 99.99	12	1.709	0.196100
[K].AAAAAPASEDEDDEDDEDDEDDDDDEEDDSEEEAMETTPAK.[G]	55956788	NCL	4691	55956788 [177-217]	1xOxidation [M35];1xPhospho [S/T];2xTMT6plex [N-Term;K41]	S8(Phospho): 49.95;S30(Phospho): 49.95	24	2.647	0.196086
[R].KALVVPEPEPDSDSNQER.[K]	767957892	RNF20	56254	767957892 [125-142]	1xPhospho [S];2xTMT6plex [N-Term;K1]	S14(Phospho): 99.02	5	1.603	0.194421
[R].ESSPLYSPTFSDSTSAVK.[E]	153945728	MAP1B	4131	153945728 [1791-1808]	2xPhospho [S/Y/T];2xTMT6plex [N-Term;K18]	S3(Phospho): 98.19;Y6(Phospho): 49.53;S7(Phospho): 49.53	4	0.494	0.193850
[K].EKLQEEGGGSDEEETGSPSEDGMQSAR.[T]	5902034	PWP1	11137	5902034 [41-67]	1xOxidation [M23];1xPhospho [S10(100)];2xTMT6plex [N-Term;K2]	S10(Phospho): 99.99	3	0.506	0.193492
[K].RESEDDPESEPDDHEKR.[I]	530368018	GCFC2	6936	530368018 [180-196]	2xPhospho [S3(100);2xTMT6plex [N-Term;K16];S9(100)]	S3(Phospho): 100;S9(Phospho): 100	2	0.305	0.191980
[R].NVPQEESLEDSDVDADFK.[A]	34485722	KPNA3	3839	34485722 [50-67]	1xPhospho [S];2xTMT6plex [N-Term;K18]	S11(Phospho): 99.99	7	0.469	0.191975
[K].GVADDVVPPGPGPAPEAPAQEGPGSGSPR.[GDAV]	1370455314	PLCH2	9651	1370455314 [1130-1158]	1xPhospho [S];1xTMT6plex [N-Term]	S25(Phospho): 50;S27(Phospho): 50	2	0.953	0.189644
[R].YETPSDAIEVISPASSPAPPQEK.[L]	530410786	NCOR1	9611	530410786 [2050-2072]	2xPhospho [S/T/Y];2xTMT6plex [N-Term;K23]	S12(Phospho): 66.67;S15(Phospho): 66.67;S16(Phospho): 66.67	12	0.61	0.188707
[K].GPPQSPVFEGVYNNSR.[M]	530407501	ATXN2L	11273	530407501 [107-122]	1xPhospho [S5(100)];1xTMT6plex [N-Term]	S5(Phospho): 100	3	1.434	0.188144
[R].MDFAFPGSTNSLHR.[M]	578830853	ITGB4	3691	578830853 [1482-1495]	1xOxidation [M1];1xTMT6plex [N-Term];2xPhospho [S8(99.2);T9(98.2)]	S8(Phospho): 99.24;T9(Phospho): 50.38;S11(Phospho): 50.38	9	2.118	0.187767
[K].ATVTPSPVKGK.[G]	56118310	NUCKS1	64710	56118310 [176-186]	2xPhospho [T4(98);3xTMT6plex [N-Term;K11];K9;S6(100)]	T4(Phospho): 100;S6(Phospho): 100	3	0.255	0.185256
[R].DHSPTPSVFNSDEER.[Y]	530376841	FIP1L1	81608	530376841 [499-513]	1xTMT6plex [N-Term];2xPhospho [S3(100);S11(100)]	S3(Phospho): 100;S11(Phospho): 100	4	0.794	0.184275
[K].TLLLSSDDEF.[-]	1034628782	TMEM184B	25829	1034628782 [443-452]	1xTMT6plex [N-Term];2xPhospho [S5(100);S6(100)]	S5(Phospho): 100;S6(Phospho): 100	21	1.508	0.184092

Patil et al,2020. Proteomic and phosphoproteomic profiling of shammah-induced signaling in oral keratinocytes

Supplementary Table S2: List of phosphopeptides identified and quantified in OKF6/TERT1-Shammah and OKF6/TERT1-Parental phosphoproteome using TMT/TiO$_2$-based quantitative phosphoproteomic approach

Peptide Sequence	Protein Accessions	Gene Symbol	Gene ID	Positions in Proteins	Modifications	PhosphoRS: Best Site Probabilities	# PSMs	Fold change	p-value
[K].MPCESSPPESADTPTSTR.[R]	103472005	MKI67	4288	103472005 [1371-1388]	1xCarbamidomethyl [C3];1xOxidation [M1];1xPhospho [S/T];1xTMT6plex [N-Term]	S5(Phospho): 49.99;S6(Phospho): 49.99	2	1.107	0.183928
[R].SVSEINSDDELSGK.[G]	1394533392	TMF1	7110	1394533392 [338-351]	1xPhospho [S7(98.9)];2xTMT6plex [N-Term;K14]	S7(Phospho): 100	6	1.195	0.183726
[R].GSPHYFSPFRPY.[-]	4506903	SRSF9	8683	4506903 [210-221]	1xTMT6plex [N-Term];2xPhospho [S2(100);S7(99.9)]	S2(Phospho): 100;S7(Phospho): 99.93	7	1.58	0.183616
[K].AAAAAPASEDEDDEDDEDDEDDDDDEEDDSEEEAMETTPAK.[G]	55956788	NCL	4691	55956788 [177-217]	1xOxidation [M35];2xPhospho [S8(100);2xTMT6plex [N-Term;K41];S30(91.7)]	S8(Phospho): 100;S30(Phospho): 96.64	574	1.263	0.183030
[R].AHFNAMFQPSSPTR.[R]	530367499	GTF3C2	2976	530367499 [922-935]	1xOxidation [M6];1xPhospho [S/T];1xTMT6plex [N-Term]	S10(Phospho): 49.66;S11(Phospho): 49.66	7	0.627	0.182655
[K].ELQGDGPPSSPTNDPTVK.[Y]	530409847	CHD3	1107	530409847 [763-780]	1xPhospho [S/T];2xTMT6plex [N-Term;K18]	S9(Phospho): 33.33;S10(Phospho): 33.33;T12(Phospho): 33.33	9	0.696	0.182299
[K].SPQVLGSSLSVRSPTGSPSR.[L]	1034573119	BCL9L	283149	1034573119 [1023-1042]	1xTMT6plex [N-Term];2xPhospho [S13(97.2);S17(95)]	S13(Phospho): 97.22;S17(Phospho): 95	5	0.647	0.182065
[R].VLSTSSTLTR.[D]	578830853	ITGB4	3691	578830853 [1516-1525]	1xTMT6plex [N-Term];2xPhospho [S6(98.5);T7(98.5)]	S6(Phospho): 98.51;T7(Phospho): 98.51	8	1.579	0.181614
[R].TEGYAAFQEDSSGDEAESPSK.[M]	5803145	RALBP1	10928	5803145 [82-102]	2xPhospho [S11(100);2xTMT6plex [N-Term;K21];S12(100)]	S11(Phospho): 99.96;S12(Phospho): 99.96	3	0.718	0.181472
[R].EQSTPPKSPQ.[-]	1034567413	LARP4B	23185	1034567413 [1264-1273]	2xPhospho [S3(100);2xTMT6plex [N-Term;K7];S8(100)]	S3(Phospho): 99.99;S8(Phospho): 100	4	0.601	0.181307
[K].TKPTQAAGPSSPQKPPTPEETK.[A]	190684675	XRCC1	7515	190684675 [437-458]	2xPhospho [S/T];4xTMT6plex [N-Term;K14;K2;K22]	S10(Phospho): 66.64;S11(Phospho): 66.64;T17(Phospho): 66.64	7	0.893	0.180125
[K].AQTPPGPSLSGSKSPCPQEK.[S]	118572613	SRRM2	23524	118572613 [1001-1020]	1xCarbamidomethyl [C16];2xPhospho [T3(100);3xTMT6plex [N-Term;K13;K20];S8(100)]	T3(Phospho): 100;S12(Phospho): 49.5;S14(Phospho): 49.5	8	1.733	0.179746
[R].ASSLSESSPPK.[A]	767974553	LIMA1	51474	767974553 [368-378]	1xPhospho [S8(99.1)];2xTMT6plex [N-Term;K11]	S8(Phospho): 99.99	16	0.76	0.179002
[R].SQTPPGVATPPIPK.[I]	28559039	MED1	5469	28559039 [1049-1062]	1xPhospho [T3(94.2)];2xTMT6plex [N-Term;K14]	S1(Phospho): 49.66;T3(Phospho): 49.66	2	0.555	0.178530
[K].VSNSPEPQK.[A]	530413055	SPAG9	9043	530413055 [248-256]	1xPhospho [S4(100)];2xTMT6plex [N-Term;K9]	S4(Phospho): 99.99	5	0.402	0.176956
[K].ESAAPASPAPSPAPSPTPAPPQK.[E]	78042577	MAP7D1	55700	78042577 [538-560]	2xPhospho [S7(86.7);2xTMT6plex [N-Term;K23];S/T]	S7(Phospho): 97.32;S11(Phospho): 34.23;S15(Phospho): 34.23;T17(Phospho): 34.23	11	0.581	0.175237
[R].DAHDVSPTSTDTEAQLTVER.[Q]	156766050	AHNAK2	113146	156766050 [289-308]	1xTMT6plex [N-Term];2xPhospho [S6(100);T/S]	S6(Phospho): 99.94;T8(Phospho): 48.87;T10(Phospho): 48.9	4	0.694	0.173719
[R].SRTASGSSVTSLDGTR.[S]	767952381	NDRG1	10397	767952381 [343-358]	1xTMT6plex [N-Term];3xPhospho [S1(97.6);S5(100);T/S]	T3(Phospho): 97.64;S7(Phospho): 98.73;S8(Phospho): 50;T10(Phospho): 49.97	1	0.308	0.173276
[R].LLEGEDAHLSSSQFSSGSQSSR.[D]	15431310	KRT14	3861	15431310 [418-439]	1xTMT6plex [N-Term];2xPhospho [S]	S15(Phospho): 38.78;S16(Phospho): 38.78;S18(Phospho): 38.78;S20(Phospho): 38.78;S21(Phospho): 38.78	12	0.222	0.169635
[K].EGAASPAPETPQPTSPETSPK.[E]	7661690	CCDC9	26093	7661690 [372-392]	2xPhospho [S5(95.2);2xTMT6plex [N-Term;K21];T/S]	T10(Phospho): 26.03;T14(Phospho): 26.03;S15(Phospho): 26.03;T18(Phospho): 26.03;S19(Phospho): 95.88	2	0.432	0.166170
[K].VLIEDTDDEANT.[-]	19923272	XPR1	9213	19923272 [685-696]	1xPhospho [T6(98.7)];1xTMT6plex [N-Term]	T6(Phospho): 98.67	4	1.651	0.165599
[R].SSSVGSSSSYPISPAVSR.[T]	530389151	PLEC	5339	530389151 [4252-4269]	1xPhospho [S/Y];1xTMT6plex [N-Term]	S3(Phospho): 99.02	23	1.539	0.165304
[R].NYAGEEEEEGSGSSEGFDPPATDR.[Q]	224586882	YBX3	8531	224586882 [191-214]	1xPhospho [S/Y/T];1xTMT6plex [N-Term]	S11(Phospho): 33.04;S13(Phospho): 33.04;S14(Phospho): 33.04	9	0.65	0.163364
[R].LQSVQATGPSSPGR.[L]	1034644963	MCC	4163	1034644963 [474-487]	1xPhospho [T7(98.5)];1xTMT6plex [N-Term]	T7(Phospho): 98.47	7	0.672	0.162722
[R].FGQAATMEGIGAIGGTPPAFNR.[A]	34932414	NONO	4841	34932414 [435-456]	1xOxidation [M7];1xPhospho [T16(99.9)];1xTMT6plex [N-Term]	T16(Phospho): 99.97	2	0.693	0.162232
[R].SPSGGAAGPLLTPSQSLDGSR.[R]	19923973	KCTD12	115207	19923973 [185-205]	1xPhospho [S1(100)];1xTMT6plex [N-Term]	S1(Phospho): 99.98	4	0.496	0.160573
[R].AGGPTTPLSPTR.[L]	5031877	LMNB1	4001	5031877 [15-26]	1xTMT6plex [N-Term];2xPhospho [T11(98);T/S]	T5(Phospho): 50.01;T6(Phospho): 50.01;S9(Phospho): 49.99;T11(Phospho): 49.99	4	0.77	0.159664
[R].DCLIEDSDDEAGQS.[-]	972781663	CTU2	348180	972781663 [573-586]	1xCarbamidomethyl [C2];1xPhospho [S14(97.4)];1xTMT6plex [N-Term]	S14(Phospho): 98.9	6	0.535	0.159277
[R].SLDDSEEDDDEDSGHSSR.[R]	4505835	PKD2	5311	4505835 [808-825]	1xPhospho [S];1xTMT6plex [N-Term]	S5(Phospho): 97.88	1	0.429	0.159200
[K].AEDGATPSPSNETPK.[K]	153070260	MARCKS	4082	153070260 [138-152]	1xPhospho [S/T];2xTMT6plex [N-Term;K15]	S8(Phospho): 97.75	22	0.77	0.157560
[R].TQPDGTSVPGEPASPISQR.[L]	767968242	NUMA1	4926	767968242 [1762-1780]	1xPhospho [S14(94.8)];1xTMT6plex [N-Term]	S14(Phospho): 99.99	12	1.316	0.157179
[R].APPQTHLPSGASSGTGSASATHGGGSPPGTR.[G]	1034651584	BAG6	7917	1034651584 [88-118]	1xPhospho [S/T];1xTMT6plex [N-Term]	T21(Phospho): 85.27	3	0.601	0.156734
[K].SSSPAPADIAQTVQEDLR.[T]	38327552	G3BP1	10146	38327552 [230-247]	1xPhospho [S/T];1xTMT6plex [N-Term]	S1(Phospho): 33.33;S2(Phospho): 33.33;S3(Phospho): 33.33	6	0.487	0.155784
[R].DAHDVSPTSTDTEAQLTVER.[Q]	156766050	AHNAK2	113146	156766050 [289-308]	1xPhospho [S6(99.9)];1xTMT6plex [N-Term]	S6(Phospho): 99.94	1	0.504	0.154962
[R].MTLSPPHSPQGAAPR.[T]	767940349	TRERF1	55809	767940349 [631-645]	1xOxidation [M1];1xTMT6plex [N-Term];2xPhospho [S8(98.2);S/T]	T2(Phospho): 50;S4(Phospho): 50;S8(Phospho): 100	5	1.245	0.154604
[R].NSPQSSPTSTPK.[L]	767908139	IVNS1ABP	10625	767908139 [328-339]	2xPhospho [S2(100);2xTMT6plex [N-Term;K12];S/T]	S2(Phospho): 99.97;S5(Phospho): 48.54;S6(Phospho): 48.54	4	0.489	0.151417
[R].AGEEDEGEEDSDSDYEISAK.[A]	41393547	NBAS	51594	41393547 [463-482]	1xPhospho [S/Y];2xTMT6plex [N-Term;K20]	S11(Phospho): 33.33;S13(Phospho): 33.33;Y15(Phospho): 33.33	9	1.492	0.151029
[K].YQQITPVNQSR.[S]	530370525	MAP3K20	51776	530370525 [624-634]	1xPhospho [S10(98.7)];1xTMT6plex [N-Term]	T5(Phospho): 50;S10(Phospho): 50	4	0.625	0.149214
[K].NSQEDSEDSEDKDVK.[T]	56118310	NUCKS1	64710	56118310 [53-67]	2xPhospho [S6(100);3xTMT6plex [N-Term;K12;K15];S9(100)]	S6(Phospho): 100;S9(Phospho): 100	11	0.239	0.148835
[K].TPASTPVSGTPQASPMIER.[S]	32171238	BAIAP2L1	55971	32171238 [248-266]	1xOxidation [M16];1xPhospho [S4(96.4)];1xTMT6plex [N-Term]	T5(Phospho): 24.84;S8(Phospho): 24.84;T10(Phospho): 24.84;S14(Phospho): 24.84	3	0.51	0.148527
[K].LGASNSPGQPNSVK.[R]	55741709	RBM25	58517	55741709 [672-685]	1xPhospho [S6(99.2)];2xTMT6plex [N-Term;K14]	S6(Phospho): 99.99	7	0.743	0.146492
[R].LIPGSNLDSEPR.[I]	29826289	CASC4	113201	29826289 [324-335]	1xTMT6plex [N-Term];2xPhospho [S5(100);S9(100)]	S5(Phospho): 100;S9(Phospho): 100	15	0.837	0.145615
[K].SHVEDGDIAGAPASSPEAPPAEQDPVQLK.[T]	110611218	RRBP1	6238	110611218 [830-858]	1xPhospho [S];2xTMT6plex [N-Term;K29]	S14(Phospho): 50;S15(Phospho): 50	6	1.501	0.145238
[R].RSSQPSPTAVPASDSPPTK.[Q]	78042577	MAP7D1	55700	78042577 [111-129]	1xPhospho [S/T];2xTMT6plex [N-Term;K19]	S2(Phospho): 50;S3(Phospho): 50	13	0.53	0.145217
[R].VAGEAAETDSEPEPEPEPTAAPR.[D]	47086495	BLOC1S3	388552	47086495 [56-78]	1xTMT6plex [N-Term];2xPhospho [T8(100);S10(100)]	T8(Phospho): 100;S10(Phospho): 100	10	0.453	0.143403
[R].SSSPVTELASRSPIR.[Q]	118572613	SRRM2	23524	118572613 [1101-1115]	1xTMT6plex [N-Term];2xPhospho [S3(97.3);T/S]	S2(Phospho): 49.44;S3(Phospho): 49.44;T6(Phospho): 100	3	1.231	0.142135
[R].VVPGQFDDADSSDSENR.[D]	23510356	RIOK1	83732	23510356 [11-27]	1xTMT6plex [N-Term];2xPhospho [S]	S11(Phospho): 66.67;S12(Phospho): 66.67;S14(Phospho): 66.67	2	0.474	0.141211
[K].SDGSDTTEQESTNLPSPNK.[S]	390979647	CRYBG3	131544	390979647 [442-460]	1xPhospho [S16(96.7)];2xTMT6plex [N-Term;K19]	S16(Phospho): 96.67	5	0.858	0.140442
[K].ICTEQSNSPPPIR.[R]	1370454062	PTPN14	5784	1370454062 [307-319]	1xCarbamidomethyl [C2];1xPhospho [S/T];1xTMT6plex [N-Term]	S6(Phospho): 50;S8(Phospho): 50	5	0.662	0.139523
[R].KGSPTPGFSTR.[R]	1034659998	FAM83H	286077	1034659998 [1080-1090]	1xPhospho [S/T];2xTMT6plex [N-Term;K1]	S3(Phospho): 50;T5(Phospho): 50	3	1.238	0.137965
[R].ASDPQSPPQVSR.[H]	45439370	TAOK1	57551	45439370 [416-427]	1xPhospho [S6(100)];1xTMT6plex [N-Term]	S6(Phospho): 100	3	0.741	0.137524
[R].SSSVGSSSSYPISPAVSR.[T]	530389151	PLEC	5339	530389151 [4252-4269]	1xTMT6plex [N-Term];2xPhospho [S/Y]	S2(Phospho): 49.99;S3(Phospho): 49.99;S6(Phospho): 99.98	43	1.493	0.137073
[R].SSSSPPPKQK.[S]	118572613	SRRM2	23524	118572613 [817-826]	2xPhospho [S2(98.4);3xTMT6plex [N-Term;K10;K8;S]	S2(Phospho): 97.69;S4(Phospho): 98.84	5	1.484	0.135203
[R].YQPLASTASDNDFVTPEPR.[R]	178056552	NCAPD2	9918	178056552 [1325-1343]	1xTMT6plex [N-Term];2xPhospho [S/T/Y]	S6(Phospho): 65.77;T7(Phospho): 65.77;S9(Phospho): 65.77	3	0.535	0.134962
[K].SSQQPSTPQQAPPGQPQQGTFVAHK.[E]	661567300	ZC3H18	124245	661567300 [814-838]	1xPhospho [S/T];2xTMT6plex [N-Term;K25]	T7(Phospho): 97.93	13	1.145	0.133888
[K].SGTAETEPVEQDSSQPSLPLVR.[A]	213972636	TP53BP1	7158	213972636 [823-844]	1xPhospho [S/T];1xTMT6plex [N-Term]	S13(Phospho): 49.98;S14(Phospho): 49.98	15	1.823	0.133050
[K].ESSPIPSPTSDRK.[A]	530368012	SPTBN1	6711	530368012 [2163-2175]	2xPhospho [S/T];2xTMT6plex [N-Term;K13]	S3(Phospho): 98.11;S7(Phospho): 100	3	0.853	0.128087
[R].LIIESPSNTSSTEPA.[-]	4758024	COIL	8161	4758024 [562-576]	1xTMT6plex [N-Term];2xPhospho [S5(99.9);S/T]	S5(Phospho): 99.89;S7(Phospho): 94.84	7	1.381	0.127390
[R].SSDGSLSHEEDLAK.[V]	578821804	PPFIA1	8500	578821804 [238-251]	2xPhospho [S];2xTMT6plex [N-Term;K14]	S1(Phospho): 50;S2(Phospho): 50;S5(Phospho): 50;S7(Phospho): 50	6	0.494	0.126495
[R].KNSTDLDSAPEDPTSPK.[R]	756140883	CIC	23152	756140883 [2304-2320]	2xPhospho [S/T];3xTMT6plex [N-Term;K1;K17]	S3(Phospho): 50;T4(Phospho): 50;S8(Phospho): 49.99;S15(Phospho): 49.99	5	1.959	0.126342
[R].GFSANSARLPGVSR.[S]	119703753;5031839	KRT6B;KRT6A	3854;3853	119703753 [17-30];5031839 [17-30]	1xTMT6plex [N-Term];2xPhospho [S3(100);S6(100)]	S3(Phospho): 100;S6(Phospho): 100	8	2.111	0.124908
[R].RLSESSALKQPATPTAAESSEGEGEEGDDGGETESR.[E]	18426902	WRNIP1	56897	18426902 [73-108]	2xPhospho [S/T];2xTMT6plex [N-Term;K9]	S3(Phospho): 33.32;S5(Phospho): 33.32;S6(Phospho): 33.32;T13(Phospho): 25.01;T15(Phospho): 25.01;S19(Phospho): 25.01;S20(Phospho): 25.01	3	3.46	0.123927
[R].NYAGEEEEEGSGSSEGFDPPATDR.[Q]	224586882	YBX3	8531	224586882 [191-214]	1xTMT6plex [N-Term];3xPhospho [S/T/Y]	S11(Phospho): 100;S13(Phospho): 100;S14(Phospho): 100	3	0.187	0.123030
[R].TPLSFTNPLHSDDSDSDER.[N]	196114951	PTPN12	5782	196114951 [593-611]	1xTMT6plex [N-Term];2xPhospho [S11(94.8);S/T]	S11(Phospho): 66.67;S14(Phospho): 66.67;S16(Phospho): 66.67	3	0.01	0.121642
[R].TRTPASINATPANINLADLTR.[A]	27597090	SUPT6H	6830	27597090 [1530-1550]	1xTMT6plex [N-Term];2xPhospho [S6(100);T]	T1(Phospho): 50;T3(Phospho): 50;S6(Phospho): 100	3	1.458	0.121125
[R].VVPGQFDDADSSDSENR.[D]	23510356	RIOK1	83732	23510356 [11-27]	1xPhospho [S];1xTMT6plex [N-Term]	S12(Phospho): 98.58	11	0.332	0.120640
[R].SSGSPYGGGYGSGGGSGGYGSR.[R]	1057503215	HNRNPA3	220988	1057503215 [355-376]	1xPhospho [S/Y];1xTMT6plex [N-Term]	S1(Phospho): 33.33;S2(Phospho): 33.33;S4(Phospho): 33.33	10	1.339	0.120305
[K].AVAGVMITASHNR.[K]	767967976	PGM2L1	283209	767967976 [187-199]	1xOxidation [M6];1xPhospho [S10(99)];1xTMT6plex [N-Term]	S10(Phospho): 100	10	0.699	0.119410
[R].RGDSESEEDEQDSEEVR.[LA]	7706557	C9orf78	51759	7706557 [12-28]	1xTMT6plex [N-Term];2xPhospho [S4(100);S6(98)]	S6(Phospho): 98.01;S13(Phospho): 98.01	3	0.396	0.119265
[R].ESDKEEPVTPTSGGGPMSPQDDEAEEESDNELSSGTGDVSK.[D]	530365778	RABGAP1L	9910	530365778 [463-503]	1xOxidation [M17];1xPhospho [S/T];3xTMT6plex [N-Term;K4;K41]	S18(Phospho): 47.02;S28(Phospho): 47.02	2	0.19	0.118921
[R].TEAQDLCRASPEPPGPESSSR.[W]	578821824	TNKS1BP1	85456	578821824 [663-683]	1xCarbamidomethyl [C7];1xPhospho [S10(100)];1xTMT6plex [N-Term]	S10(Phospho): 100	3	0.756	0.117195
[R].ASSRSPVFTPR.[V]	1034671241	VAV2	7410	1034671241 [795-805]	1xTMT6plex [N-Term];2xPhospho [S5(100);S/T]	S2(Phospho): 50;S3(Phospho): 50;S5(Phospho): 99.99	11	0.537	0.117162
[R].TDSCSSAQAQYDTPK.[A]	54792129	DCBLD2	131566	54792129 [722-736]	1xCarbamidomethyl [C4];2xPhospho [S/Y/T];2xTMT6plex [N-Term;K15]	S3(Phospho): 96.61;S5(Phospho): 50.53;S6(Phospho): 50.53	5	1.218	0.116954
[R].AAAGSPRSPGPLPGAR.[G]	1034591603	ISL2	64843	1034591603 [150-165]	1xTMT6plex [N-Term];2xPhospho [S5(100);S8(100)]	S5(Phospho): 100;S8(Phospho): 100	3	1.632	0.112867
[R].NVSSFPDDATSPLQENR.[N]	1002341781	KPNA2	3838	1002341781 [52-68]	1xPhospho [T/S];1xTMT6plex [N-Term]	T10(Phospho): 50;S11(Phospho): 50	10	0.469	0.111517
[K].KETESEAEDNLDDLEK.[H]	1034554585	SRRM1	10250	1034554585 [909-924]	2xPhospho [T3(100);3xTMT6plex [N-Term;K1;K16];S5(100)]	T3(Phospho): 100;S5(Phospho): 100	10	0.757	0.109929
[R].SQSDLDDQHDYDSVASDEDTDQEPLR.[S]	1370470787	GIT1	28964	1370470787 [414-439]	1xTMT6plex [N-Term];3xPhospho [S16(99.7);T20(99.7);Y/S]	Y11(Phospho): 50.23;S13(Phospho): 50.23;S16(Phospho): 99.74;T20(Phospho): 99.74	6	0.323	0.107674
[K].EIPSATQSPISK.[K]	157694494	MYBBP1A	10514	157694494 [1156-1167]	2xPhospho [S4(99.2);2xTMT6plex [N-Term;K12];T/S]	S4(Phospho): 99.23;T6(Phospho): 50.38;S8(Phospho): 50.38	3	0.53	0.107024
[R].LGSLSARSDSEATISR.[S]	578802413	CEP170	9859	578802413 [1220-1235]	1xTMT6plex [N-Term];2xPhospho [S3(100);S/T]	S3(Phospho): 99.99;S5(Phospho): 49.66;S8(Phospho): 49.66	5	0.487	0.106760
[R].GSLASLDSLRK.[G]	146231940	CTNND1	1500	146231940 [345-355]	2xPhospho [S5(100);2xTMT6plex [N-Term;K11];S8(100)]	S5(Phospho): 100;S8(Phospho): 100	11	1.841	0.106038
[R].TNSTGGSSGSSVGGGSGK.[T]	1034578688	ERC1	23085	1034578688 [35-52]	1xPhospho [T/S];2xTMT6plex [N-Term;K18]	T1(Phospho): 49.78;S3(Phospho): 49.78	8	0.234	0.103923
[K].KPAQEETEETSSQESAEED.[-]	4504431	HMGA2	8091	4504431 [91-109]	1xPhospho [S/T];2xTMT6plex [N-Term;K1]	T10(Phospho): 96.69	16	1.619	0.103608
[K].GGNLPPVSPNDSGAK.[I]	530372165	FAM208A	23272	530372165 [1096-1110]	1xPhospho [S8(100)];2xTMT6plex [N-Term;K15]	S8(Phospho): 100	4	1.813	0.103199
[K].ASSPHGLGSPLVASPR.[L]	157738639	TNS4	84951	157738639 [240-255]	1xTMT6plex [N-Term];2xPhospho [S9(100);S14(100)]	S2(Phospho): 50;S3(Phospho): 50;S9(Phospho): 100	11	0.847	0.102459
[K].GQDTVAIEGFTDEEDTESGGEGQYR.[E]	1034575511	QSER1	79832	1034575511 [1460-1484]	1xTMT6plex [N-Term];2xPhospho [T/S/Y]	T11(Phospho): 98.17;T16(Phospho): 50.87;S18(Phospho): 50.87	3	1.467	0.102173
[R].GEGDAPFSEPGTTSTQRPSSPETATK.[Q]	118572613	SRRM2	23524	118572613 [304-329]	1xPhospho [T/S];2xTMT6plex [N-Term;K26]	S20(Phospho): 91.06	25	1.573	0.100087
[R].NLALDEAGQRSTM.[-]	188536036	CYBRD1	79901	188536036 [274-286]	1xOxidation [M13];1xPhospho [T/S];1xTMT6plex [N-Term]	S11(Phospho): 50;T12(Phospho): 50	10	1.965	0.098959
[R].TSMGGTQQQFVEGVR.[M]	1370483230	CTNNB1	1499	1370483230 [551-565]	1xOxidation [M3];1xPhospho [S2(98.4)];1xTMT6plex [N-Term]	S2(Phospho): 98.93	8	1.589	0.098897
[R].RYSPSPPPKR.[R]	1034554585	SRRM1	10250	1034554585 [642-651]	2xPhospho [S5(100);2xTMT6plex [N-Term;K9];Y/S]	S3(Phospho): 98.72;S5(Phospho): 99.98	9	1.661	0.098130
[R].RALVEFESNPEETREPGSPPSVQR.[A]	13129104	CCDC86	79080	13129104 [30-53]	1xPhospho [T/S];1xTMT6plex [N-Term]	T13(Phospho): 49.93;S18(Phospho): 49.93	5	0.783	0.097438
[R].SSTPLHSPSPIR.[V]	14043024	BAG3	9531	14043024 [283-294]	1xTMT6plex [N-Term];2xPhospho [S7(97.9);S/T]	T3(Phospho): 98.18;S7(Phospho): 98.18	3	0.245	0.097174
[R].AAAAGLGHPASPGGSEDGPPGSEEEDAAR.[E]	4885193	ARID3A	1820	4885193 [67-95]	1xTMT6plex [N-Term];2xPhospho [S15(98.3);S]	S11(Phospho): 50;S15(Phospho): 50;S22(Phospho): 100	8	1.388	0.096652
[K].SLDSDESEDEEDDYQQKR.[K]	7657441	PDAP1	11333	7657441 [57-74]	2xPhospho [S4(99.9);2xTMT6plex [N-Term;K17];S7(99.9)]	S4(Phospho): 100;S7(Phospho): 100	4	0.418	0.096478
[K].LQEEGGGSDEEETGSPSEDGMQSAR.[T]	5902034	PWP1	11137	5902034 [43-67]	1xOxidation [M21];1xTMT6plex [N-Term];2xPhospho [S8(98.7);T/S]	S8(Phospho): 98.66;T13(Phospho): 50.67;S15(Phospho): 50.67	3	0.624	0.096413
[K].NLDPDPEPPSPDSPTETFAAPAEVR.[H]	767967520	SPINDOC	144097	767967520 [239-263]	1xTMT6plex [N-Term];2xPhospho [T15(93);T17(93)]	T15(Phospho): 92.98;T17(Phospho): 92.98	3	1.597	0.095729
[R].KFSKEEPVSSGPEEAVGK.[S]	32483374	NOP56	10528	32483374 [561-578]	2xPhospho [S9(99.8);4xTMT6plex [N-Term;K1;K18];K4;S10(99.8)]	S9(Phospho): 100;S10(Phospho): 100	4	0.508	0.094831
[R].ERSPSPPTIQSK.[R]	1034594073	GSE1	23199	1034594073 [1532-1543]	2xPhospho [S3(100);2xTMT6plex [N-Term;K12];S5(100)]	S3(Phospho): 100;S5(Phospho): 100	3	1.158	0.089697
[K].SATRPSPSPERSSTGPEPPAPTPLLAER.[H]	118572613	SRRM2	23524	118572613 [346-373]	1xTMT6plex [N-Term];2xPhospho [S6(93.4);S8(87.3)]	S6(Phospho): 99.93;S8(Phospho): 99.99	9	2.795	0.089434
[R].APQTSSSPPPVR.[R]	1034554585	SRRM1	10250	1034554585 [729-740]	1xTMT6plex [N-Term];2xPhospho [S/T]	S6(Phospho): 97.38;S7(Phospho): 99.94	22	1.69	0.088258
[R].ASVRGSSEEDEDARTPDR.[A]	31044432	LEMD2	221496	31044432 [133-150]	1xTMT6plex [N-Term];2xPhospho [S6(100);S7(100)]	S6(Phospho): 100;S7(Phospho): 100	3	1.423	0.088066
[R].TSSKESSPIPSPTSDR.[K]	530368012	SPTBN1	6711	530368012 [2159-2174]	2xPhospho [S6(98.3);2xTMT6plex [N-Term;K4];S11(96.8)]	S6(Phospho): 98.33;S11(Phospho): 96.78	8	1.979	0.087819
[R].APSPPVEHPR.[L]	1034567534	PROSER2	254427	1034567534 [177-186]	1xPhospho [S3(100)];1xTMT6plex [N-Term]	S3(Phospho): 100	3	0.525	0.087560
[R].LTGSQLSITQVASIK.[Q]	1034563451	RASAL2	9462	1034563451 [1010-1024]	2xPhospho [T2(98.4);2xTMT6plex [N-Term;K15];S7(96.7)]	T2(Phospho): 98.95;S4(Phospho): 50.51;S7(Phospho): 50.51	4	0.617	0.086731
[R].GQVIISDSDDDDDER.[I]	530427120	SETX	23064	530427120 [1011-1026]	1xTMT6plex [N-Term];2xPhospho [S7(100);S9(100)]	S7(Phospho): 100;S9(Phospho): 100	3	0.502	0.084215
[K].GASAATGIPLESDEDSNDNDNDIENENCMHTN.[-]	355330276	USP15	9958	355330276 [950-981]	1xCarbamidomethyl [C28];1xOxidation [M29];1xPhospho [S/T];1xTMT6plex [N-Term]	S12(Phospho): 49.24;S16(Phospho): 49.24	9	0.453	0.083888
[R].GRESDEDTEDASETDLAK.[H]	767975125	SUDS3	64426	767975125 [121-138]	2xTMT6plex [N-Term;3xPhospho [S4(100);K18];S12(97.2)];T8(100)]	S4(Phospho): 100;T8(Phospho): 99.93;S12(Phospho): 99.93	2	0.297	0.082673
[K].EGQPSPADEKGNDSDGEGESDDPEKK.[K]	17865802	VPS4B	9525	17865802 [89-114]	1xPhospho [S14(97)];4xTMT6plex [N-Term;K10;K25;K26]	S5(Phospho): 49.97;S14(Phospho): 49.97	5	0.473	0.082282
[R].ALSRQLSSGVSEIR.[H]	4504517	HSPB1	3315	4504517 [76-89]	1xTMT6plex [N-Term];2xPhospho [S3(99);S]	S3(Phospho): 100;S7(Phospho): 99.99	3	1.227	0.082237
[R].RSSQPSPTAVPASDSPPTK.[Q]	78042577	MAP7D1	55700	78042577 [111-129]	2xPhospho [S6(100);2xTMT6plex [N-Term;K19];S/T]	S2(Phospho): 98.4;S6(Phospho): 100	8	0.418	0.081332
[R].EQSTPPKSPQ.[-]	1034567413	LARP4B	23185	1034567413 [1264-1273]	1xPhospho [T/S];2xTMT6plex [N-Term;K7]	S8(Phospho): 100	7	0.41	0.080498
[R].AASSAAQGAFQGN.[-]	1003088831	SCAMP2	10066	1003088831 [360-372]	1xPhospho [S4(99.3)];1xTMT6plex [N-Term]	S4(Phospho): 99.34	4	1.615	0.080370
[R].IQEQESSGEEDSDLSPEEREK.[K]	1186517946	PPP1R2B	153743	1186517946 [116-136]	2xPhospho [S6(98);2xTMT6plex [N-Term;K21];S7(98)]	S6(Phospho): 98.05;S7(Phospho): 98.05	5	0.69	0.078588
[K].KPAQEETEETSSQESAEED.[-]	4504431	HMGA2	8091	4504431 [91-109]	2xTMT6plex [N-Term;3xPhospho [T/S];K1]	T10(Phospho): 98.66;S11(Phospho): 50.69;S12(Phospho): 50.69;S15(Phospho): 99.96	6	0.851	0.077643
[K].SHSSPSLNPDTSPITAK.[V]	1034659763	PSD3	23362	1034659763 [1028-1044]	2xPhospho [S3(96.5);2xTMT6plex [N-Term;K17];S6(94.8)]	S3(Phospho): 98.51;T11(Phospho): 49.99;S12(Phospho): 49.99	4	0.81	0.077082
[K].LTPAEEENNSFQRLSP.[-]	1394533216	MTX3	345778	1394533216 [297-312]	1xPhospho [S/T];1xTMT6plex [N-Term]	S15(Phospho): 98.23	1	1.193	0.076970
[R].ASTASPCNNNINAATAVALQEPR.[K]	767972993	LARP4	113251	767972993 [601-623]	1xCarbamidomethyl [C7];1xPhospho [S5(100)];1xTMT6plex [N-Term]	S5(Phospho): 99.97	3	0.705	0.076544
[R].LPLVPESPRR.[M]	66348045	ZC3HC1	51530	66348045 [315-324]	1xPhospho [S7(100)];1xTMT6plex [N-Term]	S7(Phospho): 100	1	0.57	0.076295
[R].DVDDGSGSPHSPHQLSSK.[S]	767971427	WNK1	65125	767971427 [2435-2452]	2xPhospho [S8(98.8);2xTMT6plex [N-Term;K18];S11(98.8)]	S6(Phospho): 50;S8(Phospho): 50;S11(Phospho): 100	7	0.451	0.076193
[R].EEGPPPPSPDGASSDAEPEPPSGR.[T]	767925441	SMC4	10051	767925441 [15-38]	1xTMT6plex [N-Term];2xPhospho [S8(99);S]	S8(Phospho): 98.95;S13(Phospho): 50.52;S14(Phospho): 50.52	5	0.583	0.075649
[R].RSPSVSSPEPAEK.[S]	118572613	SRRM2	23524	118572613 [1726-1738]	2xPhospho [S6(96.5);2xTMT6plex [N-Term;K13];S7(99.9)]	S2(Phospho): 99.99;S4(Phospho): 33.34;S6(Phospho): 33.33;S7(Phospho): 33.33	5	2.087	0.074881
[R].KPSPAASPATK.[K]	530420479	MICALL1	85377	530420479 [638-648]	2xPhospho [S3(100);3xTMT6plex [N-Term;K1;K11];S7(100)]	S3(Phospho): 100;S7(Phospho): 99.99	4	0.348	0.074796
[K].KAASPSPQSVR.[R]	1034554585	SRRM1	10250	1034554585 [774-784]	2xPhospho [S4(100);2xTMT6plex [N-Term;K1];S]	S4(Phospho): 100;S6(Phospho): 98.23	17	1.529	0.074344
[K].DEILPTTPISEQK.[G]	386869503	RPS3	6188	386869503 [231-243]	1xPhospho [T/S];2xTMT6plex [N-Term;K13]	T6(Phospho): 49.61;T7(Phospho): 49.61	4	0.686	0.073692
[K].LTGSTSSLNK.[L]	530376797	EXOC1	55763	530376797 [485-494]	1xPhospho [S/T];2xTMT6plex [N-Term;K10]	S7(Phospho): 99.31	6	0.443	0.072697
[R].VSIRLPSTSGSEGVPFR.[T]	32967311	EPHA2	1969	32967311 [891-907]	1xTMT6plex [N-Term];2xPhospho [S/T]	S7(Phospho): 50;T8(Phospho): 50;S9(Phospho): 50;S11(Phospho): 50	5	6.931	0.072539
[K].YVSGSSPDLVTR.[K]	1370454062	PTPN14	5784	1370454062 [589-600]	1xTMT6plex [N-Term];2xPhospho [S5(98.3);S6(98.3)]	Y1(Phospho): 74.49;S3(Phospho): 25.51;S5(Phospho): 50;S6(Phospho): 50	11	0.605	0.072406
[R].HCAPSPDRSPELSSSR.[D]	47933339	RBM15	64783	47933339 [666-681]	1xCarbamidomethyl [C2];1xTMT6plex [N-Term];2xPhospho [S5(100);S]	S5(Phospho): 100;S9(Phospho): 100	9	1.452	0.069600
[R].VAAAAGSGPSPPGSPGHDRER.[Q]	13559514	PI4K2A	55361	13559514 [38-58]	1xPhospho [S];1xTMT6plex [N-Term]	S10(Phospho): 99.98	10	0.675	0.069332
[R].SDEGQLSPATR.[G]	530389151	PLEC	5339	530389151 [582-592]	1xPhospho [S7(100)];1xTMT6plex [N-Term]	S7(Phospho): 100	3	1.702	0.068975
[R].LNASPAAREEATSPGAK.[D]	578837375	TCF20	6942	578837375 [571-587]	2xPhospho [S4(100);2xTMT6plex [N-Term;K17];T/S]	S4(Phospho): 98.86;T12(Phospho): 50.57;S13(Phospho): 50.57	8	2.256	0.068200
[R].KLSGDQPAARTPR.[S]	530380665	TCOF1	6949	530380665 [1386-1398]	2xPhospho [S3(100);2xTMT6plex [N-Term;K1];T11(100)]	S3(Phospho): 100;T11(Phospho): 100	4	0.322	0.067817
[K].KEESEESDDDMGFGLFD.[-]	4506671	RPLP2	6181	4506671 [99-115]	1xOxidation [M11];2xPhospho [S4(100);2xTMT6plex [N-Term;K1];S7(100)]	S4(Phospho): 100;S7(Phospho): 100	12	0.406	0.067707
[K].AASLDEQTMFYGSVKTGYTIGYGLSLATLLVATAILSLFRK.[L]	530373089	VIPR1	7433	530373089 [128-168]	1xPhospho [S37(95)];3xTMT6plex [N-Term;K15;K41]	S37(Phospho): 55.49	2	2.358	0.067682
[R].AGSPFSPPPSSSSLTGEAAISR.[S]	311771772	PDLIM2	64236	311771772 [382-403]	1xTMT6plex [N-Term];2xPhospho [S3(100);S6(100)]	S3(Phospho): 100;S6(Phospho): 100	3	0.643	0.067316
[R].NHNTDDSDDFYD.[-]	118150654	KIZ	55857	118150654 [662-673]	1xTMT6plex [N-Term];2xPhospho [T4(98.6);S/Y]	T4(Phospho): 99.98;S7(Phospho): 50.01;Y11(Phospho): 50.01	2	0.532	0.067023
[R].TASPPPPPK.[R]	1034554585	SRRM1	10250	1034554585 [653-661]	2xPhospho [T1(100);2xTMT6plex [N-Term;K9];S3(100)]	T1(Phospho): 100;S3(Phospho): 100	23	1.487	0.066204
[K].EVDGLLTSEPMGSPVSSK.[T]	530407501	ATXN2L	11273	530407501 [588-605]	1xOxidation [M11];1xPhospho [S13(94.8)];2xTMT6plex [N-Term;K18]	S13(Phospho): 94.75	7	0.536	0.064285
[K].SVESTSPEPSK.[I]	520261969	SON	6651	520261969 [278-288]	1xPhospho [T/S];2xTMT6plex [N-Term;K11]	T5(Phospho): 49.76;S6(Phospho): 49.76	8	1.756	0.063167
[R].SHTSEGAHLDITPNSGAAGNSAGPK.[S]	767952381	NDRG1	10397	767952381 [381-405]	1xPhospho [S/T];2xTMT6plex [N-Term;K25]	S1(Phospho): 33.33;T3(Phospho): 33.33;S4(Phospho): 33.33	8	0.744	0.063097
[R].TDSREDEISPPPPNPVVK.[G]	47132581	PRKAR1A	5573	47132581 [75-92]	2xPhospho [S3(98.7);2xTMT6plex [N-Term;K18];S9(100)]	S3(Phospho): 98.69;S9(Phospho): 100	4	0.635	0.062708
[R].RLSPSASPPR.[R]	1034554585	SRRM1	10250	1034554585 [412-421]	1xTMT6plex [N-Term];2xPhospho [S3(100);S7(100)]	S3(Phospho): 100;S5(Phospho): 98.93	11	1.707	0.062550
[R].LESSDLTPPHSPPPSSR.[Q]	768010618	PLEKHG2	64857	768010618 [1252-1268]	1xTMT6plex [N-Term];2xPhospho [T7(96.3);S11(96.3)]	T7(Phospho): 96.29;S11(Phospho): 96.29	2	0.613	0.062007
[R].LIHGEDSDSEGEEEGR.[G]	1370471860	UBE2O	63893	1370471860 [81-96]	1xTMT6plex [N-Term];2xPhospho [S7(100);S9(100)]	S7(Phospho): 100;S9(Phospho): 100	3	0.769	0.061880
[K].LQTPNTFPK.[R]	530394600	NOLC1	9221	530394600 [616-624]	2xPhospho [T3(100);2xTMT6plex [N-Term;K9];T6(100)]	T3(Phospho): 100;T6(Phospho): 100	2	0.222	0.061324
[R].GAPSSPATGVLPSPQGK.[S]	62955803	NUP188	23511	62955803 [1705-1721]	2xPhospho [S13(100);2xTMT6plex [N-Term;K17];S/T]	S4(Phospho): 49.6;S5(Phospho): 49.6;S13(Phospho): 99.96	5	1.865	0.060571
[R].APSDSSLGTPSDGRPELR.[G]	578833732	TRIP10	9322	578833732 [294-311]	1xTMT6plex [N-Term];2xPhospho [S3(96.4);S/T]	S3(Phospho): 96.37;S11(Phospho): 99.89	18	0.389	0.059818
[K].IDDRDSDEEGASDR.[R]	164419743	DDX54	79039	164419743 [777-790]	1xPhospho [S];1xTMT6plex [N-Term]	S6(Phospho): 100	3	1.233	0.059781
[R].NYQQNYQNSESGEKNEGSESAPEGQAQQR.[R]	34098946	YBX1	4904	34098946 [157-185]	1xPhospho [S18(99.9)];2xTMT6plex [N-Term;K14]	S18(Phospho): 99.93	39	0.673	0.058832
[K].LTPLPEDNSMNVDQDGDPSDR.[M]	13654237	PRKDC	5591	13654237 [3197-3217]	1xOxidation [M10];1xPhospho [S/T];1xTMT6plex [N-Term]	S9(Phospho): 100	5	2.145	0.058735
[R].VSSPLPSPSAMTDAANSQAAAK.[L]	530365128	GATAD2B	57459	530365128 [332-353]	1xOxidation [M11];2xPhospho [S3(97.8);2xTMT6plex [N-Term;K22];S17(99.8)]	S3(Phospho): 97.84;S17(Phospho): 99.84	3	1.573	0.057514
[K].SDGAPASDSKPGSSEAAPSSK.[E]	30795231	BASP1	10409	30795231 [164-184]	2xPhospho [S];3xTMT6plex [N-Term;K10;K21]	S13(Phospho): 84.68;S14(Phospho): 84.68	3	2.153	0.057172
[K].KEDSDEEEDDDSEEDEEDDEDEDEDEIEPAAMK.[A]	55956788	NCL	4691	55956788 [142-176]	1xOxidation [M34];2xPhospho [S4(100);3xTMT6plex [N-Term;K1;K35];S12(100)]	S4(Phospho): 100;S12(Phospho): 100	23	0.868	0.056070
[K].ADRDQSPFSK.[I]	530371189	PPIG	9360	530371189 [682-691]	1xPhospho [S6(100)];2xTMT6plex [N-Term;K10]	S6(Phospho): 100	3	1.37	0.053891
[R].YSCPESPSQELGGPLPSPGPR.[L]	1417435944	MPRIP	23164	1417435944 [332-352]	1xCarbamidomethyl [C3];1xTMT6plex [N-Term];2xPhospho [S6(96.9);S17(99.9)]	S6(Phospho): 99.75;S17(Phospho): 100	3	1.746	0.052650
[R].LAEDEGDSEPEAVGQSR.[G]	1034657353	BAZ1B	9031	1034657353 [1461-1477]	1xPhospho [S8(100)];1xTMT6plex [N-Term]	S8(Phospho): 100	6	0.711	0.051161
[R].SFSTASAITPSVSR.[T]	119395754	KRT5	3852	119395754 [16-29]	1xPhospho [S3(97.8)];1xTMT6plex [N-Term]	S6(Phospho): 100	8	2.157	0.050939
